# Pervasive microRNA Duplication in Chelicerates: Insights from the Embryonic microRNA Repertoire of the Spider *Parasteatoda tepidariorum*

**DOI:** 10.1093/gbe/evw143

**Published:** 2016-06-19

**Authors:** Daniel J. Leite, Maria Ninova, Maarten Hilbrant, Saad Arif, Sam Griffiths-Jones, Matthew Ronshaugen, Alistair P. McGregor

**Affiliations:** 1 Department of Biological and Medical Sciences, Oxford Brookes University, Gipsy Lane, Oxford, OX3 0BP, United Kingdom; 2 Faculty of Life Sciences, University of Manchester, United Kingdom; 1 Department of Biological and Medical Sciences, Oxford Brookes University, Gipsy Lane, Oxford, OX3 0BP, United Kingdom; 1 Department of Biological and Medical Sciences, Oxford Brookes University, Gipsy Lane, Oxford, OX3 0BP, United Kingdom; 2 Faculty of Life Sciences, University of Manchester, United Kingdom; 2 Faculty of Life Sciences, University of Manchester, United Kingdom; 1 Department of Biological and Medical Sciences, Oxford Brookes University, Gipsy Lane, Oxford, OX3 0BP, United Kingdom

**Keywords:** *Parasteatoda tepidariorum*, spiders, Cheliceratal, microRNA, gene duplication, arm usage

## Abstract

MicroRNAs are small (∼22 nt) noncoding RNAs that repress translation and therefore regulate the production of proteins from specific target mRNAs. microRNAs have been found to function in diverse aspects of gene regulation within animal development and many other processes. Among invertebrates, both conserved and novel, lineage specific, microRNAs have been extensively studied predominantly in holometabolous insects such as *Drosophila melanogaster* . However little is known about microRNA repertoires in other arthropod lineages such as the chelicerates. To understand the evolution of microRNAs in this poorly sampled subphylum, we characterized the microRNA repertoire expressed during embryogenesis of the common house spider *Parasteatoda tepidariorum* . We identified a total of 148 microRNAs in *P. tepidariorum* representing 66 families. Approximately half of these microRNA families are conserved in other metazoans, while the remainder are specific to this spider. Of the 35 conserved microRNAs families 15 had at least two copies in the *P. tepidariorum* genome. A BLAST-based approach revealed a similar pattern of duplication in other spiders and a scorpion, but not among other chelicerates and arthropods, with the exception of a horseshoe crab. Among the duplicated microRNAs we found examples of lineage-specific tandem duplications, and the duplication of entire microRNA clusters in three spiders, a scorpion, and in a horseshoe crab. Furthermore, we found that paralogs of many *P. tepidariorum* microRNA families exhibit arm switching, which suggests that duplication was often followed by sub- or neofunctionalization. Our work shows that understanding the evolution of microRNAs in the chelicerates has great potential to provide insights into the process of microRNA duplication and divergence and the evolution of animal development.

## Introduction

MicroRNAs are a class of small noncoding RNAs that fine-tune gene expression by repressing translation of targeted mRNAs ( Bartel 2004 ; Eichhorn et al. 2014 ). Perturbed microRNA function in metazoans has been shown to disrupt diverse developmental processes such as embryonic survival and viability ( Chen et al. 2014 ), stem cell proliferation ( Hatfield et al. 2005 ), metamorphosis in insects ( Sokol et al. 2008 ; Ambros 2011 ), organ development ( Chen et al. 2014 ), and vertebral number and identity ( Wong et al. 2015 ). Moreover, variation in microRNA function has become increasingly associated with the evolution of cellular differentiation, morphological complexity, and even multicellularity ( Heimberg et al. 2008 ; Peterson et al. 2009 ; Wheeler et al. 2009 ; Campo-Paysaa et al. 2011 ; Chen et al. 2012 ; Guerra-Assunção and Enright 2012 ; Arif et al. 2013 ; Fromm et al. 2013 ; Tarver et al. 2015 ).

The current understanding of canonical microRNA biogenesis in metazoans is that the microRNA primary transcript folds into a hairpin structure that is cleaved by the endonuclease Drosha forming the so-called precursor microRNA hairpin (pre-microRNA). Pre-microRNAs are then exported to the cytoplasm, where they are further cleaved by Dicer to produce a double stranded RNA molecule ( Bartel 2004 ; Chendrimada et al. 2005 ). One of the two RNA strands is subsequently loaded into the RNA-Induced Silencing Complex (RISC) ( Gong et al. 2014 ; Guennewig et al. 2014 ). The mature microRNA guides the RISC to specific mRNAs through complementary binding to regions in their 3′-UTRs. Once bound, the miRISC represses translation by destabilizing the mRNA, repressing binding of translation initiation factors, and can lead to the degradation of transcripts ( Bartel 2004 ; Chendrimada et al. 2005 ; Kawamata et al. 2011 ; Juvvuna et al. 2012 ; Eichhorn et al. 2014 : Fukaya et al. 2014 ).

Normally, one of the two arms of a microRNA hairpin is preferentially incorporated into the RISC. However, some pre-microRNAs can generate two functional products, one from each arm, which normally target different mRNA repertoires ( Marco, Macpherson, et al 2012 ; Gong et al. 2014 ; Guennewig et al. 2014 ). This mode of processing has been found to play a role in cell differentiation ( Zhou et al. 2010 ) and disease progression ( Pink et al. 2015 ). Moreover, for several microRNAs, it has been observed that differences have evolved between insects with respect to which arm of a conserved pre-microRNA is dominantly loaded into the RISC ( Marco et al. 2010 ; Griffiths-Jones et al. 2011 ). This evolutionary change is likely to have altered the target repertoires of these microRNAs between species, which is predicted to lead to different functional consequences because the two mature products from the precursor microRNA have different sequences and are therefore expected bind to distinct recognition sites and different mRNA targets ( Marco, Macpherson, et al. 2012 ).

Comparative studies in animals such as vertebrates ( Heimberg et al. 2010 ; Field et al. 2014 ; Fromm et al. 2015 ), arthropods ( Campbell et al. 2011 ; Rota-Stabelli et al. 2011 ; Wiegmann et al. 2011 ), flatworms ( Fromm et al. 2013 ), and annelids ( Helm et al. 2012 ) have contributed greatly to our knowledge of microRNA function and evolution ( Hatfield et al. 2005 ; Okamura et al. 2008 ; Marco et al. 2010 , 2013 ; Griffiths-Jones et al. 2011 ; Chen et al. 2014 ; Marco, 2014 ; Mohammed, Bortolamiol-Becet, et al. 2014 ; Mohammed, Siepel, et al. 2014 ; Yang et al. 2014 ). However, our current understanding of microRNA evolution in arthropods is generally limited to hexapod lineages.

The Chelicerata subphylum, which diverged from the Mandibulata approximately 500 MYA, and includes morphologically diverse animals such as spiders, scorpions, mites, ticks, and horseshoe crabs, could provide new insights into the evolution of microRNAs in arthropods ( Dunlop 2010 ; Rota-Stabelli et al. 2011 , 2013 ; Sharma, Gupta, et al. 2014 ; Sharma, Kaluziak, et al. 2014 ; Schwager et al. 2015 ) ( supplementary fig. S1 , Supplementary Material online). However, to date, the characterization of microRNAs in chelicerates has been limited primarily to mites and ticks, representing a rather narrow sampling of this subphylum ( Barrero et al. 2011 ; Rota-Stabelli et al. 2011 ; [Bibr evw143-B98][Bibr evw143-B52] ; Luo et al. 2015 ). Therefore, we chose to survey microRNAs in the common house spider *Parasteatoda tepidariorum* (formerly *Achaearanea tepidariorum* ), which has emerged as the main model spider for evolutionary developmental biology ( Hilbrant et al. 2012 ) and thus offers the potential for future functional investigations. Studies in this spider have already made important contributions to understanding the regulation and evolution of axis formation, segmentation, and nervous system development ( McGregor et al. 2008 , Hilbrant et al. 2012 , Schwager et al. 2015 ). Moreover, sequencing of the transcriptome ( Posnien et al. 2014 ) and genome of *P. tepidariorum* (i5k Consortium, unpublished data) now facilitates genomic approaches to understanding gene regulation in this spider in comparison to other chelicerates and other arthropods.

Surveying the evolution of microRNAs in spiders is of a particular interest because, intriguingly, there is evidence of extensive gene duplications in spider genomes ( Schwager et al. 2007 ; Janssen et al. 2010 , 2015 ; Clarke et al. 2015 ; Turetzek et al. 2015 ). Furthermore, duplication of coding genes has also been found in other chelicerate species such as scorpions and horseshoe crabs ( Nossa et al. 2014 ; Sharma, Schwager, et al. 2014 ; Kenny et al. 2016 ; Sharma et al. 2015 ). It is therefore important to estimate copy numbers of microRNA families within *P. tepidariorum* and other chelicerates to determine if the patterns of protein-coding and microRNA gene duplication are similar.

In this study, we have used deep sequencing and informatics approaches to characterize the embryonic microRNA repertoire of *P. tepidariorum* , and used this resource to identify novel microRNAs as well as to determine putative paralogy and orthology relationships of the microRNAs of this spider with those of other chelicerates. We found that conserved microRNA orthologues show a signature of duplication in *P. tepidariorum* . We also found this pattern of duplication in two other spiders and a scorpion but not in the other arachnids (mites and ticks) surveyed. Interestingly, the horseshoe crab *Limulus polyphemus* also contains many duplicated microRNAs, but based on previous studies of the chelicerate phylogeny, these expansions are possibly independent of those we find in arachnids (discussed in Schwager et al. 2016 ). Interestingly, the paralogous *P. tepidariorum* microRNAs exhibit divergence in arm usage suggesting that they may have diversified in target gene repertoire and function, perhaps reflecting specific roles in the regulation of spider development.

## Materials and Methods

### *Parasteatoda t* *epidariorum* Culture and RNA Sequencing

*Parasteatoda tepidariorum* (from a strain collected in Göttingen, Germany) were raised at 25 °C and fed on a diet of *Drosophila* and *Gryllodes sigillatus* . Total RNA was extracted from the embryos of ten healthy cocoons, corresponding to stages 1–10 of embryogenesis in this spider ( Mittmann and Wolff 2012 ), using QIAzol (Qiagen) following the manufacturer’s instructions. Small RNA (∼15–30 nt) libraries were prepared with the Illumina TruSeq® Small RNA Sample Prep Kit according to the manufacturer’s instructions, and library quality was assessed on the 2200 TapeStation instrument. Size selection between 18 and 30 bp was performed by excision of RNA from a 6% DNA PAGE gel, 1 mm (Invitrogen). Sequencing was performed on the Illumina HiSeq 2000 platform at the University of Manchester Genomic Technologies facility, and yielded a total of 177,789,607 reads (100 bp). The raw read data have been deposited in the Sequence Read Archive, with accession PRJEB13119.

### Analysis of Small RNA Sequencing Data and Identification of *P. tepidariorum* microRNAs

The raw read quality was confirmed using FastQC v0.11.2 ( Andrews, 2010 ), after which adapter sequences were trimmed using the Cutadapt v1.4.1 tool ( Martin 2011 ), and reads longer than 17 bp were retained. Reads were then mapped against the *P. tepidariorum* genome (GCA_000365465.1) using Bowtie v1.0.0 ( Langmead et al. 2009 ) with the parameters -n 0 -m 5 -a –best –strata, to best discern among reads from paralogous microRNAs. All mapped reads were analyzed using miRDeep2 v0.0.5 ( Friedländer et al. 2008 ) to predict microRNAs. To preliminarily identify orthologs, the predicted *P. tepidariorum* microRNAs hairpins were then compared against metazoan microRNA precursor sequences from miRBase v21 ( Kozomara and Griffiths-Jones 2014 ) using BLAST v2.2.28+ (BLASTn; -word_size 4; -reward 2; -penalty -3; -evalue 0.01; -perc_identity 70) ( Altschul et al. 1990 ). Predicted microRNAs that did not have significant sequence similarity to any other microRNA in miRBase were then manually inspected. Those that met the following criteria were discarded from the study: fewer than ten reads mapping to the locus; poorly defined Dicer processing sites, defined as less than 50% of reads for a given mature microRNA having the same five end; the predicted mature microRNA sequence had a BLAST hit to more than ten loci in the *P. tepidariorum* genome with two or fewer mismatches. Predicted paralogous microRNAs were aligned using Clustal-Omega ( Sievers et al. 2011 ) and then manually inspected in RALEE v0.8 ( Griffiths-Jones 2005 ).

### Identification of *P. tepidariorum* microRNA Orthologs in Other Species

We searched for orthologs of all *P. tepidariorum* pre-microRNAs in the genomes of two other spiders ( *Acanthoscurria geniculata* —GCA_000661875.1 [ Sanggaard et al. 2014 ], *Stegodyphus mimosarum* —GCA_000611955.2 [ Sanggaard et al. 2014 ]), a scorpion ( *Centruroides sculpturatus* —GCA_000671375.1), four parasitiforms ( *Ixodes scapularis* —GCA_000208615.1 [ Lawson et al. 2009 ], *Metaseiulus occidentalis* —GCA_000255335.1 [ Hoy 2009 ], *Rhipicephalus microplus* —GCA_000181235.2, *Varroa destructor* —GCA_000181155.1 [ Cornman et al. 2010 ]), an acariform ( *Tetranychus urticae* —GCA_000239435.1 [ Grbić et al 2011 ]), the horseshoe crab ( *L. polyphemus* —GCA_000517525.1 [ Nossa et al. 2014 ]), five mandibulates ( *Strigamia maritima* —GCA_000239455.1 [ Chipman et al. 2014 ], *Daphnia pulex* —GCA_000187875.1, *Tribolium castaneum* —GCA_000002335.2 [ Tribolium Genome Sequencing Consortium et al. 2008 ], *Apis mellifera* —GCA_000002195.1, *Drosophila melanogaster* —GCA_000001215.4), and a nematode ( *Caenorhabditis elegans* —GCA_000002985.3s) using a combination of BLAST (BLASTn; -word_size 4; -reward 2; -penalty -3; -evalue 0.1) ( Altschul et al. 1990 ) and INFERNAL v1.1.1 (E-value cut-off of 1) ( Nawrocki et al. 2009 ). For each species, the predicted microRNA repertoire was supplemented with known microRNAs from miRBase v21 ( Kozomara and Griffiths-Jones 2014 ) and for *R. microplus* from Barrero et al. (2011) . For each microRNA, all BLAST and INFERNAL hits were aligned using Clustalw2 ( Larkin et al. 2007 ) in RALEE ( Griffiths-Jones 2005 ). Putative orthologs were confirmed by manual inspection of the multiple sequence alignment using RALEE v0.8 ( Griffiths-Jones 2005 ), looking for high similarity of one or both mature arms of the hairpin precursor. To determine the structure and minimum free energy of all chelicerate microRNAs detected by BLAST and INFERNAL searches, precursor microRNA sequences were analyzed with RNAfold v1.8.4 using default settings ( Gruber et al. 2008 ). All predictions retained had greater than −0.2 kcal/mol/nt ( Kozomara and Griffiths-Jones 2014 ).

### Comparisons of Relative Arm Usage

To compare the relative arm usage of *P. tepidariorum* microRNAs to those of other species we used a similar approach to Marco et al. (2010) . The average number of reads per experiment catalogued in miRBase for each microRNA arm for *D. melanogaster* and *T. castaneum* were obtained from miRBase v21 ( Kozomara and Griffiths-Jones 2014 ). For the multicopy microRNA families in *D. melanogaster* and *T. castaneum* the average arm usage across the family was used. Arm usage of *P. tepidariorum* microRNAs with unique 5’ and 3’ mature sequences was also quantified. *P. tepidariorum* microRNAs with one unique mature sequence were also analyzed, with the caveat that the expression of the other arm was possibly over or under estimated. Note that arm usage of *P. tepidariorum* microRNAs that have nonunique sequences for both mature products could not be reliably assessed because reads could not be unambiguously mapped to one location.

## Results

### Sequencing of Small RNAs and Prediction of microRNAs in *P. tepidariorum*

Small RNAs were sequenced to determine the repertoire of microRNAs expressed during *P. tepidariorum* embryogenesis. This yielded a total of 177,789,607 raw reads of which 159,258,789 (95.5%) processed reads were mapped to the genome of *P. tepidariorum* . The distribution of these mapped read sizes contain two peaks at 22 and 29 bp, indicating the presence of mature microRNAs and a presumptive pool of piRNAs, respectively ( fig. 1 *A* ) ( Saito et al. 2006 ). From these mapped reads miRDeep2 analysis initially predicted 278 microRNAs, which constituted 14,942,552 (9.4%) of the total mapped reads. Of these predicted microRNAs, 130 had either less than 50% of reads that had the same 5’ end, low read numbers, or were found repeatedly in the *P. tepidariorum* genome (see Materials and Methods). While these discarded candidates could potentially be functional microRNAs, they were removed to give a conservative final prediction of 148 microRNAs ( supplementary files S1 and S2 , Supplementary Material online) that are expressed during embryogenesis in *P. tepidariorum* .

**Fig . 1.—:**
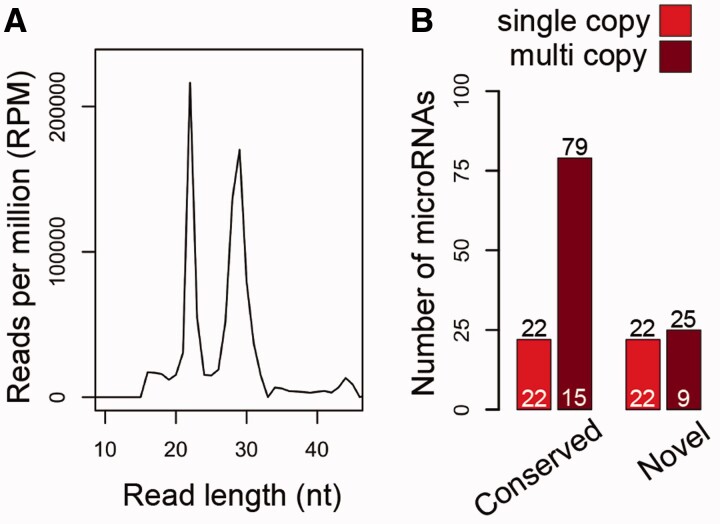
*Parasteatoda tepidariorum* contains many highly expressed multicopy microRNA families. ( *A* ) Distribution of the read lengths of mapped reads with peaks at 22 and 29 nt. ( *B* ) Numbers of microRNAs (black numbers) and microRNA families (white numbers).

We then used BLAST and metazoan pre-microRNAs from miRBase v21 ( Kozomara and Griffiths-Jones 2014 ) to annotate orthologs of the 148 predicated microRNAs. This approach identified 101 conserved microRNAs, with similarity to at least one previously annotated metazoan microRNA in miRBase. The remaining 47 were therefore classified as “novel” *P. tepidariorum* microRNAs ( fig. 1 *B* ) despite some having putative orthologs in other chelicerate species ( fig. 2 ). Note that we cannot rule out the possibility that our novel microRNA set includes some genes conserved in other species but diverged in sequence beyond our ability to recognize them as homologs, even using state-of-the-art structure-aware RNA homology search tools.

**Fig . 2.—:**
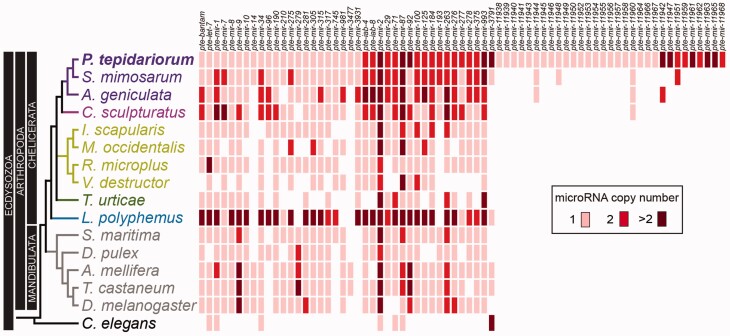
MicroRNA family copy number in *P. tepidariorum* and other ecdysozoans. Presence of *P. tepidariorum* single-copy and multicopy microRNA families in other spiders (purple), scorpions (magenta), horseshoe crab (blue) parasitiformes (green), acariformes (dark green), mandibulates (gray), and a nematode (black). The spiders, the scorpion, and the horseshoe crab all display large numbers of multi-copy microRNA families relative to other species.

We also grouped microRNAs based on whether they were found as a single copy or if the microRNA family had been duplicated and therefore contained multiple microRNAs (multicopy microRNAs). Of the conserved microRNAs, there were 22 single-copy microRNAs, and 79 multicopy microRNAs belonging to 15 families ( fig. 1 *B* ). For the novel microRNAs, there were 22 single-copy microRNAs, and 25 multicopy microRNAs belonging to nine families ( fig. 1 *B* ). Our prediction and annotation process therefore suggests that at least 36% of *P. tepidariorum* microRNA families have been duplicated at least once.

### Identification of Conserved *P. tepidariorum* microRNA Families in Other Chelicerates

We next searched for all of the 148 predicted *P. tepidariorum* microRNAs in the genomes of other chelicerates ( supplementary file S3 , Supplementary Material online) and also compared the results to the repertoire of microRNAs present in other selected ecdysozoans ( fig. 2 ). We identified 32 conserved microRNA families from *P. tepidariorum* in two other spiders and a scorpion. The conserved families of mir-14, mir-3477, and mir-3791 were present in *S. mimosarum* , but absent in *A. genticulata* and *C. sculpturatus* ( fig. 2 ). The mir-3791 family appears to be absent from the genomes of all the other chelicerates, except *S. mimosarum* and *L. polyphemus* , but is present in some insects ( *T. castaneum* and *A. mellifera* ) that we surveyed, and also shares sequence similarity with the *mir-35-41* cluster in *Ca. elegans* ( Massirer et al. 2012 ; Fromm et al. 2013 ; McJunkin and Ambros 2014 ).

The other arachnids showed variable patterns of retention and loss of the 35 conserved microRNA families found in *P. tepidariorum* . There were nine microRNA families that were present in all of these animals. The lowest number of families in any chelicerate surveyed was 17 in *V. destructor* ( fig. 2 ). Apart from the spiders and scorpion, mir-193 is likely to have been lost from the genomes of other chelicerates including *L. polyphemus* ( fig. 2 ). Note that this microRNA is commonly lost in metazoan lineages ( Tarver et al. 2013 ). Other potential losses in chelicerates include mir-14 and mir-3477, which was only present in *P. tepidariorum* and *S. mimosarum* . *Limulus polyphemus* has also lost mir-981 and mir-279, but has retained the other 31 conserved *P. tepidariorum* microRNA families ( fig. 2 ).

Comparing the conserved microRNA families found in *P. tepidariorum* to other nonchelicerate arthropods reveals that 23 out of 35 are found in the genomes of all the insects, the myriapod and the crustacean in our survey ( fig. 2 ). Interestingly, mir-96 may have been lost in insects but retained in the crustacean, *Da. pulex* , as well as most chelicerates, while mir-3931 appears to have evolved in chelicerates as previously reported ( Tarver et al. 2013 ; Wheeler et al. 2009 ), although it may also have been lost in the common ancestor of the other arthropod subphyla ( fig. 2 ).

### Novel *P. tepidariorum* microRNAs Are Largely Species-Specific

Of the 22 novel single-copy microRNA families we identified in *P. tepidariorum* , two were identified in the genome of *A. geniculata* and three in *S. mimosarum* , whereas only one (mir-11960) was found in *C. sculpturatus* , and none was found in any other chelicerate ( fig. 2 ). As described above, we also identified nine novel multicopy microRNA families in *P. tepidariorum* . However of these nine families, we were only able to identify mir-11951 in *S. mimosarum* and mir-11942 in *A. geniculata.* Though interestingly, both of these microRNA families contained duplicates ( fig. 2 ). Taken together our analysis suggests that most of the novel microRNAs identified in *P. tepidariorum* are likely to be young and to have recently evolved in the lineage leading to this spider.

### Incidence of Multicopy *P. tepidariorum* microRNA Families in Other Chelicerates and Ecdysozoans

As mentioned above, we found that 15 out of the 35 conserved microRNA families in *P. tepidariorum* were represented by two or more paralogs. There were also many multicopy families in the other spiders ( *S. mimosarum* and *A. geniculata* ) and the scorpion ( *C. sculpturatus* ) with 16, 17, and 16 out of 35 conserved families represented by two or more paralogs, respectively ( fig. 2 ). There was considerable overlap of multicopy families between these species ( *S. mimosarum* , *A. geniculate* , and *C. sculpturatus* ) and *P. tepidariorum* with 12, 11, and 10 families represented by at least two paralogs in the respective species and *P. tepidariorum* ( fig. 2 ). Moreover, eight families are multicopy among all four of these species ( fig. 2 ). In contrast, only up to five (in the parasitiforms *M. occidentalis* and *I. scapularis* ) multicopy microRNA families were observed in any other chelicerate, or ecdysozoan, we surveyed with the notable exception of *L. polyphemus* ( fig. 2 ). In the horseshoe crab, 26 out of the 30 microRNAs found (with respect to the conserved *P. tepidariorum* microRNAs) were present as multiple copies, with 21 present as three or more copies. These included mir-8 and mir-10, which were not duplicated in any of the other ecdysozoans that we surveyed ( fig. 2 ).

In the manidibulates, parasitiformes and acariformes analyzed, approximately 75–90% of microRNA are represented by a single microRNA ( supplementary fig. S2 , Supplementary Material online). In contrast, only approximately 40–55% of microRNAs were present as single copy in the spiders and scorpion, whereas approximately 35–40% contain two copies in these species ( supplementary fig. S2 , Supplementary Material online). In the horseshoe crab microRNAs were represented by one up to 17 copies; however most contained five copies ( supplementary fig. S2 , Supplementary Material online).

### Small- and Large-Scale Duplications of microRNAs in Chelicerates

These observations strongly suggest that both small-scale tandem duplications as well as larger scale duplications contributed to the pattern of duplicated microRNAs in spiders. In *P. tepidariorum* , we found that the mir-3791 family is represented by 34 copies, but in contrast only single copies of this family were identified in *S. mimosarum* and *L. polyphemus* ( fig. 2 ). Interestingly, 13 of the *P. tepidariorum mir-3791* paralogs are located within a 50 kb locus on a single 671 kb scaffold ( supplementary fig. S3 , Supplementary Material online). Therefore it is clear that tandem duplications have at least in part contributed to the expansion of this microRNA family in the lineage leading to this spider.

We also found evidence that larger scale duplications have contributed to the expansion of microRNA repertoires in *P. tepidariorum* and other chelicerates. The *mir-71* / *mir-2* cluster is an invertebrate-specific microRNA cluster that has expanded in arthropods probably due to tandem duplications of *mir-2* ( Marco et al. 2010 ; Marco, Hooks, et al. 2012 ). In the acariform and parasitiform lineages, the copy numbers of *mir-71* and *mir-2* are variable, though usually there is one copy of *mir-71* and two copies of *mir-2* ( fig. 2 ). In the spiders, the scorpion and the horseshoe crab, we consistently found at least two copies of *mir-71* and more than two copies of *mir-2* ( fig. 2 ). In the spiders and the scorpion, these microRNAs were generally found as two separate clusters on different scaffolds ( fig. 3 ). *Parasteatoda tepidariorum* contains one cluster containing a single copy of *mir-71* and three copies of *mir-2* , and a second cluster, on a different scaffold, also with one copy of *mir-71* , but with six copies of *mir-2* ( fig. 3 ).

**Fig . 3.—:**
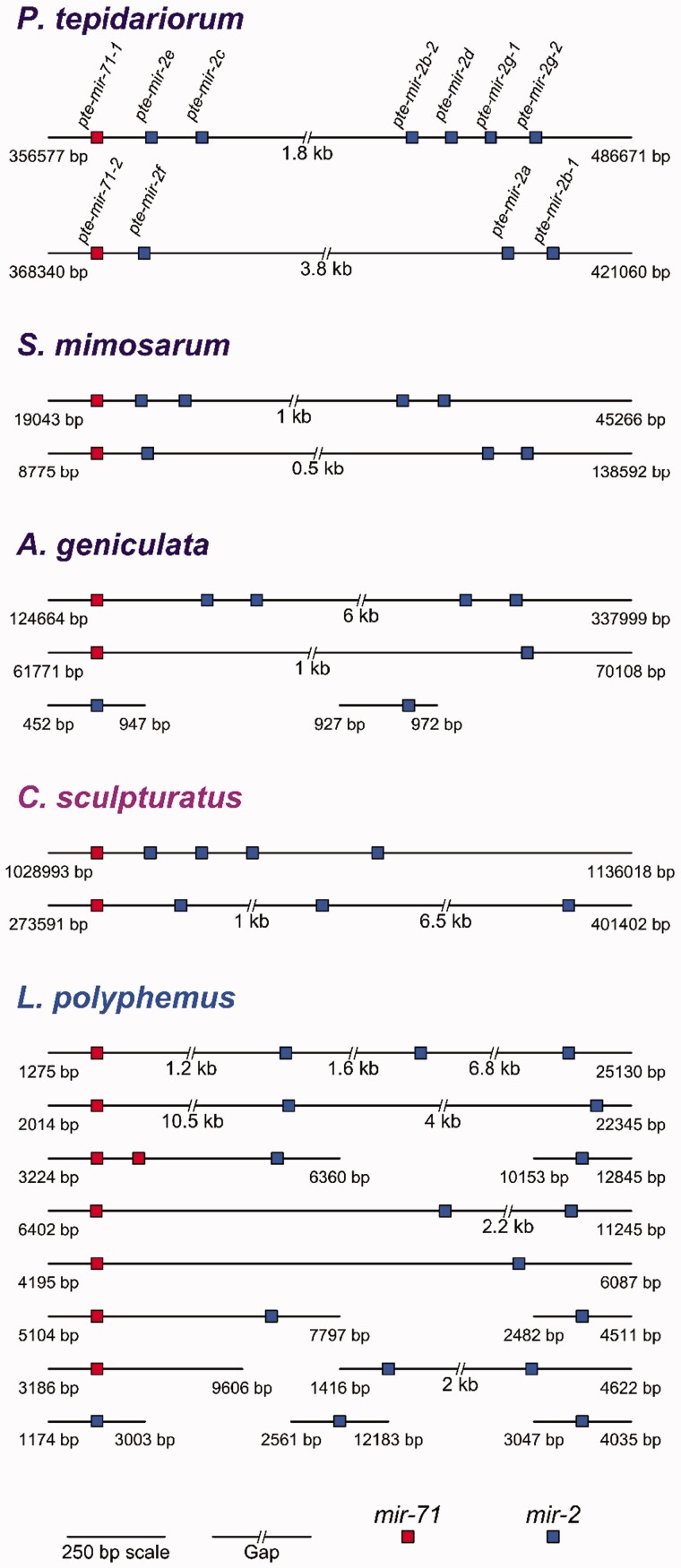
The duplicate *mir-71* / *mir-2* clusters in chelicerate lineages. The *mir-71* / *mir-2* cluster is duplicated in spiders (purple), the scorpion (magenta), and the horseshoe crab (blue). Each species displays lineage-specific retention, loss and further small segmental duplications. The position of the left most microRNA and the total scaffold length are indicated on the left and right hand side, respectively.

In the *S. mimosarum* and *C. sculpturatus* genomes we found one scaffold containing one copy of *mir-71* and four copies of *mir-2* ( fig. 3 ), and a second scaffold with one copy of *mir-71* and three copies of *mir-2* ( fig. 3 ). In *A. geniculata* there is one complete cluster with one *mir-71* and four *mir-2* copies, all on one scaffold ( fig. 3 ). There is another scaffold with one copy of *mir-71* and one copy of *mir-2* located approximately 6 kb from the end of the scaffold. There are also two other copies of *mir-2* , each on approximately 1 kb scaffolds. The arrangement of *L. polyphemus mir-71* / *mir-2* genes is much more fragmented ( fig. 3 ). In this horseshoe crab we found eight copies of *mir-71* located on seven scaffolds, of which six also contain at least one copy of *mir-2* ( fig. 3 ). The seven copies of *mir-2* that are located on six different scaffolds are all found close to the ends of the scaffolds, and may therefore possibly be fragments of larger *mir-71* / *mir-2* clusters ( fig. 3 ).

A second microRNA cluster that we observed to be duplicated in some species is the *mir-100* / *let-7* / *mir-125* cluster ( supplementary fig. S4 , Supplementary Material online). However, in all lineages there appeared to be possible loss or rearrangement of at least one cluster, although we cannot exclude the possibility that this is an artifact of the genome assembly.

In *P. tepidariorum* we found one complete cluster ( supplementary fig. S4 , Supplementary Material online), and additional copies of *mir-100* and *mir-125* on separate scaffolds but no paralog of *let-7* ( supplementary fig. S4 , Supplementary Material online). The arrangement of these microRNAs in *S. mimosarum* is similar to *P. tepidariorum* ( supplementary fig. S4 , Supplementary Material online) although the two *mir-100* paralogs are on separate scaffolds ( supplementary fig. S4 , Supplementary Material online). *Acanthoscurria geniculata* has a complete *mir-100* / *let-7* / *mir-125* cluster and two additional copies of *mir-125* are found on separate scaffolds ( supplementary fig. S4 , Supplementary Material online). In *C. sculpturatus* , we found a single cluster, which appears to have been rearranged, similar to one of the three *L. polyphemus* clusters ( supplementary fig. S4 , Supplementary Material online).

Therefore the *mir-71* / *mir-2* and *mir-100* / *let-7* / *mir-125* clusters appear to have been duplicated in spiders and scorpions (Arachnopulmonata) and *L. polyphemus* but not other chelicerates or mandibulates that we surveyed. Taken together, our results show that both tandem duplications and larger scale duplications of entire clusters, followed by subsequent lineage-specific expansion or loss of microRNAs, have contributed to the evolution of chelicerate microRNA repertoires.

### Divergence in microRNA Arm Usage in Multicopy microRNAs

Duplicate microRNAs have the potential to diversify their functions by a number of evolutionary mechanisms, including arm usage ( Okamura et al. 2008 ; de Wit et al. 2009 ; Marco et al. 2010 ; Griffiths-Jones et al. 2011 ). Inspection of microRNA arm usage in *P. tepidariorum* suggests that there is a bias toward the mature sequence deriving from the 3’ arm of the hairpin ( supplementary fig. S5 , Supplementary Material online). The distribution was statistically different from that in both *T. castaneum* ( *D* = 0.2124; *P* = 0.002) and *D. melanogaster* ( *D* = 0.2955; *P* = 5.4 × 10 ^−^^7^ ), but was not different between *T. castaneum* and *D. melanogaster* ( *D* = 0.1343; *P* = 0.08) (Kolmogorov–Smirnov tests).

We then compared relative arm usage for individual microRNAs between *P. tepidariorum* , *T. castaneum* , and *D. melanogaster* and observed multiple cases of arm switching among these species ( fig. 4 ). Considering first the microRNAs that have unique mature sequences in *P. tepidariorum* , only *mir-275* and *mir-3477* exhibit differences in relative arm usage between *P. tepidariorum* and *T. castaneum* , ( fig. 4 *A* ). Comparisons between *P. tepidariorum* and *D. melanogaster* shows that *mir-10* , *mir-993a* , *mir-278b* , *mir-281* , also display changes in arm use ( fig. 4 *B* ).

**Fig . 4.—:**
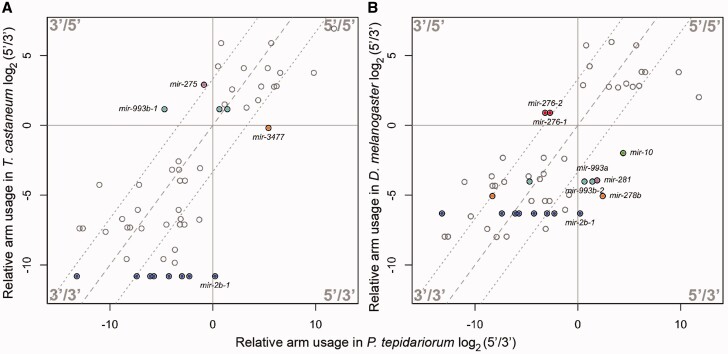
Relative arm usage changes in microRNAs. Comparisons of the relative strand usage of *P. tepidariorum* microRNAs to ( *A* ) *T. castaneum* and ( *B* ) *D. melanogaster* show microRNAs that have undergone strand switching in the 3′/5′ and 5′/3′ quadrants. The dashed line indicates the theoretical expectation for conserved arm usage between the two species. Dotted lines limit the boundaries of the dashed line to less than 10-fold differences in arm usage.

There were also occurrences of arm switching for microRNAs that contained only one unique mature sequence in *P. tepidariorum* . Between *P. tepidariorum* and *T. castaneum* , *mir-993b-1* , *mir-2b-1* all exhibit differences in relative arm usage ( fig. 4 *A* ). Comparing *P. tepidariorum* with *D. melanogaster* , showed that *mir-993b-2* , both copies of *mir-276* , and again *mir-2b-1* exhibited arm switching ( fig. 4 *B* ).

We also identified that one *P. tepidariorum mir-3791* paralog had been subject to relative arm switching, and another paralog that had similar 5’ and 3’ expression, which is in contrast with the rest of this mostly 3’ dominant family in *P. tepidariorum* ( supplementary fig. S6 , Supplementary Material online). Both of these microRNAs have unique mature sequences in *P. tepidariorum* ( supplementary file S1 , Supplementary Material online), though some of the other paralogs of this family do not have unique sequences for both mature products ( supplementary file S1 , Supplementary Material online).

It was also possible to compare the arm usage among the paralogs of the nine novel multicopy microRNA families identified in *P. tepidariorum* ( fig. 2 ). The mir-11942 family exhibited differential arm usage between paralogs ( supplementary fig. S7 , Supplementary Material online). Another family, mir-11961, also showed variation in arm usage between paralogs, however there was less striking differences between paralogs compared to those observed in the mir-11942 family ( supplementary fig. S7 , Supplementary Material online). Importantly, the paralogs of these two microRNA families all had unique mature sequences in *P. tepidariorum* ( supplementary file S1 , Supplementary Material online). The other novel multicopy families showed relatively similar arm usage between paralogs. However, these belonged to microRNA families with one nonunique mature sequence in *P. tepidariorum* in relation to their paralogs ( supplementary file S1 , Supplementary Material online).

## Discussion

In this study, we surveyed the repertoire of microRNAs expressed during embryogenesis in *P. tepidariorum* and compared them with other chelicerates to examine patterns of duplication during the evolution of these genes in arthropods and other animals. From the initial 278 miRDeep2 predictions, we focused on a conservative total of 148 microRNAs representing 66 families expressed during *P. tepidariorum* embryogenesis ( fig. 1 ). This number is similar to the complement of 172 microRNAs expressed during *D. melanogaster* embryogenesis ( Ninova et al. 2014 ). However, *D. melanogaster* has a total of 256 microRNAs identified across all life stages ( Kozomara and Griffiths-Jones 2014 ). Therefore, it is likely that further microRNAs expressed later in development and in adults remain to be identified in *P. tepidariorum* . Despite this, we have characterized more microRNAs in this spider than previously identified in other arachnids ( *R. microplus* [87], *I. scapularis* [49], and *T. urticae* [52]) ( Wheeler et al. 2009 ; Barrero et al. 2011 ; Grbić et al. 2011 ) except the tick *Hyalomma anatolicum anatolicum* ( Luo et al. 2015 ). Thus *P. tepidariorum* possesses one of the largest chelicerate microRNA repertoires sequenced to date, and is the only one where small RNAs have been mapped against the specific corresponding genome sequence.

Approximately half of the microRNA families identified in *P. tepidariorum* by small RNA seq were conserved in other metazoans ( fig. 2 ). These included 21 out of 31 families that are common to most bilateral animals, and 7 out of the 12 families present in protostomes ( Tarver et al. 2013 ). The losses include mir-33, mir-219, mir-2001, and mir-1993, which have been commonly lost in metazoans ( Tarver et al. 2013 ). *P. tepidariorum* also contains panarthropod (mir-276 and mir-305), arthropod (iab-4/8 and mir-275), and chelicerate-specific (mir-3931) microRNAs ( Rota-Stabelli et al. 2011 ; Tarver et al. 2013 ). We did not find mir-242 and mir-216, which are also lost in other arthropods, nor mir-31, which is not found in the chelicerate family of Ixodidae, or the mandibulate-specific mir-282 or mir-965 ( Rota-Stabelli et al. 2011 ; Tarver et al. 2013 ). It is possible that due to our sampling of embryonic small RNA we may have missed microRNA families ( Tarver et al. 2013 ) that are only expressed during other life stages. Indeed, we were able to identify one copy of *mir-133* , two copies of *mir-137* , and three copies of *mir-124* ( supplementary file S4 , Supplementary Material online) in the genome of *P. tepidariorum* , further increasing the number of microRNAs that we identified.

### Evidence of Duplication of Both Coding and Noncoding Genes in *P. tepidariorum*

Over one-third of the conserved microRNA families and many of the novel microRNA families are present in multiple copies in *P. tepidariorum* ( fig. 1 *B* ). This expansion appears to have resulted from both local tandem duplications and larger scale segmental duplications ( fig. 3 , supplementary figs. S3 and S4 , Supplementary Material online). Previous reports have found that some protein-coding genes in *P. tepidariorum* have also been duplicated ( Posnien et al. 2014 ). Of the 8,917 *P. tepidariorum* transcripts identified as being orthologous to a *Drosophila* gene, approximately 28% are likely to be expressed from duplicated genes ( Posnien et al. 2014 ). This therefore suggests that there has possibly been greater retention of duplicated microRNAs compared to protein-coding genes in *P. tepidariorum.* This difference is similar to previous estimates of microRNAs and coding gene retention following large-scale duplication ( Berthelot et al. 2014 ).

### Duplication and Divergence of microRNAs in Multiple Chelicerate Lineages

Evidence of gene duplication in chelicerates is not limited to *P. tepidariorum* . Analysis of the transcriptomes of representatives of the Mygalomorphae and Araneomorphae suggests that these spiders are likely to have shared a large-scale duplication event and that retained paralogs may have contributed to the origin and evolution of silk glands ( Clarke et al. 2015 ). Hox genes in the spider *Cupiennius salei* and scorpions, including *C. sculpturatus* and *Mesobuthus martensii* , have also been found to be duplicated, though it is unclear whether these are shared or independent duplication events ( Schwager et al. 2007 ; Sharma, Schwager, et al. 2014 ; Di et al. 2015 , Sharma et al. 2015 ). In the horseshoe crab, there is also evidence of two rounds of whole-genome duplication, which are may be independent of the duplication events that have occurred in spiders or scorpions ( Nossa et al. 2014 ; Sharma, Gupta, et al. 2014 ; Sharma, Kaluziak, et al. 2014 ; Kenny et al. 2016 ; Schwager et al. 2015 ). The presence of mir-193 in Arachnopulmonata and its absence in all the other chelicerates we surveyed further supports the hypothesis of independent duplication events.

We also identified many duplicate microRNA families in spiders, scorpions, and a horseshoe crab ( fig. 2 and supplementary fig. S2 , Supplementary Material online). We found that *L. polyphemus* has the largest estimated microRNA repertoire among chelicerates. Interestingly, many microRNA families were found to be represented by multiple genes in all three spiders and the scorpion that we surveyed ( fig. 2 ). These findings are consistent with a possible large-scale duplication event in the common ancestor of spiders and scorpions (Arachnopulmonata), and the two possibly independent rounds of whole-genome duplication in horseshoe crabs ( Nossa et al. 2014 ; Sharma, Gupta, et al. 2014 ; Sharma, Kaluziak, et al. 2014 ; Kenny et al. 2016 ; Schwager et al. 2015 ).

In contrast to the shared duplication of microRNAs among Arachnopulmonata species, there were differences in the retention of paralogs in some families ( fig. 2 ). These patterns of gain and loss may be due to genome assembly quality or a lack of small RNA sequencing from adult stages of *P. tepidariorum* . Alternatively, differential patterns of retention of microRNA paralogs potentially relates to differential retention of duplicate coding genes between these spider species ( Clarke et al. 2015 ). It is also possible that the paralogous microRNAs found in some species could have been produced by lineages-specific duplication events. However, further analysis of the genomes and small RNA sequencing of all of these chelicerates is required to investigate these different evolutionary scenarios.

### Evolution of microRNA Function

Despite the shared retention of many duplicated genes generated by putative tandem and large-scale duplication events, it is clear from our results that there are also common lineage-specific losses ( figs. 2 and 3 , supplementary figs. S3 and S4 , Supplementary Material online). Changes in microRNA biogenesis may have also contributed to the evolution of their function among chelicerates. We identified that *P. tepidariorum* , like some other ecdysozoans ( de Wit et al. 2009 ), has a general 3’ arm bias with respect to preferential strand loading into the RISC. This bias is greater than that reported for 3’ arm usage compared to *T. castaneum* and *D. melanogaster* ( Marco et al. 2010 ) ( supplementary fig. S5 , Supplementary Material online). This could perhaps be caused by the many paralogs of the mir-3791 and mir-2 families, which were generally 3’ dominant, though both of these families do show instances of arm switching ( fig. 4 and supplementary fig. S6 , Supplementary Material online). However, the microRNA that switched in the mir-2 family did not contain completely unique mature sequences and relative arm switching may have been over or underestimated. Many of the microRNAs that show arm switching, relative to *T. castaneum* and *D. melanogaster* , also belong to multicopy microRNA families. These results suggest that duplication may facilitate functional change between paralogs and provides further evidence that microRNA duplication facilitates changes that can alter strand selection ( de Wit et al. 2009 ).

There were also cases of single-copy microRNAs that have been subject to arm switching between species. miR-10-5p is more abundant than miR-10-3p in both *P. tepidariorum* and *T. castaneum* , while miR-10-3p dominates in *D. melanogaster* ( Griffiths-Jones et al. 2011 ). In both *P. tepidariorum* and *Drosophila* , mature products from both arms are expressed at detectable levels ( Stark et al. 2007 ). The expression of both mature arms may be a feature that contributes to a microRNAs ability to switch strand usage between species.

The pervasive duplication and subsequent divergence in retention and copy number, as well as arm switching, that we have identified among chelicerate microRNAs may have led to their subfunctionalization and neofunctionalization ( Ohno 1970 ; Taylor and Raes 2004 ; Li et al. 2008 ; Amoutzias and Van de Peer 2010 ; Innan and Kondrashov 2010 ; Abrouk et al. 2012 ; Huminiecki and Conant 2012 ; Wang and Adams 2015 ). These evolutionary differences, therefore, may have contributed to the divergence in the developmental programs of chelicerates.

## Conclusions

Our characterization of microRNAs expressed during *P. tepidariorum* embryogenesis and the identification of their orthologs in other arthropods show that there has been pervasive duplication and subsequent divergence in the sequences of these paralogous genes in spiders, scorpions, and the horseshoe crab. It is now essential to apply the tools for analysis of gene expression and function available in *P. tepidariorum* to test the developmental implications of these changes to provide a perspective on the evolution of microRNAs in chelicerates, arthropods, and metazoans.

## Supplementary Material

Supplementary files S1–S4 and figures S1–S7 are available at *Genome Biology and Evolution* online http://www.gbe.oxfordjournals.org/ .

## Supplementary Material

Supplementary Data

## References

[evw143-B1] Abrouk M , . 2012 . Grass microRNA gene paleohistory unveils new insights into gene dosage balance in subgenome partitioning after whole-genome duplication . Plant Cell . 24 ( 5 ): 1776 – 1792 . 22589464 10.1105/tpc.112.095752 PMC3442569

[evw143-B2] Altschul SF Gish W Miller W Myers EW Lipman DJ. 1990 . Basic local alignment search tool . J Mol Biol. 215 : 403 – 410 . 2231712 10.1016/S0022-2836(05)80360-2

[evw143-B3] Ambros V. 2011 . MicroRNAs and developmental timing . Curr Opin Genet Dev. 21 ( 4 ): 511 – 517 . 21530229 10.1016/j.gde.2011.04.003 PMC3149784

[evw143-B4] Amoutzias G Van de Peer Y. 2010 . Single-gene and whole-genome duplications and the evolution of protein-protein interaction networks . In: Caetano-Anolles G , editors. Evolutionary genomics and systems biology . UK : Wiley-Blackwell . p. 413 – 429 .

[evw143-B99] Andrews S. 2010 . FASTQC: A Quality Control Tool for High Throughput Sequence Data. [cited 2015 Jan 10]. Available from: http://www.bioinformatics.babraham.ac.uk/projects/fastqc/ .

[evw143-B5] Arif S , . 2013 . Evolution of mir-92a underlies natural morphological variation in *Drosophila melanogaster.* Curr Biol. 23 ( 6 ): 523 – 528 . 23453955 10.1016/j.cub.2013.02.018 PMC3605577

[evw143-B7] Barrero RA , . 2011 . Evolutionary conserved miRNAs are ubiquitously expressed compared to tick-specific miRNAs in the cattle tick *Rhipicephalus* ( *Boophilus* ) *microplus.* BMC Genomics 12 : 328. 21699734 10.1186/1471-2164-12-328 PMC3141673

[evw143-B8] Bartel DP. 2004 . MicroRNAs: genomics, biogenesis, mechanism, and function . Cell 116 ( 2 ): 281 – 297 . 14744438 10.1016/s0092-8674(04)00045-5

[evw143-B9] Berthelot C , . 2014 . The rainbow trout genome provides novel insights into evolution after whole-genome duplication in vertebrates . Nat Commun. 5 : 3657. 24755649 10.1038/ncomms4657 PMC4071752

[evw143-B10] Campbell LI , . 2011 . MicroRNAs and phylogenomics resolve the relationships of Tardigrada and suggest that velvet worms are the sister group of Arthropoda . Proc Natl Acad Sci U S A. 108 ( 38 ): 15920 – 15924 . 21896763 10.1073/pnas.1105499108 PMC3179045

[evw143-B11] Campo-Paysaa F Sémon M Cameron RA Peterson KJ Schubert M. 2011 . microRNA complements in deuterostomes: origin and evolution of microRNAs . Evol Dev. 13 ( 1 ): 15 – 27 . 21210939 10.1111/j.1525-142X.2010.00452.x

[evw143-B12] Chen CY Chen ST Juan HF Huang HC. 2012 . Lengthening of 3'UTR increases with morphological complexity in animal evolution . Bioinformatics 28 ( 24 ): 3178 – 3781 . 23080117 10.1093/bioinformatics/bts623

[evw143-B13] Chen YW , . 2014 . Systematic study of *Drosophila* microRNA functions using a collection of targeted knockout mutations . Dev Cell . 31 ( 6 ): 784 – 800 . 25535920 10.1016/j.devcel.2014.11.029

[evw143-B14] Chendrimada TP , . 2005 . TRBP recruits the Dicer complex to Ago2 for microRNA processing and gene silencing . Nature 436 ( 7051 ): 740 – 744 . 15973356 10.1038/nature03868 PMC2944926

[evw143-B15] Chipman AD , . 2014 . The first myriapod genome sequence reveals conservative arthropod gene content and genome organisation in the centipede *Strigamia maritima.* PLoS Biol. 12 ( 11 ): e1002005. 25423365 10.1371/journal.pbio.1002005 PMC4244043

[evw143-B16] Clarke TH Garb JE Hayashi CY Arensburger P Ayoub NA. 2015 . Spider transcriptomes identify ancient large-scale gene duplication event potentially important in silk gland evolution . Genome Biol Evol. 7 ( 7 ): 1856 – 1870 . 26058392 10.1093/gbe/evv110 PMC4524477

[evw143-B17] Cornman SR , . 2010 . Genomic survey of the ectoparasitic mite *Varroa destructor* , a major pest of the honey bee *Apis mellifera.* BMC Genomics 11 : 602. 20973996 10.1186/1471-2164-11-602 PMC3091747

[evw143-B18] de Wit E Linsen SE Cuppen E Berezikov E. 2009 . Repertoire and evolution of miRNA genes in four divergent nematode species . Genome Res. 19 ( 11 ): 2064 – 2074 . 19755563 10.1101/gr.093781.109 PMC2775598

[evw143-B19] Di Z , . 2015 . Genome-wide analysis of homeobox genes from *Mesobuthus martensii* reveals Hox gene duplication in scorpions . Insect Biochem Mol Biol. 61 : 25 – 33 . 25910680 10.1016/j.ibmb.2015.04.002

[evw143-B20] Dunlop JA. 2010 . Geological history and phylogeny of Chelicerata . Arthropod Struct Dev. 39 : 124 – 142 . 20093195 10.1016/j.asd.2010.01.003

[evw143-B21] Eichhorn SW , . 2014 . mRNA destabilization is the dominant effect of mammalian microRNAs by the time substantial repression ensues . Mol Cell 56 ( 1 ): 104 – 115 . 25263593 10.1016/j.molcel.2014.08.028 PMC4292926

[evw143-B22] Field DJ , . 2014 . Toward consilience in reptile phylogeny: miRNAs support an archosaur, not lepidosaur, affinity for turtles . Evol Dev. 16 ( 4 ): 189 – 196 . 24798503 10.1111/ede.12081 PMC4215941

[evw143-B23] Friedländer MR , . 2008 . Discovering miRNAs from deep sequencing data using miRDeep . Nat Biotechnol . 26 : 407 – 415 . 18392026 10.1038/nbt1394

[evw143-B24] Fromm B , . 2015 . A uniform system for the annotation of vertebrate microRNA genes and the evolution of the human microRNAome . Annu Rev Genet. 49 : 213 – 242 . 26473382 10.1146/annurev-genet-120213-092023 PMC4743252

[evw143-B25] Fromm B Worren MM Hahn C Hovig E Bachmann L. 2013 . Substantial loss of conserved and gain of novel microRNA families in flatworms . Mol Biol Evol. 30 ( 12 ): 2619 – 2628 . 24025793 10.1093/molbev/mst155 PMC3840308

[evw143-B26] Fukaya T Iwakawa HO Tomari Y. 2014 . MicroRNAs block assembly of eIF4F translation initiation complex in *Drosophila.* Mol Cell 56 ( 1 ): 67 – 78 . 25280104 10.1016/j.molcel.2014.09.004

[evw143-B27] Gong J , . 2014 . Comprehensive analysis of human small RNA sequencing data provides insights into expression profiles and miRNA editing . RNA Biol. 11 ( 11 ): 1375 – 1385 . 25692236 10.1080/15476286.2014.996465 PMC4615373

[evw143-B28] Grbić M , . 2011 . The genome of *Tetranychus urticae* reveals herbivorous pest adaptations . Nature 479 ( 7374 ): 487 – 492 . 22113690 10.1038/nature10640 PMC4856440

[evw143-B29] Griffiths-Jones S. 2005 . RALEE–RNA alignment editor in emacs . Bioinformatics 21 : 257 – 259 . 15377506 10.1093/bioinformatics/bth489

[evw143-B30] Griffiths-Jones S Hui JH Marco A Ronshaugen M. 2011 . microRNA evolution by arm switching . EMBO Rep . 12 ( 2 ): 172 – 177 . 21212805 10.1038/embor.2010.191 PMC3049427

[evw143-B31] Gruber AR Lorenz R Bernhart SH Neuböck R Hofacker IL. 2008 . The Vienna RNA websuite . Nucleic Acids Res. 36 ( Web Server issue ): W70 – W74 . 18424795 10.1093/nar/gkn188 PMC2447809

[evw143-B32] Guennewig B , . 2014 . Synthetic pre-miRNAs reveal dual-strand activity of miR-34a on TNF-α . rna 20 ( 1 ): 61 – 75 . 24249224 10.1261/rna.038968.113 PMC3866645

[evw143-B33] Guerra-Assunção JA Enright AJ. 2012 . Large-scale analysis of microRNA evolution . BMC Genomics 13 : 218. 22672736 10.1186/1471-2164-13-218 PMC3497579

[evw143-B34] Hatfield SD , . 2005 . Stem cell division is regulated by the microRNA pathway . Nature 435 ( 7044 ): 974 – 978 . 15944714 10.1038/nature03816

[evw143-B35] Heimberg AM Cowper-Sal-lari R Semon M Donoghue PC Peterson KJ. 2010 . microRNAs reveal the interrelationships of hagfish, lampreys, and gnathostomes and the nature of the ancestral vertebrate . Proc Natl Acad Sci U S A. 107 : 19379 – 19383 . 20959416 10.1073/pnas.1010350107 PMC2984222

[evw143-B36] Heimberg AM Sempere LF Moy VN Donoghue PC Peterson KJ. 2008 . MicroRNAs and the advent of vertebrate morphological complexity . Proc Natl Acad Sci U S A. 105 : 2946 – 2950 . 18287013 10.1073/pnas.0712259105 PMC2268565

[evw143-B37] Helm C Bernhart SH Siederdissen CHZ Nickel B Bleidorn C. 2012 . Deep sequencing of small RNAs confirms an annelid affinity of Myzostomida . Mol Phylogenet Evol. 64 ( 1 ): 198 – 203 . 22724136 10.1016/j.ympev.2012.03.017

[evw143-B38] Hilbrant M Damen WGM McGregor AP. 2012 . Evolutionary crossroads in developmental biology: the spider *Parasteatoda tepidariorum.* Development 139 ( 15 ): 2655 – 2662 . 22782720 10.1242/dev.078204

[evw143-B39] Hoy MA. 2009 . The predatory mite *Metaseiulus occidentalis* : mitey small and mitey large genomes . Bioessays 31 ( 5 ): 581 – 590 . 19334003 10.1002/bies.200800175

[evw143-B40] Huminiecki L Conant GC. 2012 . Polyploidy and the evolution of complex traits . Int J Evol Biol. 2012 : 292068. 22900230 10.1155/2012/292068 PMC3413983

[evw143-B41] Innan H Kondrashov F. 2010 . The evolution of gene duplications: classifying and distinguishing between models . Nat Rev Genet. 11 : 97 – 108 . 20051986 10.1038/nrg2689

[evw143-B42] Janssen R , . 2010 . Conservation, loss, and redeployment of Wnt ligands in protostomes: implications for understanding the evolution of segment formation . BMC Evol Biol. 10 : 374. 21122121 10.1186/1471-2148-10-374 PMC3003278

[evw143-B43] Janssen R , . 2015 . The evolution and expression of panarthropod frizzled genes . Front Ecol Evol. 3 : 96.

[evw143-B44] Juvvuna PK Khandelia P Lee LM Makeyev EV. 2012 . Argonaute identity defines the length of mature mammalian miRNAs . Nucleic Acids Res. 40 ( 14 ): 6808 – 6820 . 22505576 10.1093/nar/gks293 PMC3413106

[evw143-B45] Kawamata T Yoda M Tomari Y. 2011 . Multilayer checkpoints for miRNA authenticity during RISC assembly . EMBO Rep . 12 ( 9 ): 944 – 949 . 21738221 10.1038/embor.2011.128 PMC3166454

[evw143-B46] Kenny , . 2016 . Ancestral whole-genome duplication in the marine chelicerate horseshoe crabs . Heredity . 116 ( 2 ): 190 – 199 . 26419336 10.1038/hdy.2015.89 PMC4806888

[evw143-B47] Kozomara A Griffiths-Jones S. 2014 . miRBase: annotating high confidence miRNAs using deep sequencing data . Nucleic Acids Res. 42 : D68 – D73 . 24275495 10.1093/nar/gkt1181 PMC3965103

[evw143-B48] Langmead B Trapnell C Pop M Salzberg SL. 2009 . Ultrafast and memory-efficient alignment of short DNA sequences to the human genome . Genome Biol. 10 : R25. 19261174 10.1186/gb-2009-10-3-r25 PMC2690996

[evw143-B49] Larkin MA , . 2007 . ClustalW and ClustalX version 2 . Bioinformatics 23 ( 21 ): 2947 – 2948 . 17846036 10.1093/bioinformatics/btm404

[evw143-B50] Lawson D Arensburger P Atkinson P Besansky NJ Bruggner RV. 2009 . VectorBase: a data resource for invertebrate vector genomics . Nucleic Acids Res. 37 ( Database issue ): D583 – D587 . 19028744 10.1093/nar/gkn857 PMC2686483

[evw143-B51] Li J Musso G Zhang Z. 2008 . Preferential regulation of duplicated genes by microRNAs in mammals . Genome Biol. 9 ( 8 ): R132. 18727826 10.1186/gb-2008-9-8-r132 PMC2575522

[evw143-B52] Liu B , . 2014 . An analysis of the small RNA transcriptome of four developmental stages of the citrus red mite ( *Panonychus citri* ) . Insect Mol Biol. 23 ( 2 ): 216 – 229 . 24330037 10.1111/imb.12075

[evw143-B53] Luo J , . 2015 . Identification and characterization of microRNAs by deep-sequencing in *Hyalomma anatolicum anatolicum* (Acari: Ixodidae) ticks . Gene 564 ( 2 ): 125 – 133 . 25592818 10.1016/j.gene.2015.01.019

[evw143-B54] Marco A Hooks K Griffiths-Jones S. 2012 . Evolution and function of the extended miR-2 microRNA family . RNA Biol. 9 ( 3 ): 242 – 248 . 22336713 10.4161/rna.19160 PMC3384581

[evw143-B55] Marco A Hui JH Ronshaugen M Griffiths-Jones S. 2010 . Functional shifts in insect miRNA evolution . Genome Biol Evol. 2 : 686 – 696 . 20817720 10.1093/gbe/evq053 PMC2956262

[evw143-B56] Marco A Macpherson JI Ronshaugen M Griffiths-Jones S. 2012 . MicroRNAs from the same precursor have different targeting properties . Silence 3 ( 1 ): 8. 23016695 10.1186/1758-907X-3-8 PMC3503882

[evw143-B57] Marco A Ninova M Ronshaugen M Griffiths-Jones S. 2013 . Clusters of microRNAs emerge by new hairpins in existing transcripts . Nucleic Acids Res. 41 ( 16 ): 7745 – 7752 . 23775791 10.1093/nar/gkt534 PMC3763532

[evw143-B58] Marco A. 2014 . Sex-biased expression of microRNAs in *Drosophila melanogaster.* Open Biol. 4 : 140024. 24694940 10.1098/rsob.140024 PMC4043116

[evw143-B59] Martin M. 2011 . Cutadapt removes adapter sequences from high-throughput sequencing reads . EMBnet J. 17 : 10 – 12 .

[evw143-B60] Massirer KB Perez SG Mondol V Pasquinelli AE. 2012 . The miR-35-41 Family of MiRNAs regulates RNAi Sensitivity in *Caenorhabditis elegans.* PLoS Genet. 8 ( 3 ): e1002536. 22412382 10.1371/journal.pgen.1002536 PMC3297572

[evw143-B61] McGregor AP , . 2008 . *Cupiennies salei* and *Achaearanea tepidariorum* : spider model for investigating evolution and development . Bioessays 30 ( 5 ): 487 – 498 . 18404731 10.1002/bies.20744

[evw143-B62] McJunkin K Ambros V. 2014 . The embryonic *mir-35* family of miRNAs promotes multiple aspects of fecundity in *Caenorhabditis elegans.* G3 4 : 1747 – 1754 . 25053708 10.1534/g3.114.011973 PMC4169167

[evw143-B63] Mittmann B Wolff C. 2012 . Embryonic development and staging of the cobweb spider *Parasteatoda tepidariorum* C. L. Koch, 1841 (syn.: *Achaearanea tepidariorum* ; Araneomorphae; Theridiidae) . Dev Genes Evol. 222 : 189 – 216 . 22569930 10.1007/s00427-012-0401-0

[evw143-B64] Mohammed J Bortolamiol-Becet D , . 2014 . Adaptive evolution of testis-specific, recently evolved, clustered miRNAs in *Drosophila.* RNA 20 ( 8 ): 1195 – 1209 . 24942624 10.1261/rna.044644.114 PMC4105746

[evw143-B65] Mohammed J Siepel A Lai EC. 2014 . Diverse modes of evolutionary emergence and flux of conserved microRNA clusters . RNA 20 ( 12 ): 1850 – 1863 . 25332374 10.1261/rna.046805.114 PMC4238352

[evw143-B66] Nawrocki EP Kolbe DL Eddy SR. 2009 . Infernal 1.0: inference of RNA alignments . Bioinformatics 25 : 1335 – 1337 . 19307242 10.1093/bioinformatics/btp157 PMC2732312

[evw143-B67] Ninova M Ronshaugen M Griffiths-Jones S. 2014 . Conserved temporal patterns of microRNA expression in *Drosophila* support a developmental hourglass model . Genome Biol Evol. 6 ( 9 ): 2459 – 2467 . 25169982 10.1093/gbe/evu183 PMC4202322

[evw143-B68] Nossa CW , . 2014 . Joint assembly and genetic mapping of the Atlantic Horseshoe crab genome reveals ancient whole genome duplication . Gigascience 3 : 9. 24987520 10.1186/2047-217X-3-9 PMC4066314

[evw143-B69] Ohno S. 1970 . Evolution by gene duplication . Berlin: Springer-Verlag .

[evw143-B70] Okamura K , . 2008 . The regulatory activity of microRNA* species has substantial influence on microRNA and 3' UTR evolution . Nat Struct Mol Biol. 15 ( 4 ): 354 – 363 . 18376413 10.1038/nsmb.1409 PMC2698667

[evw143-B71] Peterson KJ Dietrich MR McPeek MA. 2009 . MicroRNAs and metazoan macroevolution: insights into canalization, complexity, and the *Cambrian* explosion . BioEssays 31 : 736 – 747 . 19472371 10.1002/bies.200900033

[evw143-B72] Pink RC , . 2015 . The passenger strand, miR-21-3p, plays a role in mediating cisplatin resistance in ovarian cancer cells . Gynecol Oncol . 137 ( 1 ): 143 – 151 . 25579119 10.1016/j.ygyno.2014.12.042

[evw143-B73] Posnien N , . 2014 . A Comprehensive reference transcriptome resource for the common house spider *Parasteatoda tepidariorum.* PLoS One 9 ( 8 ): e104885. 25118601 10.1371/journal.pone.0104885 PMC4132015

[evw143-B74] Rota-Stabelli O , . 2011 . A congruent solution to arthropod phylogeny: phylogenomics, miRNAs and morphology support monophyletic mandibulata . Proc Biol Sci R Soc . 278 ( 1703 ): 298 – 306 . 10.1098/rspb.2010.0590 PMC3013382 20702459

[evw143-B75] Rota-Stabelli O Daley AC Pisani D. 2013 . Molecular timetrees reveal a cambrian colonization of land and a new scenario for ecdysozoan evolution . Curr Biol. 23 ( 5 ): 392 – 398 . 23375891 10.1016/j.cub.2013.01.026

[evw143-B76] Saito K , . 2006 . Specific association of Piwi with rasiRNAs derived from retrotransposon and heterochromatic regions in the *Drosophila* genome . Genes Dev. 20 ( 16 ): 2214 – 2222 . 16882972 10.1101/gad.1454806 PMC1553205

[evw143-B77] Sanggaard KW Bechsgaard JS Fang X Duan J Dyrlund TF. 2014 . Spider genomes provide insight into composition and evolution of venom and silk . Nat Commun. 5 : 3765. 24801114 10.1038/ncomms4765 PMC4273655

[evw143-B78] Schwager EE Schönauer A Leite DJ Sharma PP McGregor AP. 2015 . Chelicerata . In: Wanninger A , editors. Evolutionary developmental biology of invertebrates . Berlin : Spinger . p. 99 – 139 .

[evw143-B79] Schwager EE Schoppmeier M Pechmann M Damen WG. 2007 . Duplicated hox genes in the spider *Cupiennius salei.* Front Zool . 13 ( 4 ): 10. 10.1186/1742-9994-4-10 PMC1838909 17355624

[evw143-B80] Sharma PP Gupta T Schwager EE Wheeler WC Extavour CG. 2014 . Subdivision of arthropod cap-n-collar expression domains is restricted to mandibulata . Evodevo 5 ( 1 ): 3. 24405788 10.1186/2041-9139-5-3 PMC3897911

[evw143-B81] Sharma PP Kaluziak ST , . 2014 . Phylogenomic interrogation of arachnida reveals systemic conflicts in phylogenetic signal . Mol Biol Evol. 31 ( 11 ): 2963 – 2984 . 25107551 10.1093/molbev/msu235

[evw143-B82] Sharma PP Santiago MA González-Santillán E Monod L Wheeler WC. 2015 . Evidence of duplicated hox genes in the most recent common ancestor of extant scorpions . Evol Dev. 17 ( 6 ): 347 – 355 . 26492826 10.1111/ede.12166

[evw143-B83] Sharma PP Schwager EE Extavour CG Wheeler WC. 2014 . Hox gene duplications correlate with posterior heteronomy in scorpions . Proc Biol Sci. 281 ( 1792 ):pii: 20140661. 25122224 10.1098/rspb.2014.0661 PMC4150311

[evw143-B84] Sievers F , . 2011 . Fast, scalable generation of high-quality protein multiple sequence alignments using clustal omega . Mol Syst Biol. 7 : 539. 21988835 10.1038/msb.2011.75 PMC3261699

[evw143-B85] Sokol NS Xu P Jan YN Ambros V. 2008 . Drosophila let-7 microRNA is required for remodeling of the neuromusculature during metamorphosis . Genes Dev. 22 : 1591 – 1596 . 18559475 10.1101/gad.1671708 PMC2428057

[evw143-B86] Stark A , . 2007 . Systematic discovery and characterization of fly miRNAs using 12 *Drosophila* genomes . Genome Res. 17 ( 12 ): 1865 – 1879 . 17989255 10.1101/gr.6593807 PMC2099594

[evw143-B87] Tarver JE , . 2015 . microRNAs and the evolution of complex multicellularity: identification of a large, diverse complement of microRNAs in the brown alga *Ectocarpus.* Nucleic Acids Res. 43 ( 13 ): 6384 – 6398 . 26101255 10.1093/nar/gkv578 PMC4513859

[evw143-B88] Tarver JE , . 2013 . miRNAs: small genes with big potential in metazoan phylogenetics . Mol Biol Evol. 30 ( 11 ): 2369 – 2382 . 23913097 10.1093/molbev/mst133

[evw143-B89] Taylor JS Raes J. 2004 . Duplication and divergence: the evolution of new genes and old genes . Ann Rev Genet. 38 : 615 – 643 . 15568988 10.1146/annurev.genet.38.072902.092831

[evw143-B90] Tribolium Genome Sequencing Consortium , . 2008 . The genome of the model beetle and pest *Tribolium castaneum.* Nature 452 ( 7190 ): 949 – 955 . 18362917 10.1038/nature06784

[evw143-B91] Turetzek N Pechmann M Schomburg C Schneider J Prpic NM. 2015 . Neofunctionalization of a duplicate dachshund gene underlies the evolution of a novel leg segment in arachnids . Mol Biol Evol. pii: msv200 . 10.1093/molbev/msv200 26443673

[evw143-B92] Wang S Adams KL. 2015 . Duplicate gene divergence by changes in microRNA binding sites in *Arabidopsis* and *Brassica.* Genome Biol Evol. 7 ( 3 ): 646 – 655 . 25644246 10.1093/gbe/evv023 PMC5322543

[evw143-B93] Wheeler BM , . 2009 . The deep evolution of metazoan microRNAs . Evol Dev. 11 ( 1 ): 50 – 68 . 19196333 10.1111/j.1525-142X.2008.00302.x

[evw143-B94] Wiegmann BM , . 2011 . Episodic radiations in the fly tree of life . Proc Natl Acad Sci U S A. 108 ( 14 ): 5690 – 5695 . 21402926 10.1073/pnas.1012675108 PMC3078341

[evw143-B95] Wong SF , . 2015 . Independent regulation of vertebral number and vertebral identity by microRNA-196 paralogs . Proc Natl Acad Sci U S A. 112 ( 35 ): E4884 – E4893 . 26283362 10.1073/pnas.1512655112 PMC4568285

[evw143-B96] Yang JS , . 2014 . Intertwined pathways for argonaute-mediated microRNA biogenesis in *Drosophila.* Nucleic Acids Res. 42 ( 3 ): 1987 – 2002 . 24220090 10.1093/nar/gkt1038 PMC3919586

[evw143-B97] Zhou H , . 2010 . miR-155 and its star-form partner miR-155* cooperatively regulate type I interferon production by human plasmacytoid dendritic cells . Blood 116 ( 26 ): 5885 – 5894 . 20852130 10.1182/blood-2010-04-280156

[evw143-B98] Zhou J Zhou Y Cao J Zhang H Yu Y. 2013 . Distinctive microRNA profiles in the salivary glands of *Haemaphysalis longicornis* related to tick blood-feeding . Exp Appl Acarol . 59 ( 3 ): 339 – 349 . 22918721 10.1007/s10493-012-9604-3

