# Supplementary material for: Pervasive microRNA Duplication in Chelicerates: Insights from the Embryonic microRNA Repertoire of the Spider Parasteatoda tepidariorum
Source: Genome Biol Evol. 2016 Jun 19;8(7):2133–44. doi: 10.1093/gbe/evw143 (PMC4987109; doi:10.1093/gbe/evw143)
Supplement: Supplementary Data [file gbe_8_7_evw143_s1.zip › File S2.pdf]

microRNA	miRDeep2 ID
pte-bantam	Scaffold2919_38102
pte-iab-4-1	Scaffold98_3281
pte-iab-4-2	Scaffold985_20492
pte-iab-8-1	Scaffold98_3254
pte-iab-8-2	Scaffold985_20495
pte-let-7	Scaffold1373_25319
pte-mir-1	Scaffold160_4618
pte-mir-10	Scaffold2609_36101
pte-mir-100a	Scaffold1373_25321
pte-mir-100b	Scaffold590_13964
pte-mir-11938	Scaffold166_4787
pte-mir-11939	Scaffold2190_33757
pte-mir-11940	Scaffold2654_36587
pte-mir-11941	Scaffold384_9844
pte-mir-11942a	Scaffold71_2398
pte-mir-11942b	Scaffold1382_25399
pte-mir-11942c	Scaffold1613_28433
pte-mir-11942d	Scaffold1613_28435
pte-mir-11943	Scaffold569_13469
pte-mir-11944	Scaffold273_7121
pte-mir-11945	Scaffold305_8052
pte-mir-11946	Scaffold2928_38140
pte-mir-11947a-1	Scaffold2369_34880
pte-mir-11947a-2	Scaffold1034_21346
pte-mir-11947b-1	Scaffold705_16398
pte-mir-11947b-2	Scaffold504_12208
pte-mir-11948	Scaffold2690_36842
pte-mir-11949	Scaffold4736_44077
pte-mir-11950	Scaffold248_6752
pte-mir-11951a	Scaffold6_385
pte-mir-11951b	Scaffold6_387
pte-mir-11952	Scaffold387_9879
pte-mir-11953	Scaffold2022_32019
pte-mir-11954	Scaffold183_5388
pte-mir-11955	Scaffold503_12154
pte-mir-11956	Scaffold5373_44911
pte-mir-11957	Scaffold504_12210
pte-mir-11958	Scaffold875_18876
pte-mir-11959-1	Scaffold8511_46167
pte-mir-11959-2	Scaffold875_18880
pte-mir-11960	Scaffold303_7878
pte-mir-11961a	Scaffold3797_41813
pte-mir-11961b	Scaffold3797_41810
pte-mir-11961c	Scaffold3797_41812
pte-mir-11962a	Scaffold331_8518
pte-mir-11962b	Scaffold331_8520
pte-mir-11963a	Scaffold3197_39489
pte-mir-11963b-1	Scaffold672_15895
pte-mir-11963b-2	Scaffold289_7580
pte-mir-11964	Scaffold2350_34693
pte-mir-11965-1	Scaffold297_7804

microRNA	miRDeep2 ID
pte-mir-11965-2	Scaffold297_7775
pte-mir-11965-3	Scaffold610_14492
pte-mir-11966	Scaffold473_11393
pte-mir-11967	Scaffold4099_42824
pte-mir-11968-1	Scaffold2482_35325
pte-mir-11968-2	Scaffold797_17806
pte-mir-125a	Scaffold1373_25317
pte-mir-125b	Scaffold1466_26411
pte-mir-14	Scaffold1644_28658
pte-mir-184-1	Scaffold313_8156
pte-mir-184-2	Scaffold390_9906
pte-mir-190	Scaffold2173_33652
pte-mir-193a	Scaffold708_16399
pte-mir-193b	Scaffold171_5150
pte-mir-210	Scaffold22_817
pte-mir-263a	Scaffold133_4000
pte-mir-263a	Scaffold133_3996
pte-mir-275	Scaffold290_7704
pte-mir-276-1	Scaffold2464_35273
pte-mir-276-2	Scaffold55_1663
pte-mir-277a	Scaffold33_1067
pte-mir-277b	Scaffold633_15234
pte-mir-278a	Scaffold1260_24209
pte-mir-278b	Scaffold605_14471
pte-mir-279	Scaffold237_6201
pte-mir-281	Scaffold1827_30268
pte-mir-29a	Scaffold395_9965
pte-mir-29b	Scaffold395_9963
pte-mir-2a	Scaffold300_7829
pte-mir-2b-1	Scaffold300_7831
pte-mir-2b-2	Scaffold2425_35140
pte-mir-2c	Scaffold2425_35142
pte-mir-2d	Scaffold2425_35134
pte-mir-2e	Scaffold2425_35144
pte-mir-2f	Scaffold300_7827
pte-mir-2g-1	Scaffold2425_35136
pte-mir-2g-2	Scaffold2425_35138
pte-mir-305	Scaffold290_7701
pte-mir-315	Scaffold38_1202
pte-mir-317	Scaffold633_15236
pte-mir-34	Scaffold633_15231
pte-mir-3477	Scaffold442_10771
pte-mir-375-1	Scaffold15_622
pte-mir-375-2	Scaffold1849_30604
pte-mir-3791a	Scaffold248_6742
pte-mir-3791b	Scaffold248_6738
pte-mir-3791c	Scaffold248_6750
pte-mir-3791d	Scaffold248_6744
pte-mir-3791e-1	Scaffold248_6724
pte-mir-3791e-2	Scaffold5285_44881
pte-mir-3791f	Scaffold248_6756

microRNA	miRDeep2 ID
pte-mir-3791g	Scaffold248_6746
pte-mir-3791h	Scaffold248_6748
pte-mir-3791i	Scaffold248_6758
pte-mir-3791j-1	Scaffold248_6726
pte-mir-3791j-2	Scaffold5285_44879
pte-mir-3791k	Scaffold248_6728
pte-mir-3791l	Scaffold6623_45530
pte-mir-3791m	Scaffold1083_21832
pte-mir-3791n-1	Scaffold29666_47930
pte-mir-3791n-2	Scaffold5285_44883
pte-mir-3791o-1	Scaffold6623_45532
pte-mir-3791o-2	Scaffold1083_21842
pte-mir-3791p	Scaffold5285_44874
pte-mir-3791q-1	Scaffold5285_44887
pte-mir-3791q-2	Scaffold29666_47928
pte-mir-3791q-3	Scaffold5285_44885
pte-mir-3791r-1	Scaffold5285_44889
pte-mir-3791r-2	Scaffold27700_47820
pte-mir-3791r-3	Scaffold248_6722
pte-mir-3791s	Scaffold2773_37223
pte-mir-3791t	Scaffold1083_21830
pte-mir-3791u	Scaffold1083_21834
pte-mir-3791v	Scaffold1083_21836
pte-mir-3791w	Scaffold6623_45528
pte-mir-3791x	Scaffold1083_21844
pte-mir-3791y	Scaffold1083_21840
pte-mir-3791z	Scaffold248_6765
pte-mir-3931	Scaffold13_568
pte-mir-7	Scaffold1792_29947
pte-mir-71-1	Scaffold2425_35146
pte-mir-71-2	Scaffold300_7825
pte-mir-745	Scaffold17_681
pte-mir-8	Scaffold406_10365
pte-mir-87a	Scaffold15_617
pte-mir-87b	Scaffold15_615
pte-mir-87c	Scaffold31_1027
pte-mir-9	Scaffold1824_30216
pte-mir-92a	Scaffold1813_30090
pte-mir-92b-1	Scaffold76_2597
pte-mir-92b-2	Scaffold774_17511
pte-mir-92c	Scaffold260_6921
pte-mir-96	Scaffold133_4002
pte-mir-981	Scaffold3_108
pte-mir-993a	Scaffold59_1802
pte-mir-993b-1	Scaffold2243_34190
pte-mir-993b-2	Scaffold19_714

Provisional ID : Scaffold2919_38102
Score total : 2794.2
Score for star read(s) : 3.9
Score for read counts : 2787.9
Score for mfe : 0.8
Score for randfold : 1.6
Score for cons. seed :
Total read count : 5480
Mature read count : 5476
Loop read count : 0
Star read count : 4

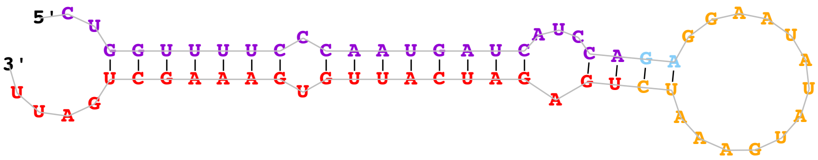

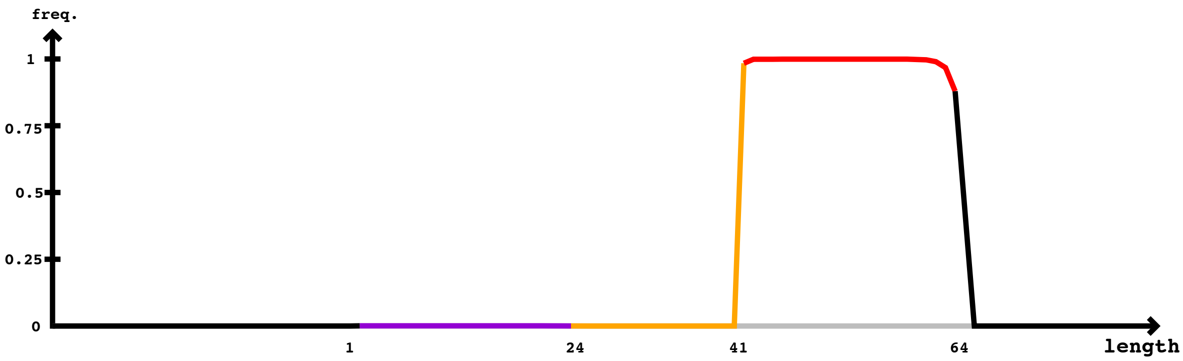

Star Mature

```
5'- ugaaagaaggaagcucaagauuagacgaaacugguuuucccaaugaucauccagaggaauauaugaaaucugagaucauugugaaagcugauuuuguugcaccuuccaccucu   -3'  obs
    ugaaagaaggaagcucaagauuagacgaaacugguuuucccaaugaucauccagaggaauauaugaaaucugagaucauugugaaagcugauuuuguugcaccuuccaccucu        exp
    .....(((((..((.........(((((((..(((((((.((((((((...((((.............)))).)))))))).)))))))..))))))))).)))))........   reads   mm   sample
    ......................................................................ugagaucauugugaaagc.........................   1       0    S05
    ......................................................................ugagaucauugugaaagcug.......................   1       0    S05
    ......................................................................ugagaucauugugaaagcuga......................   6       0    S05
    ......................................................................ugagaucauugugaaagcugau.....................   13      0    S05
    ......................................................................ugagaucauugugaaagcugauu....................   35      0    S05
    ......................................................................ugagaucauugugaaagcugauuu...................   4       0    S05
    .......................................................................gagaucauugugaaagcugau.....................   2       0    S05
    .......................................................................gagaucauugugaaagcugauu....................   1       0    S05

    ..............................cugguuuucccaaugaucaucca............................................................   1       0    S10
    ......................................................................ugagaucauugugaaagc.........................   1       0    S10
    ......................................................................ugagaucauugugaaagcu........................   3       0    S10
    ......................................................................ugagaucauugugaaagcug.......................   11      0    S10
    ......................................................................ugagaucauugugaaagcuga......................   26      0    S10
    ......................................................................ugagaucauugugaaagcugau.....................   123     0    S10
    ......................................................................ugagaucauugugaaagcugauu....................   1891    0    S10
    ......................................................................ugagaucauugugaaagcugauuu...................   89      0    S10
    .......................................................................gagaucauugugaaagcugau.....................   2       0    S10
    .......................................................................gagaucauugugaaagcugauu....................   35      0    S10
    ..........................................................................aucauugugaaagcugauu....................   1       0    S10

    ..............................cugguuuucccaaugaucau...............................................................   1       0    S06
    ......................................................................ugagaucauugugaaagcuga......................   5       0    S06
    ......................................................................ugagaucauugugaaagcugau.....................   19      0    S06
    ......................................................................ugagaucauugugaaagcugauu....................   44      0    S06
    ......................................................................ugagaucauugugaaagcugauuu...................   8       0    S06
    .......................................................................gagaucauugugaaagcugauu....................   1       0    S06

    ......................................................................ugagaucauugugaaagc.........................   1       0    S01
    ......................................................................ugagaucauugugaaagcug.......................   3       0    S01
    ......................................................................ugagaucauugugaaagcuga......................   4       0    S01
    ......................................................................ugagaucauugugaaagcugau.....................   12      0    S01
    ......................................................................ugagaucauugugaaagcugauu....................   32      0    S01
```

Star / Mature

```
                              Star                                    Mature
ugaaagaaggaagcucaagauuagacgaaacugguuuucccaaugaucauccagaggaauauaugaaaucugagaucauugugaaagcugauuuuguugcaccuuccaccucu
......................................................................ugagaucauugugaaagcugauuu...................	2	0	S01

......................................................................ugagaucauugugaaagcug.......................	1	0	S07
......................................................................ugagaucauugugaaagcuga......................	9	0	S07
......................................................................ugagaucauugugaaagcugau.....................	66	0	S07
......................................................................ugagaucauugugaaagcugauu....................	265	0	S07
......................................................................ugagaucauugugaaagcugauuu...................	27	0	S07
.......................................................................gagaucauugugaaagcugauu....................	3	0	S07

......................................................................ugagaucauugugaaagcug.......................	2	0	S03
......................................................................ugagaucauugugaaagcuga......................	5	0	S03
......................................................................ugagaucauugugaaagcugau.....................	11	0	S03
......................................................................ugagaucauugugaaagcugauu....................	15	0	S03

..............................cugguuuucccaaugaucauc..............................................................	1	0	S09
..............................cugguuuucccaaugaucaucc.............................................................	1	0	S09
......................................................................ugagaucauugugaaagc.........................	2	0	S09
......................................................................ugagaucauugugaaagcu........................	3	0	S09
......................................................................ugagaucauugugaaagcug.......................	11	0	S09
......................................................................ugagaucauugugaaagcuga......................	46	0	S09
......................................................................ugagaucauugugaaagcugau.....................	166	0	S09
......................................................................ugagaucauugugaaagcugauu....................	1933	0	S09
......................................................................ugagaucauugugaaagcugauuu...................	186	0	S09
.......................................................................gagaucauugugaaagcugau.....................	2	0	S09
.......................................................................gagaucauugugaaagcugauu....................	30	0	S09
.......................................................................gagaucauugugaaagcugauuu...................	3	0	S09

......................................................................ugagaucauugugaaagcug.......................	7	0	S08
......................................................................ugagaucauugugaaagcuga......................	8	0	S08
......................................................................ugagaucauugugaaagcugau.....................	31	0	S08
......................................................................ugagaucauugugaaagcugauu....................	122	0	S08
......................................................................ugagaucauugugaaagcugauuu...................	24	0	S08
.......................................................................gagaucauugugaaagcugau.....................	1	0	S08
.......................................................................gagaucauugugaaagcugauu....................	1	0	S08
.......................................................................gagaucauugugaaagcugauuu...................	1	0	S08
..........................................................................aucauugugaaagcugauu....................	1	0	S08

......................................................................ugagaucauugugaaagcug.......................	1	0	S02
......................................................................ugagaucauugugaaagcuga......................	8	0	S02
......................................................................ugagaucauugugaaagcugau.....................	25	0	S02
......................................................................ugagaucauugugaaagcugauu....................	30	0	S02
......................................................................ugagaucauugugaaagcugauuu...................	2	0	S02

......................................................................ugagaucauugugaaagc.........................	2	0	S04
......................................................................ugagaucauugugaaagcu........................	1	0	S04
......................................................................ugagaucauugugaaagcug.......................	1	0	S04
......................................................................ugagaucauugugaaagcuga......................	5	0	S04
......................................................................ugagaucauugugaaagcugau.....................	11	0	S04
......................................................................ugagaucauugugaaagcugauu....................	28	0	S04
......................................................................ugagaucauugugaaagcugauuu...................	4	0	S04
```

Provisional ID	:	Scaffold98_3281
Score total	:	1.1
Score for star read(s)	:	-1.3
Score for read counts	:	0
Score for mfe	:	0.8
Score for randfold	:	1.6
Score for cons. seed	:	
Total read count	:	697
Mature read count	:	697
Loop read count	:	0
Star read count	:	0

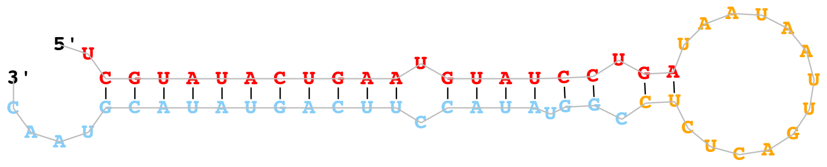

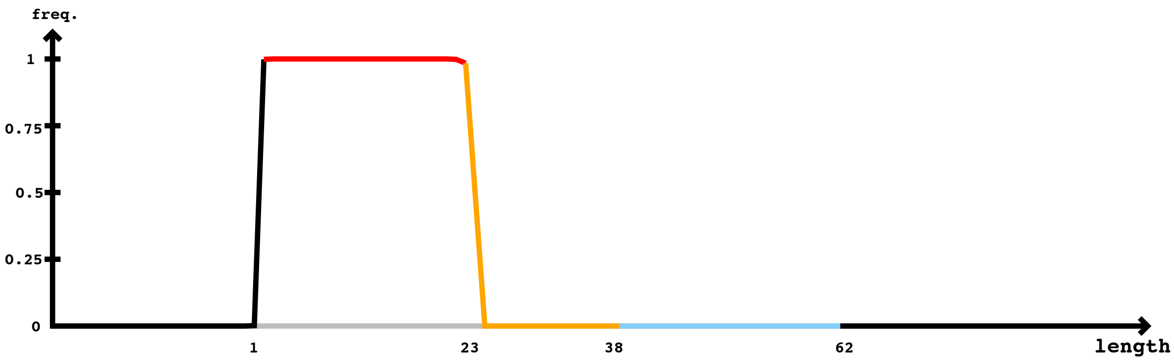

Mature Star

```
5'- gaaaauuaagagaauucuguucguauacugaauguauccugauaauaaauugacucuccgguauaccuucaguauacguaacagaaccaucuaaaaaucuucacuuacguuga   -3'    exp
    (((.(((.(((...((((((((((((((((.((((((.((.............)).)).)))).)))))))))))).)))))))...)))...))).)))...........    reads      mm          sample
    ....................ucguauacugaauguauccuga.......................................................................    1          0           S05

    ....................ucguauacugaauguauccu.........................................................................    1          0           S09
    ....................ucguauacugaauguauccug........................................................................    3          0           S09
    ....................ucguauacugaauguauccuga.......................................................................    342        0           S09
    ....................ucguauacugaauguauccugau......................................................................    2          0           S09

    ....................ucguauacugaauguauccuga.......................................................................    2          0           S03

    ...................uucguauacugaauguauccug........................................................................    1          0           S10
    ....................ucguauacugaauguauccug........................................................................    6          0           S10
    ....................ucguauacugaauguauccuga.......................................................................    337        0           S10
    ....................ucguauacugaauguauccugau......................................................................    1          0           S10
    .....................cguauacugaauguauccuga.......................................................................    1          0           S10
```

Provisional ID	:	Scaffold985_20492
Score total	:	1.8
Score for star read(s)	:	-1.3
Score for read counts	:	0
Score for mfe	:	1.5
Score for randfold	:	1.6
Score for cons. seed	:	
Total read count	:	695
Mature read count	:	695
Loop read count	:	0
Star read count	:	0

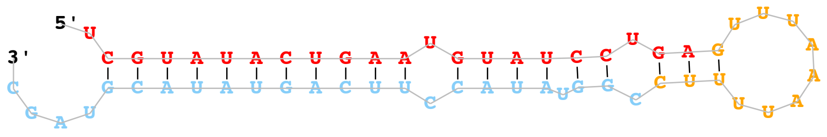

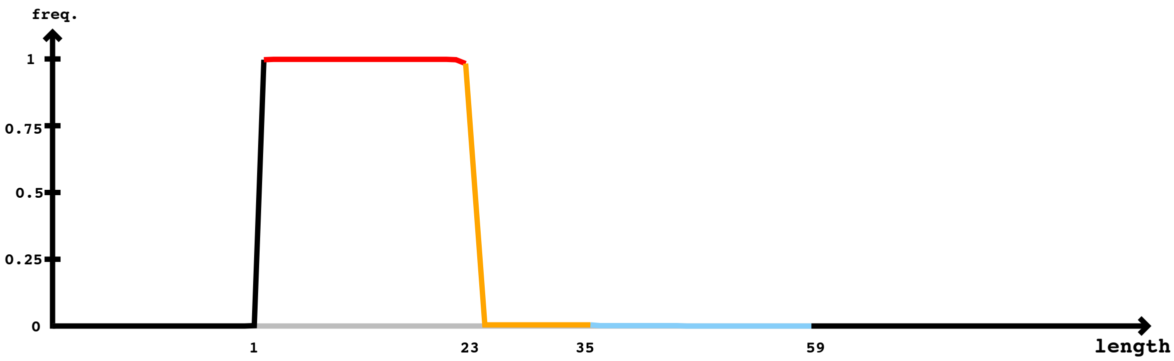

Mature Star

```
5'- gcuaguuucaaggcaccuguucguauacugaauguauccugaguuuaaauuuuccgguauaccuucaguauacguagcagggaaccauggcuuacgucugauucauauccgc  -3'    exp
    ....(..((..((..(((((((((((((((.((((((.(((........))).)).)))).))))))))))).))))))...))..(((....))).))..)........  reads   mm   sample
    ....................ucguauacugaauguauccu........................................................................  1       0    S09
    ....................ucguauacugaauguauccug.......................................................................  3       0    S09
    ....................ucguauacugaauguauccuga......................................................................  342     0    S09
    ....................ucguauacugaauguauccugag.....................................................................  1       0    S09
    ....................ucguauacugaauguauccugaguuuaaauuuucc.........................................................  2       0    S09
    ..........................................guuuaaauuuuccgguauaccu................................................  1       0    S09

    ....................ucguauacugaauguauccuga......................................................................  1       0    S05

    ....................ucguauacugaauguauccuga......................................................................  2       0    S03

    ...................uucguauacugaauguauccug.......................................................................  1       0    S10
    ....................ucguauacugaauguauccug.......................................................................  6       0    S10
    ....................ucguauacugaauguauccuga......................................................................  337     0    S10
    .....................cguauacugaauguauccuga......................................................................  1       0    S10
```

Provisional ID	:	Scaffold98_3254
Score total	:	35.3
Score for star read(s)	:	3.9
Score for read counts	:	28.2
Score for mfe	:	1.6
Score for randfold	:	1.6
Score for cons. seed	:	
Total read count	:	67
Mature read count	:	36
Loop read count	:	0
Star read count	:	31

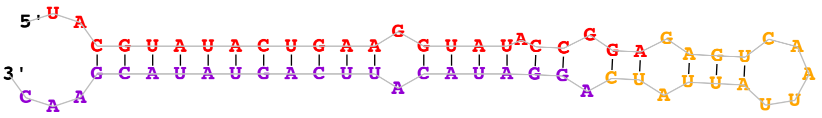

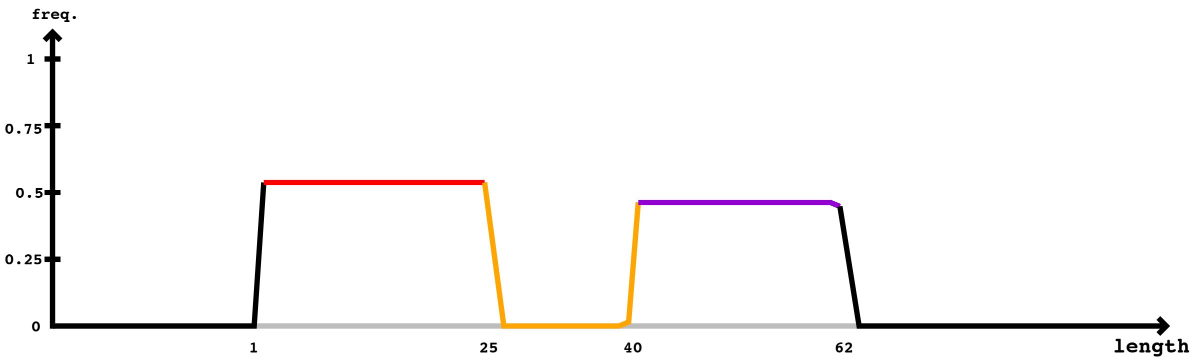

Mature **Star**

```
5'- agauuuuuagaugguucuguuacguauacugaagguauaccggagagucaauuauuaucaggauacauucaguauacgaacagaauucucuuaauuuucgucuaagccuggaug   -3'    obs
    agauuuuuagaugguucuguuacguauacugaagguauaccggagagucaauuauuaucaggauacauucaguauacgaacagaauucucuuaauuuucgucuaagccuggaug          exp
    ((((...(((..((((((((.((((((((((.((((.((.((.(((.....))).)).)))))).))))))))))))))))))))..))).))))).((((((....))))))   reads      mm      sample
    ....................uacguauacugaagguauaccgga......................................................................   26         0       S10
    ..........................................................caggauacauucaguauacgaac.................................   1          0       S10
    ...........................................................aggauacauucaguauacgaac.................................   16         0       S10

    ....................uacguauacugaagguauaccgga......................................................................   9          0       S09
    ...........................................................aggauacauucaguauacgaa..................................   1          0       S09
    ...........................................................aggauacauucaguauacgaac.................................   12         0       S09

    ....................uacguauacugaagguauaccgga......................................................................   1          0       S05

    ...........................................................aggauacauucaguauacgaac.................................   1          0       S02
```

Provisional ID	:	Scaffold985_20495
Score total	:	32.2
Score for star read(s)	:	3.9
Score for read counts	:	29.7
Score for mfe	:	0.8
Score for randfold	:	-2.2
Score for cons. seed	:	
Total read count	:	70
Mature read count	:	39
Loop read count	:	0
Star read count	:	31

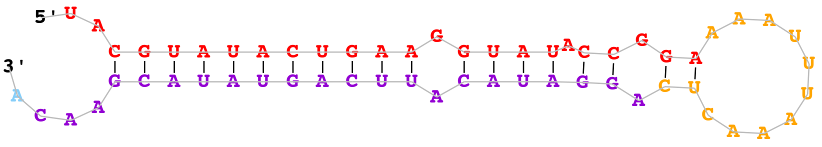

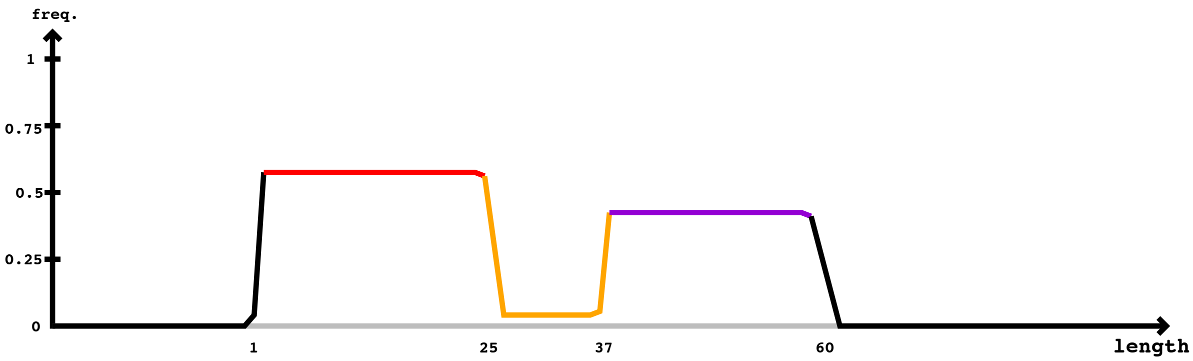

Mature Star

```
5'- cguaagccaugguucccugcuacguauacugaagguauaccggaaaauuuaaacucaggauacauucaguauacgaacaggugccuugaaacuagcaucauucgucgauuggaa  -3'  obs
    cguaagccaugguucccugcuacguauacugaagguauaccggaaaauuuaaacucaggauacauucaguauacgaacaggugccuugaaacuagcaucauucgucgauuggaa       exp
    .((.((.((.(((..((((...(((((((((((.((((.((.((..........)).)))))).)))))))))))..)))).))).))...)).))....((((.....)))).   reads  mm  sample
    ........................................................aggauacauucaguauacgaac....................................   1      0   S02

    ...................cuacguauacugaagguauaccgg.......................................................................   1      0   S10
    ...................cuacguauacugaagguauaccgga......................................................................   1      0   S10
    ....................uacguauacugaagguauaccgga......................................................................   26     0   S10
    ....................uacguauacugaagguauaccggaaaauuuaaacuc..........................................................   1      0   S10
    .......................................................caggauacauucaguauacgaac....................................   1      0   S10
    ........................................................aggauacauucaguauacgaac....................................   16     0   S10

    ...................cuacguauacugaagguauaccgga......................................................................   1      0   S09
    ....................uacguauacugaagguauaccgga......................................................................   9      0   S09
    ....................uacguauacugaagguauaccggaaaauuuaaacuc..........................................................   2      0   S09
    ........................................................aggauacauucaguauacgaa.....................................   1      0   S09
    ........................................................aggauacauucaguauacgaac....................................   12     0   S09

    ....................uacguauacugaagguauaccgga......................................................................   1      0   S05
```

Provisional ID	:	Scaffold1373_25319
Score total	:	175.7
Score for star read(s)	:	3.9
Score for read counts	:	168.4
Score for mfe	:	1.8
Score for randfold	:	1.6
Score for cons. seed	:	
Total read count	:	342
Mature read count	:	308
Loop read count	:	2
Star read count	:	32

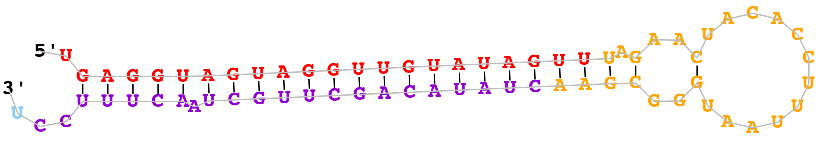

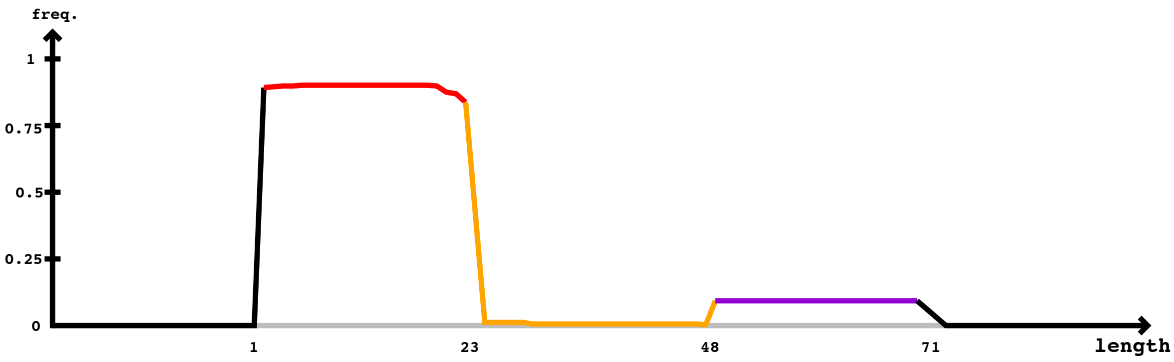

Mature **Star**

```
5'- guuaugguauguuuucgaugugagguaguagguuguauaguuuagaacuacaccuuuaaugggcgaacuauacagcuugcuaacuuuccucgauacaugccgauugcaucuu   -3'    obs
    guuaugguauguuuucgaugugagguaguagguuguauaguuuagaacuacaccuuuaaugggcgaacuauacagcuugcuaacuuuccucgauacaugccgauugcaucuu          exp
    ((..((((((((..((((.(..(((((((((((((((((((((.(..(............)..)))))))))))))))))).))))..))))).))))))))...)).....   reads   mm   sample
    ....................ugagguaguagguuguaua.........................................................................   1       0    S03
    ....................ugagguaguagguuguauagu.......................................................................   1       0    S03
    ....................ugagguaguagguuguauaguu......................................................................   36      0    S03
    ....................ugagguaguagguuguauaguuu.....................................................................   1       0    S03
    ....................ugagguaguagguuguauaguuuagaac................................................................   1       0    S03
    ......................agguaguagguuguauaguuuagaac................................................................   1       0    S03
    ...................................................................cuauacagcuugcuaacuuucc.......................   6       0    S03
    ...................................................................cuauacagcuugcuaacuuuccu......................   2       0    S03

    ....................ugagguaguagguuguauaguu......................................................................   4       0    S09
    ....................ugagguaguagguuguauaguuu.....................................................................   2       0    S09
    ...................................................................cuauacagcuugcuaacuuucc.......................   2       0    S09
    ...................................................................cuauacagcuugcuaacuuuccu......................   1       0    S09

    ....................ugagguaguagguuguauaguu......................................................................   7       0    S08

    ....................ugagguaguagguuguauagu.......................................................................   2       0    S04
    ....................ugagguaguagguuguauaguu......................................................................   42      0    S04
    ....................ugagguaguagguuguauaguuu.....................................................................   2       0    S04
    ...................................................................cuauacagcuugcuaacuuucc.......................   7       0    S04
    ...................................................................cuauacagcuugcuaacuuuccu......................   2       0    S04

    ....................ugagguaguagguuguau..........................................................................   1       0    S02
    ....................ugagguaguagguuguauag........................................................................   1       0    S02
    ....................ugagguaguagguuguauagu.......................................................................   2       0    S02
    ....................ugagguaguagguuguauaguu......................................................................   31      0    S02
    ....................ugagguaguagguuguauaguuu.....................................................................   1       0    S02

    ....................ugagguaguagguuguauagu.......................................................................   2       0    S05
    ....................ugagguaguagguuguauaguu......................................................................   48      0    S05
    ....................ugagguaguagguuguauaguuu.....................................................................   1       0    S05
    ..........................................uagaacuacaccuuuaaugggcgaa.............................................   1       0    S05
    ...................................................................cuauacagcuugcuaacuuucc.......................   3       0    S05
```

```
                    Mature                                             Star
guuaugguauguuuucgaugugagguaguagguuguauaguuuagaacuacaccuuuaaugggcgaacuauacagcuugcuaacuuuccucgauacaugccgauugcaucuu

....................ugagguaguagguuguauaguu......................................................................	21	0	S10
....................ugagguaguagguuguauaguuu.....................................................................	2	0	S10
..........................................uagaacuacaccuuuaaugggcga..............................................	1	0	S10
...................................................................cuauacagcuugcuaacuuucc.......................	4	0	S10

....................ugagguaguagguuguauag........................................................................	1	0	S06
....................ugagguaguagguuguauaguu......................................................................	25	0	S06
....................ugagguaguagguuguauaguuu.....................................................................	1	0	S06
.....................gagguaguagguuguauaguu......................................................................	1	0	S06
...................................................................cuauacagcuugcuaacuuucc.......................	4	0	S06

....................ugagguaguagguuguaua.........................................................................	7	0	S01
....................ugagguaguagguuguauagu.......................................................................	3	0	S01
....................ugagguaguagguuguauaguu......................................................................	41	0	S01
....................ugagguaguagguuguauaguuu.....................................................................	1	0	S01
........................guaguagguuguauaguu......................................................................	1	0	S01

....................ugagguaguagguuguauagu.......................................................................	1	0	S07
....................ugagguaguagguuguauaguu......................................................................	18	0	S07
...................................................................cuauacagcuugcuaacuuucc.......................	1	0	S07
```

Provisional ID	:	Scaffold160_4618
Score total	:	591.4
Score for star read(s)	:	3.9
Score for read counts	:	583.9
Score for mfe	:	2
Score for randfold	:	1.6
Score for cons. seed	:	
Total read count	:	1157
Mature read count	:	806
Loop read count	:	2
Star read count	:	349

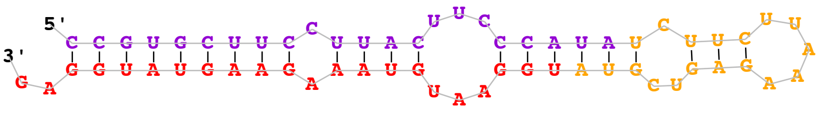

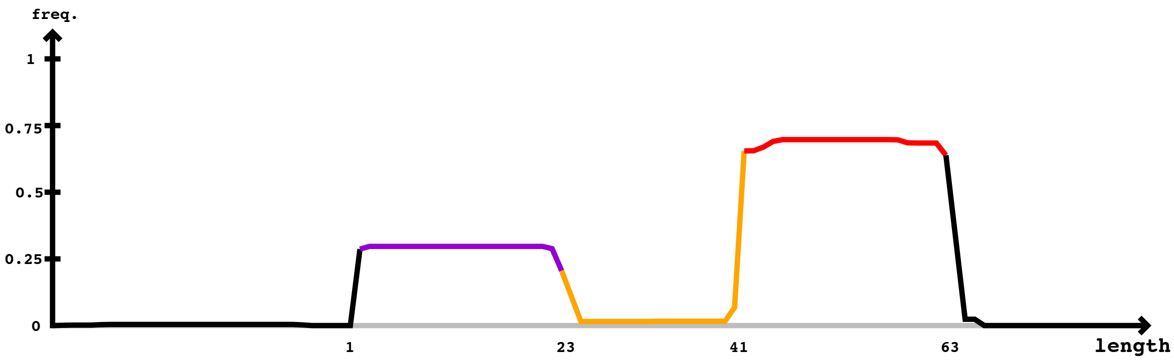

Star Mature

```
5'- uccagaagcuggaaguccucugucuggauuccgugcuuccuuacuucccauaucuucuuaaagagucguauggaauguaaagaaguauggaguguugacggagugauucauc   -3'   obs
    uccagaagcuggaaguccucugucuggauuccgugcuuccuuacuucccauaucuucuuaaagagucguauggaauguaaagaaguauggaguguugacggagugauucauc         exp
    (((((...))))).((((((((((.(.((((((((((((.((((...((((((.(((.....)))..))))))...)))).)))))))))))).).))))))).))).....   reads   mm   sample
    ..............................ccgugcuuccuuacuucccau.............................................................   1       0    S04
    ..............................ccgugcuuccuuacuucccaua............................................................   4       0    S04
    .....................................................................auggaauguaaagaaguaugga.....................   1       0    S04
    ......................................................................uggaauguaaagaaguauggag....................   11      0    S04
    ......................................................................uggaauguaaagaaguauggagu...................   1       0    S04

    ..............................ccgugcuuccuuacuucccau.............................................................   1       0    S02
    ......................................................................uggaauguaaagaaguauggagu...................   1       0    S02
    ........................................................................gaauguaaagaaguauggag....................   2       0    S02

    ..............................ccgugcuuccuuacuuccca..............................................................   2       0    S08
    ..............................ccgugcuuccuuacuucccau.............................................................   30      0    S08
    ..............................ccgugcuuccuuacuucccaua............................................................   65      0    S08
    .....................................................................auggaauguaaagaaguauggag....................   2       0    S08
    ......................................................................uggaauguaaagaaguauggag....................   28      0    S08
    ......................................................................uggaauguaaagaaguauggagu...................   4       0    S08
    ........................................................................gaauguaaagaaguauggag....................   2       0    S08
    .........................................................................aauguaaagaaguauggag....................   1       0    S08

    ..............................ccgugcuuccuuacuuccca..............................................................   3       0    S09
    ..............................ccgugcuuccuuacuucccau.............................................................   14      0    S09
    ..............................ccgugcuuccuuacuucccaua............................................................   34      0    S09
    ..............................ccgugcuuccuuacuucccauau...........................................................   1       0    S09
    ..............................ccgugcuuccuuacuucccauaucuucuuaaagagucgua..........................................   1       0    S09
    ...............................cgugcuuccuuacuucccau.............................................................   3       0    S09
    ...............................cgugcuuccuuacuucccaua............................................................   3       0    S09
    ...............................cgugcuuccuuacuucccauau...........................................................   3       0    S09
    ....................................................ucuucuuaaagagucgua..........................................   2       0    S09
    ....................................................ucuucuuaaagagucguauggaauguaaagaagua.........................   8       0    S09
    ....................................................ucuucuuaaagagucguauggaauguaaagaaguaugga.....................   1       0    S09
    .............................................................agagucguauggaauguaaagaaguauggagu...................   1       0    S09
    .....................................................................auggaauguaaagaaguaugga.....................   17      0    S09
    .....................................................................auggaauguaaagaaguauggag....................   7       0    S09
```

Star Mature

```
uccagaagcuggaaguccucugucuggauuccgugcuuccuuacuucccauaucuucuuaaagagucguauggaauguaaagaaguauggaguguugacggagugauucauc
......................................................................uggaauguaaagaaguaugga.....................	6	0	S09
......................................................................uggaauguaaagaaguauggag....................	312	0	S09
......................................................................uggaauguaaagaaguauggagu...................	23	0	S09
........................................................................gaauguaaagaaguauggagu...................	1	0	S09
........................................................................gaauguaaagaaguauggagugu.................	2	0	S09
.........................................................................aauguaaagaaguauggag....................	3	0	S09
.........................................................................aauguaaagaaguauggagugu.................	3	0	S09
..........................................................................auguaaagaaguauggag....................	4	0	S09

...aUaagcuggaaguccucugucu.......................................................................................	1	1	S03
..............................ccgugcuuccuuacuucccau.............................................................	3	0	S03
..............................ccgugcuuccuuacuucccaua............................................................	2	0	S03
..............................ccgugcuuccuuacuucccauau...........................................................	1	0	S03
.....................................................................auggaauguaaagaaguaugga.....................	1	0	S03
......................................................................uggaauguaaagaaguauggag....................	1	0	S03

....Uaagcuggaaguccucugucu.......................................................................................	1	1	S07
..............................ccgugcuuccuuacuucccau.............................................................	12	0	S07
..............................ccgugcuuccuuacuucccaua............................................................	36	0	S07
...............................cgugcuuccuuacuucccaua............................................................	1	0	S07
...............................cgugcuuccuuacuucccauau...........................................................	1	0	S07
.....................................................................auggaauguaaagaaguaugga.....................	1	0	S07
.....................................................................auggaauguaaagaaguauggag....................	3	0	S07
......................................................................uggaauguaaagaaguauggag....................	20	0	S07
......................................................................uggaauguaaagaaguauggagu...................	4	0	S07

uccaUaagcuggaaguccucuguc........................................................................................	2	1	S06
..............................ccgugcuuccuuacuucccau.............................................................	1	0	S06
......................................................................uggaauguaaagaaguaugga.....................	1	0	S06
......................................................................uggaauguaaagaaguauggag....................	7	0	S06
......................................................................uggaauguaaagaaguauggagu...................	1	0	S06
........................................................................gaauguaaagaaguauggag....................	1	0	S06

..............................ccgugcuuccuuacuucccaua............................................................	1	0	S01
.....................................................................auggaauguaaagaaguaugga.....................	2	0	S01
......................................................................uggaauguaaagaaguaugga.....................	2	0	S01
......................................................................uggaauguaaagaaguauggag....................	3	0	S01
......................................................................uggaauguaaagaaguauggagu...................	2	0	S01

...aUaagcuggaaguccucugucu.......................................................................................	1	1	S10
..............................ccgugcuuccuuacuuccca..............................................................	5	0	S10
..............................ccgugcuuccuuacuucccau.............................................................	31	0	S10
..............................ccgugcuuccuuacuucccaua............................................................	88	0	S10
...............................cgugcuuccuuacuucccaua............................................................	1	0	S10
................................................ucuucuuaaagagucguauggaauguaaagaagu..............................	1	0	S10
................................................ucuucuuaaagagucguauggaauguaaagaagua.............................	4	0	S10
................................................ucuucuuaaagagucguauggaauguaaagaaguau............................	1	0	S10
.....................................................................auggaauguaaagaagua.........................	1	0	S10
.....................................................................auggaauguaaagaaguaugga.....................	17	0	S10
.....................................................................auggaauguaaagaaguauggag....................	9	0	S10
......................................................................uggaauguaaagaaguaugga.....................	5	0	S10
......................................................................uggaauguaaagaaguauggag....................	255	0	S10
......................................................................uggaauguaaagaaguauggagu...................	6	0	S10
.......................................................................ggaauguaaagaaguauggagu...................	1	0	S10
........................................................................gaauguaaagaaguauggagu...................	1	0	S10
........................................................................gaauguaaagaaguauggagugu.................	7	0	S10
.........................................................................aauguaaagaaguauggag....................	2	0	S10
.........................................................................aauguaaagaaguauggagugu.................	16	0	S10
..........................................................................auguaaagaaguauggag....................	4	0	S10

..............................ccgugcuuccuuacuucccau.............................................................	2	0	S05
......................................................................uggaauguaaagaaguauggag....................	2	0	S05
```

Provisional ID	: Scaffold300_7829
Score total	: 63960.9
Score for star read(s)	: 3.9
Score for read counts	: 63954.1
Score for mfe	: 1.3
Score for randfold	: 1.6
Score for cons. seed	:
Total read count	: 125455
Mature read count	: 123153
Loop read count	: 0
Star read count	: 2302

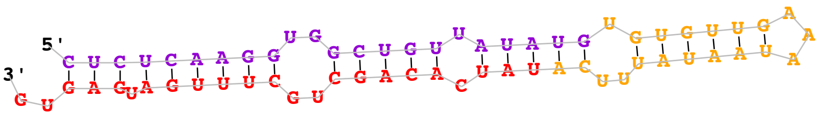

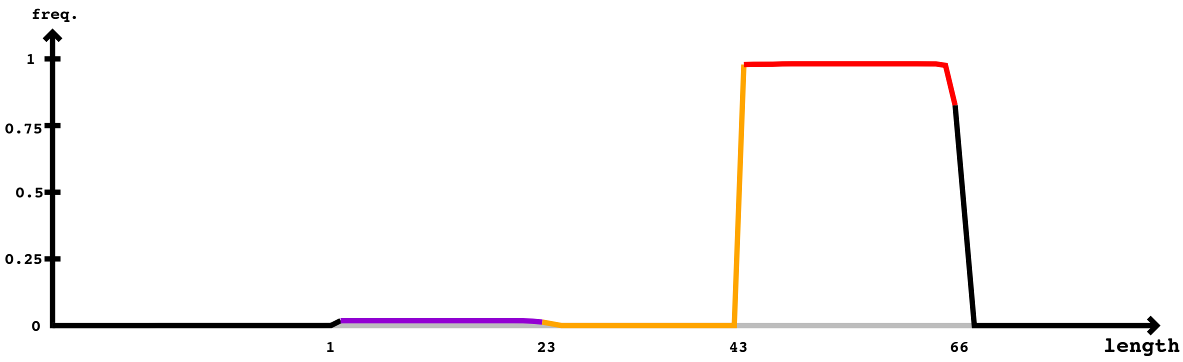


```
                                       Star                                    Mature
5'- aagaagaugaugugugcguguguccccucucucaagguggcuguuauauguguguugaaauaauauuucauaucacagcugcuuugaugagugugaaugcauugaaguauuca   -3'    obs
    aagaagaugaugugugcguguguccccucucucaagguggcuguuauauguguguugaaauaauauuucauaucacagcugcuuugaugagugugaaugcauugaaguauuca          exp
    ..(((.((.....((((((...((.(..(((((((((..(((((.(((((.((((((...))))))..))))).)))))..)))))).))).).))))))))....)).))).     reads      mm         sample
    ..gaagaugaugugugcguguguccccu.....................................................................................     2          0          S04
...........................ucucucaagguggcuguuauau................................................................     1          0          S04
    ............................cucucaagguggcuguuau..................................................................     1          0          S04
    ............................cucucaagguggcuguuaua.................................................................     3          0          S04
    ............................cucucaagguggcuguuauau................................................................     19         0          S04
    ............................cucucaagguggcuguuauaug...............................................................     18         0          S04
    ............................cucucaagguggcuguuauaugu..............................................................     1          0          S04
    ......................................................................uaucacagcugcuuugaugag......................     9          0          S04
    ......................................................................uaucacagcugcuuugaugagu.....................     155        0          S04
    ......................................................................uaucacagcugcuuugaugagug....................     4120       0          S04
    ......................................................................uaucacagcugcuuugaugagugu...................     15         0          S04
    .......................................................................aucacagcugcuuugaugagug....................     3          0          S04
    ..........................................................................acagcugcuuugaugagug....................     13         0          S04
    ...........................................................................cagcugcuuugaugagug....................     4          0          S04

    ............................cucucaagguggcuguuaua.................................................................     1          0          S02
    ............................cucucaagguggcuguuauau................................................................     5          0          S02
    ............................cucucaagguggcuguuauaug...............................................................     1          0          S02
    ...............................ucaagguggcuguuauaug...............................................................     1          0          S02
    ......................................................................uaucacagcugcuuugaug........................     1          0          S02
    ......................................................................uaucacagcugcuuugauga.......................     1          0          S02
    ......................................................................uaucacagcugcuuugaugagu.....................     32         0          S02
    ......................................................................uaucacagcugcuuugaugagug....................     889        0          S02
    ......................................................................uaucacagcugcuuugaugagugu...................     5          0          S02
    .......................................................................aucacagcugcuuugaugagug....................     1          0          S02
    ..........................................................................acagcugcuuugaugagug....................     4          0          S02

    ..........ugugugcguguguccccu.....................................................................................     2          0          S08
    ............................cucucaagguggcuguua...................................................................     1          0          S08
    ............................cucucaagguggcuguuau..................................................................     7          0          S08
    ............................cucucaagguggcuguuaua.................................................................     31         0          S08
    ............................cucucaagguggcuguuauau................................................................     48         0          S08
    ............................cucucaagguggcuguuauaug...............................................................     106        0          S08
```

```
                                              Star                                        Mature
aagaagaugaugugugcguguguccccucucucaagguggcuguuauauguguguugaaauaauauuucauaucacagcugcuuugaugagugugaaugcauugaaguauuca
............................cucucaagguggcuguuauauguguguugaa......................................................    1     0     S08
............................cucucaagguggcuguuauauguguguugaaauaauauuu.............................................    1     0     S08
............................cucucaagguggcuguuauauguguguugaaauaauauuuca...........................................    2     0     S08
.............................ucucaagguggcuguuauaugu..............................................................    1     0     S08
.............................ucucaagguggcuguuauaugug.............................................................    1     0     S08
......................................................................uaucacagcugcuuugaug........................    1     0     S08
......................................................................uaucacagcugcuuugauga.......................    2     0     S08
......................................................................uaucacagcugcuuugaugag......................    46    0     S08
......................................................................uaucacagcugcuuugaugagu.....................    700   0     S08
......................................................................uaucacagcugcuuugaugagug....................    8985  0     S08
......................................................................uaucacagcugcuuugaugagugu...................    67    0     S08
......................................................................uaucacagcugcuuugaugagugug..................    1     0     S08
.......................................................................aucacagcugcuuugaugagug....................    22    0     S08
.......................................................................aucacagcugcuuugaugagugu...................    3     0     S08
........................................................................ucacagcugcuuugaugagu.....................    1     0     S08
........................................................................ucacagcugcuuugaugagug....................    4     0     S08
.........................................................................cacagcugcuuugaugagug....................    6     0     S08
..........................................................................acagcugcuuugaugagug....................    23    0     S08
...........................................................................cagcugcuuugaugagug....................    13    0     S08

......augaugugugcguguguccccu.....................................................................................    1     0     S09
...........................ucucucaagguggcuguuauau................................................................    1     0     S09
...........................ucucucaagguggcuguuauaug...............................................................    3     0     S09
...........................ucucucaagguggcuguuauaugu..............................................................    1     0     S09
............................cucucaagguggcuguua...................................................................    1     0     S09
............................cucucaagguggcuguuau..................................................................    1     0     S09
............................cucucaagguggcuguuaua.................................................................    92    0     S09
............................cucucaagguggcuguuauau................................................................    96    0     S09
............................cucucaagguggcuguuauaug...............................................................    684   0     S09
............................cucucaagguggcuguuauaugu..............................................................    4     0     S09
............................cucucaagguggcuguuauaugugug...........................................................    1     0     S09
............................cucucaagguggcuguuauauguguguugaaauaauauuuc............................................    1     0     S09
............................cucucaagguggcuguuauauguguguugaaauaauauuuca...........................................    2     0     S09
.............................ucucaagguggcuguuauaug...............................................................    3     0     S09
...............................ucaagguggcuguuauauguguguugaaauaaua................................................    1     0     S09
....................................................................cauaucacagcugcuuugaug........................    1     0     S09
.....................................................................auaucacagcugcuuugaugagug....................    3     0     S09
......................................................................uaucacagcugcuuugau.........................    1     0     S09
......................................................................uaucacagcugcuuugaug........................    5     0     S09
......................................................................uaucacagcugcuuugauga.......................    7     0     S09
......................................................................uaucacagcugcuuugaugag......................    215   0     S09
......................................................................uaucacagcugcuuugaugagu.....................    8416  0     S09
......................................................................uaucacagcugcuuugaugagug....................    32707 0     S09
......................................................................uaucacagcugcuuugaugagugu...................    461   0     S09
......................................................................uaucacagcugcuuugaugagugug..................    1     0     S09
.......................................................................aucacagcugcuuugaugagu.....................    7     0     S09
.......................................................................aucacagcugcuuugaugagug....................    13    0     S09
.......................................................................aucacagcugcuuugaugagugu...................    3     0     S09
........................................................................ucacagcugcuuugaugagu.....................    2     0     S09
........................................................................ucacagcugcuuugaugagug....................    1     0     S09
.........................................................................cacagcugcuuugaugagu.....................    1     0     S09
.........................................................................cacagcugcuuugaugagug....................    2     0     S09
..........................................................................acagcugcuuugaugagu.....................    12    0     S09
..........................................................................acagcugcuuugaugagug....................    14    0     S09
...........................................................................cagcugcuuugaugagug....................    4     0     S09

...........................ucucucaagguggcuguuauaug...............................................................    1     0     S03
............................cucucaagguggcuguua...................................................................    1     0     S03
............................cucucaagguggcuguuau..................................................................    1     0     S03
............................cucucaagguggcuguuaua.................................................................    4     0     S03
............................cucucaagguggcuguuauau................................................................    12    0     S03
............................cucucaagguggcuguuauaug...............................................................    20    0     S03
............................cucucaagguggcuguuauaugug.............................................................    1     0     S03
....................................................................cauaucacagcugcuuugaugagu.....................    1     0     S03
......................................................................uaucacagcugcuuugauga.......................    2     0     S03
......................................................................uaucacagcugcuuugaugag......................    17    0     S03
......................................................................uaucacagcugcuuugaugagu.....................    102   0     S03
......................................................................uaucacagcugcuuugaugagug....................    2823  0     S03
......................................................................uaucacagcugcuuugaugagugu...................    11    0     S03
.......................................................................aucacagcugcuuugaugag......................    1     0     S03
```

```
                            Star                                       Mature
aagaagaugaugugugcgugugucccccucucucaagguggcuguuauauguguguugaaauaaauauuucauaucacagcugcuuugaugagugugaaugcauugaaguauuca
.........................................................................aucacagcugcuuugaugagug....................    2       0    S03
..........................................................................ucacagcugcuuugaugagug....................    1       0    S03
............................................................................acagcugcuuugaugagug....................    7       0    S03
.............................................................................cagcugcuuugaugagug....................    2       0    S03

.....gaugaugugugcgugugucccccu......................................................................................    1       0    S07
.........augugugcgugugucccccu......................................................................................    2       0    S07
............................ucucucaagguggcuguuaua..................................................................    1       0    S07
.............................cucucaagguggcuguuau...................................................................    2       0    S07
.............................cucucaagguggcuguuaua..................................................................    34      0    S07
.............................cucucaagguggcuguuauau.................................................................    44      0    S07
.............................cucucaagguggcuguuauaug................................................................    371     0    S07
.............................cucucaagguggcuguuauaugu...............................................................    2       0    S07
..............................ucucaagguggcuguuauau.................................................................    1       0    S07
........................................................................uaucacagcugcuuugaug........................    3       0    S07
........................................................................uaucacagcugcuuugauga.......................    3       0    S07
........................................................................uaucacagcugcuuugaugag......................    119     0    S07
........................................................................uaucacagcugcuuugaugagu.....................    2444    0    S07
........................................................................uaucacagcugcuuugaugagug....................    12485   0    S07
........................................................................uaucacagcugcuuugaugagugu...................    135     0    S07
.........................................................................aucacagcugcuuugaugagug....................    7       0    S07
.........................................................................aucacagcugcuuugaugagugu...................    4       0    S07
..........................................................................ucacagcugcuuugaugagu.....................    2       0    S07
...........................................................................cacagcugcuuugaugagu.....................    1       0    S07
...........................................................................cacagcugcuuugaugagug....................    1       0    S07
............................................................................acagcugcuuugaugagu.....................    4       0    S07
............................................................................acagcugcuuugaugagug....................    13      0    S07
.............................................................................cagcugcuuugaugagug....................    6       0    S07

........................................................................uaucacagcugcuuugaugag......................    8       0    S01
........................................................................uaucacagcugcuuugaugagu.....................    25      0    S01
........................................................................uaucacagcugcuuugaugagug....................    599     0    S01
.........................................................................aucacagcugcuuugaugagug....................    3       0    S01
............................................................................acagcugcuuugaugagug....................    1       0    S01

.........augugugcgugugucccccu......................................................................................    1       0    S06
............................ucucucaagguggcuguuauau.................................................................    1       0    S06
.............................cucucaagguggcuguua....................................................................    1       0    S06
.............................cucucaagguggcuguuau...................................................................    2       0    S06
.............................cucucaagguggcuguuaua..................................................................    3       0    S06
.............................cucucaagguggcuguuauau.................................................................    22      0    S06
.............................cucucaagguggcuguuauaug................................................................    29      0    S06
........................................................................uaucacagcugcuuugaug........................    2       0    S06
........................................................................uaucacagcugcuuugaugag......................    20      0    S06
........................................................................uaucacagcugcuuugaugagu.....................    140     0    S06
........................................................................uaucacagcugcuuugaugagug....................    5509    0    S06
........................................................................uaucacagcugcuuugaugagugu...................    35      0    S06
.........................................................................aucacagcugcuuugaugagug....................    30      0    S06
...........................................................................cacagcugcuuugaugagug....................    2       0    S06
............................................................................acagcugcuuugaugagug....................    19      0    S06
.............................................................................cagcugcuuugaugagug....................    5       0    S06

............................ucucucaagguggcuguuauaug................................................................    2       0    S10
.............................cucucaagguggcuguua....................................................................    4       0    S10
.............................cucucaagguggcuguuau...................................................................    9       0    S10
.............................cucucaagguggcuguuaua..................................................................    53      0    S10
.............................cucucaagguggcuguuauau.................................................................    132     0    S10
.............................cucucaagguggcuguuauaug................................................................    374     0    S10
.............................cucucaagguggcuguuauaugu...............................................................    1       0    S10
.............................cucucaagguggcuguuauauguguguugaaauaaauauuuc............................................    1       0    S10
..............................ucucaagguggcuguuauaug................................................................    1       0    S10
..............................ucucaagguggcuguuauaugu...............................................................    1       0    S10
........................................................................uaucacagcugcuuugau.........................    1       0    S10
........................................................................uaucacagcugcuuugaug........................    7       0    S10
........................................................................uaucacagcugcuuugauga.......................    11      0    S10
........................................................................uaucacagcugcuuugaugag......................    236     0    S10
........................................................................uaucacagcugcuuugaugagu.....................    6587    0    S10
........................................................................uaucacagcugcuuugaugagug....................    29979   0    S10
........................................................................uaucacagcugcuuugaugagugu...................    410     0    S10
.........................................................................aucacagcugcuuugaugagu.....................    1       0    S10
```

```
                                                  Star                                      Mature
aagaagaugaugugugcguguguccccucucucaagguggcuguuauauguguguugaaauaauauuucauaucacagcugcuuugaugagugugaaugcauugaaguauuca
.......................................................................aucacagcugcuuugaugagug....................   10     0   S10
.......................................................................aucacagcugcuuugaugagugu...................   4      0   S10
........................................................................ucacagcugcuuugaugagu.....................   1      0   S10
.........................................................................cacagcugcuuugaugagug....................   1      0   S10
..........................................................................acagcugcuuugaugagu.....................   11     0   S10
..........................................................................acagcugcuuugaugagug....................   20     0   S10
..........................................................................acagcugcuuugaugagugu...................   1      0   S10
...........................................................................cagcugcuuugaugagug....................   3      0   S10

............................cucucaagguggcuguuau..................................................................   1      0   S05
............................cucucaagguggcuguuaua.................................................................   5      0   S05
............................cucucaagguggcuguuauau................................................................   16     0   S05
............................cucucaagguggcuguuauaug...............................................................   17     0   S05
......................................................................uaucacagcugcuuugaug........................   1      0   S05
......................................................................uaucacagcugcuuugauga.......................   1      0   S05
......................................................................uaucacagcugcuuugaugag......................   18     0   S05
......................................................................uaucacagcugcuuugaugagu.....................   154    0   S05
......................................................................uaucacagcugcuuugaugagug....................   4039   0   S05
......................................................................uaucacagcugcuuugaugagugu...................   28     0   S05
.......................................................................aucacagcugcuuugaugagu.....................   2      0   S05
.......................................................................aucacagcugcuuugaugagug....................   7      0   S05
........................................................................ucacagcugcuuugaugagug....................   1      0   S05
..........................................................................acagcugcuuugaugagug....................   21     0   S05
...........................................................................cagcugcuuugaugagug....................   2      0   S05
```

Provisional ID	:	Scaffold300_7831
Score total	:	37225.1
Score for star read(s)	:	3.9
Score for read counts	:	37217.9
Score for mfe	:	1.7
Score for randfold	:	1.6
Score for cons. seed	:	
Total read count	:	73013
Mature read count	:	49147
Loop read count	:	6
Star read count	:	23860

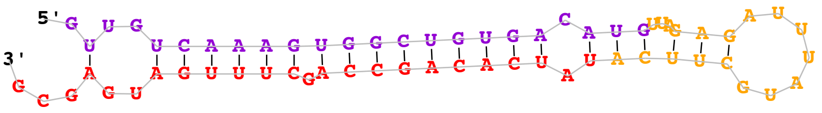

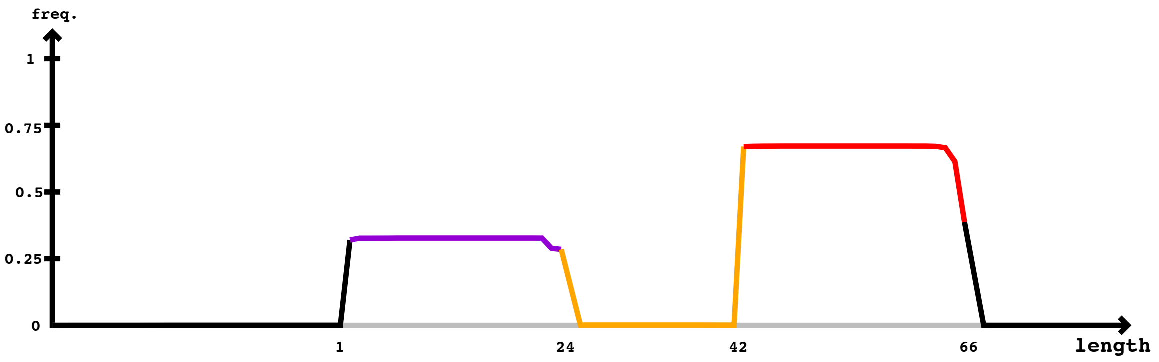

Star **Mature**

```
5'- aucaagaagaaguugugguuguguuucugguugucaaaguggcugugacauguuagagauuuaugcuucauaucacagccagcuuugaugagcggaacuucaacuggaga  -3'  obs
    aucaagaagaaguugugguuguguuucugguugucaaaguggcugugacauguuagagauuuaugcuucauaucacagccagcuuugaugagcggaacuucaacuggaga       exp
    ............((.((((((...(((((.(..(((((((((((((((.(((...(((.......)))))).))))))))).))))))..).)))))...)))))).)).  reads  mm  sample
    ..........aguugugguuguguuucug.................................................................................  1      0   S04
    .............................guugucaaaguggcuguga..............................................................  1      0   S04
    .............................guugucaaaguggcugugaca............................................................  102    0   S04
    .............................guugucaaaguggcugugacau...........................................................  16     0   S04
    .............................guugucaaaguggcugugacaug..........................................................  1457   0   S04
    .............................guugucaaaguggcugugacaugu.........................................................  1      0   S04
    .............................guugucaaaguggcugugacauguuagagauuuaugcu...........................................  1      0   S04
    .............................guugucaaaguggcugugacauguuagagauuuaugcuuca........................................  2      0   S04
    ..............................uugucaaaguggcugugaca............................................................  2      0   S04
    ..............................uugucaaaguggcugugacaug..........................................................  11     0   S04
    ..................................caaaguggcugugacaug..........................................................  1      0   S04
    .....................................................................auaucacagccagcuuugaugag..................  1      0   S04
    ......................................................................uaucacagccagcuuugaug....................  4      0   S04
    ......................................................................uaucacagccagcuuugauga...................  4      0   S04
    ......................................................................uaucacagccagcuuugaugag..................  47     0   S04
    ......................................................................uaucacagccagcuuugaugagc.................  157    0   S04
    ......................................................................uaucacagccagcuuugaugagcg................  471    0   S04
    .......................................................................aucacagccagcuuugaugagcg................  2      0   S04
    ........................................................................ucacagccagcuuugauga...................  1      0   S04

    .............................guugucaaaguggcugugaca............................................................  15     0   S02
    .............................guugucaaaguggcugugacau...........................................................  7      0   S02
    .............................guugucaaaguggcugugacaug..........................................................  318    0   S02
    ..............................uugucaaaguggcugugacaug..........................................................  7      0   S02
    ..................................caaaguggcugugacaug..........................................................  1      0   S02
    ......................................................................uaucacagccagcuuugaug....................  5      0   S02
    ......................................................................uaucacagccagcuuugauga...................  2      0   S02
    ......................................................................uaucacagccagcuuugaugag..................  14     0   S02
    ......................................................................uaucacagccagcuuugaugagc.................  24     0   S02
    ......................................................................uaucacagccagcuuugaugagcg................  94     0   S02
    .......................................................................aucacagccagcuuugaugagcg................  1      0   S02
    ........................................................................ucacagccagcuuugaugag..................  2      0   S02
    ..........................................................................acagccagcuuugaugagcg................  1      0   S02
```

Star **Mature**

```
aucaagaagaaguugugguuguguuucugguugucaaaguggcugugacauguuagagauuuaugcuucauaucacagccagcuuugaugagcggaacuucaacuggaga

aucaagaagaaguugugguuguguuucug.................................................................................  2     0  S08
...aagaagaaguugugguuguguuucug.................................................................................  1     0  S08
.......agaaguugugguuguguuucug.................................................................................  1     0  S08
........gaaguugugguuguguuucug.................................................................................  1     0  S08
.........aaguugugguuguguuucug.................................................................................  2     0  S08
...........guugugguuguguuucug.................................................................................  2     0  S08
.............................guugucaaaguggcugugaca............................................................  81    0  S08
.............................guugucaaaguggcugugacau...........................................................  12    0  S08
.............................guugucaaaguggcugugacaug..........................................................  570   0  S08
.............................guugucaaaguggcugugacauguuagagauuuaugcuuca........................................  1     0  S08
..............................uugucaaaguggcugugaca............................................................  13    0  S08
..............................uugucaaaguggcugugacaug..........................................................  47    0  S08
................................gucaaaguggcugugaca............................................................  1     0  S08
.................................ucaaaguggcugugacaug..........................................................  6     0  S08
..................................caaaguggcugugacaug..........................................................  4     0  S08
..................................caaaguggcugugacaugu.........................................................  1     0  S08
...................................aaaguggcugugacauguuagagauuuaugcuu..........................................  2     0  S08
......................................................................uaucacagccagcuuugaug....................  2     0  S08
......................................................................uaucacagccagcuuugauga...................  16    0  S08
......................................................................uaucacagccagcuuugaugag..................  177   0  S08
......................................................................uaucacagccagcuuugaugagc.................  652   0  S08
......................................................................uaucacagccagcuuugaugagcg................  2025  0  S08
......................................................................uaucacagccagcuuugaugagcgg...............  2     0  S08
......................................................................uaucacagccagcuuugaugagcgga..............  1     0  S08
.......................................................................aucacagccagcuuugaugagc.................  4     0  S08
.......................................................................aucacagccagcuuugaugagcg................  5     0  S08
........................................................................ucacagccagcuuugaug....................  1     0  S08

.............................guugucaaaguggcugugaca............................................................  96    0  S03
.............................guugucaaaguggcugugacau...........................................................  12    0  S03
.............................guugucaaaguggcugugacaug..........................................................  953   0  S03
..............................uugucaaaguggcugugaca............................................................  1     0  S03
..............................uugucaaaguggcugugacaug..........................................................  9     0  S03
................................gucaaaguggcugugacaug..........................................................  1     0  S03
....................................................uuagagauuuaugcuuca........................................  1     0  S03
.....................................................................auaucacagccagcuuugaugagc.................  1     0  S03
......................................................................uaucacagccagcuuuga......................  3     0  S03
......................................................................uaucacagccagcuuugauga...................  4     0  S03
......................................................................uaucacagccagcuuugaugag..................  22    0  S03
......................................................................uaucacagccagcuuugaugagc.................  114   0  S03
......................................................................uaucacagccagcuuugaugagcg................  352   0  S03
......................................................................uaucacagccagcuuugaugagcgg...............  1     0  S03
......................................................................uaucacagccagcuuugaugagcgga..............  1     0  S03
.......................................................................aucacagccagcuuugaugagc.................  1     0  S03
.......................................................................aucacagccagcuuugaugagcg................  1     0  S03

aucaagaagaaguugugguuguguuucug.................................................................................  2     0  S09
...aagaagaaguugugguuguguuucug.................................................................................  1     0  S09
......aagaaguugugguuguguuucu..................................................................................  1     0  S09
.........aaguugugguuguguuucug.................................................................................  1     0  S09
..........aguugugguuguguuucug.................................................................................  1     0  S09
.............................guugucaaaguggcugugac.............................................................  6     0  S09
.............................guugucaaaguggcugugaca............................................................  806   0  S09
.............................guugucaaaguggcugugacau...........................................................  59    0  S09
.............................guugucaaaguggcugugacaug..........................................................  4437  0  S09
.............................guugucaaaguggcugugacaugu.........................................................  11    0  S09
.............................guugucaaaguggcugugacauguuaga.....................................................  1     0  S09
.............................guugucaaaguggcugugacauguuagagauuuaugc............................................  1     0  S09
.............................guugucaaaguggcugugacauguuagagauuuaugcuuc.........................................  3     0  S09
.............................guugucaaaguggcugugacauguuagagauuuaugcuuca........................................  22    0  S09
..............................uugucaaaguggcugugaca............................................................  18    0  S09
..............................uugucaaaguggcugugacaug..........................................................  84    0  S09
................................gucaaaguggcugugaca............................................................  1     0  S09
..................................caaaguggcugugacaug..........................................................  2     0  S09
....................................................uuagagauuuaugcuuca........................................  1     0  S09
.....................................................................auaucacagccagcuuugaugag..................  1     0  S09
.....................................................................auaucacagccagcuuugaugagc.................  4     0  S09
......................................................................uaucacagccagcuuugaug....................  21    0  S09
......................................................................uaucacagccagcuuugauga...................  168   0  S09
```

```
                             Star                                      Mature
aucaagaagaaguugugguuguguuucugguugucaaaguggcugugacauguuagagauuuaugcuucauaucacagccagcuuugaugagcggaacuucaacuggaga
......................................................................uaucacagccagcuuugaugag..................  1559   0  S09
......................................................................uaucacagccagcuuugaugagc.................  7802   0  S09
......................................................................uaucacagccagcuuugaugagcg................  10721  0  S09
......................................................................uaucacagccagcuuugaugagcgg...............  7      0  S09
......................................................................uaucacagccagcuuugaugagcgga..............  3      0  S09
.......................................................................aucacagccagcuuugaugagc.................  21     0  S09
.......................................................................aucacagccagcuuugaugagcg................  1      0  S09
........................................................................ucacagccagcuuugaugagc.................  9      0  S09
........................................................................ucacagccagcuuugaugagcg................  2      0  S09
.........................................................................cacagccagcuuugaugag..................  1      0  S09
.........................................................................cacagccagcuuugaugagc.................  1      0  S09
..........................................................................acagccagcuuugaugag..................  3      0  S09
..........................................................................acagccagcuuugaugagcg................  3      0  S09

.........aaguugugguuguguuucug.................................................................................  1      0  S07
.............................guugucaaaguggcugugac.............................................................  3      0  S07
.............................guugucaaaguggcugugaca............................................................  433    0  S07
.............................guugucaaaguggcugugacau...........................................................  26     0  S07
.............................guugucaaaguggcugugacaug..........................................................  4216   0  S07
.............................guugucaaaguggcugugacaugu.........................................................  14     0  S07
.............................guugucaaaguggcugugacauguuagagauuuaugcu...........................................  1      0  S07
.............................guugucaaaguggcugugacauguuagagauuuaugcuuc.........................................  1      0  S07
.............................guugucaaaguggcugugacauguuagagauuuaugcuuca........................................  14     0  S07
..............................uugucaaaguggcugugaca............................................................  12     0  S07
..............................uugucaaaguggcugugacaug..........................................................  79     0  S07
..............................uugucaaaguggcugugacaugu.........................................................  2      0  S07
...............................ugucaaaguggcugugaca............................................................  1      0  S07
................................gucaaaguggcugugaca............................................................  1      0  S07
.................................ucaaaguggcugugacaug..........................................................  1      0  S07
..................................caaaguggcugugacaug..........................................................  2      0  S07
..................................caaaguggcugugacaugu.........................................................  1      0  S07
.....................................................................auaucacagccagcuuugaugag..................  1      0  S07
.....................................................................auaucacagccagcuuugaugagc.................  1      0  S07
......................................................................uaucacagccagcuuuga......................  1      0  S07
......................................................................uaucacagccagcuuugau.....................  1      0  S07
......................................................................uaucacagccagcuuugaug....................  10     0  S07
......................................................................uaucacagccagcuuugauga...................  36     0  S07
......................................................................uaucacagccagcuuugaugag..................  372    0  S07
......................................................................uaucacagccagcuuugaugagc.................  1165   0  S07
......................................................................uaucacagccagcuuugaugagcg................  3181   0  S07
......................................................................uaucacagccagcuuugaugagcgg...............  4      0  S07
.......................................................................aucacagccagcuuugaugag..................  1      0  S07
.......................................................................aucacagccagcuuugaugagc.................  2      0  S07
.......................................................................aucacagccagcuuugaugagcg................  3      0  S07
..........................................................................acagccagcuuugaugagcg................  1      0  S07

.............................guugucaaaguggcuguga..............................................................  1      0  S06
.............................guugucaaaguggcugugac.............................................................  2      0  S06
.............................guugucaaaguggcugugaca............................................................  247    0  S06
.............................guugucaaaguggcugugacau...........................................................  26     0  S06
.............................guugucaaaguggcugugacaug..........................................................  1938   0  S06
.............................guugucaaaguggcugugacaugu.........................................................  4      0  S06
.............................guugucaaaguggcugugacauguuagagauuuaugcuu..........................................  2      0  S06
.............................guugucaaaguggcugugacauguuagagauuuaugcuuc.........................................  1      0  S06
.............................guugucaaaguggcugugacauguuagagauuuaugcuuca........................................  1      0  S06
..............................uugucaaaguggcugugaca............................................................  8      0  S06
..............................uugucaaaguggcugugacaug..........................................................  38     0  S06
...............................ugucaaaguggcugugaca............................................................  1      0  S06
...............................ugucaaaguggcugugacaug..........................................................  1      0  S06
................................gucaaaguggcugugacaug..........................................................  1      0  S06
..................................caaaguggcugugacaug..........................................................  3      0  S06
................................................cauguuagagauuuaugcuuc.........................................  1      0  S06
......................................................................uaucacagccagcuuugaug....................  1      0  S06
......................................................................uaucacagccagcuuugauga...................  3      0  S06
......................................................................uaucacagccagcuuugaugag..................  60     0  S06
......................................................................uaucacagccagcuuugaugagc.................  215    0  S06
......................................................................uaucacagccagcuuugaugagcg................  512    0  S06
.......................................................................aucacagccagcuuugaugagcg................  2      0  S06

.............................guugucaaaguggcuguga..............................................................  1      0  S01
```

Star **Mature**

```
aucaagaagaaguugugguuguguuucugguugucaaaguggcugugacauguuagagauuuaugcuucauaucacagccagcuuugaugagcggaacuucaacuggaga
.............................guugucaaaguggcugugaca............................................................   1      0    S01
.............................guugucaaaguggcugugacau...........................................................   3      0    S01
.............................guugucaaaguggcugugacaug..........................................................   63     0    S01
.............................guugucaaaguggcugugacaugu.........................................................   2      0    S01
..............................uugucaaaguggcugugacaug..........................................................   2      0    S01
...............................................acauguuagagauuuaugcuu..........................................   1      0    S01
......................................................................uaucacagccagcuuugaug....................   1      0    S01
......................................................................uaucacagccagcuuugaugag..................   5      0    S01
......................................................................uaucacagccagcuuugaugagc.................   14     0    S01
......................................................................uaucacagccagcuuugaugagcg................   69     0    S01
.......................................................................aucacagccagcuuugaugagc.................   1      0    S01

.ucaagaagaaguugugguuguguuucug.................................................................................   1      0    S10
........gaaguugugguuguguuucug.................................................................................   1      0    S10
.........aaguugugguuguguuucug.................................................................................   2      0    S10
.............................guugucaaaguggcugugac.............................................................   5      0    S10
.............................guugucaaaguggcugugaca............................................................   813    0    S10
.............................guugucaaaguggcugugacau...........................................................   77     0    S10
.............................guugucaaaguggcugugacaug..........................................................   5188   0    S10
.............................guugucaaaguggcugugacaugu.........................................................   11     0    S10
.............................guugucaaaguggcugugacauguuagagauu.................................................   1      0    S10
.............................guugucaaaguggcugugacauguuagagauuuaugcu...........................................   1      0    S10
.............................guugucaaaguggcugugacauguuagagauuuaugcuu..........................................   3      0    S10
.............................guugucaaaguggcugugacauguuagagauuuaugcuuc.........................................   2      0    S10
.............................guugucaaaguggcugugacauguuagagauuuaugcuuca........................................   9      0    S10
..............................uugucaaaguggcugugac.............................................................   2      0    S10
..............................uugucaaaguggcugugaca............................................................   31     0    S10
..............................uugucaaaguggcugugacaug..........................................................   92     0    S10
...............................ugucaaaguggcugugaca............................................................   2      0    S10
................................gucaaaguggcugugaca............................................................   1      0    S10
................................gucaaaguggcugugacaug..........................................................   1      0    S10
..................................caaaguggcugugacaug..........................................................   4      0    S10
....................................................uuagagauuuaugcuuca........................................   4      0    S10
.....................................................................auaucacagccagcuuugaugagc.................   1      0    S10
......................................................................uaucacagccagcuuuga......................   1      0    S10
......................................................................uaucacagccagcuuugau.....................   1      0    S10
......................................................................uaucacagccagcuuugaug....................   17     0    S10
......................................................................uaucacagccagcuuugauga...................   154    0    S10
......................................................................uaucacagccagcuuugaugag..................   1455   0    S10
......................................................................uaucacagccagcuuugaugagc.................   6203   0    S10
......................................................................uaucacagccagcuuugaugagcg................   10360  0    S10
......................................................................uaucacagccagcuuugaugagcgg...............   4      0    S10
......................................................................uaucacagccagcuuugaugagcgga..............   2      0    S10
.......................................................................aucacagccagcuuugaugag..................   3      0    S10
.......................................................................aucacagccagcuuugaugagc.................   23     0    S10
.......................................................................aucacagccagcuuugaugagcg................   1      0    S10
........................................................................ucacagccagcuuugaugagc.................   12     0    S10
........................................................................ucacagccagcuuugaugagcg................   7      0    S10
.........................................................................cacagccagcuuugaugagcg................   2      0    S10
..........................................................................acagccagcuuugaugag..................   4      0    S10
..........................................................................acagccagcuuugaugagc.................   2      0    S10
..........................................................................acagccagcuuugaugagcg................   1      0    S10

...aagaagaaguugugguuguguuucug.................................................................................   1      0    S05
.............................guugucaaaguggcugugaca............................................................   127    0    S05
.............................guugucaaaguggcugugacau...........................................................   9      0    S05
.............................guugucaaaguggcugugacaug..........................................................   1166   0    S05
.............................guugucaaaguggcugugacauguu........................................................   1      0    S05
..............................uugucaaaguggcugugaca............................................................   4      0    S05
..............................uugucaaaguggcugugacaug..........................................................   22     0    S05
...............................................acauguuagagauuuaugcuuc.........................................   2      0    S05
......................................................................uaucacagccagcuuugaug....................   3      0    S05
......................................................................uaucacagccagcuuugauga...................   5      0    S05
......................................................................uaucacagccagcuuugaugag..................   46     0    S05
......................................................................uaucacagccagcuuugaugagc.................   153    0    S05
......................................................................uaucacagccagcuuugaugagcg................   481    0    S05
.......................................................................aucacagccagcuuugaugagcg................   2      0    S05
```

Provisional ID	:	Scaffold2425_35140
Score total	:	25119.6
Score for star read(s)	:	3.9
Score for read counts	:	25112.6
Score for mfe	:	1.5
Score for randfold	:	1.6
Score for cons. seed	:	
Total read count	:	49269
Mature read count	:	49148
Loop read count	:	0
Star read count	:	121

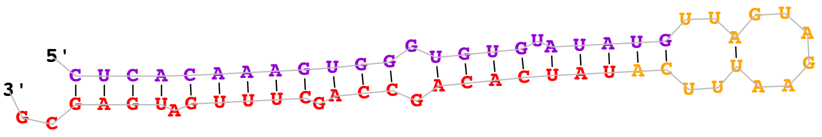

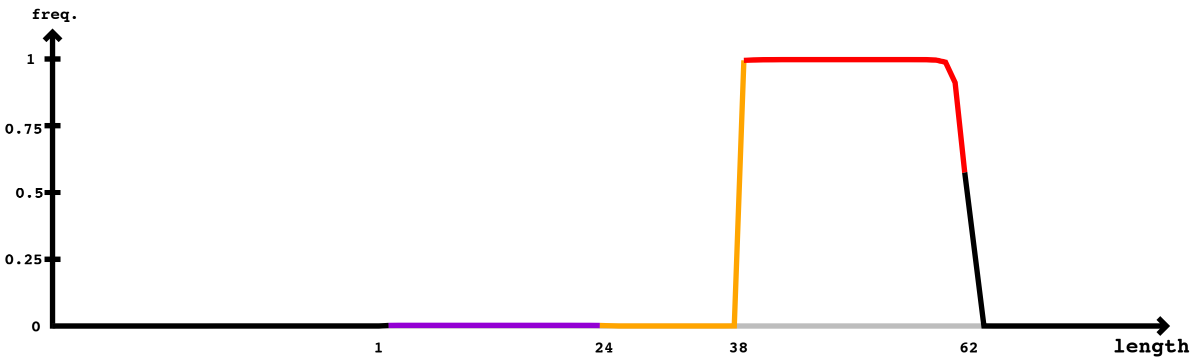

Star Mature

```
5'- gcgcauaaaauugagcauaguggcuuuucuucucucacaaaguggguguguauauguuaguagaauuucauaucacagccagcuuugaugagcggagugagguuaaauuucauca   -3'   obs
    gcgcauaaaauugagcauaguggcuuuucuucucucacaaaguggguguguauauguuaguagaauuucauaucacagccagcuuugaugagcggagugagguuaaauuucauca          exp
    ((.((......)).))....(((((((.((((.(((((((((((.((((.(((((..(......)..)))))))))).))).))))).)))).)))).))))))).........   reads    mm    sample
    ......................................................................uaucacagccagcuuugaug.........................   3        0     S05
    ......................................................................uaucacagccagcuuugauga........................   5        0     S05
    ......................................................................uaucacagccagcuuugaugag.......................   46       0     S05
    ......................................................................uaucacagccagcuuugaugagc......................   153      0     S05
    ......................................................................uaucacagccagcuuugaugagcg.....................   481      0     S05
    .......................................................................aucacagccagcuuugaugagcg.....................   2        0     S05

    ...............cauaguggcuuuucuucu..................................................................................   1        0     S10
    ...............cauaguggcuuuucuucuc.................................................................................   1        0     S10
    .................................cucacaaagugggugugua...............................................................   1        0     S10
    .................................cucacaaaguggguguguauau............................................................   11       0     S10
    .................................cucacaaaguggguguguauaug...........................................................   35       0     S10
    ..................................ucacaaaguggguguguauaug...........................................................   4        0     S10
    ................................................uguauauguuaguagaauuuca.............................................   1        0     S10
    ................................................uguauauguuaguagaauuucau............................................   1        0     S10
    .....................................................................auaucacagccagcuuugaugagc......................   1        0     S10
    ......................................................................uaucacagccagcuuuga...........................   1        0     S10
    ......................................................................uaucacagccagcuuugau..........................   1        0     S10
    ......................................................................uaucacagccagcuuugaug.........................   17       0     S10
    ......................................................................uaucacagccagcuuugauga........................   154      0     S10
    ......................................................................uaucacagccagcuuugaugag.......................   1455     0     S10
    ......................................................................uaucacagccagcuuugaugagc......................   6203     0     S10
    ......................................................................uaucacagccagcuuugaugagcg.....................   10360    0     S10
    ......................................................................uaucacagccagcuuugaugagcgg....................   4        0     S10
    ......................................................................uaucacagccagcuuugaugagcgga...................   2        0     S10
    .......................................................................aucacagccagcuuugaugag.......................   3        0     S10
    .......................................................................aucacagccagcuuugaugagc......................   23       0     S10
    .......................................................................aucacagccagcuuugaugagcg.....................   1        0     S10
    ........................................................................ucacagccagcuuugaugagc......................   12       0     S10
    ........................................................................ucacagccagcuuugaugagcg.....................   7        0     S10
    .........................................................................cacagccagcuuugaugagcg.....................   2        0     S10
    ..........................................................................acagccagcuuugaugag.......................   4        0     S10
    ..........................................................................acagccagcuuugaugagc......................   2        0     S10
```

```
                                  Star                                 Mature
gcgcauaaaaauugagcauaguggcuuuucuucucucacaaaguggguguguauauguuaguagaauuucauaucacagccagcuuugaugagcggagugagguuaaauuucauca
...........................................................................acagccagcuuugaugagcg.....................   1      0      S10

.......................................................................uaucacagccagcuuugaug.........................   1      0      S01
.......................................................................uaucacagccagcuuugaugag.......................   5      0      S01
.......................................................................uaucacagccagcuuugaugagc......................   14     0      S01
.......................................................................uaucacagccagcuuugaugagcg.....................   69     0      S01
........................................................................aucacagccagcuuugaugagc......................   1      0      S01

..................................cucacaaaguggguguguauaug...........................................................   1      0      S06
...................................ucacaaaguggguguguauaug...........................................................   1      0      S06
.........................................................uuaguagaauuucauaucacagccagcuuugaugagcg.....................   1      0      S06
.......................................................................uaucacagccagcuuugaug.........................   1      0      S06
.......................................................................uaucacagccagcuuugauga........................   3      0      S06
.......................................................................uaucacagccagcuuugaugag.......................   60     0      S06
.......................................................................uaucacagccagcuuugaugagc......................   215    0      S06
.......................................................................uaucacagccagcuuugaugagcg.....................   512    0      S06
........................................................................aucacagccagcuuugaugagcg.....................   2      0      S06

..................................cucacaaaguggguguguau..............................................................   1      0      S07
..................................cucacaaaguggguguguauau............................................................   1      0      S07
..................................cucacaaaguggguguguauaug...........................................................   4      0      S07
...................................ucacaaaguggguguguauaug...........................................................   2      0      S07
......................................................................auaucacagccagcuuugaugag.......................   1      0      S07
......................................................................auaucacagccagcuuugaugagc......................   1      0      S07
.......................................................................uaucacagccagcuuuga...........................   1      0      S07
.......................................................................uaucacagccagcuuugau..........................   1      0      S07
.......................................................................uaucacagccagcuuugaug.........................   10     0      S07
.......................................................................uaucacagccagcuuugauga........................   36     0      S07
.......................................................................uaucacagccagcuuugaugag.......................   372    0      S07
.......................................................................uaucacagccagcuuugaugagc......................   1165   0      S07
.......................................................................uaucacagccagcuuugaugagcg.....................   3181   0      S07
.......................................................................uaucacagccagcuuugaugagcgg....................   4      0      S07
........................................................................aucacagccagcuuugaugag.......................   1      0      S07
........................................................................aucacagccagcuuugaugagc......................   2      0      S07
........................................................................aucacagccagcuuugaugagcg.....................   3      0      S07
...........................................................................acagccagcuuugaugagcg.....................   1      0      S07

...................................ucacaaaguggguguguaua.............................................................   1      0      S03
......................................................................auaucacagccagcuuugaugagc......................   1      0      S03
.......................................................................uaucacagccagcuuuga...........................   3      0      S03
.......................................................................uaucacagccagcuuugauga........................   4      0      S03
.......................................................................uaucacagccagcuuugaugag.......................   22     0      S03
.......................................................................uaucacagccagcuuugaugagc......................   114    0      S03
.......................................................................uaucacagccagcuuugaugagcg.....................   352    0      S03
.......................................................................uaucacagccagcuuugaugagcgg....................   1      0      S03
.......................................................................uaucacagccagcuuugaugagcgga...................   1      0      S03
........................................................................aucacagccagcuuugaugagc......................   1      0      S03
........................................................................aucacagccagcuuugaugagcg.....................   1      0      S03

...............gcauaguggcuuuucuucu..................................................................................   2      0      S09
..................................cucacaaagugggugugua...............................................................   2      0      S09
..................................cucacaaaguggguguguauau............................................................   5      0      S09
..................................cucacaaaguggguguguauaug...........................................................   35     0      S09
..................................cucacaaaguggguguguauauguuaguagaauuuc..............................................   1      0      S09
...................................ucacaaaguggguguguauaug...........................................................   6      0      S09
........................................................guuaguagaauuucauaucacagcca..................................   1      0      S09
......................................................................auaucacagccagcuuugaugag.......................   1      0      S09
......................................................................auaucacagccagcuuugaugagc......................   4      0      S09
.......................................................................uaucacagccagcuuugaug.........................   21     0      S09
.......................................................................uaucacagccagcuuugauga........................   168    0      S09
.......................................................................uaucacagccagcuuugaugag.......................   1559   0      S09
.......................................................................uaucacagccagcuuugaugagc......................   7802   0      S09
.......................................................................uaucacagccagcuuugaugagcg.....................   10721  0      S09
.......................................................................uaucacagccagcuuugaugagcgg....................   7      0      S09
.......................................................................uaucacagccagcuuugaugagcgga...................   3      0      S09
.......................................................................uaucacagccagcuuugaugagcggag..................   1      0      S09
........................................................................aucacagccagcuuugaugagc......................   21     0      S09
........................................................................aucacagccagcuuugaugagcg.....................   1      0      S09
.........................................................................ucacagccagcuuugaugagc......................   9      0      S09
.........................................................................ucacagccagcuuugaugagcg.....................   2      0      S09
```

```
                                 Star                                 Mature
gcgcauaaaauugagcauaguggcuuuucuucucucacaaaguggguguguauauguuaguagaauuucauaucacagccagcuuugaugagcggagugagguuaaauuucauca
.........................................................................cacagccagcuuugaugag.......................    1      0    S09
.........................................................................cacagccagcuuugaugagc......................    1      0    S09
..........................................................................acagccagcuuugaugag.......................    3      0    S09
..........................................................................acagccagcuuugaugagcg.....................    3      0    S09

.................................cucacaaaguggguguguau..............................................................    1      0    S08
.................................cucacaaaguggguguguauaug...........................................................    7      0    S08
..................................ucacaaaguggguguguauaug...........................................................    2      0    S08
......................................................................uaucacagccagcuuugaug.........................    2      0    S08
......................................................................uaucacagccagcuuugauga........................    16     0    S08
......................................................................uaucacagccagcuuugaugag.......................    177    0    S08
......................................................................uaucacagccagcuuugaugagc......................    652    0    S08
......................................................................uaucacagccagcuuugaugagcg.....................    2025   0    S08
......................................................................uaucacagccagcuuugaugagcgg....................    2      0    S08
......................................................................uaucacagccagcuuugaugagcgga...................    1      0    S08
.......................................................................aucacagccagcuuugaugagc......................    4      0    S08
.......................................................................aucacagccagcuuugaugagcg.....................    5      0    S08
........................................................................ucacagccagcuuugaug.........................    1      0    S08

......................................................................uaucacagccagcuuugaug.........................    5      0    S02
......................................................................uaucacagccagcuuugauga........................    2      0    S02
......................................................................uaucacagccagcuuugaugag.......................    14     0    S02
......................................................................uaucacagccagcuuugaugagc......................    24     0    S02
......................................................................uaucacagccagcuuugaugagcg.....................    94     0    S02
.......................................................................aucacagccagcuuugaugagcg.....................    1      0    S02
........................................................................ucacagccagcuuugaugag.......................    2      0    S02
..........................................................................acagccagcuuugaugagcg.....................    1      0    S02

..................................ucacaaaguggguguguauaug...........................................................    1      0    S04
........................................................uuaguagaauuucauaucacagccagcu...............................    1      0    S04
.....................................................................auaucacagccagcuuugaugag.......................    1      0    S04
......................................................................uaucacagccagcuuugaug.........................    4      0    S04
......................................................................uaucacagccagcuuugauga........................    4      0    S04
......................................................................uaucacagccagcuuugaugag.......................    47     0    S04
......................................................................uaucacagccagcuuugaugagc......................    157    0    S04
......................................................................uaucacagccagcuuugaugagcg.....................    471    0    S04
.......................................................................aucacagccagcuuugaugagcg.....................    2      0    S04
........................................................................ucacagccagcuuugauga........................    1      0    S04
```

Provisional ID	:	Scaffold2425_35142
Score total	:	12697.4
Score for star read(s)	:	3.9
Score for read counts	:	12690.2
Score for mfe	:	1.8
Score for randfold	:	1.6
Score for cons. seed	:	
Total read count	:	24903
Mature read count	:	23679
Loop read count	:	0
Star read count	:	1224

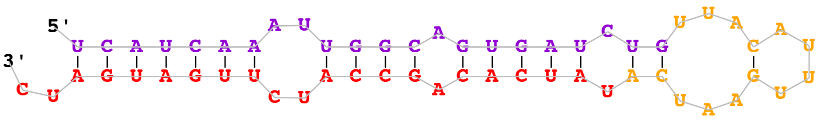

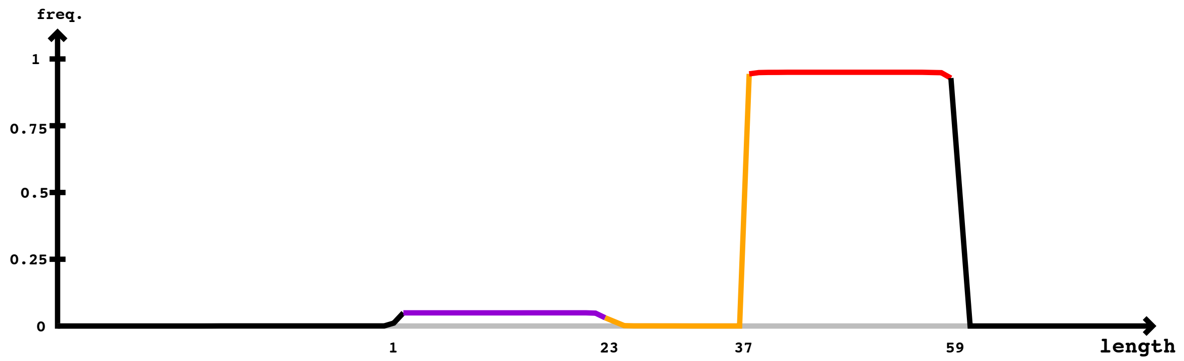

Star **Mature**

```
5'- uacuuggauccgaugcuuucuuuuaguuuucugaucaucaaauuggcagugaucuguuacauuugaaucauaucacagccaucuugaugauccgaaggcucaucagggcguc   -3'    obs
    uacuuggauccgaugcuuucuuuuaguuuucugaucaucaaauuggcagugaucuguuacauuugaaucauaucacagccaucuugaugauccgaaggcucaucagggcguc         exp
    ...........((((((((.....(((((((.(((((((((..((((.(((((.((...(....)...)).))))).))))..))))))))).)))))))....))))))))   reads     mm      sample
    ...............cuuucuuuuaguuuucuga..............................................................................   1         0       S04
    .................................aucaucaaauuggcagugaucu.........................................................   6         0       S04
    ..................................ucaucaaauuggcagugaucu.........................................................   3         0       S04
    ..................................ucaucaaauuggcagugaucug........................................................   10        0       S04
    ..................................ucaucaaauuggcagugaucuguu......................................................   2         0       S04
    ......................................................................uaucacagccaucuugauga......................   1         0       S04
    ......................................................................uaucacagccaucuugaugau.....................   8         0       S04
    ......................................................................uaucacagccaucuugaugauc....................   198       0       S04
    .......................................................................aucacagccaucuugaugaucc...................   8         0       S04
    .......................................................................................ugauccgaaggcucaucagggc...   1         0       S04

    ..................................ucaucaaauuggcagugaucug........................................................   3         0       S02
    ......................................................................uaucacagccaucuugau........................   1         0       S02
    ......................................................................uaucacagccaucuugaug.......................   2         0       S02
    ......................................................................uaucacagccaucuugaugau.....................   11        0       S02
    ......................................................................uaucacagccaucuugaugauc....................   47        0       S02
    .......................................................................aucacagccaucuugaugaucc...................   2         0       S02

    .......auccgaugcuuucuuuuaguuuucuga..............................................................................   1         0       S08
    ...............cuuucuuuuaguuuucuga..............................................................................   2         0       S08
    .................................aucaucaaauuggcagugaucu.........................................................   8         0       S08
    ..................................ucaucaaauuggcagugauc..........................................................   4         0       S08
    ..................................ucaucaaauuggcagugaucu.........................................................   30        0       S08
    ..................................ucaucaaauuggcagugaucug........................................................   86        0       S08
    ..................................ucaucaaauuggcagugaucuguu......................................................   4         0       S08
    ..................................ucaucaaauuggcagugaucuguuacau..................................................   1         0       S08
    ..................................ucaucaaauuggcagugaucuguuacauu.................................................   1         0       S08
    ................................................gugaucuguuacauuugaauca..........................................   1         0       S08
    ......................................................................uaucacagccaucuugaug.......................   3         0       S08
    ......................................................................uaucacagccaucuugaugau.....................   15        0       S08
    ......................................................................uaucacagccaucuugaugauc....................   752       0       S08
    ......................................................................uaucacagccaucuugaugaucc...................   3         0       S08
    .......................................................................aucacagccaucuugaugauc....................   7         0       S08
```

```
                                  Star                                Mature
uacuuggauccgaugcuuucuuuuaguuuucugaucaucaaauuggcagugaucuguuacauuugaaucauaucacagccaucuugaugauccgaaggcucaucagggcguc
.......................................................................aucacagccaucuugaugaucc...................   6      0    S08
........................................................................ucacagccaucuugaugauc....................   4      0    S08
.........................................................................cacagccaucuugaugauc....................   2      0    S08
..........................................................................acagccaucuugaugauc....................   2      0    S08
..........................................................................acagccaucuugaugaucc...................   1      0    S08

..............gcuuucuuuuaguuuucuga..............................................................................   1      0    S09
...............cuuucuuuuaguuuucuga..............................................................................   1      0    S09
.................................aucaucaaauuggcagugaucu.........................................................   76     0    S09
.................................aucaucaaauuggcagugaucug........................................................   16     0    S09
.................................aucaucaaauuggcagugaucuguu......................................................   1      0    S09
..................................ucaucaaauuggcagugauc..........................................................   14     0    S09
..................................ucaucaaauuggcagugaucu.........................................................   71     0    S09
..................................ucaucaaauuggcagugaucug........................................................   317    0    S09
..................................ucaucaaauuggcagugaucugu.......................................................   9      0    S09
..................................ucaucaaauuggcagugaucuguu......................................................   5      0    S09
..................................ucaucaaauuggcagugaucuguuacauu.................................................   1      0    S09
.....................................ucaaauuggcagugaucug........................................................   1      0    S09
................................................gugaucuguuacauuugaauca..........................................   1      0    S09
......................................................................uaucacagccaucuugau........................   2      0    S09
......................................................................uaucacagccaucuugaug.......................   6      0    S09
......................................................................uaucacagccaucuugauga......................   5      0    S09
......................................................................uaucacagccaucuugaugau.....................   173    0    S09
......................................................................uaucacagccaucuugaugauc....................   9779   0    S09
......................................................................uaucacagccaucuugaugaucc...................   27     0    S09
.......................................................................aucacagccaucuugaugauc....................   7      0    S09
.......................................................................aucacagccaucuugaugaucc...................   10     0    S09
........................................................................ucacagccaucuugaugauc....................   10     0    S09
........................................................................ucacagccaucuugaugaucc...................   1      0    S09
..........................................................................acagccaucuugaugauc....................   2      0    S09

...............cuuucuuuuaguuuucuga..............................................................................   1      0    S03
.................................aucaucaaauuggcagugaucu.........................................................   4      0    S03
..................................ucaucaaauuggcagugaucu.........................................................   3      0    S03
..................................ucaucaaauuggcagugaucug........................................................   1      0    S03
..................................ucaucaaauuggcagugaucugu.......................................................   1      0    S03
......................................................................uaucacagccaucuugaug.......................   4      0    S03
......................................................................uaucacagccaucuugaugau.....................   10     0    S03
......................................................................uaucacagccaucuugaugauc....................   102    0    S03
......................................................................uaucacagccaucuugaugaucc...................   2      0    S03
.......................................................................aucacagccaucuugaugauc....................   1      0    S03
.......................................................................aucacagccaucuugaugaucc...................   2      0    S03

.................................aucaucaaauuggcagugauc..........................................................   2      0    S07
.................................aucaucaaauuggcagugaucu.........................................................   15     0    S07
.................................aucaucaaauuggcagugaucug........................................................   8      0    S07
..................................ucaucaaauuggcagugauc..........................................................   1      0    S07
..................................ucaucaaauuggcagugaucu.........................................................   11     0    S07
..................................ucaucaaauuggcagugaucug........................................................   34     0    S07
..................................ucaucaaauuggcagugaucuguuacauuu................................................   1      0    S07
......................................................................uaucacagccaucuugauga......................   1      0    S07
......................................................................uaucacagccaucuugaugau.....................   34     0    S07
......................................................................uaucacagccaucuugaugauc....................   1778   0    S07
......................................................................uaucacagccaucuugaugaucc...................   9      0    S07
.......................................................................aucacagccaucuugaugauc....................   3      0    S07
.......................................................................aucacagccaucuugaugaucc...................   19     0    S07
..........................................................................acagccaucuugaugauc....................   1      0    S07

.................................aucaucaaauuggcagugaucu.........................................................   2      0    S01
..................................ucaucaaauuggcagugaucuguu......................................................   2      0    S01
......................................................................uaucacagccaucuugaugau.....................   6      0    S01
......................................................................uaucacagccaucuugaugauc....................   31     0    S01
.......................................................................aucacagccaucuugaugaucc...................   2      0    S01

...............cuuucuuuuaguuuucuga..............................................................................   1      0    S06
.................................aucaucaaauuggcagugaucu.........................................................   12     0    S06
.................................aucaucaaauuggcagugaucug........................................................   3      0    S06
..................................ucaucaaauuggcagugaucu.........................................................   3      0    S06
..................................ucaucaaauuggcagugaucug........................................................   10     0    S06
..................................ucaucaaauuggcagugaucugu.......................................................   2      0    S06
```

Star **Mature**

```
uacuuggauccgaugcuuucuuuuaguuuucugaucaucaaauuggcagugaucuguuacauuugaaucauaucacagccaucuugaugauccgaaggcucaucagggcguc
......................................................................uaucacagccaucuugaug.......................	1	0	S06
......................................................................uaucacagccaucuugauga......................	1	0	S06
......................................................................uaucacagccaucuugaugau.....................	9	0	S06
......................................................................uaucacagccaucuugaugauc....................	256	0	S06
......................................................................uaucacagccaucuugaugaucc...................	2	0	S06
.......................................................................aucacagccaucuugaugauc....................	2	0	S06
.......................................................................aucacagccaucuugaugaucc...................	11	0	S06
.........................................................................cacagccaucuugaugauc....................	1	0	S06

.................................aucaucaaauuggcagugauc..........................................................	1	0	S10
.................................aucaucaaauuggcagugaucu.........................................................	91	0	S10
.................................aucaucaaauuggcagugaucug........................................................	9	0	S10
.................................aucaucaaauuggcagugaucugu.......................................................	1	0	S10
..................................ucaucaaauuggcagugauc..........................................................	7	0	S10
..................................ucaucaaauuggcagugaucu.........................................................	85	0	S10
..................................ucaucaaauuggcagugaucug........................................................	208	0	S10
..................................ucaucaaauuggcagugaucugu.......................................................	11	0	S10
..................................ucaucaaauuggcagugaucuguu......................................................	7	0	S10
..................................ucaucaaauuggcagugaucuguuac....................................................	1	0	S10
..................................ucaucaaauuggcagugaucuguuaca...................................................	1	0	S10
..................................ucaucaaauuggcagugaucuguuacauu.................................................	1	0	S10
...............................................agugaucuguuacauuugaauc...........................................	1	0	S10
......................................................................uaucacagccaucuugaug.......................	5	0	S10
......................................................................uaucacagccaucuugauga......................	8	0	S10
......................................................................uaucacagccaucuugaugau.....................	223	0	S10
......................................................................uaucacagccaucuugaugauc....................	9765	0	S10
......................................................................uaucacagccaucuugaugaucc...................	33	0	S10
.......................................................................aucacagccaucuugaugau.....................	1	0	S10
.......................................................................aucacagccaucuugaugauc....................	2	0	S10
.......................................................................aucacagccaucuugaugaucc...................	29	0	S10
........................................................................ucacagccaucuugaugaucc...................	2	0	S10
..........................................................................acagccaucuugaugauc....................	4	0	S10

.................................aucaucaaauuggcagugauc..........................................................	1	0	S05
.................................aucaucaaauuggcagugaucu.........................................................	8	0	S05
.................................aucaucaaauuggcagugaucug........................................................	1	0	S05
..................................ucaucaaauuggcagugauc..........................................................	1	0	S05
..................................ucaucaaauuggcagugaucu.........................................................	1	0	S05
..................................ucaucaaauuggcagugaucug........................................................	8	0	S05
..................................ucaucaaauuggcagugaucugu.......................................................	1	0	S05
..................................ucaucaaauuggcagugaucuguu......................................................	1	0	S05
......................................................................uaucacagccaucuugauga......................	2	0	S05
......................................................................uaucacagccaucuugaugau.....................	13	0	S05
......................................................................uaucacagccaucuugaugauc....................	197	0	S05
......................................................................uaucacagccaucuugaugaucc...................	1	0	S05
.......................................................................aucacagccaucuugaugauc....................	3	0	S05
.......................................................................aucacagccaucuugaugaucc...................	8	0	S05
```

Provisional ID	:	Scaffold2425_35134
Score total	:	11670.8
Score for star read(s)	:	3.9
Score for read counts	:	11663.4
Score for mfe	:	1.9
Score for randfold	:	1.6
Score for cons. seed	:	
Total read count	:	22889
Mature read count	:	21495
Loop read count	:	1
Star read count	:	1393

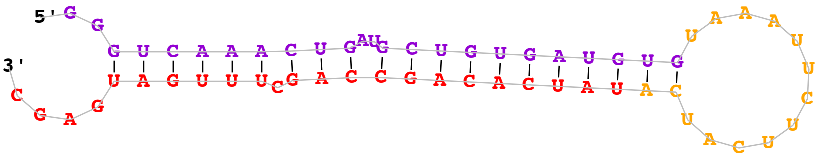

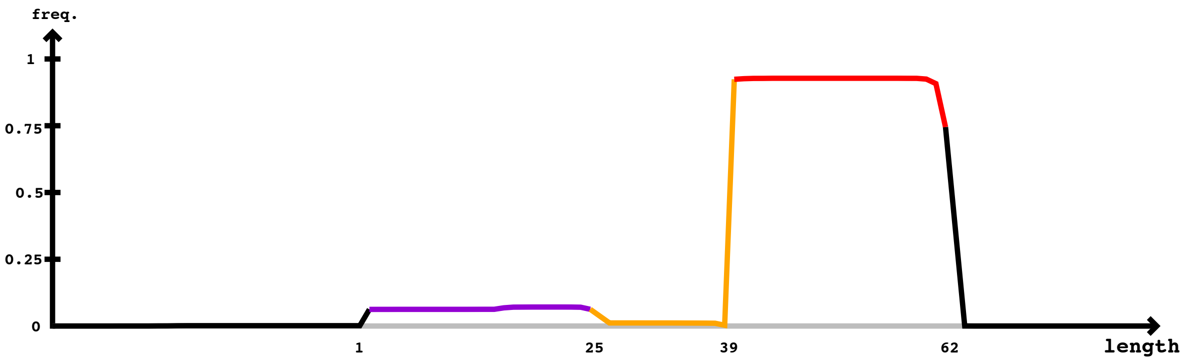


```
                                  Star                                    Mature
5'- gaauggucuuagauugauugcauauacuugcgggucaaacugaugcugugauguguaaauucuucaucauaucacagccagcuuugaugagcagguguauguuuaauauucaa  -3'  obs
    gaauggucuuagauugauugcauauacuugcgggucaaacugaugcugugauguguaaauucuucaucauaucacagccagcuuugaugagcagguguauguuuaauauucaa       exp
    (((((((.......))).(((((((((((..((((((((..((((((((((............)))))))))))))).))))))..))))))))))))....)))))..       reads  mm  sample
    .....gucuuagauugauugcauauacuug...................................................................................   1      0   S05
    ...............................gggucaaacugaugcugugaugu...........................................................   2      0   S05
    ...............................gggucaaacugaugcugugaugug..........................................................   23     0   S05
    ...............................gggucaaacugaugcugugauguguaaauucu..................................................   1      0   S05
    ...............................gggucaaacugaugcugugauguguaaauucuucauca............................................   1      0   S05
    ..........................................augcugugauguguaaauucuucauc.............................................   1      0   S05
    ..........................................augcugugauguguaaauucuucaucau...........................................   1      0   S05
    .............................................cugugauguguaaauucuucau..............................................   1      0   S05
    .............................................cugugauguguaaauucuucauc.............................................   15     0   S05
    .............................................cugugauguguaaauucuucauca............................................   1      0   S05
    ..............................................ugugauguguaaauucuucauc.............................................   3      0   S05
    .....................................................................uaucacagccagcuuugaug........................   3      0   S05
    .....................................................................uaucacagccagcuuugauga.......................   5      0   S05
    .....................................................................uaucacagccagcuuugaugag......................   46     0   S05
    .....................................................................uaucacagccagcuuugaugagc.....................   153    0   S05
    .....................................................................uaucacagccagcuuugaugagca....................   23     0   S05

    .........uagauugauugcauauacuugc..................................................................................   2      0   S10
    ..........agauugauugcauauacuugc..................................................................................   1      0   S10
    ...........gauugauugcauauacuugc..................................................................................   2      0   S10
    ............auugauugcauauacuugc..................................................................................   2      0   S10
    ...............................gggucaaacugaugcugugau.............................................................   1      0   S10
    ...............................gggucaaacugaugcugugaug............................................................   8      0   S10
    ...............................gggucaaacugaugcugugaugu...........................................................   79     0   S10
    ...............................gggucaaacugaugcugugaugug..........................................................   447    0   S10
    ...............................gggucaaacugaugcugugaugugu.........................................................   3      0   S10
    ...............................gggucaaacugaugcugugauguguaaauu....................................................   1      0   S10
    ...............................gggucaaacugaugcugugauguguaaauucu..................................................   2      0   S10
    ...............................gggucaaacugaugcugugauguguaaauucuu.................................................   1      0   S10
    ...............................gggucaaacugaugcugugauguguaaauucuuc................................................   1      0   S10
    ...............................gggucaaacugaugcugugauguguaaauucuuca...............................................   1      0   S10
    ...............................gggucaaacugaugcugugauguguaaauucuucau..............................................   1      0   S10
    ...............................gggucaaacugaugcugugauguguaaauucuucauc.............................................   1      0   S10
```

Star | Mature

```
gaauggucuuagauugauugcauauacuugcgggucaaacugaugcugugauguguaaauucuucaucauaucacagccagcuuugaugagcagguguauguuuaaauauucaa
...............................gggucaaacugaugcugugauguguaaauucuucauca.............................................  10  0  S10
................................ggucaaacugaugcugugaugu............................................................  1  0  S10
................................ggucaaacugaugcugugaugug...........................................................  1  0  S10
..................................ucaaacugaugcugugaugug...........................................................  1  0  S10
.............................................cugugauguguaaauucuucau...............................................  3  0  S10
.............................................cugugauguguaaauucuucauc..............................................  18  0  S10
.............................................cugugauguguaaauucuucauca.............................................  1  0  S10
..............................................ugugauguguaaauucuucauc..............................................  17  0  S10
...............................................gugauguguaaauucuucauc..............................................  2  0  S10
...............................................gugauguguaaauucuucauca.............................................  1  0  S10
................................................ugauguguaaauucuucauc..............................................  2  0  S10
................................................ugauguguaaauucuucauca.............................................  1  0  S10
.....................................................uguaaauucuucaucaua...........................................  1  0  S10
.......................................................uaaauucuucaucauaucacagccagcuuugaugagc......................  1  0  S10
...............................................................ucaucauaucacagccagcuuugaugagc......................  1  0  S10
....................................................................auaucacagccagcuuugaugagc......................  1  0  S10
.....................................................................uaucacagccagcuuuga...........................  1  0  S10
.....................................................................uaucacagccagcuuugau..........................  1  0  S10
.....................................................................uaucacagccagcuuugaug.........................  17  0  S10
.....................................................................uaucacagccagcuuugauga........................  154  0  S10
.....................................................................uaucacagccagcuuugaugag.......................  1455  0  S10
.....................................................................uaucacagccagcuuugaugagc......................  6203  0  S10
.....................................................................uaucacagccagcuuugaugagca.....................  232  0  S10
.....................................................................uaucacagccagcuuugaugagcag....................  1  0  S10
......................................................................aucacagccagcuuugaugag.......................  3  0  S10
......................................................................aucacagccagcuuugaugagc......................  23  0  S10
.......................................................................ucacagccagcuuugaugagc......................  12  0  S10
.........................................................................acagccagcuuugaugag.......................  4  0  S10
.........................................................................acagccagcuuugaugagc......................  2  0  S10

...............................gggucaaacugaugcugugaugu............................................................  1  0  S01
.............................................cugugauguguaaauucuucauc..............................................  1  0  S01
..............................................ugugauguguaaauucuucauca.............................................  1  0  S01
.....................................................................uaucacagccagcuuugaug.........................  1  0  S01
.....................................................................uaucacagccagcuuugaugag.......................  5  0  S01
.....................................................................uaucacagccagcuuugaugagc......................  14  0  S01
.....................................................................uaucacagccagcuuugaugagca.....................  2  0  S01
......................................................................aucacagccagcuuugaugagc......................  1  0  S01

...............................gggucaaacugaugcugugaug.............................................................  1  0  S06
...............................gggucaaacugaugcugugaugu............................................................  11  0  S06
...............................gggucaaacugaugcugugaugug...........................................................  67  0  S06
...............................gggucaaacugaugcugugaugugu..........................................................  1  0  S06
...............................gggucaaacugaugcugugauguguaaauucuuca................................................  1  0  S06
................................ggucaaacugaugcugugaug.............................................................  1  0  S06
....................................aaacugaugcugugaugug...........................................................  1  0  S06
.............................................cugugauguguaaauucuucauc..............................................  14  0  S06
.............................................cugugauguguaaauucuucauca.............................................  3  0  S06
..............................................ugugauguguaaauucuucauc..............................................  3  0  S06
.....................................................................uaucacagccagcuuugaug.........................  1  0  S06
.....................................................................uaucacagccagcuuugauga........................  3  0  S06
.....................................................................uaucacagccagcuuugaugag.......................  60  0  S06
.....................................................................uaucacagccagcuuugaugagc......................  215  0  S06
.....................................................................uaucacagccagcuuugaugagca.....................  6  0  S06

...........gauugauugcauauacuugc...................................................................................  1  0  S07
...............................gggucaaacugaugcugugaugu............................................................  12  0  S07
...............................gggucaaacugaugcugugaugug...........................................................  143  0  S07
...............................gggucaaacugaugcugugauguguaaauucu...................................................  1  0  S07
...............................gggucaaacugaugcugugauguguaaauucuucauc..............................................  2  0  S07
...............................gggucaaacugaugcugugauguguaaauucuucauca.............................................  2  0  S07
...........................................ugcugugauguguaaauucuuc.................................................  1  0  S07
...........................................ugcugugauguguaaauucuucauc..............................................  1  0  S07
.............................................cugugauguguaaauucuuca................................................  1  0  S07
.............................................cugugauguguaaauucuucauc..............................................  8  0  S07
..............................................ugugauguguaaauucuucauc..............................................  2  0  S07
.......................................................uaaauucuucaucauaucacagccagcuuugaugagc......................  5  0  S07
....................................................................auaucacagccagcuuugaugag.......................  1  0  S07
....................................................................auaucacagccagcuuugaugagc......................  1  0  S07
.....................................................................uaucacagccagcuuuga...........................  1  0  S07
```

Star **Mature**

```
gaauggucuuagauugauugcauauacuugcgggucaaacugaugcugugauguguaaauucuucaucauaucacagccagcuuugaugagcagguguauguuuaaauauucaa
.....................................................................uaucacagccagcuuugau..........................   1      0   S07
.....................................................................uaucacagccagcuuugaug.........................   10     0   S07
.....................................................................uaucacagccagcuuugauga........................   36     0   S07
.....................................................................uaucacagccagcuuugaugag.......................   372    0   S07
.....................................................................uaucacagccagcuuugaugagc......................   1165   0   S07
.....................................................................uaucacagccagcuuugaugagca.....................   47     0   S07
......................................................................aucacagccagcuuugaugag.......................   1      0   S07
......................................................................aucacagccagcuuugaugagc......................   2      0   S07
..........................................................................cagccagcuuugaugagca.....................   1      0   S07

...............................gggucaaacugaugcugugaug.............................................................   1      0   S03
...............................gggucaaacugaugcugugaugu............................................................   2      0   S03
...............................gggucaaacugaugcugugaugug...........................................................   20     0   S03
...............................gggucaaacugaugcugugauguguaaau......................................................   2      0   S03
.............................................cugugauguguaaauucuucauc..............................................   5      0   S03
..............................................ugugauguguaaauucuucauc..............................................   1      0   S03
....................................................................auaucacagccagcuuugaugagc......................   1      0   S03
.....................................................................uaucacagccagcuuuga...........................   3      0   S03
.....................................................................uaucacagccagcuuugauga........................   4      0   S03
.....................................................................uaucacagccagcuuugaugag.......................   22     0   S03
.....................................................................uaucacagccagcuuugaugagc......................   114    0   S03
.....................................................................uaucacagccagcuuugaugagca.....................   15     0   S03
......................................................................aucacagccagcuuugaugagc......................   1      0   S03

.....gucuuagauugauugcauauacuugc...................................................................................   2      0   S09
.........uagauugauugcauauacuugc...................................................................................   5      0   S09
...........gauugauugcauauacuugc...................................................................................   6      0   S09
............auugauugcauauacuugc...................................................................................   1      0   S09
...............................gggucaaacugaugcugugau..............................................................   1      0   S09
...............................gggucaaacugaugcugugaug.............................................................   3      0   S09
...............................gggucaaacugaugcugugaugu............................................................   58     0   S09
...............................gggucaaacugaugcugugaugug...........................................................   381    0   S09
...............................gggucaaacugaugcugugaugugu..........................................................   6      0   S09
...............................gggucaaacugaugcugugauguguaaauucu...................................................   1      0   S09
...............................gggucaaacugaugcugugauguguaaauucuuca................................................   2      0   S09
...............................gggucaaacugaugcugugauguguaaauucuucauc..............................................   2      0   S09
...............................gggucaaacugaugcugugauguguaaauucuucauca.............................................   7      0   S09
...............................gggucaaacugaugcugugauguguaaauucuucaucauaucacagccagcuuugaugagc......................   1      0   S09
..................................ucaaacugaugcugugaugu............................................................   1      0   S09
..........................................augcugugauguguaaauucuucau...............................................   1      0   S09
.............................................cugugauguguaaauucuucau...............................................   1      0   S09
.............................................cugugauguguaaauucuucauc..............................................   28     0   S09
.............................................cugugauguguaaauucuucauca.............................................   2      0   S09
..............................................ugugauguguaaauucuucauc..............................................   20     0   S09
..............................................ugugauguguaaauucuucauca.............................................   1      0   S09
...............................................gugauguguaaauucuucauc..............................................   3      0   S09
................................................ugauguguaaauucuucauca.............................................   1      0   S09
.......................................................uaaauucuucaucauaucacag.....................................   1      0   S09
.......................................................uaaauucuucaucauaucacagccagcuuugaugagc......................   2      0   S09
...............................................................ucaucauaucacagccagcuuugaugagc......................   1      0   S09
....................................................................auaucacagccagcuuugaugag.......................   1      0   S09
....................................................................auaucacagccagcuuugaugagc......................   4      0   S09
.....................................................................uaucacagccagcuuugaug.........................   21     0   S09
.....................................................................uaucacagccagcuuugauga........................   168    0   S09
.....................................................................uaucacagccagcuuugaugag.......................   1559   0   S09
.....................................................................uaucacagccagcuuugaugagc......................   7802   0   S09
.....................................................................uaucacagccagcuuugaugagca.....................   265    0   S09
.....................................................................uaucacagccagcuuugaugagcag....................   1      0   S09
......................................................................aucacagccagcuuugaugagc......................   21     0   S09
.......................................................................ucacagccagcuuugaugagc......................   9      0   S09
.......................................................................ucacagccagcuuugaugagca.....................   1      0   S09
........................................................................cacagccagcuuugaugag.......................   1      0   S09
........................................................................cacagccagcuuugaugagc......................   1      0   S09
.........................................................................acagccagcuuugaugag.......................   3      0   S09

...........gauugauugcauauacuugc...................................................................................   2      0   S08
...............................gggucaaacugaugcugugaugu............................................................   9      0   S08
...............................gggucaaacugaugcugugaugug...........................................................   67     0   S08
...............................gggucaaacugaugcugugauguguaaauucuu..................................................   3      0   S08
...............................gggucaaacugaugcugugauguguaaauucuucauca.............................................   10     0   S08
```

```
                                Star                                    Mature
gaauggucuuagauugauugcauauacuugcgggucaaacugaugcugugauguguaaauucuucaucauaucacagccagcuuugaugagcagguguauguuuaaauauucaa
...............................gggucaaacugaugcugugauguguaaauucuucaucau............................................	1	0	S08
.............................................cugugauguguaaauucuucauc..............................................	5	0	S08
..............................................ugugauguguaaauucuucauc..............................................	1	0	S08
.....................................................................uaucacagccagcuuugaug.........................	2	0	S08
.....................................................................uaucacagccagcuuugauga........................	16	0	S08
.....................................................................uaucacagccagcuuugaugag.......................	177	0	S08
.....................................................................uaucacagccagcuuugaugagc......................	652	0	S08
.....................................................................uaucacagccagcuuugaugagca.....................	33	0	S08
.....................................................................uaucacagccagcuuugaugagcag....................	3	0	S08
......................................................................aucacagccagcuuugaugagc......................	4	0	S08
.......................................................................ucacagccagcuuugaug.........................	1	0	S08

...............................gggucaaacugaugcugugaugu............................................................	4	0	S04
...............................gggucaaacugaugcugugaugug...........................................................	22	0	S04
...............................gggucaaacugaugcugugauguguaaauucuucauca.............................................	1	0	S04
.............................................cugugauguguaaauucuucauc..............................................	14	0	S04
..............................................ugugauguguaaauucuucauc..............................................	7	0	S04
....................................................................auaucacagccagcuuugaugag.......................	1	0	S04
.....................................................................uaucacagccagcuuugaug.........................	4	0	S04
.....................................................................uaucacagccagcuuugauga........................	4	0	S04
.....................................................................uaucacagccagcuuugaugag.......................	47	0	S04
.....................................................................uaucacagccagcuuugaugagc......................	157	0	S04
.....................................................................uaucacagccagcuuugaugagca.....................	30	0	S04
.......................................................................ucacagccagcuuugauga........................	1	0	S04

...........gauugauugcauauacuugc...................................................................................	1	0	S02
............auugauugcauauacuugc...................................................................................	1	0	S02
...............................gggucaaacugaugcugugaugu............................................................	1	0	S02
...............................gggucaaacugaugcugugaugug...........................................................	9	0	S02
...............................gggucaaacugaugcugugaugugu..........................................................	2	0	S02
.............................................cugugauguguaaauucuucau...............................................	1	0	S02
.............................................cugugauguguaaauucuucauc..............................................	5	0	S02
..............................................ugugauguguaaauucuucauc..............................................	3	0	S02
.....................................................................uaucacagccagcuuugaug.........................	5	0	S02
.....................................................................uaucacagccagcuuugauga........................	2	0	S02
.....................................................................uaucacagccagcuuugaugag.......................	14	0	S02
.....................................................................uaucacagccagcuuugaugagc......................	24	0	S02
.....................................................................uaucacagccagcuuugaugagca.....................	14	0	S02
.......................................................................ucacagccagcuuugaugag.......................	2	0	S02
```

Provisional ID : Scaffold2425_35144
Score total : 8763.9
Score for star read(s) : 3.9
Score for read counts : 8756.4
Score for mfe : 2
Score for randfold : 1.6
Score for cons. seed :
Total read count : 17187
Mature read count : 15446
Loop read count : 0
Star read count : 1741

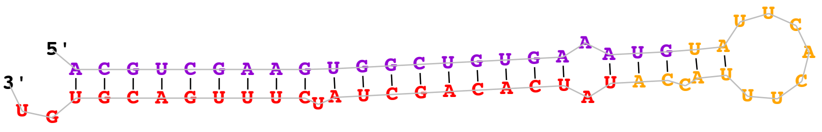

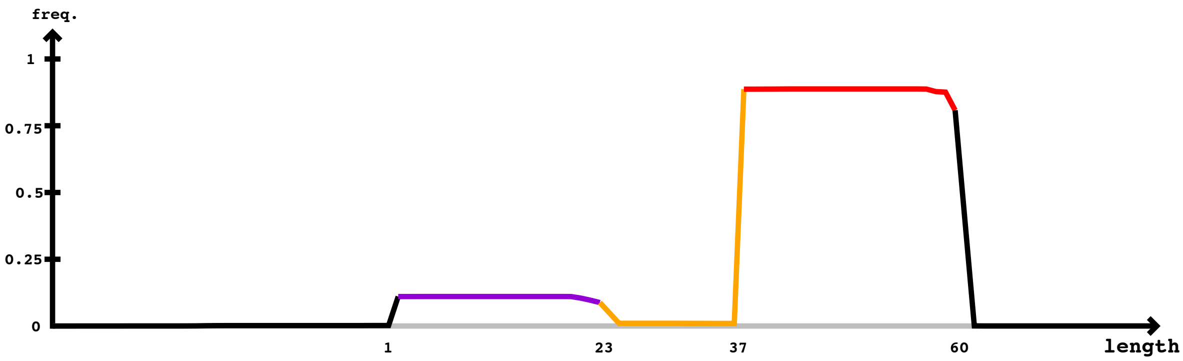


```
                                                  Star                              Mature
5'- aagaggauugcuggauuaccuguguucauuaugcacgucgaaguggcugugaaauguauucacuuuaccauaucacagcuaucuuugacguguuuaaugggcauucuuucauc  -3'  obs
    aagaggauugcuggauuaccuguguucauuaugcacgucgaaguggcugugaaauguauucacuuuaccauaucacagcuaucuuugacguguuuaaugggcauucuuucauc       exp
    ..(((((.....((....)).((((((((((.((((((((((((((((((((.(((((.......)).))).))))))))).))))))))))).))))))))))))))).....    reads    mm    sample
    ..............auuaccuguguucauuaugc...............................................................................    6        0     S10
    ...............uuaccuguguucauuaugc...............................................................................    1        0     S10
    ................uaccuguguucauuaugc...............................................................................    1        0     S10
    ...............................ugcacgucgaaguggcuguga.............................................................    1        0     S10
    ...............................ugcacgucgaaguggcugugaaa...........................................................    1        0     S10
    .................................cacgucgaaguggcugugaa............................................................    1        0     S10
    .................................cacgucgaaguggcugugaaaug.........................................................    1        0     S10
    ..................................acgucgaaguggcuguga.............................................................    1        0     S10
    ..................................acgucgaaguggcugugaa............................................................    35       0     S10
    ..................................acgucgaaguggcugugaaa...........................................................    46       0     S10
    ..................................acgucgaaguggcugugaaau..........................................................    57       0     S10
    ..................................acgucgaaguggcugugaaaug.........................................................    466      0     S10
    ..................................acgucgaaguggcugugaaaugu........................................................    3        0     S10
    ..................................acgucgaaguggcugugaaauguauucacu.................................................    1        0     S10
    ..................................acgucgaaguggcugugaaauguauucacuuua..............................................    2        0     S10
    ..................................acgucgaaguggcugugaaauguauucacuuuacc............................................    1        0     S10
    ..................................acgucgaaguggcugugaaauguauucacuuuacca...........................................    48       0     S10
    ....................................gucgaaguggcugugaaaug.........................................................    1        0     S10
    ...................................................................ccauaucacagcuaucuuugac........................    1        0     S10
    ......................................................................uaucacagcuaucuuugac........................    1        0     S10
    ......................................................................uaucacagcuaucuuugacg.......................    54       0     S10
    ......................................................................uaucacagcuaucuuugacgu......................    8        0     S10
    ......................................................................uaucacagcuaucuuugacgug.....................    403      0     S10
    ......................................................................uaucacagcuaucuuugacgugu....................    6538     0     S10
    ......................................................................uaucacagcuaucuuugacguguu...................    286      0     S10
    ......................................................................uaucacagcuaucuuugacguguuu..................    2        0     S10
    .......................................................................aucacagcuaucuuugacgugu....................    1        0     S10
    .........................................................................cacagcuaucuuugacgugu....................    1        0     S10
    ..........................................................................acagcuaucuuugacguguu...................    1        0     S10
    ...........................................................................cagcuaucuuugacgugu....................    2        0     S10
    .............................................................................................uuaaugggcauucuuucauc    2        0     S10

    ...........uggauuaccuguguucauuaugc...............................................................................    1        0     S05
```

Star **Mature**

```
aagaggauugcuggauuaccuguguucauuaugcacgucgaaguggcugugaaauguauucacuuuaccauaucacagcuaucuuugacguguuuaaugggcauucuuucauc
................uaccuguguucauuaugc...............................................................................	1	0	S05
..................................acgucgaaguggcugugaaa...........................................................	3	0	S05
..................................acgucgaaguggcugugaaau..........................................................	3	0	S05
..................................acgucgaaguggcugugaaaug.........................................................	18	0	S05
..................................acgucgaaguggcugugaaauguauucacuuuacca...........................................	4	0	S05
......................................................................uaucacagcuaucuuugacg.......................	7	0	S05
......................................................................uaucacagcuaucuuugacgu......................	4	0	S05
......................................................................uaucacagcuaucuuugacgug.....................	47	0	S05
......................................................................uaucacagcuaucuuugacgugu....................	49	0	S05
......................................................................uaucacagcuaucuuugacguguu...................	29	0	S05

..................................acgucgaaguggcugugaa............................................................	5	0	S07
..................................acgucgaaguggcugugaaa...........................................................	9	0	S07
..................................acgucgaaguggcugugaaau..........................................................	10	0	S07
..................................acgucgaaguggcugugaaaug.........................................................	114	0	S07
..................................acgucgaaguggcugugaaaugu........................................................	1	0	S07
..................................acgucgaaguggcugugaaauguauucacuu................................................	1	0	S07
..................................acgucgaaguggcugugaaauguauucacuuua..............................................	1	0	S07
..................................acgucgaaguggcugugaaauguauucacuuuacc............................................	1	0	S07
..................................acgucgaaguggcugugaaauguauucacuuuacca...........................................	27	0	S07
....................................gucgaaguggcugugaaaug.........................................................	1	0	S07
.....................................ucgaaguggcugugaaaugu........................................................	1	0	S07
......................................................................uaucacagcuaucuuugacg.......................	18	0	S07
......................................................................uaucacagcuaucuuugacgu......................	5	0	S07
......................................................................uaucacagcuaucuuugacgug.....................	101	0	S07
......................................................................uaucacagcuaucuuugacgugu....................	341	0	S07
......................................................................uaucacagcuaucuuugacguguu...................	54	0	S07
......................................................................uaucacagcuaucuuugacguguuu..................	1	0	S07
........................................................................ucacagcuaucuuugacgug.....................	1	0	S07
.............................................................................................uuaaugggcauucuuucauc	1	0	S07

.............gauuaccuguguucauuaugc...............................................................................	1	0	S06
..................................acgucgaaguggcugugaa............................................................	3	0	S06
..................................acgucgaaguggcugugaaa...........................................................	1	0	S06
..................................acgucgaaguggcugugaaau..........................................................	5	0	S06
..................................acgucgaaguggcugugaaaug.........................................................	33	0	S06
..................................acgucgaaguggcugugaaauguauucacu.................................................	1	0	S06
..................................acgucgaaguggcugugaaauguauucacuu................................................	1	0	S06
..................................acgucgaaguggcugugaaauguauucacuuuacca...........................................	7	0	S06
..................................acgucgaaguggcugugaaauguauucacuuuaccau..........................................	1	0	S06
......................................................................uaucacagcuaucuuugac........................	1	0	S06
......................................................................uaucacagcuaucuuugacg.......................	14	0	S06
......................................................................uaucacagcuaucuuugacgu......................	3	0	S06
......................................................................uaucacagcuaucuuugacgug.....................	47	0	S06
......................................................................uaucacagcuaucuuugacgugu....................	79	0	S06
......................................................................uaucacagcuaucuuugacguguu...................	40	0	S06

..................................acgucgaaguggcugugaaau..........................................................	1	0	S01
......................................................................uaucacagcuaucuuugacg.......................	3	0	S01
......................................................................uaucacagcuaucuuugacgu......................	1	0	S01
......................................................................uaucacagcuaucuuugacgug.....................	13	0	S01
......................................................................uaucacagcuaucuuugacgugu....................	20	0	S01
......................................................................uaucacagcuaucuuugacguguu...................	1	0	S01

......auugcuggauuaccuguguucauuaugc...............................................................................	2	0	S08
.............gauuaccuguguucauuaugc...............................................................................	2	0	S08
................uaccuguguucauuaugc...............................................................................	1	0	S08
..................................acgucgaaguggcugugaa............................................................	5	0	S08
..................................acgucgaaguggcugugaaa...........................................................	13	0	S08
..................................acgucgaaguggcugugaaau..........................................................	20	0	S08
..................................acgucgaaguggcugugaaaug.........................................................	128	0	S08
..................................acgucgaaguggcugugaaaugu........................................................	1	0	S08
..................................acgucgaaguggcugugaaauguauucacuu................................................	1	0	S08
..................................acgucgaaguggcugugaaauguauucacuuuacca...........................................	25	0	S08
......................................................................uaucacagcuaucuuugac........................	1	0	S08
......................................................................uaucacagcuaucuuugacg.......................	5	0	S08
......................................................................uaucacagcuaucuuugacgu......................	1	0	S08
......................................................................uaucacagcuaucuuugacgug.....................	55	0	S08
......................................................................uaucacagcuaucuuugacgugu....................	247	0	S08
......................................................................uaucacagcuaucuuugacguguu...................	30	0	S08
```

Star Mature

```
aagaggauugcuggauuaccuguguucauuaugcacgucgaaguggcugugaaauguauucacuuuaccauaucacagcuaucuuugacguguuuaaugggcauucuuucauc
..........................................................................acagcuaucuuugacgugu....................   1      0   S08

.agaggauugcuggauuaccuguguucauuaugc...............................................................................   1      0   S09
............ggauuaccuguguucauuaugc...............................................................................   1      0   S09
.............gauuaccuguguucauuaugc...............................................................................   1      0   S09
..............auuaccuguguucauuaugc...............................................................................   4      0   S09
................uaccuguguucauuaugc...............................................................................   1      0   S09
...............................ugcacgucgaaguggcuguga.............................................................   2      0   S09
..................................acgucgaaguggcugugaa............................................................   49     0   S09
..................................acgucgaaguggcugugaaa...........................................................   59     0   S09
..................................acgucgaaguggcugugaaau..........................................................   52     0   S09
..................................acgucgaaguggcugugaaaug.........................................................   538    0   S09
..................................acgucgaaguggcugugaaaugu........................................................   3      0   S09
..................................acgucgaaguggcugugaaauguauucacuu................................................   1      0   S09
..................................acgucgaaguggcugugaaauguauucacuuua..............................................   2      0   S09
..................................acgucgaaguggcugugaaauguauucacuuuacca...........................................   50     0   S09
..................................................................accauaucacagcuaucuuugacgug.....................   1      0   S09
......................................................................uaucacagcuaucuuuga.........................   1      0   S09
......................................................................uaucacagcuaucuuugac........................   1      0   S09
......................................................................uaucacagcuaucuuugacg.......................   36     0   S09
......................................................................uaucacagcuaucuuugacgu......................   3      0   S09
......................................................................uaucacagcuaucuuugacgug.....................   410    0   S09
......................................................................uaucacagcuaucuuugacgugu....................   5857   0   S09
......................................................................uaucacagcuaucuuugacguguu...................   298    0   S09
......................................................................uaucacagcuaucuuugacguguuu..................   1      0   S09
........................................................................ucacagcuaucuuugacgug.....................   1      0   S09
........................................................................ucacagcuaucuuugacgugu....................   2      0   S09
..........................................................................acagcuaucuuugacgugu....................   4      0   S09
...........................................................................cagcuaucuuugacgugu....................   1      0   S09
.............................................................................................uuaaugggcauucuuucauc   1      0   S09

..................................acgucgaaguggcugugaaa...........................................................   2      0   S03
..................................acgucgaaguggcugugaaau..........................................................   3      0   S03
..................................acgucgaaguggcugugaaaug.........................................................   14     0   S03
..................................acgucgaaguggcugugaaauguauucacuuuacca...........................................   2      0   S03
......................................................................uaucacagcuaucuuugacg.......................   3      0   S03
......................................................................uaucacagcuaucuuugacgug.....................   27     0   S03
......................................................................uaucacagcuaucuuugacgugu....................   30     0   S03
......................................................................uaucacagcuaucuuugacguguu...................   5      0   S03

..............auuaccuguguucauuaugc...............................................................................   1      0   S02
...............uuaccuguguucauuaugc...............................................................................   1      0   S02
..................................acgucgaaguggcugugaaa...........................................................   1      0   S02
..................................acgucgaaguggcugugaaaug.........................................................   3      0   S02
......................................................................uaucacagcuaucuuugac........................   1      0   S02
......................................................................uaucacagcuaucuuugacg.......................   5      0   S02
......................................................................uaucacagcuaucuuugacgu......................   2      0   S02
......................................................................uaucacagcuaucuuugacgug.....................   22     0   S02
......................................................................uaucacagcuaucuuugacgugu....................   20     0   S02
......................................................................uaucacagcuaucuuugacguguu...................   5      0   S02

...............uuaccuguguucauuaugc...............................................................................   1      0   S04
..................................acgucgaaguggcugugaaa...........................................................   1      0   S04
..................................acgucgaaguggcugugaaau..........................................................   6      0   S04
..................................acgucgaaguggcugugaaaug.........................................................   24     0   S04
..................................acgucgaaguggcugugaaauguauucacuuuacca...........................................   1      0   S04
......................................................................uaucacagcuaucuuugac........................   1      0   S04
......................................................................uaucacagcuaucuuugacg.......................   19     0   S04
......................................................................uaucacagcuaucuuugacgu......................   8      0   S04
......................................................................uaucacagcuaucuuugacgug.....................   58     0   S04
......................................................................uaucacagcuaucuuugacgugu....................   75     0   S04
......................................................................uaucacagcuaucuuugacguguu...................   34     0   S04
```

Provisional ID	:	Scaffold300_7827
Score total	:	8291.1
Score for star read(s)	:	3.9
Score for read counts	:	8284.3
Score for mfe	:	1.4
Score for randfold	:	1.6
Score for cons. seed	:	
Total read count	:	16261
Mature read count	:	16025
Loop read count	:	2
Star read count	:	234

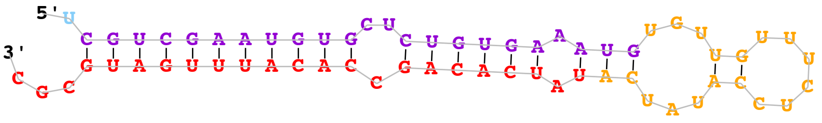

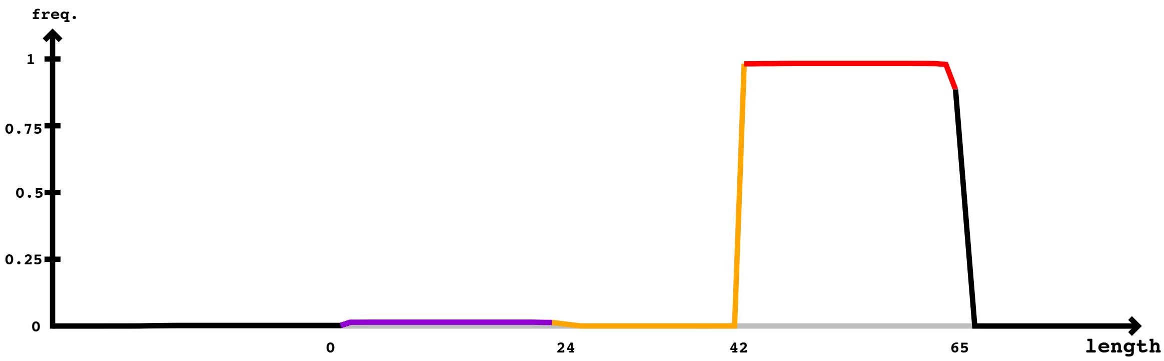


```
                                Star                                            Mature
5'- ucucucuuaaccaccacccucugcgaugucgucgaaugugcucugugaaauguguuguuucuccauaucauaucacagccacauuugaugcgcaucucauagguucuucug  -3'   obs
    ucucucuuaaccaccacccucugcgaugucgucgaaugugcucugugaaauguguuguuucuccauaucauaucacagccacauuugaugcgcaucucauagguucuucug         exp
    .................(((.((.((((((((((((((((..((((((.(((...((......))...))).)))))).))))))))))).))))).)).)))........   reads   mm   sample
    ........aaccaccacccucugcgaugu..................................................................................   2       0    S10
    .........accaccacccucugcgaugu..................................................................................   2       0    S10
    ..........ccaccacccucugcgaugu..................................................................................   1       0    S10
    ...........caccacccucugcgaugu..................................................................................   1       0    S10
    .............................cgucgaaugugcucugugaa..............................................................   6       0    S10
    .............................cgucgaaugugcucugugaaa.............................................................   2       0    S10
    .............................cgucgaaugugcucugugaaau............................................................   5       0    S10
    .............................cgucgaaugugcucugugaaaug...........................................................   65      0    S10
    ...............................ucgaaugugcucugugaaa.............................................................   1       0    S10
    .................................gaaugugcucugugaaaugu..........................................................   1       0    S10
    ....................................................uguuguuucuccauauca.........................................   1       0    S10
    ...................................................................ucauaucacagccacauuugaugcgc..................   1       0    S10
    ......................................................................uaucacagccacauuuga.......................   1       0    S10
    ......................................................................uaucacagccacauuugaug.....................   3       0    S10
    ......................................................................uaucacagccacauuugaugc....................   17      0    S10
    ......................................................................uaucacagccacauuugaugcg...................   570     0    S10
    ......................................................................uaucacagccacauuugaugcgc..................   5532    0    S10
    ......................................................................uaucacagccacauuugaugcgca.................   19      0    S10
    .......................................................................aucacagccacauuugaugcgc..................   1       0    S10
    ........................................................................ucacagccacauuugaugcgc..................   1       0    S10
    ..........................................................................acagccacauuugaugcgc..................   3       0    S10

    .............................cgucgaaugugcucugugaaa.............................................................   1       0    S05
    .............................cgucgaaugugcucugugaaaug...........................................................   7       0    S05
    ......................................................................uaucacagccacauuugaugcg...................   37      0    S05
    ......................................................................uaucacagccacauuugaugcgc..................   254     0    S05
    ......................................................................uaucacagccacauuugaugcgca.................   2       0    S05
    ..........................................................................acagccacauuugaugcgc..................   1       0    S05

    ........aaccaccacccucugcgaugu..................................................................................   2       0    S07
    .........accaccacccucugcgaugu..................................................................................   2       0    S07
    .............................cgucgaaugugcucugugaaau............................................................   2       0    S07
    .............................cgucgaaugugcucugugaaaug...........................................................   18      0    S07
```

Star **Mature**

```
ucucucuuaaccaccacccucugcgaugucgucgaaugugcucugugaaauguguuguuucuccauaucauaucacagccacauuugaugcgcaucucauagguucuucug
..............................gucgaaugugcucugugaaaug...........................................................  1     0  S07
......................................................................uaucacagccacauuugaugc....................  3     0  S07
......................................................................uaucacagccacauuugaugcg...................  107   0  S07
......................................................................uaucacagccacauuugaugcgc..................  630   0  S07
......................................................................uaucacagccacauuugaugcgca.................  2     0  S07

.............................cgucgaaugugcucugugaaau............................................................  1     0  S01
......................................................................uaucacagccacauuugaugcg...................  6     0  S01
......................................................................uaucacagccacauuugaugcgc..................  53    0  S01
......................................................................uaucacagccacauuugaugcgca.................  1     0  S01

.......uaaccaccacccucugcgaugu..................................................................................  1     0  S06
..........ccaccacccucugcgaugu..................................................................................  3     0  S06
...........caccacccucugcgaugu..................................................................................  1     0  S06
.............................cgucgaaugugcucugugaaau............................................................  3     0  S06
.............................cgucgaaugugcucugugaaaug...........................................................  13    0  S06
.............................cgucgaaugugcucugugaaauguguuguuucuccau.............................................  1     0  S06
......................................................................uaucacagccacauuugaugc....................  1     0  S06
......................................................................uaucacagccacauuugaugcg...................  46    0  S06
......................................................................uaucacagccacauuugaugcgc..................  301   0  S06

....ucuuaaccaccacccucugcgaugu..................................................................................  1     0  S08
........aaccaccacccucugcgaugu..................................................................................  5     0  S08
.........accaccacccucugcgaugu..................................................................................  2     0  S08
..........ccaccacccucugcgaugu..................................................................................  2     0  S08
...........caccacccucugcgaugu..................................................................................  2     0  S08
.............................cgucgaaugugcucugugaa..............................................................  1     0  S08
.............................cgucgaaugugcucugugaaa.............................................................  1     0  S08
.............................cgucgaaugugcucugugaaau............................................................  1     0  S08
.............................cgucgaaugugcucugugaaaug...........................................................  16    0  S08
......................................................................uaucacagccacauuugaugc....................  8     0  S08
......................................................................uaucacagccacauuugaugcg...................  84    0  S08
......................................................................uaucacagccacauuugaugcgc..................  511   0  S08
......................................................................uaucacagccacauuugaugcgca.................  4     0  S08
......................................................................uaucacagccacauuugaugcgcau................  1     0  S08
...........................................................................cagccacauuugaugcgc..................  3     0  S08

........aaccaccacccucugcgaugu..................................................................................  2     0  S09
.........accaccacccucugcgaug...................................................................................  1     0  S09
.........accaccacccucugcgaugu..................................................................................  1     0  S09
...........caccacccucugcgaugu..................................................................................  1     0  S09
.............................cgucgaaugugcucugugaa..............................................................  5     0  S09
.............................cgucgaaugugcucugugaaau............................................................  8     0  S09
.............................cgucgaaugugcucugugaaaug...........................................................  39    0  S09
..............................gucgaaugugcucugugaaaug...........................................................  1     0  S09
....................................................uguuguuucuccauauca.........................................  1     0  S09
......................................................................uaucacagccacauuugaug.....................  7     0  S09
......................................................................uaucacagccacauuugaugc....................  20    0  S09
......................................................................uaucacagccacauuugaugcg...................  609   0  S09
......................................................................uaucacagccacauuugaugcgc..................  6654  0  S09
......................................................................uaucacagccacauuugaugcgca.................  21    0  S09
.......................................................................aucacagccacauuugaugcgc..................  2     0  S09
........................................................................ucacagccacauuugaugcgc..................  8     0  S09
..........................................................................acagccacauuugaugcgc..................  4     0  S09

.....cuuaaccaccacccucugcgaugu..................................................................................  1     0  S03
........aaccaccacccucugcgaugu..................................................................................  1     0  S03
.............................cgucgaaugugcucugugaaau............................................................  1     0  S03
.............................cgucgaaugugcucugugaaaug...........................................................  17    0  S03
.............................cgucgaaugugcucugugaaaugu..........................................................  1     0  S03
...............................ucgaaugugcucugugaaaug...........................................................  1     0  S03
......................................................................uaucacagccacauuugau......................  1     0  S03
......................................................................uaucacagccacauuugaugc....................  1     0  S03
......................................................................uaucacagccacauuugaugcg...................  19    0  S03
......................................................................uaucacagccacauuugaugcgc..................  146   0  S03
......................................................................uaucacagccacauuugaugcgca.................  1     0  S03
..........................................................................acagccacauuugaugcgc..................  1     0  S03

........aaccaccacccucugcgaugu..................................................................................  1     0  S02
.............................cgucgaaugugcucugugaa..............................................................  1     0  S02
```

```
                             Star                                    Mature
ucucucuuaaccaccacccucugcgaugucgucgaaugugcucugugaaaugcuguuguuucuccauaucauaucacagccacauuugaugcgcaucucauagguucuucug
.............................cgucgaaugugcucugugaaau...........................................................    2    0    S02
.............................cgucgaaugugcucugugaaaug..........................................................    2    0    S02
......................................................................uaucacagccacauuugaugc....................    1    0    S02
......................................................................uaucacagccacauuugaugcg...................    9    0    S02
......................................................................uaucacagccacauuugaugcgc..................    67   0    S02

.........accaccacccucugcgaugu.................................................................................    1    0    S04
.............................cgucgaaugugcucugugaaau...........................................................    1    0    S04
.............................cgucgaaugugcucugugaaaug..........................................................    10   0    S04
......................................................................uaucacagccacauuugaugcg...................    39   0    S04
......................................................................uaucacagccacauuugaugcgc..................    213  0    S04
```

Provisional ID : Scaffold2425_35136
Score total : 1.7
Score for star read(s) : -1.3
Score for read counts : 0
Score for mfe : 1.4
Score for randfold : 1.6
Score for cons. seed :
Total read count : 4437
Mature read count : 4436
Loop read count : 0
Star read count : 1

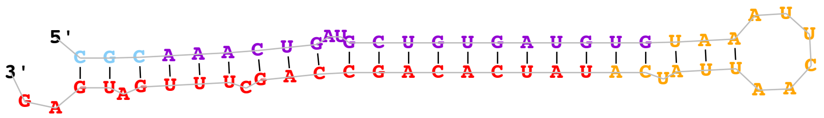

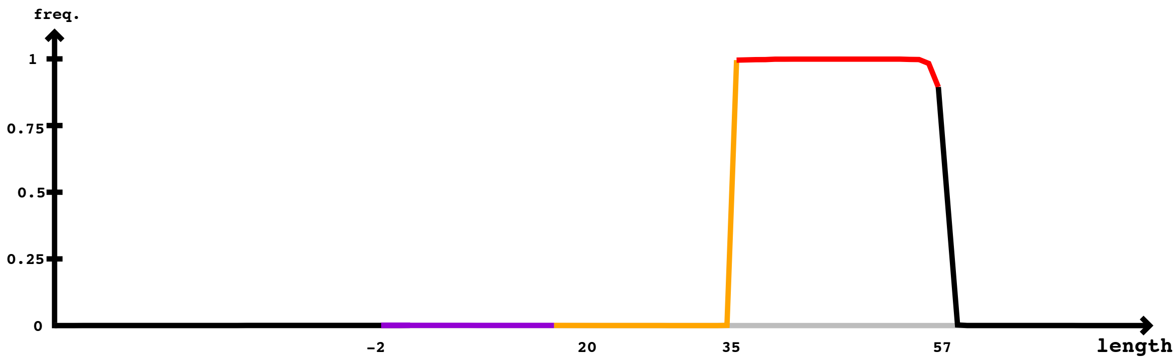

Star Mature

```
5'- cguuucauccaauggaauggucuuggauugaucgcaaacugaugcugugauguguaaauucaauuaucauaucacagccagcuuugaugaguggcgugaggucguuucaucc -3'   obs
    cguuucauccaauggaauggucuuggauugaucgcaaacugaugcugugauguguaaauucaauuaucauaucacagccagcuuugaugaguggcgugaggucguuucaucc       exp
    ...........((((((((..(((.......((((((((((..((((((((((((((......))).)))))))))))))).)))).))).......)))..))))))))..   reads   mm   sample
    ..................ggucuuggauugaucgcaaacu........................................................................   1       0    S09
    ....................................................................auaucacagccagcuuugaugag.....................   1       0    S09
    .....................................................................uaucacagccagcuuugaug.......................   21      0    S09
    .....................................................................uaucacagccagcuuugauga......................   168     0    S09
    .....................................................................uaucacagccagcuuugaugag.....................   1559    0    S09
    .....................................................................uaucacagccagcuuugaugagu....................   81      0    S09
    .....................................................................uaucacagccagcuuugaugagug...................   3       0    S09
    ........................................................................cacagccagcuuugaugag.....................   1       0    S09
    .........................................................................acagccagcuuugaugag.....................   3       0    S09
    ...........................................................................agccagcuuugaugaguggcgugaggucgu.......   1       0    S09

    ........................................gaugcugugauguguaaauuca..................................................   1       0    S03
    .....................................................................uaucacagccagcuuuga.........................   3       0    S03
    .....................................................................uaucacagccagcuuugauga......................   4       0    S03
    .....................................................................uaucacagccagcuuugaugag.....................   22      0    S03
    .....................................................................uaucacagccagcuuugaugagu....................   2       0    S03

    .guuucauccaauggaauggucuuggauugaucgcaaacuga......................................................................   1       0    S08
    .....................................................................uaucacagccagcuuugaug.......................   2       0    S08
    .....................................................................uaucacagccagcuuugauga......................   16      0    S08
    .....................................................................uaucacagccagcuuugaugag.....................   177     0    S08
    .....................................................................uaucacagccagcuuugaugagu....................   8       0    S08
    .....................................................................uaucacagccagcuuugaugagug...................   1       0    S08
    .......................................................................ucacagccagcuuugaug.......................   1       0    S08

    .....................................................................uaucacagccagcuuugaug.......................   5       0    S02
    .....................................................................uaucacagccagcuuugauga......................   2       0    S02
    .....................................................................uaucacagccagcuuugaugag.....................   14      0    S02
    .......................................................................ucacagccagcuuugaugag.....................   2       0    S02

    ....................................................................auaucacagccagcuuugaugag.....................   1       0    S04
    .....................................................................uaucacagccagcuuugaug.......................   4       0    S04
    .....................................................................uaucacagccagcuuugauga......................   4       0    S04
```

Star **Mature**

```
cguuucauccaauggaauggucuuggauugaucgcaaacugaugcugugauguguaaaauucaauuaucauaucacagccagcuuugaugaguggcgugaggucguuucaucc
......................................................................uaucacagccagcuuugaugag.....................   47     0   S04
........................................................................ucacagccagcuuugauga......................   1      0   S04

......................................................................uaucacagccagcuuugaug.......................   3      0   S05
......................................................................uaucacagccagcuuugauga......................   5      0   S05
......................................................................uaucacagccagcuuugaugag.....................   46     0   S05
......................................................................uaucacagccagcuuugaugagu....................   2      0   S05

......................................................................uaucacagccagcuuuga.........................   1      0   S10
......................................................................uaucacagccagcuuugau........................   1      0   S10
......................................................................uaucacagccagcuuugaug.......................   17     0   S10
......................................................................uaucacagccagcuuugauga......................   154    0   S10
......................................................................uaucacagccagcuuugaugag.....................   1455   0   S10
......................................................................uaucacagccagcuuugaugagu....................   64     0   S10
......................................................................uaucacagccagcuuugaugagug..................   4      0   S10
.......................................................................aucacagccagcuuugaugag.....................   3      0   S10
..........................................................................acagccagcuuugaugag.....................   4      0   S10

......................................................................uaucacagccagcuuugaug.......................   1      0   S01
......................................................................uaucacagccagcuuugaugag.....................   5      0   S01

...................................aaacugaugcugugaugug...........................................................   1      0   S06
......................................................................uaucacagccagcuuugaug.......................   1      0   S06
......................................................................uaucacagccagcuuugauga......................   3      0   S06
......................................................................uaucacagccagcuuugaugag.....................   60     0   S06
......................................................................uaucacagccagcuuugaugagu....................   6      0   S06

.....................................................................auaucacagccagcuuugaugag.....................   1      0   S07
......................................................................uaucacagccagcuuuga.........................   1      0   S07
......................................................................uaucacagccagcuuugau........................   1      0   S07
......................................................................uaucacagccagcuuugaug.......................   10     0   S07
......................................................................uaucacagccagcuuugauga......................   36     0   S07
......................................................................uaucacagccagcuuugaugag.....................   372    0   S07
......................................................................uaucacagccagcuuugaugagu....................   25     0   S07
......................................................................uaucacagccagcuuugaugagug..................   1      0   S07
.......................................................................aucacagccagcuuugaugag.....................   1      0   S07
```

Provisional ID	:	Scaffold2425_35138
Score total	:	0.8
Score for star read(s)	:	-1.3
Score for read counts	:	0
Score for mfe	:	0.5
Score for randfold	:	1.6
Score for cons. seed	:	
Total read count	:	4436
Mature read count	:	4436
Loop read count	:	0
Star read count	:	0

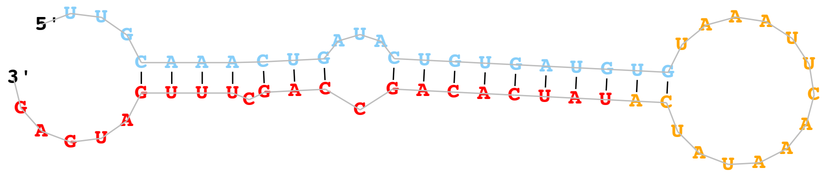

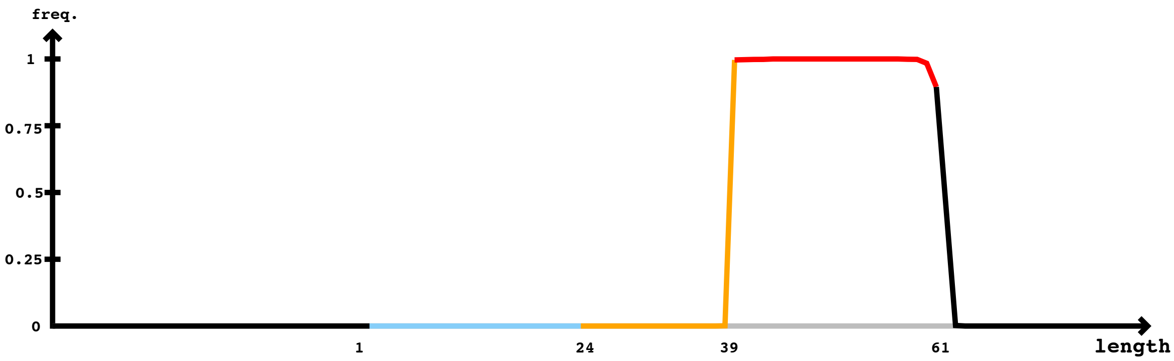

Star Mature

```
5'- aauuucaucauuuggaauggucuuagauugauugcaaacugauacugugauguguaaauucaaauaucauaucacagccagcuuugaugaguggcaugaggucguuucaucc   -3'    exp
    ............(((((((..((((.........(((((((...((((((((((.............)))))))))).))).))))..........))))..)))))))...   reads      mm          sample
    ....................................................................auaucacagccagcuuugaugag.....................   1          0           S07
    .....................................................................uaucacagccagcuuuga.........................   1          0           S07
    .....................................................................uaucacagccagcuuugau........................   1          0           S07
    .....................................................................uaucacagccagcuuugaug.......................   10         0           S07
    .....................................................................uaucacagccagcuuugauga......................   36         0           S07
    .....................................................................uaucacagccagcuuugaugag.....................   372        0           S07
    .....................................................................uaucacagccagcuuugaugagu....................   25         0           S07
    .....................................................................uaucacagccagcuuugaugagug...................   1          0           S07
    ......................................................................aucacagccagcuuugaugag.....................   1          0           S07

    .....................................................................uaucacagccagcuuugaug.......................   1          0           S06
    .....................................................................uaucacagccagcuuugauga......................   3          0           S06
    .....................................................................uaucacagccagcuuugaugag.....................   60         0           S06
    .....................................................................uaucacagccagcuuugaugagu....................   6          0           S06

    .....................................................................uaucacagccagcuuugaug.......................   1          0           S01
    .....................................................................uaucacagccagcuuugaugag.....................   5          0           S01

    .....................................................................uaucacagccagcuuuga.........................   1          0           S10
    .....................................................................uaucacagccagcuuugau........................   1          0           S10
    .....................................................................uaucacagccagcuuugaug.......................   17         0           S10
    .....................................................................uaucacagccagcuuugauga......................   154        0           S10
    .....................................................................uaucacagccagcuuugaugag.....................   1455       0           S10
    .....................................................................uaucacagccagcuuugaugagu....................   64         0           S10
    .....................................................................uaucacagccagcuuugaugagug...................   4          0           S10
    ......................................................................aucacagccagcuuugaugag.....................   3          0           S10
    .........................................................................acagccagcuuugaugag.....................   4          0           S10

    .....................................................................uaucacagccagcuuugaug.......................   3          0           S05
    .....................................................................uaucacagccagcuuugauga......................   5          0           S05
    .....................................................................uaucacagccagcuuugaugag.....................   46         0           S05
    .....................................................................uaucacagccagcuuugaugagu....................   2          0           S05

    ....................................................................auaucacagccagcuuugaugag.....................   1          0           S04
```

```
                              Star                                 Mature
aauuucaucauuuggaauggucuuagauugauugcaaacugauacugugauguguaaauucaaauaucauaucacagccagcuuugaugaguggcaugaggucguuucaucc
.....................................................................uaucacagccagcuuugaug.......................	4	0	S04
.....................................................................uaucacagccagcuuugauga......................	4	0	S04
.....................................................................uaucacagccagcuuugaugag.....................	47	0	S04
.......................................................................ucacagccagcuuugauga......................	1	0	S04

.....................................................................uaucacagccagcuuugaug.......................	5	0	S02
.....................................................................uaucacagccagcuuugauga......................	2	0	S02
.....................................................................uaucacagccagcuuugaugag.....................	14	0	S02
.......................................................................ucacagccagcuuugaugag.....................	2	0	S02

.....................................................................uaucacagccagcuuugaug.......................	2	0	S08
.....................................................................uaucacagccagcuuugauga......................	16	0	S08
.....................................................................uaucacagccagcuuugaugag.....................	177	0	S08
.....................................................................uaucacagccagcuuugaugagu....................	8	0	S08
.....................................................................uaucacagccagcuuugaugagug...................	1	0	S08
.......................................................................ucacagccagcuuugaug.......................	1	0	S08

.....................................................................uaucacagccagcuuuga.........................	3	0	S03
.....................................................................uaucacagccagcuuugauga......................	4	0	S03
.....................................................................uaucacagccagcuuugaugag.....................	22	0	S03
.....................................................................uaucacagccagcuuugaugagu....................	2	0	S03

....................................................................auaucacagccagcuuugaugag.....................	1	0	S09
.....................................................................uaucacagccagcuuugaug.......................	21	0	S09
.....................................................................uaucacagccagcuuugauga......................	168	0	S09
.....................................................................uaucacagccagcuuugaugag.....................	1559	0	S09
.....................................................................uaucacagccagcuuugaugagu....................	81	0	S09
.....................................................................uaucacagccagcuuugaugagug...................	3	0	S09
........................................................................cacagccagcuuugaugag.....................	1	0	S09
.........................................................................acagccagcuuugaugag.....................	3	0	S09
```

Provisional ID	:	Scaffold1792_29947
Score total	:	23.6
Score for star read(s)	:	3.9
Score for read counts	:	17
Score for mfe	:	1.1
Score for randfold	:	1.6
Score for cons. seed	:	
Total read count	:	45
Mature read count	:	31
Loop read count	:	0
Star read count	:	14

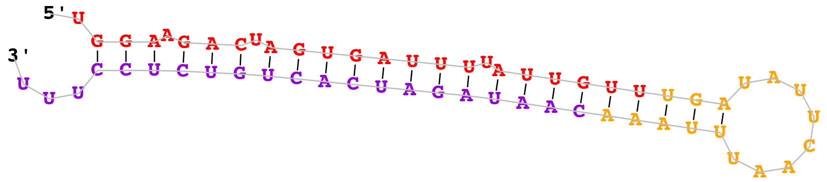

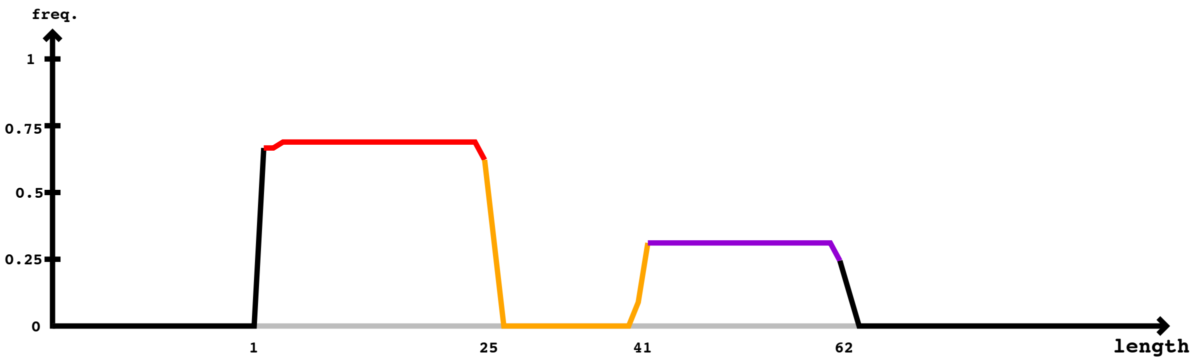

Mature **Star**

```
5'- aguagauuucauuuucugcuuggaagacuagugauuuuauuguuugauauucaauuuaaacaauagaucacugucuccuuucggugaaugaaaaaaaucgucacauccaccccc  -3'  obs
    aguagauuucauuuucugcuuggaagacuagugauuuuauuguuugauauucaauuuaaacaauagaucacugucuccuuucggugaaugaaaaaaaucgucacauccaccccc       exp
    ((((((........)))))).(((.(((.((((((((.(((((((((........)))))))))))))))))))))))....((((.(((.............))).))))...   reads  mm  sample
    ....................uggaagacuagugauuuuauuguu......................................................................   3      0   S08
    ............................................................caauagaucacugucuccuuu.................................   3      0   S08

    ....................uggaagacuagugauuuuauuguu......................................................................   2      0   S03
    ......................gaagacuagugauuuuauuguu......................................................................   1      0   S03
    ............................................................caauagaucacugucuccuuu.................................   1      0   S03

    ....................uggaagacuagugauuuuauugu.......................................................................   1      0   S09
    ....................uggaagacuagugauuuuauuguu......................................................................   13     0   S09
    ...........................................................acaauagaucacugucuccuuu.................................   1      0   S09

    ...........................................................acaauagaucacugucuccuu..................................   1      0   S02
    ............................................................caauagaucacugucuccuuu.................................   1      0   S02

    ....................uggaagacuagugauuuuauuguuu.....................................................................   1      0   S04
    ...........................................................acaauagaucacugucuccuu..................................   1      0   S04
    ............................................................caauagaucacugucuccuuu.................................   1      0   S04

    ....................uggaagacuagugauuuuauugu.......................................................................   2      0   S10
    ....................uggaagacuagugauuuuauuguu......................................................................   6      0   S10
    ............................................................caauagaucacugucuccuu..................................   1      0   S10
    ............................................................caauagaucacugucuccuuu.................................   1      0   S10

    ...........................................................acaauagaucacugucuccuuu.................................   1      0   S05
    ............................................................caauagaucacugucuccuuu.................................   1      0   S05

    ....................uggaagacuagugauuuuauuguu......................................................................   2      0   S07

    ............................................................caauagaucacugucuccuuu.................................   1      0   S06
```

Provisional ID	:	Scaffold406_10365
Score total	:	9868.8
Score for star read(s)	:	3.9
Score for read counts	:	9861.7
Score for mfe	:	1.6
Score for randfold	:	1.6
Score for cons. seed	:	
Total read count	:	19355
Mature read count	:	17608
Loop read count	:	0
Star read count	:	1747

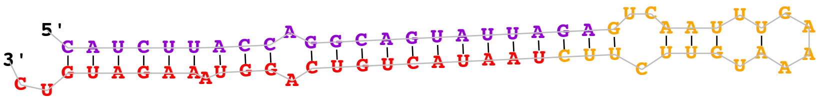

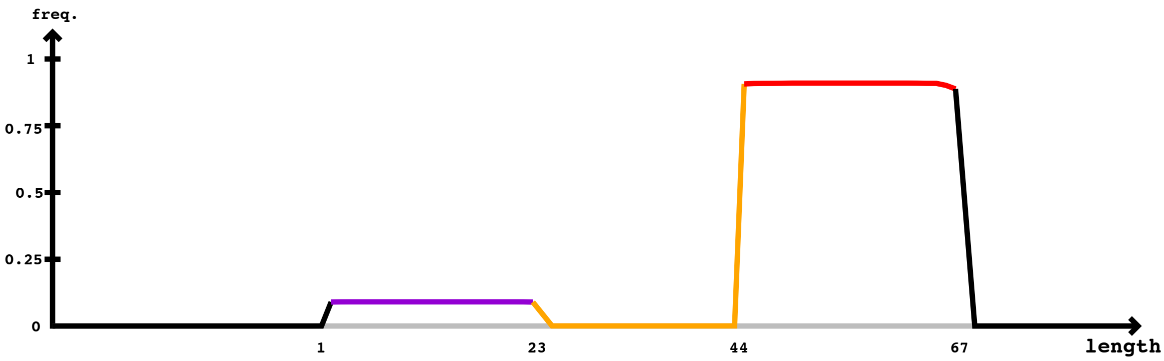

Star Mature

```
5'- augucuucaaggaaggaguaucgacgucaucuuaccaggcaguauuagagucaauuugaaaauguucuucuaauacugucagguaaagaugucuacgauguccucacgcau  -3'   obs
    augucuucaaggaaggaguaucgacgucaucuuaccaggcaguauuagagucaauuugaaaauguucuucuaauacugucagguaaagaugucuacgauguccucacgcau         exp
    ..........(..((((.(((((..(.(((((((((.(((((((((((((..(((.(....).))).))))))))))))).))).)))))).)..)))))))))..)....    reads   mm   sample
    ...........................caucuuaccaggcaguauuag...............................................................    1       0    S02
    ...........................caucuuaccaggcaguauuaga..............................................................    32      0    S02
    .....................................................................cuaauacugucagguaaagauguc..................    1       0    S02
    ......................................................................uaauacugucagguaaag.......................    2       0    S02
    ......................................................................uaauacugucagguaaaga......................    3       0    S02
    ......................................................................uaauacugucagguaaagau.....................    1       0    S02
    ......................................................................uaauacugucagguaaagaug....................    10      0    S02
    ......................................................................uaauacugucagguaaagaugu...................    23      0    S02
    ......................................................................uaauacugucagguaaagauguc..................    668     0    S02
    ......................................................................uaauacugucagguaaagaugucu.................    32      0    S02
    .......................................................................aauacugucagguaaagauguc..................    2       0    S02
    ........................................................................auacugucagguaaagauguc..................    2       0    S02
    ..........................................................................acugucagguaaagauguc..................    1       0    S02
    ...........................................................................cugucagguaaagauguc..................    1       0    S02

    ...........................caucuuaccaggcaguauuaga..............................................................    37      0    S04
    ......................................................................uaauacugucagguaaaga......................    3       0    S04
    ......................................................................uaauacugucagguaaagau.....................    1       0    S04
    ......................................................................uaauacugucagguaaagaug....................    21      0    S04
    ......................................................................uaauacugucagguaaagaugu...................    31      0    S04
    ......................................................................uaauacugucagguaaagauguc..................    831     0    S04
    ......................................................................uaauacugucagguaaagaugucu.................    23      0    S04
    ........................................................................auacugucagguaaagauguc..................    1       0    S04
    ...........................................................................cugucagguaaagauguc..................    1       0    S04

    ..........................ucaucuuaccaggcaguauuaga..............................................................    1       0    S08
    ...........................caucuuaccaggcaguauua................................................................    2       0    S08
    ...........................caucuuaccaggcaguauuag...............................................................    2       0    S08
    ...........................caucuuaccaggcaguauuaga..............................................................    268     0    S08
    ...........................caucuuaccaggcaguauuagagucaauuugaaaauguucuuc.........................................    3       0    S08
    ......................................................................uaauacugucagguaaagaug....................    8       0    S08
    ......................................................................uaauacugucagguaaagaugu...................    3       0    S08
    ......................................................................uaauacugucagguaaagauguc..................    1505    0    S08
```

```
                           Star                                      Mature
augucuucaaggaaggaguaucgacgucaucuuaccaggcaguauuagagucaauuugaaaauguucuucuaauacugucagguaaagaugucuacgauguccucacgcau
......................................................................uaauacugucagguaaagaugucu.................    22    0    S08
.......................................................................aauacugucagguaaagauguc..................    1     0    S08
.........................................................................uacugucagguaaagauguc..................    1     0    S08
..........................................................................acugucagguaaagauguc..................    2     0    S08
...........................................................................cugucagguaaagauguc..................    4     0    S08

...........................caucuuaccaggcaguau..................................................................    1     0    S09
...........................caucuuaccaggcaguauuag...............................................................    5     0    S09
...........................caucuuaccaggcaguauuaga..............................................................    486   0    S09
............................aucuuaccaggcaguauuaga..............................................................    4     0    S09
.............................ucuuaccaggcaguauuaga..............................................................    1     0    S09
.....................................................................cuaauacugucagguaaagauguc..................    1     0    S09
......................................................................uaauacugucagguaaaga......................    2     0    S09
......................................................................uaauacugucagguaaagaug....................    22    0    S09
......................................................................uaauacugucagguaaagaugu...................    58    0    S09
......................................................................uaauacugucagguaaagauguc..................    4063  0    S09
......................................................................uaauacugucagguaaagaugucu.................    63    0    S09
.......................................................................aauacugucagguaaagauguc..................    7     0    S09
.........................................................................uacugucagguaaagaugu...................    1     0    S09
..........................................................................acugucagguaaagauguc..................    2     0    S09
...........................................................................cugucagguaaagauguc..................    1     0    S09

...........................caucuuaccaggcaguauuaga..............................................................    12    0    S03
............................aucuuaccaggcaguauuaga..............................................................    1     0    S03
......................................................................uaauacugucagguaaagaug....................    13    0    S03
......................................................................uaauacugucagguaaagaugu...................    14    0    S03
......................................................................uaauacugucagguaaagauguc..................    442   0    S03
......................................................................uaauacugucagguaaagaugucu.................    13    0    S03
.......................................................................aauacugucagguaaagauguc..................    1     0    S03
.......................................................................aauacugucagguaaagaugucu.................    1     0    S03

...........................caucuuaccaggcaguauuag...............................................................    1     0    S07
...........................caucuuaccaggcaguauuaga..............................................................    279   0    S07
...........................caucuuaccaggcaguauuagagucaauuugaaaaugu..............................................    1     0    S07
...........................caucuuaccaggcaguauuagagucaauuugaaaauguucuuc.........................................    1     0    S07
............................aucuuaccaggcaguauuaga..............................................................    2     0    S07
.............................ucuuaccaggcaguauuaga..............................................................    1     0    S07
......................................................................uaauacugucagguaaagaug....................    12    0    S07
......................................................................uaauacugucagguaaagaugu...................    18    0    S07
......................................................................uaauacugucagguaaagauguc..................    2246  0    S07
......................................................................uaauacugucagguaaagaugucu.................    46    0    S07
.......................................................................aauacugucagguaaagauguc..................    2     0    S07

...........................caucuuaccaggcaguauuaga..............................................................    44    0    S06
............................aucuuaccaggcaguauuaga..............................................................    1     0    S06
......................................................................uaauacugucagguaaagaug....................    18    0    S06
......................................................................uaauacugucagguaaagaugu...................    15    0    S06
......................................................................uaauacugucagguaaagauguc..................    975   0    S06
......................................................................uaauacugucagguaaagaugucu.................    25    0    S06
.......................................................................aauacugucagguaaagauguc..................    2     0    S06

...........................caucuuaccaggcaguauuaga..............................................................    7     0    S01
......................................................................uaauacugucagguaaagaug....................    6     0    S01
......................................................................uaauacugucagguaaagaugu...................    4     0    S01
......................................................................uaauacugucagguaaagauguc..................    237   0    S01
......................................................................uaauacugucagguaaagaugucu.................    10    0    S01
.......................................................................aauacugucagguaaagauguc..................    1     0    S01
..........................................................................acugucagguaaagauguc..................    1     0    S01

...........................caucuuaccaggcaguauuag...............................................................    4     0    S10
...........................caucuuaccaggcaguauuaga..............................................................    524   0    S10
...........................caucuuaccaggcaguauuagagucaauuugaaaauguucuuc.........................................    1     0    S10
............................aucuuaccaggcaguauuaga..............................................................    1     0    S10
.............................ucuuaccaggcaguauuaga..............................................................    1     0    S10
.....................................................................cuaauacugucagguaaagauguc..................    1     0    S10
......................................................................uaauacugucagguaaag.......................    1     0    S10
......................................................................uaauacugucagguaaaga......................    3     0    S10
......................................................................uaauacugucagguaaagau.....................    1     0    S10
......................................................................uaauacugucagguaaagaug....................    17    0    S10
......................................................................uaauacugucagguaaagaugu...................    76    0    S10
```

Star **Mature**

```
augucuucaaggaaggaguaucgacgucaucuuaccaggcaguauuagagucaauuugaaaauguucuucuaauacugucagguaaagaugucuacgauguccucacgcau
......................................................................uaauacugucagguaaagauguc..................  5143  0  S10
......................................................................uaauacugucagguaaagaugucu.................  104   0  S10
.......................................................................aauacugucagguaaagauguc..................  12    0  S10
.......................................................................aauacugucagguaaagaugucu.................  1     0  S10
........................................................................auacugucagguaaagauguc..................  6     0  S10
..........................................................................acugucagguaaagauguc..................  1     0  S10
...........................................................................cugucagguaaagauguc..................  1     0  S10

...........................caucuuaccaggcaguauuaga..............................................................  29    0  S05
......................................................................uaauacugucagguaaagaug....................  16    0  S05
......................................................................uaauacugucagguaaagaugu...................  17    0  S05
......................................................................uaauacugucagguaaagauguc..................  654   0  S05
......................................................................uaauacugucagguaaagaugucu.................  26    0  S05
.......................................................................aauacugucagguaaagauguc..................  1     0  S05
...........................................................................cugucagguaaagauguc..................  1     0  S05
```

Provisional ID	:	Scaffold1824_30216
Score total	:	28189.4
Score for star read(s)	:	3.9
Score for read counts	:	28182.2
Score for mfe	:	1.7
Score for randfold	:	1.6
Score for cons. seed	:	
Total read count	:	55290
Mature read count	:	54417
Loop read count	:	0
Star read count	:	873

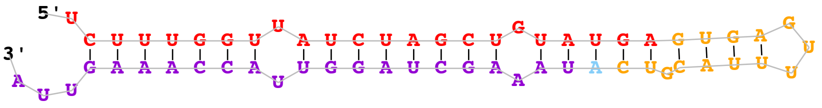

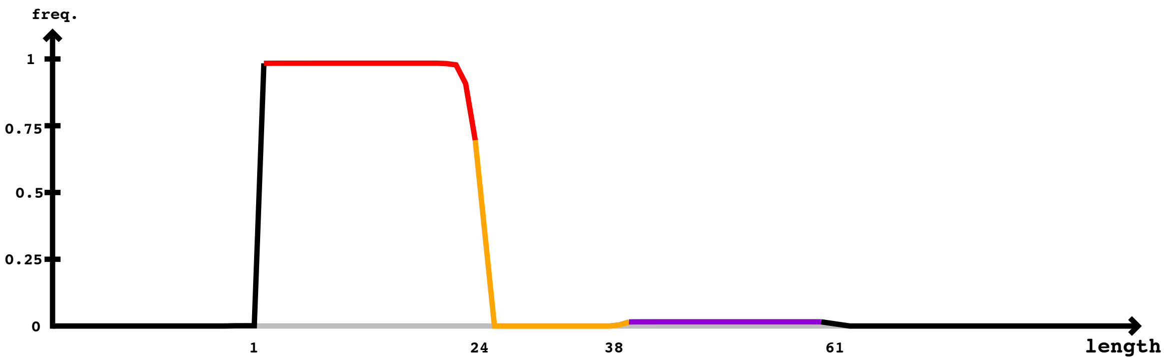

Mature **Star**

```
5'- guuguguaacguugguauuuucuuugguuaucuagcuguaugagugaguuuuacgucauaaagcuagguuaccaaaguuaagaacaacggcaugaaaaucuucaagagaug   -3'   obs
    guuguguaacguugguauuuucuuugguuaucuagcuguaugagugaguuuuacgucauaaagcuagguuaccaaaguuaagaacaacggcaugaaaaucuucaagagaug         exp
    .(..(((..(((((...((..(((((((.((((((((.(((((((((...)))).))))).)))))))).)))))))..))...))))))))..)..(((((...))))).    reads   mm   sample
    ....................ucuuugguuaucuagcuguau......................................................................    2       0    S01
    ....................ucuuugguuaucuagcuguaug.....................................................................    1       0    S01
    ....................ucuuugguuaucuagcuguauga....................................................................    2       0    S01

    .................uuuucuuugguuaucuagcugua.......................................................................    1       0    S06
    ....................ucuuugguuaucuagcugua.......................................................................    7       0    S06
    ....................ucuuugguuaucuagcuguau......................................................................    81      0    S06
    ....................ucuuugguuaucuagcuguaug.....................................................................    398     0    S06
    ....................ucuuugguuaucuagcuguauga....................................................................    1784    0    S06
    ....................ucuuugguuaucuagcuguaugag...................................................................    1       0    S06
    ....................ucuuugguuaucuagcuguaugagu..................................................................    1       0    S06
    ..........................................................uaaagcuagguuaccaaagu.................................    1       0    S06
    ..........................................................uaaagcuagguuaccaaaguu................................    3       0    S06
    ..........................................................uaaagcuagguuaccaaaguua...............................    3       0    S06

    .................uuuucuuugguuaucuagcuguau......................................................................    2       0    S07
    .................uuuucuuugguuaucuagcuguauga....................................................................    1       0    S07
    ....................ucuuugguuaucuagcugu........................................................................    3       0    S07
    ....................ucuuugguuaucuagcugua.......................................................................    10      0    S07
    ....................ucuuugguuaucuagcuguau......................................................................    205     0    S07
    ....................ucuuugguuaucuagcuguaug.....................................................................    514     0    S07
    ....................ucuuugguuaucuagcuguauga....................................................................    1836    0    S07
    ....................ucuuugguuaucuagcuguaugag...................................................................    1       0    S07
    ....................ucuuugguuaucuagcuguaugagu..................................................................    2       0    S07
    ....................ucuuugguuaucuagcuguaugagugaguuuuacguca.....................................................    1       0    S07
    .........................................................auaaagcuagguuaccaaaguu................................    1       0    S07
    .........................................................auaaagcuagguuaccaaaguua...............................    13      0    S07
    ..........................................................uaaagcuagguuaccaaaguu................................    4       0    S07
    ..........................................................uaaagcuagguuaccaaaguua...............................    33      0    S07

    .................uuuucuuugguuaucuagcugua.......................................................................    2       0    S05
    .................uuuucuuugguuaucuagcuguaug.....................................................................    1       0    S05
    ....................ucuuugguuaucuagcugu........................................................................    1       0    S05
```

```
                    Mature                                Star
guuguguaacguugguauuuucuuugguuaucuagcuguaugagugaguuuuacgucauaaagcuagguuaccaaaguuaagaacaacggcaugaaaaucuucaagagaug
....................ucuuugguuaucuagcugua.......................................................................   5       0   S05
....................ucuuugguuaucuagcuguau......................................................................   74      0   S05
....................ucuuugguuaucuagcuguaug.....................................................................   355     0   S05
....................ucuuugguuaucuagcuguauga....................................................................   1437    0   S05
....................ucuuugguuaucuagcuguaugag...................................................................   1       0   S05
.........................................................auaaagcuagguuaccaaaguu................................   1       0   S05
..........................................................uaaagcuagguuaccaaaguu................................   3       0   S05
..........................................................uaaagcuagguuaccaaaguua...............................   5       0   S05

................auuuucuuugguuaucuagcuguau......................................................................   2       0   S10
.................uuuucuuugguuaucuagcugua.......................................................................   10      0   S10
.................uuuucuuugguuaucuagcuguau......................................................................   8       0   S10
.................uuuucuuugguuaucuagcuguauga....................................................................   2       0   S10
..................uuucuuugguuaucuagcugua.......................................................................   2       0   S10
....................ucuuugguuaucuagcugu........................................................................   33      0   S10
....................ucuuugguuaucuagcugua.......................................................................   90      0   S10
....................ucuuugguuaucuagcuguau......................................................................   1814    0   S10
....................ucuuugguuaucuagcuguaug.....................................................................   5626    0   S10
....................ucuuugguuaucuagcuguauga....................................................................   14874   0   S10
....................ucuuugguuaucuagcuguaugag...................................................................   3       0   S10
....................ucuuugguuaucuagcuguaugagu..................................................................   1       0   S10
.....................cuuugguuaucuagcuguauga....................................................................   9       0   S10
......................uuugguuaucuagcuguaug.....................................................................   1       0   S10
......................uuugguuaucuagcuguauga....................................................................   1       0   S10
.......................uugguuaucuagcuguau......................................................................   1       0   S10
.......................uugguuaucuagcuguaug.....................................................................   1       0   S10
........................ugguuaucuagcuguauga....................................................................   1       0   S10
.........................gguuaucuagcuguauga....................................................................   1       0   S10
.........................................................auaaagcuagguuaccaaaguu................................   28      0   S10
.........................................................auaaagcuagguuaccaaaguua...............................   102     0   S10
..........................................................uaaagcuagguuaccaaagu.................................   2       0   S10
..........................................................uaaagcuagguuaccaaaguu................................   41      0   S10
..........................................................uaaagcuagguuaccaaaguua...............................   213     0   S10
...........................................................aaagcuagguuaccaaaguu................................   4       0   S10
...........................................................aaagcuagguuaccaaaguua...............................   5       0   S10
.............................................................agcuagguuaccaaaguu................................   1       0   S10
.............................................................agcuagguuaccaaaguua...............................   1       0   S10

.................uuuucuuugguuaucuagcuguau......................................................................   1       0   S02
....................ucuuugguuaucuagcugua.......................................................................   1       0   S02
....................ucuuugguuaucuagcuguaug.....................................................................   3       0   S02
....................ucuuugguuaucuagcuguauga....................................................................   37      0   S02
..........................................................uaaagcuagguuaccaaaguua...............................   1       0   S02

.uuguguaacguugguauuuucu........................................................................................   1       0   S04
.................uuuucuuugguuaucuagcuguau......................................................................   2       0   S04
....................ucuuugguuaucuagcug.........................................................................   1       0   S04
....................ucuuugguuaucuagcugu........................................................................   3       0   S04
....................ucuuugguuaucuagcugua.......................................................................   10      0   S04
....................ucuuugguuaucuagcuguau......................................................................   84      0   S04
....................ucuuugguuaucuagcuguaug.....................................................................   386     0   S04
....................ucuuugguuaucuagcuguauga....................................................................   1524    0   S04
.........................gguuaucuagcuguauga....................................................................   1       0   S04
.........................................................auaaagcuagguuaccaaaguu................................   1       0   S04
.........................................................auaaagcuagguuaccaaaguua...............................   1       0   S04
..........................................................uaaagcuagguuaccaaaguu................................   1       0   S04
..........................................................uaaagcuagguuaccaaaguua...............................   4       0   S04
...........................................................aaagcuagguuaccaaaguu................................   1       0   S04
...........................................................aaagcuagguuaccaaaguua...............................   1       0   S04
............................................................aagcuagguuaccaaaguu................................   1       0   S04

................auuuucuuugguuaucuagcuguau......................................................................   1       0   S03
....................ucuuugguuaucuagcugua.......................................................................   6       0   S03
....................ucuuugguuaucuagcuguau......................................................................   38      0   S03
....................ucuuugguuaucuagcuguaug.....................................................................   192     0   S03
....................ucuuugguuaucuagcuguauga....................................................................   776     0   S03
.........................................................auaaagcuagguuaccaaaguu................................   1       0   S03
.........................................................auaaagcuagguuaccaaaguua...............................   2       0   S03
..........................................................uaaagcuagguuaccaaaguu................................   2       0   S03
..........................................................uaaagcuagguuaccaaaguua...............................   6       0   S03
```

Mature **Star**

```
guuguguaacguugguauuuucuuugguuaucuagcuguaugagugaguuuuacgucauaaagcuagguuaccaaaguuaagaacaacggcaugaaaaucuucaagagaug

.................uuuucuuugguuaucuagcugua.......................................................................   10      0   S09
.................uuuucuuugguuaucuagcuguau......................................................................   5       0   S09
.................uuuucuuugguuaucuagcuguauga....................................................................   1       0   S09
..................uuucuuugguuaucuagcuguau......................................................................   1       0   S09
....................ucuuugguuaucuagcugu........................................................................   32      0   S09
....................ucuuugguuaucuagcugua.......................................................................   103     0   S09
....................ucuuugguuaucuagcuguau......................................................................   1393    0   S09
....................ucuuugguuaucuagcuguaug.....................................................................   3802    0   S09
....................ucuuugguuaucuagcuguauga....................................................................   13760   0   S09
....................ucuuugguuaucuagcuguaugag...................................................................   4       0   S09
.....................cuuugguuaucuagcuguaug.....................................................................   2       0   S09
.....................cuuugguuaucuagcuguauga....................................................................   6       0   S09
......................uuugguuaucuagcuguauga....................................................................   1       0   S09
.......................uugguuaucuagcuguauga....................................................................   1       0   S09
.........................gguuaucuagcuguauga....................................................................   1       0   S09
.........................................................auaaagcuagguuaccaaaguu................................   18      0   S09
.........................................................auaaagcuagguuaccaaaguua...............................   74      0   S09
..........................................................uaaagcuagguuaccaaaguu................................   38      0   S09
..........................................................uaaagcuagguuaccaaaguua...............................   201     0   S09
..........................................................uaaagcuagguuaccaaaguuaa..............................   5       0   S09
...........................................................aaagcuagguuaccaaaguu................................   4       0   S09
...........................................................aaagcuagguuaccaaaguua...............................   4       0   S09
............................................................aagcuagguuaccaaaguu................................   1       0   S09

.................uuuucuuugguuaucuagcugua.......................................................................   1       0   S08
.................uuuucuuugguuaucuagcuguauga....................................................................   1       0   S08
....................ucuuugguuaucuagcugu........................................................................   3       0   S08
....................ucuuugguuaucuagcugua.......................................................................   17      0   S08
....................ucuuugguuaucuagcuguau......................................................................   161     0   S08
....................ucuuugguuaucuagcuguaug.....................................................................   459     0   S08
....................ucuuugguuaucuagcuguauga....................................................................   2428    0   S08
.......................uugguuaucuagcuguauga....................................................................   1       0   S08
.........................gguuaucuagcuguauga....................................................................   1       0   S08
.........................................................auaaagcuagguuaccaaaguu................................   6       0   S08
.........................................................auaaagcuagguuaccaaaguua...............................   4       0   S08
..........................................................uaaagcuagguuaccaaaguu................................   13      0   S08
..........................................................uaaagcuagguuaccaaaguua...............................   19      0   S08
```

Provisional ID	:	Scaffold2609_36101
Score total	:	65499.5
Score for star read(s)	:	3.9
Score for read counts	:	65491.8
Score for mfe	:	2.2
Score for randfold	:	1.6
Score for cons. seed	:	
Total read count	:	128471
Mature read count	:	122681
Loop read count	:	0
Star read count	:	5790

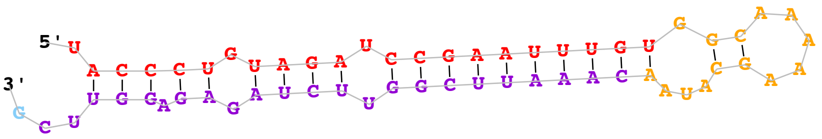

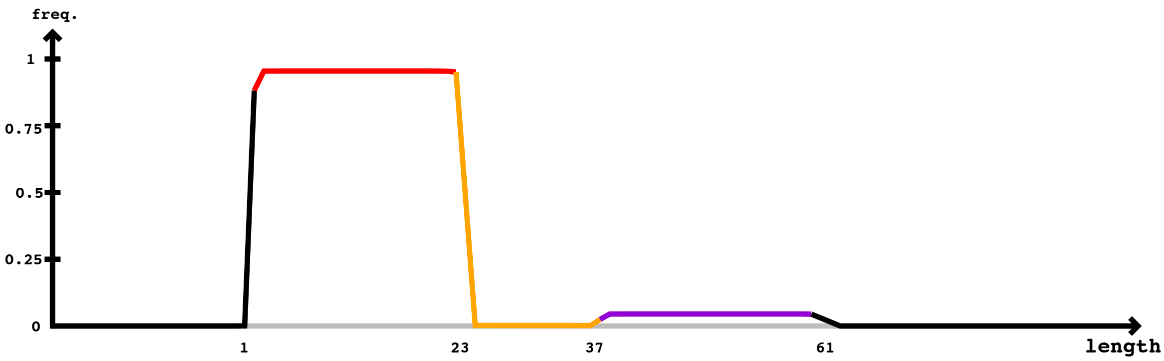

Mature **Star**

```
5'- aguuuuugugcucuacaucuacccuguagauccgaauuuguggcaaaaagcauaacaaauucgguucuagagagguucguguggggcacgagauacaaaagaaagauacag  -3'   obs
    aguuuuugugcucuacaucuacccuguagauccgaauuuguggcaaaaagcauaacaaauucgguucuagagagguucguguggggcacgagauacaaaagaaagauacag         exp
    .(((((((((((((((((..(((((.((((.(((((((((.((.....))...))))))))).)))).)).)))..))))))))))))))).)).................   reads   mm   sample
    .................ucuacccuguagauccgaauuugu......................................................................   2       0    S07
    ..................cuacccuguagauccgaauuugu......................................................................   3       0    S07
    ...................uacccuguagauccgaauu.........................................................................   3       0    S07
    ...................uacccuguagauccgaauuu........................................................................   24      0    S07
    ...................uacccuguagauccgaauuug.......................................................................   26      0    S07
    ...................uacccuguagauccgaauuugu......................................................................   7535    0    S07
    ...................uacccuguagauccgaauuugug.....................................................................   7       0    S07
    ...................uacccuguagauccgaauuuguggcaaa................................................................   1       0    S07
    ...................uacccuguagauccgaauuuguggcaaaaagcaua.........................................................   1       0    S07
    ...................uacccuguagauccgaauuuguggcaaaaagcauaa........................................................   3       0    S07
    ....................acccuguagauccgaauu.........................................................................   1       0    S07
    ....................acccuguagauccgaauuug.......................................................................   7       0    S07
    ....................acccuguagauccgaauuugu......................................................................   534     0    S07
    ....................acccuguagauccgaauuugug.....................................................................   139     0    S07
    ....................acccuguagauccgaauuuguggca..................................................................   1       0    S07
    ....................acccuguagauccgaauuuguggcaaaaagcau..........................................................   1       0    S07
    ....................acccuguagauccgaauuuguggcaaaaagcaua.........................................................   4       0    S07
    ....................acccuguagauccgaauuuguggcaaaaagcauaa........................................................   34      0    S07
    ....................acccuguagauccgaauuuguggcaaaaagcauaac.......................................................   4       0    S07
    ......................ccuguagauccgaauuugu......................................................................   2       0    S07
    .......................................................caaauucgguucuagagagguuc.................................   330     0    S07
    .......................................................caaauucgguucuagagagguucg................................   1       0    S07
    ........................................................aaauucgguucuagagaggu...................................   1       0    S07
    ........................................................aaauucgguucuagagagguuc.................................   140     0    S07
    ........................................................aaauucgguucuagagagguucg................................   70      0    S07
    ........................................................aaauucgguucuagagagguucgu...............................   9       0    S07
    .........................................................aauucgguucuagagagguucgu...............................   1       0    S07
    ..........................................................auucgguucuagagagguucgu...............................   1       0    S07

    ...................uacccuguagauccgaauuugu......................................................................   11      0    S01
    ...................uacccuguagauccgaauuugug.....................................................................   3       0    S01
    ....................acccuguagauccgaauu.........................................................................   1       0    S01
    ....................acccuguagauccgaauuu........................................................................   1       0    S01
```

Mature **Star**

```
aguuuuugugcucuacaucuacccuguagauccgaauuuguggcaaaaagcauaacaaauucgguucuagagagguucguguggggcacgagauacaaaagaaagauacag
....................acccuguagauccgaauuug.......................................................................	2	0	S01
....................acccuguagauccgaauuugu......................................................................	4	0	S01
.......................................................caaauucgguucuagagagguuc.................................	2	0	S01

...................uacccuguagauccgaauuug.......................................................................	1	0	S06
...................uacccuguagauccgaauuugu......................................................................	355	0	S06
...................uacccuguagauccgaauuugug.....................................................................	10	0	S06
....................acccuguagauccgaauu.........................................................................	1	0	S06
....................acccuguagauccgaauuugu......................................................................	25	0	S06
....................acccuguagauccgaauuugug.....................................................................	19	0	S06
....................acccuguagauccgaauuuguggcaaaaagcauaa........................................................	3	0	S06
.......................................................caaauucgguucuagagagguuc.................................	47	0	S06
........................................................aaauucgguucuagagagguuc.................................	22	0	S06
........................................................aaauucgguucuagagagguucg................................	5	0	S06

aguuuuugugcucuacaucu...........................................................................................	1	0	S10
.................ucuacccuguagauccgaauuugu......................................................................	2	0	S10
..................cuacccuguagauccgaauuugu......................................................................	19	0	S10
...................uacccuguagauccgaau..........................................................................	1	0	S10
...................uacccuguagauccgaauu.........................................................................	4	0	S10
...................uacccuguagauccgaauuu........................................................................	16	0	S10
...................uacccuguagauccgaauuug.......................................................................	115	0	S10
...................uacccuguagauccgaauuugu......................................................................	42791	0	S10
...................uacccuguagauccgaauuugug.....................................................................	21	0	S10
...................uacccuguagauccgaauuuguggcaaaaagcauaac.......................................................	2	0	S10
....................acccuguagauccgaauu.........................................................................	3	0	S10
....................acccuguagauccgaauuu........................................................................	2	0	S10
....................acccuguagauccgaauuug.......................................................................	26	0	S10
....................acccuguagauccgaauuugu......................................................................	2642	0	S10
....................acccuguagauccgaauuugug.....................................................................	610	0	S10
....................acccuguagauccgaauuuguggc...................................................................	2	0	S10
....................acccuguagauccgaauuuguggca..................................................................	2	0	S10
....................acccuguagauccgaauuuguggcaaaaagc............................................................	1	0	S10
....................acccuguagauccgaauuuguggcaaaaagca...........................................................	1	0	S10
....................acccuguagauccgaauuuguggcaaaaagcaua.........................................................	2	0	S10
....................acccuguagauccgaauuuguggcaaaaagcauaa........................................................	71	0	S10
....................acccuguagauccgaauuuguggcaaaaagcauaac.......................................................	2	0	S10
.....................cccuguagauccgaauuugu......................................................................	1	0	S10
......................ccuguagauccgaauuugu......................................................................	6	0	S10
......................ccuguagauccgaauuugug.....................................................................	1	0	S10
.......................cuguagauccgaauuugu......................................................................	1	0	S10
........................uguagauccgaauuugug.....................................................................	1	0	S10
.......................................................caaauucgguucuagagagguu..................................	27	0	S10
.......................................................caaauucgguucuagagagguuc.................................	1150	0	S10
.......................................................caaauucgguucuagagagguucg................................	29	0	S10
.......................................................caaauucgguucuagagagguucgu...............................	2	0	S10
........................................................aaauucgguucuagagagguu..................................	12	0	S10
........................................................aaauucgguucuagagagguuc.................................	505	0	S10
........................................................aaauucgguucuagagagguucg................................	615	0	S10
........................................................aaauucgguucuagagagguucgu...............................	27	0	S10
........................................................aaauucgguucuagagagguucgugu.............................	1	0	S10
.........................................................aauucgguucuagagagguuc.................................	1	0	S10
.........................................................aauucgguucuagagagguucgu...............................	2	0	S10
............................................................ucgguucuagagagguuc.................................	1	0	S10
.............................................................cgguucuagagagguucg................................	1	0	S10

...................uacccuguagauccgaauuugu......................................................................	7	0	S05
...................uacccuguagauccgaauuugug.....................................................................	1	0	S05
....................acccuguagauccgaauuuguggcaaaag..............................................................	1	0	S05
.......................................................caaauucgguucuagagagguuc.................................	1	0	S05

...................uacccuguagauccgaauuugu......................................................................	21	0	S02
...................uacccuguagauccgaauuugug.....................................................................	4	0	S02
....................acccuguagauccgaauu.........................................................................	1	0	S02
....................acccuguagauccgaauuugu......................................................................	4	0	S02
........................................................aaauucgguucuagagagguuc.................................	1	0	S02

...................uacccuguagauccgaauuugu......................................................................	20	0	S04
...................uacccuguagauccgaauuugug.....................................................................	2	0	S04
....................acccuguagauccgaauuug.......................................................................	1	0	S04
```

```
                   Mature                             Star
aguuuuugugcucuacaucuacccuguagauccgaauuuguggcaaaaagcauaacaaauucgguucuagagagguucguguggggcacgagauacaaaagaaagauacag
....................acccuguagauccgaauuugu......................................................................   2      0   S04
.......................................................caaauucgguucuagagagguuc.................................   2      0   S04

..................cuacccuguagauccgaauuugu......................................................................   4      0   S08
...................uacccuguagauccgaau..........................................................................   3      0   S08
...................uacccuguagauccgaauu.........................................................................   4      0   S08
...................uacccuguagauccgaauuu........................................................................   3      0   S08
...................uacccuguagauccgaauuug.......................................................................   26     0   S08
...................uacccuguagauccgaauuugu......................................................................   6531   0   S08
...................uacccuguagauccgaauuugug.....................................................................   10     0   S08
...................uacccuguagauccgaauuuguggcaaaaagcauaa........................................................   1      0   S08
....................acccuguagauccgaauu.........................................................................   1      0   S08
....................acccuguagauccgaauuu........................................................................   1      0   S08
....................acccuguagauccgaauuug.......................................................................   20     0   S08
....................acccuguagauccgaauuugu......................................................................   770    0   S08
....................acccuguagauccgaauuugug.....................................................................   496    0   S08
....................acccuguagauccgaauuugugg....................................................................   1      0   S08
....................acccuguagauccgaauuuguggc...................................................................   3      0   S08
....................acccuguagauccgaauuuguggca..................................................................   1      0   S08
....................acccuguagauccgaauuuguggcaa.................................................................   3      0   S08
....................acccuguagauccgaauuuguggcaaa................................................................   5      0   S08
....................acccuguagauccgaauuuguggcaaaa...............................................................   1      0   S08
....................acccuguagauccgaauuuguggcaaaaa..............................................................   1      0   S08
....................acccuguagauccgaauuuguggcaaaaagc............................................................   1      0   S08
....................acccuguagauccgaauuuguggcaaaaagca...........................................................   1      0   S08
....................acccuguagauccgaauuuguggcaaaaagcau..........................................................   2      0   S08
....................acccuguagauccgaauuuguggcaaaaagcaua.........................................................   6      0   S08
....................acccuguagauccgaauuuguggcaaaaagcauaa........................................................   17     0   S08
....................acccuguagauccgaauuuguggcaaaaagcauaac.......................................................   5      0   S08
.....................cccuguagauccgaauu.........................................................................   2      0   S08
.....................cccuguagauccgaauuug.......................................................................   1      0   S08
.....................cccuguagauccgaauuugu......................................................................   3      0   S08
......................ccuguagauccgaauuugu......................................................................   4      0   S08
.......................cuguagauccgaauuugu......................................................................   2      0   S08
.......................................................caaauucgguucuagagagguu..................................   5      0   S08
.......................................................caaauucgguucuagagagguuc.................................   562    0   S08
.......................................................caaauucgguucuagagagguucg................................   2      0   S08
.......................................................caaauucgguucuagagagguucgu...............................   1      0   S08
........................................................aaauucgguucuagagagguu..................................   5      0   S08
........................................................aaauucgguucuagagagguuc.................................   170    0   S08
........................................................aaauucgguucuagagagguucg................................   45     0   S08
........................................................aaauucgguucuagagagguucgu...............................   4      0   S08

...................uacccuguagauccgaauuugu......................................................................   24     0   S03
...................uacccuguagauccgaauuugug.....................................................................   4      0   S03
....................acccuguagauccgaauuugu......................................................................   1      0   S03
.......................................................caaauucgguucuagagagguuc.................................   2      0   S03
........................................................aaauucgguucuagagagguuc.................................   1      0   S03

.................ucuacccuguagauccgaauuugu......................................................................   6      0   S09
..................cuacccuguagauccgaauuug.......................................................................   1      0   S09
..................cuacccuguagauccgaauuugu......................................................................   27     0   S09
...................uacccuguagauccgaau..........................................................................   4      0   S09
...................uacccuguagauccgaauu.........................................................................   8      0   S09
...................uacccuguagauccgaauuu........................................................................   19     0   S09
...................uacccuguagauccgaauuug.......................................................................   137    0   S09
...................uacccuguagauccgaauuugu......................................................................   55683  0   S09
...................uacccuguagauccgaauuugug.....................................................................   26     0   S09
...................uacccuguagauccgaauuuguggc...................................................................   1      0   S09
...................uacccuguagauccgaauuuguggcaa.................................................................   1      0   S09
...................uacccuguagauccgaauuuguggcaaaaagc............................................................   1      0   S09
...................uacccuguagauccgaauuuguggcaaaaagcaua.........................................................   1      0   S09
...................uacccuguagauccgaauuuguggcaaaaagcauaa........................................................   1      0   S09
...................uacccuguagauccgaauuuguggcaaaaagcauaac.......................................................   1      0   S09
....................acccuguagauccgaauu.........................................................................   1      0   S09
....................acccuguagauccgaauuu........................................................................   1      0   S09
....................acccuguagauccgaauuug.......................................................................   24     0   S09
....................acccuguagauccgaauuugu......................................................................   3169   0   S09
....................acccuguagauccgaauuugug.....................................................................   594    0   S09
....................acccuguagauccgaauuuguggca..................................................................   2      0   S09
```

```
                    Mature                                  Star
aguuuuugugcucuacaucuacccuguagauccgaauuuguggcaaaaagcauaacaaauucgguucuagagagguucguguggggcacgagauacaaaagaaagauacag
....................acccuguagauccgaauuuguggcaaaaagc............................................................   2    0   S09
....................acccuguagauccgaauuuguggcaaaaagcau..........................................................   1    0   S09
....................acccuguagauccgaauuuguggcaaaaagcaua.........................................................   5    0   S09
....................acccuguagauccgaauuuguggcaaaaagcauaa........................................................   64   0   S09
....................acccuguagauccgaauuuguggcaaaaagcauaac.......................................................   9    0   S09
.....................cccuguagauccgaauuugu......................................................................   5    0   S09
......................ccuguagauccgaauuugu......................................................................   7    0   S09
.......................cuguagauccgaauuugu......................................................................   1    0   S09
.......................................................caaauucgguucuagagagguu..................................   13   0   S09
.......................................................caaauucgguucuagagagguuc.................................   977  0   S09
.......................................................caaauucgguucuagagagguucg................................   10   0   S09
.......................................................caaauucgguucuagagagguucgu...............................   3    0   S09
........................................................aaauucgguucuagagagguu..................................   9    0   S09
........................................................aaauucgguucuagagagguuc.................................   519  0   S09
........................................................aaauucgguucuagagagguucg................................   424  0   S09
........................................................aaauucgguucuagagagguucgu...............................   23   0   S09
.........................................................aauucgguucuagagagguuc.................................   1    0   S09
.........................................................aauucgguucuagagagguucgu...............................   7    0   S09
..........................................................auucgguucuagagagguucgu...............................   1    0   S09
```

Provisional ID	:	Scaffold395_9965
Score total	:	322.7
Score for star read(s)	:	3.9
Score for read counts	:	315.7
Score for mfe	:	1.5
Score for randfold	:	1.6
Score for cons. seed	:	
Total read count	:	631
Mature read count	:	573
Loop read count	:	0
Star read count	:	58

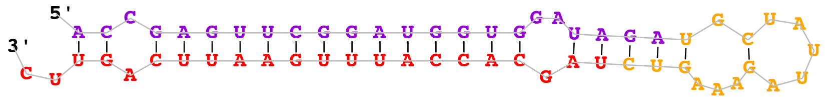

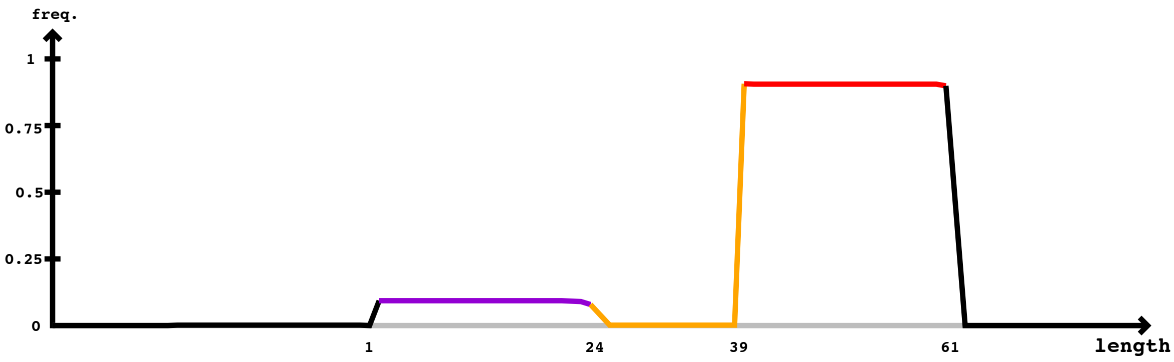

Star **Mature**

```
5'- ccuaaagaaagaucucguuuagucuuugggcuaccgaguucggaugguggauagaugcuauuagaaagucuagcaccauuugaauucaguucauccaaagagaauggcugcu  -3'   obs
    ccuaaagaaagaucucguuuagucuuugggcuaccgaguucggaugguggauagaugcuauuagaaagucuagcaccauuugaauucaguucauccaaagagaauggcugcu         exp
    ........((...((((((..((((((((..((.((((((((((((((..(((((.(.....)...))))).)))))))))))))).))...))))))))))))))...))   reads   mm   sample
    ................................accgaguucggaugguggauag..........................................................   1       0    S08
    ................................accgaguucggaugguggauaga.........................................................   8       0    S08
    ................................accgaguucggaugguggauagaugcuauuagaaagucu.........................................   1       0    S08
    .....................................................................uagcaccauuugaauucaguuc....................   40      0    S08

    ................................accgaguucggaugguggaua...........................................................   1       0    S09
    ................................accgaguucggaugguggauag..........................................................   2       0    S09
    ................................accgaguucggaugguggauaga.........................................................   11      0    S09
    .....................................................................uagcaccauuugaauucaguuc....................   187     0    S09
    .....................................................................uagcaccauuugaauucaguuca...................   1       0    S09

    ...........aucucguuuagucuuugggc................................................................................   1       0    S03
    ................................accgaguucggaugguggauaga.........................................................   3       0    S03
    .....................................................................uagcaccauuugaauucaguuc....................   8       0    S03

    .....................................................................uagcaccauuugaauucaguuc....................   18      0    S04

    .....................................................................uagcaccauuugaauucaguuc....................   17      0    S02
    .....................................................................uagcaccauuugaauucaguuca...................   1       0    S02

    ................................accgaguucggaugguggauag..........................................................   2       0    S10
    ................................accgaguucggaugguggauaga.........................................................   12      0    S10
    .....................................................................uagcaccauuugaauucaguu.....................   2       0    S10
    .....................................................................uagcaccauuugaauucaguuc....................   238     0    S10
    .....................................................................uagcaccauuugaauucaguuca...................   1       0    S10

    ................................accgaguucggaugguggau............................................................   1       0    S05
    ................................accgaguucggaugguggauag..........................................................   1       0    S05
    .....................................................................uagcaccauuugaauucaguuc....................   16      0    S05

    ................................accgaguucggaugguggauaga.........................................................   8       0    S07
    .....................................................................uagcaccauuugaauucaguuc....................   24      0    S07

    ................................accgaguucggaugguggauag..........................................................   1       0    S06
```

```
                                Star                                 Mature
ccuaaagaaagaucucguuuagucuuugggcuaccgaguucggaugguggauagaugcuauuagaaagucuagcaccauuugaauucaguucauccaaagagaauggcugcu
................................accgaguucggaugguggauaga.........................................................    7     0     S06
......................................................................uagcaccauuugaauucaguu.....................    2     0     S06
......................................................................uagcaccauuugaauucaguuc....................    12    0     S06

......................................................................uagcaccauuugaauucaguuc....................    6     0     S01
```

Provisional ID : Scaffold395_9963
Score total : 101.4
Score for star read(s) : 3.9
Score for read counts : 94.4
Score for mfe : 1.5
Score for randfold : 1.6
Score for cons. seed :
Total read count : 197
Mature read count : 185
Loop read count : 0
Star read count : 12

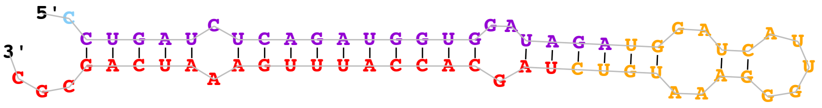

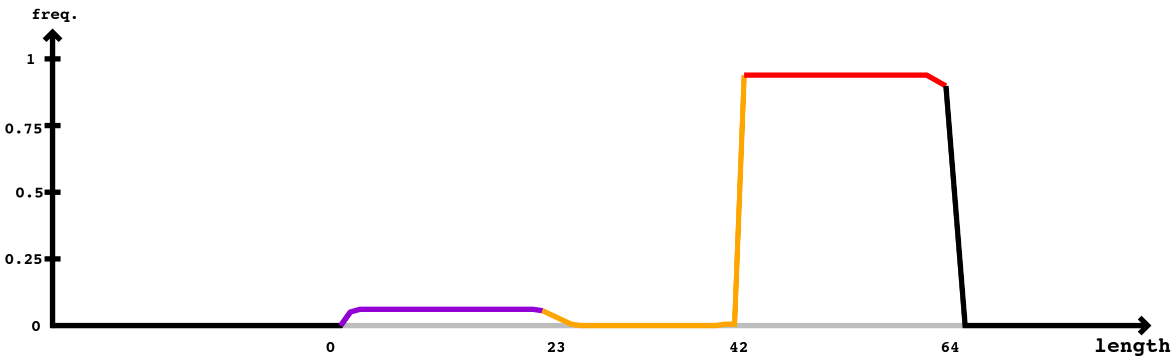

Star Mature

```
5'- gaagaacucuguaccgcacuuccugaaaccugaucucagaugguggauagauggaucauugggaaaugucuagcaccauuugaaaucagcgcuucugggagcuucccgacau  -3'   obs
    gaagaacucuguaccgcacuuccugaaaccugaucucagaugguggauagauggaucauugggaaaugucuagcaccauuugaaaucagcgcuucugggagcuucccgacau         exp
    .........(((......(((((.(((.((((((.((((((((((..((((((..((.....))..)))))).)))))))))).))))).).))).))))).......))).  reads   mm   sample
    ......................................................................uagcaccauuugaaaucagcgc....................  2       0    S04

    ......................................................................uagcaccauuugaaaucagcgc....................  3       0    S02

    .............................cugaucucagaugguggauaga.............................................................  1       0    S09
    ..............................ugaucucagaugguggauaga.............................................................  1       0    S09
    ..............................ugaucucagaugguggauagaug...........................................................  1       0    S09
    ......................................................................uagcaccauuugaaaucagc......................  1       0    S09
    ......................................................................uagcaccauuugaaaucagcg.....................  3       0    S09
    ......................................................................uagcaccauuugaaaucagcgc....................  60      0    S09
    ......................................................................uagcaccauuugaaaucagcgcu...................  14      0    S09

    ......................................................................uagcaccauuugaaaucagcgc....................  3       0    S03

    .............................cugaucucagaugguggauag..............................................................  1       0    S08
    .............................cugaucucagaugguggauaga.............................................................  2       0    S08
    ......................................................................uagcaccauuugaaaucagcgc....................  16      0    S08

    ......................................................................uagcaccauuugaaaucagcgc....................  1       0    S06
    ......................................................................uagcaccauuugaaaucagcgcu...................  1       0    S06

    .............................cugaucucagaugguggauaga.............................................................  1       0    S07
    ......................................................................uagcaccauuugaaaucagc......................  1       0    S07
    ......................................................................uagcaccauuugaaaucagcgc....................  8       0    S07
    ......................................................................uagcaccauuugaaaucagcgcu...................  1       0    S07

    ......................................................................uagcaccauuugaaaucagcgc....................  1       0    S05
    ......................................................................uagcaccauuugaaaucagcgcu...................  2       0    S05

    .............................cugaucucagaugguggaua...............................................................  1       0    S10
    .............................cugaucucagaugguggauag..............................................................  1       0    S10
    .............................cugaucucagaugguggauaga.............................................................  3       0    S10
    ....................................................................ucuagcaccauuugaaaucagc......................  1       0    S10
```

```
                                Star                                         Mature
gaagaacucuguaccgcacuuccugaaaccugaucucagaugguggauagauggaucauugggaaaugucuagcaccauuugaaaucagcgcuucugggagcuucccgacau
......................................................................uagcaccauuugaaaucagc......................    1    0    S10
......................................................................uagcaccauuugaaaucagcg.....................    1    0    S10
......................................................................uagcaccauuugaaaucagcgc....................    55   0    S10
......................................................................uagcaccauuugaaaucagcgcu...................    10   0    S10
```

Provisional ID : Scaffold633_15231
Score total : 306.3
Score for star read(s) : 3.9
Score for read counts : 299.4
Score for mfe : 1.4
Score for randfold : 1.6
Score for cons. seed :
Total read count : 599
Mature read count : 444
Loop read count : 0
Star read count : 155

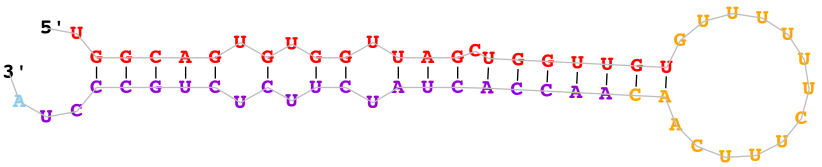

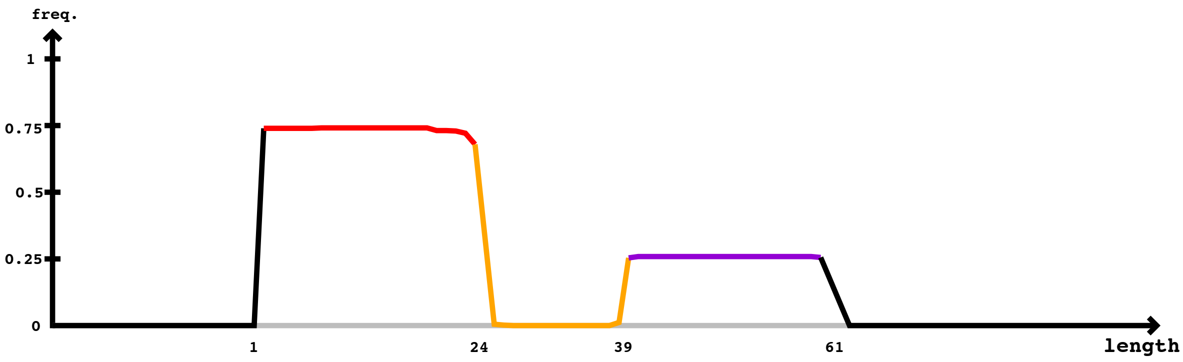

Mature   Star

```
5'- ggauguuuugugacugauucuggcagugugguuagcugguugugguuuuuucuuucaacaaccacuaucuucucugcccuaagcagaucacuuguugucauccacucauaccuu   -3'   obs
    ggauguuuugugacugauucuggcagugugguuagcugguugugguuuuuucuuucaacaaccacuaucuucucugcccuaagcagaucacuuguugucauccacucauaccuu         exp
    ((((....((((((.....(((((.(.((.(((.(((((((.............))))))))).)).).)))))).....))).)))).......)))))..........          reads   mm   sample
    ....................uggcagugugguuagcug...........................................................................   2   0   S01
    ....................uggcagugugguuagcugguug.......................................................................   1   0   S01
    ....................uggcagugugguuagcugguugu......................................................................   3   0   S01
    ....................uggcagugugguuagcugguugug.....................................................................   1   0   S01
    ..........................ugugguuagcugguugug.....................................................................   1   0   S01
    ..........................................................aaccacuaucuucucugcccu..................................   4   0   S01

    ....................uggcagugugguuagcug...........................................................................   3   0   S06
    ....................uggcagugugguuagcuggu.........................................................................   1   0   S06
    ....................uggcagugugguuagcugguu........................................................................   1   0   S06
    ....................uggcagugugguuagcugguug.......................................................................   2   0   S06
    ....................uggcagugugguuagcugguugu......................................................................   15  0   S06
    ....................uggcagugugguuagcugguuguC.....................................................................   3   1   S06
    ....................uggcagugugguuagcugguugug.....................................................................   1   0   S06
    .........................................................caaccacuaucuucucugcccu..................................   1   0   S06
    ..........................................................aaccacuaucuucucugccc...................................   1   0   S06
    ..........................................................aaccacuaucuucucugcccu..................................   11  0   S06
    ..........................................................aaccacuaucuucucugcccua.................................   2   0   S06
    ...........................................................accacuaucuucucugcccu..................................   1   0   S06
    ...........................................................accacuaucuucucugcccua.................................   1   0   S06

    ....................uggcagugugguuagcugguugu......................................................................   70  0   S07
    ....................uggcagugugguuagcugguuguC.....................................................................   3   1   S07
    .........................................................caaccacuaucuucucugcccu..................................   2   0   S07
    ..........................................................aaccacuaucuucucugcccu..................................   50  0   S07
    ..........................................................aaccacuaucuucucugcccua.................................   20  0   S07

    ....................uggcagugugguuagcugguugu......................................................................   20  0   S05
    .........................................................caaccacuaucuucucugcccu..................................   1   0   S05
    ..........................................................aaccacuaucuucucugcccu..................................   4   0   S05

    ....................uggcagugugguuagcugguu........................................................................   1   0   S10
    ....................uggcagugugguuagcugguug.......................................................................   11  0   S10
```

```
                    Mature                                  Star
ggauguuuugugacugauucuggcagugugguuagcugguuguguuuuuucuuucaacaaccacuaucuucucugcccuaagcagaucacuuguugucauccacucauaccuu
....................uggcagugugguuagcugguugu......................................................................    133    0    S10
....................uggcagugugguuagcugguuguC.....................................................................    2      1    S10
....................uggcagugugguuagcugguugugu....................................................................    2      0    S10
.........................................................aaccacuaucuucucugcccu...................................    13     0    S10
.........................................................aaccacuaucuucucugcccua..................................    5      0    S10

....................uggcagugugguuagcugguu........................................................................    2      0    S04
....................uggcagugugguuagcugguug.......................................................................    1      0    S04
....................uggcagugugguuagcugguugu......................................................................    7      0    S04
....................uggcagugugguuagcugguuguC.....................................................................    2      1    S04
.........................................................aaccacuaucuucucugcccua..................................    2      0    S04

....................uggcagugugguuagcug...........................................................................    1      0    S02
....................uggcagugugguuagcugguug.......................................................................    1      0    S02
....................uggcagugugguuagcugguugu......................................................................    3      0    S02

....................uggcagugugguuagcugguug.......................................................................    1      0    S03
....................uggcagugugguuagcugguugu......................................................................    19     0    S03
....................uggcagugugguuagcugguuguguu...................................................................    1      0    S03

....................uggcagugugguuagcugguu........................................................................    1      0    S09
....................uggcagugugguuagcugguug.......................................................................    8      0    S09
....................uggcagugugguuagcugguugu......................................................................    89     0    S09
.........................................................aaccacuaucuucucugcccu...................................    11     0    S09
.........................................................aaccacuaucuucucugcccua..................................    5      0    S09

....................uggcagugugguuagcugguugu......................................................................    32     0    S08
........................................................caaccacuaucuucucugcccu...................................    3      0    S08
.........................................................aaccacuaucuucucugccc....................................    1      0    S08
.........................................................aaccacuaucuucucugcccu...................................    11     0    S08
.........................................................aaccacuaucuucucugcccua..................................    5      0    S08
..........................................................accacuaucuucucugcccua..................................    1      0    S08
```

Provisional ID	:	Scaffold248_6750
Score total	:	328353.2
Score for star read(s)	:	3.9
Score for read counts	:	328346.4
Score for mfe	:	1.4
Score for randfold	:	1.6
Score for cons. seed	:	
Total read count	:	644049
Mature read count	:	642692
Loop read count	:	0
Star read count	:	1357

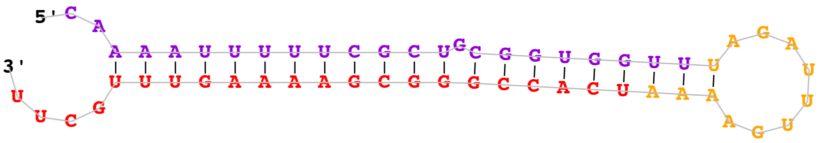

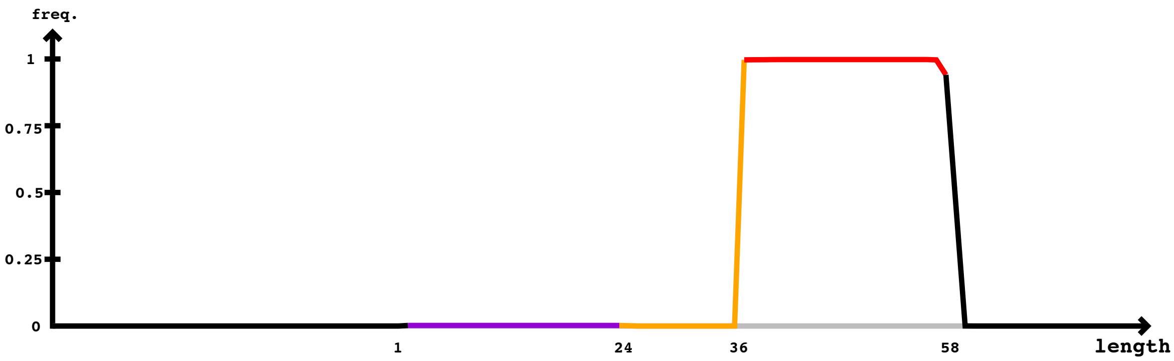

Star **Mature**

```
5'- uguuauguuucaucucuguuuuuuucuuuacgggacaaaauuuuucgcugcgguggguuuagauuugaaaaucaccgggcgaaaaguuugcuuuugugaugaaauaaaucugu   -3'    obs
    uguuauguuucaucucuguuuuuuucuuuacgggacaaaauuuuucgcugcgguggguuuagauuugaaaaucaccgggcgaaaaguuugcuuuugugaugaaauaaaucugu          exp
    .....(((((((((.((((..........))))....((((((((((((.(((((((((........)))))))))))))))))))))........))))))))).......    reads    mm    sample
    ...................................caaaauuuuucgcugcggugg........................................................    1        0     S10
    ...................................caaaauuuuucgcugcggugggu.......................................................    5        0     S10
    ...................................caaaauuuuucgcugcgguggguu......................................................    76       0     S10
    ...................................caaaauuuuucgcugcgguggguuu.....................................................    2        0     S10
    .........................................................uuagauuugaaaaucaccgggc.................................    1        0     S10
    ......................................................................ucaccgggcgaaaaguuu........................    2        0     S10
    ......................................................................ucaccgggcgaaaaguuug.......................    1        0     S10
    ......................................................................ucaccgggcgaaaaguuugc......................    68       0     S10
    ......................................................................ucaccgggcgaaaaguuugcu.....................    2792     0     S10
    ......................................................................ucaccgggcgaaaaguuugcuu....................    28836    0     S10
    ......................................................................ucaccgggcgaaaaguuugcuuu...................    557      0     S10
    ......................................................................ucaccgggcgaaaaguuugcuuuu..................    21       0     S10
    .......................................................................caccgggcgaaaaguuugcuu....................    16       0     S10
    .......................................................................caccgggcgaaaaguuugcuuu...................    1        0     S10
    ........................................................................accgggcgaaaaguuugcuu....................    4        0     S10
    .........................................................................ccgggcgaaaaguuugcu.....................    1        0     S10
    .........................................................................ccgggcgaaaaguuugcuu....................    8        0     S10
    .........................................................................ccgggcgaaaaguuugcuuu...................    1        0     S10
    ..........................................................................cgggcgaaaaguuugcuu....................    8        0     S10

    .......uuucaucucuguuuuuuucuuuacggga.............................................................................    1        0     S05
    ..........caucucuguuuuuuucuuuacggga.............................................................................    1        0     S05
    .............cucuguuuuuuucuuuacggga.............................................................................    1        0     S05
    ..............ucuguuuuuuucuuuacggga.............................................................................    4        0     S05
    ...............cuguuuuuuucuuuacggga.............................................................................    2        0     S05
    ................uguuuuuuucuuuacggga.............................................................................    2        0     S05
    ...................................caaaauuuuucgcugcgg...........................................................    1        0     S05
    ...................................caaaauuuuucgcugcggugggu.......................................................    6        0     S05
    ...................................caaaauuuuucgcugcgguggguu......................................................    169      0     S05
    ...................................caaaauuuuucgcugcgguggguuu.....................................................    7        0     S05
    ...................................caaaauuuuucgcugcgguggguuuagauuu...............................................    1        0     S05
    ....................................aaaauuuuucgcugcgguggguu......................................................    3        0     S05
    ....................................aaaauuuuucgcugcgguggguuuagauuugaaaa..........................................    2        0     S05
```

```
                                   Star                              Mature
uguuauguuucaucucuguuuuuuucuuuacgggacaaaauuuuucgcugcggugguuuagauuugaaaaucaccgggcgaaaaguuugcuuuugugaugaaauaaaucugu
.....................................aaauuuuucgcugcggugguu......................................................  1      0  S05
......................................aauuuuucgcugcggugguu......................................................  1      0  S05
........................................uuuuucgcugcggugguu......................................................  2      0  S05
........................................................uuuagauuugaaaaucaccgggcgaaaaguuugcuuu...................  1      0  S05
..........................................................uagauuugaaaaucaccgggcgaaaaguuugcuu....................  1      0  S05
................................................................ugaaaaucaccgggcgaaaaguuugcuu....................  1      0  S05
..................................................................aaaaucaccgggcgaaaaguuugcuu....................  1      0  S05
...................................................................aaaucaccgggcgaaaaguuugcu.....................  1      0  S05
...................................................................aaaucaccgggcgaaaaguuugcuu....................  2      0  S05
....................................................................aaucaccgggcgaaaaguuugcuu....................  2      0  S05
.....................................................................aucaccgggcgaaaaguuugcu.....................  1      0  S05
......................................................................ucaccgggcgaaaaguuu........................  5      0  S05
......................................................................ucaccgggcgaaaaguuug.......................  1      0  S05
......................................................................ucaccgggcgaaaaguuugc......................  97     0  S05
......................................................................ucaccgggcgaaaaguuugcu.....................  3360   0  S05
......................................................................ucaccgggcgaaaaguuugcuu....................  81583  0  S05
......................................................................ucaccgggcgaaaaguuugcuuu...................  1303   0  S05
......................................................................ucaccgggcgaaaaguuugcuuuu..................  15     0  S05
.......................................................................caccgggcgaaaaguuugcuu....................  39     0  S05
........................................................................accgggcgaaaaguuugcuu....................  9      0  S05
........................................................................accgggcgaaaaguuugcuuuu..................  1      0  S05
.........................................................................ccgggcgaaaaguuugcu.....................  2      0  S05
.........................................................................ccgggcgaaaaguuugcuu....................  35     0  S05
.........................................................................ccgggcgaaaaguuugcuuu...................  1      0  S05
..........................................................................cgggcgaaaaguuugcuu....................  30     0  S05
..........................................................................cgggcgaaaaguuugcuuu...................  2      0  S05

.......uuucaucucuguuuuuuucuuuacggga.............................................................................  1      0  S07
...................................caaaauuuuucgcugcgguggu.......................................................  6      0  S07
...................................caaaauuuuucgcugcggugguu......................................................  236    0  S07
...................................caaaauuuuucgcugcggugguuu.....................................................  3      0  S07
...................................caaaauuuuucgcugcggugguuuagau.................................................  1      0  S07
....................................aaaauuuuucgcugcggugguu......................................................  2      0  S07
......................................aauuuuucgcugcggugguu......................................................  2      0  S07
.......................................auuuuucgcugcggugguu......................................................  3      0  S07
........................................uuuuucgcugcggugguu......................................................  1      0  S07
.........................................................uuagauuugaaaaucaccgggc.................................  1      0  S07
...................................................................aaaucaccgggcgaaaaguuugcuuu...................  1      0  S07
....................................................................aaucaccgggcgaaaaguuugcuu....................  1      0  S07
......................................................................ucaccgggcgaaaaguuu........................  9      0  S07
......................................................................ucaccgggcgaaaaguuugc......................  121    0  S07
......................................................................ucaccgggcgaaaaguuugcu.....................  5330   0  S07
......................................................................ucaccgggcgaaaaguuugcuu....................  78186  0  S07
......................................................................ucaccgggcgaaaaguuugcuuu...................  1441   0  S07
......................................................................ucaccgggcgaaaaguuugcuuuu..................  21     0  S07
.......................................................................caccgggcgaaaaguuugcu.....................  1      0  S07
.......................................................................caccgggcgaaaaguuugcuu....................  17     0  S07
.......................................................................caccgggcgaaaaguuugcuuu...................  1      0  S07
........................................................................accgggcgaaaaguuugcu.....................  1      0  S07
........................................................................accgggcgaaaaguuugcuu....................  13     0  S07
........................................................................accgggcgaaaaguuugcuuu...................  1      0  S07
.........................................................................ccgggcgaaaaguuugcu.....................  2      0  S07
.........................................................................ccgggcgaaaaguuugcuu....................  27     0  S07
..........................................................................cgggcgaaaaguuugcuu....................  14     0  S07

...................................caaaauuuuucgcugcggug.........................................................  1      0  S01
...................................caaaauuuuucgcugcggugguu......................................................  4      0  S01
......................................................................ucaccgggcgaaaaguuu........................  2      0  S01
......................................................................ucaccgggcgaaaaguuugc......................  8      0  S01
......................................................................ucaccgggcgaaaaguuugcu.....................  220    0  S01
......................................................................ucaccgggcgaaaaguuugcuu....................  6680   0  S01
......................................................................ucaccgggcgaaaaguuugcuuu...................  9      0  S01
.......................................................................caccgggcgaaaaguuugcuu....................  4      0  S01
........................................................................accgggcgaaaaguuugcuu....................  5      0  S01
.........................................................................ccgggcgaaaaguuugcuu....................  3      0  S01
..........................................................................cgggcgaaaaguuugcuu....................  3      0  S01

...................................caaaauuuuucgcugcgg...........................................................  1      0  S06
...................................caaaauuuuucgcugcggu..........................................................  1      0  S06
...................................caaaauuuuucgcugcggugg........................................................  1      0  S06
```

```
                                   Star                              Mature
uguuauguuucaucucuguuuuuuucuuuacgggacaaaauuuuucgcugcggugguuuagauuugaaaaucaccgggcgaaaaguuugcuuuugugaugaaauaaaucugu
...................................caaaauuuuucgcugcgguggu.......................................................   3       0   S06
...................................caaaauuuuucgcugcggugguu......................................................   119     0   S06
...................................caaaauuuuucgcugcggugguuu.....................................................   5       0   S06
....................................aaaauuuuucgcugcggugguu......................................................   1       0   S06
.....................................aaauuuuucgcugcggugguu......................................................   2       0   S06
......................................aauuuuucgcugcggugguu......................................................   3       0   S06
........................................................uuuagauuugaaaaucaccgggcgaaaaguuugcuu....................   1       0   S06
.........................................................uuagauuugaaaaucaccgggc.................................   3       0   S06
....................................................................aaucaccgggcgaaaaguuugc......................   1       0   S06
....................................................................aaucaccgggcgaaaaguuugcuu....................   1       0   S06
.....................................................................aucaccgggcgaaaaguuugc......................   1       0   S06
.....................................................................aucaccgggcgaaaaguuugcu.....................   2       0   S06
......................................................................ucaccgggcgaaaaguuu........................   11      0   S06
......................................................................ucaccgggcgaaaaguuugc......................   165     0   S06
......................................................................ucaccgggcgaaaaguuugcu.....................   5848    0   S06
......................................................................ucaccgggcgaaaaguuugcuu....................   88899   0   S06
......................................................................ucaccgggcgaaaaguuugcuuu...................   1181    0   S06
......................................................................ucaccgggcgaaaaguuugcuuuu..................   17      0   S06
.......................................................................caccgggcgaaaaguuugcu.....................   1       0   S06
.......................................................................caccgggcgaaaaguuugcuu....................   36      0   S06
........................................................................accgggcgaaaaguuugcuu....................   16      0   S06
.........................................................................ccgggcgaaaaguuugcu.....................   1       0   S06
.........................................................................ccgggcgaaaaguuugcuu....................   48      0   S06
..........................................................................cgggcgaaaaguuugcuu....................   26      0   S06

............ucucuguuuuuuucuuuacggga.............................................................................   1       0   S08
...................................caaaauuuuucgcugcgg...........................................................   2       0   S08
...................................caaaauuuuucgcugcgguggu.......................................................   14      0   S08
...................................caaaauuuuucgcugcggugguu......................................................   205     0   S08
...................................caaaauuuuucgcugcggugguuu.....................................................   6       0   S08
....................................aaaauuuuucgcugcggugguu......................................................   2       0   S08
.....................................aaauuuuucgcugcggugguu......................................................   1       0   S08
......................................aauuuuucgcugcggugguu......................................................   3       0   S08
.........................................................uuagauuugaaaaucaccgggc.................................   1       0   S08
...............................................................uugaaaaucaccgggcgaaaaguuugcuu....................   1       0   S08
......................................................................ucaccgggcgaaaaguuu........................   6       0   S08
......................................................................ucaccgggcgaaaaguuugc......................   193     0   S08
......................................................................ucaccgggcgaaaaguuugcu.....................   10833   0   S08
......................................................................ucaccgggcgaaaaguuugcuu....................   141159  0   S08
......................................................................ucaccgggcgaaaaguuugcuuu...................   1719    0   S08
......................................................................ucaccgggcgaaaaguuugcuuuu..................   33      0   S08
.......................................................................caccgggcgaaaaguuugcu.....................   1       0   S08
.......................................................................caccgggcgaaaaguuugcuu....................   60      0   S08
.......................................................................caccgggcgaaaaguuugcuuu...................   1       0   S08
........................................................................accgggcgGaaaguuugc......................   1       1   S08
........................................................................accgggcgaaaaguuugcu.....................   2       0   S08
........................................................................accgggcgaaaaguuugcuu....................   23      0   S08
.........................................................................ccgggcgaaaaguuugcu.....................   2       0   S08
.........................................................................ccgggcgaaaaguuugcuu....................   70      0   S08
.........................................................................ccgggcgaaaaguuugcuuu...................   1       0   S08
..........................................................................cgggcgaaaaguuugcuu....................   53      0   S08
..........................................................................cgggcgaaaaguuugcuuuu..................   1       0   S08

............ucucuguuuuuuucuuuacggga.............................................................................   1       0   S03
..............ucuguuuuuuucuuuacggga.............................................................................   1       0   S03
...............cuguuuuuuucuuuacggga.............................................................................   1       0   S03
...................................caaaauuuuucgcugcgg...........................................................   1       0   S03
...................................caaaauuuuucgcugcggugg........................................................   1       0   S03
...................................caaaauuuuucgcugcgguggu.......................................................   5       0   S03
...................................caaaauuuuucgcugcggugguu......................................................   156     0   S03
...................................caaaauuuuucgcugcggugguuu.....................................................   2       0   S03
...................................caaaauuuuucgcugcggugguuuagauuu...............................................   1       0   S03
...................................caaaauuuuucgcugcggugguuuagauuugaaa...........................................   1       0   S03
...................................caaaauuuuucgcugcggugguuuagauuugaaaa..........................................   1       0   S03
....................................aaaauuuuucgcugcggugguu......................................................   1       0   S03
......................................aauuuuucgcugcgguggu.......................................................   1       0   S03
........................................uuuuucgcugcggugguu......................................................   1       0   S03
..............................................................uuugaaaaucaccgggcgaaaaguuugcuu....................   1       0   S03
.....................................................................aucaccgggcgaaaaguuugcu.....................   1       0   S03
......................................................................ucaccgggcgaaaaguuu........................   5       0   S03
```

Star Mature

uguuauguuucaucucuguuuuuuucuuuacgggacaaaauuuuucgcugcgguggu uuagauuugaaaaucaccgggcgaaaaguuugcuuuugugaugaaauaaaucugu			
....ucaccgggcgaaaaguuugc......................	76	0	S03
....ucaccgggcgaaaaguuugcu.....................	2850	0	S03
....ucaccgggcgaaaaguuugcuu....................	67942	0	S03
....ucaccgggcgaaaaguuugcuuu...................	1245	0	S03
....ucaccgggcgaaaaguuugcuuuu..................	35	0	S03
....caccgggcgaaaaguuugcuu....................	23	0	S03
....caccgggcgaaaaguuugcuuu...................	1	0	S03
....accgggcgaaaaguuugcuu....................	10	0	S03
....ccgggcgaaaaguuugcuu....................	26	0	S03
....cgggcgaaaaguuugcuu....................	15	0	S03
....cgggcgaaaaguuugcuuu...................	1	0	S03
...................................caaaauuuuucgcugcggugg..	2	0	S09
...................................caaaauuuuucgcugcgguggu...	1	0	S09
...................................caaaauuuuucgcugcggugguu..	36	0	S09
...................................caaaauuuuucgcugcggugguuu...	1	0	S09
....ucaccgggcgaaaaguuu........................	1	0	S09
....ucaccgggcgaaaaguuugc......................	21	0	S09
....ucaccgggcgaaaaguuugcu.....................	1732	0	S09
....ucaccgggcgaaaaguuugcuu....................	15059	0	S09
....ucaccgggcgaaaaguuugcuuu...................	329	0	S09
....ucaccgggcgaaaaguuugcuuuu..................	12	0	S09
....caccgggcgaaaaguuugcuu....................	4	0	S09
....accgggcgaaaaguuugcuu....................	3	0	S09
....accgggcgaaaaguuugcuuu...................	1	0	S09
...................................caaaauuuuucgcugcggugguu..	18	0	S02
....ucaccgggcgaaaaguuu........................	7	0	S02
....ucaccgggcgaaaaguuugc......................	8	0	S02
....ucaccgggcgaaaaguuugcu.....................	213	0	S02
....ucaccgggcgaaaaguuugcuu....................	8021	0	S02
....ucaccgggcgaaaaguuugcuuu...................	50	0	S02
....ucaccgggcgaaaaguuugcuuuu..................	3	0	S02
....caccgggcgaaaaguuugcuu....................	3	0	S02
....ccgggcgaaaaguuugcuu....................	3	0	S02
....cgggcgaaaaguuugcuu....................	9	0	S02
.........ucaucucuguuuuuuucuuuacggga...	1	0	S04
..............ucuguuuuuuucuuuacggga...	6	0	S04
...............cuguuuuuuucuuuacggga...	1	0	S04
................uguuuuuuucuuuacggga...	1	0	S04
...................................caaaauuuuucgcugcgguggu...	5	0	S04
...................................caaaauuuuucgcugcggugguu..	213	0	S04
...................................caaaauuuuucgcugcggugguuu...	4	0	S04
....................................aaaauuuuucgcugcggugguu..	6	0	S04
......................................aauuuuucgcugcggugguu..	1	0	S04
....uuuagauuugaaaaucaccgggcgaaaaguuugcuuu...................	1	0	S04
....aaaucaccgggcgaaaaguuugc......................	1	0	S04
....aaucaccgggcgaaaaguuugcuu....................	2	0	S04
....aucaccgggcgaaaaguuugcuu....................	1	0	S04
....ucaccgggcgaaaaguuu........................	3	0	S04
....ucaccgggcgaaaaguuugc......................	64	0	S04
....ucaccgggcgaaaaguuugcu.....................	2879	0	S04
....ucaccgggcgaaaaguuugcuu....................	79112	0	S04
....ucaccgggcgaaaaguuugcuuu...................	1455	0	S04
....ucaccgggcgaaaaguuugcuuuu..................	34	0	S04
....caccgggcgaaaaguuugc......................	1	0	S04
....caccgggcgaaaaguuugcuu....................	25	0	S04
....caccgggcgaaaaguuugcuuu...................	5	0	S04
....accgggcgaaaaguuugcuu....................	13	0	S04
....accgggcgaaaaguuugcuuu...................	1	0	S04
....ccgggcgaaaaguuugcuu....................	30	0	S04
....ccgggcgaaaaguuugcuuu...................	2	0	S04
....cgggcgaaaaguuugcuu....................	22	0	S04

Provisional ID : Scaffold6623_45532
Score total : 2253
Score for star read(s) : 3.9
Score for read counts : 2247.4
Score for mfe : 0.1
Score for randfold : 1.6
Score for cons. seed :
Total read count : 4420
Mature read count : 4416
Loop read count : 0
Star read count : 4

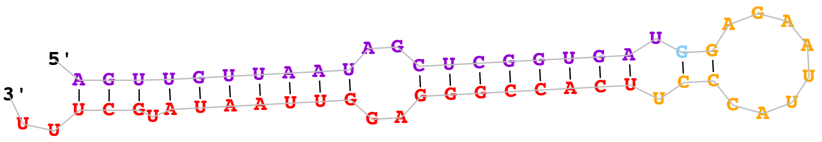

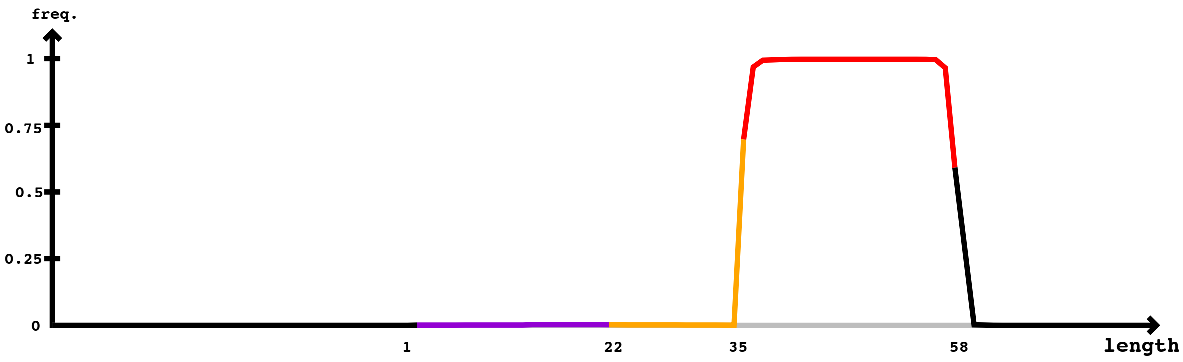

Star **Mature**

```
5'- aacagccuuuuauguucaucuaauuuuuucauuacuaguuguuaauagcucggugauggagaauuacccuucaccgggagguuaauaugcuuuuaaacuugagaaccaggguu -3'  obs
    aacagccuuuuauguucaucuaauuuuuucauuacuaguuguuaauagcucggugauggagaauuacccuucaccgggagguuaauaugcuuuuaaacuugagaaccaggguu       exp
    ...((((((....((((...........(((((..((((((((((..((((((((.((........)).))))))))..))))))).)))..)))...))))))).))))))   reads  mm  sample
    ....................................aguuguuaauagcucggugau........................................................   1      0   S04
    ......................................................................ucaccgggagguuaauaugcu......................   16     0   S04
    ......................................................................ucaccgggagguuaauaugcuu.....................   166    0   S04
    ......................................................................ucaccgggagguuaauaugcuuu....................   312    0   S04
    ......................................................................ucaccgggagguuaauaugcuuuu...................   26     0   S04
    .......................................................................caccgggagguuaauaugcu......................   12     0   S04
    .......................................................................caccgggagguuaauaugcuu.....................   107    0   S04
    .......................................................................caccgggagguuaauaugcuuu....................   39     0   S04
    .......................................................................caccgggagguuaauaugcuuuu...................   14     0   S04
    ........................................................................accgggagguuaauaugcuuu....................   4      0   S04
    ........................................................................accgggagguuaauaugcuuuu...................   9      0   S04
    .........................................................................ccgggagguuaauaugcuu.....................   1      0   S04
    ..........................................................................cgggagguuaauaugcuu.....................   2      0   S04
    ...........................................................................gggagguuaauaugcuuu....................   1      0   S04

    ....................................aguuguuaauagcucggugа.........................................................   1      0   S02
    ......................................................................ucaccgggagguuaauaugcu......................   5      0   S02
    ......................................................................ucaccgggagguuaauaugcuu.....................   52     0   S02
    ......................................................................ucaccgggagguuaauaugcuuu....................   166    0   S02
    ......................................................................ucaccgggagguuaauaugcuuuu...................   20     0   S02
    .......................................................................caccgggagguuaauaugcu......................   2      0   S02
    .......................................................................caccgggagguuaauaugcuu.....................   106    0   S02
    .......................................................................caccgggagguuaauaugcuuu....................   84     0   S02
    .......................................................................caccgggagguuaauaugcuuuu...................   34     0   S02
    ........................................................................accgggagguuaauaugcuuu....................   2      0   S02
    ........................................................................accgggagguuaauaugcuuuu...................   10     0   S02
    .........................................................................ccgggagguuaauaugcuuuu...................   2      0   S02
    ..........................................................................cgggagguuaauaugcuuu....................   1      0   S02

    ....................................aguuguuaauagcucggugaug.......................................................   1      0   S03
    ......................................................................ucaccgggagguuaauaugc.......................   2      0   S03
    ......................................................................ucaccgggagguuaauaugcu......................   19     0   S03
    ......................................................................ucaccgggagguuaauaugcuu.....................   203    0   S03
```

```
                                    Star                             Mature
aacagccuuuuauguucaucuaauuuuuucauuacuaguuguuaauagcucggugauggagaauuacccuucaccgggagguuaauaugcuuuuaaacuugagaaccaggguu
......................................................................ucaccgggagguuaauaugcuuu....................    395    0    S03
......................................................................ucaccgggagguuaauaugcuuuu...................    30     0    S03
......................................................................ucaccgggagguuaauaugcuuuua..................    1      0    S03
.......................................................................caccgggagguuaauaugc.......................    1      0    S03
.......................................................................caccgggagguuaauaugcu......................    9      0    S03
.......................................................................caccgggagguuaauaugcuu.....................    115    0    S03
.......................................................................caccgggagguuaauaugcuuu....................    40     0    S03
.......................................................................caccgggagguuaauaugcuuuu...................    12     0    S03
........................................................................accgggagguuaauaugcuuu....................    4      0    S03
........................................................................accgggagguuaauaugcuuuu...................    16     0    S03
........................................................................accgggagguuaauaugcuuuuaa.................    1      0    S03
........................................................................accgggagguuaauaugcuuuuaaa................    1      0    S03
.........................................................................ccgggagguuaauaugcuu.....................    1      0    S03

......................................................................ucaccgggagguuaauaugcu......................    4      0    S09
......................................................................ucaccgggagguuaauaugcuu.....................    27     0    S09
......................................................................ucaccgggagguuaauaugcuuu....................    103    0    S09
......................................................................ucaccgggagguuaauaugcuuuu...................    9      0    S09
.......................................................................caccgggagguuaauaugcu......................    1      0    S09
.......................................................................caccgggagguuaauaugcuu.....................    8      0    S09
........................................................................accgggagguuaauaugcuuuu...................    5      0    S09

......................................................................ucaccgggagguuaauaugcu......................    5      0    S08
......................................................................ucaccgggagguuaauaugcuu.....................    140    0    S08
......................................................................ucaccgggagguuaauaugcuuu....................    239    0    S08
......................................................................ucaccgggagguuaauaugcuuuu...................    10     0    S08
.......................................................................caccgggagguuaauaugcu......................    7      0    S08
.......................................................................caccgggagguuaauaugcuu.....................    56     0    S08
.......................................................................caccgggagguuaauaugcuuu....................    8      0    S08
.......................................................................caccgggagguuaauaugcuuuu...................    2      0    S08
........................................................................accgggagguuaauaugcuuu....................    1      0    S08
........................................................................accgggagguuaauaugcuuuu...................    11     0    S08
.........................................................................ccgggagguuaauaugcuu.....................    1      0    S08
..........................................................................cgggagguuaauaugcuu.....................    1      0    S08

................................................cucggugauggagaauuacc.............................................    1      0    S06
................................................cucggugauggagaauuacccu...........................................    1      0    S06
......................................................................ucaccgggagguuaauaugcu......................    5      0    S06
......................................................................ucaccgggagguuaauaugcuu.....................    39     0    S06
......................................................................ucaccgggagguuaauaugcuuu....................    47     0    S06
......................................................................ucaccgggagguuaauaugcuuuu...................    8      0    S06
.......................................................................caccgggagguuaauaugc.......................    1      0    S06
.......................................................................caccgggagguuaauaugcu......................    6      0    S06
.......................................................................caccgggagguuaauaugcuu.....................    58     0    S06
.......................................................................caccgggagguuaauaugcuuu....................    15     0    S06
.......................................................................caccgggagguuaauaugcuuuu...................    6      0    S06
........................................................................accgggagguuaauaugc.......................    1      0    S06
........................................................................accgggagguuaauaugcuu.....................    1      0    S06
........................................................................accgggagguuaauaugcuuu....................    2      0    S06
........................................................................accgggagguuaauaugcuuuu...................    4      0    S06
...........................................................................gggagguuaauaugcuuuu...................    1      0    S06

....................................aguuguuaauagcucggugau........................................................    1      0    S01
.....................................................................uucaccgggagguuaauaugcuu.....................    1      0    S01
......................................................................ucaccgggagguuaauaugcu......................    11     0    S01
......................................................................ucaccgggagguuaauaugcuu.....................    49     0    S01
......................................................................ucaccgggagguuaauaugcuuu....................    129    0    S01
......................................................................ucaccgggagguuaauaugcuuuu...................    15     0    S01
......................................................................ucaccgggagguuaauaugcuuuua..................    1      0    S01
......................................................................ucaccgggagguuaauaugcuuuuaa.................    1      0    S01
.......................................................................caccgggagguuaauaugcu......................    5      0    S01
.......................................................................caccgggagguuaauaugcuu.....................    95     0    S01
.......................................................................caccgggagguuaauaugcuuu....................    85     0    S01
.......................................................................caccgggagguuaauaugcuuuu...................    49     0    S01
........................................................................accgggagguuaauaugcuuu....................    2      0    S01
........................................................................accgggagguuaauaugcuuuu...................    10     0    S01
........................................................................accgggagguuaauaugcuuuuaa.................    1      0    S01
..........................................................................cgggagguuaauaugcuuuu...................    1      0    S01

......................................................................ucaccgggagguuaauaugc.......................    1      0    S07
```

Star Mature

aacagccuuuuauguucaucuaauuuuuuucauuacuaguuguuaauagcucggugauggagaauuacccuucaccgggagguuaaauaugcuuuuaaacuugagaaccaggguu			
....ucaccgggagguuaaauaugcu......................	8	0	S07
....ucaccgggagguuaaauaugcuu.....................	80	0	S07
....ucaccgggagguuaaauaugcuuu....................	146	0	S07
....ucaccgggagguuaaauaugcuuuu...................	15	0	S07
....caccgggagguuaaauaugcu......................	3	0	S07
....caccgggagguuaaauaugcuu.....................	41	0	S07
....caccgggagguuaaauaugcuuu....................	14	0	S07
....caccgggagguuaaauaugcuuuu...................	5	0	S07
....accgggagguuaaauaugcuuu....................	2	0	S07
....accgggagguuaaauaugcuuuu...................	7	0	S07
....gggagguuaaauaugcuuu....................	1	0	S07
....uuaauagcucggugauggagaau..	1	0	S05
....cucggugauggagaauuaccc...	2	0	S05
....cucggugauggagaauuacccu..	1	0	S05
....ucaccgggagguuaaauaug........................	1	0	S05
....ucaccgggagguuaaauaugcu......................	7	0	S05
....ucaccgggagguuaaauaugcuu.....................	179	0	S05
....ucaccgggagguuaaauaugcuuu....................	274	0	S05
....ucaccgggagguuaaauaugcuuuu...................	38	0	S05
....ucaccgggagguuaaauaugcuuuua..................	1	0	S05
....caccgggagguuaaauaugcu......................	7	0	S05
....caccgggagguuaaauaugcuu.....................	83	0	S05
....caccgggagguuaaauaugcuuu....................	28	0	S05
....caccgggagguuaaauaugcuuuu...................	8	0	S05
....accgggagguuaaauaugcu......................	1	0	S05
....accgggagguuaaauaugcuu.....................	2	0	S05
....accgggagguuaaauaugcuuu....................	4	0	S05
....accgggagguuaaauaugcuuuu...................	12	0	S05
....cgggagguuaaauaugcuuu....................	1	0	S05
....cgggagguuaaauaugcuuuu...................	1	0	S05
....ggagguuaaauaugcuuuu...................	1	0	S05
....ucaccgggagguuaaauaugcu......................	2	0	S10
....ucaccgggagguuaaauaugcuu.....................	20	0	S10
....ucaccgggagguuaaauaugcuuu....................	47	0	S10
....ucaccgggagguuaaauaugcuuuu...................	11	0	S10
....caccgggagguuaaauaugcu......................	1	0	S10
....caccgggagguuaaauaugcuu.....................	21	0	S10
....caccgggagguuaaauaugcuuu....................	9	0	S10
....caccgggagguuaaauaugcuuuu...................	3	0	S10
....accgggagguuaaauaugcuuuuaa.................	1	0	S10

Provisional ID	: Scaffold5285_44887
Score total	: 100425.6
Score for star read(s)	: 3.9
Score for read counts	: 100425.5
Score for mfe	: -5.2
Score for randfold	: 1.6
Score for cons. seed	:
Total read count	: 196992
Mature read count	: 196460
Loop read count	: 3
Star read count	: 529

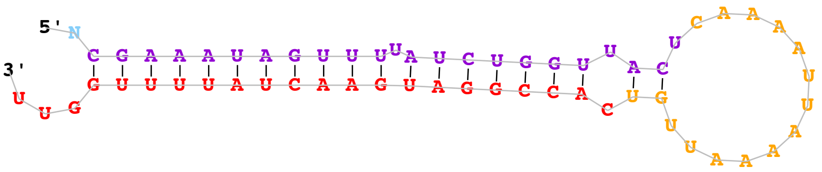

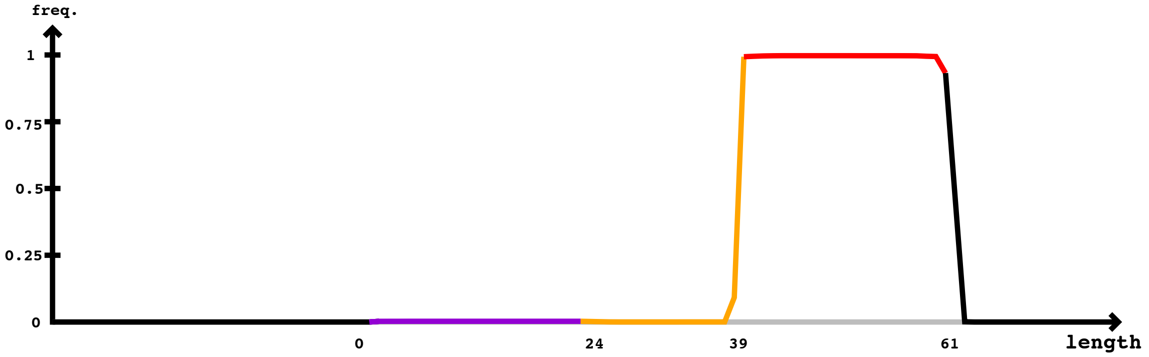

Star Mature

```
5'- nnnnnnnnnnnnnnnnnnnnnnnnnnnnnnnncgaaauaguuuuaucugguuacucaaaauuaaaaauugucaccggaugaacuauuuugguucuaugggauauuuauca  -3'   obs
    nnnnnnnnnnnnnnnnnnnnnnnnnnnnnnnncgaaauaguuuuaucugguuacucaaaauuaaaaauugucaccggaugaacuauuuugguucuaugggauauuuauca        exp
    ................................(((((((((((.(((((((.((...............)).)))))))))))))))))).........(((....))).  reads  mm  sample
    ................................cgaaauaguuuuaucugguuac........................................................  2      0   S07
    ................................cgaaauaguuuuaucugguuacu.......................................................  28     0   S07
    ................................cgaaauaguuuuaucugguuacuc......................................................  23     0   S07
    ................................cgaaauaguuuuaucugguuacuca.....................................................  11     0   S07
    ................................cgaaauaguuuuaucugguuacucaaaauuaaaau...........................................  1      0   S07
    ................................cgaaauaguuuuaucugguuacucaaaauuaaaaau..........................................  1      0   S07
    ................................cgaaauaguuuuaucugguuacucaaaauuaaaaauug........................................  3      0   S07
    ................................cgaaauaguuuuaucugguuacucaaaauuaaaaauugu.......................................  1      0   S07
    .................................gaaauaguuuuaucugguuac........................................................  1      0   S07
    .................................gaaauaguuuuaucugguuacu.......................................................  1      0   S07
    .................................gaaauaguuuuaucugguuacuc......................................................  3      0   S07
    ..................................aaauaguuuuaucugguuacuc......................................................  1      0   S07
    .....................................uaguuuuaucugguuacu.......................................................  1      0   S07
    .....................................................cucaaaauuaaaaauugucaccg..................................  2      0   S07
    .......................................................caaaauuaaaaauugucaccggau...............................  2      0   S07
    ...........................................................auuaaaaauugucaccggaugaacuauuuugguu.................  1      0   S07
    ............................................................uuaaaaauugucaccggaugaacuauuuugguu.................  2      0   S07
    .............................................................uaaaaauugucaccggaugaacuauuuugguu.................  1      0   S07
    .....................................................................gucaccggaugaacuauuuugguu.................  1      0   S07
    ......................................................................ucaccggaugaacuauuu......................  2      0   S07
    ......................................................................ucaccggaugaacuauuuug....................  7      0   S07
    ......................................................................ucaccggaugaacuauuuugg...................  29     0   S07
    ......................................................................ucaccggaugaacuauuuuggu..................  850    0   S07
    ......................................................................ucaccggaugaacuauuuugguu.................  2036   0   S07
    ......................................................................ucaccggaugaacuauuuugguuc................  28     0   S07
    ......................................................................ucaccggaugaacuauuuugguucu...............  1      0   S07
    .......................................................................caccggaugaacuauuu......................  6      0   S07
    .......................................................................caccggaugaacuauuuug....................  60     0   S07
    .......................................................................caccggaugaacuauuuugg...................  20     0   S07
    .......................................................................caccggaugaacuauuuuggu..................  890    0   S07
    .......................................................................caccggaugaacuauuuugguu.................  33079  0   S07
    .......................................................................caccggaugaacuauuuugguuc................  316    0   S07
    .......................................................................caccggaugaacuauuuugguucu...............  5      0   S07
```

Star Mature

```
nnnnnnnnnnnnnnnnnnnnnnnnnnnnnnnncgaaauaguuuuaucugguuacucaaaauuaaaauugucaccggaugaacuauuuugguucuaugggauauuuauca
.......................................................................accggaugaacuauuuug....................    1    0    S07
.......................................................................accggaugaacuauuuugg...................    1    0    S07
.......................................................................accggaugaacuauuuuggu..................    2    0    S07
.......................................................................accggaugaacuauuuugguu.................    24   0    S07
.......................................................................accggaugaacuauuuugguuc................    9    0    S07
.......................................................................accggaugaacuauuuugguucu...............    6    0    S07
........................................................................ccggaugaacuauuuuggu..................    1    0    S07
........................................................................ccggaugaacuauuuugguu.................    10   0    S07
........................................................................ccggaugaacuauuuugguuc................    13   0    S07
........................................................................ccggaugaacuauuuugguucu...............    15   0    S07
.........................................................................cggaugaacuauuuuggu..................    3    0    S07
.........................................................................cggaugaacuauuuugguu.................    6    0    S07
.........................................................................cggaugaacuauuuugguuc................    1    0    S07
..........................................................................ggaugaacuauuuugguu.................    5    0    S07

.....................................................................ucaccggaugaacuauuuugguu.................    1    0    S01
......................................................................caccggaugaacuauuuugguu.................    8    0    S01

................................cgaaauaguuuuaucugguu.........................................................    1    0    S06
................................cgaaauaguuuuaucugguuac.......................................................    4    0    S06
................................cgaaauaguuuuaucugguuacu......................................................    30   0    S06
................................cgaaauaguuuuaucugguuacuc.....................................................    6    0    S06
................................cgaaauaguuuuaucugguuacuca....................................................    5    0    S06
.................................gaaauaguuuuaucugguuacuc.....................................................    1    0    S06
...................................................................ugucaccggaugaacuauuuugguuc................    1    0    S06
....................................................................gucaccggaugaacuauuuugguu.................    1    0    S06
.....................................................................ucaccggaugaacuauuu......................    1    0    S06
.....................................................................ucaccggaugaacuauuuu.....................    2    0    S06
.....................................................................ucaccggaugaacuauuuug....................    2    0    S06
.....................................................................ucaccggaugaacuauuuugg...................    5    0    S06
.....................................................................ucaccggaugaacuauuuuggu..................    138  0    S06
.....................................................................ucaccggaugaacuauuuugguu.................    472  0    S06
.....................................................................ucaccggaugaacuauuuugguuc................    4    0    S06
.....................................................................ucaccggaugaacuauuuugguucu...............    1    0    S06
......................................................................caccggaugaacuauuuu.....................    5    0    S06
......................................................................caccggaugaacuauuuug....................    8    0    S06
......................................................................caccggaugaacuauuuugg...................    4    0    S06
......................................................................caccggaugaacuauuuuggu..................    188  0    S06
......................................................................caccggaugaacuauuuugguu.................    6221 0    S06
......................................................................caccggaugaacuauuuugguuc................    91   0    S06
......................................................................caccggaugaacuauuuugguucu...............    2    0    S06
.......................................................................accggaugaacuauuuug....................    1    0    S06
.......................................................................accggaugaacuauuuugguu.................    6    0    S06
.......................................................................accggaugaacuauuuugguuc................    7    0    S06
.......................................................................accggaugaacuauuuugguucu...............    3    0    S06
........................................................................ccggaugaacuauuuugguu.................    2    0    S06
........................................................................ccggaugaacuauuuugguuc................    3    0    S06
........................................................................ccggaugaacuauuuugguucu...............    3    0    S06
.........................................................................cggaugaacuauuuugguu.................    4    0    S06
.........................................................................cggaugaacuauuuugguuc................    1    0    S06
.........................................................................cggaugaacuauuuugguucu...............    1    0    S06
..........................................................................ggaugaacuauuuugguu.................    1    0    S06

................................cgaaauaguuuuaucugg...........................................................    1    0    S10
................................cgaaauaguuuuaucugguua........................................................    1    0    S10
................................cgaaauaguuuuaucugguuac.......................................................    13   0    S10
................................cgaaauaguuuuaucugguuacu......................................................    98   0    S10
................................cgaaauaguuuuaucugguuacuc.....................................................    32   0    S10
................................cgaaauaguuuuaucugguuacuca....................................................    8    0    S10
................................cgaaauaguuuuaucugguuacucaaaauuaaaauu.........................................    6    0    S10
.................................gaaauaguuuuaucugguuacu......................................................    1    0    S10
..................................aaauaguuuuaucugguua........................................................    1    0    S10
....................................auaguuuuaucugguuac.......................................................    1    0    S10
.....................................................cucaaaauuaaaauugucaccg..................................    1    0    S10
.......................................................caaaauuaaaauugucaccggaug..............................    1    0    S10
.........................................................aaauuaaaauugucaccggaug..............................    1    0    S10
............................................................uuaaaauugucaccggaug..............................    1    0    S10
............................................................uuaaaauugucaccggaugaac...........................    3    0    S10
...............................................................aaauugucaccggaugaacuauu.......................    1    0    S10
................................................................aauugucaccggaugaacu..........................    1    0    S10
```

Star **Mature**

```
nnnnnnnnnnnnnnnnnnnnnnnnnnnnnnnncgaaauaguuuuaucugguuacucaaaauuaaaaauugucaccggaugaacuauuuugguucuaugggauauuuauca
.................................................................aauugucaccggaugaacuauuuugguu.................   2      0   S10
..................................................................auugucaccggaugaacu..........................   1      0   S10
....................................................................ugucaccggaugaacuauuuug....................   1      0   S10
....................................................................ugucaccggaugaacuauuuugguu.................   1      0   S10
......................................................................ucaccggaugaacuauuu......................   2      0   S10
......................................................................ucaccggaugaacuauuuug....................   10     0   S10
......................................................................ucaccggaugaacuauuuugg...................   36     0   S10
......................................................................ucaccggaugaacuauuuuggu..................   2167   0   S10
......................................................................ucaccggaugaacuauuuugguu.................   3455   0   S10
......................................................................ucaccggaugaacuauuuugguuc................   62     0   S10
......................................................................ucaccggaugaacuauuuugguucu...............   2      0   S10
.......................................................................caccggaugaacuauuuu.....................   11     0   S10
.......................................................................caccggaugaacuauuuug....................   90     0   S10
.......................................................................caccggaugaacuauuuugg...................   34     0   S10
.......................................................................caccggaugaacuauuuuggu..................   1937   0   S10
.......................................................................caccggaugaacuauuuugguu.................   53494  0   S10
.......................................................................caccggaugaacuauuuugguuc................   472    0   S10
.......................................................................caccggaugaacuauuuugguucu...............   10     0   S10
........................................................................accggaugaacuauuuug....................   1      0   S10
........................................................................accggaugaacuauuuugg...................   1      0   S10
........................................................................accggaugaacuauuuuggu..................   4      0   S10
........................................................................accggaugaacuauuuugguu.................   56     0   S10
........................................................................accggaugaacuauuuugguuc................   18     0   S10
........................................................................accggaugaacuauuuugguucu...............   4      0   S10
.........................................................................ccggaugaacuauuuugguu.................   20     0   S10
.........................................................................ccggaugaacuauuuugguuc................   5      0   S10
.........................................................................ccggaugaacuauuuugguucu...............   17     0   S10
..........................................................................cggaugaacuauuuuggu..................   4      0   S10
..........................................................................cggaugaacuauuuugguu.................   18     0   S10
...........................................................................ggaugaacuauuuugguu.................   21     0   S10

................................cgaaauaguuuuaucugguuacu.......................................................   11     0   S05
................................cgaaauaguuuuaucugguuacuc......................................................   6      0   S05
................................cgaaauaguuuuaucugguuacuca.....................................................   2      0   S05
..................................aaauaguuAuaucugguua.........................................................   1      1   S05
......................................................................ucaccggaugaacuauuu......................   1      0   S05
......................................................................ucaccggaugaacuauuuug....................   3      0   S05
......................................................................ucaccggaugaacuauuuugg...................   2      0   S05
......................................................................ucaccggaugaacuauuuuggu..................   97     0   S05
......................................................................ucaccggaugaacuauuuugguu.................   318    0   S05
......................................................................ucaccggaugaacuauuuugguuc................   10     0   S05
.......................................................................caccggaugaacuauuuu.....................   1      0   S05
.......................................................................caccggaugaacuauuuug....................   3      0   S05
.......................................................................caccggaugaacuauuuugg...................   4      0   S05
.......................................................................caccggaugaacuauuuuggu..................   88     0   S05
.......................................................................caccggaugaacuauuuugguu.................   4470   0   S05
.......................................................................caccggaugaacuauuuugguuc................   79     0   S05
.......................................................................caccggaugaacuauuuugguucu...............   1      0   S05
........................................................................accggaugaacuauuuuggu..................   1      0   S05
........................................................................accggaugaacuauuuugguu.................   7      0   S05
........................................................................accggaugaacuauuuugguuc................   9      0   S05
........................................................................accggaugaacuauuuugguucu...............   2      0   S05
.........................................................................ccggaugaacuauuuugguu.................   3      0   S05
.........................................................................ccggaugaacuauuuugguuc................   6      0   S05
.........................................................................ccggaugaacuauuuugguucu...............   6      0   S05
..........................................................................cggaugaacuauuuugguu.................   4      0   S05
...........................................................................ggaugaacuauuuugguu.................   2      0   S05

..................................aaauaguuAuaucugguu..........................................................   2      1   S02
..................................aaauaguuAuaucugguua.........................................................   2      1   S02
......................................................................ucaccggaugaacuauuuuggu..................   2      0   S02
.......................................................................caccggaugaacuauuuuggu..................   1      0   S02
.......................................................................caccggaugaacuauuuugguu.................   14     0   S02
.......................................................................caccggaugaacuauuuugguuc................   1      0   S02

................................cgaaauaguuuuaucugguuacu.......................................................   13     0   S04
................................cgaaauaguuuuaucugguuacuc......................................................   4      0   S04
................................cgaaauaguuuuaucugguuacuca.....................................................   1      0   S04
............................................................uuaaaaauugucaccggaugaac...........................   1      0   S04
.................................................................aauugucaccggaugaacu..........................   1      0   S04
```

Star Mature

```
nnnnnnnnnnnnnnnnnnnnnnnnnnnnnnnncgaaauaguuuuaucugguuacucaaaauuaaaaauugucaccggaugaacuauuuugguucuaugggauauuuauca
......................................................................ucaccggaugaacuauuuugg...................   3      0   S04
......................................................................ucaccggaugaacuauuuuggu..................   109    0   S04
......................................................................ucaccggaugaacuauuuugguu.................   237    0   S04
......................................................................ucaccggaugaacuauuuugguuc................   10     0   S04
.......................................................................caccggaugaacuauuuu.....................   1      0   S04
.......................................................................caccggaugaacuauuuug....................   6      0   S04
.......................................................................caccggaugaacuauuuugg...................   4      0   S04
.......................................................................caccggaugaacuauuuuggu..................   92     0   S04
.......................................................................caccggaugaacuauuuugguu.................   3737   0   S04
.......................................................................caccggaugaacuauuuugguuc................   53     0   S04
.......................................................................caccggaugaacuauuuugguucu...............   1      0   S04
........................................................................accggaugaacuauuuugguu.................   8      0   S04
........................................................................accggaugaacuauuuugguuc................   4      0   S04
.........................................................................ccggaugaacuauuuugguu.................   4      0   S04
.........................................................................ccggaugaacuauuuugguuc................   1      0   S04
.........................................................................ccggaugaacuauuuugguucu...............   9      0   S04
..........................................................................cggaugaacuauuuuggu..................   1      0   S04
..........................................................................cggaugaacuauuuugguu.................   2      0   S04
...........................................................................ggaugaacuauuuugguu.................   3      0   S04

................................cgaaauaguuuuaucugguuac........................................................   5      0   S08
................................cgaaauaguuuuaucugguuacu.......................................................   49     0   S08
................................cgaaauaguuuuaucugguuacuc......................................................   13     0   S08
................................cgaaauaguuuuaucugguuacuca.....................................................   6      0   S08
................................cgaaauaguuuuaucugguuacucaaaauuaaaaauu.........................................   4      0   S08
................................cgaaauaguuuuaucugguuacucaaaauuaaaaauugu.......................................   1      0   S08
.................................gaaauaguuuuaucugguuac........................................................   1      0   S08
..................................aaauaguuuuaucugguuac........................................................   1      0   S08
...................................aauaguuuuaucugguuac........................................................   2      0   S08
.........................................uuuaucugguuacucaaaauuaaaaauugu.......................................   1      0   S08
...................................................uacucaaaauuaaaaauugucaccggaugaacuauuuugguu.................   3      0   S08
.....................................................cucaaaauuaaaaauugucaccgg..................................   1      0   S08
......................................................ucaaaauuaaaaauugucaccggaugaacu..........................   1      0   S08
.........................................................aaauuaaaaauugucaccggaugaacuauuuugguu.................   1      0   S08
.................................................................aauugucaccggaugaacuauuuugguu.................   2      0   S08
....................................................................ugucaccggaugaacuauuuugguu.................   2      0   S08
......................................................................ucaccggaugaacuauuu......................   1      0   S08
......................................................................ucaccggaugaacuauuuug....................   3      0   S08
......................................................................ucaccggaugaacuauuuugg...................   8      0   S08
......................................................................ucaccggaugaacuauuuuggu..................   443    0   S08
......................................................................ucaccggaugaacuauuuugguu.................   1206   0   S08
......................................................................ucaccggaugaacuauuuugguuc................   19     0   S08
.......................................................................caccggaugaacuauuuu.....................   9      0   S08
.......................................................................caccggaugaacuauuuug....................   15     0   S08
.......................................................................caccggaugaacuauuuugg...................   10     0   S08
.......................................................................caccggaugaacuauuuuggu..................   453    0   S08
.......................................................................caccggaugaacuauuuugguu.................   14088  0   S08
.......................................................................caccggaugaacuauuuugguuc................   198    0   S08
.......................................................................caccggaugaacuauuuugguucu...............   9      0   S08
........................................................................accggaugaacuauuuugguu.................   19     0   S08
........................................................................accggaugaacuauuuugguuc................   8      0   S08
........................................................................accggaugaacuauuuugguucu...............   3      0   S08
.........................................................................ccggaugaacuauuuuggu..................   11     0   S08
.........................................................................ccggaugaacuauuuugguu.................   30     0   S08
.........................................................................ccggaugaacuauuuugguuc................   23     0   S08
.........................................................................ccggaugaacuauuuugguucu...............   23     0   S08
..........................................................................cggaugaacuauuuugguu.................   45     0   S08
..........................................................................cggaugaacuauuuugguuc................   1      0   S08
...........................................................................ggaugaacuauuuugguu.................   10     0   S08

................................cgaaauaguuuuaucugguuac........................................................   12     0   S09
................................cgaaauaguuuuaucugguuacu.......................................................   78     0   S09
................................cgaaauaguuuuaucugguuacuc......................................................   28     0   S09
................................cgaaauaguuuuaucugguuacuca.....................................................   3      0   S09
.................................gaaauaguuuuaucugguuac........................................................   1      0   S09
..................................aaauaguuAuaucugguu..........................................................   3      1   S09
....................................auaguuuuaucugguuac........................................................   1      0   S09
.....................................................cucaaaauuaaaaauugucaccggaugaacuauuuugguu.................   1      0   S09
......................................................ucaaaauuaaaaauugucaccggaugaac...........................   1      0   S09
........................................................aaaauuaaaaauugucaccggau...............................   1      0   S09
```

```
                                Star                                 Mature
nnnnnnnnnnnnnnnnnnnnnnnnnnnnnnnncgaaauaguuuuaucugguuacucaaaauuaaaauugucaccggaugaacuauuuugguucuaugggauauuuauca
............................................................uuaaaauugucaccggaugaacu..........................  1      0  S09
............................................................uuaaaauugucaccggaugaacuauuuugguu.................  2      0  S09
....................................................................gucaccggaugaacuauuuugguu.................  1      0  S09
.....................................................................ucaccggaugaacuauuu......................  8      0  S09
.....................................................................ucaccggaugaacuauuuug....................  7      0  S09
.....................................................................ucaccggaugaacuauuuugg...................  30     0  S09
.....................................................................ucaccggaugaacuauuuuggu..................  2302   0  S09
.....................................................................ucaccggaugaacuauuuugguu.................  3653   0  S09
.....................................................................ucaccggaugaacuauuuugguuc................  63     0  S09
.....................................................................ucaccggaugaacuauuuugguucu...............  1      0  S09
......................................................................caccggaugaacuauuuu.....................  9      0  S09
......................................................................caccggaugaacuauuuug....................  74     0  S09
......................................................................caccggaugaacuauuuugg...................  30     0  S09
......................................................................caccggaugaacuauuuuggu..................  2303   0  S09
......................................................................caccggaugaacuauuuugguu.................  51824  0  S09
......................................................................caccggaugaacuauuuugguuc................  536    0  S09
......................................................................caccggaugaacuauuuugguucu...............  7      0  S09
.......................................................................accggaugaacuauuuug....................  4      0  S09
.......................................................................accggaugaacuauuuugg...................  1      0  S09
.......................................................................accggaugaacuauuuuggu..................  1      0  S09
.......................................................................accggaugaacuauuuugguu.................  58     0  S09
.......................................................................accggaugaacuauuuugguuc................  19     0  S09
.......................................................................accggaugaacuauuuugguucu...............  4      0  S09
........................................................................ccggaugaacuauuuuggu..................  1      0  S09
........................................................................ccggaugaacuauuuugguu.................  20     0  S09
........................................................................ccggaugaacuauuuugguuc................  8      0  S09
........................................................................ccggaugaacuauuuugguucu...............  12     0  S09
.........................................................................cggaugaacuauuuuggu..................  3      0  S09
.........................................................................cggaugaacuauuuugguu.................  24     0  S09
..........................................................................ggaugaacuauuuugguu.................  14     0  S09

................................cgaaauaguuuuaucugguuacu......................................................  6      0  S03
................................cgaaauaguuuuaucugguuacuc.....................................................  1      0  S03
................................cgaaauaguuuuaucugguuacuca....................................................  1      0  S03
................................cgaaauaguuuuaucugguuacucaaaauuaaaau..........................................  1      0  S03
.................................gaaauaguuuuaucugguuacu......................................................  1      0  S03
...................................aauaguuuuaucugguuac.......................................................  1      0  S03
.............................................................uaaaauugucaccggaugaacuauuuugguu.................  1      0  S03
..................................................................uugucaccggaugaacuauuuugguu.................  1      0  S03
.....................................................................ucaccggaugaacuauuu......................  1      0  S03
.....................................................................ucaccggaugaacuauuuug....................  3      0  S03
.....................................................................ucaccggaugaacuauuuugg...................  1      0  S03
.....................................................................ucaccggaugaacuauuuuggu..................  52     0  S03
.....................................................................ucaccggaugaacuauuuugguu.................  136    0  S03
.....................................................................ucaccggaugaacuauuuugguuc................  3      0  S03
......................................................................caccggaugaacuauuuu.....................  3      0  S03
......................................................................caccggaugaacuauuuug....................  6      0  S03
......................................................................caccggaugaacuauuuugg...................  1      0  S03
......................................................................caccggaugaacuauuuuggu..................  55     0  S03
......................................................................caccggaugaacuauuuugguu.................  2499   0  S03
......................................................................caccggaugaacuauuuugguuc................  32     0  S03
.......................................................................accggaugaacuauuuug....................  1      0  S03
.......................................................................accggaugaacuauuuugg...................  1      0  S03
.......................................................................accggaugaacuauuuugguuc................  4      0  S03
.......................................................................accggaugaacuauuuugguucu...............  1      0  S03
........................................................................ccggaugaacuauuuugguu.................  5      0  S03
........................................................................ccggaugaacuauuuugguuc................  6      0  S03
........................................................................ccggaugaacuauuuugguucu...............  12     0  S03
..........................................................................ggaugaacuauuuugguu.................  2      0  S03
```

Provisional ID	:	Scaffold1083_21830
Score total	:	580.9
Score for star read(s)	:	3.9
Score for read counts	:	579.3
Score for mfe	:	0
Score for randfold	:	-2.2
Score for cons. seed	:	
Total read count	:	1148
Mature read count	:	1082
Loop read count	:	0
Star read count	:	66

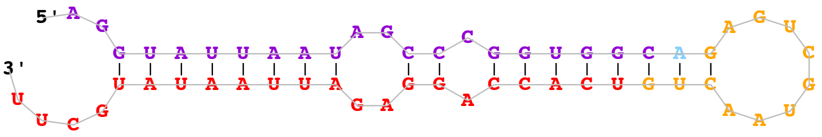

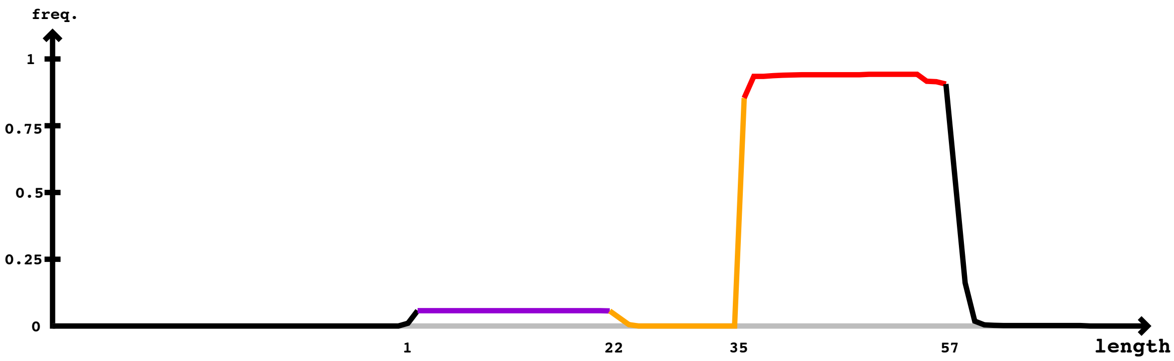

Star **Mature**

```
5'- aacugcguuuuauguucauuuaaaucuauucaagaccagguauuaaauagcccgguggcagagucguaacugucaccaggagauuaauaugcuuuuaaaccugaggacuagugu  -3'   obs
    aacugcguuuuauguucauuuaaaucuauucaagaccagguauuaaauagcccgguggcagagucguaacugucaccaggagauuaauaugcuuuuaaaccugaggacuagugu         exp
    .((((.(((((..(((........(((.....)))....((((((((..((.((((((((........)))))))).))..))))))))).......)))...)))))))))..    reads   mm   sample
    ....................................cagguauuaaauagcccgguggc.......................................................    4       0    S02
    .....................................agguauuaaauagcccgguggc.......................................................    23      0    S02
    .....................................agguauuaaauagcccgguggcag.....................................................    3       0    S02
    ........................................................................ucaccaggagauuaauaug.......................    6       0    S02
    ........................................................................ucaccaggagauuaauaugc......................    2       0    S02
    ........................................................................ucaccaggagauuaauaugcu.....................    2       0    S02
    ........................................................................ucaccaggagauuaauaugcuu....................    117     0    S02
    ........................................................................ucaccaggagauuaauaugcuuu...................    90      0    S02
    ........................................................................ucaccaggagauuaauaugcuuuu..................    39      0    S02
    ........................................................................ucaccaggagauuaauaugcuuuua.................    5       0    S02
    ........................................................................ucaccaggagauuaauaugcuuuuaaa...............    1       0    S02
    .........................................................................caccaggagauuaauaugcuu....................    3       0    S02
    .........................................................................caccaggagauuaauaugcuuu...................    12      0    S02
    .........................................................................caccaggagauuaauaugcuuuu..................    11      0    S02
    .........................................................................caccaggagauuaauaugcuuuua.................    2       0    S02
    ..............................................................................ggagauuaauaugcuuu...................    1       0    S02

    .....................................agguauuaaauagcccgguggc.......................................................    1       0    S04
    .....................................agguauuaaauagcccgguggca......................................................    1       0    S04
    ........................................................................ucaccaggagauuaauaug.......................    2       0    S04
    ........................................................................ucaccaggagauuaauaugcu.....................    1       0    S04
    ........................................................................ucaccaggagauuaauaugcuu....................    51      0    S04
    ........................................................................ucaccaggagauuaauaugcuuu...................    17      0    S04
    ........................................................................ucaccaggagauuaauaugcuuuu..................    17      0    S04
    ........................................................................ucaccaggagauuaauaugcuuuua.................    2       0    S04
    .........................................................................caccaggagauuaauaug.......................    2       0    S04
    .........................................................................caccaggagauuaauaugcuu....................    2       0    S04
    .........................................................................caccaggagauuaauaugcuuu...................    1       0    S04
    .........................................................................caccaggagauuaauaugcuuuu..................    1       0    S04

    ....................................cagguauuaaauagcccgguggca......................................................    1       0    S08
    ........................................................................ucaccaggagauuaauaug.......................    1       0    S08
    ........................................................................ucaccaggagauuaauaugcu.....................    1       0    S08
```

```
                                    Star                              Mature
aacugcguuuuauguucauuuaaucuauucaagaccagguauuaaauagcccgguggcagagucguaacugucaccaggagauuaauaugcuuuuaaaccugaggacuagugu
.......................................................................ucaccaggagauuaauaugcuu....................  21  0  S08
.......................................................................ucaccaggagauuaauaugcuuu...................  14  0  S08
.......................................................................ucaccaggagauuaauaugcuuuu..................  3   0  S08
..........................................................................ccaggagauuaauaugcu.....................  1   0  S08
..........................................................................ccaggagauuaauaugcuu....................  1   0  S08

...................................cagguauuaaauagcccgguggc.......................................................  2   0  S03
...................................cagguauuaaauagcccgguggca......................................................  1   0  S03
....................................agguauuaaauagcccgguggc.......................................................  5   0  S03
....................................agguauuaaauagcccgguggcag.....................................................  1   0  S03
.......................................................................ucaccaggagauuaauaug.......................  1   0  S03
.......................................................................ucaccaggagauuaauaugcu.....................  2   0  S03
.......................................................................ucaccaggagauuaauaugcuu....................  39  0  S03
.......................................................................ucaccaggagauuaauaugcuuu...................  24  0  S03
.......................................................................ucaccaggagauuaauaugcuuuu..................  6   0  S03
........................................................................caccaggagauuaauaugcuuu...................  4   0  S03
........................................................................caccaggagauuaauaugcuuuu..................  6   0  S03
........................................................................caccaggagauuaauaugcuuuuaa................  1   0  S03
...........................................................................caggagauuaauaugcuu....................  1   0  S03
...........................................................................caggagauuaauaugcuuu...................  1   0  S03
............................................................................aggagauuaauaugcuuu...................  1   0  S03

.......................................................................ucaccaggagauuaauaug.......................  2   0  S09
.......................................................................ucaccaggagauuaauaugcuu....................  11  0  S09
.......................................................................ucaccaggagauuaauaugcuuu...................  25  0  S09
.......................................................................ucaccaggagauuaauaugcuuuu..................  5   0  S09
........................................................................caccaggagauuaauaugcuuu...................  1   0  S09

.......................................................................ucaccaggagauuaauaug.......................  1   0  S07
.......................................................................ucaccaggagauuaauaugcuu....................  12  0  S07
.......................................................................ucaccaggagauuaauaugcuuu...................  14  0  S07
.......................................................................ucaccaggagauuaauaugcuuuu..................  5   0  S07
........................................................................caccaggagauuaauaugcuu....................  2   0  S07
........................................................................caccaggagauuaauaugcuuuu..................  1   0  S07

....................................agguauuaaauagcccgguggc.......................................................  1   0  S06
.......................................................................ucaccaggagauuaauaug.......................  1   0  S06
.......................................................................ucaccaggagauuaauaugcu.....................  2   0  S06
.......................................................................ucaccaggagauuaauaugcuu....................  43  0  S06
.......................................................................ucaccaggagauuaauaugcuuu...................  21  0  S06
.......................................................................ucaccaggagauuaauaugcuuuu..................  15  0  S06
........................................................................caccaggagauuaauaugcuuuu..................  6   0  S06
........................................................................caccaggagauuaauaugcuuuua.................  1   0  S06
....................................................................................aauaugcuuuuaaaccugaggac......  2   0  S06

...................................cagguauuaaauagcccgguggc.......................................................  1   0  S01
...................................cagguauuaaauagcccgguggca......................................................  1   0  S01
....................................agguauuaaauagcccgguggc.......................................................  8   0  S01
....................................agguauuaaauagcccgguggca......................................................  3   0  S01
....................................agguauuaaauagcccgguggcag.....................................................  2   0  S01
.......................................................................ucaccaggagauuaauaug.......................  5   0  S01
.......................................................................ucaccaggagauuaauaugcuu....................  92  0  S01
.......................................................................ucaccaggagauuaauaugcuuu...................  53  0  S01
.......................................................................ucaccaggagauuaauaugcuuuu..................  21  0  S01
.......................................................................ucaccaggagauuaauaugcuuuua.................  2   0  S01
.......................................................................ucaccaggagauuaauaugcuuuuaa................  1   0  S01
........................................................................caccaggagauuaauaug.......................  2   0  S01
........................................................................caccaggagauuaauaugcuu....................  2   0  S01
........................................................................caccaggagauuaauaugcuuu...................  6   0  S01
........................................................................caccaggagauuaauaugcuuuu..................  6   0  S01
........................................................................caccaggagauuaauaugcuuuua.................  1   0  S01
..........................................................................ccaggagauuaauaugcuu....................  1   0  S01

....................................agguauuaaauagcccggugg........................................................  1   0  S10
....................................agguauuaaauagcccgguggc.......................................................  4   0  S10
.......................................................................ucaccaggagauuaauaug.......................  1   0  S10
.......................................................................ucaccaggagauuaauaugcu.....................  1   0  S10
.......................................................................ucaccaggagauuaauaugcuu....................  28  0  S10
.......................................................................ucaccaggagauuaauaugcuuu...................  82  0  S10
.......................................................................ucaccaggagauuaauaugcuuuu..................  8   0  S10
```

```
                                                     Star                             Mature
aacugcguuuuauguucauuuaaaucuauucaagaccagguauuaaauagcccgguggcagagucguaacugucaccaggagauuaaauaugcuuuuaaaccugaggacuagugu
........................................................................ucaccaggagauuaaauaugcuuuua.................    2    0    S10
.........................................................................caccaggagauuaaauaug.......................    1    0    S10
.........................................................................caccaggagauuaaauaugcuuu...................    7    0    S10
.........................................................................caccaggagauuaaauaugcuuuu..................    6    0    S10

....................................cagguauuaaauagcccgguggc........................................................    2    0    S05
.....................................agguauuaaauagcccgguggc........................................................    1    0    S05
........................................................................ucaccaggagauuaaauaug.......................    5    0    S05
........................................................................ucaccaggagauuaaauaugcuu....................    37   0    S05
........................................................................ucaccaggagauuaaauaugcuuu...................    19   0    S05
........................................................................ucaccaggagauuaaauaugcuuuu..................    4    0    S05
.........................................................................caccaggagauuaaauaugcuuu...................    1    0    S05
.........................................................................caccaggagauuaaauaugcuuuu..................    4    0    S05
.........................................................................caccaggagauuaaauaugcuuuua.................    1    0    S05
```

Provisional ID : Scaffold29666_47930
Score total : 83521.3
Score for star read(s) : 3.9
Score for read counts : 83515.1
Score for mfe : 0.7
Score for randfold : 1.6
Score for cons. seed :
Total read count : 163823
Mature read count : 156487
Loop read count : 0
Star read count : 7336

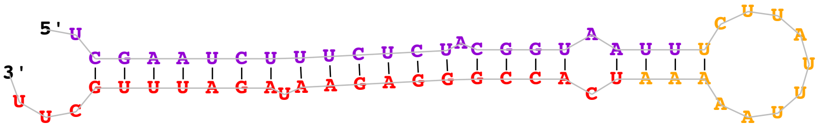

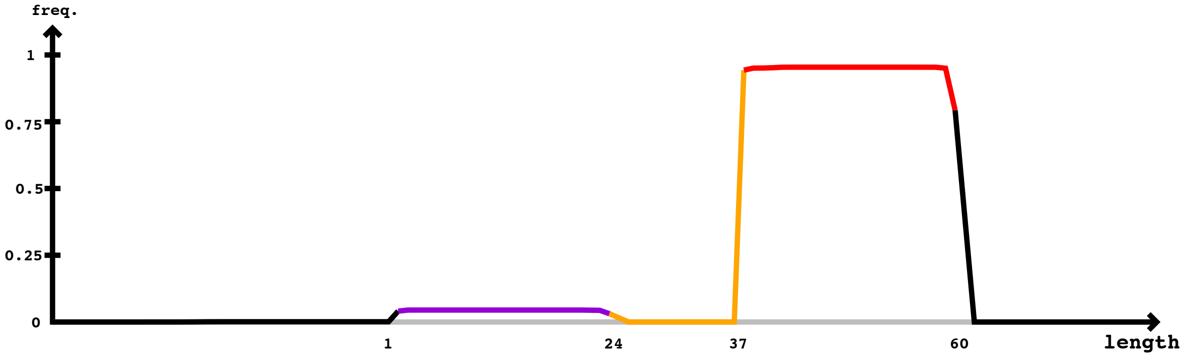


```
                                 Star                              Mature
5'- gggacugaauggaaucgagauugaauggauggaaucgaaucuuucucuacgguaauuucuuauuuaaaaaucaccgggagaauagauuugcuucuauucauaauacuaaccac  -3'    obs
    gggacugaauggaaucgagauugaauggauggaaucgaaucuuucucuacgguaauuucuuauuuaaaaaucaccgggagaauagauuugcuucuauucauaauacuaaccac         exp
    ((..((((......)).))).....((((((((((.(((((((((((((.((((.((((.........)))).)))))))))).))))))).))))))))))........))..   reads      mm      sample
    ......................................................................ucaccgggagaauagauuugcu.....................   4          0       S02
    ......................................................................ucaccgggagaauagauuugcuu....................   30         0       S02

    ............aaucgagauugaauggauggaa...............................................................................   4          0       S04
    .............aucgagauugaauggauggaa...............................................................................   2          0       S04
    ..............ucgagauugaauggauggaa...............................................................................   5          0       S04
    ...............cgagauugaauggauggaa...............................................................................   2          0       S04
    ..................................ucgaaucuuucucuacgguaau.........................................................   9          0       S04
    ..................................ucgaaucuuucucuacgguaauu........................................................   37         0       S04
    ...................................cgaaucuuucucuacgguaau.........................................................   2          0       S04
    ...................................cgaaucuuucucuacgguaauu........................................................   5          0       S04
    ................................................................uaaaaaucaccgggagaauagauuugcuu....................   1          0       S04
    ......................................................................ucaccgggagaauagauuugc......................   15         0       S04
    ......................................................................ucaccgggagaauagauuugcu.....................   665        0       S04
    ......................................................................ucaccgggagaauagauuugcuu....................   4897       0       S04
    ......................................................................ucaccgggagaauagauuugcuuc...................   1          0       S04
    .......................................................................caccgggagaauagauuugcu.....................   2          0       S04
    .......................................................................caccgggagaauagauuugcuu....................   52         0       S04
    .........................................................................ccgggagaauagauuugcuu....................   5          0       S04
    ..........................................................................cgggagaauagauuugcu.....................   1          0       S04
    ..........................................................................cgggagaauagauuugcuu....................   4          0       S04

    ....cugaauggaaucgagauugaauggauggaa...............................................................................   1          0       S03
    ............aaucgagauugaauggauggaa...............................................................................   3          0       S03
    .............aucgagauugaauggauggaa...............................................................................   3          0       S03
    ..............ucgagauugaauggauggaa...............................................................................   7          0       S03
    ...............cgagauugaauggauggaa...............................................................................   2          0       S03
    ...............cgagauugaauggauggaau..............................................................................   1          0       S03
    ..................................ucgaaucuuucucuacgguaau.........................................................   7          0       S03
    ..................................ucgaaucuuucucuacgguaauu........................................................   39         0       S03
    ...................................cgaaucuuucucuacgguaau.........................................................   2          0       S03
    ...................................cgaaucuuucucuacgguaauu........................................................   6          0       S03
    ................................................................uaaaaaucaccgggagaauagauuugcuu....................   2          0       S03
```

Star Mature

gggacugaauggaaucgagauugaauggauggaaucgaaucuuucucuacgguaauuucuuauuuaaaaaucaccgggagaauagauuugcuucuauucauaaauacuaaccac			
....ucaccgggagaauagauuu.........................	1	0	S03
....ucaccgggagaauagauuugc.......................	14	0	S03
....ucaccgggagaauagauuugcu......................	819	0	S03
....ucaccgggagaauagauuugcuu.....................	4206	0	S03
....caccgggagaauagauuugcu......................	2	0	S03
....caccgggagaauagauuugcuu.....................	42	0	S03
....accgggagaauagauuugcuu.....................	1	0	S03
....ccgggagaauagauuugcu......................	1	0	S03
....ccgggagaauagauuugcuu.....................	3	0	S03
....cgggagaauagauuugcu......................	1	0	S03
....cgggagaauagauuugcuu.....................	5	0	S03
....gggagaauagauuugcuu.....................	1	0	S03
...........gaaucgagauugaauggauggaa..	2	0	S09
............aaucgagauugaauggaugga...	1	0	S09
............aaucgagauugaauggauggaa..	2	0	S09
.............aucgagauugaauggauggaa..	5	0	S09
..............ucgagauugaauggauggaa..	10	0	S09
...............cgagauugaauggauggaau...	2	0	S09
..................................ucgaaucuuucucuacggu...	1	0	S09
..................................ucgaaucuuucucuacggua..	14	0	S09
..................................ucgaaucuuucucuacgguaa...	12	0	S09
..................................ucgaaucuuucucuacgguaau..	474	0	S09
..................................ucgaaucuuucucuacgguaauu...	1243	0	S09
..................................ucgaaucuuucucuacgguaauuu..	1	0	S09
..................................ucgaaucuuucucuacgguaauuucuuauuuaaaa...	1	0	S09
..................................ucgaaucuuucucuacgguaauuucuuauuuaaaaa..	1	0	S09
...................................cgaaucuuucucuacggua..	1	0	S09
...................................cgaaucuuucucuacgguaau..	21	0	S09
...................................cgaaucuuucucuacgguaauu...	110	0	S09
....................................gaaucuuucucuacgguaau..	1	0	S09
....................................gaaucuuucucuacgguaauu...	2	0	S09
....uacgguaauuucuuauuuaaaa...	1	0	S09
....cuuauuuaaaaaucaccgggagaauagauuugcuu.....................	1	0	S09
....uaaaaaucaccgggagaauagauuugcuu.....................	1	0	S09
....aucaccgggagaauagauuugcuu.....................	2	0	S09
....ucaccgggagaauagauu..........................	3	0	S09
....ucaccgggagaauagauuu.........................	2	0	S09
....ucaccgggagaauagauuug........................	2	0	S09
....ucaccgggagaauagauuugc.......................	136	0	S09
....ucaccgggagaauagauuugcu......................	5495	0	S09
....ucaccgggagaauagauuugcuu.....................	23815	0	S09
....ucaccgggagaauagauuugcuuc....................	6	0	S09
....caccgggagaauagauuugc.......................	1	0	S09
....caccgggagaauagauuugcu......................	15	0	S09
....caccgggagaauagauuugcuu.....................	211	0	S09
....accgggagaauagauuugcu......................	1	0	S09
....accgggagaauagauuugcuu.....................	3	0	S09
....ccgggagaauagauuugcu......................	4	0	S09
....ccgggagaauagauuugcuu.....................	12	0	S09
....cgggagaauagauuugcu......................	2	0	S09
....cgggagaauagauuugcuu.....................	25	0	S09
....gggagaauagauuugcuu.....................	6	0	S09
gggacugaauggaaucgagauugaauggauggaa..	2	0	S08
...acugaauggaaucgagauugaauggauggaa..	1	0	S08
.....ugaauggaaucgagauugaauggauggaa..	1	0	S08
......gaauggaaucgagauugaauggauggaa..	1	0	S08
............aaucgagauugaauggauggaa..	3	0	S08
.............aucgagauugaauggauggaa..	3	0	S08
..............ucgagauugaauggauggaa..	10	0	S08
..............ucgagauugaauggauggaau...	1	0	S08
...............cgagauugaauggauggaa..	5	0	S08
................gagauugaauggauggaa..	2	0	S08
..................................ucgaaucuuucucuacggu...	1	0	S08
..................................ucgaaucuuucucuacggua..	18	0	S08
..................................ucgaaucuuucucuacgguaa...	32	0	S08
..................................ucgaaucuuucucuacgguaau..	370	0	S08
..................................ucgaaucuuucucuacgguaauu...	715	0	S08
..................................ucgaaucuuucucuacgguaauuu..	2	0	S08

```
                                     Star                                   Mature
gggacugaauggaaucgagauugaauggauggaaucgaaucuuucucuacgguaauuucuuauuuaaaaaucaccgggagaauagauuugcuucuauucauaaauacuaaccac
..................................ucgaaucuuucucuacgguaauuucuua....................................................	1	0	S08
..................................ucgaaucuuucucuacgguaauuucuuauu..................................................	2	0	S08
..................................ucgaaucuuucucuacgguaauuucuuauuuaa...............................................	2	0	S08
...................................cgaaucuuucucuacggua............................................................	5	0	S08
...................................cgaaucuuucucuacgguaa...........................................................	2	0	S08
...................................cgaaucuuucucuacgguaau..........................................................	42	0	S08
...................................cgaaucuuucucuacgguaauu.........................................................	111	0	S08
.........................................................ucuuauuuaaaaaucaccgggagaauagauuugcuu.....................	1	0	S08
................................................................uaaaaaucaccgggagaauagauuugcuu.....................	3	0	S08
....................................................................aaucaccgggagaauagauuugcu......................	1	0	S08
.....................................................................aucaccgggagaauagauuugcuu.....................	2	0	S08
......................................................................ucaccgggagaauagauuu.........................	3	0	S08
......................................................................ucaccgggagaauagauuugc.......................	114	0	S08
......................................................................ucaccgggagaauagauuugcu......................	5406	0	S08
......................................................................ucaccgggagaauagauuugcuu.....................	23069	0	S08
......................................................................ucaccgggagaauagauuugcuuc....................	5	0	S08
.......................................................................caccgggagaauagauuugc.......................	1	0	S08
.......................................................................caccgggagaauagauuugcu......................	10	0	S08
.......................................................................caccgggagaauagauuugcuu.....................	178	0	S08
........................................................................accgggagaauagauuugcu......................	2	0	S08
........................................................................accgggagaauagauuugcuu.....................	8	0	S08
.........................................................................ccgggagaauagauuugcu......................	18	0	S08
.........................................................................ccgggagaauagauuugcuu.....................	64	0	S08
..........................................................................cgggagaauagauuugcu......................	23	0	S08
..........................................................................cgggagaauagauuugcuu.....................	105	0	S08
...........................................................................gggagaauagauuugcuu.....................	15	0	S08

............aaucgagauugaauggauggaa................................................................................	2	0	S06
.............aucgagauugaauggauggaa................................................................................	3	0	S06
..............ucgagauugaauggauggaa................................................................................	12	0	S06
...............cgagauugaauggauggaa................................................................................	4	0	S06
..................................ucgaaucuuucucuacgg..............................................................	3	0	S06
..................................ucgaaucuuucucuacggua............................................................	2	0	S06
..................................ucgaaucuuucucuacgguaa...........................................................	9	0	S06
..................................ucgaaucuuucucuacgguaau..........................................................	62	0	S06
..................................ucgaaucuuucucuacgguaauu.........................................................	98	0	S06
...................................cgaaucuuucucuacggua............................................................	1	0	S06
...................................cgaaucuuucucuacgguaa...........................................................	2	0	S06
...................................cgaaucuuucucuacgguaau..........................................................	6	0	S06
...................................cgaaucuuucucuacgguaauu.........................................................	14	0	S06
...................................cgaaucuuucucuacgguaauuu........................................................	1	0	S06
....................................gaaucuuucucuacgguaauu.........................................................	1	0	S06
................................................................uaaaaaucaccgggagaauagauuugcuu.....................	2	0	S06
......................................................................ucaccgggagaauagauuug........................	1	0	S06
......................................................................ucaccgggagaauagauuugc.......................	27	0	S06
......................................................................ucaccgggagaauagauuugcu......................	1365	0	S06
......................................................................ucaccgggagaauagauuugcuu.....................	9085	0	S06
......................................................................ucaccgggagaauagauuugcuuc....................	2	0	S06
.......................................................................caccgggagaauagauuugcu......................	6	0	S06
.......................................................................caccgggagaauagauuugcuu.....................	83	0	S06
........................................................................accgggagaauagauuugcu......................	1	0	S06
........................................................................accgggagaauagauuugcuu.....................	3	0	S06
.........................................................................ccgggagaauagauuugcu......................	2	0	S06
.........................................................................ccgggagaauagauuugcuu.....................	15	0	S06
..........................................................................cgggagaauagauuugcu......................	1	0	S06
..........................................................................cgggagaauagauuugcuu.....................	22	0	S06
...........................................................................gggagaauagauuugcuu.....................	1	0	S06

......................................................................ucaccgggagaauagauuugcu......................	3	0	S01
......................................................................ucaccgggagaauagauuugcuu.....................	8	0	S01

........auggaaucgagauugaauggauggaa................................................................................	1	0	S07
............aaucgagauugaauggauggaa................................................................................	6	0	S07
.............aucgagauugaauggauggaa................................................................................	5	0	S07
..............ucgagauugaauggauggaa................................................................................	6	0	S07
...............cgagauugaauggaugga.................................................................................	1	0	S07
...............cgagauugaauggauggaa................................................................................	3	0	S07
..................................ucgaaucuuucucuacgg..............................................................	1	0	S07
..................................ucgaaucuuucucuacggu.............................................................	1	0	S07
..................................ucgaaucuuucucuacggua............................................................	23	0	S07
```

Star Mature

```
gggacugaauggaaucgagauugaauggauggaaucgaaucuuucucuacgguaauuucuuauuuaaaaaucaccgggagaauagauuugcuucuauucauaaauacuaaccac
..................................ucgaaucuuucucuacgguaa...........................................................	32	0	S07
..................................ucgaaucuuucucuacgguaau..........................................................	838	0	S07
..................................ucgaaucuuucucuacgguaauu.........................................................	1897	0	S07
..................................ucgaaucuuucucuacgguaauuu........................................................	2	0	S07
..................................ucgaaucuuucucuacgguaauuucuu.....................................................	2	0	S07
..................................ucgaaucuuucucuacgguaauuucuuauuuaaaaa............................................	5	0	S07
..................................ucgaaucuuucucuacgguaauuucuuauuuaaaaau...........................................	1	0	S07
...................................cgaaucuuucucuacggua............................................................	2	0	S07
...................................cgaaucuuucucuacgguaa...........................................................	2	0	S07
...................................cgaaucuuucucuacgguaau..........................................................	49	0	S07
...................................cgaaucuuucucuacgguaauu.........................................................	221	0	S07
....................................gaaucuuucucuacgguaauu.........................................................	5	0	S07
..........................................................cuuauuuaaaaaucaccgggagaauagauuugcuu.....................	1	0	S07
..............................................................uuuaaaaaucaccgggagaauagauuugcuu.....................	1	0	S07
................................................................uaaaaaucaccgggagaauagauuugcuu.....................	2	0	S07
...................................................................aaaucaccgggagaauagauuugcuu.....................	1	0	S07
.....................................................................aucaccgggagaauagauuugcuu.....................	2	0	S07
......................................................................ucaccgggagaauagauu..........................	2	0	S07
......................................................................ucaccgggagaauagauuu.........................	1	0	S07
......................................................................ucaccgggagaauagauuug........................	3	0	S07
......................................................................ucaccgggagaauagauuugc.......................	87	0	S07
......................................................................ucaccgggagaauagauuugcu......................	3188	0	S07
......................................................................ucaccgggagaauagauuugcuu.....................	19106	0	S07
......................................................................ucaccgggagaauagauuugcuuc....................	1	0	S07
.......................................................................caccgggagaauagauuugc.......................	1	0	S07
.......................................................................caccgggagaauagauuugcu......................	19	0	S07
.......................................................................caccgggagaauagauuugcuu.....................	178	0	S07
........................................................................accgggagaauagauuugcu......................	1	0	S07
........................................................................accgggagaauagauuugcuu.....................	5	0	S07
.........................................................................ccgggagaauagauuugcu......................	2	0	S07
.........................................................................ccgggagaauagauuugcuu.....................	26	0	S07
..........................................................................cgggagaauagauuugcu......................	5	0	S07
..........................................................................cgggagaauagauuugcuu.....................	43	0	S07
..........................................................................cgggagaauagauuugcuuc....................	1	0	S07
...........................................................................gggagaauagauuugcuu.....................	5	0	S07

........auggaaucgagauugaauggauggaa................................................................................	1	0	S05
.............aucgagauugaauggauggaa................................................................................	2	0	S05
..............ucgagauugaauggauggaa................................................................................	1	0	S05
..................................ucgaaucuuucucuacgguaau..........................................................	11	0	S05
..................................ucgaaucuuucucuacgguaauu.........................................................	40	0	S05
...................................cgaaucuuucucuacggua............................................................	1	0	S05
...................................cgaaucuuucucuacgguaa...........................................................	1	0	S05
...................................cgaaucuuucucuacgguaauu.........................................................	4	0	S05
....................................................auuuaaaaaucaccgggagaauagauuugcuu.....................	1	0	S05
......................................................................ucaccgggagaauagauuug........................	1	0	S05
......................................................................ucaccgggagaauagauuugc.......................	11	0	S05
......................................................................ucaccgggagaauagauuugcu......................	705	0	S05
......................................................................ucaccgggagaauagauuugcuu.....................	4966	0	S05
......................................................................ucaccgggagaauagauuugcuuc....................	1	0	S05
.......................................................................caccgggagaauagauuugcuu.....................	47	0	S05
.........................................................................ccgggagaauagauuugcuu.....................	8	0	S05
..........................................................................cgggagaauagauuugcuu.....................	11	0	S05
...........................................................................gggagaauagauuugcuu.....................	2	0	S05

gggacugaauggaaucgagauugaauggauggaa................................................................................	1	0	S10
..........ggaaucgagauugaauggauggaa................................................................................	1	0	S10
...........gaaucgagauugaauggauggaa................................................................................	1	0	S10
............aaucgagauugaauggauggaa................................................................................	4	0	S10
.............aucgagauugaauggauggaa................................................................................	7	0	S10
..............ucgagauugaauggauggaa................................................................................	9	0	S10
..............ucgagauugaauggauggaau...............................................................................	2	0	S10
...............cgagauugaauggauggaa................................................................................	3	0	S10
................gagauugaauggauggaa................................................................................	1	0	S10
..................................ucgaaucuuucucuacggu.............................................................	1	0	S10
..................................ucgaaucuuucucuacggua............................................................	5	0	S10
..................................ucgaaucuuucucuacgguaa...........................................................	5	0	S10
..................................ucgaaucuuucucuacgguaau..........................................................	166	0	S10
..................................ucgaaucuuucucuacgguaauu.........................................................	496	0	S10
..................................ucgaaucuuucucuacgguaauuu........................................................	1	0	S10
```

Star Mature

```
gggacugaauggaaucgagauugaauggauggaaucgaaucuuucucuacgguaauuucuuauuuaaaaaucaccgggagaauagauuugcuucuauucauaaauacuaaccac
..................................ucgaaucuuucucuacgguaauuucuuauuuaaaaau...........................................	1	0	S10
...................................cgaaucuuucucuacgguaau..........................................................	6	0	S10
...................................cgaaucuuucucuacgguaauu.........................................................	38	0	S10
....................................gaaucuuucucuacgguaauu.........................................................	1	0	S10
.....................................aaucuuucucuacgguaauuucuuauuua................................................	1	0	S10
.................................................................aaaaaucaccgggagaauagauuugcu......................	1	0	S10
..................................................................aaaaucaccgggagaauagauuugcuu.....................	1	0	S10
.....................................................................aucaccgggagaauagauuugcu......................	1	0	S10
.....................................................................aucaccgggagaauagauuugcuu.....................	3	0	S10
......................................................................ucaccgggagaauagauu..........................	2	0	S10
......................................................................ucaccgggagaauagauuu.........................	3	0	S10
......................................................................ucaccgggagaauagauuug........................	4	0	S10
......................................................................ucaccgggagaauagauuugc.......................	213	0	S10
......................................................................ucaccgggagaauagauuugcu......................	8018	0	S10
......................................................................ucaccgggagaauagauuugcuu.....................	39152	0	S10
......................................................................ucaccgggagaauagauuugcuuc....................	11	0	S10
.......................................................................caccgggagaauagauuugc.......................	2	0	S10
.......................................................................caccgggagaauagauuugcu......................	30	0	S10
.......................................................................caccgggagaauagauuugcuu.....................	366	0	S10
........................................................................accgggagaauagauuug........................	1	0	S10
........................................................................accgggagaauagauuugcu......................	2	0	S10
........................................................................accgggagaauagauuugcuu.....................	6	0	S10
.........................................................................ccgggagaauagauuugcu......................	5	0	S10
.........................................................................ccgggagaauagauuugcuu.....................	25	0	S10
..........................................................................cgggagaauagauuugcu......................	6	0	S10
..........................................................................cgggagaauagauuugcuu.....................	35	0	S10
...........................................................................gggagaauagauuugcuu.....................	12	0	S10
```

Provisional ID : Scaffold5285_44883
Score total : 83521.3
Score for star read(s) : 3.9
Score for read counts : 83515.1
Score for mfe : 0.7
Score for randfold : 1.6
Score for cons. seed :
Total read count : 163823
Mature read count : 156487
Loop read count : 0
Star read count : 7336

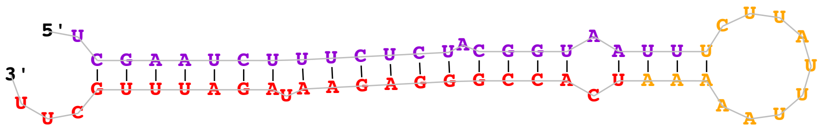

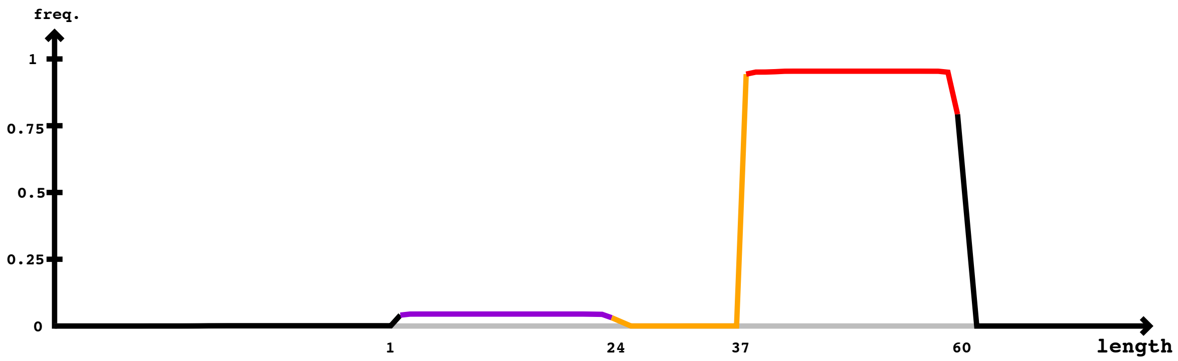

Star Mature

```
5'- gggacugaauggaaucgagauugaauggauggaaucgaaucuuucucuacgguaauuucuuauuuaaaaaucaccgggagaauagauuugcuucuauucauaauacuaacca  -3'   obs
    gggacugaauggaaucgagauugaauggauggaaucgaaucuuucucuacgguaauuucuuauuuaaaaaucaccgggagaauagauuugcuucuauucauaauacuaacca        exp
    ((..((((......)).)).....((((((((((.((((((((((((.((((.((((.........)))).))))))))))).))))))).))))))))))........)).   reads    mm    sample
    ........auggaaucgagauugaauggauggaa..............................................................................   1        0     S07
    ............aaucgagauugaauggauggaa..............................................................................   6        0     S07
    .............aucgagauugaauggauggaa..............................................................................   5        0     S07
    ..............ucgagauugaauggauggaa..............................................................................   6        0     S07
    ...............cgagauugaauggaugga...............................................................................   1        0     S07
    ...............cgagauugaauggauggaa..............................................................................   3        0     S07
    ..................................ucgaaucuuucucuacgg............................................................   1        0     S07
    ..................................ucgaaucuuucucuacggu...........................................................   1        0     S07
    ..................................ucgaaucuuucucuacggua..........................................................   23       0     S07
    ..................................ucgaaucuuucucuacgguaa.........................................................   32       0     S07
    ..................................ucgaaucuuucucuacgguaau........................................................   838      0     S07
    ..................................ucgaaucuuucucuacgguaauu.......................................................   1897     0     S07
    ..................................ucgaaucuuucucuacgguaauuu......................................................   2        0     S07
    ..................................ucgaaucuuucucuacgguaauuucuu...................................................   2        0     S07
    ..................................ucgaaucuuucucuacgguaauuucuuauuuaaaaa..........................................   5        0     S07
    ..................................ucgaaucuuucucuacgguaauuucuuauuuaaaaau.........................................   1        0     S07
    ...................................cgaaucuuucucuacggua..........................................................   2        0     S07
    ...................................cgaaucuuucucuacgguaa.........................................................   2        0     S07
    ...................................cgaaucuuucucuacgguaau........................................................   49       0     S07
    ...................................cgaaucuuucucuacgguaauu.......................................................   221      0     S07
    ....................................gaaucuuucucuacgguaauu.......................................................   5        0     S07
    ..........................................................cuuauuuaaaaaucaccgggagaauagauuugcuu...................   1        0     S07
    ..............................................................uuuaaaaaucaccgggagaauagauuugcuu...................   1        0     S07
    ................................................................uaaaaaucaccgggagaauagauuugcuu...................   2        0     S07
    ...................................................................aaaucaccgggagaauagauuugcuu...................   1        0     S07
    .....................................................................aucaccgggagaauagauuugcuu...................   2        0     S07
    ......................................................................ucaccgggagaauagauu........................   2        0     S07
    ......................................................................ucaccgggagaauagauuu.......................   1        0     S07
    ......................................................................ucaccgggagaauagauuug......................   3        0     S07
    ......................................................................ucaccgggagaauagauuugc.....................   87       0     S07
    ......................................................................ucaccgggagaauagauuugcu....................   3188     0     S07
    ......................................................................ucaccgggagaauagauuugcuu...................   19106    0     S07
    ......................................................................ucaccgggagaauagauuugcuuc..................   1        0     S07
```

Star Mature

```
gggacugaauggaaucgagauugaauggauggaaucgaaucuuucucuacgguaauuucuuauuuaaaaaucaccgggagaauagauuugcuucuauucauaaauacuaacca
.......................................................................caccgggagaauagauuugc......................	1	0	S07
.......................................................................caccgggagaauagauuugcu.....................	19	0	S07
.......................................................................caccgggagaauagauuugcuu....................	178	0	S07
........................................................................accgggagaauagauuugcu.....................	1	0	S07
........................................................................accgggagaauagauuugcuu....................	5	0	S07
.........................................................................ccgggagaauagauuugcu.....................	2	0	S07
.........................................................................ccgggagaauagauuugcuu....................	26	0	S07
..........................................................................cgggagaauagauuugcu.....................	5	0	S07
..........................................................................cgggagaauagauuugcuu....................	43	0	S07
..........................................................................cgggagaauagauuugcuuc...................	1	0	S07
...........................................................................gggagaauagauuugcuu....................	5	0	S07

............aaucgagauugaauggauggaa...............................................................................	2	0	S06
.............aucgagauugaauggauggaa...............................................................................	3	0	S06
..............ucgagauugaauggauggaa...............................................................................	12	0	S06
...............cgagauugaauggauggaa...............................................................................	4	0	S06
..................................ucgaaucuuucucuacgg.............................................................	3	0	S06
..................................ucgaaucuuucucuacggua...........................................................	2	0	S06
..................................ucgaaucuuucucuacgguaa..........................................................	9	0	S06
..................................ucgaaucuuucucuacgguaau.........................................................	62	0	S06
..................................ucgaaucuuucucuacgguaauu........................................................	98	0	S06
...................................cgaaucuuucucuacggua...........................................................	1	0	S06
...................................cgaaucuuucucuacgguaa..........................................................	2	0	S06
...................................cgaaucuuucucuacgguaau.........................................................	6	0	S06
...................................cgaaucuuucucuacgguaauu........................................................	14	0	S06
...................................cgaaucuuucucuacgguaauuu.......................................................	1	0	S06
....................................gaaucuuucucuacgguaauu........................................................	1	0	S06
................................................................uaaaaaucaccgggagaauagauuugcuu....................	2	0	S06
......................................................................ucaccgggagaauagauuug.......................	1	0	S06
......................................................................ucaccgggagaauagauuugc......................	27	0	S06
......................................................................ucaccgggagaauagauuugcu.....................	1365	0	S06
......................................................................ucaccgggagaauagauuugcuu....................	9085	0	S06
......................................................................ucaccgggagaauagauuugcuuc...................	2	0	S06
.......................................................................caccgggagaauagauuugcu.....................	6	0	S06
.......................................................................caccgggagaauagauuugcuu....................	83	0	S06
........................................................................accgggagaauagauuugcu.....................	1	0	S06
........................................................................accgggagaauagauuugcuu....................	3	0	S06
.........................................................................ccgggagaauagauuugcu.....................	2	0	S06
.........................................................................ccgggagaauagauuugcuu....................	15	0	S06
..........................................................................cgggagaauagauuugcu.....................	1	0	S06
..........................................................................cgggagaauagauuugcuu....................	22	0	S06
...........................................................................gggagaauagauuugcuu....................	1	0	S06

......................................................................ucaccgggagaauagauuugcu.....................	3	0	S01
......................................................................ucaccgggagaauagauuugcuu....................	8	0	S01

gggacugaauggaaucgagauugaauggauggaa...............................................................................	1	0	S10
..........ggaaucgagauugaauggauggaa...............................................................................	1	0	S10
...........gaaucgagauugaauggauggaa...............................................................................	1	0	S10
............aaucgagauugaauggauggaa...............................................................................	4	0	S10
.............aucgagauugaauggauggaa...............................................................................	7	0	S10
..............ucgagauugaauggauggaa...............................................................................	9	0	S10
..............ucgagauugaauggauggaau..............................................................................	2	0	S10
...............cgagauugaauggauggaa...............................................................................	3	0	S10
................gagauugaauggauggaa...............................................................................	1	0	S10
..................................ucgaaucuuucucuacggu............................................................	1	0	S10
..................................ucgaaucuuucucuacggua...........................................................	5	0	S10
..................................ucgaaucuuucucuacgguaa..........................................................	5	0	S10
..................................ucgaaucuuucucuacgguaau.........................................................	166	0	S10
..................................ucgaaucuuucucuacgguaauu........................................................	496	0	S10
..................................ucgaaucuuucucuacgguaauuu.......................................................	1	0	S10
..................................ucgaaucuuucucuacgguaauuucuuauuuaaaaau..........................................	1	0	S10
...................................cgaaucuuucucuacgguaau.........................................................	6	0	S10
...................................cgaaucuuucucuacgguaauu........................................................	38	0	S10
....................................gaaucuuucucuacgguaauu........................................................	1	0	S10
.....................................aaucuuucucuacgguaauuucuuauuua...............................................	1	0	S10
.................................................................aaaaaucaccgggagaauagauuugcu.....................	1	0	S10
..................................................................aaaaucaccgggagaauagauuugcuu....................	1	0	S10
.....................................................................aucaccgggagaauagauuugcu.....................	1	0	S10
.....................................................................aucaccgggagaauagauuugcuu....................	3	0	S10
```

```
                                  Star                                Mature
gggacugaauggaaucgagauugaauggauggaaucgaaucuuucucuacgguaauuucuuauuuaaaaaucaccgggagaauagauuugcuucuauucauaauacuaacca
......................................................................ucaccgggagaauagauu........................   2      0   S10
......................................................................ucaccgggagaauagauuu.......................   3      0   S10
......................................................................ucaccgggagaauagauuug......................   4      0   S10
......................................................................ucaccgggagaauagauuugc.....................   213    0   S10
......................................................................ucaccgggagaauagauuugcu....................   8018   0   S10
......................................................................ucaccgggagaauagauuugcuu...................   39152  0   S10
......................................................................ucaccgggagaauagauuugcuuc..................   11     0   S10
.......................................................................caccgggagaauagauuugc.....................   2      0   S10
.......................................................................caccgggagaauagauuugcu....................   30     0   S10
.......................................................................caccgggagaauagauuugcuu...................   366    0   S10
........................................................................accgggagaauagauuug......................   1      0   S10
........................................................................accgggagaauagauuugcu....................   2      0   S10
........................................................................accgggagaauagauuugcuu...................   6      0   S10
.........................................................................ccgggagaauagauuugcu....................   5      0   S10
.........................................................................ccgggagaauagauuugcuu...................   25     0   S10
..........................................................................cgggagaauagauuugcu....................   6      0   S10
..........................................................................cgggagaauagauuugcuu...................   35     0   S10
...........................................................................gggagaauagauuugcuu...................   12     0   S10

........auggaaucgagauugaauggauggaa..............................................................................   1      0   S05
.............aucgagauugaauggauggaa..............................................................................   2      0   S05
..............ucgagauugaauggauggaa..............................................................................   1      0   S05
..................................ucgaaucuuucucuacgguaau........................................................   11     0   S05
..................................ucgaaucuuucucuacgguaauu.......................................................   40     0   S05
...................................cgaaucuuucucuacggua..........................................................   1      0   S05
...................................cgaaucuuucucuacgguaa.........................................................   1      0   S05
...................................cgaaucuuucucuacgguaauu.......................................................   4      0   S05
.............................................................auuuaaaaaucaccgggagaauagauuugcuu...................   1      0   S05
......................................................................ucaccgggagaauagauuug......................   1      0   S05
......................................................................ucaccgggagaauagauuugc.....................   11     0   S05
......................................................................ucaccgggagaauagauuugcu....................   705    0   S05
......................................................................ucaccgggagaauagauuugcuu...................   4966   0   S05
......................................................................ucaccgggagaauagauuugcuuc..................   1      0   S05
.......................................................................caccgggagaauagauuugcuu...................   47     0   S05
.........................................................................ccgggagaauagauuugcuu...................   8      0   S05
..........................................................................cgggagaauagauuugcuu...................   11     0   S05
...........................................................................gggagaauagauuugcuu...................   2      0   S05

............aaucgagauugaauggauggaa..............................................................................   4      0   S04
.............aucgagauugaauggauggaa..............................................................................   2      0   S04
..............ucgagauugaauggauggaa..............................................................................   5      0   S04
...............cgagauugaauggauggaa..............................................................................   2      0   S04
..................................ucgaaucuuucucuacgguaau........................................................   9      0   S04
..................................ucgaaucuuucucuacgguaauu.......................................................   37     0   S04
...................................cgaaucuuucucuacgguaau........................................................   2      0   S04
...................................cgaaucuuucucuacgguaauu.......................................................   5      0   S04
................................................................uaaaaaucaccgggagaauagauuugcuu...................   1      0   S04
......................................................................ucaccgggagaauagauuugc.....................   15     0   S04
......................................................................ucaccgggagaauagauuugcu....................   665    0   S04
......................................................................ucaccgggagaauagauuugcuu...................   4897   0   S04
......................................................................ucaccgggagaauagauuugcuuc..................   1      0   S04
.......................................................................caccgggagaauagauuugcu....................   2      0   S04
.......................................................................caccgggagaauagauuugcuu...................   52     0   S04
.........................................................................ccgggagaauagauuugcuu...................   5      0   S04
..........................................................................cgggagaauagauuugcu....................   1      0   S04
..........................................................................cgggagaauagauuugcuu...................   4      0   S04

......................................................................ucaccgggagaauagauuugcu....................   4      0   S02
......................................................................ucaccgggagaauagauuugcuu...................   30     0   S02

gggacugaauggaaucgagauugaauggauggaa..............................................................................   2      0   S08
...acugaauggaaucgagauugaauggauggaa..............................................................................   1      0   S08
.....ugaauggaaucgagauugaauggauggaa..............................................................................   1      0   S08
......gaauggaaucgagauugaauggauggaa..............................................................................   1      0   S08
............aaucgagauugaauggauggaa..............................................................................   3      0   S08
.............aucgagauugaauggauggaa..............................................................................   3      0   S08
..............ucgagauugaauggauggaa..............................................................................   10     0   S08
..............ucgagauugaauggauggaau.............................................................................   1      0   S08
...............cgagauugaauggauggaa..............................................................................   5      0   S08
................gagauugaauggauggaa..............................................................................   2      0   S08
```

```
                                  Star                                  Mature
gggacugaauggaaucgagauugaauggauggaaucgaaucuuucucuacgguaauuucuuauuuaaaaaucaccgggagaauagauuugcuucuauucauaaauacuaacca
..................................ucgaaucuuucucuacggu............................................................   1      0   S08
..................................ucgaaucuuucucuacggua...........................................................   18     0   S08
..................................ucgaaucuuucucuacgguaa..........................................................   32     0   S08
..................................ucgaaucuuucucuacgguaau.........................................................   370    0   S08
..................................ucgaaucuuucucuacgguaauu........................................................   715    0   S08
..................................ucgaaucuuucucuacgguaauuu.......................................................   2      0   S08
..................................ucgaaucuuucucuacgguaauuucuua...................................................   1      0   S08
..................................ucgaaucuuucucuacgguaauuucuuauu.................................................   2      0   S08
..................................ucgaaucuuucucuacgguaauuucuuauuuaa..............................................   2      0   S08
...................................cgaaucuuucucuacggua...........................................................   5      0   S08
...................................cgaaucuuucucuacgguaa..........................................................   2      0   S08
...................................cgaaucuuucucuacgguaau.........................................................   42     0   S08
...................................cgaaucuuucucuacgguaauu........................................................   111    0   S08
.........................................................ucuuauuuaaaaaucaccgggagaauagauuugcuu....................   1      0   S08
................................................................uaaaaaucaccgggagaauagauuugcuu....................   3      0   S08
....................................................................aaucaccgggagaauagauuugcu.....................   1      0   S08
.....................................................................aucaccgggagaauagauuugcuu....................   2      0   S08
......................................................................ucaccgggagaauagauuu........................   3      0   S08
......................................................................ucaccgggagaauagauuugc......................   114    0   S08
......................................................................ucaccgggagaauagauuugcu.....................   5406   0   S08
......................................................................ucaccgggagaauagauuugcuu....................   23069  0   S08
......................................................................ucaccgggagaauagauuugcuuc...................   5      0   S08
.......................................................................caccgggagaauagauuugc......................   1      0   S08
.......................................................................caccgggagaauagauuugcu.....................   10     0   S08
.......................................................................caccgggagaauagauuugcuu....................   178    0   S08
........................................................................accgggagaauagauuugcu.....................   2      0   S08
........................................................................accgggagaauagauuugcuu....................   8      0   S08
.........................................................................ccgggagaauagauuugcu.....................   18     0   S08
.........................................................................ccgggagaauagauuugcuu....................   64     0   S08
..........................................................................cgggagaauagauuugcu.....................   23     0   S08
..........................................................................cgggagaauagauuugcuu....................   105    0   S08
...........................................................................gggagaauagauuugcuu....................   15     0   S08

...........gaaucgagauugaauggauggaa...............................................................................   2      0   S09
............aaucgagauugaauggaugga................................................................................   1      0   S09
............aaucgagauugaauggauggaa...............................................................................   2      0   S09
.............aucgagauugaauggauggaa...............................................................................   5      0   S09
..............ucgagauugaauggauggaa...............................................................................   10     0   S09
...............cgagauugaauggauggaau..............................................................................   2      0   S09
..................................ucgaaucuuucucuacggu............................................................   1      0   S09
..................................ucgaaucuuucucuacggua...........................................................   14     0   S09
..................................ucgaaucuuucucuacgguaa..........................................................   12     0   S09
..................................ucgaaucuuucucuacgguaau.........................................................   474    0   S09
..................................ucgaaucuuucucuacgguaauu........................................................   1243   0   S09
..................................ucgaaucuuucucuacgguaauuu.......................................................   1      0   S09
..................................ucgaaucuuucucuacgguaauuucuuauuuaaa.............................................   1      0   S09
..................................ucgaaucuuucucuacgguaauuucuuauuuaaaaa...........................................   1      0   S09
...................................cgaaucuuucucuacggua...........................................................   1      0   S09
...................................cgaaucuuucucuacgguaau.........................................................   21     0   S09
...................................cgaaucuuucucuacgguaauu........................................................   110    0   S09
....................................gaaucuuucucuacgguaau.........................................................   1      0   S09
....................................gaaucuuucucuacgguaauu........................................................   2      0   S09
...............................................uacgguaauuucuuauuuaaaa............................................   1      0   S09
..........................................................cuuauuuaaaaaucaccgggagaauagauuugcuu....................   1      0   S09
................................................................uaaaaaucaccgggagaauagauuugcuu....................   1      0   S09
.....................................................................aucaccgggagaauagauuugcuu....................   2      0   S09
......................................................................ucaccgggagaauagauu.........................   3      0   S09
......................................................................ucaccgggagaauagauuu........................   2      0   S09
......................................................................ucaccgggagaauagauuug.......................   2      0   S09
......................................................................ucaccgggagaauagauuugc......................   136    0   S09
......................................................................ucaccgggagaauagauuugcu.....................   5495   0   S09
......................................................................ucaccgggagaauagauuugcuu....................   23815  0   S09
......................................................................ucaccgggagaauagauuugcuuc...................   6      0   S09
.......................................................................caccgggagaauagauuugc......................   1      0   S09
.......................................................................caccgggagaauagauuugcu.....................   15     0   S09
.......................................................................caccgggagaauagauuugcuu....................   211    0   S09
........................................................................accgggagaauagauuugcu.....................   1      0   S09
........................................................................accgggagaauagauuugcuu....................   3      0   S09
.........................................................................ccgggagaauagauuugcu.....................   4      0   S09
.........................................................................ccgggagaauagauuugcuu....................   12     0   S09
```

Star Mature

```
gggacugaauggaaucgagauugaauggauggaaucgaaucuuucucuacgguaauuucuuauuuaaaaaucaccgggagaauagauuugcuucuauucauaaauacuaacca
..........................................................................cgggagaauagauuugcu.....................	2	0	S09
..........................................................................cgggagaauagauuugcuu....................	25	0	S09
...........................................................................gggagaauagauuugcuu....................	6	0	S09

....cugaauggaaucgagauugaauggauggaa...............................................................................	1	0	S03
............aaucgagauugaauggauggaa...............................................................................	3	0	S03
.............aucgagauugaauggauggaa...............................................................................	3	0	S03
..............ucgagauugaauggauggaa...............................................................................	7	0	S03
...............cgagauugaauggauggaa...............................................................................	2	0	S03
...............cgagauugaauggauggaau..............................................................................	1	0	S03
..................................ucgaaucuuucucuacgguaau.........................................................	7	0	S03
..................................ucgaaucuuucucuacgguaauu........................................................	39	0	S03
...................................cgaaucuuucucuacgguaau.........................................................	2	0	S03
...................................cgaaucuuucucuacgguaauu........................................................	6	0	S03
................................................................uaaaaaucaccgggagaauagauuugcuu....................	2	0	S03
......................................................................ucaccgggagaauagauuu........................	1	0	S03
......................................................................ucaccgggagaauagauuugc......................	14	0	S03
......................................................................ucaccgggagaauagauuugcu.....................	819	0	S03
......................................................................ucaccgggagaauagauuugcuu....................	4206	0	S03
.......................................................................caccgggagaauagauuugcu.....................	2	0	S03
.......................................................................caccgggagaauagauuugcuu....................	42	0	S03
........................................................................accgggagaauagauuugcuu....................	1	0	S03
.........................................................................ccgggagaauagauuugcu.....................	1	0	S03
.........................................................................ccgggagaauagauuugcuu....................	3	0	S03
..........................................................................cgggagaauagauuugcu.....................	1	0	S03
..........................................................................cgggagaauagauuugcuu....................	5	0	S03
...........................................................................gggagaauagauuugcuu....................	1	0	S03
```

Provisional ID	:	Scaffold248_6728
Score total	:	79930
Score for star read(s)	:	3.9
Score for read counts	:	79932
Score for mfe	:	-3.7
Score for randfold	:	-2.2
Score for cons. seed	:	
Total read count	:	156795
Mature read count	:	156415
Loop read count	:	0
Star read count	:	380

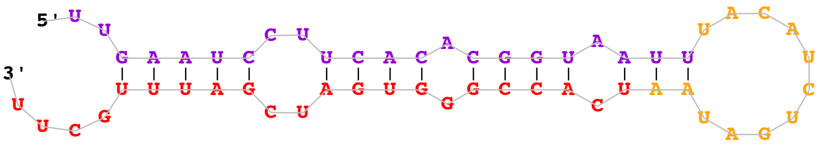

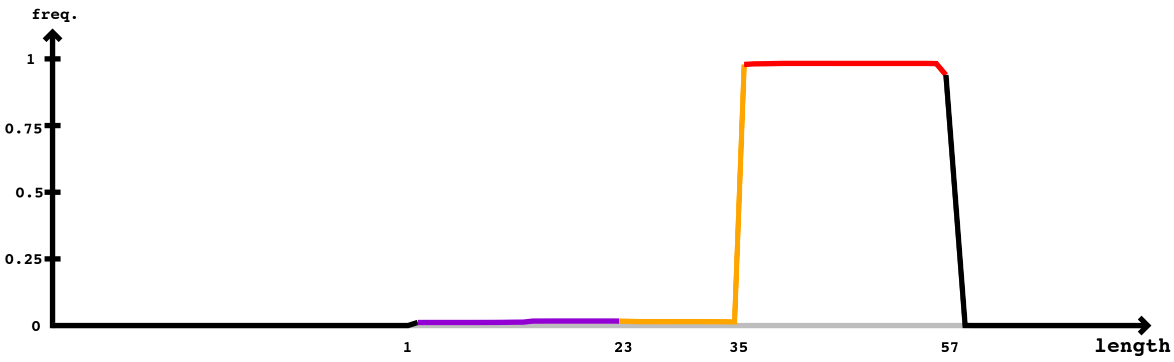

Star Mature

```
5'- cguaauuccuauaaccugaacuuuuugcugaugaacuugaauccuucacacgguaauuuacaucugauaaucaccgggugaucgauuugcuuuuauugcaaacaaaucuaca   -3'    obs
    cguaauuccuauaaccugaacuuuuugcugaugaacuugaauccuucacacgguaauuuacaucugauaaucaccgggugaucgauuugcuuuuauugcaaacaaaucuaca          exp
    .......................(((((.((((((..((((((..((((.((((.(((..........))).)))).))))..))))))..)))))))))))..........    reads     mm     sample
    ....................................uugaauccuucacacgguaauu......................................................    14        0      S05
    ....................................uugaauccuucacacgguaauuuacaucu...............................................    1         0      S05
    ....................................uugaauccuucacacgguaauuuacaucug..............................................    1         0      S05
    ....................................uugaauccuucacacgguaauuuacaucugaua...........................................    4         0      S05
    ....................................uugaauccuucacacgguaauuuacaucugauaa..........................................    101       0      S05
    ....................................uugaauccuucacacgguaauuuacaucugauaau.........................................    4         0      S05
    .....................................ugaauccuucacacgguaauuua....................................................    1         0      S05
    ........................................auccuucacacgguaauuuacaucugauaa..........................................    1         0      S05
    ..............................................cacacgguaauuuacaucugau............................................    1         0      S05
    ..............................................cacacgguaauuuacaucugauaa..........................................    1         0      S05
    ...............................................acacgguaauuuacaucugauaa..........................................    3         0      S05
    ................................................cacgguaauuuacaucugauaa..........................................    6         0      S05
    .................................................acgguaauuuacaucugauaa..........................................    2         0      S05
    .....................................................................aucaccgggugaucgauuugcuu....................    4         0      S05
    ......................................................................ucaccgggugaucgauuugc......................    2         0      S05
    ......................................................................ucaccgggugaucgauuugcu.....................    253       0      S05
    ......................................................................ucaccgggugaucgauuugcuu....................    10516     0      S05
    ......................................................................ucaccgggugaucgauuugcuuu...................    103       0      S05
    ......................................................................ucaccgggugaucgauuugcuuuu..................    1         0      S05
    .......................................................................caccgggugaucgauuugcu.....................    1         0      S05
    .......................................................................caccgggugaucgauuugcuu....................    38        0      S05
    ........................................................................accgggugaucgauuugcuu....................    6         0      S05
    .........................................................................ccgggugaucgauuugcuu....................    9         0      S05
    ..........................................................................cgggugaucgauuugcuu....................    11        0      S05
    ...........................................................................gggugaucgauuugcuuu...................    1         0      S05

    ..uaauuccuauaaccugaacuuuuugcugaugaac............................................................................    1         0      S10
    ....................................uugaauccuucacacggua.........................................................    1         0      S10
    ....................................uugaauccuucacacgguaau.......................................................    6         0      S10
    ....................................uugaauccuucacacgguaauu......................................................    100       0      S10
    ....................................uugaauccuucacacgguaauuuacaucu...............................................    1         0      S10
    ....................................uugaauccuucacacgguaauuuacaucug..............................................    2         0      S10
    ....................................uugaauccuucacacgguaauuuacaucuga.............................................    1         0      S10
```

Star **Mature**

```
cguaauuccuauaaccugaacuuuuugcugaugaacuugaauccuucacacgguaauuuacaucugauaaucaccgggugaucgauuugcuuuuauugcaaacaaaucuaca
....................................uugaauccuucacacgguaauuuacaucugau............................................ 4 0 S10
....................................uugaauccuucacacgguaauuuacaucugaua........................................... 7 0 S10
....................................uugaauccuucacacgguaauuuacaucugauaa.......................................... 89 0 S10
....................................uugaauccuucacacgguaauuuacaucugauaau......................................... 7 0 S10
.....................................ugaauccuucacacgguaauu...................................................... 4 0 S10
.....................................ugaauccuucacacgguaauuua.................................................... 1 0 S10
.........................................uccuucacacgguaauuuacaucugauaa.......................................... 2 0 S10
............................................uucacacgguaauuuacaucugau............................................ 1 0 S10
............................................uucacacgguaauuuacaucugauaa.......................................... 7 0 S10
.............................................ucacacgguaauuuacaucugau............................................ 2 0 S10
.............................................ucacacgguaauuuacaucugauaa.......................................... 14 0 S10
..............................................cacacgguaauuuacaucugau............................................ 14 0 S10
..............................................cacacgguaauuuacaucugaua........................................... 1 0 S10
..............................................cacacgguaauuuacaucugauaa.......................................... 18 0 S10
..............................................cacacgguaauuuacaucugauaau......................................... 1 0 S10
...............................................acacgguaauuuacaucugauaa.......................................... 9 0 S10
...............................................acacgguaauuuacaucugauaau......................................... 3 0 S10
................................................cacgguaauuuacaucug.............................................. 1 0 S10
................................................cacgguaauuuacaucugaua........................................... 1 0 S10
................................................cacgguaauuuacaucugauaa.......................................... 200 0 S10
................................................cacgguaauuuacaucugauaau......................................... 24 0 S10
.................................................acgguaauuuacaucugauaa.......................................... 6 0 S10
.................................................acgguaauuuacaucugauaau......................................... 3 0 S10
..................................................cgguaauuuacaucugauaa.......................................... 1 0 S10
..................................................cgguaauuuacaucugauaauc........................................ 1 0 S10
..........................................................uacaucugauaaucaccgggug................................ 9 0 S10
..........................................................uacaucugauaaucaccgggugaucgauuugcuu.................... 1 0 S10
..............................................................ucugauaaucaccgggugaucgauu......................... 1 0 S10
.....................................................................aucaccgggugaucgauuugcuu.................... 1 0 S10
......................................................................ucaccgggugaucgauuu........................ 2 0 S10
......................................................................ucaccgggugaucgauuugc...................... 29 0 S10
......................................................................ucaccgggugaucgauuugcu..................... 1511 0 S10
......................................................................ucaccgggugaucgauuugcuu.................... 25950 0 S10
......................................................................ucaccgggugaucgauuugcuuu................... 385 0 S10
......................................................................ucaccgggugaucgauuugcuuuu.................. 8 0 S10
.......................................................................caccgggugaucgauuug....................... 1 0 S10
.......................................................................caccgggugaucgauuugcu..................... 1 0 S10
.......................................................................caccgggugaucgauuugcuu.................... 86 0 S10
.......................................................................caccgggugaucgauuugcuuu................... 2 0 S10
........................................................................accgggugaucgauuugcuu.................... 6 0 S10
.........................................................................ccgggugaucgauuugcuu.................... 2 0 S10
..........................................................................cgggugaucgauuugcuu.................... 4 0 S10
...........................................................................gggugaucgauuugcuuu................... 1 0 S10

....................................uugaauccuucacacgguaau....................................................... 1 0 S06
....................................uugaauccuucacacgguaauu...................................................... 17 0 S06
....................................uugaauccuucacacgguaauuuacauc................................................ 1 0 S06
....................................uugaauccuucacacgguaauuuacaucu............................................... 1 0 S06
....................................uugaauccuucacacgguaauuuacaucug.............................................. 2 0 S06
....................................uugaauccuucacacgguaauuuacaucugau............................................ 3 0 S06
....................................uugaauccuucacacgguaauuuacaucugaua........................................... 2 0 S06
....................................uugaauccuucacacgguaauuuacaucugauaa.......................................... 110 0 S06
....................................uugaauccuucacacgguaauuuacaucugauaau......................................... 5 0 S06
.....................................ugaauccuucacacgguaauu...................................................... 1 0 S06
.....................................ugaauccuucacacgguaauuuacaucugauaa.......................................... 2 0 S06
.............................................ucacacgguaauuuacaucugauaa.......................................... 1 0 S06
..............................................cacacgguaauuuacaucugau............................................ 1 0 S06
..............................................cacacgguaauuuacaucugauaa.......................................... 3 0 S06
...............................................acacgguaauuuacaucugau............................................ 1 0 S06
...............................................acacgguaauuuacaucugauaa.......................................... 2 0 S06
................................................cacgguaauuuacaucugauaa.......................................... 13 0 S06
.................................................acgguaauuuacaucugauaa.......................................... 1 0 S06
....................................................guaauuuacaucugauaa.......................................... 1 0 S06
...........................................................acaucugauaaucaccgggugaucgauuugcu..................... 1 0 S06
...............................................................cugauaaucaccgggugaucgauuugcuu.................... 1 0 S06
....................................................................aaucaccgggugaucgauuugcuu.................... 1 0 S06
.....................................................................aucaccgggugaucgauuugcuu.................... 1 0 S06
......................................................................ucaccgggugaucgauuugc...................... 4 0 S06
......................................................................ucaccgggugaucgauuugcu..................... 656 0 S06
......................................................................ucaccgggugaucgauuugcuu.................... 17684 0 S06
```

```
                                    Star                              Mature
cguaauuccuauaaccugaacuuuuugcugaugaacuugaauccuucacacgguaauuuacaucugauaaucaccgggugaucgauuugcuuuuauugcaaacaaaucuaca
......................................................................ucaccgggugaucgauuugcuuu...................  141     0   S06
......................................................................ucaccgggugaucgauuugcuuuu..................  1       0   S06
.......................................................................caccgggugaucgauuugc......................  1       0   S06
.......................................................................caccgggugaucgauuugcu.....................  1       0   S06
.......................................................................caccgggugaucgauuugcuu....................  47      0   S06
........................................................................accgggugaucgauuugcuu....................  15      0   S06
.........................................................................ccgggugaucgauuugcu.....................  1       0   S06
.........................................................................ccgggugaucgauuugcuu....................  16      0   S06
..........................................................................cgggugaucgauuugcuu....................  20      0   S06
..........................................................................cgggugaucgauuugcuuu...................  3       0   S06
...........................................................................gggugaucgauuugcuuu...................  1       0   S06

......................................................................ucaccgggugaucgauuugcuu....................  7       0   S01

....auuccuauaaccugaacuuuuugcugaugaac............................................................................  1       0   S07
....................................uugaauccuucacacggua.........................................................  2       0   S07
....................................uugaauccuucacacgguaa........................................................  3       0   S07
....................................uugaauccuucacacgguaau.......................................................  2       0   S07
....................................uugaauccuucacacgguaauu......................................................  81      0   S07
....................................uugaauccuucacacgguaauuuacauc................................................  1       0   S07
....................................uugaauccuucacacgguaauuuacaucu...............................................  2       0   S07
....................................uugaauccuucacacgguaauuuacaucug..............................................  2       0   S07
....................................uugaauccuucacacgguaauuuacaucuga.............................................  1       0   S07
....................................uugaauccuucacacgguaauuuacaucugau............................................  9       0   S07
....................................uugaauccuucacacgguaauuuacaucugaua...........................................  30      0   S07
....................................uugaauccuucacacgguaauuuacaucugauaa..........................................  499     0   S07
....................................uugaauccuucacacgguaauuuacaucugauaau.........................................  36      0   S07
....................................uugaauccuucacacgguaauuuacaucugauaaucaccg....................................  1       0   S07
.....................................ugaauccuucacacgguaauu......................................................  1       0   S07
.....................................ugaauccuucacacgguaauuuacaucugauaa..........................................  2       0   S07
.....................................ugaauccuucacacgguaauuuacaucugauaau.........................................  1       0   S07
.......................................aauccuucacacgguaauuuacaucugauaa..........................................  1       0   S07
..........................................ccuucacacgguaauuuacaucugauaa..........................................  1       0   S07
..........................................ccuucacacgguaauuuacaucugauaau.........................................  1       0   S07
...........................................cuucacacgguaauuuacaucugauaa..........................................  1       0   S07
............................................uucacacgguaauuuacaucugaua...........................................  2       0   S07
............................................uucacacgguaauuuacaucugauaa..........................................  2       0   S07
............................................uucacacgguaauuuacaucugauaau.........................................  1       0   S07
.............................................ucacacgguaauuuacaucug..............................................  1       0   S07
.............................................ucacacgguaauuuacaucugau............................................  2       0   S07
.............................................ucacacgguaauuuacaucugauaa..........................................  4       0   S07
..............................................cacacgguaauuuacaucugau............................................  4       0   S07
..............................................cacacgguaauuuacaucugaua...........................................  1       0   S07
..............................................cacacgguaauuuacaucugauaa..........................................  9       0   S07
...............................................acacgguaauuuacaucugaua...........................................  1       0   S07
...............................................acacgguaauuuacaucugauaa..........................................  11      0   S07
...............................................acacgguaauuuacaucugauaau.........................................  1       0   S07
................................................cacgguaauuuacaucugauaa..........................................  69      0   S07
................................................cacgguaauuuacaucugauaau.........................................  7       0   S07
.................................................acgguaauuuacaucugauaa..........................................  4       0   S07
..................................................cgguaauuuacaucugauaa..........................................  1       0   S07
..........................................................uacaucugauaaucaccgggug................................  1       0   S07
...........................................................acaucugauaaucaccgggugaucgauuugcuu....................  1       0   S07
...............................................................cugauaaucaccgggugaucgauuugcuu....................  1       0   S07
................................................................ugauaaucaccgggugauc.............................  1       0   S07
.................................................................gauaaucaccgggugaucgauuugcu.....................  1       0   S07
.................................................................gauaaucaccgggugaucgauuugcuu....................  2       0   S07
.................................................................gauaaucaccgggugaucgauuugcuuu...................  1       0   S07
.....................................................................aucaccgggugaucgauuugcu.....................  1       0   S07
.....................................................................aucaccgggugaucgauuugcuu....................  1       0   S07
......................................................................ucaccgggugaucgauuugc......................  14      0   S07
......................................................................ucaccgggugaucgauuugcu.....................  1236    0   S07
......................................................................ucaccgggugaucgauuugcuu....................  24854   0   S07
......................................................................ucaccgggugaucgauuugcuuu...................  349     0   S07
......................................................................ucaccgggugaucgauuugcuuuu..................  5       0   S07
.......................................................................caccgggugaucgauuugcu.....................  1       0   S07
.......................................................................caccgggugaucgauuugcuu....................  77      0   S07
.......................................................................caccgggugaucgauuugcuuuu..................  1       0   S07
........................................................................accgggugaucgauuugcuu....................  7       0   S07
.........................................................................ccgggugaucgauuugcuu....................  6       0   S07
```

```
                                    Star                          Mature
cguaauuccuauaaccugaacuuuuugcugaugaacuugaauccuucacacgguaauuuacaucugauaaucaccgggugaucgauuugcuuuuauugcaaacaaaucuaca
..........................................................................cgggugaucgauuugcuu....................    6      0   S07

....................................uugaauccuucacacgguaau.......................................................    10     0   S09
....................................uugaauccuucacacgguaauu......................................................    85     0   S09
....................................uugaauccuucacacgguaauuuacauc................................................    2      0   S09
....................................uugaauccuucacacgguaauuuacaucug..............................................    3      0   S09
....................................uugaauccuucacacgguaauuuacaucuga.............................................    1      0   S09
....................................uugaauccuucacacgguaauuuacaucugaua...........................................    8      0   S09
....................................uugaauccuucacacgguaauuuacaucugauaa..........................................    126    0   S09
....................................uugaauccuucacacgguaauuuacaucugauaau.........................................    12     0   S09
....................................uugaauccuucacacgguaauuuacaucugauaaucaccgggugaucgauuug.......................    1      0   S09
.......................................aauccuucacacgguaauuuacaucugauaa..........................................    1      0   S09
...........................................cuucacacgguaauuuacaucugaua...........................................    1      0   S09
...........................................cuucacacgguaauuuacaucugauaa..........................................    2      0   S09
............................................uucacacgguaauuuacaucug..............................................    2      0   S09
............................................uucacacgguaauuuacaucugau............................................    1      0   S09
............................................uucacacgguaauuuacaucugauaa..........................................    2      0   S09
.............................................ucacacgguaauuuacaucug..............................................    1      0   S09
.............................................ucacacgguaauuuacaucuga.............................................    1      0   S09
.............................................ucacacgguaauuuacaucugau............................................    1      0   S09
.............................................ucacacgguaauuuacaucugauaa..........................................    11     0   S09
..............................................cacacgguaauuuacaucugau............................................    5      0   S09
..............................................cacacgguaauuuacaucugauaa..........................................    19     0   S09
..............................................cacacgguaauuuacaucugauaau.........................................    1      0   S09
...............................................acacgguaauuuacaucugaua...........................................    3      0   S09
...............................................acacgguaauuuacaucugauaa..........................................    4      0   S09
...............................................acacgguaauuuacaucugauaau.........................................    1      0   S09
................................................cacgguaauuuacaucugaua...........................................    1      0   S09
................................................cacgguaauuuacaucugauaa..........................................    219    0   S09
................................................cacgguaauuuacaucugauaau.........................................    20     0   S09
.................................................acgguaauuuacaucugauaa..........................................    4      0   S09
..........................................................uacaucugauaaucaccgggug................................    1      0   S09
..........................................................uacaucugauaaucaccgggugaucgauuugcuu....................    1      0   S09
................................................................ugauaaucaccgggugaucgauuugcuu....................    1      0   S09
.....................................................................aucaccgggugaucgauuugcu.....................    1      0   S09
......................................................................ucaccgggugaucgauuu........................    1      0   S09
......................................................................ucaccgggugaucgauuugc......................    17     0   S09
......................................................................ucaccgggugaucgauuugcu.....................    1070   0   S09
......................................................................ucaccgggugaucgauuugcuu....................    15299  0   S09
......................................................................ucaccgggugaucgauuugcuuu...................    236    0   S09
......................................................................ucaccgggugaucgauuugcuuuu..................    3      0   S09
.......................................................................caccgggugaucgauuugcu.....................    1      0   S09
.......................................................................caccgggugaucgauuugcuu....................    46     0   S09
.......................................................................caccgggugaucgauuugcuuu...................    1      0   S09
........................................................................accgggugaucgauuugcuu....................    2      0   S09
.........................................................................ccgggugaucgauuugcuu....................    1      0   S09

.....uuccuauaaccugaacuuuuugcugau................................................................................    1      0   S03
....................................uugaauccuucacacgguaauu......................................................    12     0   S03
....................................uugaauccuucacacgguaauuuacaucu...............................................    1      0   S03
....................................uugaauccuucacacgguaauuuacaucug..............................................    1      0   S03
....................................uugaauccuucacacgguaauuuacaucugauaa..........................................    94     0   S03
....................................uugaauccuucacacgguaauuuacaucugauaau.........................................    5      0   S03
.....................................ugaauccuucacacgguaauuuacaucugauaa..........................................    1      0   S03
................................................cacgguaauuuacaucugauaa..........................................    3      0   S03
................................................cacgguaauuuacaucugauaau.........................................    1      0   S03
................................................................ugauaaucaccgggugaucgauuugcuu....................    1      0   S03
....................................................................aaucaccgggugaucgauuugcuu....................    1      0   S03
......................................................................ucaccgggugaucgauuugc......................    4      0   S03
......................................................................ucaccgggugaucgauuugcu.....................    194    0   S03
......................................................................ucaccgggugaucgauuugcuu....................    7123   0   S03
......................................................................ucaccgggugaucgauuugcuuu...................    99     0   S03
......................................................................ucaccgggugaucgauuugcuuuu..................    3      0   S03
.......................................................................caccgggugaucgauuugcuu....................    24     0   S03
........................................................................accgggugaucgauuugcuu....................    6      0   S03
........................................................................accgggugaucgauuugcuuu...................    1      0   S03
.........................................................................ccgggugaucgauuugcuu....................    4      0   S03
..........................................................................cgggugaucgauuugcuu....................    7      0   S03

............aaccugaacuuuuugcugaugaa.............................................................................    1      0   S08
```

Star **Mature**

```
cguaauuccuauaaccugaacuuuuugcugaugaacuugaauccuucacacgguaauuuacaucugauaaucaccgggugaucgauuugcuuuuauugcaaacaaaucuaca
.............accugaacuuuuugcugaugaac............................................................................   1       0   S08
....................................uugaauccuucacacgguaau.......................................................   2       0   S08
....................................uugaauccuucacacgguaauu......................................................   28      0   S08
....................................uugaauccuucacacgguaauuuacaucug..............................................   1       0   S08
....................................uugaauccuucacacgguaauuuacaucugaua...........................................   4       0   S08
....................................uugaauccuucacacgguaauuuacaucugauaa..........................................   102     0   S08
....................................uugaauccuucacacgguaauuuacaucugauaau.........................................   2       0   S08
.....................................ugaauccuucacacgguaauuua....................................................   2       0   S08
.....................................ugaauccuucacacgguaauuuacaucugauaa..........................................   1       0   S08
............................................uucacacgguaauuuacaucugaua...........................................   1       0   S08
............................................uucacacgguaauuuacaucugauaa..........................................   3       0   S08
.............................................ucacacgguaauuuacaucugauaa..........................................   5       0   S08
..............................................cacacgguaauuuacaucugau............................................   7       0   S08
..............................................cacacgguaauuuacaucugaua...........................................   1       0   S08
..............................................cacacgguaauuuacaucugauaa..........................................   1       0   S08
...............................................acacgguaauuuacaucugauaa..........................................   10      0   S08
................................................cacgguaauuuacaucugauaa..........................................   37      0   S08
................................................cacgguaauuuacaucugauaau.........................................   2       0   S08
.................................................acgguaauuuacaucugauaa..........................................   4       0   S08
.................................................acgguaauuuacaucugauaau.........................................   1       0   S08
.................................................acgguaauuuacaucugauaaucaccgggugaucgauuugcuu....................   1       0   S08
..................................................cgguaauuuacaucugauaa..........................................   1       0   S08
..................................................cgguaauuuacaucugauaaucaccgggugaucgauuugcu.....................   1       0   S08
........................................................uuuacaucugauaaucaccggg..................................   2       0   S08
..........................................................uacaucugauaaucaccgggugaucgauuugcu.....................   1       0   S08
..........................................................uacaucugauaaucaccgggugaucgauuugcuu....................   1       0   S08
.............................................................aucugauaaucaccgggugaucgauuugc......................   2       0   S08
................................................................ugauaaucaccgggugaucgauuugcu.....................   3       0   S08
.................................................................gauaaucaccgggugaucgauuugcuu....................   2       0   S08
....................................................................aaucaccgggugaucgauuugcuu....................   2       0   S08
.....................................................................aucaccgggugaucgauuugcu.....................   2       0   S08
.....................................................................aucaccgggugaucgauuugcuu....................   1       0   S08
......................................................................ucaccgggugaucgauuu........................   1       0   S08
......................................................................ucaccgggugaucgauuugc......................   13      0   S08
......................................................................ucaccgggugaucgauuugcu.....................   1654    0   S08
......................................................................ucaccgggugaucgauuugcuu....................   37109   0   S08
......................................................................ucaccgggugaucgauuugcuuu...................   324     0   S08
......................................................................ucaccgggugaucgauuugcuuuu..................   1       0   S08
.......................................................................caccgggugaucgauuugcu.....................   3       0   S08
.......................................................................caccgggugaucgauuugcuu....................   169     0   S08
.......................................................................caccgggugaucgauuugcuuu...................   4       0   S08
........................................................................accgggugaucgauuugcu.....................   1       0   S08
........................................................................accgggugaucgauuugcuu....................   19      0   S08
.........................................................................ccgggugaucgauuugcu.....................   1       0   S08
.........................................................................ccgggugaucgauuugcuu....................   45      0   S08
..........................................................................cgggugaucgauuugcuu....................   46      0   S08
...........................................................................gggugaucgauuugcuuu...................   1       0   S08

......................................................................ucaccgggugaucgauuugcu.....................   1       0   S02
......................................................................ucaccgggugaucgauuugcuu....................   24      0   S02
.......................................................................caccgggugaucgauuugcuu....................   1       0   S02

....auuccuauaaccugaacuuuuugcugaugaac............................................................................   1       0   S04
....................................uugaauccuucacacgguaauu......................................................   5       0   S04
....................................uugaauccuucacacgguaauuu.....................................................   1       0   S04
....................................uugaauccuucacacgguaauuuacaucuga.............................................   2       0   S04
....................................uugaauccuucacacgguaauuuacaucugaua...........................................   11      0   S04
....................................uugaauccuucacacgguaauuuacaucugauaa..........................................   150     0   S04
....................................uugaauccuucacacgguaauuuacaucugauaau.........................................   5       0   S04
.............................................ucacacgguaauuuacaucugauaa..........................................   1       0   S04
..............................................cacacgguaauuuacaucugau............................................   1       0   S04
...............................................acacgguaauuuacaucugauaa..........................................   1       0   S04
................................................cacgguaauuuacaucugauaa..........................................   5       0   S04
.................................................acgguaauuuacaucugauaau.........................................   1       0   S04
....................................................guaauuuacaucugauaa..........................................   1       0   S04
..........................................................uacaucugauaaucaccgggugaucgauuugcu.....................   1       0   S04
.....................................................................aucaccgggugaucgauuugcuu....................   1       0   S04
......................................................................ucaccgggugaucgauuugc......................   4       0   S04
......................................................................ucaccgggugaucgauuugcu.....................   219     0   S04
......................................................................ucaccgggugaucgauuugcuu....................   8409    0   S04
```

```
                                    Star                              Mature
cguaauuccuauaaccugaacuuuuugcugaugaacuugaauccuucacacgguaauuuacaucugauaaucaccgggugaucgauuugcuuuuauugcaaacaaaucuaca
......................................................................ucaccgggugaucgauuugcuuu...................    78    0    S04
......................................................................ucaccgggugaucgauuugcuuuu..................    1     0    S04
.......................................................................caccgggugaucgauuugcuu....................    23    0    S04
........................................................................accgggugaucgauuugcuu....................    6     0    S04
.........................................................................ccgggugaucgauuugcuu....................    2     0    S04
..........................................................................cgggugaucgauuugcuu....................    13    0    S04
...........................................................................gggugaucgauuugcuuu...................    1     0    S04
```

Provisional ID	:	Scaffold248_6744
Score total	:	190165.1
Score for star read(s)	:	3.9
Score for read counts	:	190158.8
Score for mfe	:	0.8
Score for randfold	:	1.6
Score for cons. seed	:	
Total read count	:	373000
Mature read count	:	371302
Loop read count	:	0
Star read count	:	1698

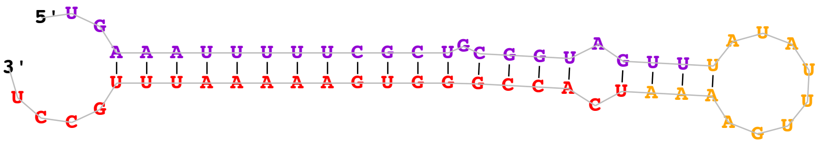

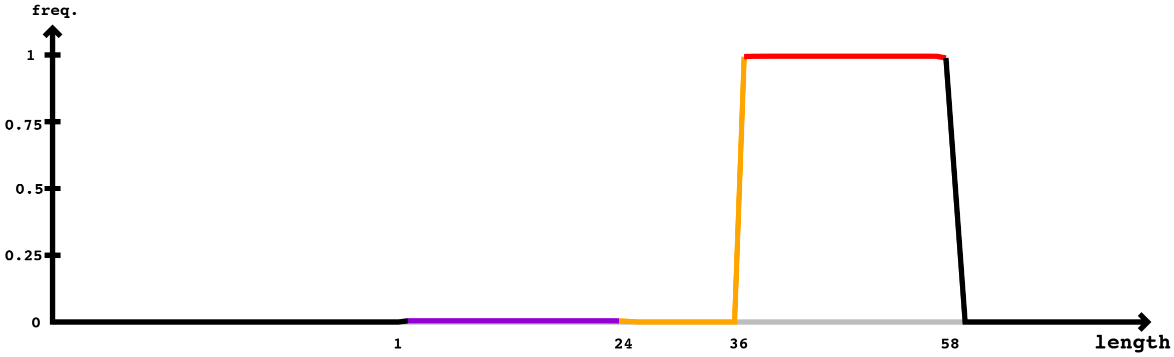


```
                                   Star                                  Mature
5'- guaaauaaagugcucguuuugauuucuucacggaaugaaauuuuucgcugcgguaguuuauauuugaaaaucaccgggugaaaaaauuugccucugugcagaagcaaaucuac  -3'  obs
    guaaauaaagugcucguuuugauuucuucacggaaugaaauuuuucgcugcgguaguuuauauuugaaaaucaccgggugaaaaaauuugccucugugcagaagcaaaucuac       exp
    ...............(.((((.(((((.((((((...((((((((((((.((((.((((.........)))).))))))))))))))))...)))))).))))))))).)...   reads  mm  sample
    ...................................ugaaauuuuucgcugcgg............................................................   3      0   S05
    ...................................ugaaauuuuucgcugcggua..........................................................   2      0   S05
    ...................................ugaaauuuuucgcugcgguag.........................................................   4      0   S05
    ...................................ugaaauuuuucgcugcgguagu........................................................   20     0   S05
    ...................................ugaaauuuuucgcugcgguaguu.......................................................   272    0   S05
    ...................................ugaaauuuuucgcugcgguaguuu......................................................   2      0   S05
    ...................................ugaaauuuuucgcugcgguaguuuauauuugaaa............................................   1      0   S05
    ...................................ugaaauuuuucgcugcgguaguuuauauuugaaaa...........................................   1      0   S05
    ....................................gaaauuuuucgcugcgguaguu.......................................................   3      0   S05
    .....................................aaauuuuucgcugcgguaguu.......................................................   1      0   S05
    ........................................uuuuucgcugcgguaguuuauauuugaaaaucacc......................................   1      0   S05
    ..........................................................uauauuugaaaaucaccgggugaaaaaauuugccu....................   1      0   S05
    ....................................................................aaucaccgggugaaaaaauuugccu....................   1      0   S05
    .....................................................................aucaccgggugaaaaaauuugcc.....................   1      0   S05
    .....................................................................aucaccgggugaaaaaauuugccu....................   2      0   S05
    ......................................................................ucaccgggugaaaaaauuugc......................   10     0   S05
    ......................................................................ucaccgggugaaaaaauuugcc.....................   283    0   S05
    ......................................................................ucaccgggugaaaaaauuugccu....................   47689  0   S05
    ......................................................................ucaccgggugaaaaaauuugccuc...................   6      0   S05
    .......................................................................caccgggugaaaaaauuugccu....................   85     0   S05
    ........................................................................accgggugaaaaaauuugccu....................   13     0   S05
    .........................................................................ccgggugaaaaaauuugccu....................   9      0   S05
    ..........................................................................cgggugaaaaaauuugccu....................   1      0   S05

    ...................................ugaaauuuuucgcugcgg............................................................   1      0   S10
    ...................................ugaaauuuuucgcugcgguagu........................................................   3      0   S10
    ...................................ugaaauuuuucgcugcgguaguu.......................................................   16     0   S10
    ...................................ugaaauuuuucgcugcgguaguuuauauu................................................   1      0   S10
    ....................................................................aaucaccgggugaaaaaauuugccu....................   1      0   S10
    ......................................................................ucaccgggugaaaaaauuug.......................   1      0   S10
    ......................................................................ucaccgggugaaaaaauuugc......................   9      0   S10
    ......................................................................ucaccgggugaaaaaauuugcc.....................   337    0   S10
    ......................................................................ucaccgggugaaaaaauuugccu....................   39226  0   S10
```

Star **Mature**

```
guaaauaaagugcucguuuugauuucuucacggaaugaaauuuuucgcugcgguaguuuauauuugaaaaucaccgggugaaaaauuugccucugugcagaagcaaaucuac
......................................................................ucaccgggugaaaaauuugccuc...................   2      0   S10
.......................................................................caccgggugaaaaauuugccu....................   38     0   S10
........................................................................accgggugaaaaauuugccu....................   5      0   S10
.........................................................................ccgggugaaaaauuugccu....................   3      0   S10
..........................................................................cgggugaaaaauuugccu....................   1      0   S10

...................................ugaaauuuuucgcugcgg...........................................................   2      0   S06
...................................ugaaauuuuucgcugcgguag........................................................   3      0   S06
...................................ugaaauuuuucgcugcgguagu.......................................................   15     0   S06
...................................ugaaauuuuucgcugcgguaguu......................................................   148    0   S06
...................................ugaaauuuuucgcugcgguaguuu.....................................................   3      0   S06
...................................ugaaauuuuucgcugcgguaguuuauauuugaaaa..........................................   1      0   S06
....................................gaaauuuuucgcugcgguaguu......................................................   2      0   S06
....................................................guaguuuauauuugaaaaucaccgggug................................   1      0   S06
...............................................................uugaaaaucaccgggugaaaaau..........................   1      0   S06
.................................................................gaaaaucaccgggugaaaaauuugccu....................   1      0   S06
......................................................................ucaccgggugaaaaauuugc......................   20     0   S06
......................................................................ucaccgggugaaaaauuugcc.....................   353    0   S06
......................................................................ucaccgggugaaaaauuugccu....................   49321  0   S06
......................................................................ucaccgggugaaaaauuugccuc...................   5      0   S06
.......................................................................caccgggugaaaaauuugccu....................   70     0   S06
........................................................................accgggugaaaaauuugccu....................   11     0   S06
.........................................................................ccgggugaaaaauuugccu....................   7      0   S06
..........................................................................cgggugaaaaauuugccu....................   2      0   S06

...................................ugaaauuuuucgcugcgguaguu......................................................   5      0   S01
......................................................................ucaccgggugaaaaauuugc......................   3      0   S01
......................................................................ucaccgggugaaaaauuugcc.....................   9      0   S01
......................................................................ucaccgggugaaaaauuugccu....................   5948   0   S01
.......................................................................caccgggugaaaaauuugccu....................   3      0   S01
........................................................................accgggugaaaaauuugccu....................   2      0   S01
.........................................................................ccgggugaaaaauuugccu....................   2      0   S01

...................................ugaaauuuuucgcugcgg...........................................................   1      0   S07
...................................ugaaauuuuucgcugcgguag........................................................   2      0   S07
...................................ugaaauuuuucgcugcgguagu.......................................................   17     0   S07
...................................ugaaauuuuucgcugcgguaguu......................................................   153    0   S07
...................................ugaaauuuuucgcugcgguaguuu.....................................................   1      0   S07
...................................ugaaauuuuucgcugcgguaguuuauauu................................................   1      0   S07
...................................ugaaauuuuucgcugcgguaguuuauauuugaaaa..........................................   1      0   S07
....................................gaaauuuuucgcugcgguaguu......................................................   2      0   S07
.........................................................uuauauuugaaaaucaccgggu.................................   1      0   S07
.....................................................................aucaccgggugaaaaauuugccu....................   1      0   S07
......................................................................ucaccgggugaaaaauuug.......................   1      0   S07
......................................................................ucaccgggugaaaaauuugc......................   13     0   S07
......................................................................ucaccgggugaaaaauuugcc.....................   346    0   S07
......................................................................ucaccgggugaaaaauuugccu....................   41778  0   S07
......................................................................ucaccgggugaaaaauuugccuc...................   4      0   S07
.......................................................................caccgggugaaaaauuugccu....................   55     0   S07
........................................................................accgggugaaaaauuugccu....................   9      0   S07
.........................................................................ccgggugaaaaauuugccu....................   5      0   S07
..........................................................................cgggugaaaaauuugccu....................   1      0   S07

...................................ugaaauuuuucgcugcgg...........................................................   1      0   S09
...................................ugaaauuuuucgcugcgguagu.......................................................   1      0   S09
...................................ugaaauuuuucgcugcgguaguu......................................................   18     0   S09
......................................................................ucaccgggugaaaaauuugc......................   6      0   S09
......................................................................ucaccgggugaaaaauuugcc.....................   314    0   S09
......................................................................ucaccgggugaaaaauuugccu....................   34916  0   S09
......................................................................ucaccgggugaaaaauuugccuc...................   2      0   S09
.......................................................................caccgggugaaaaauuugccu....................   24     0   S09
........................................................................accgggugaaaaauuugccu....................   5      0   S09
.........................................................................ccgggugaaaaauuugccu....................   3      0   S09
..........................................................................cgggugaaaaauuugccu....................   1      0   S09

...................................ugaaauuuuucgcugcgg...........................................................   2      0   S03
...................................ugaaauuuuucgcugcgguag........................................................   2      0   S03
...................................ugaaauuuuucgcugcgguagu.......................................................   29     0   S03
...................................ugaaauuuuucgcugcgguaguu......................................................   376    0   S03
...................................ugaaauuuuucgcugcgguaguuu.....................................................   4      0   S03
```

```
                                  Star                              Mature
guaaauaaagugcucguuuugauuucuucacggaaugaaauuuuucgcugcgguaguuuauauuugaaaaucaccgggugaaaaauuugccucugugcagaagcaaaucuac
...................................ugaaauuuuucgcugcgguaguuuauauuugaaa...........................................  1      0  S03
...................................ugaaauuuuucgcugcgguaguuuauauuugaaaa..........................................  2      0  S03
....................................gaaauuuuucgcugcgguaguu......................................................  1      0  S03
........................................................uuauauuugaaaaucaccgggugaaaaauuugccu....................  1      0  S03
..............................................................uuugaaaaucaccgggugaaaaauuugccu....................  2      0  S03
......................................................................ucaccgggugaaaaauuugc......................  7      0  S03
......................................................................ucaccgggugaaaaauuugcc.....................  173    0  S03
......................................................................ucaccgggugaaaaauuugccu....................  32147  0  S03
......................................................................ucaccgggugaaaaauuugccuc...................  5      0  S03
.......................................................................caccgggugaaaaauuugcc.....................  1      0  S03
.......................................................................caccgggugaaaaauuugccu....................  51     0  S03
........................................................................accgggugaaaaauuugccu....................  7      0  S03
.........................................................................ccgggugaaaaauuugccu....................  2      0  S03

...............guuuugauuucuucacggaa.............................................................................  1      0  S08
.................................aaugaaauuuuucgcugcgguagu.......................................................  2      0  S08
...................................ugaaauuuuucgcugcgguagu.......................................................  4      0  S08
...................................ugaaauuuuucgcugcgguaguu......................................................  28     0  S08
....................................gaaauuuuucgcugcgguagu.......................................................  1      0  S08
....................................................................aucaccgggugaaaaauuugccu....................  1      0  S08
......................................................................ucaccgggugaaaaauuu........................  1      0  S08
......................................................................ucaccgggugaaaaauuugc......................  20     0  S08
......................................................................ucaccgggugaaaaauuugcc.....................  408    0  S08
......................................................................ucaccgggugaaaaauuugccu....................  68241  0  S08
......................................................................ucaccgggugaaaaauuugccuc...................  6      0  S08
.......................................................................caccgggugaaaaauuugccu....................  100    0  S08
........................................................................accgggugaaaaauuugccu....................  12     0  S08
.........................................................................ccgggugaaaaauuugccu....................  17     0  S08
..........................................................................cgggugaaaaauuugccu....................  3      0  S08

................uuuugauuucuucacggaa.............................................................................  1      0  S04
...................................ugaaauuuuucgcugcgg...........................................................  3      0  S04
...................................ugaaauuuuucgcugcgguag........................................................  3      0  S04
...................................ugaaauuuuucgcugcgguagu.......................................................  29     0  S04
...................................ugaaauuuuucgcugcgguaguu......................................................  463    0  S04
...................................ugaaauuuuucgcugcgguaguuu.....................................................  5      0  S04
...................................ugaaauuuuucgcugcgguaguuuauauu................................................  1      0  S04
...................................ugaaauuuuucgcugcgguaguuuauauuu...............................................  1      0  S04
...................................ugaaauuuuucgcugcgguaguuuauauuugaaa...........................................  1      0  S04
...................................ugaaauuuuucgcugcgguaguuuauauuugaaaa..........................................  1      0  S04
....................................gaaauuuuucgcugcgguaguu......................................................  4      0  S04
....................................................guaguuuauauuugaaaaucaccgggug................................  1      0  S04
......................................................................ucaccgggugaaaaauuugc......................  6      0  S04
......................................................................ucaccgggugaaaaauuugcc.....................  160    0  S04
......................................................................ucaccgggugaaaaauuugccu....................  42724  0  S04
......................................................................ucaccgggugaaaaauuugccuc...................  4      0  S04
.......................................................................caccgggugaaaaauuugc......................  1      0  S04
.......................................................................caccgggugaaaaauuugccu....................  65     0  S04
........................................................................accgggugaaaaauuugccu....................  10     0  S04
.........................................................................ccgggugaaaaauuugccu....................  5      0  S04
..........................................................................cgggugaaaaauuugccu....................  1      0  S04

...................................ugaaauuuuucgcugcgguagu.......................................................  5      0  S02
...................................ugaaauuuuucgcugcgguaguu......................................................  36     0  S02
....................................................................aucaccgggugaaaaauuugccu....................  1      0  S02
......................................................................ucaccgggugaaaaauuugc......................  1      0  S02
......................................................................ucaccgggugaaaaauuugcc.....................  14     0  S02
......................................................................ucaccgggugaaaaauuugccu....................  6131   0  S02
......................................................................ucaccgggugaaaaauuugccucu..................  1      0  S02
.......................................................................caccgggugaaaaauuugccu....................  12     0  S02
.........................................................................ccgggugaaaaauuugccu....................  1      0  S02
```

Provisional ID : Scaffold248_6726
Score total : 125944.2
Score for star read(s) : 3.9
Score for read counts : 125937.1
Score for mfe : 1.6
Score for randfold : 1.6
Score for cons. seed :
Total read count : 247032
Mature read count : 243778
Loop read count : 0
Star read count : 3254

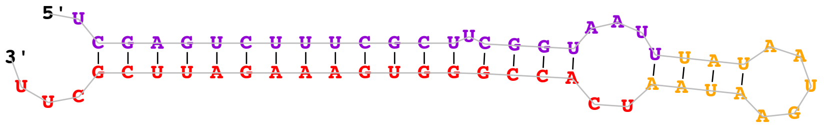

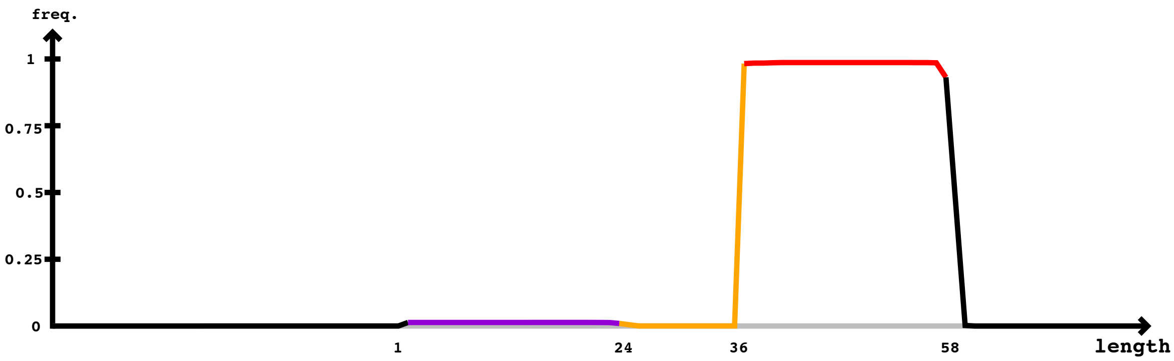

Star Mature

```
5'- uacagaacuuuuauguugggcauuuuuugaugaauucgagucuuucgcuucgguaauuuauaaugaauaaucaccgggugaaagauucgcuuuuauugaagaauaugucugc   -3'   obs
    uacagaacuuuuauguugggcauuuuuugaugaauucgagucuuucgcuucgguaauuuauaaugaauaaucaccgggugaaagauucgcuuuuauugaagaauaugucugc         exp
    .................((((((((((..(((((..(((((((((((((.((((...((((.....))))..)))))))))))))))))..)))))..))))..))))))..   reads   mm   sample
    ......acuuuuauguugggcauuuuuugaugaau.............................................................................   1   0   S08
    ........uuuuauguugggcauuuuuugauga...............................................................................   1   0   S08
    ...............uugggcauuuuuugaugaau.............................................................................   3   0   S08
    ...................................ucgagucuuucgcuucggu..........................................................   2   0   S08
    ...................................ucgagucuuucgcuucggua.........................................................   16   0   S08
    ...................................ucgagucuuucgcuucgguaa........................................................   19   0   S08
    ...................................ucgagucuuucgcuucgguaau.......................................................   265   0   S08
    ...................................ucgagucuuucgcuucgguaauu......................................................   641   0   S08
    ...................................ucgagucuuucgcuucgguaauuuauaaug...............................................   1   0   S08
    ...................................ucgagucuuucgcuucgguaauuuauaaugaau............................................   6   0   S08
    ...................................ucgagucuuucgcuucgguaauuuauaaugaauaau.........................................   1   0   S08
    ................................................uucgguaauuuauaaugaauaa..........................................   1   0   S08
    ...............................................................ugaauaaucaccgggugaaagauucgcuu....................   1   0   S08
    ................................................................gaauaaucaccgggugaaagauucgcuu....................   1   0   S08
    ....................................................................aaucaccgggugaaagauucgcuu....................   4   0   S08
    ....................................................................aaucaccgggugaaagauucgcuuu...................   1   0   S08
    .....................................................................aucaccgggugaaagauucgcu.....................   1   0   S08
    .....................................................................aucaccgggugaaagauucgcuu....................   2   0   S08
    ......................................................................ucaccgggugaaagauuc........................   15   0   S08
    ......................................................................ucaccgggugaaagauucg.......................   1   0   S08
    ......................................................................ucaccgggugaaagauucgc......................   39   0   S08
    ......................................................................ucaccgggugaaagauucgcu.....................   2407   0   S08
    ......................................................................ucaccgggugaaagauucgcuu....................   40132   0   S08
    ......................................................................ucaccgggugaaagauucgcuuu...................   2800   0   S08
    ......................................................................ucaccgggugaaagauucgcuuuu..................   71   0   S08
    ......................................................................ucaccgggugaaagauucgcuuuua.................   1   0   S08
    .......................................................................caccgggugaaagauucgcu.....................   7   0   S08
    .......................................................................caccgggugaaagauucgcuu....................   86   0   S08
    .......................................................................caccgggugaaagauucgcuuu...................   7   0   S08
    ........................................................................accgggugaaagauucgcuu....................   6   0   S08
    .........................................................................ccgggugaaagauucgcu.....................   5   0   S08
    .........................................................................ccgggugaaagauucgcuu....................   143   0   S08
    .........................................................................ccgggugaaagauucgcuuu...................   12   0   S08
```

Star Mature

```
uacagaacuuuuauguugggcauuuuuugaugaauucgagucuuucgcuucgguaauuuauaaugaauaaucaccgggugaaagauucgcuuuuauugaagaauaugucugc
..........................................................................cgggugaaagauucgcuu....................	91	0	S08
..........................................................................cgggugaaagauucgcuuu...................	6	0	S08
...........................................................................gggugaaagauucgcuuu...................	2	0	S08

.........uuuauguugggcauuuuuugaugaa..............................................................................	1	0	S03
..............guugggcauuuuuugaugaau.............................................................................	3	0	S03
...............uugggcauuuuuugaugaau.............................................................................	1	0	S03
...................................ucgagucuuucgcuucggua.........................................................	3	0	S03
...................................ucgagucuuucgcuucgguaa........................................................	6	0	S03
...................................ucgagucuuucgcuucgguaau.......................................................	52	0	S03
...................................ucgagucuuucgcuucgguaauu......................................................	164	0	S03
......................................agucuuucgcuucgguaauu......................................................	1	0	S03
................................................................gaauaaucaccgggugaaagauucgcuu....................	1	0	S03
.................................................................aauaaucaccgggugaaagauucgcuu....................	2	0	S03
...................................................................uaaucaccgggugaaagauucgcuuu...................	1	0	S03
....................................................................aaucaccgggugaaagauucgcuu....................	1	0	S03
......................................................................ucaccgggugaaagauuc........................	1	0	S03
......................................................................ucaccgggugaaagauucgc......................	8	0	S03
......................................................................ucaccgggugaaagauucgcu.....................	274	0	S03
......................................................................ucaccgggugaaagauucgcuu....................	6514	0	S03
......................................................................ucaccgggugaaagauucgcuuu...................	512	0	S03
......................................................................ucaccgggugaaagauucgcuuuu..................	28	0	S03
.......................................................................caccgggugaaagauucgcu.....................	1	0	S03
.......................................................................caccgggugaaagauucgcuu....................	13	0	S03
.......................................................................caccgggugaaagauucgcuuu...................	4	0	S03
........................................................................accgggugaaagauucgcuu....................	1	0	S03
.........................................................................ccgggugaaagauucgcuu....................	6	0	S03
.........................................................................ccgggugaaagauucgcuuu...................	3	0	S03
..........................................................................cgggugaaagauucgcuu....................	1	0	S03

.........uuuauguugggcauuuuuugaugaau.............................................................................	1	0	S09
............auguugggcauuuuuugaugaau.............................................................................	2	0	S09
...............uugggcauuuuuugaugaau.............................................................................	1	0	S09
...................................ucgagucuuucgcuucggua.........................................................	2	0	S09
...................................ucgagucuuucgcuucgguaa........................................................	3	0	S09
...................................ucgagucuuucgcuucgguaau.......................................................	27	0	S09
...................................ucgagucuuucgcuucgguaauu......................................................	212	0	S09
...................................ucgagucuuucgcuucgguaauuu.....................................................	1	0	S09
...................................ucgagucuuucgcuucgguaauuuauaaugaauaa..........................................	1	0	S09
.....................................gagucuuucgcuucgguaau.......................................................	1	0	S09
............................................ucgcuucgguaauuuauaauga..............................................	1	0	S09
.............................................cgcuucgguaauuuauaaugaa.............................................	1	0	S09
................................................uucgguaauuuauaaugaaua...........................................	1	0	S09
................................................uucgguaauuuauaaugaauaa..........................................	3	0	S09
.................................................ucgguaauuuauaaugaauaau.........................................	2	0	S09
..........................................................uauaaugaauaaucaccgggugaaagauucgcuu....................	1	0	S09
....................................................................aaucaccgggugaaagauucgcuu....................	1	0	S09
.....................................................................aucaccgggugaaagauucgcuu....................	1	0	S09
......................................................................ucaccgggugaaagauuc........................	10	0	S09
......................................................................ucaccgggugaaagauucg.......................	1	0	S09
......................................................................ucaccgggugaaagauucgc......................	24	0	S09
......................................................................ucaccgggugaaagauucgcu.....................	2797	0	S09
......................................................................ucaccgggugaaagauucgcuu....................	43943	0	S09
......................................................................ucaccgggugaaagauucgcuuu...................	2720	0	S09
......................................................................ucaccgggugaaagauucgcuuuu..................	84	0	S09
.......................................................................caccgggugaaagauucgcu.....................	6	0	S09
.......................................................................caccgggugaaagauucgcuu....................	60	0	S09
.......................................................................caccgggugaaagauucgcuuu...................	11	0	S09
........................................................................accgggugaaagauucgcuu....................	11	0	S09
.........................................................................ccgggugaaagauucgcuu....................	16	0	S09
..........................................................................cgggugaaagauucgcuu....................	10	0	S09

................................aauucgagucuuucgcuucggua.........................................................	1	0	S04
...................................ucgagucuuucgcuucggu..........................................................	2	0	S04
...................................ucgagucuuucgcuucggua.........................................................	3	0	S04
...................................ucgagucuuucgcuucgguaa........................................................	4	0	S04
...................................ucgagucuuucgcuucgguaau.......................................................	41	0	S04
...................................ucgagucuuucgcuucgguaauu......................................................	165	0	S04
...................................ucgagucuuucgcuucgguaauuuauaaugaauaa..........................................	2	0	S04
.............................................cgcuucgguaauuuauaaugaa.............................................	1	0	S04
```

Star Mature

```
uacagaacuuuuauguugggcauuuuuugaugaauucgagucuuucgcuucgguaauuuauaaugaauaaucaccgggugaaagauucgcuuuuauugaagaauaugucugc
.................................................................aauaaucaccgggugaaagauucgcuu....................    1      0   S04
...................................................................uaaucaccgggugaaagauucgcuu....................    1      0   S04
......................................................................ucaccgggugaaagauuc........................    1      0   S04
......................................................................ucaccgggugaaagauucgc......................    5      0   S04
......................................................................ucaccgggugaaagauucgcu.....................    333    0   S04
......................................................................ucaccgggugaaagauucgcuu....................    7084   0   S04
......................................................................ucaccgggugaaagauucgcuuu...................    709    0   S04
......................................................................ucaccgggugaaagauucgcuuuu..................    26     0   S04
.......................................................................caccgggugaaagauucgcu.....................    1      0   S04
.......................................................................caccgggugaaagauucgcuu....................    25     0   S04
.........................................................................ccgggugaaagauucgcuu....................    6      0   S04
.........................................................................ccgggugaaagauucgcuuu...................    2      0   S04
..........................................................................cgggugaaagauucgcuu....................    1      0   S04

...................................ucgagucuuucgcuucgguaau.......................................................    1      0   S02
......................................................................ucaccgggugaaagauucgcu.....................    1      0   S02
......................................................................ucaccgggugaaagauucgcuu....................    41     0   S02
......................................................................ucaccgggugaaagauucgcuuu...................    2      0   S02

.acagaacuuuuauguugggcauuuuuugaugaau.............................................................................    1      0   S10
...........uauguugggcauuuuuugaugaau.............................................................................    1      0   S10
.............uguugggcauuuuuugaugaau.............................................................................    1      0   S10
...............uugggcauuuuuugaugaau.............................................................................    1      0   S10
...................................ucgagucuuucgcuucgg...........................................................    1      0   S10
...................................ucgagucuuucgcuucggua.........................................................    2      0   S10
...................................ucgagucuuucgcuucgguaa........................................................    4      0   S10
...................................ucgagucuuucgcuucgguaau.......................................................    64     0   S10
...................................ucgagucuuucgcuucgguaauu......................................................    306    0   S10
...................................ucgagucuuucgcuucgguaauuu.....................................................    2      0   S10
...................................ucgagucuuucgcuucgguaauuuauaau................................................    1      0   S10
...................................ucgagucuuucgcuucgguaauuuauaaug...............................................    1      0   S10
...................................ucgagucuuucgcuucgguaauuuauaaugaa.............................................    1      0   S10
...................................ucgagucuuucgcuucgguaauuuauaaugaau............................................    1      0   S10
...................................ucgagucuuucgcuucgguaauuuauaaugaauaau.........................................    1      0   S10
.....................................gagucuuucgcuucgguaau.......................................................    1      0   S10
............................................ucgcuucgguaauuuauaaugaau............................................    1      0   S10
.............................................cgcuucgguaauuuauaaugaa.............................................    1      0   S10
.............................................cgcuucgguaauuuauaaugaau............................................    1      0   S10
................................................uucgguaauuuauaaugaaua...........................................    3      0   S10
................................................uucgguaauuuauaaugaauaa..........................................    3      0   S10
................................................uucgguaauuuauaaugaauaau.........................................    3      0   S10
...................................................................aaucaccgggugaaagauucgcuu....................    2      0   S10
....................................................................aucaccgggugaaagauucgcu.....................    1      0   S10
....................................................................aucaccgggugaaagauucgcuu....................    1      0   S10
......................................................................ucaccgggugaaagauuc........................    11     0   S10
......................................................................ucaccgggugaaagauucg.......................    1      0   S10
......................................................................ucaccgggugaaagauucgc......................    47     0   S10
......................................................................ucaccgggugaaagauucgcu.....................    3777   0   S10
......................................................................ucaccgggugaaagauucgcuu....................    65221  0   S10
......................................................................ucaccgggugaaagauucgcuuu...................    4535   0   S10
......................................................................ucaccgggugaaagauucgcuuuu..................    149    0   S10
.......................................................................caccgggugaaagauucgcu.....................    6      0   S10
.......................................................................caccgggugaaagauucgcuu....................    81     0   S10
.......................................................................caccgggugaaagauucgcuuu...................    16     0   S10
........................................................................accgggugaaagauucgcu.....................    2      0   S10
........................................................................accgggugaaagauucgcuu....................    9      0   S10
.........................................................................ccgggugaaagauucgcuu....................    35     0   S10
.........................................................................ccgggugaaagauucgcuuu...................    4      0   S10
..........................................................................cgggugaaagauucgcuu....................    17     0   S10
..........................................................................cgggugaaagauucgcuuu...................    2      0   S10

.........uuuauguugggcauuuuuugaugaau.............................................................................    1      0   S05
................................aauucgagucuuucgcuucggua.........................................................    1      0   S05
...................................ucgagucuuucgcuucggu..........................................................    1      0   S05
...................................ucgagucuuucgcuucggua.........................................................    1      0   S05
...................................ucgagucuuucgcuucgguaa........................................................    2      0   S05
...................................ucgagucuuucgcuucgguaau.......................................................    58     0   S05
...................................ucgagucuuucgcuucgguaauu......................................................    204    0   S05
...................................ucgagucuuucgcuucgguaauuuauaaugaauaau.........................................    1      0   S05
..................................................................auaaucaccgggugaaagauucgcuu....................    1      0   S05
```

Star Mature

```
uacagaacuuuuauguugggcauuuuuugaugaauucgagucuuucgcuucgguaauuuauaaugaauaaucaccgggugaaagauucgcuuuuauugaagaauaugucugc
......................................................................ucaccgggugaaagauucgc......................    7       0    S05
......................................................................ucaccgggugaaagauucgcu.....................    432     0    S05
......................................................................ucaccgggugaaagauucgcuu....................    8850    0    S05
......................................................................ucaccgggugaaagauucgcuuu...................    879     0    S05
......................................................................ucaccgggugaaagauucgcuuuu..................    41      0    S05
.......................................................................caccgggugaaagauucgcu.....................    1       0    S05
.......................................................................caccgggugaaagauucgcuu....................    31      0    S05
.......................................................................caccgggugaaagauucgcuuu...................    2       0    S05
........................................................................accgggugaaagauucgcuu....................    1       0    S05
.........................................................................ccgggugaaagauucgcuu....................    15      0    S05
.........................................................................ccgggugaaagauucgcuuu...................    2       0    S05
..........................................................................cgggugaaagauucgcuu....................    6       0    S05

........uuuuauguugggcauuuuuugaugaau.............................................................................    1       0    S07
..........uuauguugggcauuuuuugaugaau.............................................................................    1       0    S07
...............uugggcauuuuuugaugaau.............................................................................    1       0    S07
...................................ucgagucuuucgcuucggu..........................................................    1       0    S07
...................................ucgagucuuucgcuucggua.........................................................    8       0    S07
...................................ucgagucuuucgcuucgguaa........................................................    7       0    S07
...................................ucgagucuuucgcuucgguaau.......................................................    109     0    S07
...................................ucgagucuuucgcuucgguaauu......................................................    329     0    S07
...................................ucgagucuuucgcuucgguaauuuauaau................................................    1       0    S07
...................................ucgagucuuucgcuucgguaauuuauaaug...............................................    1       0    S07
...................................ucgagucuuucgcuucgguaauuuauaaugaau............................................    4       0    S07
...................................ucgagucuuucgcuucgguaauuuauaaugaaua...........................................    2       0    S07
...................................ucgagucuuucgcuucgguaauuuauaaugaauaa..........................................    2       0    S07
...................................ucgagucuuucgcuucgguaauuuauaaugaauaau.........................................    3       0    S07
.....................................gagucuuucgcuucgguaauu......................................................    1       0    S07
........................................ucuuucgcuucgguaauu......................................................    1       0    S07
............................................ucgcuucgguaauuuauaaugaau............................................    1       0    S07
.............................................cgcuucgguaauuuauaaugaa.............................................    1       0    S07
...............................................cuucgguaauuuauaaugaau............................................    1       0    S07
................................................uucgguaauuuauaaugaauaau.........................................    1       0    S07
..........................................................uauaaugaauaaucaccgggugaaagauucgcuu....................    1       0    S07
................................................................gaauaaucaccgggugaaagauucgcuu....................    1       0    S07
......................................................................ucaccgggugaaagauuc........................    5       0    S07
......................................................................ucaccgggugaaagauucgc......................    29      0    S07
......................................................................ucaccgggugaaagauucgcu.....................    2415    0    S07
......................................................................ucaccgggugaaagauucgcuu....................    28527   0    S07
......................................................................ucaccgggugaaagauucgcuuu...................    2225    0    S07
......................................................................ucaccgggugaaagauucgcuuuu..................    77      0    S07
.......................................................................caccgggugaaagauucgcu.....................    5       0    S07
.......................................................................caccgggugaaagauucgcuu....................    57      0    S07
.......................................................................caccgggugaaagauucgcuuu...................    3       0    S07
.......................................................................caccgggugaaagauucgcuuuu..................    1       0    S07
........................................................................accgggugaaagauucgcuu....................    3       0    S07
.........................................................................ccgggugaaagauucgcuu....................    26      0    S07
.........................................................................ccgggugaaagauucgcuuu...................    4       0    S07
..........................................................................cgggugaaagauucgcuu....................    13      0    S07
..........................................................................cgggugaaagauucgcuuu...................    4       0    S07

......................................................................ucaccgggugaaagauucgcu.....................    1       0    S01
......................................................................ucaccgggugaaagauucgcuu....................    15      0    S01
......................................................................ucaccgggugaaagauucgcuuu...................    1       0    S01

...............uugggcauuuuuugaugaau.............................................................................    2       0    S06
...................................ucgagucuuucgcuucggu..........................................................    1       0    S06
...................................ucgagucuuucgcuucggua.........................................................    19      0    S06
...................................ucgagucuuucgcuucgguaa........................................................    12      0    S06
...................................ucgagucuuucgcuucgguaau.......................................................    195     0    S06
...................................ucgagucuuucgcuucgguaauu......................................................    292     0    S06
...................................ucgagucuuucgcuucgguaauuuauaaugaau............................................    1       0    S06
...................................ucgagucuuucgcuucgguaauuuauaaugaauaau.........................................    1       0    S06
.....................................gagucuuucgcuucgguaa........................................................    1       0    S06
.....................................gagucuuucgcuucgguaauu......................................................    1       0    S06
..............................................gcuucgguaauuuauaaugaau............................................    1       0    S06
..........................................................uauaaugaauaaucaccgggugaaagauucgcuu....................    1       0    S06
.................................................................aauaaucaccgggugaaagauucgcuu....................    2       0    S06
....................................................................aaucaccgggugaaagauucgcuu....................    1       0    S06
......................................................................ucaccgggugaaagauuc........................    1       0    S06
```

```
                                   Star                               Mature
uacagaacuuuuauguugggcauuuuuugaugaauucgagucuuucgcuucgguaauuuauaaaugaauaaucaccgggugaaagauucgcuuuuauugaagaauaugucugc
.......................................................................ucaccgggugaaagauucgc......................   18      0   S06
.......................................................................ucaccgggugaaagauucgcu.....................   996     0   S06
.......................................................................ucaccgggugaaagauucgcuu....................   12650   0   S06
.......................................................................ucaccgggugaaagauucgcuuu...................   1268    0   S06
.......................................................................ucaccgggugaaagauucgcuuuu..................   31      0   S06
........................................................................caccgggugaaagauucgcu.....................   2       0   S06
........................................................................caccgggugaaagauucgcuu....................   44      0   S06
........................................................................caccgggugaaagauucgcuuu...................   3       0   S06
..........................................................................ccgggugaaagauucgcuu....................   16      0   S06
..........................................................................ccgggugaaagauucgcuuu...................   4       0   S06
...........................................................................cgggugaaagauucgcuu....................   11      0   S06
```

Provisional ID	:	Scaffold5285_44879
Score total	:	125943.9
Score for star read(s)	:	3.9
Score for read counts	:	125937.1
Score for mfe	:	1.3
Score for randfold	:	1.6
Score for cons. seed	:	
Total read count	:	247032
Mature read count	:	243778
Loop read count	:	0
Star read count	:	3254

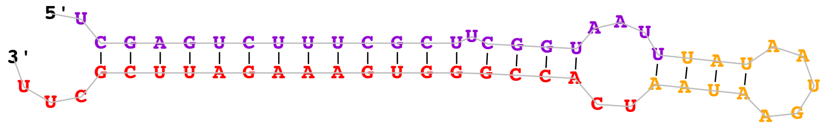

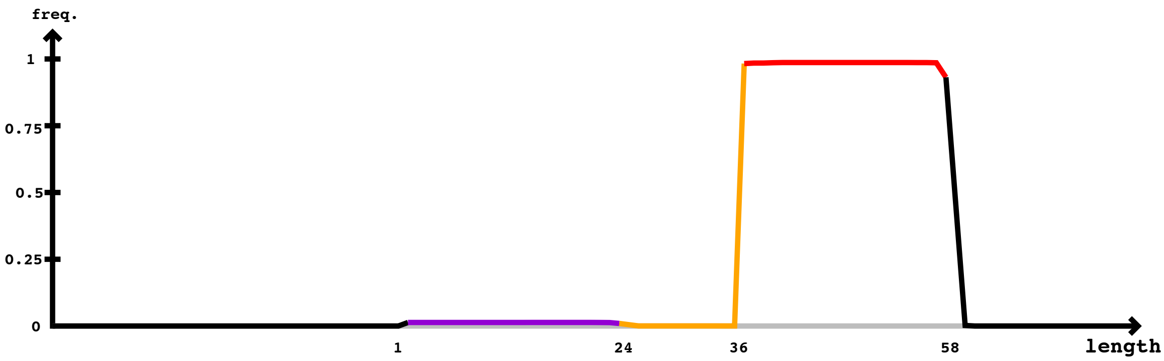

Star Mature

```
5'- uacagaacuuuuauguugagcauuuuuugaugaauucgagucuuucgcuucgguaauuuauaaugaauaaucaccgggugaaagauucgcuuuuauugaagaauuuugucug  -3'   obs
    uacagaacuuuuauguugagcauuuuuugaugaauucgagucuuucgcuucgguaauuuauaaugaauaaucaccgggugaaagauucgcuuuuauugaagaauuuugucug         exp
    ..((((.(((.......))).((((((..(((((..(((((((((((((.((((...((((.....))))..)))))))))))))))))..)))))..))))))...))))   reads   mm   sample
    ...................................ucgagucuuucgcuucgguaau.......................................................   1       0    S02
    ......................................................................ucaccgggugaaagauucgcu.....................   1       0    S02
    ......................................................................ucaccgggugaaagauucgcuu....................   41      0    S02
    ......................................................................ucaccgggugaaagauucgcuuu...................   2       0    S02

    ................................aauucgagucuuucgcuucggua.........................................................   1       0    S04
    ...................................ucgagucuuucgcuucggu..........................................................   2       0    S04
    ...................................ucgagucuuucgcuucggua.........................................................   3       0    S04
    ...................................ucgagucuuucgcuucgguaa........................................................   4       0    S04
    ...................................ucgagucuuucgcuucgguaau.......................................................   41      0    S04
    ...................................ucgagucuuucgcuucgguaauu......................................................   165     0    S04
    ...................................ucgagucuuucgcuucgguaauuuauaaugaauaa..........................................   2       0    S04
    .............................................cgcuucgguaauuuauaaugaa.............................................   1       0    S04
    .................................................................aauaaucaccgggugaaagauucgcuu....................   1       0    S04
    ...................................................................uaaucaccgggugaaagauucgcuu....................   1       0    S04
    ......................................................................ucaccgggugaaagauuc........................   1       0    S04
    ......................................................................ucaccgggugaaagauucgc......................   5       0    S04
    ......................................................................ucaccgggugaaagauucgcu.....................   333     0    S04
    ......................................................................ucaccgggugaaagauucgcuu....................   7084    0    S04
    ......................................................................ucaccgggugaaagauucgcuuu...................   709     0    S04
    ......................................................................ucaccgggugaaagauucgcuuuu..................   26      0    S04
    .......................................................................caccgggugaaagauucgcu.....................   1       0    S04
    .......................................................................caccgggugaaagauucgcuu....................   25      0    S04
    .........................................................................ccgggugaaagauucgcuu....................   6       0    S04
    .........................................................................ccgggugaaagauucgcuuu...................   2       0    S04
    ..........................................................................cgggugaaagauucgcuu....................   1       0    S04

    ...................................ucgagucuuucgcuucggu..........................................................   2       0    S08
    ...................................ucgagucuuucgcuucggua.........................................................   16      0    S08
    ...................................ucgagucuuucgcuucgguaa........................................................   19      0    S08
    ...................................ucgagucuuucgcuucgguaau.......................................................   265     0    S08
    ...................................ucgagucuuucgcuucgguaauu......................................................   641     0    S08
    ...................................ucgagucuuucgcuucgguaauuuauaaug...............................................   1       0    S08
```

Star **Mature**

```
uacagaacuuuuauguugagcauuuuuugaugaauucgagucuuucgcuucgguaauuuauaaaugaauaaucaccgggugaaagauucgcuuuuauugaagaauuugucug
...................................ucgagucuuucgcuucgguaauuuauaaaugaau...........................................  6      0  S08
...................................ucgagucuuucgcuucgguaauuuauaaaugaauaau........................................  1      0  S08
................................................uucgguaauuuauaaaugaauaa.........................................  1      0  S08
................................................................ugaauaaucaccgggugaaagauucgcuu...................  1      0  S08
.................................................................gaauaaucaccgggugaaagauucgcuu...................  1      0  S08
.....................................................................aaucaccgggugaaagauucgcuu...................  4      0  S08
.....................................................................aaucaccgggugaaagauucgcuuu..................  1      0  S08
......................................................................aucaccgggugaaagauucgcu....................  1      0  S08
......................................................................aucaccgggugaaagauucgcuu...................  2      0  S08
.......................................................................ucaccgggugaaagauuc.......................  15     0  S08
.......................................................................ucaccgggugaaagauucg......................  1      0  S08
.......................................................................ucaccgggugaaagauucgc.....................  39     0  S08
.......................................................................ucaccgggugaaagauucgcu....................  2407   0  S08
.......................................................................ucaccgggugaaagauucgcuu...................  40132  0  S08
.......................................................................ucaccgggugaaagauucgcuuu..................  2800   0  S08
.......................................................................ucaccgggugaaagauucgcuuuu.................  71     0  S08
.......................................................................ucaccgggugaaagauucgcuuuua................  1      0  S08
........................................................................caccgggugaaagauucgcu....................  7      0  S08
........................................................................caccgggugaaagauucgcuu...................  86     0  S08
........................................................................caccgggugaaagauucgcuuu..................  7      0  S08
.........................................................................accgggugaaagauucgcuu...................  6      0  S08
..........................................................................ccgggugaaagauucgcu....................  5      0  S08
..........................................................................ccgggugaaagauucgcuu...................  143    0  S08
..........................................................................ccgggugaaagauucgcuuu..................  12     0  S08
...........................................................................cgggugaaagauucgcuu...................  91     0  S08
...........................................................................cgggugaaagauucgcuuu..................  6      0  S08
............................................................................gggugaaagauucgcuuu..................  2      0  S08

...................................ucgagucuuucgcuucggua.........................................................  3      0  S03
...................................ucgagucuuucgcuucgguaa........................................................  6      0  S03
...................................ucgagucuuucgcuucgguaau.......................................................  52     0  S03
...................................ucgagucuuucgcuucgguaauu......................................................  164    0  S03
......................................agucuuucgcuucgguaauu......................................................  1      0  S03
.................................................................gaauaaucaccgggugaaagauucgcuu...................  1      0  S03
..................................................................aauaaucaccgggugaaagauucgcuu...................  2      0  S03
....................................................................uaaucaccgggugaaagauucgcuuu..................  1      0  S03
.....................................................................aaucaccgggugaaagauucgcuu...................  1      0  S03
.......................................................................ucaccgggugaaagauuc.......................  1      0  S03
.......................................................................ucaccgggugaaagauucgc.....................  8      0  S03
.......................................................................ucaccgggugaaagauucgcu....................  274    0  S03
.......................................................................ucaccgggugaaagauucgcuu...................  6514   0  S03
.......................................................................ucaccgggugaaagauucgcuuu..................  512    0  S03
.......................................................................ucaccgggugaaagauucgcuuuu.................  28     0  S03
........................................................................caccgggugaaagauucgcu....................  1      0  S03
........................................................................caccgggugaaagauucgcuu...................  13     0  S03
........................................................................caccgggugaaagauucgcuuu..................  4      0  S03
.........................................................................accgggugaaagauucgcuu...................  1      0  S03
..........................................................................ccgggugaaagauucgcuu...................  6      0  S03
..........................................................................ccgggugaaagauucgcuuu..................  3      0  S03
...........................................................................cgggugaaagauucgcuu...................  1      0  S03

...................................ucgagucuuucgcuucggua.........................................................  2      0  S09
...................................ucgagucuuucgcuucgguaa........................................................  3      0  S09
...................................ucgagucuuucgcuucgguaau.......................................................  27     0  S09
...................................ucgagucuuucgcuucgguaauu......................................................  212    0  S09
...................................ucgagucuuucgcuucgguaauuu.....................................................  1      0  S09
...................................ucgagucuuucgcuucgguaauuuauaaaugaauaa.........................................  1      0  S09
.....................................gagucuuucgcuucgguaau.......................................................  1      0  S09
............................................ucgcuucgguaauuuauaaauga..............................................  1      0  S09
.............................................cgcuucgguaauuuauaaaugaa.............................................  1      0  S09
................................................uucgguaauuuauaaaugaaua..........................................  1      0  S09
................................................uucgguaauuuauaaaugaauaa.........................................  3      0  S09
.................................................ucgguaauuuauaaaugaauaau........................................  2      0  S09
..........................................................uauaaaugaauaaucaccgggugaaagauucgcuu...................  1      0  S09
.....................................................................aaucaccgggugaaagauucgcuu...................  1      0  S09
......................................................................aucaccgggugaaagauucgcuu...................  1      0  S09
.......................................................................ucaccgggugaaagauuc.......................  10     0  S09
.......................................................................ucaccgggugaaagauucg......................  1      0  S09
.......................................................................ucaccgggugaaagauucgc.....................  24     0  S09
.......................................................................ucaccgggugaaagauucgcu....................  2797   0  S09
```

```
                                   Star                              Mature
uacagaacuuuuauguugagcauuuuuugaugaauucgagucuuucgcuucgguaauuuauaaaugaauaaucaccgggugaaagauucgcuuuuauugaagaauuugucug
.......................................................................ucaccgggugaaagauucgcuu...................   43943   0   S09
.......................................................................ucaccgggugaaagauucgcuuu..................   2720    0   S09
.......................................................................ucaccgggugaaagauucgcuuuu.................   84      0   S09
........................................................................caccgggugaaagauucgcu....................   6       0   S09
........................................................................caccgggugaaagauucgcuu...................   60      0   S09
........................................................................caccgggugaaagauucgcuuu..................   11      0   S09
.........................................................................accgggugaaagauucgcuu...................   11      0   S09
..........................................................................ccgggugaaagauucgcuu...................   16      0   S09
...........................................................................cgggugaaagauucgcuu...................   10      0   S09

...................................ucgagucuuucgcuucggu..........................................................   1       0   S07
...................................ucgagucuuucgcuucggua.........................................................   8       0   S07
...................................ucgagucuuucgcuucgguaa........................................................   7       0   S07
...................................ucgagucuuucgcuucgguaau.......................................................   109     0   S07
...................................ucgagucuuucgcuucgguaauu......................................................   329     0   S07
...................................ucgagucuuucgcuucgguaauuuauaaau...............................................   1       0   S07
...................................ucgagucuuucgcuucgguaauuuauaaaug..............................................   1       0   S07
...................................ucgagucuuucgcuucgguaauuuauaaaugaau...........................................   4       0   S07
...................................ucgagucuuucgcuucgguaauuuauaaaugaaua..........................................   2       0   S07
...................................ucgagucuuucgcuucgguaauuuauaaaugaauaa.........................................   2       0   S07
...................................ucgagucuuucgcuucgguaauuuauaaaugaauaau........................................   3       0   S07
.....................................gagucuuucgcuucgguaauu......................................................   1       0   S07
........................................ucuuucgcuucgguaauu......................................................   1       0   S07
............................................ucgcuucgguaauuuauaaaugaau...........................................   1       0   S07
.............................................cgcuucgguaauuuauaaaugaa............................................   1       0   S07
...............................................cuucgguaauuuauaaaugaau...........................................   1       0   S07
................................................uucgguaauuuauaaaugaauaau........................................   1       0   S07
..........................................................uauaaaugaauaaucaccgggugaaagauucgcuu...................   1       0   S07
.................................................................gaauaaucaccgggugaaagauucgcuu...................   1       0   S07
.......................................................................ucaccgggugaaagauuc.......................   5       0   S07
.......................................................................ucaccgggugaaagauucgc.....................   29      0   S07
.......................................................................ucaccgggugaaagauucgcu....................   2415    0   S07
.......................................................................ucaccgggugaaagauucgcuu...................   28527   0   S07
.......................................................................ucaccgggugaaagauucgcuuu..................   2225    0   S07
.......................................................................ucaccgggugaaagauucgcuuuu.................   77      0   S07
........................................................................caccgggugaaagauucgcu....................   5       0   S07
........................................................................caccgggugaaagauucgcuu...................   57      0   S07
........................................................................caccgggugaaagauucgcuuu..................   3       0   S07
........................................................................caccgggugaaagauucgcuuuu.................   1       0   S07
.........................................................................accgggugaaagauucgcuu...................   3       0   S07
..........................................................................ccgggugaaagauucgcuu...................   26      0   S07
..........................................................................ccgggugaaagauucgcuuu..................   4       0   S07
...........................................................................cgggugaaagauucgcuu...................   13      0   S07
...........................................................................cgggugaaagauucgcuuu..................   4       0   S07

...................................ucgagucuuucgcuucggu..........................................................   1       0   S06
...................................ucgagucuuucgcuucggua.........................................................   19      0   S06
...................................ucgagucuuucgcuucgguaa........................................................   12      0   S06
...................................ucgagucuuucgcuucgguaau.......................................................   195     0   S06
...................................ucgagucuuucgcuucgguaauu......................................................   292     0   S06
...................................ucgagucuuucgcuucgguaauuuauaaaugaau...........................................   1       0   S06
...................................ucgagucuuucgcuucgguaauuuauaaaugaauaau........................................   1       0   S06
.....................................gagucuuucgcuucgguaa........................................................   1       0   S06
.....................................gagucuuucgcuucgguaauu......................................................   1       0   S06
..............................................gcuucgguaauuuauaaaugaau...........................................   1       0   S06
..........................................................uauaaaugaauaaucaccgggugaaagauucgcuu...................   1       0   S06
..................................................................aauaaucaccgggugaaagauucgcuu...................   2       0   S06
.....................................................................aaucaccgggugaaagauucgcuu...................   1       0   S06
.......................................................................ucaccgggugaaagauuc.......................   1       0   S06
.......................................................................ucaccgggugaaagauucgc.....................   18      0   S06
.......................................................................ucaccgggugaaagauucgcu....................   996     0   S06
.......................................................................ucaccgggugaaagauucgcuu...................   12650   0   S06
.......................................................................ucaccgggugaaagauucgcuuu..................   1268    0   S06
.......................................................................ucaccgggugaaagauucgcuuuu.................   31      0   S06
........................................................................caccgggugaaagauucgcu....................   2       0   S06
........................................................................caccgggugaaagauucgcuu...................   44      0   S06
........................................................................caccgggugaaagauucgcuuu..................   3       0   S06
..........................................................................ccgggugaaagauucgcuu...................   16      0   S06
..........................................................................ccgggugaaagauucgcuuu..................   4       0   S06
...........................................................................cgggugaaagauucgcuu...................   11      0   S06
```

Star Mature

```
uacagaacuuuuauguugagcauuuuuugaugaauucgagucuuucgcuucgguaauuuauaaugaauaaucaccgggugaaagauucgcuuuuauugaagaauuugucug

......................................................................ucaccgggugaaagauucgcu....................  1      0  S01
......................................................................ucaccgggugaaagauucgcuu...................  15     0  S01
......................................................................ucaccgggugaaagauucgcuuu..................  1      0  S01

...................................ucgagucuuucgcuucgg..........................................................  1      0  S10
...................................ucgagucuuucgcuucggua........................................................  2      0  S10
...................................ucgagucuuucgcuucgguaa.......................................................  4      0  S10
...................................ucgagucuuucgcuucgguaau......................................................  64     0  S10
...................................ucgagucuuucgcuucgguaauu.....................................................  306    0  S10
...................................ucgagucuuucgcuucgguaauuu....................................................  2      0  S10
...................................ucgagucuuucgcuucgguaauuuauaau...............................................  1      0  S10
...................................ucgagucuuucgcuucgguaauuuauaaug..............................................  1      0  S10
...................................ucgagucuuucgcuucgguaauuuauaaugaa............................................  1      0  S10
...................................ucgagucuuucgcuucgguaauuuauaaugaau...........................................  1      0  S10
...................................ucgagucuuucgcuucgguaauuuauaaugaauaau........................................  1      0  S10
.....................................gagucuuucgcuucgguaau......................................................  1      0  S10
............................................ucgcuucgguaauuuauaaugaau...........................................  1      0  S10
.............................................cgcuucgguaauuuauaaugaa............................................  1      0  S10
.............................................cgcuucgguaauuuauaaugaau...........................................  1      0  S10
................................................uucgguaauuuauaaugaaua..........................................  3      0  S10
................................................uucgguaauuuauaaugaauaa.........................................  3      0  S10
................................................uucgguaauuuauaaugaauaau........................................  3      0  S10
....................................................................aaucaccgggugaaagauucgcuu...................  2      0  S10
.....................................................................aucaccgggugaaagauucgcu....................  1      0  S10
.....................................................................aucaccgggugaaagauucgcuu...................  1      0  S10
......................................................................ucaccgggugaaagauuc.......................  11     0  S10
......................................................................ucaccgggugaaagauucg......................  1      0  S10
......................................................................ucaccgggugaaagauucgc.....................  47     0  S10
......................................................................ucaccgggugaaagauucgcu....................  3777   0  S10
......................................................................ucaccgggugaaagauucgcuu...................  65221  0  S10
......................................................................ucaccgggugaaagauucgcuuu..................  4535   0  S10
......................................................................ucaccgggugaaagauucgcuuuu.................  149    0  S10
.......................................................................caccgggugaaagauucgcu....................  6      0  S10
.......................................................................caccgggugaaagauucgcuu...................  81     0  S10
.......................................................................caccgggugaaagauucgcuuu..................  16     0  S10
........................................................................accgggugaaagauucgcu....................  2      0  S10
........................................................................accgggugaaagauucgcuu...................  9      0  S10
.........................................................................ccgggugaaagauucgcuu...................  35     0  S10
.........................................................................ccgggugaaagauucgcuuu..................  4      0  S10
..........................................................................cgggugaaagauucgcuu...................  17     0  S10
..........................................................................cgggugaaagauucgcuuu..................  2      0  S10

................................aauucgagucuuucgcuucggua........................................................  1      0  S05
...................................ucgagucuuucgcuucggu.........................................................  1      0  S05
...................................ucgagucuuucgcuucggua........................................................  1      0  S05
...................................ucgagucuuucgcuucgguaa.......................................................  2      0  S05
...................................ucgagucuuucgcuucgguaau......................................................  58     0  S05
...................................ucgagucuuucgcuucgguaauu.....................................................  204    0  S05
...................................ucgagucuuucgcuucgguaauuuauaaugaauaau........................................  1      0  S05
..................................................................auaaucaccgggugaaagauucgcuu...................  1      0  S05
......................................................................ucaccgggugaaagauucgc.....................  7      0  S05
......................................................................ucaccgggugaaagauucgcu....................  432    0  S05
......................................................................ucaccgggugaaagauucgcuu...................  8850   0  S05
......................................................................ucaccgggugaaagauucgcuuu..................  879    0  S05
......................................................................ucaccgggugaaagauucgcuuuu.................  41     0  S05
.......................................................................caccgggugaaagauucgcu....................  1      0  S05
.......................................................................caccgggugaaagauucgcuu...................  31     0  S05
.......................................................................caccgggugaaagauucgcuuu..................  2      0  S05
........................................................................accgggugaaagauucgcuu...................  1      0  S05
.........................................................................ccgggugaaagauucgcuu...................  15     0  S05
.........................................................................ccgggugaaagauucgcuuu..................  2      0  S05
..........................................................................cgggugaaagauucgcuu...................  6      0  S05
```

Provisional ID : Scaffold248_6724
Score total : 30617.8
Score for star read(s) : 3.9
Score for read counts : 30612.1
Score for mfe : 0.2
Score for randfold : 1.6
Score for cons. seed :
Total read count : 60056
Mature read count : 55185
Loop read count : 0
Star read count : 4871

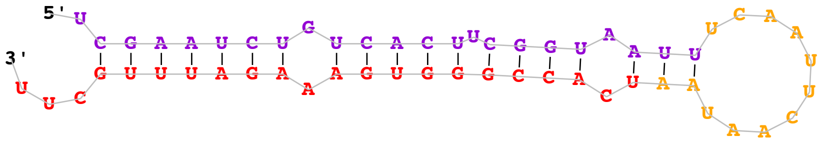

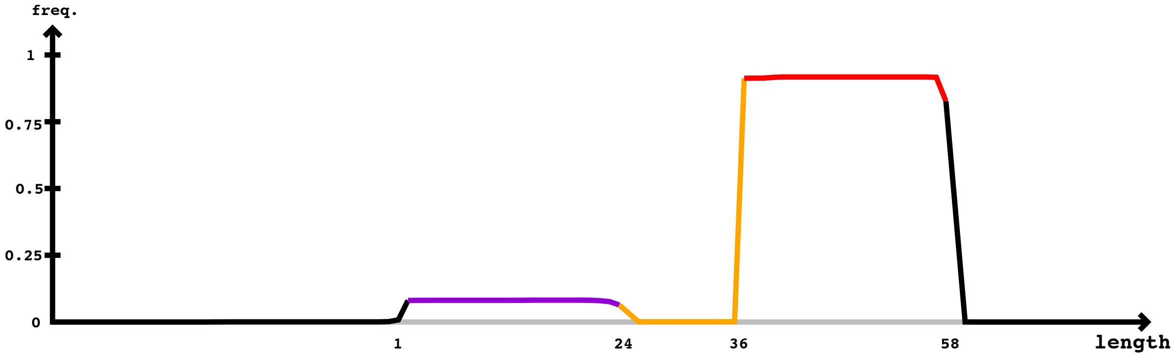

Star Mature

```
5'- ggaaucgugccuguuuuguguaauuuuugauggauucgaaucugucacuucgguaauuucaauucaauaaucaccgggugaaagauuugcuuucauuaagaaaauagaucgu  -3'  obs
    ggaaucgugccuguuuuguguaauuuuugauggauucgaaucugucacuucgguaauuucaauucaauaaucaccgggugaaagauuugcuuucauuaagaaaauagaucgu        exp
    ............(.(((((....(((((((((((..(((((((.(((((.((((.(((..........))).))))))))).)))))))..))))))))))).))))).)..  reads  mm  sample
    ...............uuguguaauuuuugauggau.............................................................................  4      0   S06
    .................................auucgaaucugucacuucgguaauu......................................................  1      0   S06
    ..................................uucgaaucugucacuucgguaau.......................................................  4      0   S06
    ..................................uucgaaucugucacuucgguaauu......................................................  13     0   S06
    ..................................uucgaaucugucacuucgguaauuu.....................................................  1      0   S06
    ...................................ucgaaucugucacuucgg...........................................................  1      0   S06
    ...................................ucgaaucugucacuucggu..........................................................  2      0   S06
    ...................................ucgaaucugucacuucggua.........................................................  6      0   S06
    ...................................ucgaaucugucacuucgguaa........................................................  22     0   S06
    ...................................ucgaaucugucacuucgguaau.......................................................  23     0   S06
    ...................................ucgaaucugucacuucgguaauu......................................................  130    0   S06
    ...................................ucgaaucugucacuucgguaauuucaauucaauaau.........................................  2      0   S06
    ....................................cgaaucugucacuucgguaauu......................................................  1      0   S06
    .....................................gaaucugucacuucgguaauu......................................................  3      0   S06
    ......................................aaucugucacuucgguaau.......................................................  1      0   S06
    .............................................cacuucgguaauuucaauucaau............................................  1      0   S06
    ...............................................cuucgguaauuucaauucaau............................................  1      0   S06
    ................................................uucgguaauuucaauucaauaa..........................................  3      0   S06
    ...............................................................ucaauaaucaccgggugaaagauuugcuu....................  1      0   S06
    ....................................................................aaucaccgggugaaagauuugcuu....................  5      0   S06
    ......................................................................ucaccgggugaaagauuugc......................  3      0   S06
    ......................................................................ucaccgggugaaagauuugcu.....................  364    0   S06
    ......................................................................ucaccgggugaaagauuugcuu....................  2420   0   S06
    ......................................................................ucaccgggugaaagauuugcuuu...................  174    0   S06
    ......................................................................ucaccgggugaaagauuugcuuuc..................  1      0   S06
    .......................................................................caccgggugaaagauuugcuu....................  3      0   S06
    ........................................................................accgggugaaagauuugcuuuc..................  1      0   S06
    .........................................................................ccgggugaaagauuugcu.....................  1      0   S06
    .........................................................................ccgggugaaagauuugcuu....................  9      0   S06
    .........................................................................ccgggugaaagauuugcuuu...................  1      0   S06
    ..........................................................................cgggugaaagauuugcuu....................  6      0   S06
    ..........................................................................cgggugaaagauuugcuuu...................  1      0   S06

    ......................................................................ucaccgggugaaagauuugcu.....................  2      0   S01
```

Star   Mature

ggaaucgugccuguuuuguguaauuuuugauggauucgaaucugucacuucgguaauuucaauucaauaaucaccgggugaaagauuugcuuucauuaagaaaauagaucgu

...............uuguguaauuuuugauggau...	2	0	S07
.................guguaauuuuugauggau...	1	0	S07
................................gauucgaaucugucacuucggu..	2	0	S07
.................................auucgaaucugucacuucggua...	1	0	S07
.................................auucgaaucugucacuucgguaa..	8	0	S07
.................................auucgaaucugucacuucgguaau...	2	0	S07
.................................auucgaaucugucacuucgguaauu..	4	0	S07
..................................uucgaaucugucacuucgguaa..	5	0	S07
..................................uucgaaucugucacuucgguaau...	11	0	S07
..................................uucgaaucugucacuucgguaauu..	40	0	S07
...................................ucgaaucugucacuucgg...	1	0	S07
...................................ucgaaucugucacuucggu..	3	0	S07
...................................ucgaaucugucacuucggua...	16	0	S07
...................................ucgaaucugucacuucgguaa..	45	0	S07
...................................ucgaaucugucacuucgguaau...	91	0	S07
...................................ucgaaucugucacuucgguaauu..	457	0	S07
...................................ucgaaucugucacuucgguaauuu...	1	0	S07
...................................ucgaaucugucacuucgguaauuucaauucaaua...	1	0	S07
...................................ucgaaucugucacuucgguaauuucaauucaauaaucaccgg...................................	1	0	S07
....................................cgaaucugucacuucgguaauu..	1	0	S07
.....................................gaaucugucacuucgguaauu..	1	0	S07
....acuucgguaauuucaauucaauaau...	1	0	S07
....uucgguaauuucaauucaauaa..	2	0	S07
....ucaauaaucaccgggugaaagauuugcuu....................	2	0	S07
....caauaaucaccgggugaaagauuugcuu....................	1	0	S07
....aauaaucaccgggugaaagauuugcuu....................	1	0	S07
....aauaaucaccgggugaaagauuugcuuu...................	1	0	S07
....aaucaccgggugaaagauuugcuu....................	1	0	S07
....aucaccgggugaaagauuugcuu....................	1	0	S07
....ucaccgggugaaagauuug.......................	1	0	S07
....ucaccgggugaaagauuugc......................	6	0	S07
....ucaccgggugaaagauuugcu.....................	850	0	S07
....ucaccgggugaaagauuugcuu....................	7504	0	S07
....ucaccgggugaaagauuugcuuu...................	325	0	S07
....caccgggugaaagauuugcuu....................	3	0	S07
....accgggugaaagauuugcuuu...................	1	0	S07
....ccgggugaaagauuugcu.....................	3	0	S07
....ccgggugaaagauuugcuu....................	11	0	S07
....cgggugaaagauuugcuu....................	8	0	S07
....cgggugaaagauuugcuuu...................	1	0	S07
...............uuguguaauuuuugauggau...	1	0	S05
................uguguaauuuuugauggau...	1	0	S05
.................guguaauuuuugauggau...	1	0	S05
.................................auucgaaucugucacuucggua...	1	0	S05
.................................auucgaaucugucacuucgguaa..	3	0	S05
..................................uucgaaucugucacuucgguaau...	2	0	S05
..................................uucgaaucugucacuucgguaauu..	11	0	S05
...................................ucgaaucugucacuucggu..	1	0	S05
...................................ucgaaucugucacuucggua...	1	0	S05
...................................ucgaaucugucacuucgguaa..	2	0	S05
...................................ucgaaucugucacuucgguaau...	4	0	S05
...................................ucgaaucugucacuucgguaauu..	55	0	S05
....................................cgaaucugucacuucgguaauu..	1	0	S05
.....................................gaaucugucacuucgguaauu..	1	0	S05
....acuucgguaauuucaauucaauaa..	1	0	S05
....ucaccgggugaaagauuugc......................	1	0	S05
....ucaccgggugaaagauuugcu.....................	163	0	S05
....ucaccgggugaaagauuugcuu....................	1703	0	S05
....ucaccgggugaaagauuugcuuu...................	162	0	S05
....ucaccgggugaaagauuugcuuuc..................	1	0	S05
....ccgggugaaagauuugcuu....................	7	0	S05
....ccgggugaaagauuugcuuu...................	2	0	S05
....cgggugaaagauuugcuu....................	3	0	S05
....gggugaaagauuugcuuu...................	1	0	S05
...............uuguguaauuuuugauggau...	5	0	S10
................uguguaauuuuugauggau...	1	0	S10
.................guguaauuuuugauggau...	1	0	S10

Star **Mature**

```
ggaaucgugccuguuuuguguaauuuuugauggauucgaaucugucacuucgguaauuucaauucaauaaucaccgggugaaagauuugcuuucauuaagaaaauagaucgu
................................gauucgaaucugucacuucggua.........................................................	1	0	S10
.................................auucgaaucugucacuucggua.........................................................	1	0	S10
.................................auucgaaucugucacuucgguaa........................................................	8	0	S10
.................................auucgaaucugucacuucgguaau.......................................................	2	0	S10
.................................auucgaaucugucacuucgguaauu......................................................	4	0	S10
..................................uucgaaucugucacuucgguaa........................................................	1	0	S10
..................................uucgaaucugucacuucgguaau.......................................................	17	0	S10
..................................uucgaaucugucacuucgguaauu......................................................	111	0	S10
..................................uucgaaucugucacuucgguaauuu.....................................................	4	0	S10
...................................ucgaaucugucacuucggu..........................................................	2	0	S10
...................................ucgaaucugucacuucggua.........................................................	23	0	S10
...................................ucgaaucugucacuucgguaa........................................................	26	0	S10
...................................ucgaaucugucacuucgguaau.......................................................	208	0	S10
...................................ucgaaucugucacuucgguaauu......................................................	1127	0	S10
...................................ucgaaucugucacuucgguaauuu.....................................................	1	0	S10
...................................ucgaaucugucacuucgguaauuucaauucaauaau.........................................	1	0	S10
....................................cgaaucugucacuucgguaau.......................................................	2	0	S10
....................................cgaaucugucacuucgguaauu......................................................	2	0	S10
...............................................cuucgguaauuucaauucaaua...........................................	1	0	S10
.................................................ucgguaauuucaauucaaua...........................................	2	0	S10
.................................................ucgguaauuucaauucaauaa..........................................	9	0	S10
.................................................ucgguaauuucaauucaauaau.........................................	1	0	S10
..................................................cgguaauuucaauucaauaa..........................................	2	0	S10
..................................................cgguaauuucaauucaauaau.........................................	1	0	S10
......................................................................ucaccgggugaaagauuug.......................	2	0	S10
......................................................................ucaccgggugaaagauuugc......................	22	0	S10
......................................................................ucaccgggugaaagauuugcu.....................	1573	0	S10
......................................................................ucaccgggugaaagauuugcuu....................	13898	0	S10
......................................................................ucaccgggugaaagauuugcuuu...................	835	0	S10
......................................................................ucaccgggugaaagauuugcuuuc..................	1	0	S10
.......................................................................caccgggugaaagauuugc......................	1	0	S10
.......................................................................caccgggugaaagauuugcuu....................	7	0	S10
........................................................................accgggugaaagauuugcu.....................	1	0	S10
........................................................................accgggugaaagauuugcuu....................	2	0	S10
.........................................................................ccgggugaaagauuugcu.....................	1	0	S10
.........................................................................ccgggugaaagauuugcuu....................	3	0	S10
.........................................................................ccgggugaaagauuugcuuu...................	2	0	S10
..........................................................................cgggugaaagauuugcuu....................	1	0	S10

...................................ucgaaucugucacuucgguaauu......................................................	2	0	S02
......................................................................ucaccgggugaaagauuugcu.....................	1	0	S02
......................................................................ucaccgggugaaagauuugcuu....................	8	0	S02

...............uuguguaauuuuugauggau.............................................................................	1	0	S04
................uguguaauuuuugauggau.............................................................................	2	0	S04
.................guguaauuuuugauggau.............................................................................	1	0	S04
................................gauucgaaucugucacuucggu..........................................................	1	0	S04
.................................auucgaaucugucacuucgguaa........................................................	1	0	S04
.................................auucgaaucugucacuucgguaau.......................................................	1	0	S04
..................................uucgaaucugucacuucgguaauu......................................................	7	0	S04
...................................ucgaaucugucacuucggu..........................................................	2	0	S04
...................................ucgaaucugucacuucgguaa........................................................	2	0	S04
...................................ucgaaucugucacuucgguaau.......................................................	8	0	S04
...................................ucgaaucugucacuucgguaauu......................................................	59	0	S04
...................................ucgaaucugucacuucgguaauuucaauucaauaau.........................................	1	0	S04
.............................................................auucaauaaucaccgggugaaagauuugcuuu...................	1	0	S04
......................................................................ucaccgggugaaagauuugc......................	2	0	S04
......................................................................ucaccgggugaaagauuugcu.....................	136	0	S04
......................................................................ucaccgggugaaagauuugcuu....................	1397	0	S04
......................................................................ucaccgggugaaagauuugcuuu...................	114	0	S04
.......................................................................caccgggugaaagauuugcuu....................	1	0	S04
.........................................................................ccgggugaaagauuugcuu....................	1	0	S04
.........................................................................ccgggugaaagauuugcuuu...................	2	0	S04
..........................................................................cgggugaaagauuugcuu....................	1	0	S04

...............uuguguaauuuuugauggau.............................................................................	1	0	S03
..................................uucgaaucugucacuucgguaauu......................................................	5	0	S03
...................................ucgaaucugucacuucgguaauu......................................................	30	0	S03
...................................ucgaaucugucacuucgguaauuucaauucaauaa..........................................	1	0	S03
...............................................................ucaauaaucaccgggugaaagauuugcuu....................	1	0	S03
```

Star Mature

```
ggaaucgugccuguuuuguguaauuuuugauggauucgaaucugucacuucgguaauuucaauucaauaaucaccgggugaaagauuugcuuucauuaagaaaauagaucgu
......................................................................ucaccgggugaaagauuugc......................   3      0   S03
......................................................................ucaccgggugaaagauuugcu.....................   156    0   S03
......................................................................ucaccgggugaaagauuugcuu....................   1488   0   S03
......................................................................ucaccgggugaaagauuugcuuu...................   151    0   S03
.......................................................................caccgggugaaagauuugcuu....................   2      0   S03
.........................................................................ccgggugaaagauuugcuu....................   1      0   S03
..........................................................................cgggugaaagauuugcuu....................   3      0   S03
..........................................................................cgggugaaagauuugcuuu...................   1      0   S03

...............uuguguaauuuuugauggau.............................................................................   3      0   S09
................uguguaauuuuugauggau.............................................................................   1      0   S09
................................gauucgaaucugucacuucggu..........................................................   1      0   S09
.................................auucgaaucugucacuucggu..........................................................   1      0   S09
.................................auucgaaucugucacuucggua.........................................................   1      0   S09
.................................auucgaaucugucacuucgguaa........................................................   8      0   S09
.................................auucgaaucugucacuucgguaau.......................................................   1      0   S09
.................................auucgaaucugucacuucgguaauu......................................................   3      0   S09
..................................uucgaaucugucacuucggua.........................................................   1      0   S09
..................................uucgaaucugucacuucgguaau.......................................................   33     0   S09
..................................uucgaaucugucacuucgguaauu......................................................   104    0   S09
...................................ucgaaucugucacuucggu..........................................................   3      0   S09
...................................ucgaaucugucacuucggua.........................................................   26     0   S09
...................................ucgaaucugucacuucgguaa........................................................   30     0   S09
...................................ucgaaucugucacuucgguaau.......................................................   238    0   S09
...................................ucgaaucugucacuucgguaauu......................................................   1200   0   S09
...................................ucgaaucugucacuucgguaauuu.....................................................   1      0   S09
...................................ucgaaucugucacuucgguaauuuc....................................................   1      0   S09
...................................ucgaaucugucacuucgguaauuucaauuc...............................................   1      0   S09
...................................ucgaaucugucacuucgguaauuucaauucaauaau.........................................   2      0   S09
....................................cgaaucugucacuucgguaauu......................................................   5      0   S09
......................................aaucugucacuucgguaau.......................................................   1      0   S09
......................................aaucugucacuucgguaauu......................................................   2      0   S09
......................................aaucugucacuucgguaauuucaauucaau............................................   1      0   S09
.............................................cacuucgguaauuucaauucaauaa..........................................   1      0   S09
...............................................cuucgguaauuucaauucaaua...........................................   1      0   S09
................................................uucgguaauuucaauucaaua...........................................   2      0   S09
................................................uucgguaauuucaauucaauaa..........................................   6      0   S09
................................................uucgguaauuucaauucaauaau.........................................   1      0   S09
...............................................................ucaauaaucaccgggugaaag............................   1      0   S09
.................................................................aauaaucaccgggugaaagauuugcuu....................   1      0   S09
.....................................................................aucaccgggugaaagauuugcuu....................   2      0   S09
......................................................................ucaccgggugaaagauuugc......................   12     0   S09
......................................................................ucaccgggugaaagauuugcu.....................   918    0   S09
......................................................................ucaccgggugaaagauuugcuu....................   8925   0   S09
......................................................................ucaccgggugaaagauuugcuuu...................   442    0   S09
......................................................................ucaccgggugaaagauuugcuuuc..................   4      0   S09
......................................................................ucaccgggugaaagauuugcuuuca.................   1      0   S09
.......................................................................caccgggugaaagauuugcuu....................   5      0   S09
........................................................................accgggugaaagauuugcuu....................   2      0   S09
.........................................................................ccgggugaaagauuugcuu....................   4      0   S09
.........................................................................ccgggugaaagauuugcuuu...................   1      0   S09
..........................................................................cgggugaaagauuugcuu....................   1      0   S09

ggaaucgugccuguuuuguguaauuuuugauggau.............................................................................   1      0   S08
..aaucgugccuguuuuguguaauuuuugauggau.............................................................................   1      0   S08
.................................auucgaaucugucacuucgguaa........................................................   2      0   S08
..................................uucgaaucugucacuucgguaau.......................................................   5      0   S08
..................................uucgaaucugucacuucgguaauu......................................................   23     0   S08
..................................uucgaaucugucacuucgguaauuu.....................................................   2      0   S08
...................................ucgaaucugucacuucggu..........................................................   5      0   S08
...................................ucgaaucugucacuucggua.........................................................   22     0   S08
...................................ucgaaucugucacuucgguaa........................................................   47     0   S08
...................................ucgaaucugucacuucgguaau.......................................................   105    0   S08
...................................ucgaaucugucacuucgguaauu......................................................   356    0   S08
...................................ucgaaucugucacuucgguaauuuc....................................................   3      0   S08
...................................ucgaaucugucacuucgguaauuucaauuc...............................................   2      0   S08
...................................ucgaaucugucacuucgguaauuucaauucaauaau.........................................   2      0   S08
....................................cgaaucugucacuucggua.........................................................   2      0   S08
....................................cgaaucugucacuucgguaa........................................................   2      0   S08
....................................cgaaucugucacuucgguaauu......................................................   1      0   S08
```

```
                                   Star                                Mature
ggaaucgugccuguuuuguguaauuuuugauggauucgaaucugucacuucgguaauuucaauucaauaaucaccgggugaaagauuugcuuucauuaagaaaauagaucgu
......................................aaucugucacuucgguaauu......................................................    3     0    S08
........................................ucugucacuucgguaauu......................................................    1     0    S08
................................................uucgguaauuucaauucaauaa..........................................    2     0    S08
..............................................................uucaauaaucaccgggugaaagauuugcuu....................    2     0    S08
....................................................................aucaccgggugaaagauuugcuu....................    1     0    S08
......................................................................ucaccgggugaaagauuug.......................    3     0    S08
......................................................................ucaccgggugaaagauuugc......................    20    0    S08
......................................................................ucaccgggugaaagauuugcu.....................    1204  0    S08
......................................................................ucaccgggugaaagauuugcuu....................    9442  0    S08
......................................................................ucaccgggugaaagauuugcuuu...................    433   0    S08
......................................................................ucaccgggugaaagauuugcuuuc..................    1     0    S08
.......................................................................caccgggugaaagauuugcu.....................    1     0    S08
.......................................................................caccgggugaaagauuugcuu....................    12    0    S08
........................................................................accgggugaaagauuugcuu....................    5     0    S08
.........................................................................ccgggugaaagauuugcu.....................    4     0    S08
.........................................................................ccgggugaaagauuugcuu....................    101   0    S08
.........................................................................ccgggugaaagauuugcuuu...................    4     0    S08
..........................................................................cgggugaaagauuugcuu....................    67    0    S08
..........................................................................cgggugaaagauuugcuuu...................    4     0    S08
```

Provisional ID	:	Scaffold248_6756
Score total	:	15405.7
Score for star read(s)	:	3.9
Score for read counts	:	15398.9
Score for mfe	:	1.3
Score for randfold	:	1.6
Score for cons. seed	:	
Total read count	:	30216
Mature read count	:	30064
Loop read count	:	0
Star read count	:	152

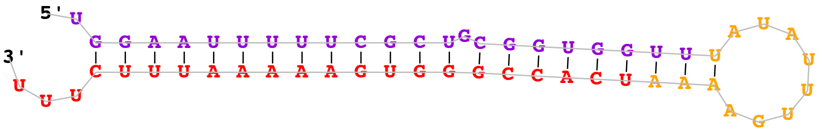

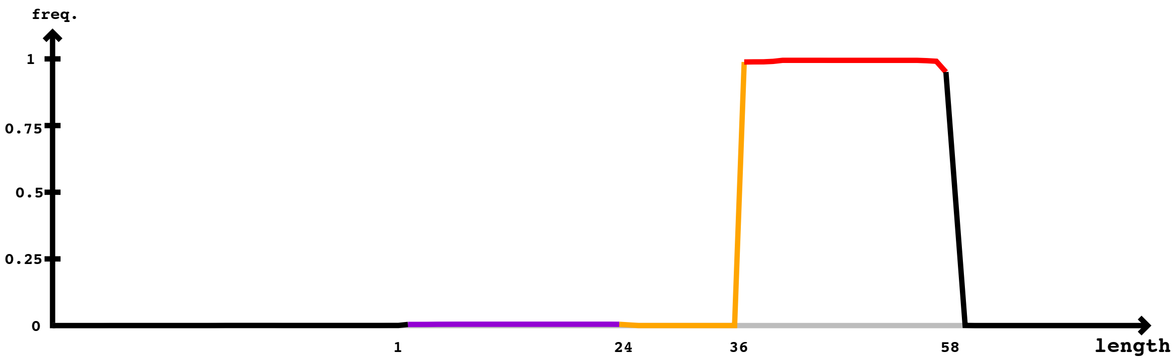

Star Mature

```
5'- guaaacuuuuauugugcauacuuuucuuuacagaauggaauuuuucgcugcggugguuuauauuugaaaaucaccgggugaaaaauuucuuucuucaaagaaauauuuaaca   -3'   obs
    guaaacuuuuauugugcauacuuuucuuuacagaauggaauuuuucgcugcggugguuuauauuugaaaaucaccgggugaaaaauuucuuucuucaaagaaauauuuaaca         exp
    ............(((.......((((((..((((.(((((((((((((.((((((((((........))))))))))))))))))))))).))))..)))))))......)))   reads   mm   sample
    ...............gcauacuuuucuuuacagaa.............................................................................   1       0    S04
    ................cauacuuuucuuuacagaa.............................................................................   3       0    S04
    ...................................uggaauuuuucgcugcggugguu......................................................   36      0    S04
    .....................................gaauuuuucgcugcggugguu......................................................   1       0    S04
    ......................................aauuuuucgcugcggugguu......................................................   1       0    S04
    ......................................................................ucaccgggugaaaaauuucu......................   4       0    S04
    ......................................................................ucaccgggugaaaaauuucuu.....................   87      0    S04
    ......................................................................ucaccgggugaaaaauuucuuu....................   2902    0    S04
    ......................................................................ucaccgggugaaaaauuucuuuc...................   9       0    S04
    ......................................................................ucaccgggugaaaaauuucuuucu..................   2       0    S04
    .......................................................................caccgggugaaaaauuucuuu....................   2       0    S04
    ........................................................................accgggugaaaaauuucuuu....................   1       0    S04
    .........................................................................ccgggugaaaaauuucuuu....................   1       0    S04
    ..........................................................................cgggugaaaaauuucuuu....................   7       0    S04

    ...................................uggaauuuuucgcugcggugguu......................................................   1       0    S02
    ......................................................................ucaccgggugaaaaauuuc.......................   5       0    S02
    ......................................................................ucaccgggugaaaaauuucu......................   1       0    S02
    ......................................................................ucaccgggugaaaaauuucuu.....................   9       0    S02
    ......................................................................ucaccgggugaaaaauuucuuu....................   264     0    S02
    ......................................................................ucaccgggugaaaaauuucuuuc...................   1       0    S02
    ......................................................................ucaccgggugaaaaauuucuuucu..................   1       0    S02
    .........................................................................ccgggugaaaaauuucuuu....................   2       0    S02

    .................................aauggaauuuuucgcugcgguggu.......................................................   1       0    S09
    ...................................uggaauuuuucgcugcggugguu......................................................   3       0    S09
    ...................................uggaauuuuucgcugcggugguuu.....................................................   1       0    S09
    ......................................................................ucaccgggugaaaaauuucu......................   4       0    S09
    ......................................................................ucaccgggugaaaaauuucuu.....................   110     0    S09
    ......................................................................ucaccgggugaaaaauuucuuu....................   2262    0    S09
    ......................................................................ucaccgggugaaaaauuucuuuc...................   5       0    S09
    .......................................................................caccgggugaaaaauuucuuu....................   1       0    S09
    ........................................................................accgggugaaaaauuucuuu....................   1       0    S09
```

```
                                   Star                       Mature
guaaacuuuuauugugcauacuuuucuuuacagaauggaauuuuucgcugcggugguuuauauuugaaaaucaccgggugaaaaauuucuuucuucaaagaaauauuuaaca
.........................................................................ccgggugaaaaauuucuuu....................	3	0	S09
..........................................................................cgggugaaaaauuucuuu....................	5	0	S09

...............gcauacuuuucuuuacagaa.............................................................................	1	0	S03
................cauacuuuucuuuacagaa.............................................................................	2	0	S03
...................................uggaauuuuucgcugcgguggu.......................................................	2	0	S03
...................................uggaauuuuucgcugcggugguu......................................................	13	0	S03
...................................uggaauuuuucgcugcggugguuu.....................................................	1	0	S03
......................................aauuuuucgcugcgguggu.......................................................	1	0	S03
........................................uuuuucgcugcggugguu......................................................	1	0	S03
....................................................................aaucaccgggugaaaaauuucuu.....................	1	0	S03
......................................................................ucaccgggugaaaaauuuc.......................	5	0	S03
......................................................................ucaccgggugaaaaauuucu......................	9	0	S03
......................................................................ucaccgggugaaaaauuucuu.....................	89	0	S03
......................................................................ucaccgggugaaaaauuucuuu....................	2871	0	S03
......................................................................ucaccgggugaaaaauuucuuuc...................	13	0	S03
......................................................................ucaccgggugaaaaauuucuuucu..................	2	0	S03
.......................................................................caccgggugaaaaauuucuuu....................	5	0	S03
.........................................................................ccgggugaaaaauuucuuu....................	1	0	S03
..........................................................................cgggugaaaaauuucuuu....................	12	0	S03

....aAuuuuauugugcauacu..........................................................................................	2	1	S08
...................................uggaauuuuucgcugcggugguu......................................................	6	0	S08
......................................aauuuuucgcugcggugguu......................................................	3	0	S08
......................................................................ucaccgggugaaaaauuu........................	1	0	S08
......................................................................ucaccgggugaaaaauuuc.......................	9	0	S08
......................................................................ucaccgggugaaaaauuucu......................	19	0	S08
......................................................................ucaccgggugaaaaauuucuu.....................	413	0	S08
......................................................................ucaccgggugaaaaauuucuuu....................	7302	0	S08
......................................................................ucaccgggugaaaaauuucuuuc...................	28	0	S08
......................................................................ucaccgggugaaaaauuucuuucu..................	1	0	S08
.........................................................................ccgggugaaaaauuucuuu....................	27	0	S08
..........................................................................cgggugaaaaauuucuuu....................	48	0	S08

................cauacuuuucuuuacagaa.............................................................................	1	0	S06
...................................uggaauuuuucgcugcgguggu.......................................................	1	0	S06
...................................uggaauuuuucgcugcggugguu......................................................	13	0	S06
...................................uggaauuuuucgcugcggugguuu.....................................................	1	0	S06
....................................ggaauuuuucgcugcggugguu......................................................	1	0	S06
.....................................gaauuuuucgcugcggugguu......................................................	1	0	S06
......................................aauuuuucgcugcggugguu......................................................	3	0	S06
..........................................................uauauuugaaaaucaccgggugaaaaauuucuuu....................	1	0	S06
...............................................................uugaaaaucaccgggugaaaaau..........................	1	0	S06
......................................................................ucaccgggugaaaaauuuc.......................	8	0	S06
......................................................................ucaccgggugaaaaauuucu......................	4	0	S06
......................................................................ucaccgggugaaaaauuucuu.....................	108	0	S06
......................................................................ucaccgggugaaaaauuucuuu....................	2804	0	S06
......................................................................ucaccgggugaaaaauuucuuuc...................	11	0	S06
......................................................................ucaccgggugaaaaauuucuuucu..................	1	0	S06
.........................................................................ccgggugaaaaauuucuuu....................	4	0	S06
..........................................................................cgggugaaaaauuucuuu....................	13	0	S06

......................................................................ucaccgggugaaaaauuuc.......................	1	0	S01
......................................................................ucaccgggugaaaaauuucu......................	1	0	S01
......................................................................ucaccgggugaaaaauuucuu.....................	14	0	S01
......................................................................ucaccgggugaaaaauuucuuu....................	207	0	S01
......................................................................ucaccgggugaaaaauuucuuucu..................	1	0	S01
.......................................................................caccgggugaaaaauuucu......................	2	0	S01
..........................................................................cgggugaaaaauuucuuu....................	1	0	S01

.................................aauggaauuuuucgcugcgg...........................................................	1	0	S07
...................................uggaauuuuucgcugcgguggu.......................................................	1	0	S07
...................................uggaauuuuucgcugcggugguu......................................................	23	0	S07
...................................uggaauuuuucgcugcggugguuu.....................................................	4	0	S07
......................................aauuuuucgcugcggugguu......................................................	2	0	S07
.......................................auuuuucgcugcggugguu......................................................	3	0	S07
........................................uuuuucgcugcggugguu......................................................	1	0	S07
.........................................................uuauauuugaaaaucaccgggu.................................	1	0	S07
......................................................................ucaccgggugaaaaauuuc.......................	5	0	S07
......................................................................ucaccgggugaaaaauuucu......................	10	0	S07
```

```
                                   Star                              Mature
guaaacuuuuauugugcauacuuuucuuuacagaauggaauuuuucgcugcggugguuuauauuugaaaaucaccgggugaaaaauuucuuucuucaaagaaauauuuaaca
......................................................................ucaccgggugaaaaauuucuu.....................  166   0  S07
......................................................................ucaccgggugaaaaauuucuuu....................  4076  0  S07
......................................................................ucaccgggugaaaaauuucuuuc...................  12    0  S07
......................................................................ucaccgggugaaaaauuucuuucu..................  4     0  S07
.........................................................................ccgggugaaaaauuucuuu....................  3     0  S07
..........................................................................cgggugaaaaauuucuuu....................  17    0  S07

...............gcauacuuuucuuuacagaa.............................................................................  1     0  S05
................cauacuuuucuuuacagaa.............................................................................  1     0  S05
...................................uggaauuuuucgcugcggugguu......................................................  13    0  S05
...................................uggaauuuuucgcugcggugguuu.....................................................  1     0  S05
.....................................gaauuuuucgcugcggugguu......................................................  1     0  S05
......................................aauuuuucgcugcggugguu......................................................  1     0  S05
........................................uuuuucgcugcggugguu......................................................  2     0  S05
......................................................................ucaccgggugaaaaauuuc.......................  4     0  S05
......................................................................ucaccgggugaaaaauuucu......................  5     0  S05
......................................................................ucaccgggugaaaaauuucuu.....................  88    0  S05
......................................................................ucaccgggugaaaaauuucuuu....................  2872  0  S05
......................................................................ucaccgggugaaaaauuucuuuc...................  16    0  S05
......................................................................ucaccgggugaaaaauuucuuucu..................  2     0  S05
.......................................................................caccgggugaaaaauuucuuu....................  3     0  S05
.........................................................................ccgggugaaaaauuucuuu....................  9     0  S05
..........................................................................cgggugaaaaauuucuuu....................  14    0  S05

...................................uggaauuuuucgcugcgguggu.......................................................  3     0  S10
...................................uggaauuuuucgcugcggugguu......................................................  5     0  S10
......................................................................ucaccgggugaaaaauuuc.......................  3     0  S10
......................................................................ucaccgggugaaaaauuucu......................  3     0  S10
......................................................................ucaccgggugaaaaauuucuu.....................  139   0  S10
......................................................................ucaccgggugaaaaauuucuuu....................  2871  0  S10
......................................................................ucaccgggugaaaaauuucuuuc...................  7     0  S10
......................................................................ucaccgggugaaaaauuucuuucu..................  2     0  S10
.......................................................................caccgggugaaaaauuucuuu....................  2     0  S10
........................................................................accgggugaaaaauuucuuu....................  1     0  S10
.........................................................................ccgggugaaaaauuucuuu....................  3     0  S10
..........................................................................cgggugaaaaauuucuuu....................  2     0  S10
```

Provisional ID	:	Scaffold248_6746
Score total	:	11502.5
Score for star read(s)	:	3.9
Score for read counts	:	11496.2
Score for mfe	:	0.9
Score for randfold	:	1.6
Score for cons. seed	:	
Total read count	:	22561
Mature read count	:	21604
Loop read count	:	0
Star read count	:	957

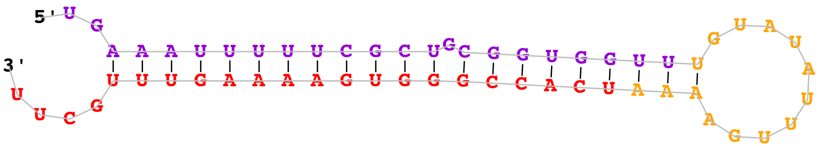

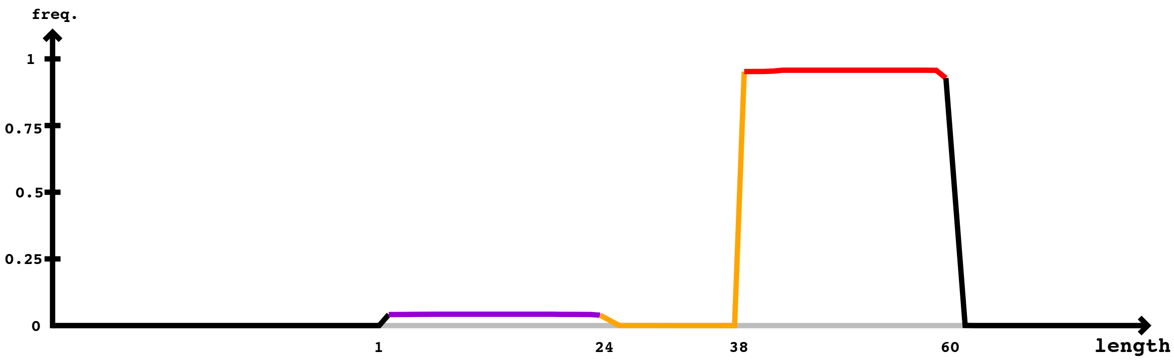


```
                                 Star                                Mature
5'- auccaaaaugcuggguuuaauuuucuuacaguaugaaauuuuucgcugcggugguuuguauauuugaaaaucaccgggugaaaaguuugcuucuguuuuaaaauuuuagcug  -3'   obs
    auccaaaaugcuggguuuaauuuucuuacaguaugaaauuuuucgcugcggugguuuguauauuugaaaaucaccgggugaaaaguuugcuucuguuuuaaaauuuuagcug         exp
    .........(((((....((((((...((((...(((((((((((((.(((((((((..........))))))))))))))))))))).)..))))...))))))))))).. reads  mm  sample
    .................................ugaaauuuuucgcugcggug........................................................... 1     0   S01
    .................................ugaaauuuuucgcugcgguggu......................................................... 1     0   S01
    .................................ugaaauuuuucgcugcggugguu........................................................ 2     0   S01
    ......................................................................ucaccgggugaaaaguuugcu..................... 2     0   S01
    ......................................................................ucaccgggugaaaaguuugcuu.................... 199   0   S01
    ......................................................................ucaccgggugaaaaguuugcuuc................... 2     0   S01
    ..........................................................................cgggugaaaaguuugcuu.................... 2     0   S01

    .................................ugaaauuuuucgcugcgg............................................................. 2     0   S06
    .................................ugaaauuuuucgcugcggugg.......................................................... 2     0   S06
    .................................ugaaauuuuucgcugcgguggu......................................................... 8     0   S06
    .................................ugaaauuuuucgcugcggugguu........................................................ 57    0   S06
    .................................ugaaauuuuucgcugcggugguuu....................................................... 2     0   S06
    ...................................aaauuuuucgcugcggugguu........................................................ 2     0   S06
    ....................................aauuuuucgcugcggugguu........................................................ 3     0   S06
    ......................................................................ucaccgggugaaaaguuugc...................... 1     0   S06
    ......................................................................ucaccgggugaaaaguuugcu..................... 70    0   S06
    ......................................................................ucaccgggugaaaaguuugcuu.................... 2229  0   S06
    ......................................................................ucaccgggugaaaaguuugcuuc................... 54    0   S06
    ......................................................................ucaccgggugaaaaguuugcuucu.................. 1     0   S06
    .......................................................................caccgggugaaaaguuugcuu.................... 1     0   S06
    .........................................................................ccgggugaaaaguuugcuu.................... 2     0   S06
    ..........................................................................cgggugaaaaguuugcuu.................... 6     0   S06

    ................................augaaauuuuucgcugcggugguu........................................................ 1     0   S07
    .................................ugaaauuuuucgcugcgg............................................................. 1     0   S07
    .................................ugaaauuuuucgcugcgguggu......................................................... 10    0   S07
    .................................ugaaauuuuucgcugcggugguu........................................................ 134   0   S07
    .................................ugaaauuuuucgcugcggugguuu....................................................... 2     0   S07
    ....................................aauuuuucgcugcggugguu........................................................ 2     0   S07
    .....................................auuuuucgcugcggugguu........................................................ 3     0   S07
    ......................................uuuuucgcugcggugguu........................................................ 1     0   S07
    ......................................................................ucaccgggugaaaaguuugc...................... 1     0   S07
```

Star Mature

auccaaaaugcuggguuuaauuuucuuacaguaugaaauuuuucgcugcggugguuuguauauuugaaaaucaccgggugaaaaguuugcuucuguuuuaaaauuuuagcug			
....ucaccgggugaaaaguuugcu.....................	98	0	S07
....ucaccgggugaaaaguuugcuu....................	3205	0	S07
....ucaccgggugaaaaguuugcuuc...................	95	0	S07
....ucaccgggugaaaaguuugcuucu..................	1	0	S07
....accgggugaaaaguuugcuu....................	1	0	S07
....ccgggugaaaaguuugcuu....................	4	0	S07
....ccgggugaaaaguuugcuuc...................	1	0	S07
....cgggugaaaaguuugcuu....................	10	0	S07

.................................ugaaauuuuucgcugcgg...	3	0	S05
.................................ugaaauuuuucgcugcggug...	1	0	S05
.................................ugaaauuuuucgcugcggugg..	1	0	S05
.................................ugaaauuuuucgcugcgguggu...	5	0	S05
.................................ugaaauuuuucgcugcggugguu..	127	0	S05
.................................ugaaauuuuucgcugcggugguuu...	4	0	S05
.................................ugaaauuuuucgcugcggugguuuguau...	1	0	S05
...................................aaauuuuucgcugcggugguu..	1	0	S05
....................................aauuuuucgcugcggugguu..	1	0	S05
......................................uuuuucgcugcggugguu..	2	0	S05
....ucaccgggugaaaaguuugc......................	2	0	S05
....ucaccgggugaaaaguuugcu.....................	50	0	S05
....ucaccgggugaaaaguuugcuu....................	2578	0	S05
....ucaccgggugaaaaguuugcuuc...................	90	0	S05
....ucaccgggugaaaaguuugcuucu..................	2	0	S05
....ccgggugaaaaguuugcuu....................	4	0	S05
....cgggugaaaaguuugcuu....................	7	0	S05

.................................ugaaauuuuucgcugcgg...	1	0	S10
.................................ugaaauuuuucgcugcgguggu...	1	0	S10
.................................ugaaauuuuucgcugcggugguu..	41	0	S10
.................................ugaaauuuuucgcugcggugguuu...	3	0	S10
.................................ugaaauuuuucgcugcggugguuug..	1	0	S10
....ucaccgggugaaaaguuugc......................	1	0	S10
....ucaccgggugaaaaguuugcu.....................	90	0	S10
....ucaccgggugaaaaguuugcuu....................	1848	0	S10
....ucaccgggugaaaaguuugcuuc...................	26	0	S10
....ucaccgggugaaaaguuugcuucu..................	1	0	S10
....cgggugaaaaguuugcuu....................	1	0	S10

.................................ugaaauuuuucgcugcgguggu...	1	0	S02
.................................ugaaauuuuucgcugcggugguu..	10	0	S02
....ucaccgggugaaaaguuugc......................	2	0	S02
....ucaccgggugaaaaguuugcu.....................	9	0	S02
....ucaccgggugaaaaguuugcuu....................	248	0	S02
....ucaccgggugaaaaguuugcuuc...................	9	0	S02

.................................ugaaauuuuucgcugcgg...	3	0	S04
.................................ugaaauuuuucgcugcggug...	1	0	S04
.................................ugaaauuuuucgcugcgguggu...	9	0	S04
.................................ugaaauuuuucgcugcggugguu..	198	0	S04
.................................ugaaauuuuucgcugcggugguuu...	2	0	S04
..................................gaaauuuuucgcugcggugguu..	2	0	S04
....................................aauuuuucgcugcggugguu..	1	0	S04
....uguauauuugaaaaucaccgggugaaaaguuugcuuc...................	1	0	S04
....ucaccgggugaaaaguuugc......................	1	0	S04
....ucaccgggugaaaaguuugcu.....................	60	0	S04
....ucaccgggugaaaaguuugcuu....................	2464	0	S04
....ucaccgggugaaaaguuugcuuc...................	117	0	S04
....ucaccgggugaaaaguuugcuucu..................	2	0	S04
....ccgggugaaaaguuugcuu....................	2	0	S04
....cgggugaaaaguuugcuu....................	14	0	S04
....cgggugaaaaguuugcuuc...................	1	0	S04
....uuugcuucuguuuuaaaauuuu.....	1	0	S04

.................................ugaaauuuuucgcugcgg...	2	0	S03
.................................ugaaauuuuucgcugcggugg..	2	0	S03
.................................ugaaauuuuucgcugcgguggu...	13	0	S03
.................................ugaaauuuuucgcugcggugguu..	154	0	S03
..................................gaaauuuuucgcugcggugguu..	1	0	S03
....................................aauuuuucgcugcgguggu...	1	0	S03

Star Mature

```
auccaaaaugcuggguuuaauuuucuuacaguaugaaauuuuucgcugcgguggguuuguauauuugaaaaucaccgggugaaaaguuugcuucuguuuuaaaauuuuagcug
.......................................uuuucgcugcgguggguu........................................................  1     0   S03
.....................................................................ucaccgggugaaaaguuugc.......................  2     0   S03
.....................................................................ucaccgggugaaaaguuugcu......................  57    0   S03
.....................................................................ucaccgggugaaaaguuugcuu.....................  2231  0   S03
.....................................................................ucaccgggugaaaaguuugcuuc....................  84    0   S03
.....................................................................ucaccgggugaaaaguuugcuucu...................  1     0   S03
.......................................................................accgggugaaaaguuugcuu.....................  1     0   S03
........................................................................ccgggugaaaaguuugcuu.....................  3     0   S03
.........................................................................cgggugaaaaguuugcuu.....................  4     0   S03

.................................ugaaauuuuucgcugcgg.............................................................  1     0   S09
.................................ugaaauuuuucgcugcgguggu.........................................................  6     0   S09
.................................ugaaauuuuucgcugcgguggguu........................................................  39    0   S09
.................................ugaaauuuuucgcugcgguggguuu.......................................................  3     0   S09
.................................ugaaauuuuucgcugcgguggguuuguauau.................................................  1     0   S09
.....................................................................ucaccgggugaaaaguuugc.......................  1     0   S09
.....................................................................ucaccgggugaaaaguuugcu......................  108   0   S09
.....................................................................ucaccgggugaaaaguuugcuu.....................  1663  0   S09
.....................................................................ucaccgggugaaaaguuugcuuc....................  20    0   S09
........................................................................ccgggugaaaaguuugcuu.....................  2     0   S09
.........................................................................cgggugaaaaguuugcuu.....................  2     0   S09

.................................ugaaauuuuucgcugcggugg..........................................................  1     0   S08
.................................ugaaauuuuucgcugcgguggu.........................................................  4     0   S08
.................................ugaaauuuuucgcugcgguggguu........................................................  70    0   S08
.................................ugaaauuuuucgcugcgguggguuuguau...................................................  1     0   S08
...................................aaauuuuucgcugcgguggguu........................................................  1     0   S08
....................................aauuuuucgcugcgguggguu........................................................  3     0   S08
.....................................................................ucaccgggugaaaaguuugc.......................  2     0   S08
.....................................................................ucaccgggugaaaaguuugcu......................  115   0   S08
.....................................................................ucaccgggugaaaaguuugcuu.....................  3570  0   S08
.....................................................................ucaccgggugaaaaguuugcuuc....................  74    0   S08
.....................................................................ucaccgggugaaaaguuugcuucu...................  3     0   S08
......................................................................caccgggugaaaaguuugcu......................  1     0   S08
......................................................................caccgggugaaaaguuugcuu.....................  4     0   S08
........................................................................ccgggugaaaaguuugcuu.....................  12    0   S08
.........................................................................cgggugaaaaguuugcuu.....................  30    0   S08
```

Provisional ID	:	Scaffold248_6748
Score total	:	9359
Score for star read(s)	:	3.9
Score for read counts	:	9353.4
Score for mfe	:	0.1
Score for randfold	:	1.6
Score for cons. seed	:	
Total read count	:	18358
Mature read count	:	16938
Loop read count	:	0
Star read count	:	1420

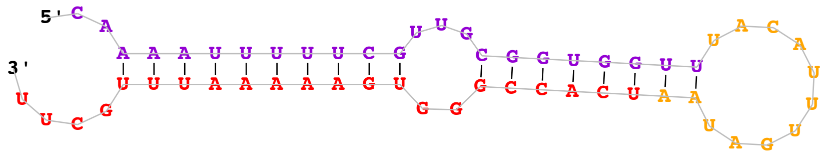

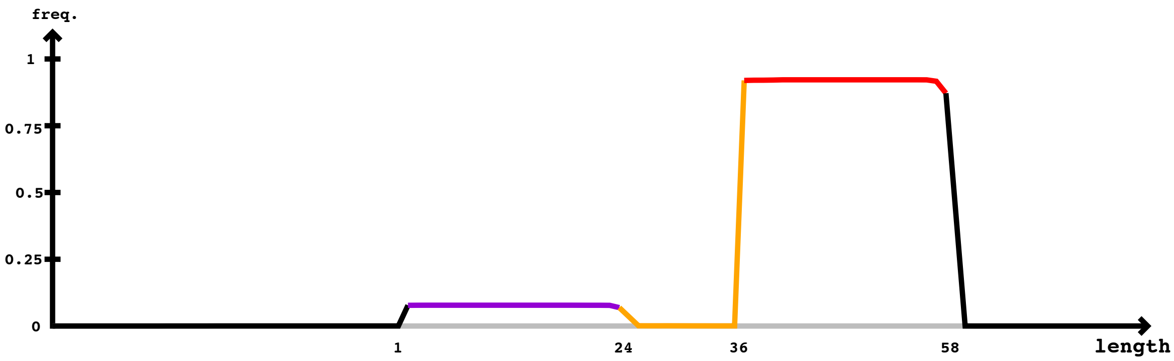

Star **Mature**

```
5'- cgcaaauaaaauguucuguuuaauuuuuuucagaacaaaauuuuucguugcggugguuuacauuugauaaucaccgggugaaaaauuugcuuuugagaagaaauaaaauccgc  -3'   obs
    cgcaaauaaaauguucuguuuaauuuuuuucagaacaaaauuuuucguugcggugguuuacauuugauaaucaccgggugaaaaauuugcuuuugagaagaaauaaaauccgc         exp
    ................((((.(((((((((((..(((((((((...((((((((..........))))))))..)))))))))..)))))))))))).)))))....    reads   mm   sample
    ...................................caaaauuuuucguugcgguggu........................................................    6       0    S08
    ...................................caaaauuuuucguugcggugguu.......................................................    72      0    S08
    ......................................................................ucaccgggugaaaaauuu.........................    1       0    S08
    ......................................................................ucaccgggugaaaaauuugc.......................    20      0    S08
    ......................................................................ucaccgggugaaaaauuugcu......................    122     0    S08
    ......................................................................ucaccgggugaaaaauuugcuu.....................    2429    0    S08
    ......................................................................ucaccgggugaaaaauuugcuuu....................    14      0    S08
    .......................................................................caccgggugaaaaauuugcu......................    1       0    S08
    .........................................................................ccgggugaaaaauuugcuu.....................    3       0    S08
    ..........................................................................cgggugaaaaauuugcuu.....................    7       0    S08

    ...................................caaaauuuuucguugcggugg.........................................................    2       0    S03
    ...................................caaaauuuuucguugcgguggu........................................................    35      0    S03
    ...................................caaaauuuuucguugcggugguu.......................................................    231     0    S03
    ...................................caaaauuuuucguugcggugguuu......................................................    7       0    S03
    ...................................caaaauuuuucguugcggugguuuacauu.................................................    1       0    S03
    ...................................caaaauuuuucguugcggugguuuacauuu................................................    1       0    S03
    ...................................caaaauuuuucguugcggugguuuacauuugaua............................................    1       0    S03
    ...................................caaaauuuuucguugcggugguuuacauuugauaau..........................................    2       0    S03
    .....................................aaauuuuucguugcggugguu.......................................................    1       0    S03
    ......................................................................ucaccgggugaaaaauuugc.......................    7       0    S03
    ......................................................................ucaccgggugaaaaauuugcu......................    80      0    S03
    ......................................................................ucaccgggugaaaaauuugcuu.....................    2052    0    S03
    ......................................................................ucaccgggugaaaaauuugcuuu....................    11      0    S03
    ......................................................................ucaccgggugaaaaauuugcuuuu...................    1       0    S03
    .......................................................................caccgggugaaaaauuugcuu.....................    5       0    S03
    .........................................................................ccgggugaaaaauuugcuu.....................    1       0    S03
    ..........................................................................cgggugaaaaauuugcuu.....................    2       0    S03

    ...................................caaaauuuuucguugcgg............................................................    1       0    S09
    ...................................caaaauuuuucguugcgguggu........................................................    12      0    S09
    ...................................caaaauuuuucguugcggugguu.......................................................    33      0    S09
    ......................................................................ucaccgggugaaaaauuugc.......................    6       0    S09
```

Star Mature

```
cgcaaauaaaauguucuguuuaauuuuuuucagaacaaaauuuuucguugcggugguuuacauuugauaaucaccgggugaaaaauuugcuuuugagaagaaauaaauccgc
......................................................................ucaccgggugaaaaauuugcu.....................  49    0  S09
......................................................................ucaccgggugaaaaauuugcuu....................  671   0  S09
......................................................................ucaccgggugaaaaauuugcuuu...................  7     0  S09
.......................................................................caccgggugaaaaauuugcuu....................  1     0  S09

...............cuguuuaauuuuuuucagaa.............................................................................  1     0  S04
...................................caaaauuuuucguugcgguggu.......................................................  31    0  S04
...................................caaaauuuuucguugcggugguu......................................................  257   0  S04
...................................caaaauuuuucguugcggugguuu.....................................................  11    0  S04
...................................caaaauuuuucguugcggugguuuac...................................................  1     0  S04
...................................caaaauuuuucguugcggugguuuacauuugauaa..........................................  2     0  S04
....................................aaaauuuuucguugcggugguu......................................................  3     0  S04
......................................................................ucaccgggugaaaaauuugc......................  6     0  S04
......................................................................ucaccgggugaaaaauuugcu.....................  77    0  S04
......................................................................ucaccgggugaaaaauuugcuu....................  2137  0  S04
......................................................................ucaccgggugaaaaauuugcuuu...................  18    0  S04
.......................................................................caccgggugaaaaauuugc......................  1     0  S04
.......................................................................caccgggugaaaaauuugcuu....................  1     0  S04
.......................................................................caccgggugaaaaauuugcuuu...................  1     0  S04
........................................................................accgggugaaaaauuugcuu....................  1     0  S04
.........................................................................ccgggugaaaaauuugcuu....................  1     0  S04
..........................................................................cgggugaaaaauuugcuu....................  2     0  S04

...............cuguuuaauuuuuuucagaa.............................................................................  1     0  S02
...................................caaaauuuuucguugcgguggu.......................................................  6     0  S02
...................................caaaauuuuucguugcggugguu......................................................  31    0  S02
...................................caaaauuuuucguugcggugguuu.....................................................  5     0  S02
......................................................................ucaccgggugaaaaauuugc......................  1     0  S02
......................................................................ucaccgggugaaaaauuugcu.....................  16    0  S02
......................................................................ucaccgggugaaaaauuugcuu....................  419   0  S02

...................................caaaauuuuucguugcggugg........................................................  1     0  S10
...................................caaaauuuuucguugcgguggu.......................................................  11    0  S10
...................................caaaauuuuucguugcggugguu......................................................  41    0  S10
...................................caaaauuuuucguugcggugguuua....................................................  1     0  S10
......................................................................ucaccgggugaaaaauuug.......................  1     0  S10
......................................................................ucaccgggugaaaaauuugc......................  9     0  S10
......................................................................ucaccgggugaaaaauuugcu.....................  67    0  S10
......................................................................ucaccgggugaaaaauuugcuu....................  1114  0  S10
......................................................................ucaccgggugaaaaauuugcuuu...................  12    0  S10
..........................................................................cgggugaaaaauuugcuu....................  2     0  S10

...................................caaaauuuuucguugcggugg........................................................  1     0  S05
...................................caaaauuuuucguugcgguggu.......................................................  25    0  S05
...................................caaaauuuuucguugcggugguu......................................................  170   0  S05
...................................caaaauuuuucguugcggugguuu.....................................................  5     0  S05
....................................aaaauuuuucguugcggugguu......................................................  1     0  S05
..............................................................uuugauaaucaccgggugaaaaauuugcuu....................  1     0  S05
......................................................................ucaccgggugaaaaauuugc......................  10    0  S05
......................................................................ucaccgggugaaaaauuugcu.....................  117   0  S05
......................................................................ucaccgggugaaaaauuugcuu....................  2277  0  S05
......................................................................ucaccgggugaaaaauuugcuuu...................  9     0  S05
.......................................................................caccgggugaaaaauuugcu.....................  1     0  S05
.......................................................................caccgggugaaaaauuugcuu....................  2     0  S05
.........................................................................ccgggugaaaaauuugcu.....................  2     0  S05
.........................................................................ccgggugaaaaauuugcuu....................  1     0  S05
..........................................................................cgggugaaaaauuugcuu....................  1     0  S05

...................................caaaauuuuucguugcggug.........................................................  1     0  S07
...................................caaaauuuuucguugcggugg........................................................  1     0  S07
...................................caaaauuuuucguugcgguggu.......................................................  17    0  S07
...................................caaaauuuuucguugcggugguu......................................................  193   0  S07
...................................caaaauuuuucguugcggugguuu.....................................................  6     0  S07
...................................caaaauuuuucguugcggugguuuacau.................................................  1     0  S07
...................................caaaauuuuucguugcggugguuuacauuuga.............................................  1     0  S07
....................................aaaauuuuucguugcggugguu......................................................  2     0  S07
......................................................................ucaccgggugaaaaauuug.......................  1     0  S07
......................................................................ucaccgggugaaaaauuugc......................  13    0  S07
......................................................................ucaccgggugaaaaauuugcu.....................  141   0  S07
......................................................................ucaccgggugaaaaauuugcuu....................  2379  0  S07
```

Star Mature

cgcaaauaaaauguucuguuuaauuuuuuucagaacaaaauuuuucguugcgguggguuuacauuugauaaucaccgggugaaaaauuugcuuuugagaagaaauaaaauccgc			
....ucaccgggugaaaaauuugcuuu....................	18	0	S07
....ucaccgggugaaaaauuugcuuuu...................	1	0	S07
....caccgggugaaaaauuugcuu.....................	2	0	S07
....ccgggugaaaaauuugcuu.....................	1	0	S07
....cgggugaaaaauuugcuu.....................	1	0	S07
...................................caaaauuuuucguugcgg..	1	0	S01
...................................caaaauuuuucguugcggu...	1	0	S01
...................................caaaauuuuucguugcgguggu..	3	0	S01
...................................caaaauuuuucguugcggugguu...	25	0	S01
....ucaccgggugaaaaauuugc.......................	3	0	S01
....ucaccgggugaaaaauuugcu......................	19	0	S01
....ucaccgggugaaaaauuugcuu.....................	433	0	S01
..........auguucuguuuaauuuuuuucagaa..	1	0	S06
...........uguucuguuuaauuuuuuucagaa..	1	0	S06
...................................caaaauuuuucguugcgguggu..	10	0	S06
...................................caaaauuuuucguugcggugguu...	137	0	S06
...................................caaaauuuuucguugcggugguuu..	21	0	S06
...................................caaaauuuuucguugcggugguuuacauu...	1	0	S06
...................................caaaauuuuucguugcggugguuuacauuu..	1	0	S06
...................................caaaauuuuucguugcggugguuuacauuugauaa...	1	0	S06
....uuugauaaucaccgggugaaaaauuugcuu.....................	1	0	S06
....ucaccgggugaaaaauuugc.......................	20	0	S06
....ucaccgggugaaaaauuugcu......................	132	0	S06
....ucaccgggugaaaaauuugcuu.....................	1962	0	S06
....ucaccgggugaaaaauuugcuuu....................	11	0	S06
....caccgggugaaaaauuugcuu.....................	3	0	S06
....cgggugaaaaauuugcuu.....................	2	0	S06

Provisional ID	:	Scaffold248_6758
Score total	:	6202.4
Score for star read(s)	:	3.9
Score for read counts	:	6195.5
Score for mfe	:	1.4
Score for randfold	:	1.6
Score for cons. seed	:	
Total read count	:	12164
Mature read count	:	11636
Loop read count	:	0
Star read count	:	528

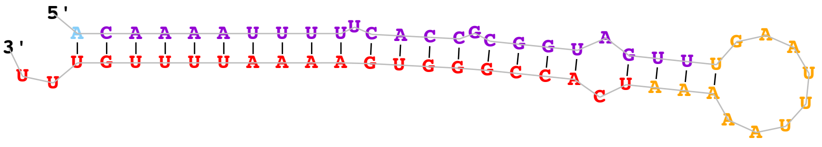

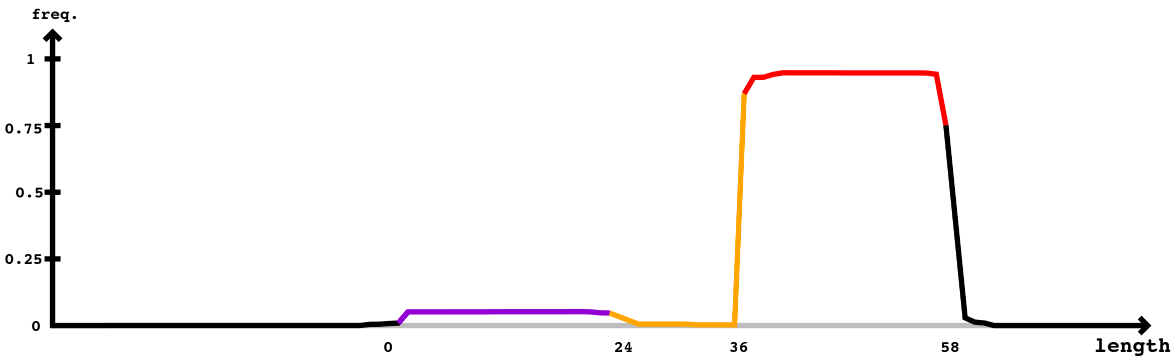

Star Mature

```
5'- auugugacuaaaaugcuguuucguuuuuuacagaacaaaauuuuucaccgcgguaguuugaauuuaaaaaucaccgggugaaaauuuuguuucuguaaagaaacaaaucuac  -3'   obs
    auugugacuaaaaugcuguuucguuuuuuacagaacaaaauuuuucaccgcgguaguuugaauuuaaaaaucaccgggugaaaauuuuguuucuguaaagaaacaaaucuac         exp
    ................((((((....((((((((((((((((((.((((.((((.((((........)))).))))))))))))))))).)))))))))))))))....... reads  mm  sample
    ...............................agaacaaaauuuuucaccgcggu.......................................................... 1  0  S02
    ...................................caaaauuuuucaccgcgguagu....................................................... 3  0  S02
    ...................................caaaauuuuucaccgcgguaguu...................................................... 13  0  S02
    ...................................caaaauuuuucaccgcgguaguuugaauuu............................................... 1  0  S02
    ......................................................................ucaccgggugaaaauuuugu...................... 1  0  S02
    ......................................................................ucaccgggugaaaauuuuguu..................... 15  0  S02
    ......................................................................ucaccgggugaaaauuuuguuu.................... 144  0  S02
    ......................................................................ucaccgggugaaaauuuuguuuc................... 11  0  S02
    ......................................................................ucaccgggugaaaauuuuguuucu.................. 1  0  S02
    .......................................................................caccgggugaaaauuuuguuu.................... 7  0  S02
    .......................................................................caccgggugaaaauuuuguuuc................... 9  0  S02
    .......................................................................caccgggugaaaauuuuguuucug................. 1  0  S02

    ...............................agaacaaaauuuuucaccgcgg........................................................... 1  0  S04
    ...............................agaacaaaauuuuucaccgcggua......................................................... 3  0  S04
    .................................aacaaaauuuuucaccgcgguagu....................................................... 5  0  S04
    ..................................acaaaauuuuucaccgcgguagu....................................................... 1  0  S04
    ..................................acaaaauuuuucaccgcgguaguu...................................................... 3  0  S04
    ..................................acaaaauuuuucaccgcgguaguuugaauuu............................................... 1  0  S04
    ...................................caaaauuuuucaccgcgguagu....................................................... 13  0  S04
    ...................................caaaauuuuucaccgcgguaguu...................................................... 129  0  S04
    ...................................caaaauuuuucaccgcgguaguuu..................................................... 2  0  S04
    ...................................caaaauuuuucaccgcgguaguuugaau................................................. 1  0  S04
    ...................................caaaauuuuucaccgcgguaguuugaauuu............................................... 4  0  S04
    ...................................caaaauuuuucaccgcgguaguuugaauuuaaaaa.......................................... 8  0  S04
    ...................................caaaauuuuucaccgcgguaguuugaauuuaaaaau......................................... 1  0  S04
    .....................................................uaguuugaauuuaaaaaucaccgggug................................ 2  0  S04
    ...............................................................uuaaaaaucaccgggugaaaauuuuguuu.................... 1  0  S04
    ......................................................................ucaccgggugaaaauuuug....................... 3  0  S04
    ......................................................................ucaccgggugaaaauuuugu...................... 3  0  S04
    ......................................................................ucaccgggugaaaauuuuguu..................... 256  0  S04
    ......................................................................ucaccgggugaaaauuuuguuu.................... 1139  0  S04
    ......................................................................ucaccgggugaaaauuuuguuuc................... 114  0  S04
```

Star   Mature

```
auugugacuaaaaugcuguuucguuuuuuacagaacaaaauuuuucaccgcgguaguuugaauuuaaaaaucaccgggugaaaauuuuguuucuguaaagaaacaaaucuac
......................................................................ucaccgggugaaaauuuuguuucu..................  5    0  S04
.......................................................................caccgggugaaaauuuuguuu....................  72   0  S04
.......................................................................caccgggugaaaauuuuguuuc...................  40   0  S04
.......................................................................caccgggugaaaauuuuguuucu..................  27   0  S04
.......................................................................caccgggugaaaauuuuguuucug.................  8    0  S04
.......................................................................caccgggugaaaauuuuguuucugu................  2    0  S04
........................................................................accgggugaaaauuuuguuu....................  1    0  S04
.........................................................................ccgggugaaaauuuuguuu....................  3    0  S04
.........................................................................ccgggugaaaauuuuguuucu..................  3    0  S04
.........................................................................ccgggugaaaauuuuguuucug.................  2    0  S04
.........................................................................ccgggugaaaauuuuguuucugu................  22   0  S04
..........................................................................cgggugaaaauuuuguuu....................  2    0  S04
..........................................................................cgggugaaaauuuuguuucugu................  7    0  S04

....ugacuaaaaugcuguuucguuuuuuacagaa.............................................................................  2    0  S08
...............................agaacaaaauuuuucaccgcggu..........................................................  1    0  S08
...............................agaacaaaauuuuucaccgcggua.........................................................  6    0  S08
.................................aacaaaauuuuucaccgcgguagu.......................................................  4    0  S08
.................................aacaaaauuuuucaccgcgguaguuu.....................................................  1    0  S08
..................................acaaaauuuuucaccgcgguaguu......................................................  2    0  S08
...................................caaaauuuuucaccgcggua.........................................................  2    0  S08
...................................caaaauuuuucaccgcgguag........................................................  1    0  S08
...................................caaaauuuuucaccgcgguagu.......................................................  5    0  S08
...................................caaaauuuuucaccgcgguaguu......................................................  21   0  S08
...................................caaaauuuuucaccgcgguaguuugaauuu...............................................  4    0  S08
............................................ucaccgcgguaguuugaauuuaaaaaucac......................................  1    0  S08
...............................................................uuaaaaaucaccgggugaaaauuuuguuu....................  1    0  S08
......................................................................ucaccgggugaaaauuuugu......................  9    0  S08
......................................................................ucaccgggugaaaauuuuguu.....................  398  0  S08
......................................................................ucaccgggugaaaauuuuguuu....................  950  0  S08
......................................................................ucaccgggugaaaauuuuguuuc...................  80   0  S08
......................................................................ucaccgggugaaaauuuuguuucu..................  6    0  S08
......................................................................ucaccgggugaaaauuuuguuucug.................  1    0  S08
.......................................................................caccgggugaaaauuuugu......................  1    0  S08
.......................................................................caccgggugaaaauuuuguu.....................  3    0  S08
.......................................................................caccgggugaaaauuuuguuu....................  27   0  S08
.......................................................................caccgggugaaaauuuuguuuc...................  21   0  S08
.......................................................................caccgggugaaaauuuuguuucu..................  20   0  S08
........................................................................accgggugaaaauuuuguu.....................  1    0  S08
.........................................................................ccgggugaaaauuuuguu.....................  2    0  S08
.........................................................................ccgggugaaaauuuuguuu....................  9    0  S08
.........................................................................ccgggugaaaauuuuguuucu..................  1    0  S08
.........................................................................ccgggugaaaauuuuguuucugu................  3    0  S08
..........................................................................cgggugaaaauuuuguuu....................  22   0  S08
..........................................................................cgggugaaaauuuuguuucugu................  2    0  S08

...............................agaacaaaauuuuucaccgcggua.........................................................  5    0  S09
...................................caaaauuuuucaccgcgguaguu......................................................  2    0  S09
......................................................................ucaccgggugaaaauuuugu......................  3    0  S09
......................................................................ucaccgggugaaaauuuuguu.....................  223  0  S09
......................................................................ucaccgggugaaaauuuuguuu....................  604  0  S09
......................................................................ucaccgggugaaaauuuuguuuc...................  6    0  S09
......................................................................ucaccgggugaaaauuuuguuucu..................  2    0  S09
.......................................................................caccgggugaaaauuuuguuu....................  21   0  S09
.......................................................................caccgggugaaaauuuuguuuc...................  8    0  S09
.......................................................................caccgggugaaaauuuuguuucu..................  7    0  S09
.........................................................................ccgggugaaaauuuuguuu....................  2    0  S09
..........................................................................cgggugaaaauuuuguuu....................  1    0  S09
..........................................................................cgggugaaaauuuuguuucugu................  2    0  S09

...............................agaacaaaauuuuucaccgcggu..........................................................  2    0  S03
...............................agaacaaaauuuuucaccgcggua.........................................................  7    0  S03
................................gaacaaaauuuuucaccgcggua.........................................................  1    0  S03
.................................aacaaaauuuuucaccgcgguagu.......................................................  9    0  S03
..................................acaaaauuuuucaccgcgguagu.......................................................  2    0  S03
...................................caaaauuuuucaccgcgguagu.......................................................  20   0  S03
...................................caaaauuuuucaccgcgguaguu......................................................  77   0  S03
...................................caaaauuuuucaccgcgguaguuu.....................................................  2    0  S03
...................................caaaauuuuucaccgcgguaguuugaauu................................................  2    0  S03
...................................caaaauuuuucaccgcgguaguuugaauuu...............................................  7    0  S03
```

```
                                   Star                                Mature
auugugacuaaaaugcuguuucguuuuuuacagaacaaaauuuuucaccgcgguaguuugaauuuaaaaaucaccgggugaaaauuuuguuucuguaaagaaacaaaucuac
...................................caaaauuuuucaccgcgguaguuugaauuuaaa............................................    1      0     S03
...................................caaaauuuuucaccgcgguaguuugaauuuaaaa...........................................    1      0     S03
...................................caaaauuuuucaccgcgguaguuugaauuuaaaaa..........................................    6      0     S03
............................................ucaccgcgguaguuugaauuuaaaaaucacc.....................................    1      0     S03
.....................................................................aucaccgggugaaaauuuuguuu....................    1      0     S03
......................................................................ucaccgggugaaaauuuugu......................    4      0     S03
......................................................................ucaccgggugaaaauuuuguu.....................    255    0     S03
......................................................................ucaccgggugaaaauuuuguuu....................    1225   0     S03
......................................................................ucaccgggugaaaauuuuguuuc...................    122    0     S03
......................................................................ucaccgggugaaaauuuuguuucu..................    4      0     S03
......................................................................ucaccgggugaaaauuuuguuucug.................    3      0     S03
.......................................................................caccgggugaaaauuuuguu.....................    1      0     S03
.......................................................................caccgggugaaaauuuuguuu....................    72     0     S03
.......................................................................caccgggugaaaauuuuguuuc...................    33     0     S03
.......................................................................caccgggugaaaauuuuguuucu..................    25     0     S03
.......................................................................caccgggugaaaauuuuguuucug.................    7      0     S03
........................................................................accgggugaaaauuuuguuu....................    1      0     S03
.........................................................................ccgggugaaaauuuuguuu....................    3      0     S03
.........................................................................ccgggugaaaauuuuguuuc...................    1      0     S03
.........................................................................ccgggugaaaauuuuguuucug.................    5      0     S03
.........................................................................ccgggugaaaauuuuguuucugu................    23     0     S03
..........................................................................cgggugaaaauuuuguuu....................    3      0     S03
..........................................................................cgggugaaaauuuuguuucugu................    9      0     S03
...........................................................................gggugaaaauuuuguuucug.................    1      0     S03

...............................agaacaaaauuuuucaccgcggu..........................................................    1      0     S07
...............................agaacaaaauuuuucaccgcggua.........................................................    8      0     S07
................................gaacaaaauuuuucaccgcggu..........................................................    1      0     S07
................................gaacaaaauuuuucaccgcgguag........................................................    2      0     S07
................................gaacaaaauuuuucaccgcgguaguu......................................................    1      0     S07
.................................aacaaaauuuuucaccgcgguagu.......................................................    8      0     S07
..................................acaaaauuuuucaccgcgguagu.......................................................    1      0     S07
..................................acaaaauuuuucaccgcgguaguu......................................................    2      0     S07
...................................caaaauuuuucaccgcgguag........................................................    2      0     S07
...................................caaaauuuuucaccgcgguagu.......................................................    8      0     S07
...................................caaaauuuuucaccgcgguaguu......................................................    47     0     S07
...................................caaaauuuuucaccgcgguaguuu.....................................................    1      0     S07
...................................caaaauuuuucaccgcgguaguuugaauuu...............................................    6      0     S07
...................................caaaauuuuucaccgcgguaguuugaauuuaaaaa..........................................    2      0     S07
........................................uuuuucaccgcgguaguuugaauuuaaaaaucac......................................    1      0     S07
......................................................................ucaccgggugaaaauuuug.......................    1      0     S07
......................................................................ucaccgggugaaaauuuugu......................    12     0     S07
......................................................................ucaccgggugaaaauuuuguu.....................    353    0     S07
......................................................................ucaccgggugaaaauuuuguuu....................    1063   0     S07
......................................................................ucaccgggugaaaauuuuguuuc...................    70     0     S07
......................................................................ucaccgggugaaaauuuuguuucu..................    1      0     S07
.......................................................................caccgggugaaaauuuuguu.....................    2      0     S07
.......................................................................caccgggugaaaauuuuguuu....................    35     0     S07
.......................................................................caccgggugaaaauuuuguuuc...................    33     0     S07
.......................................................................caccgggugaaaauuuuguuucu..................    29     0     S07
.........................................................................ccgggugaaaauuuuguu.....................    1      0     S07
.........................................................................ccgggugaaaauuuuguuu....................    3      0     S07
.........................................................................ccgggugaaaauuuuguuucug.................    1      0     S07
.........................................................................ccgggugaaaauuuuguuucugu................    3      0     S07
..........................................................................cgggugaaaauuuuguuu....................    8      0     S07
..........................................................................cgggugaaaauuuuguuucu..................    1      0     S07
..........................................................................cgggugaaaauuuuguuucug.................    1      0     S07
..........................................................................cgggugaaaauuuuguuucugu................    5      0     S07
..........................................................................cgggugaaaauuuuguuucugua...............    1      0     S07

................uguuucguuuuuuacagaa.............................................................................    1      0     S06
...............................agaacaaaauuuuucaccgc.............................................................    1      0     S06
...............................agaacaaaauuuuucaccgcggua.........................................................    7      0     S06
..................................acaaaauuuuucaccgcgguag........................................................    1      0     S06
..................................acaaaauuuuucaccgcgguaguu......................................................    1      0     S06
..................................acaaaauuuuucaccgcgguaguuugaauuu...............................................    1      0     S06
...................................caaaauuuuucaccgcgguag........................................................    1      0     S06
...................................caaaauuuuucaccgcgguagu.......................................................    5      0     S06
...................................caaaauuuuucaccgcgguaguu......................................................    47     0     S06
...................................caaaauuuuucaccgcgguaguuugaauuu...............................................    1      0     S06
```

Star **Mature**

```
auugugacuaaaaugcuguuucguuuuuuacagaacaaaauuuuucaccgcgguaguuugaauuuaaaaaucaccgggugaaaauuuuguuucuguaaagaaacaaaucuac
............................................ucaccgcgguaguuugaauuuaaaaaucac......................................	2	0	S06
....................................................guaguuugaauuuaaaaaucaccggguga...............................	1	0	S06
......................................................................ucaccgggugaaaauuuug.......................	2	0	S06
......................................................................ucaccgggugaaaauuuugu......................	6	0	S06
......................................................................ucaccgggugaaaauuuuguu.....................	272	0	S06
......................................................................ucaccgggugaaaauuuuguuu....................	652	0	S06
......................................................................ucaccgggugaaaauuuuguuuc...................	112	0	S06
......................................................................ucaccgggugaaaauuuuguuucu..................	7	0	S06
.......................................................................caccgggugaaaauuuuguu.....................	2	0	S06
.......................................................................caccgggugaaaauuuuguuu....................	35	0	S06
.......................................................................caccgggugaaaauuuuguuuc...................	23	0	S06
.......................................................................caccgggugaaaauuuuguuucu..................	20	0	S06
.......................................................................caccgggugaaaauuuuguuucug.................	1	0	S06
.........................................................................ccgggugaaaauuuuguu.....................	2	0	S06
.........................................................................ccgggugaaaauuuuguuucu..................	1	0	S06
.........................................................................ccgggugaaaauuuuguuucug.................	1	0	S06
.........................................................................ccgggugaaaauuuuguuucugu................	12	0	S06
..........................................................................cgggugaaaauuuuguuu....................	1	0	S06
..........................................................................cgggugaaaauuuuguuucug.................	1	0	S06
..........................................................................cgggugaaaauuuuguuucugu................	1	0	S06

...................................caaaauuuuucaccgcgguaguu......................................................	2	0	S01
......................................................................ucaccgggugaaaauuuugu......................	2	0	S01
......................................................................ucaccgggugaaaauuuuguu.....................	16	0	S01
......................................................................ucaccgggugaaaauuuuguuu....................	141	0	S01
......................................................................ucaccgggugaaaauuuuguuuc...................	9	0	S01
.......................................................................caccgggugaaaauuuuguuu....................	6	0	S01
.......................................................................caccgggugaaaauuuuguuuc...................	2	0	S01
.......................................................................caccgggugaaaauuuuguuucu..................	4	0	S01
.........................................................................ccgggugaaaauuuuguu.....................	1	0	S01
.........................................................................ccgggugaaaauuuuguuucugu................	1	0	S01

...............................agaacaaaauuuuucaccgcggu..........................................................	3	0	S10
...............................agaacaaaauuuuucaccgcggua.........................................................	1	0	S10
..................................acaaaauuuuucaccgcgguaguu......................................................	1	0	S10
...................................caaaauuuuucaccgcgguaguu......................................................	6	0	S10
......................................................................ucaccgggugaaaauuuugu......................	3	0	S10
......................................................................ucaccgggugaaaauuuuguu.....................	284	0	S10
......................................................................ucaccgggugaaaauuuuguuu....................	744	0	S10
......................................................................ucaccgggugaaaauuuuguuuc...................	30	0	S10
......................................................................ucaccgggugaaaauuuuguuucu..................	1	0	S10
.......................................................................caccgggugaaaauuuuguu.....................	2	0	S10
.......................................................................caccgggugaaaauuuuguuu....................	35	0	S10
.......................................................................caccgggugaaaauuuuguuuc...................	7	0	S10
.......................................................................caccgggugaaaauuuuguuucu..................	11	0	S10
.........................................................................ccgggugaaaauuuuguu.....................	1	0	S10
.........................................................................ccgggugaaaauuuuguuucugu................	2	0	S10
..........................................................................cgggugaaaauuuuguuucugu................	1	0	S10

...............................agaacaaaauuuuucaccgcggua.........................................................	4	0	S05
................................gaacaaaauuuuucaccgcggu..........................................................	1	0	S05
................................gaacaaaauuuuucaccgcggua.........................................................	1	0	S05
................................gaacaaaauuuuucaccgcgguaguu......................................................	1	0	S05
.................................aacaaaauuuuucaccgcgguagu.......................................................	4	0	S05
..................................acaaaauuuuucaccgcgguagu.......................................................	3	0	S05
..................................acaaaauuuuucaccgcgguaguu......................................................	2	0	S05
...................................caaaauuuuucaccgcgguag........................................................	1	0	S05
...................................caaaauuuuucaccgcgguagu.......................................................	8	0	S05
...................................caaaauuuuucaccgcgguaguu......................................................	50	0	S05
...................................caaaauuuuucaccgcgguaguuu.....................................................	2	0	S05
...................................caaaauuuuucaccgcgguaguuugaauu................................................	1	0	S05
...................................caaaauuuuucaccgcgguaguuugaauuu...............................................	1	0	S05
...................................caaaauuuuucaccgcgguaguuugaauuuaaaaa..........................................	3	0	S05
........................................uuuucaccgcgguaguuugaauuuaaaaaucacc.....................................	1	0	S05
............................................ucaccgcgguaguuugaauuuaaaaauc........................................	1	0	S05
..............................................accgcgguaguuugaauuuaaa............................................	1	0	S05
....................................................guaguuugaauuuaaaaaucaccgggug................................	1	0	S05
....................................................guaguuugaauuuaaaaaucaccggguga...............................	1	0	S05
......................................................................ucaccgggugaaaauuuug.......................	1	0	S05
......................................................................ucaccgggugaaaauuuugu......................	8	0	S05
```

```
                                   Star                              Mature
auugugacuaaaaugcuguuucguuuuuuacagaacaaaauuuuucaccgcgguaguuugaauuuaaaaaucaccgggugaaaauuuuguuucuguaaagaaacaaaucuac
......................................................................ucaccgggugaaaauuuuguu.....................    263    0    S05
......................................................................ucaccgggugaaaauuuuguuu....................    907    0    S05
......................................................................ucaccgggugaaaauuuuguuuc...................    109    0    S05
......................................................................ucaccgggugaaaauuuuguuucu..................    7      0    S05
......................................................................ucaccgggugaaaauuuuguuucug.................    1      0    S05
.......................................................................caccgggugaaaauuuugu......................    1      0    S05
.......................................................................caccgggugaaaauuuuguuu....................    46     0    S05
.......................................................................caccgggugaaaauuuuguuuc...................    33     0    S05
.......................................................................caccgggugaaaauuuuguuucu..................    10     0    S05
.......................................................................caccgggugaaaauuuuguuucug.................    4      0    S05
........................................................................accgggugaaaauuuuguu.....................    1      0    S05
.........................................................................ccgggugaaaauuuuguuu....................    2      0    S05
.........................................................................ccgggugaaaauuuuguuucu..................    6      0    S05
.........................................................................ccgggugaaaauuuuguuucug.................    1      0    S05
.........................................................................ccgggugaaaauuuuguuucugu................    16     0    S05
..........................................................................cgggugaaaauuuuguuu....................    5      0    S05
..........................................................................cgggugaaaauuuuguuuc...................    1      0    S05
..........................................................................cgggugaaaauuuuguuucugu................    6      0    S05
```

Provisional ID	:	Scaffold2773_37223
Score total	:	163822.2
Score for star read(s)	:	3.9
Score for read counts	:	163815.1
Score for mfe	:	1.6
Score for randfold	:	1.6
Score for cons. seed	:	
Total read count	:	321328
Mature read count	:	313296
Loop read count	:	1
Star read count	:	8031

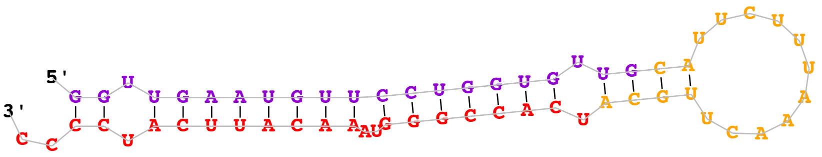

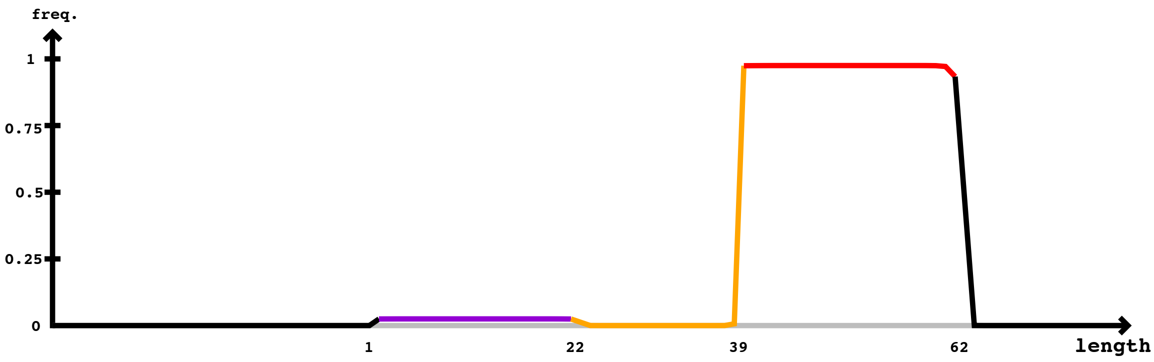

Star **Mature**

```
5'- ucauccugcugaaugauaaguuucuuuuauuggguugaauguuccugguguugcauucuuuaaacuugcaucaccggguaaacauucauccccauguugaaacgcauaac  -3'   obs
    ucauccugcugaaugauaaguuucuuuuauuggguugaauguuccugguguugcauucuuuaaacuugcaucaccggguaaacauucauccccauguugaaacgcauaac         exp
    ((((.(....).))))...(((((...(((.(((.(((((((((((((((.((((...........)))).)))))))..)))))))).))).)))..))))).......      reads   mm   sample
    ...............................ggguugaauguuccugguguug.........................................................      1       0    S03
    ................................gguugaauguuccuggugu...........................................................      3       0    S03
    ................................gguugaauguuccugguguu..........................................................      7       0    S03
    ................................gguugaauguuccugguguug.........................................................      367     0    S03
    ................................gguugaauguuccugguguugc........................................................      5       0    S03
    .................................guugaauguuccugguguug.........................................................      1       0    S03
    .....................................................................aucaccggguaaacauucaucc...................      1       0    S03
    .....................................................................aucaccggguaaacauucaucccc.................      17      0    S03
    ......................................................................ucaccggguaaacauucau.....................      1       0    S03
    ......................................................................ucaccggguaaacauucauc....................      3       0    S03
    ......................................................................ucaccggguaaacauucaucc...................      15      0    S03
    ......................................................................ucaccggguaaacauucauccc..................      103     0    S03
    ......................................................................ucaccggguaaacauucaucccc.................      598     0    S03
    ......................................................................ucaccggguaaacauucauccccа................      5       0    S03

    ................................gguugaauguuccuggug............................................................      1       0    S09
    ................................gguugaauguuccuggugu...........................................................      3       0    S09
    ................................gguugaauguuccugguguu..........................................................      18      0    S09
    ................................gguugaauguuccugguguug.........................................................      1571    0    S09
    ................................gguugaauguuccugguguugc........................................................      4       0    S09
    ................................gguugaauguuccugguguugcauu.....................................................      1       0    S09
    ................................gguugaauguuccugguguugcauuc....................................................      1       0    S09
    ................................gguugaauguuccugguguugcauucuuuaaacuugcau.......................................      1       0    S09
    .................................guugaauguuccugguguug.........................................................      6       0    S09
    .................................guugaauguuccugguguugc........................................................      1       0    S09
    .................................guugaauguuccugguguugca.......................................................      3       0    S09
    ..................................uugaauguuccugguguug.........................................................      9       0    S09
    ..................................uugaauguuccugguguugc........................................................      1       0    S09
    ..................................uugaauguuccugguguugca.......................................................      1       0    S09
    .....................................................cauucuuuaaacuugcaucacc...................................      1       0    S09
    .....................................................cauucuuuaaacuugcaucaccggguaaacauucauccc..................      2       0    S09
    ..................................................................ugcaucaccggguaaacauucauccc..................      4       0    S09
    ....................................................................caucaccggguaaacauucauccc..................      2       0    S09
```

Star Mature

ucauccugcugaaugauaaguuucuuuuauuggguugaauguuccugguguugcauucuuuaaacuugcaucaccggguaaacauucauccccauguugaaacgcauaac			
....caucaccggguaaacauucaucccc.................	1	0	S09
....aucaccggguaaacauucauc....................	1	0	S09
....aucaccggguaaacauucaucc...................	1	0	S09
....aucaccggguaaacauucauccc..................	7	0	S09
....aucaccggguaaacauucaucccc.................	597	0	S09
....aucaccggguaaacauucaucccca................	2	0	S09
....ucaccggguaaacauucau.....................	2	0	S09
....ucaccggguaaacauucauc....................	40	0	S09
....ucaccggguaaacauucaucc...................	381	0	S09
....ucaccggguaaacauucauccc..................	4698	0	S09
....ucaccggguaaacauucaucccc.................	104418	0	S09
....ucaccggguaaacauucaucccca................	659	0	S09
....ucaccggguaaacauucauccccau...............	23	0	S09
....ucaccggguaaacauucauccccaug..............	1	0	S09
....caccggguaaacauucauccc..................	2	0	S09
....caccggguaaacauucaucccc.................	20	0	S09
....caccggguaaacauucaucccca................	1	0	S09
....accggguaaacauucauccc..................	1	0	S09
....accggguaaacauucaucccc.................	17	0	S09
....ccggguaaacauucaucccc.................	5	0	S09
....cggguaaacauucaucccc.................	6	0	S09
....ggguaaacauucaucccc.................	9	0	S09
................................gguugaauguuccugguguu..	4	0	S08
................................gguugaauguuccugguguug...	339	0	S08
................................gguugaauguuccugguguugc..	1	0	S08
..................................uugaauguuccugguguug...	2	0	S08
...................................ugaauguuccugguguug...	1	0	S08
....acuugcaucaccggguaaacauucaucccc.................	1	0	S08
....caucaccggguaaacauucauccc..................	1	0	S08
....aucaccggguaaacauucauccc..................	2	0	S08
....aucaccggguaaacauucaucccc.................	93	0	S08
....aucaccggguaaacauucaucccca................	1	0	S08
....ucaccggguaaacauucauc....................	22	0	S08
....ucaccggguaaacauucaucc...................	88	0	S08
....ucaccggguaaacauucauccc..................	836	0	S08
....ucaccggguaaacauucaucccc.................	14056	0	S08
....ucaccggguaaacauucaucccca................	103	0	S08
....ucaccggguaaacauucauccccau...............	2	0	S08
....caccggguaaacauucaucccc.................	7	0	S08
....accggguaaacauucaucccc.................	5	0	S08
....ccggguaaacauucauccc..................	2	0	S08
....ccggguaaacauucaucccc.................	9	0	S08
....cggguaaacauucaucccc.................	1	0	S08
....ggguaaacauucaucccc.................	3	0	S08
................................gguugaauguuccugguguug...	51	0	S02
....aucaccggguaaacauucaucc...................	1	0	S02
....aucaccggguaaacauucaucccc.................	2	0	S02
....ucaccggguaaacauucaucc...................	7	0	S02
....ucaccggguaaacauucauccc..................	57	0	S02
....ucaccggguaaacauucaucccc.................	145	0	S02
....ucaccggguaaacauucaucccca................	4	0	S02
....ucaccggguaaacauucauccccau...............	2	0	S02
................................gguugaauguuccuggug..	1	0	S04
................................gguugaauguuccuggugu...	1	0	S04
................................gguugaauguuccugguguu..	5	0	S04
................................gguugaauguuccugguguug...	589	0	S04
................................gguugaauguuccugguguugc..	5	0	S04
.................................guugaauguuccugguguugc..	1	0	S04
..................................uugaauguuccugguguug...	1	0	S04
....aucaccggguaaacauucauccc..................	2	0	S04
....aucaccggguaaacauucaucccc.................	22	0	S04
....ucaccggguaaacauucau.....................	1	0	S04
....ucaccggguaaacauucauc....................	6	0	S04
....ucaccggguaaacauucaucc...................	17	0	S04
....ucaccggguaaacauucauccc..................	159	0	S04
....ucaccggguaaacauucaucccc.................	1108	0	S04
....ucaccggguaaacauucaucccca................	6	0	S04

Star   Mature

```
ucauccugcugaaugauaaguuucuuuuauugggguugaauguuccugguguugcauucuuuaaacuugcaucaccggguaaacauucauccccauguugaaacgcauaac
......................................................................ucaccggguaaacauucauccccau...............  1       0   S04
........................................................................accggguaaacauucaucccc.................  2       0   S04
.........................................................................ccggguaaacauucaucccc.................  1       0   S04
...........................................................................ggguaaacauucaucccc.................  1       0   S04

................................gguugaauguuccugguguu..........................................................  4       0   S05
................................gguugaauguuccugguguug.........................................................  418     0   S05
................................gguugaauguuccugguguugc........................................................  3       0   S05
.................................guugaauguuccugguguug.........................................................  1       0   S05
.................................guugaauguuccugguguugca.......................................................  1       0   S05
...................................ugaauguuccugguguug.........................................................  1       0   S05
.....................................................................aucaccggguaaacauucaucccc.................  16      0   S05
......................................................................ucaccggguaaacauucau.....................  1       0   S05
......................................................................ucaccggguaaacauucauc....................  4       0   S05
......................................................................ucaccggguaaacauucaucc...................  7       0   S05
......................................................................ucaccggguaaacauucauccc..................  126     0   S05
......................................................................ucaccggguaaacauucaucccc.................  968     0   S05
......................................................................ucaccggguaaacauucaucccca................  9       0   S05
.........................................................................ccggguaaacauucaucccc.................  1       0   S05

..............................uggguugaauguuccugguguu..........................................................  1       0   S10
...............................ggguugaauguuccugguguug.........................................................  3       0   S10
................................gguugaauguuccuggug............................................................  1       0   S10
................................gguugaauguuccuggugu...........................................................  3       0   S10
................................gguugaauguuccugguguu..........................................................  28      0   S10
................................gguugaauguuccugguguug.........................................................  1071    0   S10
................................gguugaauguuccugguguugc........................................................  2       0   S10
................................gguugaauguuccugguguugcauucuuuaaacuugca........................................  1       0   S10
................................gguugaauguuccugguguugcauucuuuaaacuugcau.......................................  1       0   S10
.................................guugaauguuccugguguug.........................................................  6       0   S10
.................................guugaauguuccugguguugca.......................................................  2       0   S10
..................................uugaauguuccugguguug.........................................................  19      0   S10
...................................ugaauguuccugguguug.........................................................  2       0   S10
.....................................................cauucuuuaaacuugcaucaccggguaaacauucaucccc.................  1       0   S10
................................................................cuugcaucaccggguaaacauuca......................  1       0   S10
.................................................................uugcaucaccggguaaacauucaucccc.................  1       0   S10
....................................................................caucaccggguaaacauucaucc...................  1       0   S10
....................................................................caucaccggguaaacauucauccc..................  1       0   S10
....................................................................caucaccggguaaacauucaucccc.................  4       0   S10
.....................................................................aucaccggguaaacauucaucc...................  1       0   S10
.....................................................................aucaccggguaaacauucauccc..................  22      0   S10
.....................................................................aucaccggguaaacauucaucccc.................  1027    0   S10
.....................................................................aucaccggguaaacauucaucccca................  1       0   S10
......................................................................ucaccggguaaacauucau.....................  11      0   S10
......................................................................ucaccggguaaacauucauc....................  58      0   S10
......................................................................ucaccggguaaacauucaucc...................  363     0   S10
......................................................................ucaccggguaaacauucauccc..................  4943    0   S10
......................................................................ucaccggguaaacauucaucccc.................  156586  0   S10
......................................................................ucaccggguaaacauucaucccca................  849     0   S10
......................................................................ucaccggguaaacauucauccccau...............  25      0   S10
......................................................................ucaccggguaaacauucauccccaugu.............  1       0   S10
.......................................................................caccggguaaacauucaucccc.................  72      0   S10
........................................................................accggguaaacauucaucccc.................  15      0   S10
.........................................................................ccggguaaacauucauccc..................  1       0   S10
.........................................................................ccggguaaacauucaucccc.................  11      0   S10
..........................................................................cggguaaacauucauccc..................  1       0   S10
..........................................................................cggguaaacauucaucccc.................  2       0   S10
...........................................................................ggguaaacauucaucccc.................  12      0   S10

................................gguugaauguuccuggugu...........................................................  3       0   S06
................................gguugaauguuccugguguu..........................................................  7       0   S06
................................gguugaauguuccugguguug.........................................................  613     0   S06
................................gguugaauguuccugguguugc........................................................  4       0   S06
.................................guugaauguuccugguguugc........................................................  1       0   S06
..................................uugaauguuccugguguugca.......................................................  1       0   S06
.....................................................cauucuuuaaacuugcaucaccggguaaacauucaucccc.................  1       0   S06
....................................................................caucaccggguaaacauucauccc..................  1       0   S06
.....................................................................aucaccggguaaacauucauccc..................  2       0   S06
.....................................................................aucaccggguaaacauucaucccc.................  23      0   S06
......................................................................ucaccggguaaacauucau.....................  2       0   S06
```

```
                                Star                                 Mature
ucauccugcugaaugauaaguuucuuuuauuggguugaauguuccugguguugcauucuuuaaacuugcaucaccggguaaacauucauccccauguugaaacgcauaac
......................................................................ucaccggguaaacauucauc....................    7       0       S06
......................................................................ucaccggguaaacauucaucc...................    13      0       S06
......................................................................ucaccggguaaacauucauccc..................    152     0       S06
......................................................................ucaccggguaaacauucaucccc.................    1372    0       S06
......................................................................ucaccggguaaacauucaucccca................    8       0       S06
..........................................................................cggguaaacauucaucccc.................    2       0       S06

................................gguugaauguuccugguguug.........................................................    6       0       S01
..................................uugaauguuccugguguugc........................................................    1       0       S01
.....................................................................aucaccggguaaacauucaucccc.................    3       0       S01
......................................................................ucaccggguaaacauucauc....................    1       0       S01
......................................................................ucaccggguaaacauucaucc...................    10      0       S01
......................................................................ucaccggguaaacauucauccc..................    61      0       S01
......................................................................ucaccggguaaacauucaucccc.................    157     0       S01
......................................................................ucaccggguaaacauucaucccca................    8       0       S01

................................gguugaauguuccuggugu...........................................................    2       0       S07
................................gguugaauguuccugguguu..........................................................    48      0       S07
................................gguugaauguuccugguguug.........................................................    2745    0       S07
................................gguugaauguuccugguguugc........................................................    12      0       S07
................................gguugaauguuccugguguugcauu.....................................................    1       0       S07
.................................guugaauguuccugguguu..........................................................    2       0       S07
.................................guugaauguuccugguguug.........................................................    7       0       S07
.................................guugaauguuccugguguugc........................................................    2       0       S07
..................................uugaauguuccugguguug.........................................................    2       0       S07
..................................uugaauguuccugguguugca.......................................................    2       0       S07
.....................................aauguuccugguguugcauuc....................................................    1       0       S07
.....................................................cauucuuuaaacuugcaucaccggguaaacauucaucccc.................    1       0       S07
....................................................................caucaccggguaaacauucauccc..................    1       0       S07
....................................................................caucaccggguaaacauucaucccc.................    1       0       S07
.....................................................................aucaccggguaaacauucauccc..................    3       0       S07
.....................................................................aucaccggguaaacauucaucccc.................    201     0       S07
......................................................................ucaccggguaaacauucau.....................    2       0       S07
......................................................................ucaccggguaaacauucauc....................    12      0       S07
......................................................................ucaccggguaaacauucaucc...................    60      0       S07
......................................................................ucaccggguaaacauucauccc..................    1001    0       S07
......................................................................ucaccggguaaacauucaucccc.................    16524   0       S07
......................................................................ucaccggguaaacauucaucccca................    102     0       S07
......................................................................ucaccggguaaacauucauccccau...............    5       0       S07
.......................................................................caccggguaaacauucaucccc.................    4       0       S07
........................................................................accggguaaacauucaucccc.................    5       0       S07
.........................................................................ccggguaaacauucaucccc.................    1       0       S07
...........................................................................ggguaaacauucaucccc.................    1       0       S07
```

Provisional ID	:	Scaffold1083_21832
Score total	:	45729.8
Score for star read(s)	:	3.9
Score for read counts	:	45723.3
Score for mfe	:	1
Score for randfold	:	1.6
Score for cons. seed	:	
Total read count	:	89696
Mature read count	:	88515
Loop read count	:	0
Star read count	:	1181

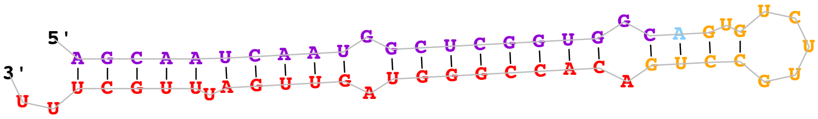

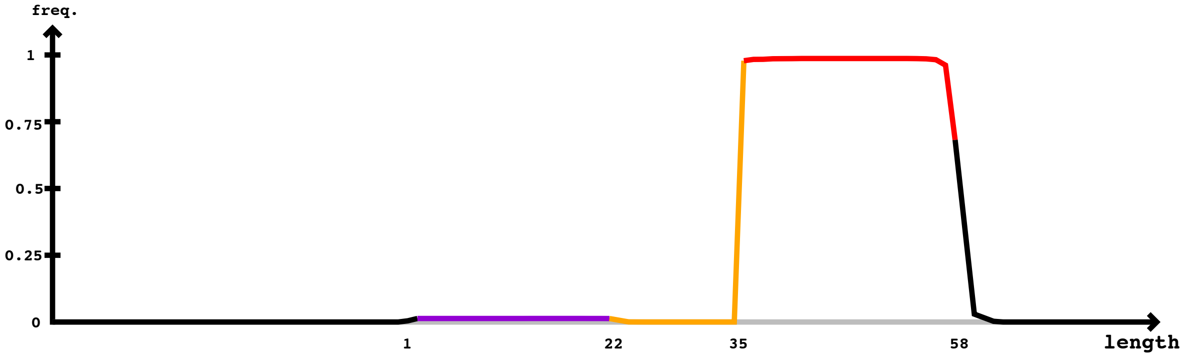

Star Mature

```
5'- aacaguguuuuauauuaauuuaauccuuuucaaaccagcaaucaauggcucgguggcagugucuugccugacaccggguaguugauuugcuuuuaaaccugaggaccaguguu   -3'  obs
    aacaguguuuuauauuaauuuaauccuuuucaaaccagcaaucaauggcucgguggcagugucuugccugacaccggguaguugauuugcuuuuaaaccugaggaccaguguu        exp
    ((((.((.....((......)).(((((........((((((((((.((((((((.(((.(.....)))).)))))))).))))).))))).........))))).)).))))   reads   mm   sample
    ...................................cagcaaucaauggcucgguggc........................................................   1       0    S10
    ....................................agcaaucaauggcucgguggc........................................................   6       0    S10
    ....................................agcaaucaauggcucgguggca.......................................................   2       0    S10
    ....................................agcaaucaauggcucgguggcaguguc..................................................   1       0    S10
    ......................................................................acaccggguaguugauuug........................   3       0    S10
    ......................................................................acaccggguaguugauuugc.......................   10      0    S10
    ......................................................................acaccggguaguugauuugcu......................   66      0    S10
    ......................................................................acaccggguaguugauuugcuu.....................   709     0    S10
    ......................................................................acaccggguaguugauuugcuuu....................   649     0    S10
    ......................................................................acaccggguaguugauuugcuuuu...................   303     0    S10
    ......................................................................acaccggguaguugauuugcuuuua..................   8       0    S10
    ......................................................................acaccggguaguugauuugcuuuuaa.................   2       0    S10
    .......................................................................caccggguaguugauuugcuu.....................   3       0    S10
    .......................................................................caccggguaguugauuugcuuu....................   2       0    S10
    .......................................................................caccggguaguugauuugcuuuu...................   2       0    S10
    ...........................................................................ggguaguugauuugcuuu....................   1       0    S10

    ...................................cagcaaucaauggcucgguggc........................................................   21      0    S05
    ...................................cagcaaucaauggcucgguggca.......................................................   8       0    S05
    ....................................agcaaucaauggcucgguggc........................................................   27      0    S05
    ....................................agcaaucaauggcucgguggca.......................................................   19      0    S05
    ...............................................................uugccugacaccggguaguugauuugcuu......................   1       0    S05
    ......................................................................acaccggguaguugauuu.........................   3       0    S05
    ......................................................................acaccggguaguugauuug........................   6       0    S05
    ......................................................................acaccggguaguugauuugc.......................   28      0    S05
    ......................................................................acaccggguaguugauuugcu......................   176     0    S05
    ......................................................................acaccggguaguugauuugcuu.....................   2696    0    S05
    ......................................................................acaccggguaguugauuugcuuu....................   3130    0    S05
    ......................................................................acaccggguaguugauuugcuuuu...................   2196    0    S05
    ......................................................................acaccggguaguugauuugcuuuua..................   140     0    S05
    ......................................................................acaccggguaguugauuugcuuuuaa.................   45      0    S05
    ......................................................................acaccggguaguugauuugcuuuuaaa................   4       0    S05
    ......................................................................acaccggguaguugauuugcuuuuaaac..............   1       0    S05
```

Star **Mature**

```
aacaguguuuuauauuaauuuaauccuuuucaaaccagcaaucaauggcucgguggcagugucuugccugacaccggguaguugauuugcuuuuaaaccugaggaccaguguu
.......................................................................caccggguaguugauuugcu......................   1      0   S05
.......................................................................caccggguaguugauuugcuu.....................   6      0   S05
.......................................................................caccggguaguugauuugcuuu....................   10     0   S05
.......................................................................caccggguaguugauuugcuuuu...................   27     0   S05
.......................................................................caccggguaguugauuugcuuuua..................   2      0   S05
........................................................................accggguaguugauuugcuuuu...................   3      0   S05
.........................................................................ccggguaguugauuugcuu.....................   9      0   S05
.........................................................................ccggguaguugauuugcuuu....................   8      0   S05
.........................................................................ccggguaguugauuugcuuuu...................   3      0   S05
..........................................................................cggguaguugauuugcuu.....................   1      0   S05
..........................................................................cggguaguugauuugcuuu....................   1      0   S05
...........................................................................ggguaguugauuugcuuu....................   2      0   S05
...........................................................................ggguaguugauuugcuuuu...................   1      0   S05
...........................................................................ggguaguugauuugcuuuua..................   1      0   S05
............................................................................gguaguugauuugcuuuu...................   4      0   S05

...................................cagcaaucaauggcucggug..........................................................   1      0   S07
...................................cagcaaucaauggcucggugg.........................................................   1      0   S07
...................................cagcaaucaauggcucgguggc........................................................   14     0   S07
...................................cagcaaucaauggcucgguggca.......................................................   2      0   S07
....................................agcaaucaauggcucggug..........................................................   1      0   S07
....................................agcaaucaauggcucgguggc........................................................   16     0   S07
....................................agcaaucaauggcucgguggca.......................................................   6      0   S07
......................................................................acaccggguaguugauuug........................   6      0   S07
......................................................................acaccggguaguugauuugc.......................   23     0   S07
......................................................................acaccggguaguugauuugcu......................   108    0   S07
......................................................................acaccggguaguugauuugcuu.....................   1483   0   S07
......................................................................acaccggguaguugauuugcuuu....................   1790   0   S07
......................................................................acaccggguaguugauuugcuuuu...................   1061   0   S07
......................................................................acaccggguaguugauuugcuuuua..................   17     0   S07
......................................................................acaccggguaguugauuugcuuuuaa.................   10     0   S07
.......................................................................caccggguaguugauuugcuu.....................   4      0   S07
.......................................................................caccggguaguugauuugcuuu....................   3      0   S07
.......................................................................caccggguaguugauuugcuuuu...................   8      0   S07
.......................................................................caccggguaguugauuugcuuuuaa.................   1      0   S07
........................................................................accggguaguugauuugcuu.....................   1      0   S07
.........................................................................ccggguaguugauuugcuu.....................   2      0   S07
.........................................................................ccggguaguugauuugcuuu....................   5      0   S07
.........................................................................ccggguaguugauuugcuuuu...................   1      0   S07
...........................................................................ggguaguugauuugcuuuu...................   1      0   S07
............................................................................gguaguugauuugcuuuu...................   3      0   S07
............................................................................gguaguugauuugcuuuA...................   1      1   S07

...................................cagcaaucaauggcucggugg.........................................................   1      0   S06
...................................cagcaaucaauggcucgguggc........................................................   13     0   S06
...................................cagcaaucaauggcucgguggca.......................................................   11     0   S06
...................................cagcaaucaauggcucgguggcag......................................................   2      0   S06
....................................agcaaucaauggcucgguggc........................................................   27     0   S06
....................................agcaaucaauggcucgguggca.......................................................   8      0   S06
....................................agcaaucaauggcucgguggcagu.....................................................   1      0   S06
......................................................................acaccggguaguugauuu.........................   6      0   S06
......................................................................acaccggguaguugauuug........................   5      0   S06
......................................................................acaccggguaguugauuugc.......................   22     0   S06
......................................................................acaccggguaguugauuugcu......................   138    0   S06
......................................................................acaccggguaguugauuugcuu.....................   2204   0   S06
......................................................................acaccggguaguugauuugcuuu....................   2233   0   S06
......................................................................acaccggguaguugauuugcuuuu...................   1710   0   S06
......................................................................acaccggguaguugauuugcuuuua..................   66     0   S06
......................................................................acaccggguaguugauuugcuuuuaa.................   21     0   S06
......................................................................acaccggguaguugauuugcuuuuaaa................   3      0   S06
.......................................................................caccggguaguugauuugc.......................   2      0   S06
.......................................................................caccggguaguugauuugcuu.....................   4      0   S06
.......................................................................caccggguaguugauuugcuuu....................   12     0   S06
.......................................................................caccggguaguugauuugcuuuu...................   15     0   S06
.......................................................................caccggguaguugauuugcuuuua..................   1      0   S06
........................................................................accggguaguugauuugcuuuu...................   2      0   S06
.........................................................................ccggguaguugauuugcuu.....................   8      0   S06
.........................................................................ccggguaguugauuugcuuu....................   1      0   S06
.........................................................................ccggguaguugauuugcuuuu...................   10     0   S06
..........................................................................cggguaguugauuugcuu.....................   1      0   S06
```

Star Mature

```
aacaguguuuuauauuaauuuaaauccuuuucaaaccagcaaucaauggcucgguggcagugucuugccugaacaccggguaguugauuugcuuuuaaaccugaggaccaguguu
.............................................................................ggguaguugauuugcuuuu...................	1	0	S06
..............................................................................gguaguugauuugcuuuu...................	3	0	S06

....................................cagcaaucaauggcucgg.............................................................	1	0	S01
....................................cagcaaucaauggcucgguggc.........................................................	51	0	S01
....................................cagcaaucaauggcucgguggca........................................................	47	0	S01
....................................cagcaaucaauggcucgguggcag.......................................................	2	0	S01
.....................................agcaaucaauggcucggugg..........................................................	1	0	S01
.....................................agcaaucaauggcucgguggc.........................................................	57	0	S01
.....................................agcaaucaauggcucgguggca........................................................	77	0	S01
.....................................agcaaucaauggcucgguggcag.......................................................	4	0	S01
.....................................agcaaucaauggcucgguggcaguguc...................................................	1	0	S01
.....................................agcaaucaauggcucgguggcagugucu..................................................	2	0	S01
......................................gcaaucaauggcucgguggc.........................................................	1	0	S01
........................................aaucaauggcucgguggca........................................................	1	0	S01
.................................................cucgguggcagugucuugccuga...........................................	1	0	S01
......................................................................gacaccggguaguugauuugcuu.....................	1	0	S01
........................................................................acaccggguaguugauuu.........................	3	0	S01
........................................................................acaccggguaguugauuug........................	11	0	S01
........................................................................acaccggguaguugauuugc.......................	41	0	S01
........................................................................acaccggguaguugauuugcu......................	304	0	S01
........................................................................acaccggguaguugauuugcuu.....................	3423	0	S01
........................................................................acaccggguaguugauuugcuuu....................	5426	0	S01
........................................................................acaccggguaguugauuugcuuuu...................	3474	0	S01
........................................................................acaccggguaguugauuugcuuuua..................	116	0	S01
........................................................................acaccggguaguugauuugcuuuuaa.................	249	0	S01
........................................................................acaccggguaguugauuugcuuuuaaa................	39	0	S01
.........................................................................caccggguaguugauuugcuu.....................	1	0	S01
.........................................................................caccggguaguugauuugcuuu....................	12	0	S01
.........................................................................caccggguaguugauuugcuuuu...................	57	0	S01
.........................................................................caccggguaguugauuugcuuuua..................	1	0	S01
.........................................................................caccggguaguugauuugcuuuuaa.................	2	0	S01
..........................................................................accggguaguugauuugcuuuua..................	1	0	S01
..........................................................................accggguaguugauuugcuuuuaa.................	1	0	S01
...........................................................................ccggguaguugauuugcuu.....................	9	0	S01
...........................................................................ccggguaguugauuugcuuu....................	15	0	S01
...........................................................................ccggguaguugauuugcuuuu...................	14	0	S01
...........................................................................ccggguaguugauuugcuuuuaa.................	1	0	S01
............................................................................cggguaguugauuugcuuu....................	1	0	S01
............................................................................cggguaguugauuugcuuuu...................	1	0	S01
.............................................................................ggguaguugauuugcuuu....................	1	0	S01
..............................................................................gguaguugauuugcuuuA...................	1	1	S01
..............................................................................gguaguugauuugcuuuu...................	7	0	S01
..............................................................................gguaguugauuugcuuuua..................	1	0	S01

....................................cagcaaucaauggcucgguggc.........................................................	18	0	S08
....................................cagcaaucaauggcucgguggca........................................................	8	0	S08
.....................................agcaaucaauggcucgguggc.........................................................	4	0	S08
.....................................agcaaucaauggcucgguggca........................................................	2	0	S08
........................................aaucaauggcucgguggcagugucuugc...............................................	1	0	S08
........................................................................acaccggguaguugauuu.........................	3	0	S08
........................................................................acaccggguaguugauuug........................	2	0	S08
........................................................................acaccggguaguugauuugc.......................	21	0	S08
........................................................................acaccggguaguugauuugcu......................	138	0	S08
........................................................................acaccggguaguugauuugcuu.....................	1522	0	S08
........................................................................acaccggguaguugauuugcuuu....................	1208	0	S08
........................................................................acaccggguaguugauuugcuuuu...................	815	0	S08
........................................................................acaccggguaguugauuugcuuuua..................	26	0	S08
........................................................................acaccggguaguugauuugcuuuuaa.................	3	0	S08
.........................................................................caccggguaguugauuugcuu.....................	2	0	S08
.........................................................................caccggguaguugauuugcuuu....................	3	0	S08
.........................................................................caccggguaguugauuugcuuuu...................	14	0	S08
..........................................................................accggguaguugauuugcuu.....................	1	0	S08
..........................................................................accggguaguugauuugcuuu....................	2	0	S08
..........................................................................accggguaguugauuugcuuuu...................	1	0	S08
...........................................................................ccggguaguugauuugcuu.....................	8	0	S08
...........................................................................ccggguaguugauuugcuuu....................	8	0	S08
...........................................................................ccggguaguugauuugcuuuu...................	5	0	S08
............................................................................cggguaguugauuugcuuu....................	2	0	S08
............................................................................cggguaguugauuugcuuuu...................	1	0	S08
```

Star **Mature**

aacaguguuuuauauuaauuuaaauccuuuucaaaccagcaaucaauggcucgguggcagugucuugccugacaccggguaguugauuugcuuuuaaaccugaggaccaguguu			
....ggguaguugauuugcuuu....................	3	0	S08
....ggguaguugauuugcuuuu...................	2	0	S08
....gguaguugauuugcuuuu...................	6	0	S08
....................................cagcaaucaauggcucggu...	1	0	S03
....................................cagcaaucaauggcucggugg...	1	0	S03
....................................cagcaaucaauggcucgguggc..	36	0	S03
....................................cagcaaucaauggcucgguggca...	17	0	S03
.....................................agcaaucaauggcucggugg...	1	0	S03
.....................................agcaaucaauggcucgguggc..	69	0	S03
.....................................agcaaucaauggcucgguggca...	61	0	S03
.....................................agcaaucaauggcucgguggcag..	3	0	S03
.....................................agcaaucaauggcucgguggcagugucuu..	1	0	S03
....gacaccggguaguugauuugc.......................	1	0	S03
....gacaccggguaguugauuugcu......................	1	0	S03
....gacaccggguaguugauuugcuu.....................	1	0	S03
....acaccggguaguugauuu.........................	2	0	S03
....acaccggguaguugauuug........................	15	0	S03
....acaccggguaguugauuugc.......................	35	0	S03
....acaccggguaguugauuugcu......................	310	0	S03
....acaccggguaguugauuugcuu.....................	4089	0	S03
....acaccggguaguugauuugcuuu....................	5792	0	S03
....acaccggguaguugauuugcuuuu...................	3728	0	S03
....acaccggguaguugauuugcuuuua..................	227	0	S03
....acaccggguaguugauuugcuuuuaa.................	117	0	S03
....acaccggguaguugauuugcuuuuaaa................	8	0	S03
....caccggguaguugauuugcu......................	2	0	S03
....caccggguaguugauuugcuu.....................	5	0	S03
....caccggguaguugauuugcuuu....................	15	0	S03
....caccggguaguugauuugcuuuu...................	39	0	S03
....caccggguaguugauuugcuuuua..................	4	0	S03
....accggguaguugauuugcuu.....................	1	0	S03
....accggguaguugauuugcuuu....................	2	0	S03
....ccggguaguugauuugcuu.....................	6	0	S03
....ccggguaguugauuugcuuu....................	8	0	S03
....ccggguaguugauuugcuuuu...................	8	0	S03
....ccggguaguugauuugcuuuua..................	1	0	S03
....cggguaguugauuugcuu.....................	1	0	S03
....cggguaguugauuugcuuu....................	2	0	S03
....cggguaguugauuugcuuuu...................	2	0	S03
....cggguaguugauuugcuuuua..................	1	0	S03
....cggguaguugauuugcuuuuaa.................	1	0	S03
....ggguaguugauuugcuuu....................	2	0	S03
....ggguaguugauuugcuuuu...................	1	0	S03
....gguaguugauuugcuuuu...................	4	0	S03
....gguaguugauuugcuuuAa..................	1	1	S03
....................................cagcaaucaauggcucggugg...	1	0	S09
....................................cagcaaucaauggcucgguggc..	5	0	S09
....................................cagcaaucaauggcucgguggca...	1	0	S09
.....................................agcaaucaauggcucggugg...	1	0	S09
.....................................agcaaucaauggcucgguggc..	8	0	S09
....acaccggguaguugauuu.........................	1	0	S09
....acaccggguaguugauuug........................	2	0	S09
....acaccggguaguugauuugc.......................	7	0	S09
....acaccggguaguugauuugcu......................	35	0	S09
....acaccggguaguugauuugcuu.....................	310	0	S09
....acaccggguaguugauuugcuuu....................	373	0	S09
....acaccggguaguugauuugcuuuu...................	214	0	S09
....acaccggguaguugauuugcuuuua..................	5	0	S09
....caccggguaguugauuugcuuu....................	1	0	S09
....caccggguaguugauuugcuuuu...................	3	0	S09
....................................cagcaaucaauggcucggug..	1	0	S04
....................................cagcaaucaauggcucgguggc..	36	0	S04
....................................cagcaaucaauggcucgguggca...	24	0	S04
.....................................agcaaucaauggcucggu...	1	0	S04
.....................................agcaaucaauggcucggugg...	2	0	S04
.....................................agcaaucaauggcucgguggc..	85	0	S04
.....................................agcaaucaauggcucgguggca...	45	0	S04

Star **Mature**

```
aacaguguuuuauauuaauuuaauccuuuucaaaccagcaaucaauggcucgguggcagugucuugccugacaccggguaguugauuugcuuuuaaaccugaggaccaguguu
....................................agcaaucaauggcucgguggcag......................................................    6     0    S04
....................................agcaaucaauggcucgguggcagu.....................................................    2     0    S04
....................................agcaaucaauggcucgguggcaguguc..................................................    1     0    S04
....................................agcaaucaauggcucgguggcagugucu.................................................    1     0    S04
.......................................aaucaauggcucgguggca.......................................................    1     0    S04
.....................................................................gacaccggguaguugauuugcuu.....................    1     0    S04
......................................................................acaccggguaguugauuu.........................    8     0    S04
......................................................................acaccggguaguugauuug........................    18    0    S04
......................................................................acaccggguaguugauuugc.......................    43    0    S04
......................................................................acaccggguaguugauuugcu......................    324   0    S04
......................................................................acaccggguaguugauuugcuu.....................    4859  0    S04
......................................................................acaccggguaguugauuugcuuu....................    5418  0    S04
......................................................................acaccggguaguugauuugcuuuu...................    3775  0    S04
......................................................................acaccggguaguugauuugcuuuua..................    183   0    S04
......................................................................acaccggguaguugauuugcuuuuaa.................    81    0    S04
......................................................................acaccggguaguugauuugcuuuuaaa................    2     0    S04
.......................................................................caccggguaguugauuugcu......................    4     0    S04
.......................................................................caccggguaguugauuugcuu.....................    7     0    S04
.......................................................................caccggguaguugauuugcuuu....................    22    0    S04
.......................................................................caccggguaguugauuugcuuuu...................    50    0    S04
........................................................................accggguaguugauuugcuu.....................    1     0    S04
........................................................................accggguaguugauuugcuuu....................    1     0    S04
........................................................................accggguaguugauuugcuuuu...................    1     0    S04
.........................................................................ccggguaguugauuugcu......................    1     0    S04
.........................................................................ccggguaguugauuugcuu.....................    5     0    S04
.........................................................................ccggguaguugauuugcuuu....................    13    0    S04
.........................................................................ccggguaguugauuugcuuuu...................    7     0    S04
..........................................................................cggguaguugauuugcuu.....................    4     0    S04
..........................................................................cggguaguugauuugcuuuu...................    1     0    S04
..........................................................................cggguaguugauuugcuuuua..................    1     0    S04
............................................................................gguaguugauuugcuuuu...................    5     0    S04
............................................................................gguaguugauuugcuuuua..................    1     0    S04

...................................cagcaaucaauggcucggu...........................................................    1     0    S02
...................................cagcaaucaauggcucgguggg.........................................................    3     0    S02
...................................cagcaaucaauggcucgguggc........................................................    53    0    S02
...................................cagcaaucaauggcucgguggca.......................................................    21    0    S02
....................................agcaaucaauggcucggu...........................................................    1     0    S02
....................................agcaaucaauggcucggugg.........................................................    2     0    S02
....................................agcaaucaauggcucgguggc........................................................    112   0    S02
....................................agcaaucaauggcucgguggca.......................................................    108   0    S02
....................................agcaaucaauggcucgguggcag......................................................    7     0    S02
...............................................gcucgguggcagugucuugcc.............................................    1     0    S02
................................................cucgguggcagugucuugccug...........................................    2     0    S02
.................................................ucgguggcagugucuugccuga..........................................    1     0    S02
.....................................................................gacaccggguaguugauuugcu......................    2     0    S02
.....................................................................gacaccggguaguugauuugcuu.....................    1     0    S02
......................................................................acaccggguaguugauuu.........................    10    0    S02
......................................................................acaccggguaguugauuug........................    15    0    S02
......................................................................acaccggguaguugauuugc.......................    52    0    S02
......................................................................acaccggguaguugauuugcu......................    227   0    S02
......................................................................acaccggguaguugauuugcuu.....................    3720  0    S02
......................................................................acaccggguaguugauuugcuuu....................    7704  0    S02
......................................................................acaccggguaguugauuugcuuuu...................    6885  0    S02
......................................................................acaccggguaguugauuugcuuuua..................    392   0    S02
......................................................................acaccggguaguugauuugcuuuuaa.................    682   0    S02
......................................................................acaccggguaguugauuugcuuuuaaa................    180   0    S02
......................................................................acaccggguaguugauuugcuuuuaaac...............    1     0    S02
.......................................................................caccggguaguugauuugcuu.....................    2     0    S02
.......................................................................caccggguaguugauuugcuuu....................    27    0    S02
.......................................................................caccggguaguugauuugcuuuu...................    78    0    S02
.......................................................................caccggguaguugauuugcuuuua..................    3     0    S02
.......................................................................caccggguaguugauuugcuuuuaa.................    1     0    S02
........................................................................accggguaguugauuugcuuuu...................    1     0    S02
........................................................................accggguaguugauuugcuuuuaa.................    1     0    S02
.........................................................................ccggguaguugauuugcuu.....................    3     0    S02
.........................................................................ccggguaguugauuugcuuu....................    14    0    S02
.........................................................................ccggguaguugauuugcuuuu...................    9     0    S02
..........................................................................cggguaguugauuugcuu.....................    3     0    S02
..........................................................................cggguaguugauuugcuuu....................    1     0    S02
```

Star Mature

```
aacaguguuuuauauuaauuuaauccuuuucaaaccagcaaucaauggcucgguggcagugucuugccugacaccggguaguugauuugcuuuuaaaccugaggaccaguguu
..........................................................................cggguaguugauuugcuuuu...................	1	0	S02
...........................................................................ggguaguugauuugcuuu....................	2	0	S02
...........................................................................ggguaguugauuugcuuuu...................	1	0	S02
............................................................................gguaguugauuugcuuuA...................	1	1	S02
............................................................................gguaguugauuugcuuuu...................	8	0	S02
............................................................................gguaguugauuugcuuuua..................	1	0	S02
............................................................................gguaguugauuugcuuuuaa.................	1	0	S02
```

Provisional ID	:	Scaffold248_6765
Score total	:	45010
Score for star read(s)	:	3.9
Score for read counts	:	45005.4
Score for mfe	:	-0.9
Score for randfold	:	1.6
Score for cons. seed	:	
Total read count	:	88288
Mature read count	:	87892
Loop read count	:	0
Star read count	:	396

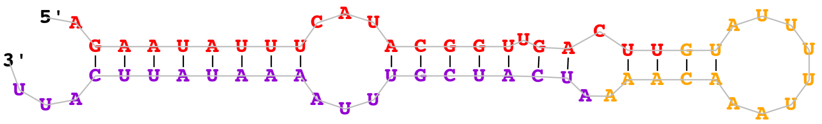

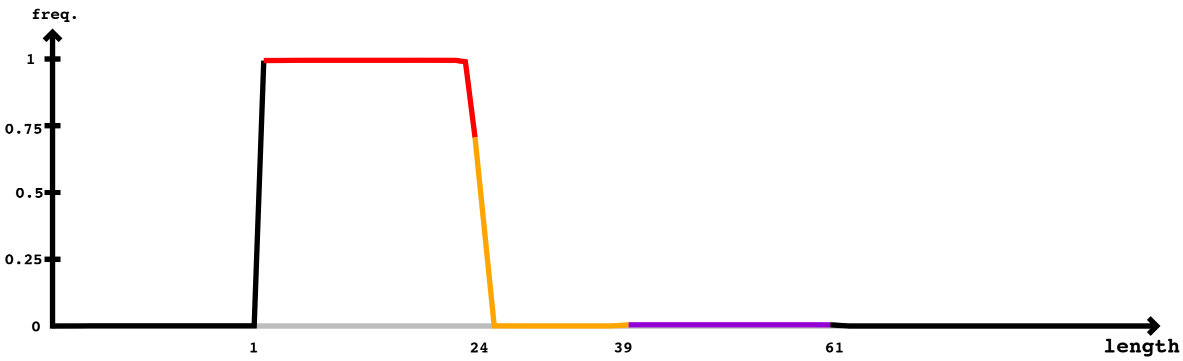

Mature **Star**

```
5'- guugcuugccucugacggaaagaauauuucauacgguugacuuguauuuuuaaacaaaaucaucguuuaaaauauucauuuuguuugaguugauuuuucaacagcagaagauu   -3'    obs
    guugcuugccucugacggaaagaauauuucauacgguugacuuguauuuuuaaacaaaaucaucguuuaaaauauucauuuuguuugaguugauuuuucaacagcagaagauu          exp
    .((((.....((.(((((((.((((((((...(((((.((.((((........))))..)))))))...)))))))).))))))).))(((((....))))).)))).....   reads    mm     sample
    ....................agaauauuucauacgguugac........................................................................   29       0      S02
    ....................agaauauuucauacgguugacu.......................................................................   652      0      S02
    ....................agaauauuucauacgguugacuu......................................................................   1097     0      S02
    ....................agaauauuucauacgguugacuug.....................................................................   3        0      S02
    ....................agaauauuucauacgguugacuuguauu.................................................................   1        0      S02
    ....................agaauauuucauacgguugacuuguauuuuuaaac..........................................................   1        0      S02
    ....................agaauauuucauacgguugacuuguauuuuuaaacaaa.......................................................   1        0      S02
    ......................aauauuucauacgguugacu.......................................................................   1        0      S02
    ......................aauauuucauacgguugacuu......................................................................   1        0      S02
    ........................uauuucauacgguugacuu......................................................................   1        0      S02
    .........................................................aaucaucguuuaaaauauucau..................................   1        0      S02
    .........................................................aaucaucguuuaaaauauucauu.................................   2        0      S02
    .........................................................aaucaucguuuaaaauauucauuu................................   1        0      S02
    ..........................................................aucaucguuuaaaauauucauu.................................   7        0      S02

    guugcuugccucugacggaa.............................................................................................   2        0      S04
    .uugcuugccucugacggaa.............................................................................................   1        0      S04
    ..ugcuugccucugacggaa.............................................................................................   6        0      S04
    ....................agaauauuucauacgguuga.........................................................................   5        0      S04
    ....................agaauauuucauacgguugac........................................................................   68       0      S04
    ....................agaauauuucauacgguugacu.......................................................................   2838     0      S04
    ....................agaauauuucauacgguugacuu......................................................................   11845    0      S04
    ....................agaauauuucauacgguugacuug.....................................................................   36       0      S04
    ....................agaauauuucauacgguugacuuguauu.................................................................   1        0      S04
    ....................agaauauuucauacgguugacuuguauuu................................................................   1        0      S04
    ....................agaauauuucauacgguugacuuguauuuuuaaacaa........................................................   2        0      S04
    ....................agaauauuucauacgguugacuuguauuuuuaaacaaa.......................................................   2        0      S04
    .....................gaauauuucauacgguugacuu......................................................................   1        0      S04
    .......................auauuucauacgguugacu.......................................................................   1        0      S04
    .......................auauuucauacgguugacuu......................................................................   1        0      S04
    .........................auuucauacgguugacuu......................................................................   2        0      S04
    .........................................................aaucaucguuuaaaauauucau..................................   2        0      S04
    .........................................................aaucaucguuuaaaauauucauu.................................   52       0      S04
```

Mature **Star**

```
guugcuugccucugacggaaagaauauuucauacgguugacuuguauuuuuaaacaaaaucaucguuuaaaauauucauuuuguuugaguugauuuuucaacagcagaagauu
.........................................................aaucaucguuuaaaauauucauuu................................	1	0	S04
..........................................................aucaucguuuaaaauauucauu.................................	73	0	S04
..........................................................aucaucguuuaaaauauucauuu................................	6	0	S04

....................agaauauuucauacgguuga.........................................................................	1	0	S08
....................agaauauuucauacgguugac........................................................................	15	0	S08
....................agaauauuucauacgguugacu.......................................................................	1173	0	S08
....................agaauauuucauacgguugacuu......................................................................	2203	0	S08
....................agaauauuucauacgguugacuug.....................................................................	6	0	S08
.....................gaauauuucauacgguugacu.......................................................................	1	0	S08
.....................gaauauuucauacgguugacuu......................................................................	2	0	S08
......................aauauuucauacgguugac........................................................................	1	0	S08
......................aauauuucauacgguugacu.......................................................................	1	0	S08
......................aauauuucauacgguugacuu......................................................................	2	0	S08
.......................auauuucauacgguugacu.......................................................................	12	0	S08
.......................auauuucauacgguugacuu......................................................................	8	0	S08
........................uauuucauacgguugacuu......................................................................	3	0	S08
.........................auuucauacgguugacuu......................................................................	2	0	S08
.................................cgguugacuuguauuuuuaaacaa........................................................	1	0	S08
..........................................................aucaucguuuaaaauauucauu.................................	8	0	S08

....................agaauauuucauacgguug..........................................................................	5	0	S09
....................agaauauuucauacgguuga.........................................................................	5	0	S09
....................agaauauuucauacgguugac........................................................................	42	0	S09
....................agaauauuucauacgguugacu.......................................................................	3944	0	S09
....................agaauauuucauacgguugacuu......................................................................	5428	0	S09
....................agaauauuucauacgguugacuug.....................................................................	19	0	S09
......................aauauuucauacgguugacuu......................................................................	1	0	S09
.......................auauuucauacgguugacu.......................................................................	6	0	S09
.......................auauuucauacgguugacuu......................................................................	1	0	S09
........................uauuucauacgguugacuu......................................................................	2	0	S09
..........................................................aucaucguuuaaaauauucau..................................	1	0	S09
..........................................................aucaucguuuaaaauauucauu.................................	8	0	S09

guugcuugccucugacggaa.............................................................................................	1	0	S03
.uugcuugccucugacggaa.............................................................................................	7	0	S03
..ugcuugccucugacggaa.............................................................................................	6	0	S03
....................agaauauuucauacgguug..........................................................................	2	0	S03
....................agaauauuucauacgguuga.........................................................................	1	0	S03
....................agaauauuucauacgguugac........................................................................	56	0	S03
....................agaauauuucauacgguugacu.......................................................................	1836	0	S03
....................agaauauuucauacgguugacuu......................................................................	7833	0	S03
....................agaauauuucauacgguugacuug.....................................................................	20	0	S03
....................agaauauuucauacgguugacuuguauu.................................................................	2	0	S03
....................agaauauuucauacgguugacuuguauuuuuaaac..........................................................	1	0	S03
....................agaauauuucauacgguugacuuguauuuuuaaacaa........................................................	1	0	S03
....................agaauauuucauacgguugacuuguauuuuuaaacaaa.......................................................	2	0	S03
.....................gaauauuucauacgguugacuu......................................................................	1	0	S03
.......................auauuucauacgguugacuu......................................................................	1	0	S03
........................uauuucauacgguugacuu......................................................................	2	0	S03
.........................auuucauacgguugacuu......................................................................	1	0	S03
..........................uuucauacgguugacuuguauuuuuaaac..........................................................	1	0	S03
.........................................................aaucaucguuuaaaauauucauu.................................	30	0	S03
..........................................................aucaucguuuaaaauauuca...................................	1	0	S03
..........................................................aucaucguuuaaaauauucauu.................................	29	0	S03
..........................................................aucaucguuuaaaauauucauuu................................	5	0	S03

.uugcuugccucugacggaa.............................................................................................	1	0	S07
....................agaauauuucauacgguugac........................................................................	43	0	S07
....................agaauauuucauacgguugacu.......................................................................	2851	0	S07
....................agaauauuucauacgguugacuu......................................................................	7709	0	S07
....................agaauauuucauacgguugacuug.....................................................................	24	0	S07
....................agaauauuucauacgguugacuugu....................................................................	1	0	S07
....................agaauauuucauacgguugacuuguauuuuuaaacaaa.......................................................	1	0	S07
.....................gaauauuucauacgguugacuu......................................................................	2	0	S07
......................aauauuucauacgguugacu.......................................................................	1	0	S07
.......................auauuucauacgguugacuu......................................................................	1	0	S07
.........................................................aaucaucguuuaaaauauucauu.................................	5	0	S07
..........................................................aucaucguuuaaaauauucauu.................................	7	0	S07

....................agaauauuucauacgguug..........................................................................	1	0	S01
```

Mature   Star

```
guugcuugccucugacggaaagaauauuucauacgguugacuuguauuuuuaaacaaaaucaucguuuaaaaauauucauuuuguuugaguugauuuucaacagcagaagauu
....................agaauauuucauacgguugac........................................................................   23     0   S01
....................agaauauuucauacgguugacu.......................................................................   657    0   S01
....................agaauauuucauacgguugacuu......................................................................   843    0   S01
....................agaauauuucauacgguugacuug.....................................................................   1      0   S01
......................aauauuucauacgguugacu.......................................................................   1      0   S01
........................uauuucauacgguugacuu......................................................................   1      0   S01
.........................auuucauacgguugacuu......................................................................   2      0   S01
....................................uugacuuguauuuuuaaacaaa.......................................................   1      0   S01
.........................................................aaucaucguuuaaaaauauucau.................................   1      0   S01
.........................................................aaucaucguuuaaaaauauucauu................................   8      0   S01
..........................................................aucaucguuuaaaaauauucau.................................   1      0   S01
..........................................................aucaucguuuaaaaauauucauu................................   14     0   S01

guugcuugccucugacggaa.............................................................................................   2      0   S06
.uugcuugccucugacggaa.............................................................................................   2      0   S06
..ugcuugccucugacggaa.............................................................................................   4      0   S06
....................agaauauuucauacgguug..........................................................................   1      0   S06
....................agaauauuucauacgguugac........................................................................   88     0   S06
....................agaauauuucauacgguugacu.......................................................................   4314   0   S06
....................agaauauuucauacgguugacuu......................................................................   9824   0   S06
....................agaauauuucauacgguugacuug.....................................................................   27     0   S06
....................agaauauuucauacgguugacuugua...................................................................   1      0   S06
....................agaauauuucauacgguugacuuguauu.................................................................   3      0   S06
....................agaauauuucauacgguugacuuguauuuuuaaacaa........................................................   1      0   S06
....................agaauauuucauacgguugacuuguauuuuuaaacaaa.......................................................   1      0   S06
......................aauauuucauacgguugacu.......................................................................   1      0   S06
......................aauauuucauacgguugacuu......................................................................   1      0   S06
.......................auauuucauacgguugacu.......................................................................   2      0   S06
.......................auauuucauacgguugacuu......................................................................   1      0   S06
........................uauuucauacgguugacu.......................................................................   2      0   S06
........................uauuucauacgguugacuu......................................................................   1      0   S06
.........................auuucauacgguugacuu......................................................................   2      0   S06
....................................uugacuuguauuuuuaaacaa........................................................   1      0   S06
.........................................................aaucaucguuuaaaaauauuc...................................   1      0   S06
.........................................................aaucaucguuuaaaaauauucau.................................   1      0   S06
.........................................................aaucaucguuuaaaaauauucauu................................   25     0   S06
..........................................................aucaucguuuaaaaauauucau.................................   1      0   S06
..........................................................aucaucguuuaaaaauauucauu................................   30     0   S06

....................agaauauuucauacgguu...........................................................................   1      0   S10
....................agaauauuucauacgguuga.........................................................................   5      0   S10
....................agaauauuucauacgguugac........................................................................   46     0   S10
....................agaauauuucauacgguugacu.......................................................................   4143   0   S10
....................agaauauuucauacgguugacuu......................................................................   5677   0   S10
....................agaauauuucauacgguugacuug.....................................................................   17     0   S10
....................agaauauuucauacgguugacuugu....................................................................   2      0   S10
....................agaauauuucauacgguugacuuguauu.................................................................   1      0   S10
.....................gaauauuucauacgguugacuu......................................................................   1      0   S10
.......................auauuucauacgguugacu.......................................................................   1      0   S10
........................uauuucauacgguugacuu......................................................................   1      0   S10
.........................................................aaucaucguuuaaaaauauuca..................................   1      0   S10
.........................................................aaucaucguuuaaaaauauucauu................................   5      0   S10
..........................................................aucaucguuuaaaaauauucauu................................   21     0   S10

guugcuugccucugacggaa.............................................................................................   1      0   S05
.uugcuugccucugacggaa.............................................................................................   3      0   S05
..ugcuugccucugacggaa.............................................................................................   5      0   S05
....................agaauauuucauacgguuga.........................................................................   2      0   S05
....................agaauauuucauacgguugac........................................................................   59     0   S05
....................agaauauuucauacgguugacu.......................................................................   2562   0   S05
....................agaauauuucauacgguugacuu......................................................................   9690   0   S05
....................agaauauuucauacgguugacuug.....................................................................   17     0   S05
....................agaauauuucauacgguugacuugu....................................................................   2      0   S05
....................agaauauuucauacgguugacuuguauu.................................................................   2      0   S05
....................agaauauuucauacgguugacuuguauuuuuaaacaa........................................................   1      0   S05
....................agaauauuucauacgguugacuuguauuuuuaaacaaa.......................................................   1      0   S05
.....................gaauauuucauacgguugacuu......................................................................   1      0   S05
......................aauauuucauacgguugacuu......................................................................   3      0   S05
.......................auauuucauacgguugacu.......................................................................   4      0   S05
.......................auauuucauacgguugacuu......................................................................   2      0   S05
```

```
                    Mature                                   Star
guugcuugccucugacggaaagaauauuucauacgguugacuuguauuuuuaaacaaaaucaucguuuaaaauauucauuuuguuugaguugauuuuucaacagcagaagauu
........................uauuucauacgguugacuu......................................................................	1	0	S05
.........................auuucauacgguugacuu......................................................................	2	0	S05
.........................................................aaucaucguuuaaaauauucauu.................................	18	0	S05
..........................................................aucaucguuuaaaauauucauu.................................	28	0	S05
..........................................................aucaucguuuaaaauauucauuu................................	2	0	S05
```

Provisional ID	:	Scaffold1083_21834
Score total	:	38383.9
Score for star read(s)	:	3.9
Score for read counts	:	38378.2
Score for mfe	:	0.2
Score for randfold	:	1.6
Score for cons. seed	:	
Total read count	:	75289
Mature read count	:	74526
Loop read count	:	0
Star read count	:	763

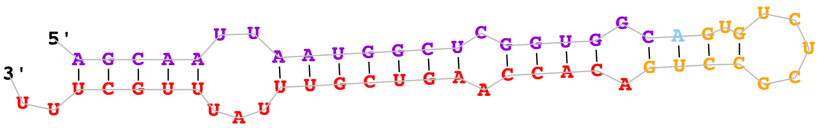

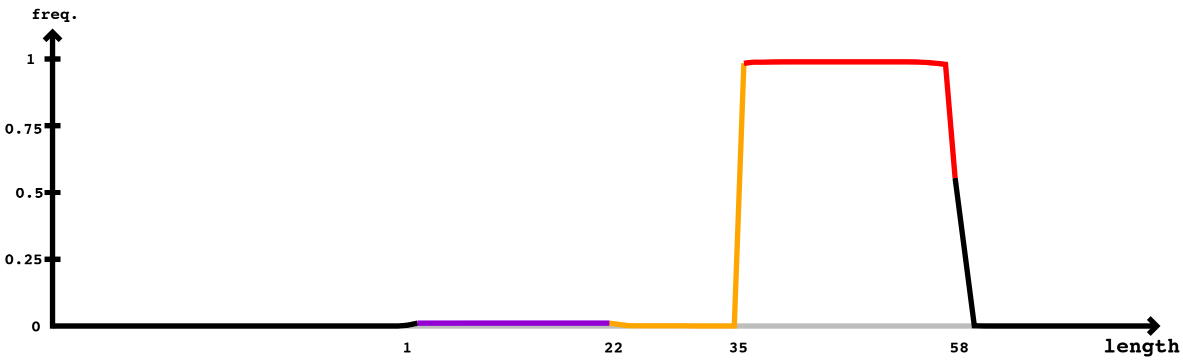

Star **Mature**

```
5'- aacagcuuuuuaauauccuuuaauccuuuucgaaccagcaauuaauggcucgguggcagugucucgccugacaccaagucguuuauuugcuuuuagaccgaaggaccaguguu  -3'   obs
    aacagcuuuuuaauauccuuuaauccuuuucgaaccagcaauuaauggcucgguggcagugucucgccugacaccaagucguuuauuugcuuuuagaccgaaggaccaguguu         exp
    .......................((((((((.....(((((..(((((((.((((.(((.(.....)))).)))).)))))))...)))))....))..))))))........  reads  mm  sample
    ...................................cagcaauuaauggcucggug..........................................................  1      0   S04
    ...................................cagcaauuaauggcucgguggc........................................................  6      0   S04
    ...................................cagcaauuaauggcucgguggca.......................................................  9      0   S04
    ...................................cagcaauuaauggcucgguggcagu.....................................................  2      0   S04
    ....................................agcaauuaauggcucgguggc........................................................  47     0   S04
    ....................................agcaauuaauggcucgguggca.......................................................  30     0   S04
    ....................................agcaauuaauggcucgguggcag......................................................  1      0   S04
    ....................................agcaauuaauggcucgguggcagugucuc................................................  1      0   S04
    .......................................aauuaauggcucgguggca.......................................................  1      0   S04
    ................................................cucgguggcagugucucgccu............................................  1      0   S04
    ......................................................................acaccaagucguuuauuu.........................  12     0   S04
    ......................................................................acaccaagucguuuauuug........................  15     0   S04
    ......................................................................acaccaagucguuuauuugc.......................  39     0   S04
    ......................................................................acaccaagucguuuauuugcu......................  37     0   S04
    ......................................................................acaccaagucguuuauuugcuu.....................  5331   0   S04
    ......................................................................acaccaagucguuuauuugcuuu....................  5607   0   S04
    ......................................................................acaccaagucguuuauuugcuuuu...................  707    0   S04
    ......................................................................acaccaagucguuuauuugcuuuua..................  3      0   S04
    ......................................................................acaccaagucguuuauuugcuuuuag.................  1      0   S04
    .......................................................................caccaagucguuuauuugcuu.....................  19     0   S04
    .......................................................................caccaagucguuuauuugcuuu....................  44     0   S04
    .......................................................................caccaagucguuuauuugcuuuu...................  10     0   S04
    .........................................................................ccaagucguuuauuugcuu.....................  1      0   S04
    .........................................................................ccaagucguuuauuugcuuu....................  4      0   S04
    ..........................................................................caagucguuuauuugcuu.....................  3      0   S04
    ..........................................................................caagucguuuauuugcuuu....................  1      0   S04

    ...................................cagcaauuaauggcucggug..........................................................  1      0   S02
    ...................................cagcaauuaauggcucgguggc........................................................  23     0   S02
    ...................................cagcaauuaauggcucgguggca.......................................................  33     0   S02
    ...................................cagcaauuaauggcucgguggcag......................................................  4      0   S02
    ....................................agcaauuaauggcucgguggc........................................................  100    0   S02
    ....................................agcaauuaauggcucgguggca.......................................................  41     0   S02
```

Star Mature

aacagcuuuuuaauauccuuuaauccuuuucgaaccagcaauuaauggcucgguggcagugucucgccugacaccaagucguuuauuugcuuuuagaccgaaggaccaguguu			
....................................agcaauuaauggcucgguggcag..	6	0	S02
....................................agcaauuaauggcucgguggcagug..	1	0	S02
....................................agcaauuaauggcucgguggcagugucu...	1	0	S02
....................................agcaauuaauggcucgguggcagugucuc..	11	0	S02
....acaccaagucguuuauuu.........................	5	0	S02
....acaccaagucguuuauuug........................	23	0	S02
....acaccaagucguuuauuugc.......................	40	0	S02
....acaccaagucguuuauuugcu......................	17	0	S02
....acaccaagucguuuauuugcuu.....................	4156	0	S02
....acaccaagucguuuauuugcuuu....................	5764	0	S02
....acaccaagucguuuauuugcuuuu...................	1538	0	S02
....acaccaagucguuuauuugcuuuua..................	22	0	S02
....caccaagucguuuauuugcuu.....................	5	0	S02
....caccaagucguuuauuugcuuu....................	21	0	S02
....caccaagucguuuauuugcuuuu...................	38	0	S02
....accaagucguuuauuugcuuu....................	2	0	S02
....ccaagucguuuauuugcuu.....................	3	0	S02
....ccaagucguuuauuugcuuu....................	3	0	S02
....ccaagucguuuauuugcuuuu...................	1	0	S02
....aagucguuuauuugcuuu....................	1	0	S02
....................................agcaauuaauggcucgguggc..	1	0	S09
....................................agcaauuaauggcucgguggcagugucuc..	1	0	S09
....acaccaagucguuuauuug........................	1	0	S09
....acaccaagucguuuauuugc.......................	2	0	S09
....acaccaagucguuuauuugcu......................	51	0	S09
....acaccaagucguuuauuugcuu.....................	740	0	S09
....acaccaagucguuuauuugcuuu....................	715	0	S09
....acaccaagucguuuauuugcuuuu...................	125	0	S09
....acaccaagucguuuauuugcuuuua..................	1	0	S09
....caccaagucguuuauuugcuuuu...................	1	0	S09
....caagucguuuauuugcuuu....................	1	0	S09
...................................cagcaauuaauggcucggu...	1	0	S03
...................................cagcaauuaauggcucggug..	1	0	S03
...................................cagcaauuaauggcucgguggc..	9	0	S03
...................................cagcaauuaauggcucgguggca...	24	0	S03
...................................cagcaauuaauggcucgguggcag..	1	0	S03
...................................cagcaauuaauggcucgguggcagu...	3	0	S03
....................................agcaauuaauggcucgguggc..	44	0	S03
....................................agcaauuaauggcucgguggca...	34	0	S03
....................................agcaauuaauggcucgguggcag..	1	0	S03
....................................agcaauuaauggcucgguggcagugu...	1	0	S03
....................................agcaauuaauggcucgguggcagugucuc..	13	0	S03
....acaccaagucguuuauuu.........................	10	0	S03
....acaccaagucguuuauuug........................	27	0	S03
....acaccaagucguuuauuugc.......................	36	0	S03
....acaccaagucguuuauuugcu......................	41	0	S03
....acaccaagucguuuauuugcuu.....................	5313	0	S03
....acaccaagucguuuauuugcuuu....................	5937	0	S03
....acaccaagucguuuauuugcuuuu...................	983	0	S03
....acaccaagucguuuauuugcuuuua..................	13	0	S03
....acaccaagucguuuauuugcuuuuag.................	2	0	S03
....caccaagucguuuauuugcu......................	1	0	S03
....caccaagucguuuauuugcuu.....................	9	0	S03
....caccaagucguuuauuugcuuu....................	4	0	S03
....caccaagucguuuauuugcuuuu...................	20	0	S03
....accaagucguuuauuugcuuu....................	1	0	S03
....ccaagucguuuauuugcuu.....................	4	0	S03
....ccaagucguuuauuugcuuu....................	4	0	S03
....caagucguuuauuugcuu.....................	1	0	S03
....caagucguuuauuugcuuu....................	3	0	S03
....caagucguuuauuugcuuuu...................	1	0	S03
....aagucguuuauuugcuuu....................	1	0	S03
...................................cagcaauuaauggcucggugg...	1	0	S08
...................................cagcaauuaauggcucgguggc..	10	0	S08
...................................cagcaauuaauggcucgguggca...	4	0	S08
....................................agcaauuaauggcucgguggc..	8	0	S08
....................................agcaauuaauggcucgguggca...	3	0	S08

Star Mature

```
aacagcuuuuuaauauccuuuaaauccuuuucgaaccagcaauuaaauggcucgguggcagugucucgccugacaccaagucguuuauuugcuuuuagaccgaaggaccaguguu
........................................................................acaccaagucguuuauuu.........................  2     0  S08
........................................................................acaccaagucguuuauuug........................  4     0  S08
........................................................................acaccaagucguuuauuugc.......................  25    0  S08
........................................................................acaccaagucguuuauuugcu......................  23    0  S08
........................................................................acaccaagucguuuauuugcuu.....................  2144  0  S08
........................................................................acaccaagucguuuauuugcuuu....................  1647  0  S08
........................................................................acaccaagucguuuauuugcuuuu...................  218   0  S08
........................................................................acaccaagucguuuauuugcuuuua..................  3     0  S08
........................................................................acaccaagucguuuauuugcuuuuag.................  1     0  S08
.........................................................................caccaagucguuuauuugcuu.....................  3     0  S08
.........................................................................caccaagucguuuauuugcuuu....................  5     0  S08
.........................................................................caccaagucguuuauuugcuuuu...................  3     0  S08
...........................................................................ccaagucguuuauuugcuuu....................  4     0  S08
...........................................................................ccaagucguuuauuugcuuuu...................  2     0  S08
............................................................................caagucguuuauuugcuu.....................  1     0  S08
.............................................................................aagucguuuauuugcuuu....................  2     0  S08

....................................cagcaauuaaauggcucgguggc........................................................  18    0  S01
....................................cagcaauuaaauggcucgguggca.......................................................  37    0  S01
....................................cagcaauuaaauggcucgguggcag......................................................  3     0  S01
....................................cagcaauuaaauggcucgguggcagu.....................................................  1     0  S01
.....................................agcaauuaaauggcucgguggc........................................................  55    0  S01
.....................................agcaauuaaauggcucgguggca.......................................................  48    0  S01
.....................................agcaauuaaauggcucgguggcag......................................................  1     0  S01
.....................................agcaauuaaauggcucgguggcaguguc..................................................  1     0  S01
.....................................agcaauuaaauggcucgguggcagugucuc................................................  8     0  S01
.........................................auuaaauggcucgguggcagugucucgccu............................................  1     0  S01
..........................................uuaaauggcucgguggcagugucucgccu............................................  1     0  S01
........................................................................acaccaagucguuuauuu.........................  3     0  S01
........................................................................acaccaagucguuuauuug........................  8     0  S01
........................................................................acaccaagucguuuauuugc.......................  23    0  S01
........................................................................acaccaagucguuuauuugcu......................  10    0  S01
........................................................................acaccaagucguuuauuugcuu.....................  2566  0  S01
........................................................................acaccaagucguuuauuugcuuu....................  4374  0  S01
........................................................................acaccaagucguuuauuugcuuuu...................  696   0  S01
........................................................................acaccaagucguuuauuugcuuuua..................  4     0  S01
........................................................................acaccaagucguuuauuugcuuuuag.................  1     0  S01
.........................................................................caccaagucguuuauuugc.......................  1     0  S01
.........................................................................caccaagucguuuauuugcuu.....................  5     0  S01
.........................................................................caccaagucguuuauuugcuuu....................  19    0  S01
.........................................................................caccaagucguuuauuugcuuuu...................  21    0  S01
...........................................................................ccaagucguuuauuugcuu.....................  1     0  S01
...........................................................................ccaagucguuuauuugcuuu....................  7     0  S01
...........................................................................ccaagucguuuauuugcuuuu...................  2     0  S01

....................................cagcaauuaaauggcucgguggc........................................................  3     0  S06
....................................cagcaauuaaauggcucgguggca.......................................................  11    0  S06
.....................................agcaauuaaauggcucgguggc........................................................  21    0  S06
.....................................agcaauuaaauggcucgguggca.......................................................  13    0  S06
.....................................agcaauuaaauggcucgguggcagugucuc................................................  5     0  S06
....................................................cgguggcagugucucgccugac.........................................  1     0  S06
........................................................................acaccaagucguuuauuu.........................  3     0  S06
........................................................................acaccaagucguuuauuug........................  18    0  S06
........................................................................acaccaagucguuuauuugc.......................  27    0  S06
........................................................................acaccaagucguuuauuugcu......................  11    0  S06
........................................................................acaccaagucguuuauuugcuu.....................  3006  0  S06
........................................................................acaccaagucguuuauuugcuuu....................  2365  0  S06
........................................................................acaccaagucguuuauuugcuuuu...................  596   0  S06
........................................................................acaccaagucguuuauuugcuuuua..................  4     0  S06
.........................................................................caccaagucguuuauuugcuu.....................  3     0  S06
.........................................................................caccaagucguuuauuugcuuu....................  9     0  S06
.........................................................................caccaagucguuuauuugcuuuu...................  14    0  S06
...........................................................................ccaagucguuuauuugcuu.....................  4     0  S06
...........................................................................ccaagucguuuauuugcuuu....................  4     0  S06
............................................................................caagucguuuauuugcuu.....................  1     0  S06
............................................................................caagucguuuauuugcuuu....................  2     0  S06
.............................................................................aagucguuuauuugcuuu....................  1     0  S06

....................................cagcaauuaaauggcucgguggc........................................................  1     0  S07
....................................cagcaauuaaauggcucgguggcag......................................................  1     0  S07
```

```
                                    Star                              Mature
aacagcuuuuuaauauccuuuaauccuuuucgaaccagcaauuaaauggcucgguggcagugucucgccugacaccaagucguuuauuugcuuuuagaccgaaggaccaguguu
...................................cagcaauuaaauggcucgguggcagu.....................................................    1     0    S07
....................................agcaauuaaauggcucgguggc........................................................    12    0    S07
....................................agcaauuaaauggcucgguggca.......................................................    2     0    S07
....................................agcaauuaaauggcucgguggcagugucuc................................................    2     0    S07
.......................................................................acaccaagucguuuauuug........................    8     0    S07
.......................................................................acaccaagucguuuauuugc.......................    9     0    S07
.......................................................................acaccaagucguuuauuugcu......................    40    0    S07
.......................................................................acaccaagucguuuauuugcuu.....................    2935  0    S07
.......................................................................acaccaagucguuuauuugcuuu....................    2961  0    S07
.......................................................................acaccaagucguuuauuugcuuuu...................    385   0    S07
.......................................................................acaccaagucguuuauuugcuuuua..................    4     0    S07
.......................................................................acaccaagucguuuauuugcuuuuag.................    1     0    S07
........................................................................caccaagucguuuauuugcuu.....................    2     0    S07
........................................................................caccaagucguuuauuugcuuu....................    3     0    S07
........................................................................caccaagucguuuauuugcuuuu...................    2     0    S07
...........................................................................caagucguuuauuugcuuu....................    1     0    S07

...................................cagcaauuaaauggcucgguggc........................................................    7     0    S05
...................................cagcaauuaaauggcucgguggca.......................................................    15    0    S05
...................................cagcaauuaaauggcucgguggcagu.....................................................    2     0    S05
....................................agcaauuaaauggcucggugg.........................................................    2     0    S05
....................................agcaauuaaauggcucgguggc........................................................    40    0    S05
....................................agcaauuaaauggcucgguggca.......................................................    13    0    S05
....................................agcaauuaaauggcucgguggcagugucuc................................................    3     0    S05
................................................gcucgguggcagugucucgccu............................................    1     0    S05
.................................................cucgguggcagugucucgccu............................................    3     0    S05
.......................................................................acaccaagucguuuauuu.........................    3     0    S05
.......................................................................acaccaagucguuuauuug........................    26    0    S05
.......................................................................acaccaagucguuuauuugc.......................    30    0    S05
.......................................................................acaccaagucguuuauuugcu......................    35    0    S05
.......................................................................acaccaagucguuuauuugcuu.....................    4983  0    S05
.......................................................................acaccaagucguuuauuugcuuu....................    4802  0    S05
.......................................................................acaccaagucguuuauuugcuuuu...................    968   0    S05
.......................................................................acaccaagucguuuauuugcuuuua..................    10    0    S05
........................................................................caccaagucguuuauuugcuu.....................    4     0    S05
........................................................................caccaagucguuuauuugcuuu....................    17    0    S05
........................................................................caccaagucguuuauuugcuuuu...................    26    0    S05
..........................................................................ccaagucguuuauuugcuu.....................    3     0    S05
..........................................................................ccaagucguuuauuugcuuu....................    7     0    S05
..........................................................................ccaagucguuuauuugcuuuu...................    4     0    S05
...........................................................................caagucguuuauuugcuu.....................    4     0    S05
...........................................................................caagucguuuauuugcuuu....................    2     0    S05
............................................................................aagucguuuauuugcuuu....................    2     0    S05

...................................cagcaauuaaauggcucgguggc........................................................    1     0    S10
....................................agcaauuaaauggcucgguggc........................................................    2     0    S10
.......................................................................acaccaagucguuuauuugc.......................    3     0    S10
.......................................................................acaccaagucguuuauuugcu......................    28    0    S10
.......................................................................acaccaagucguuuauuugcuu.....................    873   0    S10
.......................................................................acaccaagucguuuauuugcuuu....................    789   0    S10
.......................................................................acaccaagucguuuauuugcuuuu...................    138   0    S10
........................................................................caccaagucguuuauuugcuuu....................    1     0    S10
```

Provisional ID	:	Scaffold5285_44881
Score total	:	30618.9
Score for star read(s)	:	3.9
Score for read counts	:	30613.1
Score for mfe	:	0.3
Score for randfold	:	1.6
Score for cons. seed	:	
Total read count	:	60058
Mature read count	:	55185
Loop read count	:	0
Star read count	:	4873

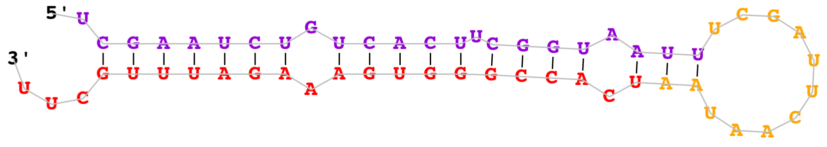

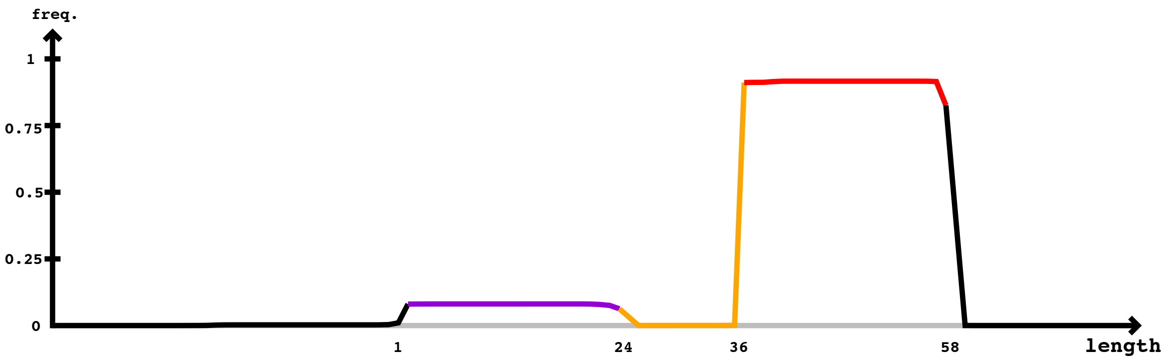

Star Mature

```
5'- ggaaucgugccuguuuuguguauuuuuugauggauucgaaucugucacuucgguaauuucgauucaauaaucaccgggugaaagauuugcuuucauuaagaaaauagaucg -3'   obs
    ggaaucgugccuguuuuguguauuuuuugauggauucgaaucugucacuucgguaauuucgauucaauaaucaccgggugaaagauuugcuuucauuaagaaaauagaucg        exp
    ............(.(((((...((((((((((((..(((((((.(((((.((((.(((..........))).))))))))).)))))))..))))))))))))))))).).   reads   mm   sample
    ..aaucgugccuguuuuguguauuuuuugauggau............................................................................   1       0    S08
    ......gugccuguuuuguguauuuuuugauggau............................................................................   1       0    S08
    ............guuuuguguauuuuuugauggau............................................................................   1       0    S08
    ................uguguauuuuuugauggau............................................................................   1       0    S08
    .................guguauuuuuugauggau............................................................................   1       0    S08
    .................................auucgaaucugucacuucgguaa.......................................................   2       0    S08
    ..................................uucgaaucugucacuucgguaau......................................................   5       0    S08
    ..................................uucgaaucugucacuucgguaauu.....................................................   23      0    S08
    ..................................uucgaaucugucacuucgguaauuu....................................................   2       0    S08
    ..................................uucgaaucugucacuucgguaauuucga.................................................   1       0    S08
    ...................................ucgaaucugucacuucggu.........................................................   5       0    S08
    ...................................ucgaaucugucacuucggua........................................................   22      0    S08
    ...................................ucgaaucugucacuucgguaa.......................................................   47      0    S08
    ...................................ucgaaucugucacuucgguaau......................................................   105     0    S08
    ...................................ucgaaucugucacuucgguaauu.....................................................   356     0    S08
    ...................................ucgaaucugucacuucgguaauuuc...................................................   3       0    S08
    ...................................ucgaaucugucacuucgguaauuucgauuc..............................................   1       0    S08
    ....................................cgaaucugucacuucggua........................................................   2       0    S08
    ....................................cgaaucugucacuucgguaa.......................................................   2       0    S08
    ....................................cgaaucugucacuucgguaauu.....................................................   1       0    S08
    ......................................aaucugucacuucgguaauu.....................................................   3       0    S08
    ........................................ucugucacuucgguaauu.....................................................   1       0    S08
    ..............................................................uucaauaaucaccgggugaaagauuugcuu...................   2       0    S08
    .....................................................................aucaccgggugaaagauuugcuu...................   1       0    S08
    ......................................................................ucaccgggugaaagauuug......................   3       0    S08
    ......................................................................ucaccgggugaaagauuugc.....................   20      0    S08
    ......................................................................ucaccgggugaaagauuugcu....................   1204    0    S08
    ......................................................................ucaccgggugaaagauuugcuu...................   9442    0    S08
    ......................................................................ucaccgggugaaagauuugcuuu..................   433     0    S08
    ......................................................................ucaccgggugaaagauuugcuuuc.................   1       0    S08
    .......................................................................caccgggugaaagauuugcu....................   1       0    S08
    .......................................................................caccgggugaaagauuugcuu...................   12      0    S08
    ........................................................................accgggugaaagauuugcuu...................   5       0    S08
```

Star Mature

ggaaucgugccuguuuuguguauuuuuugauggauucgaaucugucacuucgguaauuucgauucaauaaucaccgggugaaagauuugcuuucauuaagaaaauagaucg			
....ccgggugaaagauuugcu....................	4	0	S08
....ccgggugaaagauuugcuu...................	101	0	S08
....ccgggugaaagauuugcuuu..................	4	0	S08
....cgggugaaagauuugcuu...................	67	0	S08
....cgggugaaagauuugcuuu..................	4	0	S08
................uguguauuuuuugauggau..	1	0	S03
..................................uucgaaucugucacuucgguaauu...	5	0	S03
...................................ucgaaucugucacuucgguaauu...	30	0	S03
....ucaauaaucaccgggugaaagauuugcuu...................	1	0	S03
....ucaccgggugaaagauuugc.....................	3	0	S03
....ucaccgggugaaagauuugcu....................	156	0	S03
....ucaccgggugaaagauuugcuu...................	1488	0	S03
....ucaccgggugaaagauuugcuuu..................	151	0	S03
....caccgggugaaagauuugcuu...................	2	0	S03
....ccgggugaaagauuugcuu...................	1	0	S03
....cgggugaaagauuugcuu...................	3	0	S03
....cgggugaaagauuugcuuu..................	1	0	S03
............guuuuguguauuuuuugauggau..	1	0	S09
..............uuuguguauuuuuugauggau..	2	0	S09
...............uuguguauuuuuugauggau..	38	0	S09
................uguguauuuuuugauggau..	15	0	S09
.................guguauuuuuugauggau..	2	0	S09
................................gauucgaaucugucacuucggu...	1	0	S09
.................................auucgaaucugucacuucggu...	1	0	S09
.................................auucgaaucugucacuucggua..	1	0	S09
.................................auucgaaucugucacuucgguaa...	8	0	S09
.................................auucgaaucugucacuucgguaau..	1	0	S09
.................................auucgaaucugucacuucgguaauu...	3	0	S09
..................................uucgaaucugucacuucggua..	1	0	S09
..................................uucgaaucugucacuucgguaau..	33	0	S09
..................................uucgaaucugucacuucgguaauu...	104	0	S09
...................................ucgaaucugucacuucggu...	3	0	S09
...................................ucgaaucugucacuucggua..	26	0	S09
...................................ucgaaucugucacuucgguaa...	30	0	S09
...................................ucgaaucugucacuucgguaau..	238	0	S09
...................................ucgaaucugucacuucgguaauu...	1200	0	S09
...................................ucgaaucugucacuucgguaauuu..	1	0	S09
...................................ucgaaucugucacuucgguaauuuc...	1	0	S09
...................................ucgaaucugucacuucgguaauuucgauucaauaaucaccgg..................................	1	0	S09
...................................ucgaaucugucacuucgguaauuucgauucaauaaucaccgggug...............................	1	0	S09
....................................cgaaucugucacuucgguaauu...	5	0	S09
......................................aaucugucacuucgguaau..	1	0	S09
......................................aaucugucacuucgguaauu...	2	0	S09
....uucgguaauuucgauucaaua..	2	0	S09
....auuucgauucaauaaucaccgggug...............................	1	0	S09
....ucaauaaucaccgggugaaag...........................	1	0	S09
....aauaaucaccgggugaaagauuugcuu...................	1	0	S09
....aucaccgggugaaagauuugcuu...................	2	0	S09
....ucaccgggugaaagauuugc.....................	12	0	S09
....ucaccgggugaaagauuugcu....................	918	0	S09
....ucaccgggugaaagauuugcuu...................	8925	0	S09
....ucaccgggugaaagauuugcuuu..................	442	0	S09
....ucaccgggugaaagauuugcuuuc.................	4	0	S09
....ucaccgggugaaagauuugcuuuca................	1	0	S09
....caccgggugaaagauuugcuu...................	5	0	S09
....accgggugaaagauuugcuu...................	2	0	S09
....ccgggugaaagauuugcuu...................	4	0	S09
....ccgggugaaagauuugcuuu..................	1	0	S09
....cgggugaaagauuugcuu...................	1	0	S09
..............uuuguguauuuuuugauggau..	1	0	S04
...............uuguguauuuuuugauggau..	1	0	S04
................................gauucgaaucugucacuucggu...	1	0	S04
.................................auucgaaucugucacuucgguaa...	1	0	S04
.................................auucgaaucugucacuucgguaau..	1	0	S04
..................................uucgaaucugucacuucgguaauu...	7	0	S04
...................................ucgaaucugucacuucggu...	2	0	S04
...................................ucgaaucugucacuucgguaa...	2	0	S04

```
                                   Star                                 Mature
ggaaucgugccuguuuuguguauuuuuugauggauucgaaucugucacuucgguaauuucgauucaauaaucaccgggugaaagauuugcuuucauuaagaaaauagaucg
...................................ucgaaucugucacuucgguaau......................................................	8	0	S04
...................................ucgaaucugucacuucgguaauu.....................................................	59	0	S04
....................................................guaauuucgauucaauaaucaccgggug...............................	1	0	S04
.............................................................auucaauaaucaccgggugaaagauuugcuuu..................	1	0	S04
......................................................................ucaccgggugaaagauuugc.....................	2	0	S04
......................................................................ucaccgggugaaagauuugcu....................	136	0	S04
......................................................................ucaccgggugaaagauuugcuu...................	1397	0	S04
......................................................................ucaccgggugaaagauuugcuuu..................	114	0	S04
.......................................................................caccgggugaaagauuugcuu...................	1	0	S04
.........................................................................ccgggugaaagauuugcuu...................	1	0	S04
.........................................................................ccgggugaaagauuugcuuu..................	2	0	S04
..........................................................................cgggugaaagauuugcuu...................	1	0	S04

...................................ucgaaucugucacuucgguaauu.....................................................	2	0	S02
......................................................................ucaccgggugaaagauuugcu....................	1	0	S02
......................................................................ucaccgggugaaagauuugcuu...................	8	0	S02

.........ccuguuuuguguauuuuuugauggau............................................................................	1	0	S10
............guuuuguguauuuuuugauggau............................................................................	1	0	S10
.............uuuuguguauuuuuugauggau............................................................................	4	0	S10
..............uuuguguauuuuuugauggau............................................................................	9	0	S10
...............uuguguauuuuuugauggau............................................................................	47	0	S10
................uguguauuuuuugauggau............................................................................	19	0	S10
.................guguauuuuuugauggau............................................................................	2	0	S10
................................gauucgaaucugucacuucggua........................................................	1	0	S10
.................................auucgaaucugucacuucggua........................................................	1	0	S10
.................................auucgaaucugucacuucgguaa.......................................................	8	0	S10
.................................auucgaaucugucacuucgguaau......................................................	2	0	S10
.................................auucgaaucugucacuucgguaauu.....................................................	4	0	S10
..................................uucgaaucugucacuucgguaa.......................................................	1	0	S10
..................................uucgaaucugucacuucgguaau......................................................	17	0	S10
..................................uucgaaucugucacuucgguaauu.....................................................	111	0	S10
..................................uucgaaucugucacuucgguaauuu....................................................	4	0	S10
...................................ucgaaucugucacuucggu.........................................................	2	0	S10
...................................ucgaaucugucacuucggua........................................................	23	0	S10
...................................ucgaaucugucacuucgguaa.......................................................	26	0	S10
...................................ucgaaucugucacuucgguaau......................................................	208	0	S10
...................................ucgaaucugucacuucgguaauu.....................................................	1127	0	S10
...................................ucgaaucugucacuucgguaauuu....................................................	1	0	S10
....................................cgaaucugucacuucgguaau......................................................	2	0	S10
....................................cgaaucugucacuucgguaauu.....................................................	2	0	S10
........................................ucugucacuucgguaauuucgauucaauaa.........................................	1	0	S10
......................................................................ucaccgggugaaagauuug......................	2	0	S10
......................................................................ucaccgggugaaagauuugc.....................	22	0	S10
......................................................................ucaccgggugaaagauuugcu....................	1573	0	S10
......................................................................ucaccgggugaaagauuugcuu...................	13898	0	S10
......................................................................ucaccgggugaaagauuugcuuu..................	835	0	S10
......................................................................ucaccgggugaaagauuugcuuuc.................	1	0	S10
.......................................................................caccgggugaaagauuugc.....................	1	0	S10
.......................................................................caccgggugaaagauuugcuu...................	7	0	S10
........................................................................accgggugaaagauuugcu....................	1	0	S10
........................................................................accgggugaaagauuugcuu...................	2	0	S10
.........................................................................ccgggugaaagauuugcu....................	1	0	S10
.........................................................................ccgggugaaagauuugcuu...................	3	0	S10
.........................................................................ccgggugaaagauuugcuuu..................	2	0	S10
..........................................................................cgggugaaagauuugcuu...................	1	0	S10

...............uuguguauuuuuugauggau............................................................................	3	0	S05
.................................auucgaaucugucacuucggua........................................................	1	0	S05
.................................auucgaaucugucacuucgguaa.......................................................	3	0	S05
..................................uucgaaucugucacuucgguaau......................................................	2	0	S05
..................................uucgaaucugucacuucgguaauu.....................................................	11	0	S05
...................................ucgaaucugucacuucggu.........................................................	1	0	S05
...................................ucgaaucugucacuucggua........................................................	1	0	S05
...................................ucgaaucugucacuucgguaa.......................................................	2	0	S05
...................................ucgaaucugucacuucgguaau......................................................	4	0	S05
...................................ucgaaucugucacuucgguaauu.....................................................	55	0	S05
...................................ucgaaucugucacuucgguaauuucgau................................................	1	0	S05
....................................cgaaucugucacuucgguaauu.....................................................	1	0	S05
.....................................gaaucugucacuucgguaauu.....................................................	1	0	S05
```

Star **Mature**

```
ggaaucgugccuguuuuguguauuuuuugauggauucgaaucugucacuucgguaauuucgauucaauaaucaccgggugaaagauuugcuuucauuaagaaaauagaucg
......................................................................ucaccgggugaaagauuugc.....................   1      0   S05
......................................................................ucaccgggugaaagauuugcu....................   163    0   S05
......................................................................ucaccgggugaaagauuugcuu...................   1703   0   S05
......................................................................ucaccgggugaaagauuugcuuu..................   162    0   S05
......................................................................ucaccgggugaaagauuugcuuuc.................   1      0   S05
.........................................................................ccgggugaaagauuugcuu...................   7      0   S05
.........................................................................ccgggugaaagauuugcuuu..................   2      0   S05
..........................................................................cgggugaaagauuugcuu...................   3      0   S05
...........................................................................gggugaaagauuugcuuu..................   1      0   S05

.............uuuuguguauuuuuugauggau............................................................................   1      0   S07
................ugugua uuuuuugauggau............................................................................   4      0   S07
................................gauucgaaucugucacuucggu.........................................................   2      0   S07
.................................auucgaaucugucacuucggua........................................................   1      0   S07
.................................auucgaaucugucacuucgguaa.......................................................   8      0   S07
.................................auucgaaucugucacuucgguaau......................................................   2      0   S07
.................................auucgaaucugucacuucgguaauu.....................................................   4      0   S07
..................................uucgaaucugucacuucgguaa.......................................................   5      0   S07
..................................uucgaaucugucacuucgguaau......................................................   11     0   S07
..................................uucgaaucugucacuucgguaauu.....................................................   40     0   S07
...................................ucgaaucugucacuucgg..........................................................   1      0   S07
...................................ucgaaucugucacuucggu.........................................................   3      0   S07
...................................ucgaaucugucacuucggua........................................................   16     0   S07
...................................ucgaaucugucacuucgguaa.......................................................   45     0   S07
...................................ucgaaucugucacuucgguaau......................................................   91     0   S07
...................................ucgaaucugucacuucgguaauu.....................................................   457    0   S07
...................................ucgaaucugucacuucgguaauuu....................................................   1      0   S07
....................................cgaaucugucacuucgguaauu.....................................................   1      0   S07
.....................................gaaucugucacuucgguaauu.....................................................   1      0   S07
....................................................guaauuucgauucaauaaucaccgggug...............................   1      0   S07
...............................................................ucaauaaucaccgggugaaagauuugcuu...................   2      0   S07
................................................................caauaaucaccgggugaaagauuugcuu...................   1      0   S07
.................................................................aauaaucaccgggugaaagauuugcuu...................   1      0   S07
.................................................................aauaaucaccgggugaaagauuugcuuu..................   1      0   S07
....................................................................aaucaccgggugaaagauuugcuu...................   1      0   S07
.....................................................................aucaccgggugaaagauuugcuu...................   1      0   S07
......................................................................ucaccgggugaaagauuug......................   1      0   S07
......................................................................ucaccgggugaaagauuugc.....................   6      0   S07
......................................................................ucaccgggugaaagauuugcu....................   850    0   S07
......................................................................ucaccgggugaaagauuugcuu...................   7504   0   S07
......................................................................ucaccgggugaaagauuugcuuu..................   325    0   S07
.......................................................................caccgggugaaagauuugcuu...................   3      0   S07
........................................................................accgggugaaagauuugcuuu..................   1      0   S07
.........................................................................ccgggugaaagauuugcu....................   3      0   S07
.........................................................................ccgggugaaagauuugcuu...................   11     0   S07
..........................................................................cgggugaaagauuugcuu...................   8      0   S07
..........................................................................cgggugaaagauuugcuuu..................   1      0   S07

.................................auucgaaucugucacuucgguaauu.....................................................   1      0   S06
..................................uucgaaucugucacuucgguaau......................................................   4      0   S06
..................................uucgaaucugucacuucgguaauu.....................................................   13     0   S06
..................................uucgaaucugucacuucgguaauuu....................................................   1      0   S06
...................................ucgaaucugucacuucgg..........................................................   1      0   S06
...................................ucgaaucugucacuucggu.........................................................   2      0   S06
...................................ucgaaucugucacuucggua........................................................   6      0   S06
...................................ucgaaucugucacuucgguaa.......................................................   22     0   S06
...................................ucgaaucugucacuucgguaau......................................................   23     0   S06
...................................ucgaaucugucacuucgguaauu.....................................................   130    0   S06
....................................cgaaucugucacuucgguaauu.....................................................   1      0   S06
.....................................gaaucugucacuucgguaauu.....................................................   3      0   S06
......................................aaucugucacuucgguaau......................................................   1      0   S06
...............................................................ucaauaaucaccgggugaaagauuugcuu...................   1      0   S06
....................................................................aaucaccgggugaaagauuugcuu...................   5      0   S06
......................................................................ucaccgggugaaagauuugc.....................   3      0   S06
......................................................................ucaccgggugaaagauuugcu....................   364    0   S06
......................................................................ucaccgggugaaagauuugcuu...................   2420   0   S06
......................................................................ucaccgggugaaagauuugcuuu..................   174    0   S06
......................................................................ucaccgggugaaagauuugcuuuc.................   1      0   S06
.......................................................................caccgggugaaagauuugcuu...................   3      0   S06
........................................................................accgggugaaagauuugcuuuc.................   1      0   S06
```

```
                                   Star                                  Mature
ggaaucgugccuguuuuguguauuuuuugauggauucgaaucugucacuucgguaauuucgauucaauaaucaccgggugaaagauuugcuuucauuaagaaaauagaucg
.........................................................................ccgggugaaagauuugcu....................   1   0   S06
.........................................................................ccgggugaaagauuugcuu...................   9   0   S06
.........................................................................ccgggugaaagauuugcuuu..................   1   0   S06
..........................................................................cgggugaaagauuugcuu...................   6   0   S06
..........................................................................cgggugaaagauuugcuuu..................   1   0   S06

......................................................................ucaccgggugaaagauuugcu.....................   2   0   S01
```

Provisional ID : Scaffold5285_44874
Score total : 0.6
Score for star read(s) : -1.3
Score for read counts : 0
Score for mfe : 0.3
Score for randfold : 1.6
Score for cons. seed :
Total read count : 15757
Mature read count : 7500
Loop read count : 0
Star read count : 8257

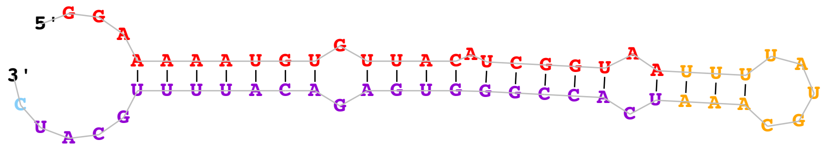

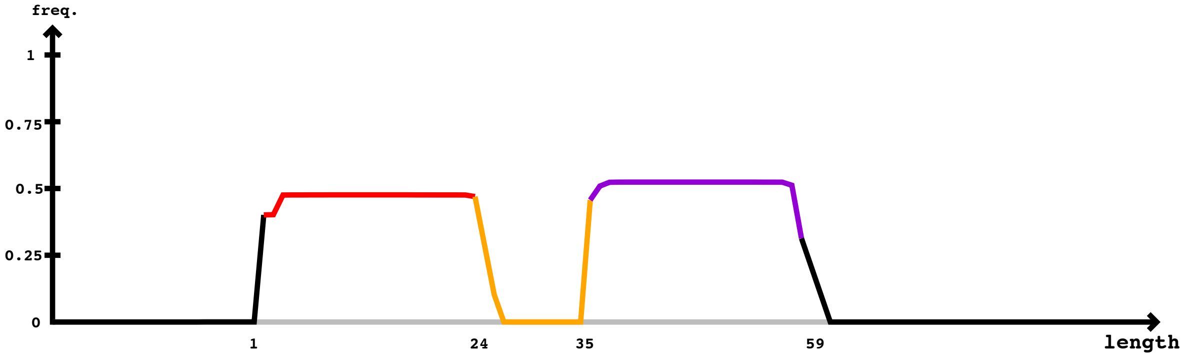

Mature Star

```
5'- auuuuuucaaguucugcggaggaaaaaauguguuacaucgguaauuuuaugcaaaucaccgggugagacauuuugcauccguuggauagauaacagcagcuucuucuugaucac -3'  obs
    auuuuuucaaguucugcggaggaaaaaauguguuacaucgguaauuuuaugcaaaucaccgggugagacauuuugcauccguuggauagauaacagcagcuucuucuugaucac       exp
    ..........((((.(((((...(((((((.((((.(((((.((((.....)))).))))))))).)))))))...))))).))))............................  reads  mm  sample
    ....................ggaaaaaauguguuacaucgguaa......................................................................  191    0   S04
    ....................ggaaaaaauguguuacaucgguaauu....................................................................  11     0   S04
    .....................gaaaaaauguguuacaucgguaau.....................................................................  1      0   S04
    ......................aaaaaauguguuacaucgguaau.....................................................................  2      0   S04
    ......................aaaaaauguguuacaucgguaauu....................................................................  37     0   S04
    .......................aaaaauguguuacaucgguaauu....................................................................  1      0   S04
    .......................................................ucaccgggugagacauuuugc......................................  3      0   S04
    .......................................................ucaccgggugagacauuuugca.....................................  101    0   S04
    .......................................................ucaccgggugagacauuuugcau....................................  108    0   S04
    ........................................................caccgggugagacauuuugcau....................................  19     0   S04
    ........................................................caccgggugagacauuuugcauc...................................  3      0   S04
    .........................................................accgggugagacauuuugcau....................................  6      0   S04
    .........................................................accgggugagacauuuugcauc...................................  5      0   S04

    ....................ggaaaaaauguguuacaucgguaa......................................................................  10     0   S02
    ......................aaaaaauguguuacaucgguaauu....................................................................  1      0   S02
    .......................................................ucaccgggugagacauuuugca.....................................  2      0   S02

    ....................ggaaaaaauguguuacaucggua.......................................................................  5      0   S08
    ....................ggaaaaaauguguuacaucgguaa......................................................................  631    0   S08
    ....................ggaaaaaauguguuacaucgguaau.....................................................................  20     0   S08
    ....................ggaaaaaauguguuacaucgguaauu....................................................................  75     0   S08
    .....................gaaaaaauguguuacaucgguaa......................................................................  2      0   S08
    .....................gaaaaaauguguuacaucgguaau.....................................................................  1      0   S08
    ......................aaaaaauguguuacaucgguaa......................................................................  7      0   S08
    ......................aaaaaauguguuacaucgguaau.....................................................................  4      0   S08
    ......................aaaaaauguguuacaucgguaauu....................................................................  110    0   S08
    .......................aaaaauguguuacaucgguaauu....................................................................  1      0   S08
    ..........................aauguguuacaucgguaau.....................................................................  2      0   S08
    ...........................auguguuacaucgguaauu....................................................................  1      0   S08
    ............................uguguuacaucgguaauu....................................................................  1      0   S08
    .......................................................ucaccgggugagacauuuugc......................................  55     0   S08
    .......................................................ucaccgggugagacauuuugca.....................................  1223   0   S08
```

Mature Star

auuuuuucaaguucugcggaggaaaaauguguuacaucgguaauuuuaugcaaaucaccgggugagacauuuugcauccguuggauagauaacagcagcuucuucuugaucac			
....ucaccgggugagacauuuugcau....................................	1608	0	S08
....ucaccgggugagacauuuugcauc...................................	2	0	S08
....caccgggugagacauuuugc......................................	2	0	S08
....caccgggugagacauuuugca.....................................	2	0	S08
....caccgggugagacauuuugcau....................................	334	0	S08
....caccgggugagacauuuugcauc...................................	20	0	S08
....accgggugagacauuuugcau....................................	23	0	S08
....accgggugagacauuuugcauc...................................	53	0	S08
....ccgggugagacauuuugca.....................................	6	0	S08
....ccgggugagacauuuugcau....................................	1	0	S08
..............ugcggaggaaaaauguguuaca...	1	0	S03
....................ggaaaaauguguuacaucgguaa..	233	0	S03
....................ggaaaaauguguuacaucgguaau...	1	0	S03
....................ggaaaaauguguuacaucgguaauu..	1	0	S03
.....................gaaaaauguguuacaucgguaa..	1	0	S03
......................aaaaauguguuacaucgguaauu..	32	0	S03
....ucaccgggugagacauuuugc......................................	1	0	S03
....ucaccgggugagacauuuugca.....................................	60	0	S03
....ucaccgggugagacauuuugcau....................................	56	0	S03
....caccgggugagacauuuugcau....................................	15	0	S03
....caccgggugagacauuuugcauc...................................	2	0	S03
....accgggugagacauuuugcau....................................	3	0	S03
....accgggugagacauuuugcauc...................................	2	0	S03
....................ggaaaaauguguuacaucggu..	2	0	S09
....................ggaaaaauguguuacaucggua...	30	0	S09
....................ggaaaaauguguuacaucgguaa..	1013	0	S09
....................ggaaaaauguguuacaucgguaau...	67	0	S09
....................ggaaaaauguguuacaucgguaauu..	187	0	S09
....................ggaaaaauguguuacaucgguaauuu...	1	0	S09
.....................gaaaaauguguuacaucgguaa..	5	0	S09
.....................gaaaaauguguuacaucgguaau...	1	0	S09
......................aaaaauguguuacaucggua...	1	0	S09
......................aaaaauguguuacaucgguaa..	10	0	S09
......................aaaaauguguuacaucgguaau...	18	0	S09
......................aaaaauguguuacaucgguaauu..	365	0	S09
......................aaaaauguguuacaucgguaauuu...	1	0	S09
.......................aaaauguguuacaucgguaauu..	1	0	S09
....ucaccgggugagacauuu...	1	0	S09
....ucaccgggugagacauuuugc......................................	51	0	S09
....ucaccgggugagacauuuugca.....................................	599	0	S09
....ucaccgggugagacauuuugcau....................................	583	0	S09
....caccgggugagacauuuugca.....................................	1	0	S09
....caccgggugagacauuuugcau....................................	134	0	S09
....caccgggugagacauuuugcauc...................................	9	0	S09
....accgggugagacauuuugcau....................................	5	0	S09
....accgggugagacauuuugcauc...................................	36	0	S09
...................aggaaaaauguguuacaucgguaa..	1	0	S07
....................ggaaaaauguguuacaucggua...	8	0	S07
....................ggaaaaauguguuacaucgguaa..	1020	0	S07
....................ggaaaaauguguuacaucgguaau...	21	0	S07
....................ggaaaaauguguuacaucgguaauu..	76	0	S07
......................aaaaauguguuacaucggua...	1	0	S07
......................aaaaauguguuacaucgguaa..	2	0	S07
......................aaaaauguguuacaucgguaau...	7	0	S07
......................aaaaauguguuacaucgguaauu..	123	0	S07
.........................aauguguuacaucgguaauu..	1	0	S07
....ucaccgggugagacauuu...	2	0	S07
....ucaccgggugagacauuuugc......................................	17	0	S07
....ucaccgggugagacauuuugca.....................................	369	0	S07
....ucaccgggugagacauuuugcau....................................	605	0	S07
....ucaccgggugagacauuuugcauc...................................	1	0	S07
....caccgggugagacauuuugca.....................................	1	0	S07
....caccgggugagacauuuugcau....................................	96	0	S07
....caccgggugagacauuuugcauc...................................	8	0	S07
....accgggugagacauuuugcau....................................	8	0	S07
....accgggugagacauuuugcauc...................................	24	0	S07
....ccgggugagacauuuugcau....................................	1	0	S07

```
                    Mature                             Star
auuuuuucaaguucugcggaggaaaaaauguguuacaucgguaauuuuaugcaaaucaccgggugagacauuuugcauccguuggauagauaacagcagcuucuucuugaucac
...........................................................cgggugagacauuuugca.....................................	1	0	S07

.......................................................ucaccgggugagacauuuugca.....................................	1	0	S01

....................ggaaaaaauguguuacaucggua.......................................................................	2	0	S06
....................ggaaaaaauguguuacaucgguaa......................................................................	533	0	S06
....................ggaaaaaauguguuacaucgguaau.....................................................................	16	0	S06
....................ggaaaaaauguguuacaucgguaauu....................................................................	34	0	S06
.....................gaaaaaauguguuacaucgguaa......................................................................	1	0	S06
.....................gaaaaaauguguuacaucgguaau.....................................................................	1	0	S06
......................aaaaaauguguuacaucgguaa......................................................................	3	0	S06
......................aaaaaauguguuacaucgguaauu....................................................................	43	0	S06
.......................................................ucaccgggugagacauuuug.......................................	1	0	S06
.......................................................ucaccgggugagacauuuugc......................................	11	0	S06
.......................................................ucaccgggugagacauuuugca.....................................	208	0	S06
.......................................................ucaccgggugagacauuuugcau....................................	350	0	S06
........................................................caccgggugagacauuuugcau....................................	49	0	S06
........................................................caccgggugagacauuuugcauc...................................	2	0	S06
.........................................................accgggugagacauuuugca.....................................	1	0	S06
.........................................................accgggugagacauuuugcau....................................	6	0	S06
.........................................................accgggugagacauuuugcauc...................................	7	0	S06
...........................................................cgggugagacauuuugcau....................................	1	0	S06

..............ugcggaggaaaaaauguguuaca.............................................................................	3	0	S10
....................ggaaaaaauguguuacauc...........................................................................	1	0	S10
....................ggaaaaaauguguuacaucggu........................................................................	1	0	S10
....................ggaaaaaauguguuacaucggua.......................................................................	46	0	S10
....................ggaaaaaauguguuacaucgguaa......................................................................	1646	0	S10
....................ggaaaaaauguguuacaucgguaau.....................................................................	66	0	S10
....................ggaaaaaauguguuacaucgguaauu....................................................................	131	0	S10
....................ggaaaaaauguguuacaucgguaauuu...................................................................	2	0	S10
.....................gaaaaaauguguuacaucgguaa......................................................................	1	0	S10
.....................gaaaaaauguguuacaucgguaau.....................................................................	1	0	S10
.....................gaaaaaauguguuacaucgguaauu....................................................................	1	0	S10
......................aaaaaauguguuacaucggua.......................................................................	1	0	S10
......................aaaaaauguguuacaucgguaa......................................................................	3	0	S10
......................aaaaaauguguuacaucgguaau.....................................................................	16	0	S10
......................aaaaaauguguuacaucgguaauu....................................................................	336	0	S10
......................aaaaaauguguuacaucgguaauuu...................................................................	1	0	S10
.......................aaaaauguguuacaucgguaauu....................................................................	1	0	S10
.......................................................ucaccgggugagacauuuugc......................................	39	0	S10
.......................................................ucaccgggugagacauuuugca.....................................	428	0	S10
.......................................................ucaccgggugagacauuuugcau....................................	405	0	S10
.......................................................ucaccgggugagacauuuugcauc...................................	2	0	S10
........................................................caccgggugagacauuuugc......................................	1	0	S10
........................................................caccgggugagacauuuugca.....................................	1	0	S10
........................................................caccgggugagacauuuugcau....................................	83	0	S10
........................................................caccgggugagacauuuugcauc...................................	4	0	S10
.........................................................accgggugagacauuuugcau....................................	9	0	S10
.........................................................accgggugagacauuuugcauc...................................	16	0	S10

....................ggaaaaaauguguuacaucgguaa......................................................................	226	0	S05
....................ggaaaaaauguguuacaucgguaau.....................................................................	1	0	S05
....................ggaaaaaauguguuacaucgguaauu....................................................................	7	0	S05
......................aaaaaauguguuacaucgguaa......................................................................	2	0	S05
......................aaaaaauguguuacaucgguaau.....................................................................	1	0	S05
......................aaaaaauguguuacaucgguaauu....................................................................	32	0	S05
.......................................................ucaccgggugagacauuuugc......................................	2	0	S05
.......................................................ucaccgggugagacauuuugca.....................................	132	0	S05
.......................................................ucaccgggugagacauuuugcau....................................	175	0	S05
........................................................caccgggugagacauuuugcau....................................	31	0	S05
........................................................caccgggugagacauuuugcauc...................................	5	0	S05
.........................................................accgggugagacauuuugcau....................................	10	0	S05
.........................................................accgggugagacauuuugcauc...................................	9	0	S05
..........................................................ccgggugagacauuuugcau....................................	1	0	S05
```

Provisional ID	:	Scaffold6623_45528
Score total	:	29641.6
Score for star read(s)	:	3.9
Score for read counts	:	29635.2
Score for mfe	:	0.9
Score for randfold	:	1.6
Score for cons. seed	:	
Total read count	:	58140
Mature read count	:	57801
Loop read count	:	0
Star read count	:	339

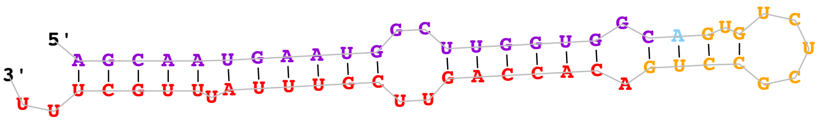

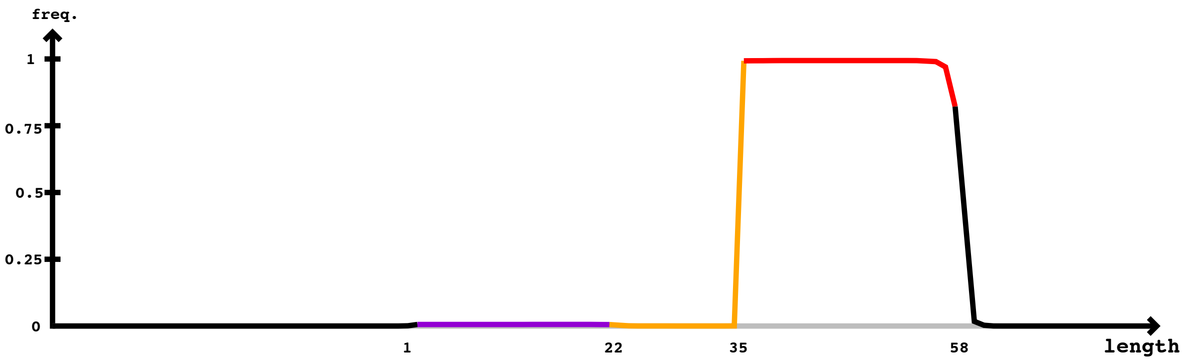

Star **Mature**

```
5'- aucgguguuuuaagaucauuuaauucuucuugaaccagcaaugaauggcuugguggcagugucucgccugacaccaguucguuuauuugcuuuuaaaccgaaguucgauccuc   -3'   obs
    aucgguguuuuaagaucauuuaauucuucuugaaccagcaaugaauggcuugguggcagugucucgccugacaccaguucguuuauuugcuuuuaaaccgaaguucgauccuc         exp
    .(((((..(((((((............)))))))..(((((((((((..((((((.(((.(.....)))).))))))..)))))).))))).....)))))............   reads   mm   sample
    ...................................cagcaaugaauggcuuggug..........................................................   2       0    S05
    ...................................cagcaaugaauggcuugguggca.......................................................   2       0    S05
    ....................................agcaaugaauggcuugguggc........................................................   18      0    S05
    ....................................agcaaugaauggcuugguggca.......................................................   2       0    S05
    ....................................agcaaugaauggcuugguggcag......................................................   4       0    S05
    ....................................agcaaugaauggcuugguggcagu.....................................................   1       0    S05
    ....................................................................gacaccaguucguuuauuugcu.......................   3       0    S05
    ....................................................................gacaccaguucguuuauuugcuu......................   3       0    S05
    .....................................................................acaccaguucguuuauuug.........................   12      0    S05
    .....................................................................acaccaguucguuuauuugc........................   7       0    S05
    .....................................................................acaccaguucguuuauuugcu.......................   96      0    S05
    .....................................................................acaccaguucguuuauuugcuu......................   620     0    S05
    .....................................................................acaccaguucguuuauuugcuuu.....................   1685    0    S05
    .....................................................................acaccaguucguuuauuugcuuuu....................   567     0    S05
    .....................................................................acaccaguucguuuauuugcuuuua...................   35      0    S05
    .....................................................................acaccaguucguuuauuugcuuuuaa..................   3       0    S05
    ......................................................................caccaguucguuuauuugcuuu.....................   2       0    S05
    ........................................................................ccaguucguuuauuugcuuu.....................   3       0    S05
    ..........................................................................aguucguuuauuugcuuu.....................   1       0    S05

    ....................................agcaaugaauggcuuggugg.........................................................   1       0    S10
    ....................................agcaaugaauggcuugguggc........................................................   1       0    S10
    .....................................................................acaccaguucguuuauuug.........................   4       0    S10
    .....................................................................acaccaguucguuuauuugc........................   2       0    S10
    .....................................................................acaccaguucguuuauuugcu.......................   18      0    S10
    .....................................................................acaccaguucguuuauuugcuu......................   112     0    S10
    .....................................................................acaccaguucguuuauuugcuuu.....................   211     0    S10
    .....................................................................acaccaguucguuuauuugcuuuu....................   56      0    S10
    .....................................................................acaccaguucguuuauuugcuuuua...................   2       0    S10

    ...................................cagcaaugaauggcuuggugg.........................................................   2       0    S06
    ....................................agcaaugaauggcuuggug..........................................................   1       0    S06
    ....................................agcaaugaauggcuugguggc........................................................   13      0    S06
```

```
                                     Star                              Mature
aucgguguuuuaagaucauuuaauucuucuugaaccagcaaugaauggcuugguggcagugucucgccugacaccaguucguuuauuugcuuuuaaaccgaaguucgauccuc
....................................agcaaugaauggcuugguggca.......................................................	3	0	S06
....................................agcaaugaauggcuugguggcag......................................................	6	0	S06
......................................................................acaccaguucguuuauuug........................	4	0	S06
......................................................................acaccaguucguuuauuugc.......................	9	0	S06
......................................................................acaccaguucguuuauuugcu......................	114	0	S06
......................................................................acaccaguucguuuauuugcuu.....................	754	0	S06
......................................................................acaccaguucguuuauuugcuuu....................	1586	0	S06
......................................................................acaccaguucguuuauuugcuuuu...................	1083	0	S06
......................................................................acaccaguucguuuauuugcuuuua..................	53	0	S06
......................................................................acaccaguucguuuauuugcuuuuaa.................	5	0	S06
.......................................................................caccaguucguuuauuugcuu.....................	3	0	S06
.......................................................................caccaguucguuuauuugcuuuu...................	1	0	S06
.........................................................................ccaguucguuuauuugcuuu....................	1	0	S06
.........................................................................ccaguucguuuauuugcuuuu...................	1	0	S06
..........................................................................caguucguuuauuugcuuu....................	1	0	S06

...................................cagcaaugaauggcuuggug..........................................................	1	0	S01
...................................cagcaaugaauggcuugguggc........................................................	3	0	S01
...................................cagcaaugaauggcuugguggca.......................................................	2	0	S01
....................................agcaaugaauggcuuggug..........................................................	2	0	S01
....................................agcaaugaauggcuugguggc........................................................	38	0	S01
....................................agcaaugaauggcuugguggca.......................................................	3	0	S01
....................................agcaaugaauggcuugguggcag......................................................	5	0	S01
....................................agcaaugaauggcuugguggcaguguc..................................................	2	0	S01
....................................agcaaugaauggcuugguggcagugucuc...............................................	1	0	S01
.....................................gcaaugaauggcuugguggca.......................................................	2	0	S01
.......................................aaugaauggcuugguggc........................................................	1	0	S01
................................................cuugguggcagugucucgccu............................................	2	0	S01
.....................................................................gacaccaguucguuuauuugcu......................	1	0	S01
.....................................................................gacaccaguucguuuauuugcuu.....................	2	0	S01
......................................................................acaccaguucguuuauuu.........................	2	0	S01
......................................................................acaccaguucguuuauuug........................	16	0	S01
......................................................................acaccaguucguuuauuugc.......................	26	0	S01
......................................................................acaccaguucguuuauuugcu......................	227	0	S01
......................................................................acaccaguucguuuauuugcuu.....................	1629	0	S01
......................................................................acaccaguucguuuauuugcuuu....................	7375	0	S01
......................................................................acaccaguucguuuauuugcuuuu...................	4706	0	S01
......................................................................acaccaguucguuuauuugcuuuua..................	176	0	S01
......................................................................acaccaguucguuuauuugcuuuuaa.................	46	0	S01
......................................................................acaccaguucguuuauuugcuuuuaaa................	1	0	S01
.......................................................................caccaguucguuuauuugcuuu....................	2	0	S01
.......................................................................caccaguucguuuauuugcuuuu...................	4	0	S01
........................................................................accaguucguuuauuugcu......................	1	0	S01
.........................................................................ccaguucguuuauuugcuuu....................	4	0	S01
.........................................................................ccaguucguuuauuugcuuuu...................	2	0	S01
..........................................................................caguucguuuauuugcuuuu...................	2	0	S01
...........................................................................aguucguuuauuugcuuu....................	1	0	S01

....................................agcaaugaauggcuuggug..........................................................	2	0	S07
....................................agcaaugaauggcuuggugg.........................................................	1	0	S07
....................................agcaaugaauggcuugguggc........................................................	20	0	S07
....................................agcaaugaauggcuugguggca.......................................................	7	0	S07
....................................agcaaugaauggcuugguggcag......................................................	1	0	S07
.....................................gcaaugaauggcuugguggca.......................................................	1	0	S07
.....................................................................gacaccaguucguuuauuugcuu.....................	1	0	S07
......................................................................acaccaguucguuuauuugcu......................	14	0	S07
......................................................................acaccaguucguuuauuugcuu.....................	207	0	S07
......................................................................acaccaguucguuuauuugcuuu....................	444	0	S07
......................................................................acaccaguucguuuauuugcuuuu...................	92	0	S07
......................................................................acaccaguucguuuauuugcuuuua..................	7	0	S07
......................................................................acaccaguucguuuauuugcuuuuaa.................	1	0	S07

...................................cagcaaugaauggcuuggug..........................................................	1	0	S09
...................................cagcaaugaauggcuugguggc........................................................	1	0	S09
......................................................................acaccaguucguuuauuu.........................	1	0	S09
......................................................................acaccaguucguuuauuug........................	2	0	S09
......................................................................acaccaguucguuuauuugcu......................	9	0	S09
......................................................................acaccaguucguuuauuugcuu.....................	61	0	S09
......................................................................acaccaguucguuuauuugcuuu....................	105	0	S09
......................................................................acaccaguucguuuauuugcuuuu...................	20	0	S09
```

```
                                    Star                              Mature
aucgguguuuuaagaucauuuaauucuucuugaaccagcaaugaauggcuugguggcagugucucgccugacaccaguucguuuauuugcuuuuaaaccgaaguucgauccuc

...................................cagcaaugaauggcuuggug..........................................................   1      0      S03
...................................cagcaaugaauggcuugguggg.........................................................   1      0      S03
...................................cagcaaugaauggcuugguggc........................................................   2      0      S03
...................................cagcaaugaauggcuugguggca.......................................................   3      0      S03
....................................agcaaugaauggcuugguggg.........................................................   1      0      S03
....................................agcaaugaauggcuugguggc........................................................   25     0      S03
....................................agcaaugaauggcuugguggca.......................................................   7      0      S03
....................................agcaaugaauggcuugguggcag......................................................   4      0      S03
.....................................gcaaugaauggcuugguggca.......................................................   1      0      S03
.....................................................................gacaccaguucguuuauuugcuu.....................   2      0      S03
.....................................................................gacaccaguucguuuauuugcuuu....................   5      0      S03
......................................................................acaccaguucguuuauuu.........................   5      0      S03
......................................................................acaccaguucguuuauuug........................   14     0      S03
......................................................................acaccaguucguuuauuugc.......................   17     0      S03
......................................................................acaccaguucguuuauuugcu......................   267    0      S03
......................................................................acaccaguucguuuauuugcuu.....................   2030   0      S03
......................................................................acaccaguucguuuauuugcuuu....................   6326   0      S03
......................................................................acaccaguucguuuauuugcuuuu...................   2158   0      S03
......................................................................acaccaguucguuuauuugcuuuua..................   105    0      S03
......................................................................acaccaguucguuuauuugcuuuuaa.................   9      0      S03
........................................................................accaguucguuuauuugcuuu....................   1      0      S03
.........................................................................ccaguucguuuauuugcuuu....................   2      0      S03
..........................................................................caguucguuCauuugcuu.....................   1      1      S03

...................................cagcaaugaauggcuuggug..........................................................   1      0      S08
...................................cagcaaugaauggcuugguggc........................................................   3      0      S08
...................................cagcaaugaauggcuugguggca.......................................................   1      0      S08
....................................agcaaugaauggcuuggug..........................................................   1      0      S08
....................................agcaaugaauggcuugguggc........................................................   4      0      S08
....................................agcaaugaauggcuugguggca.......................................................   3      0      S08
....................................agcaaugaauggcuugguggcag......................................................   1      0      S08
......................................................................acaccaguucguuuauuu.........................   1      0      S08
......................................................................acaccaguucguuuauuugc.......................   4      0      S08
......................................................................acaccaguucguuuauuugcu......................   4      0      S08
......................................................................acaccaguucguuuauuugcuu.....................   73     0      S08
......................................................................acaccaguucguuuauuugcuuu....................   172    0      S08
......................................................................acaccaguucguuuauuugcuuuu...................   34     0      S08
......................................................................acaccaguucguuuauuugcuuuuaa.................   1      0      S08

...................................cagcaaugaauggcuugg............................................................   1      0      S02
...................................cagcaaugaauggcuuggug..........................................................   1      0      S02
...................................cagcaaugaauggcuugguggc........................................................   5      0      S02
...................................cagcaaugaauggcuugguggca.......................................................   7      0      S02
....................................agcaaugaauggcuuggug..........................................................   3      0      S02
....................................agcaaugaauggcuugguggg.........................................................   1      0      S02
....................................agcaaugaauggcuugguggc........................................................   42     0      S02
....................................agcaaugaauggcuugguggca.......................................................   10     0      S02
....................................agcaaugaauggcuugguggcag......................................................   8      0      S02
................................................cuugguggcagugucucgccu............................................   4      0      S02
......................................................................acaccaguucguuuauuu.........................   2      0      S02
......................................................................acaccaguucguuuauuug........................   40     0      S02
......................................................................acaccaguucguuuauuugc.......................   32     0      S02
......................................................................acaccaguucguuuauuugcu......................   255    0      S02
......................................................................acaccaguucguuuauuugcuu.....................   2003   0      S02
......................................................................acaccaguucguuuauuugcuuu....................   8397   0      S02
......................................................................acaccaguucguuuauuugcuuuu...................   7536   0      S02
......................................................................acaccaguucguuuauuugcuuuua..................   408    0      S02
......................................................................acaccaguucguuuauuugcuuuuaa.................   90     0      S02
......................................................................acaccaguucguuuauuugcuuuuaaa................   7      0      S02
.......................................................................caccaguucguuuauuugcuuu....................   2      0      S02
.......................................................................caccaguucguuuauuugcuuuu...................   4      0      S02
........................................................................accaguucguuuauuugcuuu....................   1      0      S02
........................................................................accaguucguuuauuugcuuuu...................   2      0      S02
.........................................................................ccaguucguuuauuugcuuu....................   5      0      S02
.........................................................................ccaguucguuuauuugcuuuu...................   5      0      S02
..........................................................................caguucguuuauuugcuuu....................   1      0      S02
..........................................................................caguucguuuauuugcuuuu...................   1      0      S02
...........................................................................aguucguuuauuugcuuuu...................   1      0      S02

...................................cagcaaugaauggcuuggug..........................................................   5      0      S04
```

Star Mature

```
aucgguguuuuaagaucauuuaauucuucuugaaccagcaaugaauggcuugguggcagugucucgccugacaccaguucguuuauuugcuuuuaaaccgaaguucgauccuc
...................................cagcaaugaauggcuuggugg.........................................................    1      0    S04
...................................cagcaaugaauggcuugguggc........................................................    1      0    S04
...................................cagcaaugaauggcuugguggca.......................................................    1      0    S04
....................................agcaaugaauggcuuggugg.........................................................    2      0    S04
....................................agcaaugaauggcuugguggc........................................................    30     0    S04
....................................agcaaugaauggcuugguggca.......................................................    10     0    S04
....................................agcaaugaauggcuugguggcag......................................................    3      0    S04
................................................cuugguggcagugucucgccu............................................    1      0    S04
.....................................................................gacaccaguucguuuauuugcuu.....................    1      0    S04
.....................................................................gacaccaguucguuuauuugcuuu....................    1      0    S04
......................................................................acaccaguucguuuauuug........................    18     0    S04
......................................................................acaccaguucguuuauuugc.......................    13     0    S04
......................................................................acaccaguucguuuauuugcu......................    159    0    S04
......................................................................acaccaguucguuuauuugcuu.....................    1114   0    S04
......................................................................acaccaguucguuuauuugcuuu....................    2942   0    S04
......................................................................acaccaguucguuuauuugcuuuu...................    1216   0    S04
......................................................................acaccaguucguuuauuugcuuuua..................    56     0    S04
......................................................................acaccaguucguuuauuugcuuuuaa.................    8      0    S04
.......................................................................caccaguucguuuauuugcuuuu...................    1      0    S04
........................................................................accaguucguuuauuugcuuu....................    1      0    S04
.........................................................................ccaguucguuuauuugcuuu....................    2      0    S04
..........................................................................caguucguuCauuugcuu.....................    1      1    S04
...........................................................................aguucguuuauuugcuuu....................    1      0    S04
```

Provisional ID	:	Scaffold6623_45530
Score total	:	186070.8
Score for star read(s)	:	3.9
Score for read counts	:	186064.4
Score for mfe	:	1
Score for randfold	:	1.6
Score for cons. seed	:	
Total read count	:	364969
Mature read count	:	364208
Loop read count	:	0
Star read count	:	761

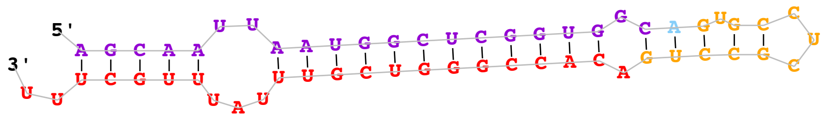

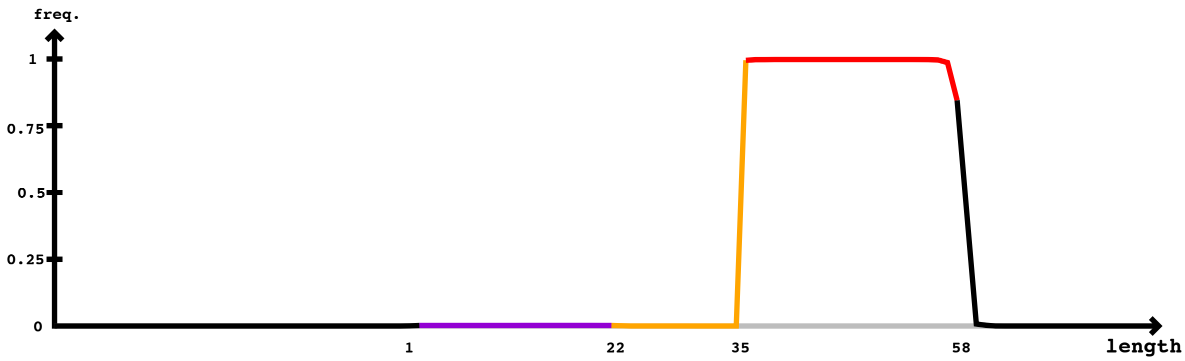

Star Mature

```
5'- aacagcguuuuaagauaauuuaauacuuuucaagccagcaauuaauggcucgguggcagugccucgccugacaccgggucguuuauuugcuuuuaaaccgaagggccagaguu  -3'  obs
    aacagcguuuuaagauaauuuaauacuuuucaagccagcaauuaauggcucgguggcagugccucgccugacaccgggucguuuauuugcuuuuaaaccgaagggccagaguu       exp
    ........................(((((.......(((((..((((((((((((.(((.((...))))).))))))))))))...)))))......((....))..))))).   reads   mm   sample
    ...................................cagcaauuaauggcucgguggc........................................................   18      0    S01
    ...................................cagcaauuaauggcucgguggca.......................................................   37      0    S01
    ...................................cagcaauuaauggcucgguggcag......................................................   3       0    S01
    ...................................cagcaauuaauggcucgguggcagu.....................................................   1       0    S01
    ...................................cagcaauuaauggcucgguggcagugccu.................................................   1       0    S01
    ....................................agcaauuaauggcucgguggc........................................................   55      0    S01
    ....................................agcaauuaauggcucgguggca.......................................................   48      0    S01
    ....................................agcaauuaauggcucgguggcag......................................................   1       0    S01
    ....................................agcaauuaauggcucgguggcagugccuc................................................   5       0    S01
    ....................................agcaauuaauggcucgguggcagugccucgc..............................................   1       0    S01
    .....................................................................gacaccgggucguuuauuugcuu.....................   1       0    S01
    .....................................................................gacaccgggucguuuauuugcuuu....................   2       0    S01
    ......................................................................acaccgggucguuuauuu.........................   8       0    S01
    ......................................................................acaccgggucguuuauuug........................   8       0    S01
    ......................................................................acaccgggucguuuauuugc.......................   65      0    S01
    ......................................................................acaccgggucguuuauuugcu......................   626     0    S01
    ......................................................................acaccgggucguuuauuugcuu.....................   9440    0    S01
    ......................................................................acaccgggucguuuauuugcuuu....................   64083   0    S01
    ......................................................................acaccgggucguuuauuugcuuuu...................   10974   0    S01
    ......................................................................acaccgggucguuuauuugcuuuua..................   319     0    S01
    ......................................................................acaccgggucguuuauuugcuuuuaa.................   235     0    S01
    ......................................................................acaccgggucguuuauuugcuuuuaaa................   3       0    S01
    .......................................................................caccgggucguuuauuugcu......................   1       0    S01
    .......................................................................caccgggucguuuauuugcuu.....................   4       0    S01
    .......................................................................caccgggucguuuauuugcuuu....................   82      0    S01
    .......................................................................caccgggucguuuauuugcuuuu...................   118     0    S01
    .......................................................................caccgggucguuuauuugcuuuua..................   4       0    S01
    ........................................................................accgggucguuuauuugcuu.....................   3       0    S01
    ........................................................................accgggucguuuauuugcuuu....................   8       0    S01
    ........................................................................accgggucguuuauuugcuuuu...................   2       0    S01
    .........................................................................ccgggucguuuauuugcu......................   1       0    S01
    .........................................................................ccgggucguuuauuugcuu.....................   1       0    S01
    .........................................................................ccgggucguuuauuugcuuu....................   18      0    S01
```

Star Mature

```
aacagcguuuuaagauaauuuaauacuuuucaagccagcaauuaauggcucgguggcagugccucgccugacaccgggucguuuauuugcuuuuaaaccgaagggccagaguu
..........................................................................cgggucguuuauuugcuu.....................  2      0  S01
..........................................................................cgggucguuuauuugcuuuua..................  1      0  S01
............................................................................ggucguuuauuugcuuuu...................  4      0  S01

...................................cagcaauuaauggcucgguggc........................................................  3      0  S06
...................................cagcaauuaauggcucgguggca.......................................................  11     0  S06
....................................agcaauuaauggcucgguggc........................................................  21     0  S06
....................................agcaauuaauggcucgguggca.......................................................  13     0  S06
....................................agcaauuaauggcucgguggcagugccuc................................................  1      0  S06
......................................................................acaccgggucguuuauuu.........................  9      0  S06
......................................................................acaccgggucguuuauuug........................  7      0  S06
......................................................................acaccgggucguuuauuugc.......................  47     0  S06
......................................................................acaccgggucguuuauuugcu......................  535    0  S06
......................................................................acaccgggucguuuauuugcuu.....................  6973   0  S06
......................................................................acaccgggucguuuauuugcuuu....................  23822  0  S06
......................................................................acaccgggucguuuauuugcuuuu...................  4701   0  S06
......................................................................acaccgggucguuuauuugcuuuua..................  101    0  S06
......................................................................acaccgggucguuuauuugcuuuuaa.................  28     0  S06
.......................................................................caccgggucguuuauuugcu......................  1      0  S06
.......................................................................caccgggucguuuauuugcuu.....................  7      0  S06
.......................................................................caccgggucguuuauuugcuuu....................  35     0  S06
.......................................................................caccgggucguuuauuugcuuuu...................  34     0  S06
........................................................................accgggucguuuauuugcuu.....................  1      0  S06
........................................................................accgggucguuuauuugcuuu....................  2      0  S06
........................................................................accgggucguuuauuugcuuuu...................  3      0  S06
.........................................................................ccgggucguuuauuugcu......................  1      0  S06
.........................................................................ccgggucguuuauuugcuuu....................  9      0  S06
.........................................................................ccgggucguuuauuugcuuuu...................  2      0  S06
.........................................................................ccgggucguuuauuugcuuuua..................  1      0  S06
..........................................................................cgggucguuuauuugcuuu....................  2      0  S06

...................................cagcaauuaauggcucgguggc........................................................  1      0  S07
...................................cagcaauuaauggcucgguggcag......................................................  1      0  S07
...................................cagcaauuaauggcucgguggcagu.....................................................  1      0  S07
....................................agcaauuaauggcucgguggc........................................................  12     0  S07
....................................agcaauuaauggcucgguggca.......................................................  2      0  S07
......................................................................acaccgggucguuuauuug........................  3      0  S07
......................................................................acaccgggucguuuauuugc.......................  29     0  S07
......................................................................acaccgggucguuuauuugcu......................  223    0  S07
......................................................................acaccgggucguuuauuugcuu.....................  2377   0  S07
......................................................................acaccgggucguuuauuugcuuu....................  7074   0  S07
......................................................................acaccgggucguuuauuugcuuuu...................  907    0  S07
......................................................................acaccgggucguuuauuugcuuuua..................  24     0  S07
......................................................................acaccgggucguuuauuugcuuuuaa.................  4      0  S07
.......................................................................caccgggucguuuauuugcuu.....................  3      0  S07
.......................................................................caccgggucguuuauuugcuuu....................  5      0  S07
.......................................................................caccgggucguuuauuugcuuuu...................  5      0  S07
........................................................................accgggucguuuauuugcuuu....................  1      0  S07
.........................................................................ccgggucguuuauuugcuuu....................  3      0  S07
.........................................................................ccgggucguuuauuugcuuuu...................  3      0  S07
............................................................................ggucguuuauuugcuuuu...................  1      0  S07

...................................cagcaauuaauggcucgguggc........................................................  7      0  S05
...................................cagcaauuaauggcucgguggca.......................................................  15     0  S05
...................................cagcaauuaauggcucgguggcagu.....................................................  2      0  S05
....................................agcaauuaauggcucggugg.........................................................  2      0  S05
....................................agcaauuaauggcucgguggc........................................................  40     0  S05
....................................agcaauuaauggcucgguggca.......................................................  13     0  S05
..........................................uaauggcucgguggcagugccucgccu............................................  1      0  S05
..................................................cgguggcagugccucgccugac.........................................  1      0  S05
.....................................................................gacaccgggucguuuauuugcu......................  1      0  S05
......................................................................acaccgggucguuuauuu.........................  1      0  S05
......................................................................acaccgggucguuuauuug........................  6      0  S05
......................................................................acaccgggucguuuauuugc.......................  31     0  S05
......................................................................acaccgggucguuuauuugcu......................  337    0  S05
......................................................................acaccgggucguuuauuugcuu.....................  4473   0  S05
......................................................................acaccgggucguuuauuugcuuu....................  18540  0  S05
......................................................................acaccgggucguuuauuugcuuuu...................  3156   0  S05
......................................................................acaccgggucguuuauuugcuuuua..................  131    0  S05
......................................................................acaccgggucguuuauuugcuuuuaa.................  26     0  S05
```

Star   Mature

```
aacagcguuuuaagauaauuuaauacuuuucaagccagcaauuaaauggcucgguggcagugccucgccugacaccgggucguuuauuugcuuuuaaaccgaagggccagaguu
........................................................................caccgggucguuuauuugcuu.....................    6      0    S05
........................................................................caccgggucguuuauuugcuuu....................    25     0    S05
........................................................................caccgggucguuuauuugcuuuu...................    25     0    S05
.........................................................................accgggucguuuauuugcuu.....................    1      0    S05
.........................................................................accgggucguuuauuugcuuu....................    3      0    S05
..........................................................................ccgggucguuuauuugcuu.....................    1      0    S05
..........................................................................ccgggucguuuauuugcuuu....................    9      0    S05

...................................cagcaauuaaauggcucgguggc........................................................    1      0    S10
....................................agcaauuaaauggcucgguggc........................................................    2      0    S10
.....................................................guggcagugccucgccugaca........................................    1      0    S10
.......................................................................acaccgggucguuuauuu.........................    1      0    S10
.......................................................................acaccgggucguuuauuugc.......................    6      0    S10
.......................................................................acaccgggucguuuauuugcu......................    86     0    S10
.......................................................................acaccgggucguuuauuugcuu.....................    537    0    S10
.......................................................................acaccgggucguuuauuugcuuu....................    1907   0    S10
.......................................................................acaccgggucguuuauuugcuuuu...................    200    0    S10
.......................................................................acaccgggucguuuauuugcuuuua..................    9      0    S10
.......................................................................acaccgggucguuuauuugcuuuuaa.................    1      0    S10
........................................................................caccgggucguuuauuugcuuu....................    2      0    S10
.........................................................................accgggucguuuauuugcuuu....................    1      0    S10

...................................cagcaauuaaauggcucggug..........................................................    1      0    S02
...................................cagcaauuaaauggcucgguggc........................................................    23     0    S02
...................................cagcaauuaaauggcucgguggca.......................................................    33     0    S02
...................................cagcaauuaaauggcucgguggcag......................................................    4      0    S02
....................................agcaauuaaauggcucgguggc........................................................    100    0    S02
....................................agcaauuaaauggcucgguggca.......................................................    41     0    S02
....................................agcaauuaaauggcucgguggcag......................................................    6      0    S02
....................................agcaauuaaauggcucgguggcagug....................................................    1      0    S02
....................................agcaauuaaauggcucgguggcagugccuc................................................    5      0    S02
......................................caauuaaauggcucgguggcagugccu.................................................    1      0    S02
................................................gcucgguggcagugccucgccu............................................    1      0    S02
......................................................................gacaccgggucguuuauuugcuu.....................    1      0    S02
.......................................................................acaccgggucguuuauuu.........................    20     0    S02
.......................................................................acaccgggucguuuauuug........................    19     0    S02
.......................................................................acaccgggucguuuauuugc.......................    88     0    S02
.......................................................................acaccgggucguuuauuugcu......................    694    0    S02
.......................................................................acaccgggucguuuauuugcuu.....................    11512  0    S02
.......................................................................acaccgggucguuuauuugcuuu....................    73099  0    S02
.......................................................................acaccgggucguuuauuugcuuuu...................    20930  0    S02
.......................................................................acaccgggucguuuauuugcuuuua..................    785    0    S02
.......................................................................acaccgggucguuuauuugcuuuuaa.................    549    0    S02
.......................................................................acaccgggucguuuauuugcuuuuaaa................    64     0    S02
........................................................................caccgggucguuuauuugcu......................    1      0    S02
........................................................................caccgggucguuuauuugcuu.....................    9      0    S02
........................................................................caccgggucguuuauuugcuuu....................    83     0    S02
........................................................................caccgggucguuuauuugcuuuu...................    178    0    S02
........................................................................caccgggucguuuauuugcuuuua..................    4      0    S02
........................................................................caccgggucguuuauuugcuuuuaa.................    1      0    S02
.........................................................................accgggucguuuauuugcuu.....................    3      0    S02
.........................................................................accgggucguuuauuugcuuu....................    7      0    S02
..........................................................................ccgggucguuuauuugcuu.....................    1      0    S02
..........................................................................ccgggucguuuauuugcuuu....................    34     0    S02
..........................................................................ccgggucguuuauuugcuuuu...................    13     0    S02
...........................................................................cgggucguuuauuugcuuu....................    3      0    S02
...........................................................................cgggucguuuauuugcuuuu...................    1      0    S02
............................................................................gggucguuuauuugcuuu....................    2      0    S02
............................................................................gggucguuuauuugcuuuu...................    1      0    S02

...................................cagcaauuaaauggcucggug..........................................................    1      0    S04
...................................cagcaauuaaauggcucgguggc........................................................    6      0    S04
...................................cagcaauuaaauggcucgguggca.......................................................    9      0    S04
...................................cagcaauuaaauggcucgguggcagu.....................................................    2      0    S04
...................................cagcaauuaaauggcucgguggcagugccu.................................................    1      0    S04
....................................agcaauuaaauggcucgguggc........................................................    47     0    S04
....................................agcaauuaaauggcucgguggca.......................................................    30     0    S04
....................................agcaauuaaauggcucgguggcag......................................................    1      0    S04
.......................................aauuaaauggcucgguggca.......................................................    1      0    S04
.......................................................................acaccgggucguuuauuu.........................    7      0    S04
```

Star **Mature**

```
aacagcguuuuaagauaauuuaauacuuuucaagccagcaauuaauggcucgguggcagugccucgccugacaccgggucguuuauuugcuuuuaaaccgaagggccagaguu
......................................................................acaccgggucguuuauuug........................ 12 0 S04
......................................................................acaccgggucguuuauuugc....................... 48 0 S04
......................................................................acaccgggucguuuauuugcu...................... 465 0 S04
......................................................................acaccgggucguuuauuugcuu..................... 6486 0 S04
......................................................................acaccgggucguuuauuugcuuu.................... 27396 0 S04
......................................................................acaccgggucguuuauuugcuuuu................... 4567 0 S04
......................................................................acaccgggucguuuauuugcuuuua.................. 159 0 S04
......................................................................acaccgggucguuuauuugcuuuuaa................. 42 0 S04
.......................................................................caccgggucguuuauuug........................ 1 0 S04
.......................................................................caccgggucguuuauuugcuu..................... 4 0 S04
.......................................................................caccgggucguuuauuugcuuu.................... 26 0 S04
.......................................................................caccgggucguuuauuugcuuuu................... 31 0 S04
........................................................................accgggucguuuauuugcuu..................... 1 0 S04
........................................................................accgggucguuuauuugcuuu.................... 5 0 S04
.........................................................................ccgggucguuuauuugcuu..................... 1 0 S04
.........................................................................ccgggucguuuauuugcuuu.................... 12 0 S04
.........................................................................ccgggucguuuauuugcuuuu................... 2 0 S04
..........................................................................cgggucguuuauuugcuuu.................... 1 0 S04

....................................agcaauuaauggcucgguggc........................................................ 1 0 S09
....................................agcaauuaauggcucgguggcagugccu................................................. 1 0 S09
......................................................................acaccgggucguuuauuug........................ 1 0 S09
......................................................................acaccgggucguuuauuugc....................... 3 0 S09
......................................................................acaccgggucguuuauuugcu...................... 38 0 S09
......................................................................acaccgggucguuuauuugcuu..................... 223 0 S09
......................................................................acaccgggucguuuauuugcuuu.................... 516 0 S09
......................................................................acaccgggucguuuauuugcuuuu................... 43 0 S09
......................................................................acaccgggucguuuauuugcuuuua.................. 2 0 S09
......................................................................acaccgggucguuuauuugcuuuuaa................. 2 0 S09

...................................cagcaauuaauggcucggu........................................................... 1 0 S03
...................................cagcaauuaauggcucggug.......................................................... 1 0 S03
...................................cagcaauuaauggcucgguggc........................................................ 9 0 S03
...................................cagcaauuaauggcucgguggca....................................................... 24 0 S03
...................................cagcaauuaauggcucgguggcag...................................................... 1 0 S03
...................................cagcaauuaauggcucgguggcagu..................................................... 3 0 S03
....................................agcaauuaauggcucgguggc........................................................ 44 0 S03
....................................agcaauuaauggcucgguggca....................................................... 34 0 S03
....................................agcaauuaauggcucgguggcag...................................................... 1 0 S03
....................................agcaauuaauggcucgguggcagugccuc................................................ 2 0 S03
........................................auuaauggcucgguggcagugccu................................................. 2 0 S03
..........................................uaauggcucgguggcagugccucgccu............................................ 1 0 S03
.....................................................................gacaccgggucguuuauuugcuu..................... 1 0 S03
......................................................................acaccgggucguuuauuu......................... 6 0 S03
......................................................................acaccgggucguuuauuug........................ 9 0 S03
......................................................................acaccgggucguuuauuugc....................... 48 0 S03
......................................................................acaccgggucguuuauuugcu...................... 418 0 S03
......................................................................acaccgggucguuuauuugcuu..................... 6882 0 S03
......................................................................acaccgggucguuuauuugcuuu.................... 29309 0 S03
......................................................................acaccgggucguuuauuugcuuuu................... 4296 0 S03
......................................................................acaccgggucguuuauuugcuuuua.................. 146 0 S03
......................................................................acaccgggucguuuauuugcuuuuaa................. 57 0 S03
.......................................................................caccgggucguuuauuugcuu..................... 8 0 S03
.......................................................................caccgggucguuuauuugcuuu.................... 27 0 S03
.......................................................................caccgggucguuuauuugcuuuu................... 37 0 S03
........................................................................accgggucguuuauuugcuu..................... 3 0 S03
........................................................................accgggucguuuauuugcuuu.................... 3 0 S03
........................................................................accgggucguuuauuugcuuuu................... 2 0 S03
.........................................................................ccgggucguuuauuugcuu..................... 5 0 S03
.........................................................................ccgggucguuuauuugcuuu.................... 6 0 S03
.........................................................................ccgggucguuuauuugcuuuu................... 2 0 S03
..........................................................................cgggucguuuauuugcuuu.................... 2 0 S03

...................................cagcaauuaauggcucggugg......................................................... 1 0 S08
...................................cagcaauuaauggcucgguggc........................................................ 10 0 S08
...................................cagcaauuaauggcucgguggca....................................................... 4 0 S08
....................................agcaauuaauggcucgguggc........................................................ 8 0 S08
....................................agcaauuaauggcucgguggca....................................................... 3 0 S08
.........................................uuaauggcucgguggcagugccucgccu............................................ 1 0 S08
.....................................................................gacaccgggucguuuauuugcuu..................... 1 0 S08
```

Star Mature

aacagcguuuuaagauaauuuaauacuuuucaagccagcaauuaaauggcucgguggcagugccucgccugacaccgggucguuuauuugcuuuuaaaccgaagggccagaguu			
....acaccgggucguuuauuug........................	2	0	S08
....acaccgggucguuuauuugc.......................	34	0	S08
....acaccgggucguuuauuugcu......................	231	0	S08
....acaccgggucguuuauuugcuu.....................	2654	0	S08
....acaccgggucguuuauuugcuuu....................	8361	0	S08
....acaccgggucguuuauuugcuuuu...................	889	0	S08
....acaccgggucguuuauuugcuuuua..................	21	0	S08
....acaccgggucguuuauuugcuuuuaa.................	5	0	S08
....caccgggucguuuauuugc.......................	4	0	S08
....caccgggucguuuauuugcuu.....................	2	0	S08
....caccgggucguuuauuugcuuu....................	11	0	S08
....caccgggucguuuauuugcuuuu...................	4	0	S08
....accgggucguuuauuugcuuu....................	2	0	S08
....ccgggucguuuauuugcuu.....................	3	0	S08
....ccgggucguuuauuugcuuuu...................	2	0	S08
....gggucguuuauuugcuuu....................	1	0	S08

Provisional ID	:	Scaffold1083_21844
Score total	:	29579.2
Score for star read(s)	:	3.9
Score for read counts	:	29578.1
Score for mfe	:	-0.5
Score for randfold	:	-2.2
Score for cons. seed	:	
Total read count	:	58028
Mature read count	:	57801
Loop read count	:	0
Star read count	:	227

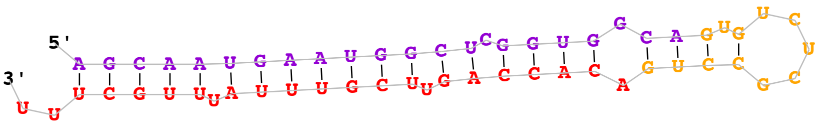

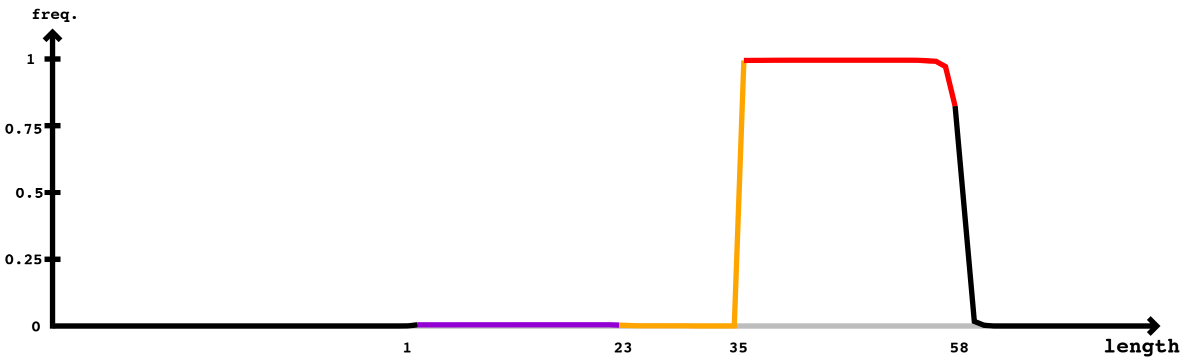

Star Mature

```
5'- aucgguguuuuaagaucauuuaauucuuuucgaaucagcaaugaauggcucgguggcagugucucgccugacaccaguucguuuauuugcuuuuaaaccgaaggaccagugu u   -3'   obs
    aucgguguuuuaagaucauuuaauucuuuucgaaucagcaaugaauggcucgguggcagugucucgccugacaccaguucguuuauuugcuuuuaaaccgaaggaccaguguu         exp
    .(((((................((((.....)))).((((((((((((((.((((.(((.(.....)))).)))))).))))))).))))).....)))))............   reads   mm   sample
    ...................................cagcaaugaauggcucgguggc........................................................   4       0    S08
    ...................................cagcaaugaauggcucgguggca.......................................................   1       0    S08
    ....................................agcaaugaauggcucgguggc........................................................   6       0    S08
    ....................................agcaaugaauggcucgguggca.......................................................   15      0    S08
    ......................................................................acaccaguucguuuauuu.........................   1       0    S08
    ......................................................................acaccaguucguuuauuugc.......................   4       0    S08
    ......................................................................acaccaguucguuuauuugcu......................   4       0    S08
    ......................................................................acaccaguucguuuauuugcuu.....................   73      0    S08
    ......................................................................acaccaguucguuuauuugcuuu....................   172     0    S08
    ......................................................................acaccaguucguuuauuugcuuuu...................   34      0    S08
    ......................................................................acaccaguucguuuauuugcuuuuaa.................   1       0    S08

    ....................................agcaaugaauggcucgguggc........................................................   6       0    S09
    ....................................agcaaugaauggcucgguggca.......................................................   3       0    S09
    ....................................agcaaugaauggcucgguggcag......................................................   1       0    S09
    .......................................aaugaauggcucgguggca.......................................................   1       0    S09
    ......................................................................acaccaguucguuuauuu.........................   1       0    S09
    ......................................................................acaccaguucguuuauuug........................   2       0    S09
    ......................................................................acaccaguucguuuauuugcu......................   9       0    S09
    ......................................................................acaccaguucguuuauuugcuu.....................   61      0    S09
    ......................................................................acaccaguucguuuauuugcuuu....................   105     0    S09
    ......................................................................acaccaguucguuuauuugcuuuu...................   20      0    S09

    ...................................cagcaaugaauggcucgguggca.......................................................   4       0    S03
    ....................................agcaaugaauggcucgguggc........................................................   16      0    S03
    ....................................agcaaugaauggcucgguggca.......................................................   26      0    S03
    ....................................agcaaugaauggcucgguggcag......................................................   6       0    S03
    ....................................agcaaugaauggcucgguggcagugucu.................................................   2       0    S03
    ....................................agcaaugaauggcucgguggcagugucuc................................................   17      0    S03
    .....................................gcaaugaauggcucgguggc........................................................   1       0    S03
    .........................................ugaauggcucgguggcagugucucgccu............................................   5       0    S03
    .....................................................................gacaccaguucguuuauuugcuu.....................   2       0    S03
    .....................................................................gacaccaguucguuuauuugcuuu....................   5       0    S03
```

Star **Mature**

```
aucgguguuuuaagaucauuuaauucuuuucgaaucagcaaugaauggcucgguggcagugucucgccugacaccaguucguuuauuugcuuuuaaaccgaaggaccaguguu
......................................................................acaccaguucguuuauuu.........................   5     0   S03
......................................................................acaccaguucguuuauuug........................   14    0   S03
......................................................................acaccaguucguuuauuugc.......................   17    0   S03
......................................................................acaccaguucguuuauuugcu......................   267   0   S03
......................................................................acaccaguucguuuauuugcuu.....................   2030  0   S03
......................................................................acaccaguucguuuauuugcuuu....................   6326  0   S03
......................................................................acaccaguucguuuauuugcuuuu...................   2158  0   S03
......................................................................acaccaguucguuuauuugcuuuua..................   105   0   S03
......................................................................acaccaguucguuuauuugcuuuuaa.................   9     0   S03
........................................................................accaguucguuuauuugcuuu....................   1     0   S03
.........................................................................ccaguucguuuauuugcuuu....................   2     0   S03
..........................................................................caguucguuCauuugcuu.....................   1     1   S03

......................................................................acaccaguucguuuauuu.........................   2     0   S02
......................................................................acaccaguucguuuauuug........................   40    0   S02
......................................................................acaccaguucguuuauuugc.......................   32    0   S02
......................................................................acaccaguucguuuauuugcu......................   255   0   S02
......................................................................acaccaguucguuuauuugcuu.....................   2003  0   S02
......................................................................acaccaguucguuuauuugcuuu....................   8397  0   S02
......................................................................acaccaguucguuuauuugcuuuu...................   7536  0   S02
......................................................................acaccaguucguuuauuugcuuuua..................   408   0   S02
......................................................................acaccaguucguuuauuugcuuuuaa.................   90    0   S02
......................................................................acaccaguucguuuauuugcuuuuaaa................   7     0   S02
.......................................................................caccaguucguuuauuugcuuu....................   2     0   S02
.......................................................................caccaguucguuuauuugcuuuu...................   4     0   S02
........................................................................accaguucguuuauuugcuuu....................   1     0   S02
........................................................................accaguucguuuauuugcuuuu...................   2     0   S02
.........................................................................ccaguucguuuauuugcuuu....................   5     0   S02
.........................................................................ccaguucguuuauuugcuuuu...................   5     0   S02
..........................................................................caguucguuuauuugcuuu....................   1     0   S02
..........................................................................caguucguuuauuugcuuuu...................   1     0   S02
...........................................................................aguucguuuauuugcuuuu...................   1     0   S02

...................................cagcaaugaauggcucgguggc........................................................   1     0   S04
...................................cagcaaugaauggcucgguggca.......................................................   4     0   S04
...................................cagcaaugaauggcucgguggcag......................................................   1     0   S04
....................................agcaaugaauggcucggugg.........................................................   1     0   S04
....................................agcaaugaauggcucgguggc........................................................   16    0   S04
....................................agcaaugaauggcucgguggca.......................................................   29    0   S04
....................................agcaaugaauggcucgguggcag......................................................   3     0   S04
....................................agcaaugaauggcucgguggcagu.....................................................   2     0   S04
....................................agcaaugaauggcucgguggcagugucuc................................................   4     0   S04
.........................................ugaauggcucgguggcagugucucgccu............................................   1     0   S04
................................................cucgguggcagugucucgccu............................................   1     0   S04
.....................................................................gacaccaguucguuuauuugcuu.....................   1     0   S04
.....................................................................gacaccaguucguuuauuugcuuu....................   1     0   S04
......................................................................acaccaguucguuuauuug........................   18    0   S04
......................................................................acaccaguucguuuauuugc.......................   13    0   S04
......................................................................acaccaguucguuuauuugcu......................   159   0   S04
......................................................................acaccaguucguuuauuugcuu.....................   1114  0   S04
......................................................................acaccaguucguuuauuugcuuu....................   2942  0   S04
......................................................................acaccaguucguuuauuugcuuuu...................   1216  0   S04
......................................................................acaccaguucguuuauuugcuuuua..................   56    0   S04
......................................................................acaccaguucguuuauuugcuuuuaa.................   8     0   S04
.......................................................................caccaguucguuuauuugcuuuu...................   1     0   S04
........................................................................accaguucguuuauuugcuuu....................   1     0   S04
.........................................................................ccaguucguuuauuugcuuu....................   2     0   S04
..........................................................................caguucguuCauuugcuu.....................   1     1   S04
...........................................................................aguucguuuauuugcuuu....................   1     0   S04

....................................agcaaugaauggcucgguggca.......................................................   2     0   S10
......................................................................acaccaguucguuuauuug........................   4     0   S10
......................................................................acaccaguucguuuauuugc.......................   2     0   S10
......................................................................acaccaguucguuuauuugcu......................   18    0   S10
......................................................................acaccaguucguuuauuugcuu.....................   112   0   S10
......................................................................acaccaguucguuuauuugcuuu....................   211   0   S10
......................................................................acaccaguucguuuauuugcuuuu...................   56    0   S10
......................................................................acaccaguucguuuauuugcuuuua..................   2     0   S10

...................................cagcaaugaauggcucggug..........................................................   1     0   S05
```

Star **Mature**

```
aucgguguuuuaagaucauuuaauucuuuucgaucagcaaugaauggcucgguggcagugucucgccugacaccaguucguuuauuugcuuuuaaaccgaaggaccaguguu
...................................cagcaaugaauggcucgguggc........................................................ 2     0   S05
...................................cagcaaugaauggcucgguggca....................................................... 4     0   S05
....................................agcaaugaauggcucggugg......................................................... 1     0   S05
....................................agcaaugaauggcucgguggc........................................................ 13    0   S05
....................................agcaaugaauggcucgguggca....................................................... 27    0   S05
....................................agcaaugaauggcucgguggcag...................................................... 4     0   S05
....................................agcaaugaauggcucgguggcagu..................................................... 1     0   S05
....................................agcaaugaauggcucgguggcaguguc.................................................. 1     0   S05
....................................agcaaugaauggcucgguggcagugucuc................................................ 6     0   S05
.....................................gcaaugaauggcucgguggca....................................................... 2     0   S05
.........................................ugaauggcucgguggcagugucucgccu............................................ 1     0   S05
...............................................gcucgguggcagugucucgccu............................................ 1     0   S05
................................................cucgguggcagugucucgccu............................................ 3     0   S05
.....................................................................gacaccaguucguuuauuugcu...................... 3     0   S05
.....................................................................gacaccaguucguuuauuugcuu..................... 3     0   S05
......................................................................acaccaguucguuuauuug........................ 12    0   S05
......................................................................acaccaguucguuuauuugc....................... 7     0   S05
......................................................................acaccaguucguuuauuugcu...................... 96    0   S05
......................................................................acaccaguucguuuauuugcuu..................... 620   0   S05
......................................................................acaccaguucguuuauuugcuuu.................... 1685  0   S05
......................................................................acaccaguucguuuauuugcuuuu................... 567   0   S05
......................................................................acaccaguucguuuauuugcuuuua.................. 35    0   S05
......................................................................acaccaguucguuuauuugcuuuuaa................. 3     0   S05
.......................................................................caccaguucguuuauuugcuu..................... 2     0   S05
.........................................................................ccaguucguuuauuugcuu..................... 3     0   S05
...........................................................................aguucguuuauuugcuu..................... 1     0   S05

...................................cagcaaugaauggcucgguggc........................................................ 2     0   S07
....................................agcaaugaauggcucgguggc........................................................ 10    0   S07
....................................agcaaugaauggcucgguggca....................................................... 7     0   S07
....................................agcaaugaauggcucgguggcag...................................................... 3     0   S07
....................................agcaaugaauggcucgguggcagugucuc................................................ 1     0   S07
.....................................................................gacaccaguucguuuauuugcuu..................... 1     0   S07
......................................................................acaccaguucguuuauuugcu...................... 14    0   S07
......................................................................acaccaguucguuuauuugcuu..................... 207   0   S07
......................................................................acaccaguucguuuauuugcuuu.................... 444   0   S07
......................................................................acaccaguucguuuauuugcuuuu................... 92    0   S07
......................................................................acaccaguucguuuauuugcuuuua.................. 7     0   S07
......................................................................acaccaguucguuuauuugcuuuuaa................. 1     0   S07

..................................................cgguggcagugucucgccugac......................................... 1     0   S06
......................................................................acaccaguucguuuauuug........................ 4     0   S06
......................................................................acaccaguucguuuauuugc....................... 9     0   S06
......................................................................acaccaguucguuuauuugcu...................... 114   0   S06
......................................................................acaccaguucguuuauuugcuu..................... 754   0   S06
......................................................................acaccaguucguuuauuugcuuu.................... 1586  0   S06
......................................................................acaccaguucguuuauuugcuuuu................... 1083  0   S06
......................................................................acaccaguucguuuauuugcuuuua.................. 53    0   S06
......................................................................acaccaguucguuuauuugcuuuuaa................. 5     0   S06
.......................................................................caccaguucguuuauuugcuu..................... 3     0   S06
.......................................................................caccaguucguuuauuugcuuu.................... 1     0   S06
.........................................................................ccaguucguuuauuugcuu..................... 1     0   S06
.........................................................................ccaguucguuuauuugcuuu.................... 1     0   S06
..........................................................................caguucguuuauuugcuu..................... 1     0   S06

.....................................................................gacaccaguucguuuauuugcu...................... 1     0   S01
.....................................................................gacaccaguucguuuauuugcuu..................... 2     0   S01
......................................................................acaccaguucguuuauuu......................... 2     0   S01
......................................................................acaccaguucguuuauuug........................ 16    0   S01
......................................................................acaccaguucguuuauuugc....................... 26    0   S01
......................................................................acaccaguucguuuauuugcu...................... 227   0   S01
......................................................................acaccaguucguuuauuugcuu..................... 1629  0   S01
......................................................................acaccaguucguuuauuugcuuu.................... 7375  0   S01
......................................................................acaccaguucguuuauuugcuuuu................... 4706  0   S01
......................................................................acaccaguucguuuauuugcuuuua.................. 176   0   S01
......................................................................acaccaguucguuuauuugcuuuuaa................. 46    0   S01
......................................................................acaccaguucguuuauuugcuuuuaaa................ 1     0   S01
.......................................................................caccaguucguuuauuugcuu..................... 2     0   S01
.......................................................................caccaguucguuuauuugcuuu.................... 4     0   S01
........................................................................accaguucguuuauuugcu...................... 1     0   S01
```

```
                                    Star                              Mature
aucgguguuuuaagaucauuuaauucuuuucgaaucagcaaugaauggcucgguggcagugucucgccugacaccaguucguuuauuugcuuuuaaaccgaaggaccagugu u
.........................................................................ccaguucguuuauuugcuuu....................     4     0     S01
.........................................................................ccaguucguuuauuugcuuuu...................     2     0     S01
..........................................................................caguucguuuauuugcuuuu...................     2     0     S01
...........................................................................aguucguuuauuugcuuu....................     1     0     S01
```

Provisional ID	:	Scaffold1083_21840
Score total	:	27626.3
Score for star read(s)	:	3.9
Score for read counts	:	27620.9
Score for mfe	:	0
Score for randfold	:	1.6
Score for cons. seed	:	
Total read count	:	54189
Mature read count	:	53851
Loop read count	:	0
Star read count	:	338

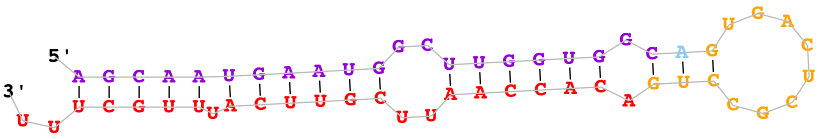

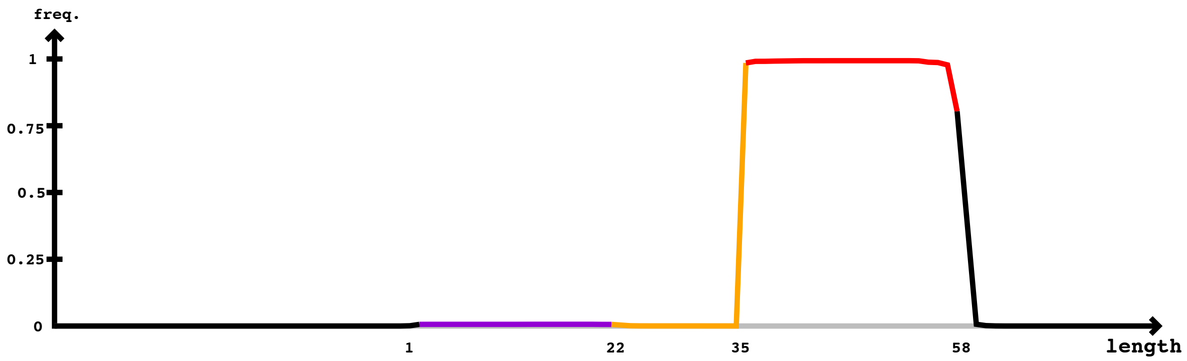

Star Mature

```
5'- accagcguuuuaagaucauuuaaucuuuuuccaaccagcaaugaauggcuugguggcagugacucgccugacaccaauucguucauuugcuuuuaaaccgaaggacagaguuc  -3'   obs
    accagcguuuuaagaucauuuaaucuuuuuccaaccagcaaugaauggcuugguggcagugacucgccugacaccaauucguucauuugcuuuuaaaccgaaggacagaguuc        exp
    .((..((.((((((((......)))...........((((((((((..((((((.(((........))).))))))..)))))).))))).))))).))..)).........   reads   mm   sample
    ...................................cagcaaugaauggcuuggug..........................................................   1       0    S08
    ...................................cagcaaugaauggcuugguggc........................................................   3       0    S08
    ...................................cagcaaugaauggcuugguggca.......................................................   1       0    S08
    ....................................agcaaugaauggcuuggug..........................................................   1       0    S08
    ....................................agcaaugaauggcuugguggc........................................................   4       0    S08
    ....................................agcaaugaauggcuugguggca.......................................................   3       0    S08
    ....................................agcaaugaauggcuugguggcag......................................................   1       0    S08
    ...............................................gcuugguggcagugacucgccu............................................   2       0    S08
    .....................................................uggcagugacucgccugacacc......................................   1       0    S08
    ......................................................................acaccaauucguucauuu.........................   1       0    S08
    ......................................................................acaccaauucguucauuug........................   12      0    S08
    ......................................................................acaccaauucguucauuugc.......................   4       0    S08
    ......................................................................acaccaauucguucauuugcu......................   12      0    S08
    ......................................................................acaccaauucguucauuugcuu.....................   407     0    S08
    ......................................................................acaccaauucguucauuugcuuu....................   658     0    S08
    ......................................................................acaccaauucguucauuugcuuuu...................   479     0    S08
    ......................................................................acaccaauucguucauuugcuuuua..................   6       0    S08
    ......................................................................acaccaauucguucauuugcuuuuaa.................   2       0    S08
    .......................................................................caccaauucguucauuugcuu.....................   3       0    S08
    .......................................................................caccaauucguucauuugcuuu....................   3       0    S08
    .......................................................................caccaauucguucauuugcuuuu...................   5       0    S08
    .........................................................................ccaauucguucauuugcuu.....................   1       0    S08
    .........................................................................ccaauucguucauuugcuuu....................   1       0    S08
    .........................................................................ccaauucguucauuugcuuuu...................   2       0    S08
    ..........................................................................caauucguucauuugcuu.....................   1       0    S08
    ..........................................................................caauucguucauuugcuuu....................   2       0    S08
    ...........................................................................aauucguucauuugcuuu....................   2       0    S08
    ...........................................................................aauucguucauuugcuuuu...................   2       0    S08

    ...................................cagcaaugaauggcuuggug..........................................................   1       0    S03
    ...................................cagcaaugaauggcuuggugg.........................................................   1       0    S03
    ...................................cagcaaugaauggcuugguggc........................................................   2       0    S03
    ...................................cagcaaugaauggcuugguggca.......................................................   3       0    S03
```

```
                                    Star                              Mature
accagcguuuuaagaucauuuaaucuuuuuccaaccagcaaugaauggcuugguggcagugacucgccugacaccaauucguucauuugcuuuuaaaccgaaggacagaguuc
....................................agcaaugaauggcuuggugg.........................................................  1     0  S03
....................................agcaaugaauggcuugguggc........................................................  25    0  S03
....................................agcaaugaauggcuugguggca.......................................................  7     0  S03
....................................agcaaugaauggcuugguggcag......................................................  4     0  S03
....................................agcaaugaauggcuugguggcagugacuc................................................  1     0  S03
.....................................gcaaugaauggcuugguggca.......................................................  1     0  S03
....................................................guggcagugacucgccugacac.......................................  1     0  S03
......................................................................acaccaauucguucauuu.........................  3     0  S03
......................................................................acaccaauucguucauuug........................  42    0  S03
......................................................................acaccaauucguucauuugc.......................  12    0  S03
......................................................................acaccaauucguucauuugcu......................  83    0  S03
......................................................................acaccaauucguucauuugcuu.....................  1412  0  S03
......................................................................acaccaauucguucauuugcuuu....................  3588  0  S03
......................................................................acaccaauucguucauuugcuuuu...................  2904  0  S03
......................................................................acaccaauucguucauuugcuuuua..................  51    0  S03
......................................................................acaccaauucguucauuugcuuuuaa.................  7     0  S03
.......................................................................caccaauucguucauuugcuu.....................  1     0  S03
.......................................................................caccaauucguucauuugcuuu....................  7     0  S03
.......................................................................caccaauucguucauuugcuuuu...................  22    0  S03
........................................................................accaauucguucauuugcuuu....................  1     0  S03
.........................................................................ccaauucguucauuugcuuu....................  3     0  S03
.........................................................................ccaauucguucauuugcuuuu...................  4     0  S03
..........................................................................caGuucguucauuugcuu.....................  1     1  S03
..........................................................................caauucguucauuugcuuu....................  1     0  S03
..........................................................................caauucguucauuugcuuuu...................  3     0  S03
...........................................................................aauucguucauuugcuuu....................  3     0  S03
...........................................................................aauucguucauuugcuuuu...................  3     0  S03
............................................................................auucguucauuugcuuuu...................  2     0  S03

...................................cagcaaugaauggcuuggug..........................................................  1     0  S09
...................................cagcaaugaauggcuugguggc........................................................  1     0  S09
......................................................................acaccaauucguucauuu.........................  4     0  S09
......................................................................acaccaauucguucauuug........................  8     0  S09
......................................................................acaccaauucguucauuugcu......................  33    0  S09
......................................................................acaccaauucguucauuugcuu.....................  628   0  S09
......................................................................acaccaauucguucauuugcuuu....................  881   0  S09
......................................................................acaccaauucguucauuugcuuuu...................  759   0  S09
......................................................................acaccaauucguucauuugcuuuua..................  3     0  S09
......................................................................acaccaauucguucauuugcuuuuaa.................  4     0  S09
......................................................................acaccaauucguucauuugcuuuuaaa................  1     0  S09

...................................cagcaaugaauggcuugg............................................................  1     0  S02
...................................cagcaaugaauggcuuggug..........................................................  1     0  S02
...................................cagcaaugaauggcuugguggc........................................................  5     0  S02
...................................cagcaaugaauggcuugguggca.......................................................  7     0  S02
....................................agcaaugaauggcuuggug..........................................................  3     0  S02
....................................agcaaugaauggcuuggugg.........................................................  1     0  S02
....................................agcaaugaauggcuugguggc........................................................  42    0  S02
....................................agcaaugaauggcuugguggca.......................................................  10    0  S02
....................................agcaaugaauggcuugguggcag......................................................  8     0  S02
....................................agcaaugaauggcuugguggcagugacuc................................................  1     0  S02
................................................cuugguggcagugacucgccu............................................  3     0  S02
......................................................................acaccaauucguucauuu.........................  4     0  S02
......................................................................acaccaauucguucauuug........................  44    0  S02
......................................................................acaccaauucguucauuugc.......................  14    0  S02
......................................................................acaccaauucguucauuugcu......................  48    0  S02
......................................................................acaccaauucguucauuugcuu.....................  851   0  S02
......................................................................acaccaauucguucauuugcuuu....................  3031  0  S02
......................................................................acaccaauucguucauuugcuuuu...................  3045  0  S02
......................................................................acaccaauucguucauuugcuuuua..................  60    0  S02
......................................................................acaccaauucguucauuugcuuuuaa.................  24    0  S02
......................................................................acaccaauucguucauuugcuuuuaaa................  3     0  S02
.......................................................................caccaauucguucauuugcuu.....................  1     0  S02
.......................................................................caccaauucguucauuugcuuu....................  12    0  S02
.......................................................................caccaauucguucauuugcuuuu...................  46    0  S02
.........................................................................ccaauucguucauuugcuu.....................  1     0  S02
.........................................................................ccaauucguucauuugcuuu....................  1     0  S02
.........................................................................ccaauucguucauuugcuuuu...................  2     0  S02
..........................................................................caauucguucauuugcuuu....................  6     0  S02
...........................................................................aauucguucauuugcuuuu...................  4     0  S02
```

```
                                     Star                                Mature
accagcguuuuaagaucauuuaaucuuuuuccaaccagcaaugaauggcuugguggcagugacucgccugacaccaauucguucauuugcuuuuaaaccgaaggacagaguuc
............................................................................auucguucauuugcuuuu...................   7      0   S02

...................................cagcaaugaauggcuuggug..........................................................   5      0   S04
...................................cagcaaugaauggcuugguggg.........................................................   1      0   S04
...................................cagcaaugaauggcuugguggc........................................................   1      0   S04
...................................cagcaaugaauggcuugguggca.......................................................   1      0   S04
....................................agcaaugaauggcuuggugg.........................................................   2      0   S04
....................................agcaaugaauggcuugguggc........................................................   30     0   S04
....................................agcaaugaauggcuugguggca.......................................................   10     0   S04
....................................agcaaugaauggcuugguggcag......................................................   3      0   S04
.............................................uggcuugguggcagugacucg...............................................   1      0   S04
................................................cuugguggcagugacucgc..............................................   1      0   S04
......................................................................acaccaauucguucauuu.........................   5      0   S04
......................................................................acaccaauucguucauuug........................   59     0   S04
......................................................................acaccaauucguucauuugc.......................   16     0   S04
......................................................................acaccaauucguucauuugcu......................   104    0   S04
......................................................................acaccaauucguucauuugcuu.....................   2226   0   S04
......................................................................acaccaauucguucauuugcuuu....................   5055   0   S04
......................................................................acaccaauucguucauuugcuuuu...................   4263   0   S04
......................................................................acaccaauucguucauuugcuuuua..................   58     0   S04
......................................................................acaccaauucguucauuugcuuuuaa.................   4      0   S04
.......................................................................caccaauucguucauuugcuu.....................   5      0   S04
.......................................................................caccaauucguucauuugcuuu....................   22     0   S04
.......................................................................caccaauucguucauuugcuuuu...................   56     0   S04
.......................................................................caccaauucguucauuugcuuuuaa.................   1      0   S04
.........................................................................ccaauucguucauuugcu......................   1      0   S04
.........................................................................ccaauucguucauuugcuu.....................   1      0   S04
.........................................................................ccaauucguucauuugcuuu....................   3      0   S04
.........................................................................ccaauucguucauuugcuuuu...................   3      0   S04
..........................................................................caGuucguucauuugcuu.....................   1      1   S04
..........................................................................caauucguucauuugcuuu....................   2      0   S04
..........................................................................caauucguucauuugcuuuu...................   3      0   S04
...........................................................................aauucguucauuugcuuu....................   2      0   S04
...........................................................................aauucguucauuugcuuuu...................   3      0   S04
............................................................................auucguucauuugcuuuu...................   7      0   S04

....................................agcaaugaauggcuuggugg.........................................................   1      0   S10
....................................agcaaugaauggcuugguggc........................................................   1      0   S10
......................................................................acaccaauucguucauuu.........................   2      0   S10
......................................................................acaccaauucguucauuug........................   10     0   S10
......................................................................acaccaauucguucauuugcu......................   37     0   S10
......................................................................acaccaauucguucauuugcuu.....................   721    0   S10
......................................................................acaccaauucguucauuugcuuu....................   1064   0   S10
......................................................................acaccaauucguucauuugcuuuu...................   834    0   S10
......................................................................acaccaauucguucauuugcuuuua..................   5      0   S10
......................................................................acaccaauucguucauuugcuuuuaa.................   1      0   S10
......................................................................acaccaauucguucauuugcuuuuaaa................   1      0   S10
.......................................................................caccaauucguucauuugcuuuu...................   1      0   S10
.........................................................................ccaauucguucauuugcuuu....................   1      0   S10

...................................cagcaaugaauggcuuggug..........................................................   2      0   S05
...................................cagcaaugaauggcuugguggca.......................................................   2      0   S05
....................................agcaaugaauggcuugguggc........................................................   18     0   S05
....................................agcaaugaauggcuugguggca.......................................................   2      0   S05
....................................agcaaugaauggcuugguggcag......................................................   4      0   S05
....................................agcaaugaauggcuugguggcagu.....................................................   1      0   S05
................................................cuugguggcagugacucgccuga..........................................   1      0   S05
......................................................................acaccaauucguucauuu.........................   3      0   S05
......................................................................acaccaauucguucauuug........................   38     0   S05
......................................................................acaccaauucguucauuugc.......................   11     0   S05
......................................................................acaccaauucguucauuugcu......................   67     0   S05
......................................................................acaccaauucguucauuugcuu.....................   1230   0   S05
......................................................................acaccaauucguucauuugcuuu....................   3133   0   S05
......................................................................acaccaauucguucauuugcuuuu...................   2506   0   S05
......................................................................acaccaauucguucauuugcuuuua..................   32     0   S05
......................................................................acaccaauucguucauuugcuuuuaa.................   4      0   S05
.......................................................................caccaauucguucauuugcu.......................   1      0   S05
.......................................................................caccaauucguucauuugcuu.....................   3      0   S05
.......................................................................caccaauucguucauuugcuuu....................   10     0   S05
.......................................................................caccaauucguucauuugcuuuu...................   20     0   S05
```

Star **Mature**

```
accagcguuuuaagaucauuuaaucuuuuuccaaccagcaaugaauggcuugguggcagugacucgccugacaccaauucguucauuugcuuuuaaaccgaaggacagaguuc
.........................................................................ccaauucguucauuugcu......................    1    0    S05
.........................................................................ccaauucguucauuugcuu.....................    2    0    S05
.........................................................................ccaauucguucauuugcuuu....................    5    0    S05
.........................................................................ccaauucguucauuugcuuuu...................    5    0    S05
..........................................................................caauucguucauuugcuu.....................    1    0    S05
..........................................................................caauucguucauuugcuuu....................    3    0    S05
..........................................................................caauucguucauuugcuuuu...................    1    0    S05
...........................................................................aauucguucauuugcuuu....................    3    0    S05
...........................................................................aauucguucauuugcuuuu...................    3    0    S05

....................................agcaaugaauggcuuggug..........................................................    2    0    S07
....................................agcaaugaauggcuuggugg.........................................................    1    0    S07
....................................agcaaugaauggcuugguggc........................................................    20   0    S07
....................................agcaaugaauggcuugguggca.......................................................    7    0    S07
....................................agcaaugaauggcuugguggcag......................................................    1    0    S07
.....................................gcaaugaauggcuugguggca.......................................................    1    0    S07
......................................................................acaccaauucguucauuu.........................    1    0    S07
......................................................................acaccaauucguucauuug........................    15   0    S07
......................................................................acaccaauucguucauuugc.......................    2    0    S07
......................................................................acaccaauucguucauuugcu......................    29   0    S07
......................................................................acaccaauucguucauuugcuu.....................    678  0    S07
......................................................................acaccaauucguucauuugcuuu....................    1460 0    S07
......................................................................acaccaauucguucauuugcuuuu...................    1399 0    S07
......................................................................acaccaauucguucauuugcuuuua..................    11   0    S07
.......................................................................caccaauucguucauuugcuuu....................    1    0    S07
.......................................................................caccaauucguucauuugcuuuu...................    4    0    S07
..........................................................................caauucguucauuugcuuuu...................    2    0    S07
...........................................................................aauucguucauuugcuuu....................    1    0    S07
............................................................................auucguucauuugcuuuu...................    1    0    S07

...................................cagcaaugaauggcuuggugg.........................................................    2    0    S06
....................................agcaaugaauggcuuggug..........................................................    1    0    S06
....................................agcaaugaauggcuugguggc........................................................    13   0    S06
....................................agcaaugaauggcuugguggca.......................................................    3    0    S06
....................................agcaaugaauggcuugguggcag......................................................    6    0    S06
................................................cuugguggcagugacucgccu............................................    1    0    S06
......................................................................acaccaauucguucauuu.........................    4    0    S06
......................................................................acaccaauucguucauuug........................    22   0    S06
......................................................................acaccaauucguucauuugc.......................    4    0    S06
......................................................................acaccaauucguucauuugcu......................    30   0    S06
......................................................................acaccaauucguucauuugcuu.....................    600  0    S06
......................................................................acaccaauucguucauuugcuuu....................    1298 0    S06
......................................................................acaccaauucguucauuugcuuuu...................    1076 0    S06
......................................................................acaccaauucguucauuugcuuuua..................    7    0    S06
......................................................................acaccaauucguucauuugcuuuuaa.................    1    0    S06
......................................................................acaccaauucguucauuugcuuuuaaa................    1    0    S06
.......................................................................caccaauucguucauuugcuu.....................    2    0    S06
.......................................................................caccaauucguucauuugcuuu....................    3    0    S06
.......................................................................caccaauucguucauuugcuuuu...................    9    0    S06
........................................................................accaauucguucauuugcuuu....................    1    0    S06
.........................................................................ccaauucguucauuugcuuuu...................    2    0    S06
...........................................................................aauucguucauuugcuuu....................    1    0    S06
............................................................................auucguucauuugcuuuu...................    2    0    S06
...............................................................................cguucauuugcuuuuaaG................    2    1    S06

...................................cagcaaugaauggcuuggug..........................................................    1    0    S01
...................................cagcaaugaauggcuugguggc........................................................    3    0    S01
...................................cagcaaugaauggcuugguggca.......................................................    2    0    S01
....................................agcaaugaauggcuuggug..........................................................    2    0    S01
....................................agcaaugaauggcuugguggc........................................................    38   0    S01
....................................agcaaugaauggcuugguggca.......................................................    3    0    S01
....................................agcaaugaauggcuugguggcag......................................................    5    0    S01
....................................agcaaugaauggcuugguggcagugac..................................................    1    0    S01
....................................agcaaugaauggcuugguggcagugacu.................................................    1    0    S01
.....................................gcaaugaauggcuugguggca.......................................................    2    0    S01
.......................................aaugaauggcuugguggc........................................................    1    0    S01
.............................................uggcuugguggcagugacucgc..............................................    1    0    S01
................................................cuugguggcagugacucgcc.............................................    1    0    S01
................................................cuugguggcagugacucgccu............................................    1    0    S01
.....................................................................gacaccaauucguucauuugc.......................    1    0    S01
```

```
                                    Star                             Mature
accagcguuuuaagaucauuuaaucuuuuuccaaccagcaaugaauggcuugguggcagugacucgccugacaccaauucguucauuugcuuuuaaaccgaaggacagaguuc
......................................................................acaccaauucguucauuug........................   24     0     S01
......................................................................acaccaauucguucauuugc.......................   6      0     S01
......................................................................acaccaauucguucauuugcu......................   36     0     S01
......................................................................acaccaauucguucauuugcuu.....................   649    0     S01
......................................................................acaccaauucguucauuugcuuu....................   2648   0     S01
......................................................................acaccaauucguucauuugcuuuu...................   2714   0     S01
......................................................................acaccaauucguucauuugcuuuua..................   34     0     S01
......................................................................acaccaauucguucauuugcuuuuaa.................   21     0     S01
......................................................................acaccaauucguucauuugcuuuuaaa................   6      0     S01
.......................................................................caccaauucguucauuugcu......................   1      0     S01
.......................................................................caccaauucguucauuugcuu.....................   1      0     S01
.......................................................................caccaauucguucauuugcuuu....................   20     0     S01
.......................................................................caccaauucguucauuugcuuuu...................   69     0     S01
.......................................................................caccaauucguucauuugcuuuua..................   1      0     S01
........................................................................accaauucguucauuugcuuuu...................   1      0     S01
.........................................................................ccaauucguucauuugcuuu....................   2      0     S01
.........................................................................ccaauucguucauuugcuuuu...................   1      0     S01
..........................................................................caauucguucauuugcuuu....................   1      0     S01
..........................................................................caauucguucauuugcuuuu...................   1      0     S01
...........................................................................aauucguucauuugcuuuu...................   1      0     S01
............................................................................auucguucauuugcuuuu...................   4      0     S01
```

Provisional ID	:	Scaffold1083_21836
Score total	:	13939.1
Score for star read(s)	:	3.9
Score for read counts	:	13933.1
Score for mfe	:	0.5
Score for randfold	:	1.6
Score for cons. seed	:	
Total read count	:	27341
Mature read count	:	26578
Loop read count	:	0
Star read count	:	763

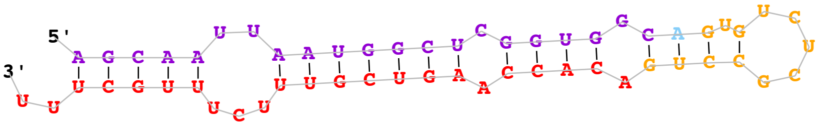

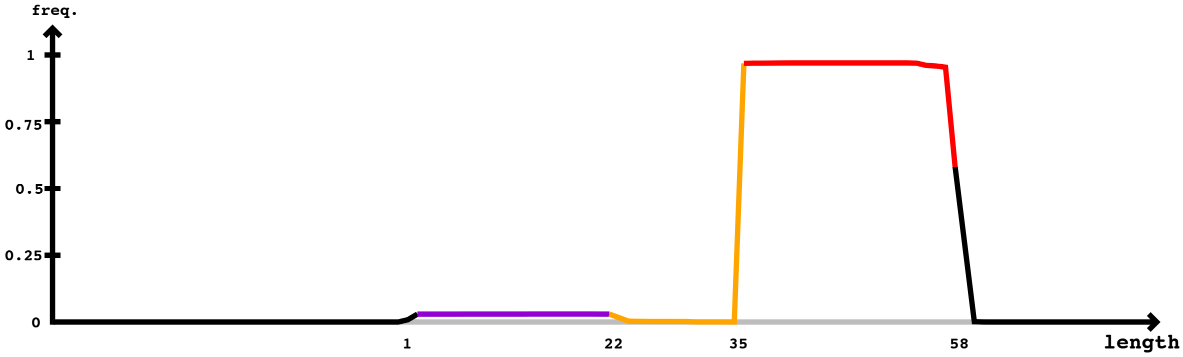

Star Mature

5'- nnnnnnnnnuuaauauccuuuaauccuuuucgaaccagcaauuaauggcucgguggcagugucucgccugacaccaagucguuucuuugcuuuuagaccgaagggccagaguu -3'	obs		
nnnnnnnnnuuaauauccuuuaauccuuuucgaaccagcaauuaauggcucgguggcagugucucgccugacaccaagucguuucuuugcuuuuagaccgaagggccagaguu	exp		
.................((((..((((((((.....(((((..(((((((.((((.(((.(.....)))).)))).)))))))...)))))....))..))))))..))))..	reads	mm	sample
...................................cagcaauuaauggcucggugg...	1	0	S08
...................................cagcaauuaauggcucgguggc..	10	0	S08
...................................cagcaauuaauggcucgguggca...	4	0	S08
....................................agcaauuaauggcucgguggc..	8	0	S08
....................................agcaauuaauggcucgguggca...	3	0	S08
....acaccaagucguuucuuug........................	1	0	S08
....acaccaagucguuucuuugc.......................	1	0	S08
....acaccaagucguuucuuugcu......................	3	0	S08
....acaccaagucguuucuuugcuu.....................	185	0	S08
....acaccaagucguuucuuugcuuu....................	171	0	S08
....acaccaagucguuucuuugcuuuu...................	47	0	S08
....caccaagucguuucuuugcuuu....................	1	0	S08
....................................agcaauuaauggcucgguggc..	1	0	S09
....................................agcaauuaauggcucgguggcagugucuc..	1	0	S09
....acaccaagucguuucuuugcu......................	6	0	S09
....acaccaagucguuucuuugcuu.....................	108	0	S09
....acaccaagucguuucuuugcuuu....................	100	0	S09
....acaccaagucguuucuuugcuuuu...................	30	0	S09
....acaccaagucguuucuuugcuuuua..................	1	0	S09
...................................cagcaauuaauggcucggu...	1	0	S03
...................................cagcaauuaauggcucggug..	1	0	S03
...................................cagcaauuaauggcucgguggc..	9	0	S03
...................................cagcaauuaauggcucgguggca...	24	0	S03
...................................cagcaauuaauggcucgguggcag..	1	0	S03
...................................cagcaauuaauggcucgguggcagu...	3	0	S03
....................................agcaauuaauggcucgguggc..	44	0	S03
....................................agcaauuaauggcucgguggca...	34	0	S03
....................................agcaauuaauggcucgguggcag..	1	0	S03
....................................agcaauuaauggcucgguggcagugu...	1	0	S03
....................................agcaauuaauggcucgguggcagugucuc..	13	0	S03
....acaccaagucguuucuuu.........................	2	0	S03

Star **Mature**

```
nnnnnnnnnuuaauauccuuuaauccuuuucgaaccagcaauuaauggcucgguggcagugucucgccugacaccaagucguuucuuugcuuuuagaccgaagggccagaguu
......................................................................acaccaagucguuucuuug........................ 29 0 S03
......................................................................acaccaagucguuucuuugc....................... 3 0 S03
......................................................................acaccaagucguuucuuugcu...................... 12 0 S03
......................................................................acaccaagucguuucuuugcuu..................... 1292 0 S03
......................................................................acaccaagucguuucuuugcuuu.................... 1207 0 S03
......................................................................acaccaagucguuucuuugcuuuu................... 425 0 S03
......................................................................acaccaagucguuucuuugcuuuua.................. 5 0 S03
.......................................................................caccaagucguuucuuugcuuuu................... 1 0 S03
........................................................................accaagucguuucuuugcuuu.................... 1 0 S03
.........................................................................ccaagucguuucuuugcuuu.................... 2 0 S03

...................................cagcaauuaauggcucggug.......................................................... 1 0 S04
...................................cagcaauuaauggcucgguggc........................................................ 6 0 S04
...................................cagcaauuaauggcucgguggca....................................................... 9 0 S04
...................................cagcaauuaauggcucgguggcagu..................................................... 2 0 S04
....................................agcaauuaauggcucgguggc........................................................ 47 0 S04
....................................agcaauuaauggcucgguggca....................................................... 30 0 S04
....................................agcaauuaauggcucgguggcag...................................................... 1 0 S04
....................................agcaauuaauggcucgguggcagugucuc................................................ 1 0 S04
.......................................aauuaauggcucgguggca....................................................... 1 0 S04
................................................cucgguggcagugucucgccu............................................ 1 0 S04
......................................................................acaccaagucguuucuuu......................... 1 0 S04
......................................................................acaccaagucguuucuuug........................ 31 0 S04
......................................................................acaccaagucguuucuuugc....................... 6 0 S04
......................................................................acaccaagucguuucuuugcu...................... 9 0 S04
......................................................................acaccaagucguuucuuugcuu..................... 1321 0 S04
......................................................................acaccaagucguuucuuugcuuu.................... 1288 0 S04
......................................................................acaccaagucguuucuuugcuuuu................... 469 0 S04
......................................................................acaccaagucguuucuuugcuuuua.................. 1 0 S04
......................................................................acaccaagucguuucuuugcuuuuag................. 1 0 S04
.......................................................................caccaagucguuucuuugcuu..................... 1 0 S04
.........................................................................ccaagucguuucuuugcuuu.................... 2 0 S04
..........................................................................caagucguuucuuugcuu..................... 1 0 S04
...........................................................................aagucguuucuuugcuuu.................... 1 0 S04

...................................cagcaauuaauggcucggug.......................................................... 1 0 S02
...................................cagcaauuaauggcucgguggc........................................................ 23 0 S02
...................................cagcaauuaauggcucgguggca....................................................... 33 0 S02
...................................cagcaauuaauggcucgguggcag...................................................... 4 0 S02
....................................agcaauuaauggcucgguggc........................................................ 100 0 S02
....................................agcaauuaauggcucgguggca....................................................... 41 0 S02
....................................agcaauuaauggcucgguggcag...................................................... 6 0 S02
....................................agcaauuaauggcucgguggcagug.................................................... 1 0 S02
....................................agcaauuaauggcucgguggcagugucu................................................. 1 0 S02
....................................agcaauuaauggcucgguggcagugucuc................................................ 11 0 S02
......................................................................acaccaagucguuucuuu......................... 7 0 S02
......................................................................acaccaagucguuucuuug........................ 71 0 S02
......................................................................acaccaagucguuucuuugc....................... 17 0 S02
......................................................................acaccaagucguuucuuugcu...................... 17 0 S02
......................................................................acaccaagucguuucuuugcuu..................... 2274 0 S02
......................................................................acaccaagucguuucuuugcuuu.................... 2952 0 S02
......................................................................acaccaagucguuucuuugcuuuu................... 1355 0 S02
......................................................................acaccaagucguuucuuugcuuuua.................. 13 0 S02
......................................................................acaccaagucguuucuuugcuuuuag................. 2 0 S02
.......................................................................caccaagucguuucuuugcuu..................... 1 0 S02
.......................................................................caccaagucguuucuuugcuuu.................... 1 0 S02
.......................................................................caccaagucguuucuuugcuuuu................... 3 0 S02
........................................................................accaagucguuucuuugcuuu.................... 1 0 S02
.........................................................................ccaagucguuucuuugcuuu.................... 1 0 S02
..........................................................................caagucguuucuuugcuuu.................... 1 0 S02
..........................................................................caagucguuucuuugcuuuu................... 1 0 S02
...........................................................................aagucguuucuuugcuuu.................... 1 0 S02
...........................................................................aagucguuucuuugcuuuu................... 1 0 S02

...................................cagcaauuaauggcucgguggc........................................................ 1 0 S10
....................................agcaauuaauggcucgguggc........................................................ 2 0 S10
......................................................................acaccaagucguuucuuu......................... 2 0 S10
......................................................................acaccaagucguuucuuugcu...................... 17 0 S10
......................................................................acaccaagucguuucuuugcuu..................... 279 0 S10
......................................................................acaccaagucguuucuuugcuuu.................... 314 0 S10
```

```
                                                     Star                               Mature
nnnnnnnnnuuaauauccuuuaauccuuuucgaaccagcaauuaauggcucgguggcagugucucgccugacaccaagucguuucuuugcuuuuagaccgaagggccagaguu
......................................................................acaccaagucguuucuuugcuuuu...................    92    0    S10
.......................................................................caccaagucguuucuuugcuu.....................    1     0    S10

...................................cagcaauuaauggcucgguggc........................................................    7     0    S05
...................................cagcaauuaauggcucgguggca.......................................................    15    0    S05
...................................cagcaauuaauggcucgguggcagu.....................................................    2     0    S05
....................................agcaauuaauggcucggugg.........................................................    2     0    S05
....................................agcaauuaauggcucgguggc........................................................    40    0    S05
....................................agcaauuaauggcucgguggca.......................................................    13    0    S05
....................................agcaauuaauggcucgguggcagugucuc................................................    3     0    S05
...............................................gcucgguggcagugucucgccu............................................    1     0    S05
................................................cucgguggcagugucucgccu............................................    3     0    S05
......................................................................acaccaagucguuucuuu.........................    5     0    S05
......................................................................acaccaagucguuucuuug........................    15    0    S05
......................................................................acaccaagucguuucuuugc.......................    3     0    S05
......................................................................acaccaagucguuucuuugcu......................    12    0    S05
......................................................................acaccaagucguuucuuugcuu.....................    1056  0    S05
......................................................................acaccaagucguuucuuugcuuu....................    1075  0    S05
......................................................................acaccaagucguuucuuugcuuuu...................    470   0    S05
......................................................................acaccaagucguuucuuugcuuuua..................    3     0    S05
.......................................................................caccaagucguuucuuugcuuu....................    2     0    S05
.......................................................................caccaagucguuucuuugcuuuu...................    2     0    S05
..........................................................................caagucguuucuuugcuu.....................    2     0    S05
..........................................................................caagucguuucuuugcuuu....................    2     0    S05

...................................cagcaauuaauggcucgguggc........................................................    1     0    S07
...................................cagcaauuaauggcucgguggcag......................................................    1     0    S07
...................................cagcaauuaauggcucgguggcagu.....................................................    1     0    S07
....................................agcaauuaauggcucgguggc........................................................    12    0    S07
....................................agcaauuaauggcucgguggca.......................................................    2     0    S07
....................................agcaauuaauggcucgguggcagugucuc................................................    2     0    S07
......................................................................acaccaagucguuucuuug........................    8     0    S07
......................................................................acaccaagucguuucuuugc.......................    2     0    S07
......................................................................acaccaagucguuucuuugcu......................    24    0    S07
......................................................................acaccaagucguuucuuugcuu.....................    535   0    S07
......................................................................acaccaagucguuucuuugcuuu....................    472   0    S07
......................................................................acaccaagucguuucuuugcuuuu...................    174   0    S07
......................................................................acaccaagucguuucuuugcuuuua..................    4     0    S07
.......................................................................caccaagucguuucuuugcuuu....................    1     0    S07
...........................................................................aagucguuucuuugcuuu....................    1     0    S07

...................................cagcaauuaauggcucgguggc........................................................    3     0    S06
...................................cagcaauuaauggcucgguggca.......................................................    11    0    S06
....................................agcaauuaauggcucgguggc........................................................    21    0    S06
....................................agcaauuaauggcucgguggca.......................................................    13    0    S06
....................................agcaauuaauggcucgguggcagugucuc................................................    5     0    S06
..................................................cgguggcagugucucgccugac.........................................    1     0    S06
......................................................................acaccaagucguuucuuu.........................    6     0    S06
......................................................................acaccaagucguuucuuug........................    36    0    S06
......................................................................acaccaagucguuucuuugc.......................    13    0    S06
......................................................................acaccaagucguuucuuugcu......................    15    0    S06
......................................................................acaccaagucguuucuuugcuu.....................    1714  0    S06
......................................................................acaccaagucguuucuuugcuuu....................    1360  0    S06
......................................................................acaccaagucguuucuuugcuuuu...................    580   0    S06
......................................................................acaccaagucguuucuuugcuuuua..................    1     0    S06
.......................................................................caccaagucguuucuuugcuuu....................    1     0    S06
.......................................................................caccaagucguuucuuugcuuuu...................    1     0    S06
...........................................................................aagucguuucuuugcuuu....................    2     0    S06

...................................cagcaauuaauggcucgguggc........................................................    18    0    S01
...................................cagcaauuaauggcucgguggca.......................................................    37    0    S01
...................................cagcaauuaauggcucgguggcag......................................................    3     0    S01
...................................cagcaauuaauggcucgguggcagu.....................................................    1     0    S01
....................................agcaauuaauggcucgguggc........................................................    55    0    S01
....................................agcaauuaauggcucgguggca.......................................................    48    0    S01
....................................agcaauuaauggcucgguggcag......................................................    1     0    S01
....................................agcaauuaauggcucgguggcaguguc..................................................    1     0    S01
....................................agcaauuaauggcucgguggcagugucuc................................................    8     0    S01
........................................auuaauggcucgguggcagugucucgccu............................................    1     0    S01
.........................................uuaauggcucgguggcagugucucgccu............................................    1     0    S01
```

```
                                    Star                              Mature
nnnnnnnnnuuaauauccuuuaauccuuuucgaaccagcaauuaauggcucgguggcagugucucgccugacaccaagucguuucuuugcuuuuagaccgaagggccagaguu
......................................................................acaccaagucguuucuuu.........................    5       0    S01
......................................................................acaccaagucguuucuuug........................    36      0    S01
......................................................................acaccaagucguuucuuugc.......................    15      0    S01
......................................................................acaccaagucguuucuuugcu......................    8       0    S01
......................................................................acaccaagucguuucuuugcuu.....................    1455    0    S01
......................................................................acaccaagucguuucuuugcuuu....................    2267    0    S01
......................................................................acaccaagucguuucuuugcuuuu...................    990     0    S01
......................................................................acaccaagucguuucuuugcuuuua..................    2       0    S01
.......................................................................caccaagucguuucuuugcuuu....................    4       0    S01
.......................................................................caccaagucguuucuuugcuuuu...................    3       0    S01
.........................................................................ccaagucguuucuuugcuu.....................    2       0    S01
.........................................................................ccaagucguuucuuugcuuu....................    5       0    S01
```

Provisional ID	:	Scaffold5285_44889
Score total	:	10060.2
Score for star read(s)	:	3.9
Score for read counts	:	10052.9
Score for mfe	:	1.8
Score for randfold	:	1.6
Score for cons. seed	:	
Total read count	:	19730
Mature read count	:	18850
Loop read count	:	0
Star read count	:	880

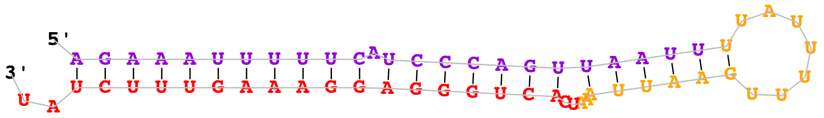

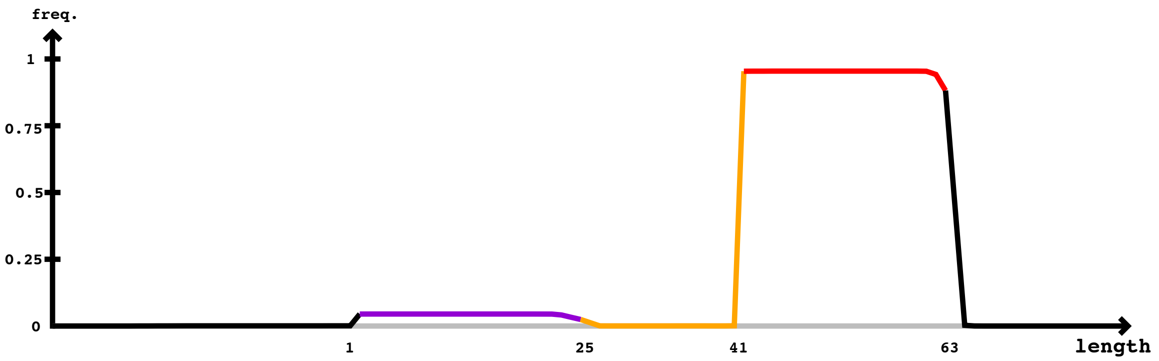


```
                                     Star                                        Mature
5'- aucaucaagaauaaagucuuggaguagaguagaaauuuuucaucccaguuaaauuuauuuuugaauuaaaucacugggaggaaaguuucuauuuugcucaagaaugacca  -3'   obs
    aucaucaagaauaaagucuuggaguagaguagaaauuuuucaucccaguuaaauuuauuuuugaauuaaaucacugggaggaaaguuucuauuuugcucaagaaugacca         exp
    .(((((((((......)))))((((((((((((((((((((.(((((((((((((.......))))))....)))))))))))))))))))))))))))....))))...   reads   mm   sample
    .......agaauaaagucuuggaguagagu................................................................................   2       0    S08
    ..............................agaaauuuuucaucccaguua...........................................................   2       0    S08
    ..............................agaaauuuuucaucccaguuaa..........................................................   21      0    S08
    ..............................agaaauuuuucaucccaguuaaau........................................................   16      0    S08
    ..............................agaaauuuuucaucccaguuaaauu.......................................................   53      0    S08
    ..............................agaaauuuuucaucccaguuaaauuu......................................................   1       0    S08
    ...............................gaaauuuuucaucccaguuaaauu.......................................................   1       0    S08
    .....................................................................aucacugggaggaaaguuucuauu.................   2       0    S08
    ......................................................................ucacugggaggaaaguuuc.....................   1       0    S08
    ......................................................................ucacugggaggaaaguuucu....................   63      0    S08
    ......................................................................ucacugggaggaaaguuucua...................   344     0    S08
    ......................................................................ucacugggaggaaaguuucuau..................   2688    0    S08
    ......................................................................ucacugggaggaaaguuucuauu.................   345     0    S08
    ......................................................................ucacugggaggaaaguuucuauuu................   4       0    S08
    ......................................................................ucacugggaggaaaguuucuauuuu...............   1       0    S08
    .......................................................................cacugggaggaaaguuucuau..................   1       0    S08
    .........................................................................cugggaggaaaguuucua...................   1       0    S08
    .........................................................................cugggaggaaaguuucuau..................   1       0    S08

    ..............................agaaauuuuucaucccaguuaa..........................................................   3       0    S03
    ..............................agaaauuuuucaucccaguuaaau........................................................   2       0    S03
    ..............................agaaauuuuucaucccaguuaaauu.......................................................   2       0    S03
    ..............................agaaauuuuucaucccaguuaaauuuauuuuugaauuaaa........................................   1       0    S03
    ......................................................................ucacugggaggaaaguuucu....................   5       0    S03
    ......................................................................ucacugggaggaaaguuucua...................   24      0    S03
    ......................................................................ucacugggaggaaaguuucuau..................   202     0    S03
    ......................................................................ucacugggaggaaaguuucuauu.................   45      0    S03

    .......agaauaaagucuuggaguagagu................................................................................   1       0    S09
    ........gaauaaagucuuggaguagagu................................................................................   1       0    S09
    .........aauaaagucuuggaguagagu................................................................................   2       0    S09
    ..........auaaagucuuggaguagagu................................................................................   1       0    S09
    ...........uaaagucuuggaguagagu................................................................................   2       0    S09
```

```
                              Star                                  Mature
aucaucaagaauaaagucuuggaguagaguagaaauuuuucaucccaguuaauuuuauuuuugaauuaaaucacugggaggaaaguuucuauuuugcucaagaaugacca
............aaagucuuggaguagagu................................................................................   1      0   S09
...........................aguagaaauuuuucaucccagu.............................................................   1      0   S09
..............................agaaauuuuucaucccagu.............................................................   1      0   S09
..............................agaaauuuuucaucccaguua...........................................................   27     0   S09
..............................agaaauuuuucaucccaguuaa..........................................................   68     0   S09
..............................agaaauuuuucaucccaguuaau.........................................................   74     0   S09
..............................agaaauuuuucaucccaguuaauu........................................................   189    0   S09
..............................agaaauuuuucaucccaguuaauuu.......................................................   7      0   S09
..............................agaaauuuuucaucccaguuaauuuuauuuuugaauuaaa........................................   1      0   S09
...............................gaaauuuuucaucccaguuaauu........................................................   1      0   S09
..................................auuuuucaucccaguuaa..........................................................   1      0   S09
.......................................................uauuuuugaauuaaaucacugggaggaaaguuucuau..................   1      0   S09
...............................................................aauuaaaucacugggaggaaaguuucuau..................   1      0   S09
.....................................................................aucacugggaggaaaguuucuau..................   1      0   S09
......................................................................ucacugggaggaaaguuuc.....................   6      0   S09
......................................................................ucacugggaggaaaguuucu....................   45     0   S09
......................................................................ucacugggaggaaaguuucua...................   184    0   S09
......................................................................ucacugggaggaaaguuucuau..................   3368   0   S09
......................................................................ucacugggaggaaaguuucuauu.................   598    0   S09
......................................................................ucacugggaggaaaguuucuauuu................   13     0   S09
.......................................................................cacugggaggaaaguuucuau..................   1      0   S09

...........................aguagaaauuuuucaucccagu.............................................................   1      0   S04
..............................agaaauuuuucaucccaguua...........................................................   1      0   S04
..............................agaaauuuuucaucccaguuaau.........................................................   2      0   S04
..............................agaaauuuuucaucccaguuaauu........................................................   6      0   S04
......................................................................ucacugggaggaaaguuucu....................   3      0   S04
......................................................................ucacugggaggaaaguuucua...................   39     0   S04
......................................................................ucacugggaggaaaguuucuau..................   280    0   S04
......................................................................ucacugggaggaaaguuucuauu.................   39     0   S04
......................................................................ucacugggaggaaaguuucuauuu................   3      0   S04

......................................................................ucacugggaggaaaguuucuau..................   3      0   S02

...........................aguagaaauuuuucaucccaguu............................................................   1      0   S10
..............................agaaauuuuucaucccaguua...........................................................   27     0   S10
..............................agaaauuuuucaucccaguuaa..........................................................   55     0   S10
..............................agaaauuuuucaucccaguuaau.........................................................   52     0   S10
..............................agaaauuuuucaucccaguuaauu........................................................   158    0   S10
..............................agaaauuuuucaucccaguuaauuu.......................................................   9      0   S10
..............................agaaauuuuucaucccaguuaauuuu......................................................   1      0   S10
..............................agaaauuuuucaucccaguuaauuuuauuuuugaauu...........................................   1      0   S10
..............................agaaauuuuucaucccaguuaauuuuauuuuugaauuaaa........................................   1      0   S10
.....................................................................aucacugggaggaaaguuucuauu.................   2      0   S10
......................................................................ucacugggaggaaaguuuc.....................   2      0   S10
......................................................................ucacugggaggaaaguuucu....................   67     0   S10
......................................................................ucacugggaggaaaguuucua...................   341    0   S10
......................................................................ucacugggaggaaaguuucuau..................   5262   0   S10
......................................................................ucacugggaggaaaguuucuauu.................   1060   0   S10
......................................................................ucacugggaggaaaguuucuauuu................   14     0   S10
......................................................................ucacugggaggaaaguuucuauuuu...............   2      0   S10
.......................................................................cacugggaggaaaguuucuau..................   1      0   S10
.......................................................................cacugggaggaaaguuucuauu.................   1      0   S10
.........................................................................cugggaggaaaguuucuau..................   1      0   S10

..............................agaaauuuuucaucccaguuaa..........................................................   1      0   S05
..............................agaaauuuuucaucccaguuaauu........................................................   6      0   S05
......................................................................ucacugggaggaaaguuuc.....................   2      0   S05
......................................................................ucacugggaggaaaguuucu....................   8      0   S05
......................................................................ucacugggaggaaaguuucua...................   37     0   S05
......................................................................ucacugggaggaaaguuucuau..................   382    0   S05
......................................................................ucacugggaggaaaguuucuauu.................   50     0   S05
......................................................................ucacugggaggaaaguuucuauuu................   2      0   S05

.........aauaaagucuuggaguagagu................................................................................   1      0   S07
..............................agaaauuuuucaucccaguua...........................................................   8      0   S07
..............................agaaauuuuucaucccaguuaa..........................................................   19     0   S07
..............................agaaauuuuucaucccaguuaau.........................................................   14     0   S07
..............................agaaauuuuucaucccaguuaauu........................................................   40     0   S07
..............................agaaauuuuucaucccaguuaauuu.......................................................   1      0   S07
```

```
                              Star                                    Mature
aucaucaagaauaaagucuuggaguagaguagaaauuuuucaucccaguuaauuuuauuuuugaauuaaaucacugggaggaaaguuucuauuuugcucaagaaugacca
.......................................................uauuuuugaauuaaaucacugggaggaaaguuucuau..................   1      0   S07
.....................................................................aucacugggaggaaaguuucuau..................   2      0   S07
.....................................................................aucacugggaggaaaguuucuauu.................   1      0   S07
......................................................................ucacugggaggaaaguuucu....................   38     0   S07
......................................................................ucacugggaggaaaguuucua...................   132    0   S07
......................................................................ucacugggaggaaaguuucuau..................   1883   0   S07
......................................................................ucacugggaggaaaguuucuauu.................   357    0   S07
......................................................................ucacugggaggaaaguuucuauuu................   7      0   S07

..............................agaaauuuuucaucccaguua...........................................................   1      0   S06
..............................agaaauuuuucaucccaguuaa..........................................................   2      0   S06
..............................agaaauuuuucaucccaguuaau.........................................................   3      0   S06
..............................agaaauuuuucaucccaguuaauu........................................................   5      0   S06
......................................................................ucacugggaggaaaguuucu....................   7      0   S06
......................................................................ucacugggaggaaaguuucua...................   90     0   S06
......................................................................ucacugggaggaaaguuucuau..................   652    0   S06
......................................................................ucacugggaggaaaguuucuauu.................   127    0   S06
......................................................................ucacugggaggaaaguuucuauuu................   7      0   S06
.......................................................................cacugggaggaaaguuucuau..................   1      0   S06
.........................................................................cugggaggaaaguuucuauuuu...............   1      0   S06

......................................................................ucacugggaggaaaguuucuau..................   1      0   S01
```

Provisional ID	:	Scaffold27700_47820
Score total	:	10060.2
Score for star read(s)	:	3.9
Score for read counts	:	10052.9
Score for mfe	:	1.8
Score for randfold	:	1.6
Score for cons. seed	:	
Total read count	:	19730
Mature read count	:	18850
Loop read count	:	0
Star read count	:	880

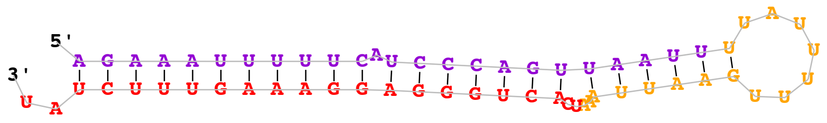

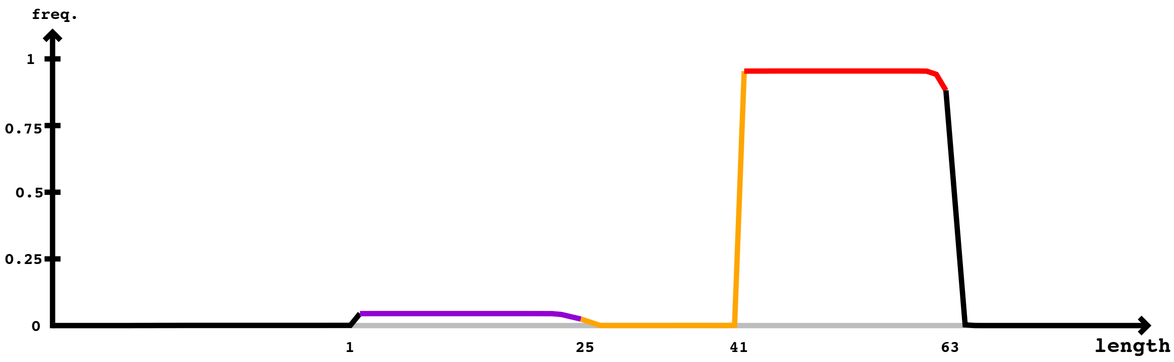

Star Mature

```
5'- aucaucaagaauaaagucuuggaguagaguagaaauuuuucaucccaguuaaauuuauuuuugaauuaaaucacugggaggaaaguuucuauuuugcucaagaaugaccagc  -3'   obs
    aucaucaagaauaaagucuuggaguagaguagaaauuuuucaucccaguuaaauuuauuuuugaauuaaaucacugggaggaaaguuucuauuuugcucaagaaugaccagc         exp
    .(((((((((......)))))((((((((((((((((((((.(((((((((((((.......))))))....)))))))))))))))))))))))))))....)))).....   reads   mm   sample
    ...........................aguagaaauuuuucaucccagu...............................................................   1       0    S04
    ..............................agaaauuuuucaucccaguua.............................................................   1       0    S04
    ..............................agaaauuuuucaucccaguuaaau..........................................................   2       0    S04
    ..............................agaaauuuuucaucccaguuaaauu.........................................................   6       0    S04
    ......................................................................ucacugggaggaaaguuucu......................   3       0    S04
    ......................................................................ucacugggaggaaaguuucua.....................   39      0    S04
    ......................................................................ucacugggaggaaaguuucuau....................   280     0    S04
    ......................................................................ucacugggaggaaaguuucuauu...................   39      0    S04
    ......................................................................ucacugggaggaaaguuucuauuu..................   3       0    S04

    ......................................................................ucacugggaggaaaguuucuau....................   3       0    S02

    .......agaauaaagucuuggaguagagu..................................................................................   1       0    S09
    ........gaauaaagucuuggaguagagu..................................................................................   1       0    S09
    .........aauaaagucuuggaguagagu..................................................................................   2       0    S09
    ..........auaaagucuuggaguagagu..................................................................................   1       0    S09
    ...........uaaagucuuggaguagagu..................................................................................   2       0    S09
    ............aaagucuuggaguagagu..................................................................................   1       0    S09
    ...........................aguagaaauuuuucaucccagu...............................................................   1       0    S09
    ..............................agaaauuuuucaucccagu...............................................................   1       0    S09
    ..............................agaaauuuuucaucccaguua.............................................................   27      0    S09
    ..............................agaaauuuuucaucccaguuaa............................................................   68      0    S09
    ..............................agaaauuuuucaucccaguuaaau..........................................................   74      0    S09
    ..............................agaaauuuuucaucccaguuaaauu.........................................................   189     0    S09
    ..............................agaaauuuuucaucccaguuaaauuu........................................................   7       0    S09
    ..............................agaaauuuuucaucccaguuaaauuuauuuuugaauuaaa..........................................   1       0    S09
    ...............................gaaauuuuucaucccaguuaaauu.........................................................   1       0    S09
    ..................................auuuuucaucccaguuaa............................................................   1       0    S09
    .......................................................uauuuuugaauuaaaucacugggaggaaaguuucuau....................   1       0    S09
    ...............................................................aauuaaaucacugggaggaaaguuucuau....................   1       0    S09
    .....................................................................aucacugggaggaaaguuucuau....................   1       0    S09
    ......................................................................ucacugggaggaaaguuuc.......................   6       0    S09
    ......................................................................ucacugggaggaaaguuucu......................   45      0    S09
```

Star **Mature**

```
aucaucaagaauaaagucuuggaguagaguagaaauuuuucaucccaguuaauuuuauuuuugaauuaaaucacugggaggaaaguuucuauuuugcucaagaaugaccagc
......................................................................ucacugggaggaaaguuucua.....................   184    0   S09
......................................................................ucacugggaggaaaguuucuau....................   3368   0   S09
......................................................................ucacugggaggaaaguuucuauu...................   598    0   S09
......................................................................ucacugggaggaaaguuucuauuu..................   13     0   S09
.......................................................................cacugggaggaaaguuucuau....................   1      0   S09

..............................agaaauuuuucaucccaguuaa............................................................   3      0   S03
..............................agaaauuuuucaucccaguuaau...........................................................   2      0   S03
..............................agaaauuuuucaucccaguuaauu..........................................................   2      0   S03
..............................agaaauuuuucaucccaguuaauuuuauuuuugaauuaaa..........................................   1      0   S03
......................................................................ucacugggaggaaaguuucu......................   5      0   S03
......................................................................ucacugggaggaaaguuucua.....................   24     0   S03
......................................................................ucacugggaggaaaguuucuau....................   202    0   S03
......................................................................ucacugggaggaaaguuucuauu...................   45     0   S03

.......agaauaaagucuuggaguagagu..................................................................................   2      0   S08
..............................agaaauuuuucaucccaguua.............................................................   2      0   S08
..............................agaaauuuuucaucccaguuaa............................................................   21     0   S08
..............................agaaauuuuucaucccaguuaau...........................................................   16     0   S08
..............................agaaauuuuucaucccaguuaauu..........................................................   53     0   S08
..............................agaaauuuuucaucccaguuaauuu.........................................................   1      0   S08
...............................gaaauuuuucaucccaguuaauu..........................................................   1      0   S08
.....................................................................aucacugggaggaaaguuucuauu...................   2      0   S08
......................................................................ucacugggaggaaaguuuc.......................   1      0   S08
......................................................................ucacugggaggaaaguuucu......................   63     0   S08
......................................................................ucacugggaggaaaguuucua.....................   344    0   S08
......................................................................ucacugggaggaaaguuucuau....................   2688   0   S08
......................................................................ucacugggaggaaaguuucuauu...................   345    0   S08
......................................................................ucacugggaggaaaguuucuauuu..................   4      0   S08
......................................................................ucacugggaggaaaguuucuauuuu.................   1      0   S08
.......................................................................cacugggaggaaaguuucuau....................   1      0   S08
.........................................................................cugggaggaaaguuucua.....................   1      0   S08
.........................................................................cugggaggaaaguuucuau....................   1      0   S08

......................................................................ucacugggaggaaaguuucuau....................   1      0   S01

..............................agaaauuuuucaucccaguua.............................................................   1      0   S06
..............................agaaauuuuucaucccaguuaa............................................................   2      0   S06
..............................agaaauuuuucaucccaguuaau...........................................................   3      0   S06
..............................agaaauuuuucaucccaguuaauu..........................................................   5      0   S06
......................................................................ucacugggaggaaaguuucu......................   7      0   S06
......................................................................ucacugggaggaaaguuucua.....................   90     0   S06
......................................................................ucacugggaggaaaguuucuau....................   652    0   S06
......................................................................ucacugggaggaaaguuucuauu...................   127    0   S06
......................................................................ucacugggaggaaaguuucuauuu..................   7      0   S06
.......................................................................cacugggaggaaaguuucuau....................   1      0   S06
.........................................................................cugggaggaaaguuucuauuu..................   1      0   S06

.........aauaaagucuuggaguagagu..................................................................................   1      0   S07
..............................agaaauuuuucaucccaguua.............................................................   8      0   S07
..............................agaaauuuuucaucccaguuaa............................................................   19     0   S07
..............................agaaauuuuucaucccaguuaau...........................................................   14     0   S07
..............................agaaauuuuucaucccaguuaauu..........................................................   40     0   S07
..............................agaaauuuuucaucccaguuaauuu.........................................................   1      0   S07
.......................................................uauuuuugaauuaaaucacugggaggaaaguuucuau....................   1      0   S07
.....................................................................aucacugggaggaaaguuucuau....................   2      0   S07
.....................................................................aucacugggaggaaaguuucuauu...................   1      0   S07
......................................................................ucacugggaggaaaguuucu......................   38     0   S07
......................................................................ucacugggaggaaaguuucua.....................   132    0   S07
......................................................................ucacugggaggaaaguuucuau....................   1883   0   S07
......................................................................ucacugggaggaaaguuucuauu...................   357    0   S07
......................................................................ucacugggaggaaaguuucuauuu..................   7      0   S07

..............................agaaauuuuucaucccaguuaa............................................................   1      0   S05
..............................agaaauuuuucaucccaguuaauu..........................................................   6      0   S05
......................................................................ucacugggaggaaaguuuc.......................   2      0   S05
......................................................................ucacugggaggaaaguuucu......................   8      0   S05
......................................................................ucacugggaggaaaguuucua.....................   37     0   S05
......................................................................ucacugggaggaaaguuucuau....................   382    0   S05
......................................................................ucacugggaggaaaguuucuauu...................   50     0   S05
```

Star Mature			
aucaucaagaauaaagucuuggaguagaguagaaauuuuucaucccaguuaauuuuauuuuugaauuaaaucacugggaggaaaguuucuauuuugcucaagaaugaccagc			
....ucacugggaggaaaguuucuauuu..................	2	0	S05
...........................aguagaaauuuuucaucccaguu..	1	0	S10
..............................agaaauuuuucaucccaguua...	27	0	S10
..............................agaaauuuuucaucccaguuaa..	55	0	S10
..............................agaaauuuuucaucccaguuaau...	52	0	S10
..............................agaaauuuuucaucccaguuaauu..	158	0	S10
..............................agaaauuuuucaucccaguuaauuu...	9	0	S10
..............................agaaauuuuucaucccaguuaauuuu..	1	0	S10
..............................agaaauuuuucaucccaguuaauuuuauuuuugaauu..	1	0	S10
..............................agaaauuuuucaucccaguuaauuuuauuuuugaauuaaa..	1	0	S10
....aucacugggaggaaaguuucuauu...................	2	0	S10
....ucacugggaggaaaguuuc.......................	2	0	S10
....ucacugggaggaaaguuucu......................	67	0	S10
....ucacugggaggaaaguuucua.....................	341	0	S10
....ucacugggaggaaaguuucuau....................	5262	0	S10
....ucacugggaggaaaguuucuauu...................	1060	0	S10
....ucacugggaggaaaguuucuauuu..................	14	0	S10
....ucacugggaggaaaguuucuauuuu.................	2	0	S10
....cacugggaggaaaguuucuau....................	1	0	S10
....cacugggaggaaaguuucuauu...................	1	0	S10
....cugggaggaaaguuucuau....................	1	0	S10

Provisional ID	:	Scaffold248_6722
Score total	:	10059
Score for star read(s)	:	3.9
Score for read counts	:	10052.9
Score for mfe	:	0.6
Score for randfold	:	1.6
Score for cons. seed	:	
Total read count	:	19730
Mature read count	:	18850
Loop read count	:	0
Star read count	:	880

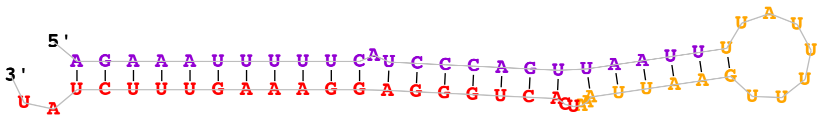

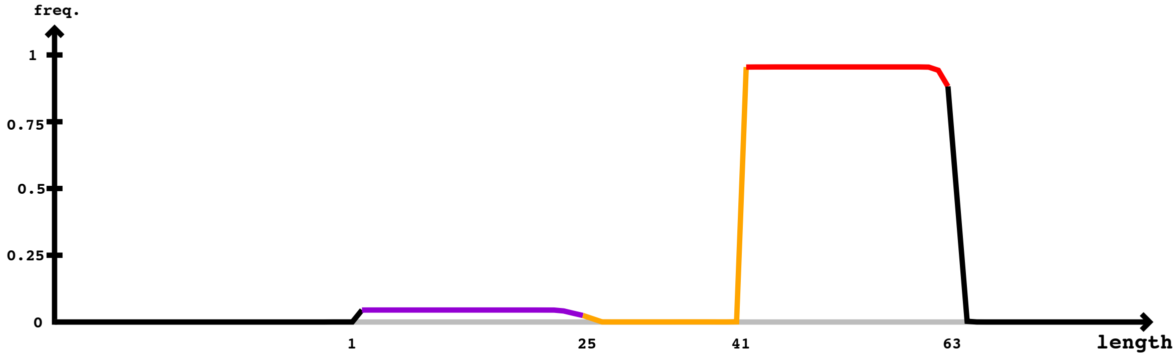

Star **Mature**

```
5'- nnnnnnnnnnnnnnnnnnnnnnaguagaguagaaauuuuucaucccaguuaauuuuauuuuugaauuaaaucacugggaggaaaguuucuauuuuauucaagagugaccagc  -3'   obs
    nnnnnnnnnnnnnnnnnnnnnnaguagaguagaaauuuuucaucccaguuaauuuuauuuuugaauuaaaucacugggaggaaaguuucuauuuuauucaagagugaccagc         exp
    ......................(((((((((((((((((((.(((((((((((((.......))))))....))))))))))))))))))))))))))..............    reads    mm    sample
    ......................................................................ucacugggaggaaaguuucuau....................    3        0     S02

    ...........................aguagaaauuuuucaucccagu...............................................................    1        0     S04
    ..............................agaaauuuuucaucccaguua.............................................................    1        0     S04
    ..............................agaaauuuuucaucccaguuaau...........................................................    2        0     S04
    ..............................agaaauuuuucaucccaguuaauu..........................................................    6        0     S04
    ......................................................................ucacugggaggaaaguuucu......................    3        0     S04
    ......................................................................ucacugggaggaaaguuucua.....................    39       0     S04
    ......................................................................ucacugggaggaaaguuucuau....................    280      0     S04
    ......................................................................ucacugggaggaaaguuucuauu...................    39       0     S04
    ......................................................................ucacugggaggaaaguuucuauuu..................    3        0     S04

    ..............................agaaauuuuucaucccaguua.............................................................    2        0     S08
    ..............................agaaauuuuucaucccaguuaa............................................................    21       0     S08
    ..............................agaaauuuuucaucccaguuaau...........................................................    16       0     S08
    ..............................agaaauuuuucaucccaguuaauu..........................................................    53       0     S08
    ..............................agaaauuuuucaucccaguuaauuu.........................................................    1        0     S08
    ...............................gaaauuuuucaucccaguuaauu..........................................................    1        0     S08
    .....................................................................aucacugggaggaaaguuucuau....................    2        0     S08
    ......................................................................ucacugggaggaaaguuuc.......................    1        0     S08
    ......................................................................ucacugggaggaaaguuucu......................    63       0     S08
    ......................................................................ucacugggaggaaaguuucua.....................    344      0     S08
    ......................................................................ucacugggaggaaaguuucuau....................    2688     0     S08
    ......................................................................ucacugggaggaaaguuucuauu...................    345      0     S08
    ......................................................................ucacugggaggaaaguuucuauuu..................    4        0     S08
    ......................................................................ucacugggaggaaaguuucuauuuu.................    1        0     S08
    .......................................................................cacugggaggaaaguuucuau....................    1        0     S08
    .........................................................................cugggaggaaaguuucua.....................    1        0     S08
    .........................................................................cugggaggaaaguuucuau....................    1        0     S08

    ..............................agaaauuuuucaucccaguuaa............................................................    3        0     S03
    ..............................agaaauuuuucaucccaguuaau...........................................................    2        0     S03
    ..............................agaaauuuuucaucccaguuaauu..........................................................    2        0     S03
```

Star Mature

```
nnnnnnnnnnnnnnnnnnnnnnaguagaguagaaauuuuucaucccaguuaauuuuauuuuugaauuaaaucacugggaggaaaguuucuauuuuauucaagagugaccagc
..............................agaaauuuuucaucccaguuaauuuuauuuuugaauuaaa..........................................   1      0    S03
......................................................................ucacugggaggaaaguuucu......................   5      0    S03
......................................................................ucacugggaggaaaguuucua.....................   24     0    S03
......................................................................ucacugggaggaaaguuucuau....................   202    0    S03
......................................................................ucacugggaggaaaguuucuauu...................   45     0    S03

...........................aguagaaauuuuucaucccagu...............................................................   1      0    S09
..............................agaaauuuuucaucccagu...............................................................   1      0    S09
..............................agaaauuuuucaucccaguua.............................................................   27     0    S09
..............................agaaauuuuucaucccaguuaa............................................................   68     0    S09
..............................agaaauuuuucaucccaguuaau...........................................................   74     0    S09
..............................agaaauuuuucaucccaguuaauu..........................................................   189    0    S09
..............................agaaauuuuucaucccaguuaauuu.........................................................   7      0    S09
..............................agaaauuuuucaucccaguuaauuuuauuuuugaauuaaa..........................................   1      0    S09
...............................gaaauuuuucaucccaguuaauu..........................................................   1      0    S09
..................................auuuuucaucccaguuaa............................................................   1      0    S09
.......................................................uauuuuugaauuaaaucacugggaggaaaguuucuau....................   1      0    S09
...............................................................aauuaaaucacugggaggaaaguuucuau....................   1      0    S09
.....................................................................aucacugggaggaaaguuucuau....................   1      0    S09
......................................................................ucacugggaggaaaguuuc.......................   6      0    S09
......................................................................ucacugggaggaaaguuucu......................   45     0    S09
......................................................................ucacugggaggaaaguuucua.....................   184    0    S09
......................................................................ucacugggaggaaaguuucuau....................   3368   0    S09
......................................................................ucacugggaggaaaguuucuauu...................   598    0    S09
......................................................................ucacugggaggaaaguuucuauuu..................   13     0    S09
.......................................................................cacugggaggaaaguuucuau....................   1      0    S09

..............................agaaauuuuucaucccaguua.............................................................   8      0    S07
..............................agaaauuuuucaucccaguuaa............................................................   19     0    S07
..............................agaaauuuuucaucccaguuaau...........................................................   14     0    S07
..............................agaaauuuuucaucccaguuaauu..........................................................   40     0    S07
..............................agaaauuuuucaucccaguuaauuu.........................................................   1      0    S07
.......................................................uauuuuugaauuaaaucacugggaggaaaguuucuau....................   1      0    S07
.....................................................................aucacugggaggaaaguuucuau....................   2      0    S07
.....................................................................aucacugggaggaaaguuucuauu...................   1      0    S07
......................................................................ucacugggaggaaaguuucu......................   38     0    S07
......................................................................ucacugggaggaaaguuucua.....................   132    0    S07
......................................................................ucacugggaggaaaguuucuau....................   1883   0    S07
......................................................................ucacugggaggaaaguuucuauu...................   357    0    S07
......................................................................ucacugggaggaaaguuucuauuu..................   7      0    S07

..............................agaaauuuuucaucccaguua.............................................................   1      0    S06
..............................agaaauuuuucaucccaguuaa............................................................   2      0    S06
..............................agaaauuuuucaucccaguuaau...........................................................   3      0    S06
..............................agaaauuuuucaucccaguuaauu..........................................................   5      0    S06
......................................................................ucacugggaggaaaguuucu......................   7      0    S06
......................................................................ucacugggaggaaaguuucua.....................   90     0    S06
......................................................................ucacugggaggaaaguuucuau....................   652    0    S06
......................................................................ucacugggaggaaaguuucuauu...................   127    0    S06
......................................................................ucacugggaggaaaguuucuauuu..................   7      0    S06
.......................................................................cacugggaggaaaguuucuau....................   1      0    S06
.........................................................................cugggaggaaaguuucuauuuu.................   1      0    S06

......................................................................ucacugggaggaaaguuucuau....................   1      0    S01

...........................aguagaaauuuuucaucccaguu..............................................................   1      0    S10
..............................agaaauuuuucaucccaguua.............................................................   27     0    S10
..............................agaaauuuuucaucccaguuaa............................................................   55     0    S10
..............................agaaauuuuucaucccaguuaau...........................................................   52     0    S10
..............................agaaauuuuucaucccaguuaauu..........................................................   158    0    S10
..............................agaaauuuuucaucccaguuaauuu.........................................................   9      0    S10
..............................agaaauuuuucaucccaguuaauuuu........................................................   1      0    S10
..............................agaaauuuuucaucccaguuaauuuuauuuuugaauu.............................................   1      0    S10
..............................agaaauuuuucaucccaguuaauuuuauuuuugaauuaaa..........................................   1      0    S10
.....................................................................aucacugggaggaaaguuucuauu...................   2      0    S10
......................................................................ucacugggaggaaaguuuc.......................   2      0    S10
......................................................................ucacugggaggaaaguuucu......................   67     0    S10
......................................................................ucacugggaggaaaguuucua.....................   341    0    S10
......................................................................ucacugggaggaaaguuucuau....................   5262   0    S10
......................................................................ucacugggaggaaaguuucuauu...................   1060   0    S10
```

```
                              Star                                    Mature
nnnnnnnnnnnnnnnnnnnnnnaguagaguagaaauuuuucaucccaguuaauuuuauuuuugaauuaaaucacugggaggaaaguuucuauuuuauucaagagugaccagc
......................................................................ucacugggaggaaaguuucuauuu..................    14      0      S10
......................................................................ucacugggaggaaaguuucuauuuu.................    2       0      S10
.......................................................................cacugggaggaaaguuucuau....................    1       0      S10
.......................................................................cacugggaggaaaguuucuauu...................    1       0      S10
.........................................................................cugggaggaaaguuucuau....................    1       0      S10

..............................agaaauuuuucaucccaguuaa............................................................    1       0      S05
..............................agaaauuuuucaucccaguuaauu..........................................................    6       0      S05
......................................................................ucacugggaggaaaguuuc.......................    2       0      S05
......................................................................ucacugggaggaaaguuucu......................    8       0      S05
......................................................................ucacugggaggaaaguuucua.....................    37      0      S05
......................................................................ucacugggaggaaaguuucuau....................    382     0      S05
......................................................................ucacugggaggaaaguuucuauu...................    50      0      S05
......................................................................ucacugggaggaaaguuucuauuu..................    2       0      S05
```

Provisional ID	:	Scaffold29666_47928
Score total	:	104153.9
Score for star read(s)	:	3.9
Score for read counts	:	104147.2
Score for mfe	:	1.2
Score for randfold	:	1.6
Score for cons. seed	:	
Total read count	:	204292
Mature read count	:	196460
Loop read count	:	3
Star read count	:	7829

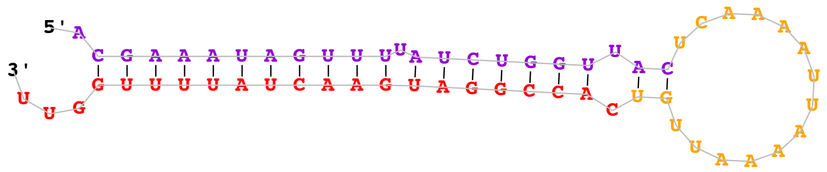

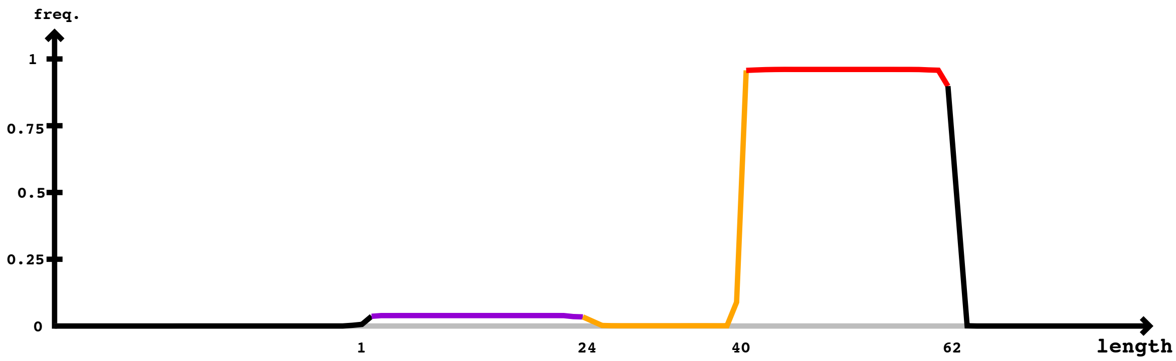

Star **Mature**

```
5'- aaauuacaaagcaaugaauaucuaaauggaaacgaaauaguuuuaucugguuacucaaaauuaaaaauugucaccggaugaacuauuuugguucuaugggauauuuuaucauca  -3'  obs
    aaauuacaaagcaaugaauaucuaaauggaaacgaaauaguuuuaucugguuacucaaaauuaaaaauugucaccggaugaacuauuuugguucuaugggauauuuuaucauca       exp
    .............((((((((((..((((((.(((((((((((.(((((((.((................)).)))))))))))))))))).)))))).))))))))))).....  reads  mm  sample
    ...............................acgaaauaguuuuaucugguuac............................................................  1      0   S02
    ..................................aaauaguuAuaucugguu..............................................................  2      1   S02
    ..................................aaauaguuAuaucugguua.............................................................  2      1   S02
    ......................................................................ucaccggaugaacuauuuuggu......................  2      0   S02
    .......................................................................caccggaugaacuauuuuggu......................  1      0   S02
    .......................................................................caccggaugaacuauuuugguu.....................  14     0   S02
    .......................................................................caccggaugaacuauuuugguuc....................  1      0   S02

    ........aagcaaugaauaucuaaauggaa...................................................................................  1      0   S04
    ..........gcaaugaauaucuaaaugga....................................................................................  1      0   S04
    .............................aaacgaaauaguuuuaucuggu...............................................................  1      0   S04
    .............................aaacgaaauaguuuuaucugguu..............................................................  39     0   S04
    ..............................aacgaaauaguuuuaucugguu..............................................................  7      0   S04
    ..............................aacgaaauaguuuuaucugguua.............................................................  4      0   S04
    ..............................aacgaaauaguuuuaucugguuac............................................................  19     0   S04
    ...............................acgaaauaguuuuaucugguu..............................................................  1      0   S04
    ...............................acgaaauaguuuuaucugguua.............................................................  3      0   S04
    ...............................acgaaauaguuuuaucugguuac............................................................  186    0   S04
    ...............................acgaaauaguuuuaucugguuacu...........................................................  9      0   S04
    ...............................acgaaauaguuuuaucugguuacuc..........................................................  4      0   S04
    ...............................acgaaauaguuuuaucugguuacuca.........................................................  2      0   S04
    ...............................acgaaauaguuuuaucugguuacucaaaauuaaaaauu.............................................  1      0   S04
    ...............................acgaaauaguuuuaucugguuacucaaaauuaaaaauugu...........................................  3      0   S04
    ................................cgaaauaguuuuaucugguuacu...........................................................  13     0   S04
    ................................cgaaauaguuuuaucugguuacuc..........................................................  4      0   S04
    ................................cgaaauaguuuuaucugguuacuca.........................................................  1      0   S04
    ............................................................uuaaaaauugucaccggaugaac...............................  1      0   S04
    .................................................................aauugucaccggaugaacu..............................  1      0   S04
    ......................................................................ucaccggaugaacuauuuugg.......................  3      0   S04
    ......................................................................ucaccggaugaacuauuuuggu......................  109    0   S04
    ......................................................................ucaccggaugaacuauuuugguu.....................  237    0   S04
    ......................................................................ucaccggaugaacuauuuugguuc....................  10     0   S04
    .......................................................................caccggaugaacuauuuu.........................  1      0   S04
```

Star **Mature**

```
aaauuacaaagcaaugaauaucuaaauggaaacgaaauaguuuuaucugguuacucaaaauuaaaauugucaccggaugaacuauuuugguucuaugggauauuuaucauca
......................................................................caccggaugaacuauuuug.......................  6     0  S04
......................................................................caccggaugaacuauuuugg......................  4     0  S04
......................................................................caccggaugaacuauuuuggu.....................  92    0  S04
......................................................................caccggaugaacuauuuugguu....................  3737  0  S04
......................................................................caccggaugaacuauuuugguuc...................  53    0  S04
......................................................................caccggaugaacuauuuugguucu..................  1     0  S04
.......................................................................accggaugaacuauuuugguu....................  8     0  S04
.......................................................................accggaugaacuauuuugguuc...................  4     0  S04
........................................................................ccggaugaacuauuuugguu....................  4     0  S04
........................................................................ccggaugaacuauuuugguuc...................  1     0  S04
........................................................................ccggaugaacuauuuugguucu..................  9     0  S04
.........................................................................cggaugaacuauuuuggu.....................  1     0  S04
.........................................................................cggaugaacuauuuugguu....................  2     0  S04
..........................................................................ggaugaacuauuuugguu....................  3     0  S04

aaauuacaaagcaaugaauaucuaaauggaa.................................................................................  1     0  S03
..........gcaaugaauaucuaaaugga..................................................................................  1     0  S03
...........caaugaauaucuaaauggaa.................................................................................  2     0  S03
.............................aaacgaaauaguuuuaucugguu............................................................  21    0  S03
.............................aaacgaaauaguuuuaucugguuac..........................................................  1     0  S03
..............................aacgaaauaguuuuaucugguu............................................................  2     0  S03
..............................aacgaaauaguuuuaucugguua...........................................................  2     0  S03
..............................aacgaaauaguuuuaucugguuac..........................................................  15    0  S03
...............................acgaaauaguuuuaucugguua...........................................................  4     0  S03
...............................acgaaauaguuuuaucugguuac..........................................................  125   0  S03
...............................acgaaauaguuuuaucugguuacu.........................................................  4     0  S03
...............................acgaaauaguuuuaucugguuacuc........................................................  2     0  S03
...............................acgaaauaguuuuaucugguuacucaaaauuaaaauu............................................  1     0  S03
...............................acgaaauaguuuuaucugguuacucaaaauuaaaauugu..........................................  4     0  S03
................................cgaaauaguuuuaucugguuacu.........................................................  6     0  S03
................................cgaaauaguuuuaucugguuacuc........................................................  1     0  S03
................................cgaaauaguuuuaucugguuacuca.......................................................  1     0  S03
................................cgaaauaguuuuaucugguuacucaaaauuaaaau.............................................  1     0  S03
.................................gaaauaguuuuaucugguuacu.........................................................  1     0  S03
...................................aauaguuuuaucugguuac..........................................................  1     0  S03
.............................................................uaaaauugucaccggaugaacuauuuugguu....................  1     0  S03
..................................................................uugucaccggaugaacuauuuugguu....................  1     0  S03
.....................................................................ucaccggaugaacuauuu.........................  1     0  S03
.....................................................................ucaccggaugaacuauuuug.......................  3     0  S03
.....................................................................ucaccggaugaacuauuuugg......................  1     0  S03
.....................................................................ucaccggaugaacuauuuuggu.....................  52    0  S03
.....................................................................ucaccggaugaacuauuuugguu....................  136   0  S03
.....................................................................ucaccggaugaacuauuuugguuc...................  3     0  S03
......................................................................caccggaugaacuauuu.........................  3     0  S03
......................................................................caccggaugaacuauuuug.......................  6     0  S03
......................................................................caccggaugaacuauuuugg......................  1     0  S03
......................................................................caccggaugaacuauuuuggu.....................  55    0  S03
......................................................................caccggaugaacuauuuugguu....................  2499  0  S03
......................................................................caccggaugaacuauuuugguuc...................  32    0  S03
.......................................................................accggaugaacuauuuug.......................  1     0  S03
.......................................................................accggaugaacuauuuugg......................  1     0  S03
.......................................................................accggaugaacuauuuugguuc...................  4     0  S03
.......................................................................accggaugaacuauuuugguucu..................  1     0  S03
........................................................................ccggaugaacuauuuugguu....................  5     0  S03
........................................................................ccggaugaacuauuuugguuc...................  6     0  S03
........................................................................ccggaugaacuauuuugguucu..................  12    0  S03
..........................................................................ggaugaacuauuuugguu....................  2     0  S03

.aauuacaaagcaaugaauaucuaaauggaa.................................................................................  1     0  S09
........aagcaaugaauaucuaaauggaa.................................................................................  1     0  S09
.............................aaacgaaauaguuuuaucuggu.............................................................  1     0  S09
.............................aaacgaaauaguuuuaucugguu............................................................  99    0  S09
.............................aaacgaaauaguuuuaucugguuac..........................................................  1     0  S09
.............................aaacgaaauaguuuuaucugguuacu.........................................................  1     0  S09
..............................aacgaaauaguuuuaucuggu.............................................................  1     0  S09
..............................aacgaaauaguuuuaucugguu............................................................  60    0  S09
..............................aacgaaauaguuuuaucugguua...........................................................  39    0  S09
..............................aacgaaauaguuuuaucugguuac..........................................................  88    0  S09
..............................aacgaaauaguuuuaucugguuacu.........................................................  4     0  S09
..............................aacgaaauaguuuuaucugguuacuc........................................................  1     0  S09
```

Star **Mature**

```
aaauuacaaagcaaugaauaucuaaauggaaacgaaauaguuuuaucugguuacucaaaauuaaaauugucaccggaugaacuauuuugguucuaugggauauuuaucauca
...............................acgaaauaguuuuaucug............................................................... 1 0 S09
...............................acgaaauaguuuuaucugguu............................................................ 2 0 S09
...............................acgaaauaguuuuaucugguua........................................................... 36 0 S09
...............................acgaaauaguuuuaucugguuac.......................................................... 1366 0 S09
...............................acgaaauaguuuuaucugguuacu......................................................... 39 0 S09
...............................acgaaauaguuuuaucugguuacuc........................................................ 7 0 S09
...............................acgaaauaguuuuaucugguuacuca....................................................... 2 0 S09
...............................acgaaauaguuuuaucugguuacucaaaauuaaaau............................................. 2 0 S09
...............................acgaaauaguuuuaucugguuacucaaaauuaaaauugu.......................................... 18 0 S09
................................cgaaauaguuuuaucugguuac.......................................................... 12 0 S09
................................cgaaauaguuuuaucugguuacu......................................................... 78 0 S09
................................cgaaauaguuuuaucugguuacuc........................................................ 28 0 S09
................................cgaaauaguuuuaucugguuacuca....................................................... 3 0 S09
.................................gaaauaguuuuaucugguuac.......................................................... 1 0 S09
..................................aaauaguuAuaucugguu............................................................ 3 1 S09
....................................auaguuuuaucugguuac.......................................................... 1 0 S09
.....................................................cucaaaauuaaaauugucaccggaugaacuauuuugguu.................... 1 0 S09
......................................................ucaaaauuaaaauugucaccggaugaac.............................. 1 0 S09
........................................................aaaauuaaaauugucaccggau.................................. 1 0 S09
............................................................uuaaaauugucaccggaugaacu............................. 1 0 S09
............................................................uuaaaauugucaccggaugaacuauuuugguu.................... 2 0 S09
....................................................................gucaccggaugaacuauuuugguu.................... 1 0 S09
.....................................................................ucaccggaugaacuauuu......................... 8 0 S09
.....................................................................ucaccggaugaacuauuuug....................... 7 0 S09
.....................................................................ucaccggaugaacuauuuugg...................... 30 0 S09
.....................................................................ucaccggaugaacuauuuuggu..................... 2302 0 S09
.....................................................................ucaccggaugaacuauuuugguu.................... 3653 0 S09
.....................................................................ucaccggaugaacuauuuugguuc................... 63 0 S09
.....................................................................ucaccggaugaacuauuuugguucu.................. 1 0 S09
......................................................................caccggaugaacuauuu......................... 9 0 S09
......................................................................caccggaugaacuauuuug....................... 74 0 S09
......................................................................caccggaugaacuauuuugg...................... 30 0 S09
......................................................................caccggaugaacuauuuuggu..................... 2303 0 S09
......................................................................caccggaugaacuauuuugguu.................... 51824 0 S09
......................................................................caccggaugaacuauuuugguuc................... 536 0 S09
......................................................................caccggaugaacuauuuugguucu.................. 7 0 S09
.......................................................................accggaugaacuauuuug....................... 4 0 S09
.......................................................................accggaugaacuauuuugg...................... 1 0 S09
.......................................................................accggaugaacuauuuuggu..................... 1 0 S09
.......................................................................accggaugaacuauuuugguu.................... 58 0 S09
.......................................................................accggaugaacuauuuugguuc................... 19 0 S09
.......................................................................accggaugaacuauuuugguucu.................. 4 0 S09
........................................................................ccggaugaacuauuuuggu..................... 1 0 S09
........................................................................ccggaugaacuauuuugguu.................... 20 0 S09
........................................................................ccggaugaacuauuuugguuc................... 8 0 S09
........................................................................ccggaugaacuauuuugguucu.................. 12 0 S09
.........................................................................cggaugaacuauuuuggu..................... 3 0 S09
.........................................................................cggaugaacuauuuugguu.................... 24 0 S09
..........................................................................ggaugaacuauuuugguu.................... 14 0 S09

......caaagcaaugaauaucuaaaugga.................................................................................. 1 0 S08
...........caaugaauaucuaaauggaa................................................................................. 2 0 S08
............aaugaauaucuaaauggaaa................................................................................ 1 0 S08
.............................aaacgaaauaguuuuaucuggu............................................................. 1 0 S08
.............................aaacgaaauaguuuuaucugguu............................................................ 60 0 S08
.............................aaacgaaauaguuuuaucugguuac.......................................................... 1 0 S08
..............................aacgaaauaguuuuaucugguu............................................................ 49 0 S08
..............................aacgaaauaguuuuaucugguua........................................................... 18 0 S08
..............................aacgaaauaguuuuaucugguuac.......................................................... 42 0 S08
...............................acgaaauaguuuuaucug............................................................... 1 0 S08
...............................acgaaauaguuuuaucugguu............................................................ 4 0 S08
...............................acgaaauaguuuuaucugguua........................................................... 9 0 S08
...............................acgaaauaguuuuaucugguuac.......................................................... 732 0 S08
...............................acgaaauaguuuuaucugguuacu......................................................... 15 0 S08
...............................acgaaauaguuuuaucugguuacuc........................................................ 7 0 S08
...............................acgaaauaguuuuaucugguuacucaaaauu.................................................. 3 0 S08
...............................acgaaauaguuuuaucugguuacucaaaauua................................................. 1 0 S08
...............................acgaaauaguuuuaucugguuacucaaaauuaaaau............................................. 1 0 S08
...............................acgaaauaguuuuaucugguuacucaaaauuaaaauu............................................ 2 0 S08
...............................acgaaauaguuuuaucugguuacucaaaauuaaaauug........................................... 1 0 S08
```

Star Mature

```
aaauuacaaagcaaugaauaucuaaauggaaacgaaauaguuuuaucugguuacucaaaauuaaaauugucaccggaugaacuauuuugguucuaugggauauuuaucauca
...............................acgaaauaguuuuaucugguuacucaaaauuaaaauugu..........................................  19     0  S08
................................cgaaauaguuuuaucugguuac..........................................................  5      0  S08
................................cgaaauaguuuuaucugguuacu.........................................................  49     0  S08
................................cgaaauaguuuuaucugguuacuc........................................................  13     0  S08
................................cgaaauaguuuuaucugguuacuca.......................................................  6      0  S08
................................cgaaauaguuuuaucugguuacucaaaauuaaaauu............................................  4      0  S08
................................cgaaauaguuuuaucugguuacucaaaauuaaaauugu..........................................  1      0  S08
.................................gaaauaguuuuaucugguuac..........................................................  1      0  S08
..................................aaauaguuuuaucugguuac..........................................................  1      0  S08
...................................aauaguuuuaucugguuac..........................................................  2      0  S08
.........................................uuuaucugguuacucaaaauuaaaauugu..........................................  1      0  S08
...................................................uacucaaaauuaaaauugucaccggaugaacuauuuugguu....................  3      0  S08
.....................................................cucaaaauuaaaauugucaccgg....................................  1      0  S08
......................................................ucaaaauuaaaauugucaccggaugaacu.............................  1      0  S08
.........................................................aaauuaaaauugucaccggaugaacuauuuugguu....................  1      0  S08
................................................................aauugucaccggaugaacuauuuugguu....................  2      0  S08
...................................................................ugucaccggaugaacuauuuugguu....................  2      0  S08
.....................................................................ucaccggaugaacuauuu.........................  1      0  S08
.....................................................................ucaccggaugaacuauuuug.......................  3      0  S08
.....................................................................ucaccggaugaacuauuuugg......................  8      0  S08
.....................................................................ucaccggaugaacuauuuuggu.....................  443    0  S08
.....................................................................ucaccggaugaacuauuuugguu....................  1206   0  S08
.....................................................................ucaccggaugaacuauuuugguuc...................  19     0  S08
......................................................................caccggaugaacuauuuu........................  9      0  S08
......................................................................caccggaugaacuauuuug.......................  15     0  S08
......................................................................caccggaugaacuauuuugg......................  10     0  S08
......................................................................caccggaugaacuauuuuggu.....................  453    0  S08
......................................................................caccggaugaacuauuuugguu....................  14088  0  S08
......................................................................caccggaugaacuauuuugguuc...................  198    0  S08
......................................................................caccggaugaacuauuuugguucu..................  9      0  S08
.......................................................................accggaugaacuauuuugguu....................  19     0  S08
.......................................................................accggaugaacuauuuugguuc...................  8      0  S08
.......................................................................accggaugaacuauuuugguucu..................  3      0  S08
........................................................................ccggaugaacuauuuuggu.....................  11     0  S08
........................................................................ccggaugaacuauuuugguu....................  30     0  S08
........................................................................ccggaugaacuauuuugguuc...................  23     0  S08
........................................................................ccggaugaacuauuuugguucu..................  23     0  S08
.........................................................................cggaugaacuauuuugguu....................  45     0  S08
.........................................................................cggaugaacuauuuugguuc...................  1      0  S08
..........................................................................ggaugaacuauuuugguu....................  10     0  S08

........aagcaaugaauaucuaaaugga..................................................................................  1      0  S06
.............................aaacgaaauaguuuuaucuggu.............................................................  3      0  S06
.............................aaacgaaauaguuuuaucugguu............................................................  54     0  S06
.............................aaacgaaauaguuuuaucugguuac..........................................................  5      0  S06
..............................aacgaaauaguuuuaucugguu............................................................  17     0  S06
..............................aacgaaauaguuuuaucugguua...........................................................  10     0  S06
..............................aacgaaauaguuuuaucugguuac..........................................................  29     0  S06
...............................acgaaauaguuuuaucugguu............................................................  2      0  S06
...............................acgaaauaguuuuaucugguua...........................................................  4      0  S06
...............................acgaaauaguuuuaucugguuac..........................................................  583    0  S06
...............................acgaaauaguuuuaucugguuacu.........................................................  13     0  S06
...............................acgaaauaguuuuaucugguuacuc........................................................  9      0  S06
...............................acgaaauaguuuuaucugguuacuca.......................................................  1      0  S06
...............................acgaaauaguuuuaucugguuacucaaaauuaaaauugu..........................................  12     0  S06
................................cgaaauaguuuuaucugguu............................................................  1      0  S06
................................cgaaauaguuuuaucugguuac..........................................................  4      0  S06
................................cgaaauaguuuuaucugguuacu.........................................................  30     0  S06
................................cgaaauaguuuuaucugguuacuc........................................................  6      0  S06
................................cgaaauaguuuuaucugguuacuca.......................................................  5      0  S06
.................................gaaauaguuuuaucugguuacuc........................................................  1      0  S06
...................................................................ugucaccggaugaacuauuuugguuc...................  1      0  S06
....................................................................gucaccggaugaacuauuuugguu....................  1      0  S06
.....................................................................ucaccggaugaacuauuu.........................  1      0  S06
.....................................................................ucaccggaugaacuauuuu........................  2      0  S06
.....................................................................ucaccggaugaacuauuuug.......................  2      0  S06
.....................................................................ucaccggaugaacuauuuugg......................  5      0  S06
.....................................................................ucaccggaugaacuauuuuggu.....................  138    0  S06
.....................................................................ucaccggaugaacuauuuugguu....................  472    0  S06
.....................................................................ucaccggaugaacuauuuugguuc...................  4      0  S06
```

```
                              Star                                  Mature
aaauuacaaagcaaugaauaucuaaauggaaacgaaauaguuuuaucugguuacucaaaauuaaaauugucaccggaugaacuauuuugguucuaugggauauuuaucauca
.....................................................................ucaccggaugaacuauuuugguucu..................    1      0    S06
......................................................................caccggaugaacuauuuu........................    5      0    S06
......................................................................caccggaugaacuauuuug.......................    8      0    S06
......................................................................caccggaugaacuauuuugg......................    4      0    S06
......................................................................caccggaugaacuauuuuggu.....................    188    0    S06
......................................................................caccggaugaacuauuuugguu....................    6221   0    S06
......................................................................caccggaugaacuauuuugguuc...................    91     0    S06
......................................................................caccggaugaacuauuuugguucu..................    2      0    S06
.......................................................................accggaugaacuauuuug.......................    1      0    S06
.......................................................................accggaugaacuauuuugguu....................    6      0    S06
.......................................................................accggaugaacuauuuugguuc...................    7      0    S06
.......................................................................accggaugaacuauuuugguucu..................    3      0    S06
........................................................................ccggaugaacuauuuugguu....................    2      0    S06
........................................................................ccggaugaacuauuuugguuc...................    3      0    S06
........................................................................ccggaugaacuauuuugguucu..................    3      0    S06
.........................................................................cggaugaacuauuuugguu....................    4      0    S06
.........................................................................cggaugaacuauuuugguuc...................    1      0    S06
.........................................................................cggaugaacuauuuugguucu..................    1      0    S06
..........................................................................ggaugaacuauuuugguu....................    1      0    S06

...............................acgaaauaguuuuaucugguuac..........................................................    1      0    S01
.....................................................................ucaccggaugaacuauuuugguu....................    1      0    S01
......................................................................caccggaugaacuauuuugguu....................    8      0    S01

.............................aaacgaaauaguuuuaucuggu.............................................................    1      0    S07
.............................aaacgaaauaguuuuaucugguu............................................................    49     0    S07
.............................aaacgaaauaguuuuaucugguuacu.........................................................    1      0    S07
..............................aacgaaauaguuuuaucugguu............................................................    10     0    S07
..............................aacgaaauaguuuuaucugguua...........................................................    9      0    S07
..............................aacgaaauaguuuuaucugguuac..........................................................    33     0    S07
..............................aacgaaauaguuuuaucugguuacu.........................................................    1      0    S07
..............................aacgaaauaguuuuaucugguuacuc........................................................    1      0    S07
...............................acgaaauaguuuuaucugguu............................................................    1      0    S07
...............................acgaaauaguuuuaucugguua...........................................................    12     0    S07
...............................acgaaauaguuuuaucugguuac..........................................................    693    0    S07
...............................acgaaauaguuuuaucugguuacu.........................................................    23     0    S07
...............................acgaaauaguuuuaucugguuacuc........................................................    14     0    S07
...............................acgaaauaguuuuaucugguuacuca.......................................................    1      0    S07
...............................acgaaauaguuuuaucugguuacucaaaauua.................................................    2      0    S07
...............................acgaaauaguuuuaucugguuacucaaaauuaaaau.............................................    2      0    S07
...............................acgaaauaguuuuaucugguuacucaaaauuaaaauugu..........................................    35     0    S07
................................cgaaauaguuuuaucugguuac..........................................................    2      0    S07
................................cgaaauaguuuuaucugguuacu.........................................................    28     0    S07
................................cgaaauaguuuuaucugguuacuc........................................................    23     0    S07
................................cgaaauaguuuuaucugguuacuca.......................................................    11     0    S07
................................cgaaauaguuuuaucugguuacucaaaauuaaaau.............................................    1      0    S07
................................cgaaauaguuuuaucugguuacucaaaauuaaaauu............................................    1      0    S07
................................cgaaauaguuuuaucugguuacucaaaauuaaaauug...........................................    3      0    S07
................................cgaaauaguuuuaucugguuacucaaaauuaaaauugu..........................................    1      0    S07
.................................gaaauaguuuuaucugguuac..........................................................    1      0    S07
.................................gaaauaguuuuaucugguuacu.........................................................    1      0    S07
.................................gaaauaguuuuaucugguuacuc........................................................    3      0    S07
..................................aaauaguuuuaucugguuacuc........................................................    1      0    S07
.....................................uaguuuuaucugguuacu.........................................................    1      0    S07
.....................................................cucaaaauuaaaauugucaccg.....................................    2      0    S07
.......................................................caaaauuaaaauugucaccggau..................................    2      0    S07
...........................................................auuaaaauugucaccggaugaacuauuuugguu....................    1      0    S07
............................................................uuaaaauugucaccggaugaacuauuuugguu....................    2      0    S07
.............................................................uaaaauugucaccggaugaacuauuuugguu....................    1      0    S07
....................................................................gucaccggaugaacuauuuugguu....................    1      0    S07
.....................................................................ucaccggaugaacuauuu.........................    2      0    S07
.....................................................................ucaccggaugaacuauuuug.......................    7      0    S07
.....................................................................ucaccggaugaacuauuuugg......................    29     0    S07
.....................................................................ucaccggaugaacuauuuuggu.....................    850    0    S07
.....................................................................ucaccggaugaacuauuuugguu....................    2036   0    S07
.....................................................................ucaccggaugaacuauuuugguuc...................    28     0    S07
.....................................................................ucaccggaugaacuauuuugguucu..................    1      0    S07
......................................................................caccggaugaacuauuuu........................    6      0    S07
......................................................................caccggaugaacuauuuug.......................    60     0    S07
......................................................................caccggaugaacuauuuugg......................    20     0    S07
```

```
                              Star                                   Mature
aaauuacaaagcaaugaauaucuaaauggaaacgaaauaguuuuaucugguuacucaaaauuaaaauugucaccggaugaacuauuuugguucuaugggauauuuaucauca
......................................................................caccggaugaacuauuuuggu.....................  890    0  S07
......................................................................caccggaugaacuauuuugguu....................  33079  0  S07
......................................................................caccggaugaacuauuuugguuc...................  316    0  S07
......................................................................caccggaugaacuauuuugguucu..................  5      0  S07
.......................................................................accggaugaacuauuuug.......................  1      0  S07
.......................................................................accggaugaacuauuuugg......................  1      0  S07
.......................................................................accggaugaacuauuuuggu.....................  2      0  S07
.......................................................................accggaugaacuauuuugguu....................  24     0  S07
.......................................................................accggaugaacuauuuugguuc...................  9      0  S07
.......................................................................accggaugaacuauuuugguucu..................  6      0  S07
........................................................................ccggaugaacuauuuuggu.....................  1      0  S07
........................................................................ccggaugaacuauuuugguu....................  10     0  S07
........................................................................ccggaugaacuauuuugguuc...................  13     0  S07
........................................................................ccggaugaacuauuuugguucu..................  15     0  S07
.........................................................................cggaugaacuauuuuggu.....................  3      0  S07
.........................................................................cggaugaacuauuuugguu....................  6      0  S07
.........................................................................cggaugaacuauuuugguuc...................  1      0  S07
..........................................................................ggaugaacuauuuugguu....................  5      0  S07

.............................aaacgaaauaguuuuaucugguu............................................................  51     0  S05
..............................aacgaaauaguuuuaucugguu............................................................  6      0  S05
..............................aacgaaauaguuuuaucugguua...........................................................  5      0  S05
..............................aacgaaauaguuuuaucugguuac..........................................................  20     0  S05
..............................aacgaaauaguuuuaucugguuacu.........................................................  2      0  S05
...............................acgaaauaguuuuaucugguu............................................................  1      0  S05
...............................acgaaauaguuuuaucugguua...........................................................  2      0  S05
...............................acgaaauaguuuuaucugguuac..........................................................  238    0  S05
...............................acgaaauaguuuuaucugguuacu.........................................................  3      0  S05
...............................acgaaauaguuuuaucugguuacuc........................................................  2      0  S05
...............................acgaaauaguuuuaucugguuacucaaaauuaaaauugu..........................................  2      0  S05
................................cgaaauaguuuuaucugguuacu.........................................................  11     0  S05
................................cgaaauaguuuuaucugguuacuc........................................................  6      0  S05
................................cgaaauaguuuuaucugguuacuca.......................................................  2      0  S05
..................................aaauaguuAuaucugguua...........................................................  1      1  S05
.....................................................................ucaccggaugaacuauuu.........................  1      0  S05
.....................................................................ucaccggaugaacuauuuug.......................  3      0  S05
.....................................................................ucaccggaugaacuauuuugg......................  2      0  S05
.....................................................................ucaccggaugaacuauuuuggu.....................  97     0  S05
.....................................................................ucaccggaugaacuauuuugguu....................  318    0  S05
.....................................................................ucaccggaugaacuauuuugguuc...................  10     0  S05
......................................................................caccggaugaacuauuu.........................  1      0  S05
......................................................................caccggaugaacuauuuug.......................  3      0  S05
......................................................................caccggaugaacuauuuugg......................  4      0  S05
......................................................................caccggaugaacuauuuuggu.....................  88     0  S05
......................................................................caccggaugaacuauuuugguu....................  4470   0  S05
......................................................................caccggaugaacuauuuugguuc...................  79     0  S05
......................................................................caccggaugaacuauuuugguucu..................  1      0  S05
.......................................................................accggaugaacuauuuuggu.....................  1      0  S05
.......................................................................accggaugaacuauuuugguu....................  7      0  S05
.......................................................................accggaugaacuauuuugguuc...................  9      0  S05
.......................................................................accggaugaacuauuuugguucu..................  2      0  S05
........................................................................ccggaugaacuauuuugguu....................  3      0  S05
........................................................................ccggaugaacuauuuugguuc...................  6      0  S05
........................................................................ccggaugaacuauuuugguucu..................  6      0  S05
.........................................................................cggaugaacuauuuugguu....................  4      0  S05
..........................................................................ggaugaacuauuuugguu....................  2      0  S05

.............................aaacgaaauaguuuuaucuggu.............................................................  2      0  S10
.............................aaacgaaauaguuuuaucugguu............................................................  124    0  S10
.............................aaacgaaauaguuuuaucugguua...........................................................  1      0  S10
.............................aaacgaaauaguuuuaucugguuac..........................................................  3      0  S10
..............................aacgaaauaguuuuaucuggu.............................................................  1      0  S10
..............................aacgaaauaguuuuaucugguu............................................................  90     0  S10
..............................aacgaaauaguuuuaucugguua...........................................................  47     0  S10
..............................aacgaaauaguuuuaucugguuac..........................................................  100    0  S10
..............................aacgaaauaguuuuaucugguuacu.........................................................  3      0  S10
...............................acgaaauaguuuuaucug...............................................................  2      0  S10
...............................acgaaauaguuuuaucuggu.............................................................  2      0  S10
...............................acgaaauaguuuuaucugguu............................................................  5      0  S10
...............................acgaaauaguuuuaucugguua...........................................................  34     0  S10
```

Star **Mature**

```
aaauuacaaagcaaugaauaucuaaauggaaacgaaauaguuuuaucugguuacucaaaauuaaaaauugucaccggaugaacuauuuugguucuaugggauauuuaucauca
...............................acgaaauaguuuuaucugguuac...........................................................  1758   0   S10
...............................acgaaauaguuuuaucugguuacu..........................................................  61     0   S10
...............................acgaaauaguuuuaucugguuacuc.........................................................  14     0   S10
...............................acgaaauaguuuuaucugguuacuca........................................................  4      0   S10
...............................acgaaauaguuuuaucugguuacucaaaauuaaaaau.............................................  1      0   S10
...............................acgaaauaguuuuaucugguuacucaaaauuaaaaauu............................................  4      0   S10
...............................acgaaauaguuuuaucugguuacucaaaauuaaaaauugu..........................................  31     0   S10
...............................acgaaauaguuuuaucugguuacucaaaauuaaaaauugucaccg.....................................  1      0   S10
................................cgaaauaguuuuaucugg...............................................................  1      0   S10
................................cgaaauaguuuuaucugguua............................................................  1      0   S10
................................cgaaauaguuuuaucugguuac...........................................................  13     0   S10
................................cgaaauaguuuuaucugguuacu..........................................................  98     0   S10
................................cgaaauaguuuuaucugguuacuc.........................................................  32     0   S10
................................cgaaauaguuuuaucugguuacuca........................................................  8      0   S10
................................cgaaauaguuuuaucugguuacucaaaauuaaaaau.............................................  6      0   S10
.................................gaaauaguuuuaucugguuacu..........................................................  1      0   S10
..................................aaauaguuuuaucugguua............................................................  1      0   S10
....................................auaguuuuaucugguuac...........................................................  1      0   S10
.....................................................cucaaaauuaaaaauugucaccg.....................................  1      0   S10
.......................................................caaaauuaaaaauugucaccggaug.................................  1      0   S10
.........................................................aaauuaaaaauugucaccggaug.................................  1      0   S10
............................................................uuaaaaauugucaccggaug.................................  1      0   S10
............................................................uuaaaaauugucaccggaugaac..............................  3      0   S10
................................................................aaauugucaccggaugaacuauu..........................  1      0   S10
.................................................................aauugucaccggaugaacu.............................  1      0   S10
.................................................................aauugucaccggaugaacuauuuugguu....................  2      0   S10
..................................................................auugucaccggaugaacu.............................  1      0   S10
....................................................................ugucaccggaugaacuauuuug.......................  1      0   S10
....................................................................ugucaccggaugaacuauuuugguu....................  1      0   S10
......................................................................ucaccggaugaacuauuu.........................  2      0   S10
......................................................................ucaccggaugaacuauuuug.......................  10     0   S10
......................................................................ucaccggaugaacuauuuugg......................  36     0   S10
......................................................................ucaccggaugaacuauuuuggu.....................  2167   0   S10
......................................................................ucaccggaugaacuauuuugguu....................  3455   0   S10
......................................................................ucaccggaugaacuauuuugguuc...................  62     0   S10
......................................................................ucaccggaugaacuauuuugguucu..................  2      0   S10
.......................................................................caccggaugaacuauuuu........................  11     0   S10
.......................................................................caccggaugaacuauuuug.......................  90     0   S10
.......................................................................caccggaugaacuauuuugg......................  34     0   S10
.......................................................................caccggaugaacuauuuuggu.....................  1937   0   S10
.......................................................................caccggaugaacuauuuugguu....................  53494  0   S10
.......................................................................caccggaugaacuauuuugguuc...................  472    0   S10
.......................................................................caccggaugaacuauuuugguucu..................  10     0   S10
........................................................................accggaugaacuauuuug.......................  1      0   S10
........................................................................accggaugaacuauuuugg......................  1      0   S10
........................................................................accggaugaacuauuuuggu.....................  4      0   S10
........................................................................accggaugaacuauuuugguu....................  56     0   S10
........................................................................accggaugaacuauuuugguuc...................  18     0   S10
........................................................................accggaugaacuauuuugguucu..................  4      0   S10
.........................................................................ccggaugaacuauuuugguu....................  20     0   S10
.........................................................................ccggaugaacuauuuugguuc...................  5      0   S10
.........................................................................ccggaugaacuauuuugguucu..................  17     0   S10
..........................................................................cggaugaacuauuuuggu.....................  4      0   S10
..........................................................................cggaugaacuauuuugguu....................  18     0   S10
...........................................................................ggaugaacuauuuugguu....................  21     0   S10
```

Provisional ID	:	Scaffold1083_21842
Score total	:	2307.1
Score for star read(s)	:	3.9
Score for read counts	:	2305
Score for mfe	:	0.3
Score for randfold	:	-2.2
Score for cons. seed	:	
Total read count	:	4533
Mature read count	:	4415
Loop read count	:	0
Star read count	:	118

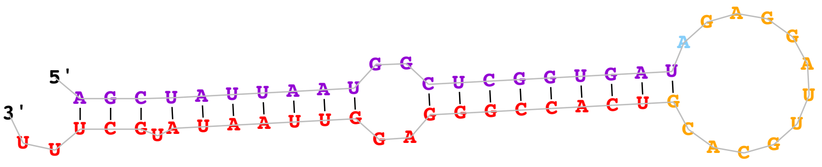

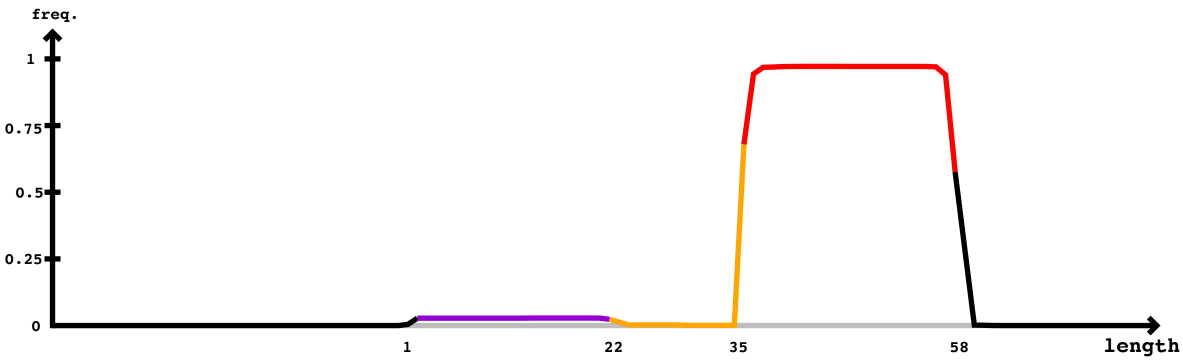


```
                                   Star                              Mature
5'- aacagccuuuuauguucaucuaaucuugucaagaccagcuauuaaauggcucggugauagaggauugcacgucaccgggagguuaauaugcuuuuaaacuugagaaccaggguu  -3'   obs
    aacagccuuuuauguucaucuaaucuugucaagaccagcuauuaaauggcucggugauagaggauugcacgucaccgggagguuaauaugcuuuuaaacuugagaaccaggguu        exp
    ...((((((....((((...........(((((...((((((((((..(((((((((..............)))))))))..))))))).))).......)))))))))).))))))   reads   mm   sample
    ...................................cagcuauuaaauggcucggugau.........................................................   4       0    S02
    ....................................agcuauuaaauggcucgguga..........................................................   5       0    S02
    ....................................agcuauuaaauggcucggugau.........................................................   8       0    S02
    ....................................agcuauuaaauggcucggugauagaggau..................................................   2       0    S02
    .................................................cucggugauagaggauugcacgu...........................................   2       0    S02
    ........................................................................ucaccgggagguuaauaugcu......................   5       0    S02
    ........................................................................ucaccgggagguuaauaugcuu.....................   52      0    S02
    ........................................................................ucaccgggagguuaauaugcuuu....................   166     0    S02
    ........................................................................ucaccgggagguuaauaugcuuuu...................   20      0    S02
    .........................................................................caccgggagguuaauaugcu......................   2       0    S02
    .........................................................................caccgggagguuaauaugcuu.....................   106     0    S02
    .........................................................................caccgggagguuaauaugcuuu....................   84      0    S02
    .........................................................................caccgggagguuaauaugcuuuu...................   34      0    S02
    ..........................................................................accgggagguuaauaugcuuu....................   2       0    S02
    ..........................................................................accgggagguuaauaugcuuuu...................   10      0    S02
    ...........................................................................ccgggagguuaauaugcuuuu...................   2       0    S02
    ............................................................................cgggagguuaauaugcuuu....................   1       0    S02

    ...................................cagcuauuaaauggcucggugau.........................................................   2       0    S04
    ....................................agcuauuaaauggcucgguga..........................................................   4       0    S04
    ....................................agcuauuaaauggcucggugau.........................................................   13      0    S04
    ....................................agcuauuaaauggcucggugaua........................................................   1       0    S04
    ....................................agcuauuaaauggcucggugauagaggau..................................................   1       0    S04
    ........................................................................ucaccgggagguuaauaugcu......................   16      0    S04
    ........................................................................ucaccgggagguuaauaugcuu.....................   166     0    S04
    ........................................................................ucaccgggagguuaauaugcuuu....................   312     0    S04
    ........................................................................ucaccgggagguuaauaugcuuuu...................   26      0    S04
    .........................................................................caccgggagguuaauaugcu......................   12      0    S04
    .........................................................................caccgggagguuaauaugcuu.....................   107     0    S04
    .........................................................................caccgggagguuaauaugcuuu....................   39      0    S04
    .........................................................................caccgggagguuaauaugcuuuu...................   14      0    S04
    ..........................................................................accgggagguuaauaugcuuu....................   4       0    S04
    ..........................................................................accgggagguuaauaugcuuuu...................   9       0    S04
```

```
                                    Star                              Mature
aacagccuuuuauguucaucuaaucuugucaagaccagcuauuaaauggcucggugauagaggauugcacgucaccgggagguuaauaugcuuuuaaacuugagaaccaggguu
..........................................................................ccgggagguuaauaugcuu.....................    1    0    S04
...........................................................................cgggagguuaauaugcuu.....................    2    0    S04
............................................................................gggagguuaauaugcuuu....................    1    0    S04

....................................agcuauuaaauggcucggug..........................................................    1    0    S09
....................................agcuauuaaauggcucggugau........................................................    4    0    S09
.......................................................................ucaccgggagguuaauaugcu......................    4    0    S09
.......................................................................ucaccgggagguuaauaugcuu.....................    27   0    S09
.......................................................................ucaccgggagguuaauaugcuuu....................    103  0    S09
.......................................................................ucaccgggagguuaauaugcuuuu...................    9    0    S09
........................................................................caccgggagguuaauaugcu......................    1    0    S09
........................................................................caccgggagguuaauaugcuu.....................    8    0    S09
.........................................................................accgggagguuaauaugcuuuu...................    5    0    S09

...................................cagcuauuaaauggcucggugau........................................................    2    0    S03
....................................agcuauuaaauggcucgguga.........................................................    2    0    S03
....................................agcuauuaaauggcucggugau........................................................    7    0    S03
............................................................aggauugcacgucaccgggagg................................    1    0    S03
.......................................................................ucaccgggagguuaauaugc.......................    2    0    S03
.......................................................................ucaccgggagguuaauaugcu......................    19   0    S03
.......................................................................ucaccgggagguuaauaugcuu.....................    203  0    S03
.......................................................................ucaccgggagguuaauaugcuuu....................    395  0    S03
.......................................................................ucaccgggagguuaauaugcuuuu...................    30   0    S03
.......................................................................ucaccgggagguuaauaugcuuuua..................    1    0    S03
........................................................................caccgggagguuaauaugc.......................    1    0    S03
........................................................................caccgggagguuaauaugcu......................    9    0    S03
........................................................................caccgggagguuaauaugcuu.....................    115  0    S03
........................................................................caccgggagguuaauaugcuuu....................    40   0    S03
........................................................................caccgggagguuaauaugcuuuu...................    12   0    S03
.........................................................................accgggagguuaauaugcuuu....................    4    0    S03
.........................................................................accgggagguuaauaugcuuuu...................    16   0    S03
.........................................................................accgggagguuaauaugcuuuuaa.................    1    0    S03
.........................................................................accgggagguuaauaugcuuuuaaa................    1    0    S03
..........................................................................ccgggagguuaauaugcuu.....................    1    0    S03

....................................agcuauuaaauggcucgguga.........................................................    6    0    S08
....................................agcuauuaaauggcucggugau........................................................    6    0    S08
.......................................................................ucaccgggagguuaauaugcu......................    5    0    S08
.......................................................................ucaccgggagguuaauaugcuu.....................    140  0    S08
.......................................................................ucaccgggagguuaauaugcuuu....................    239  0    S08
.......................................................................ucaccgggagguuaauaugcuuuu...................    10   0    S08
........................................................................caccgggagguuaauaugcu......................    7    0    S08
........................................................................caccgggagguuaauaugcuu.....................    56   0    S08
........................................................................caccgggagguuaauaugcuuu....................    8    0    S08
........................................................................caccgggagguuaauaugcuuuu...................    2    0    S08
.........................................................................accgggagguuaauaugcuuu....................    1    0    S08
.........................................................................accgggagguuaauaugcuuuu...................    11   0    S08
..........................................................................ccgggagguuaauaugcuu.....................    1    0    S08
...........................................................................cgggagguuaauaugcuu.....................    1    0    S08

....................................agcuauuaaauggcucgguga.........................................................    1    0    S06
....................................agcuauuaaauggcucggugau........................................................    5    0    S06
.......................................................................ucaccgggagguuaauaugcu......................    5    0    S06
.......................................................................ucaccgggagguuaauaugcuu.....................    39   0    S06
.......................................................................ucaccgggagguuaauaugcuuu....................    47   0    S06
.......................................................................ucaccgggagguuaauaugcuuuu...................    8    0    S06
........................................................................caccgggagguuaauaugc.......................    1    0    S06
........................................................................caccgggagguuaauaugcu......................    6    0    S06
........................................................................caccgggagguuaauaugcuu.....................    58   0    S06
........................................................................caccgggagguuaauaugcuuu....................    15   0    S06
........................................................................caccgggagguuaauaugcuuuu...................    6    0    S06
.........................................................................accgggagguuaauaugc.......................    1    0    S06
.........................................................................accgggagguuaauaugcuu.....................    1    0    S06
.........................................................................accgggagguuaauaugcuuu....................    2    0    S06
.........................................................................accgggagguuaauaugcuuuu...................    4    0    S06
............................................................................gggagguuaauaugcuuuu...................    1    0    S06

...................................cagcuauuaaauggcucggugau........................................................    10   0    S01
....................................agcuauuaaauggcucggugau........................................................    12   0    S01
....................................agcuauuaaauggcucggugauagaggau.................................................    4    0    S01
```

Star **Mature**

```
aacagccuuuuauguucaucuaaucuugucaagaccagcuauuaauggcucggugauagaggauugcacgucaccgggagguuaauaugcuuuuaaacuugagaaccaggguu
....................................agcuauuaauggcucggugauagaggauu................................................	1	0	S01
......................................................................ucaccgggagguuaauaugcu......................	11	0	S01
......................................................................ucaccgggagguuaauaugcuu.....................	49	0	S01
......................................................................ucaccgggagguuaauaugcuuu....................	129	0	S01
......................................................................ucaccgggagguuaauaugcuuuu...................	15	0	S01
......................................................................ucaccgggagguuaauaugcuuuua..................	1	0	S01
......................................................................ucaccgggagguuaauaugcuuuuaa.................	1	0	S01
.......................................................................caccgggagguuaauaugcu......................	5	0	S01
.......................................................................caccgggagguuaauaugcuu.....................	95	0	S01
.......................................................................caccgggagguuaauaugcuuu....................	85	0	S01
.......................................................................caccgggagguuaauaugcuuuu...................	49	0	S01
........................................................................accgggagguuaauaugcuuu....................	2	0	S01
........................................................................accgggagguuaauaugcuuuu...................	10	0	S01
........................................................................accgggagguuaauaugcuuuuaa.................	1	0	S01
..........................................................................cgggagguuaauaugcuuuu...................	1	0	S01

....................................agcuauuaauggcucggugau........................................................	5	0	S07
......................................................................ucaccgggagguuaauaugc.......................	1	0	S07
......................................................................ucaccgggagguuaauaugcu......................	8	0	S07
......................................................................ucaccgggagguuaauaugcuu.....................	80	0	S07
......................................................................ucaccgggagguuaauaugcuuu....................	146	0	S07
......................................................................ucaccgggagguuaauaugcuuuu...................	15	0	S07
.......................................................................caccgggagguuaauaugcu......................	3	0	S07
.......................................................................caccgggagguuaauaugcuu.....................	41	0	S07
.......................................................................caccgggagguuaauaugcuuu....................	14	0	S07
.......................................................................caccgggagguuaauaugcuuuu...................	5	0	S07
........................................................................accgggagguuaauaugcuuu....................	2	0	S07
........................................................................accgggagguuaauaugcuuuu...................	7	0	S07
...........................................................................gggagguuaauaugcuuu....................	1	0	S07

...................................cagcuauuaauggcucggugau........................................................	1	0	S05
....................................agcuauuaauggcucggugua.........................................................	3	0	S05
....................................agcuauuaauggcucggugau........................................................	10	0	S05
......................................................................ucaccgggagguuaauaug........................	1	0	S05
......................................................................ucaccgggagguuaauaugcu......................	7	0	S05
......................................................................ucaccgggagguuaauaugcuu.....................	179	0	S05
......................................................................ucaccgggagguuaauaugcuuu....................	274	0	S05
......................................................................ucaccgggagguuaauaugcuuuu...................	38	0	S05
......................................................................ucaccgggagguuaauaugcuuuua..................	1	0	S05
.......................................................................caccgggagguuaauaugcu......................	7	0	S05
.......................................................................caccgggagguuaauaugcuu.....................	83	0	S05
.......................................................................caccgggagguuaauaugcuuu....................	28	0	S05
.......................................................................caccgggagguuaauaugcuuuu...................	8	0	S05
........................................................................accgggagguuaauaugcu......................	1	0	S05
........................................................................accgggagguuaauaugcuu.....................	2	0	S05
........................................................................accgggagguuaauaugcuuu....................	4	0	S05
........................................................................accgggagguuaauaugcuuuu...................	12	0	S05
..........................................................................cgggagguuaauaugcuuu....................	1	0	S05
..........................................................................cgggagguuaauaugcuuuu...................	1	0	S05
............................................................................ggagguuaauaugcuuuu...................	1	0	S05

....................................agcuauuaauggcucggugau........................................................	6	0	S10
......................................................................ucaccgggagguuaauaugcu......................	2	0	S10
......................................................................ucaccgggagguuaauaugcuu.....................	20	0	S10
......................................................................ucaccgggagguuaauaugcuuu....................	47	0	S10
......................................................................ucaccgggagguuaauaugcuuuu...................	11	0	S10
.......................................................................caccgggagguuaauaugcu......................	1	0	S10
.......................................................................caccgggagguuaauaugcuu.....................	21	0	S10
.......................................................................caccgggagguuaauaugcuuu....................	9	0	S10
.......................................................................caccgggagguuaauaugcuuuu...................	3	0	S10
........................................................................accgggagguuaauaugcuuuuaa.................	1	0	S10
```

Provisional ID	:	Scaffold5285_44885
Score total	:	104153.9
Score for star read(s)	:	3.9
Score for read counts	:	104147.2
Score for mfe	:	1.2
Score for randfold	:	1.6
Score for cons. seed	:	
Total read count	:	204292
Mature read count	:	196460
Loop read count	:	3
Star read count	:	7829

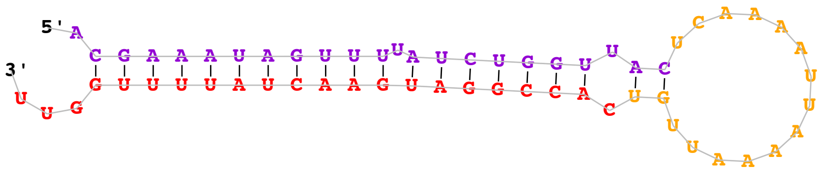

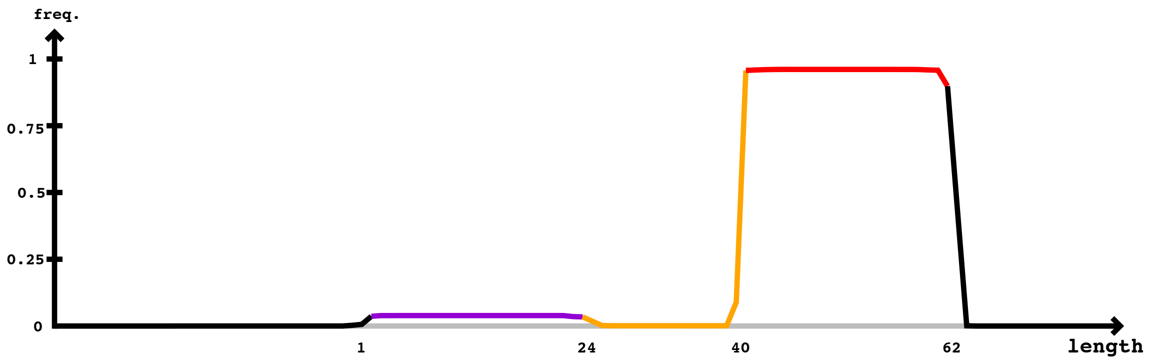


```
                                 Star                                   Mature
5'- aaauuacaaagcaaugaauaucuaaauggaaacgaaauaguuuuaucugguuacucaaaauuaaaaauugucaccggaugaacuauuuugguucuaugggauauuuauca  -3'   obs
    aaauuacaaagcaaugaauaucuaaauggaaacgaaauaguuuuaucugguuacucaaaauuaaaaauugucaccggaugaacuauuuugguucuaugggauauuuauca         exp
    .............((((((((((..((((((.(((((((((((.(((((((.((..............)).)))))))))))))))))).)))))).))))))))))..   reads   mm   sample
    ...............................acgaaauaguuuuaucugguuac........................................................   1       0    S02
    ..................................aaauaguuAuaucugguu..........................................................   2       1    S02
    ..................................aaauaguuAuaucugguua.........................................................   2       1    S02
    ......................................................................ucaccggaugaacuauuuuggu..................   2       0    S02
    .......................................................................caccggaugaacuauuuuggu..................   1       0    S02
    .......................................................................caccggaugaacuauuuugguu.................   14      0    S02
    .......................................................................caccggaugaacuauuuugguuc................   1       0    S02

    ........aagcaaugaauaucuaaauggaa...............................................................................   1       0    S04
    ..........gcaaugaauaucuaaaugga................................................................................   1       0    S04
    .............................aaacgaaauaguuuuaucuggu...........................................................   1       0    S04
    .............................aaacgaaauaguuuuaucugguu..........................................................   39      0    S04
    ..............................aacgaaauaguuuuaucugguu..........................................................   7       0    S04
    ..............................aacgaaauaguuuuaucugguua.........................................................   4       0    S04
    ..............................aacgaaauaguuuuaucugguuac........................................................   19      0    S04
    ...............................acgaaauaguuuuaucugguu..........................................................   1       0    S04
    ...............................acgaaauaguuuuaucugguua.........................................................   3       0    S04
    ...............................acgaaauaguuuuaucugguuac........................................................   186     0    S04
    ...............................acgaaauaguuuuaucugguuacu.......................................................   9       0    S04
    ...............................acgaaauaguuuuaucugguuacuc......................................................   4       0    S04
    ...............................acgaaauaguuuuaucugguuacuca.....................................................   2       0    S04
    ...............................acgaaauaguuuuaucugguuacucaaaauuaaaaauu.........................................   1       0    S04
    ...............................acgaaauaguuuuaucugguuacucaaaauuaaaaauugu.......................................   3       0    S04
    ................................cgaaauaguuuuaucugguuacu.......................................................   13      0    S04
    ................................cgaaauaguuuuaucugguuacuc......................................................   4       0    S04
    ................................cgaaauaguuuuaucugguuacuca.....................................................   1       0    S04
    ............................................................uuaaaaauugucaccggaugaac...........................   1       0    S04
    .................................................................aauugucaccggaugaacu..........................   1       0    S04
    ......................................................................ucaccggaugaacuauuuugg...................   3       0    S04
    ......................................................................ucaccggaugaacuauuuuggu..................   109     0    S04
    ......................................................................ucaccggaugaacuauuuugguu.................   237     0    S04
    ......................................................................ucaccggaugaacuauuuugguuc................   10      0    S04
    .......................................................................caccggaugaacuauuuu.....................   1       0    S04
```

```
                              Star                                    Mature
aaauuacaaagcaaugaauaucuaaauggaaacgaaauaguuuuaucugguuacucaaaauuaaaaauugucaccggaugaacuauuuugguucuaugggauauuuauca
.......................................................................caccggaugaacuauuuug....................   6      0   S04
.......................................................................caccggaugaacuauuuugg...................   4      0   S04
.......................................................................caccggaugaacuauuuuggu..................   92     0   S04
.......................................................................caccggaugaacuauuuugguu.................   3737   0   S04
.......................................................................caccggaugaacuauuuugguuc................   53     0   S04
.......................................................................caccggaugaacuauuuugguucu...............   1      0   S04
........................................................................accggaugaacuauuuugguu.................   8      0   S04
........................................................................accggaugaacuauuuugguuc................   4      0   S04
.........................................................................ccggaugaacuauuuugguu.................   4      0   S04
.........................................................................ccggaugaacuauuuugguuc................   1      0   S04
.........................................................................ccggaugaacuauuuugguucu...............   9      0   S04
..........................................................................cggaugaacuauuuuggu..................   1      0   S04
..........................................................................cggaugaacuauuuugguu.................   2      0   S04
...........................................................................ggaugaacuauuuugguu.................   3      0   S04

.aauuacaaagcaaugaauaucuaaauggaa...............................................................................   1      0   S09
........aagcaaugaauaucuaaauggaa...............................................................................   1      0   S09
.............................aaacgaaauaguuuuaucuggu...........................................................   1      0   S09
.............................aaacgaaauaguuuuaucugguu..........................................................   99     0   S09
.............................aaacgaaauaguuuuaucugguuac........................................................   1      0   S09
.............................aaacgaaauaguuuuaucugguuacu.......................................................   1      0   S09
..............................aacgaaauaguuuuaucuggu...........................................................   1      0   S09
..............................aacgaaauaguuuuaucugguu..........................................................   60     0   S09
..............................aacgaaauaguuuuaucugguua.........................................................   39     0   S09
..............................aacgaaauaguuuuaucugguuac........................................................   88     0   S09
..............................aacgaaauaguuuuaucugguuacu.......................................................   4      0   S09
..............................aacgaaauaguuuuaucugguuacuc......................................................   1      0   S09
...............................acgaaauaguuuuaucug.............................................................   1      0   S09
...............................acgaaauaguuuuaucugguu..........................................................   2      0   S09
...............................acgaaauaguuuuaucugguua.........................................................   36     0   S09
...............................acgaaauaguuuuaucugguuac........................................................   1366   0   S09
...............................acgaaauaguuuuaucugguuacu.......................................................   39     0   S09
...............................acgaaauaguuuuaucugguuacuc......................................................   7      0   S09
...............................acgaaauaguuuuaucugguuacuca.....................................................   2      0   S09
...............................acgaaauaguuuuaucugguuacucaaaauuaaaaau..........................................   2      0   S09
...............................acgaaauaguuuuaucugguuacucaaaauuaaaaauugu.......................................   18     0   S09
................................cgaaauaguuuuaucugguuac........................................................   12     0   S09
................................cgaaauaguuuuaucugguuacu.......................................................   78     0   S09
................................cgaaauaguuuuaucugguuacuc......................................................   28     0   S09
................................cgaaauaguuuuaucugguuacuca.....................................................   3      0   S09
.................................gaaauaguuuuaucugguuac........................................................   1      0   S09
..................................aaauaguuAuaucugguu..........................................................   3      1   S09
....................................auaguuuuaucugguuac........................................................   1      0   S09
.....................................................cucaaaauuaaaaauugucaccggaugaacuauuuugguu.................   1      0   S09
......................................................ucaaaauuaaaaauugucaccggaugaac...........................   1      0   S09
........................................................aaaauuaaaaauugucaccggau...............................   1      0   S09
............................................................uuaaaaauugucaccggaugaacu..........................   1      0   S09
............................................................uuaaaaauugucaccggaugaacuauuuugguu.................   2      0   S09
.....................................................................gucaccggaugaacuauuuugguu.................   1      0   S09
......................................................................ucaccggaugaacuauuu......................   8      0   S09
......................................................................ucaccggaugaacuauuuug....................   7      0   S09
......................................................................ucaccggaugaacuauuuugg...................   30     0   S09
......................................................................ucaccggaugaacuauuuuggu..................   2302   0   S09
......................................................................ucaccggaugaacuauuuugguu.................   3653   0   S09
......................................................................ucaccggaugaacuauuuugguuc................   63     0   S09
......................................................................ucaccggaugaacuauuuugguucu...............   1      0   S09
.......................................................................caccggaugaacuauuuu.....................   9      0   S09
.......................................................................caccggaugaacuauuuug....................   74     0   S09
.......................................................................caccggaugaacuauuuugg...................   30     0   S09
.......................................................................caccggaugaacuauuuuggu..................   2303   0   S09
.......................................................................caccggaugaacuauuuugguu.................   51824  0   S09
.......................................................................caccggaugaacuauuuugguuc................   536    0   S09
.......................................................................caccggaugaacuauuuugguucu...............   7      0   S09
........................................................................accggaugaacuauuuug....................   4      0   S09
........................................................................accggaugaacuauuuugg...................   1      0   S09
........................................................................accggaugaacuauuuuggu..................   1      0   S09
........................................................................accggaugaacuauuuugguu.................   58     0   S09
........................................................................accggaugaacuauuuugguuc................   19     0   S09
........................................................................accggaugaacuauuuugguucu...............   4      0   S09
.........................................................................ccggaugaacuauuuuggu..................   1      0   S09
```

Star **Mature**

```
aaauuacaaagcaaugaauaucuaaauggaaacgaaauaguuuuaucugguuacucaaaauuaaaauugucaccggaugaacuauuuugguucuaugggauauuuauca
........................................................................ccggaugaacuauuuugguu.................   20     0    S09
........................................................................ccggaugaacuauuuugguuc................   8      0    S09
........................................................................ccggaugaacuauuuugguucu...............   12     0    S09
.........................................................................cggaugaacuauuuuggu..................   3      0    S09
.........................................................................cggaugaacuauuuugguu.................   24     0    S09
..........................................................................ggaugaacuauuuugguu.................   14     0    S09

aaauuacaaagcaaugaauaucuaaauggaa..............................................................................   1      0    S03
..........gcaaugaauaucuaaaugga...............................................................................   1      0    S03
...........caaugaauaucuaaauggaa..............................................................................   2      0    S03
.............................aaacgaaauaguuuuaucugguu.........................................................   21     0    S03
.............................aaacgaaauaguuuuaucugguuac.......................................................   1      0    S03
..............................aacgaaauaguuuuaucugguu.........................................................   2      0    S03
..............................aacgaaauaguuuuaucugguua........................................................   2      0    S03
..............................aacgaaauaguuuuaucugguuac.......................................................   15     0    S03
...............................acgaaauaguuuuaucugguua........................................................   4      0    S03
...............................acgaaauaguuuuaucugguuac.......................................................   125    0    S03
...............................acgaaauaguuuuaucugguuacu......................................................   4      0    S03
...............................acgaaauaguuuuaucugguuacuc.....................................................   2      0    S03
...............................acgaaauaguuuuaucugguuacucaaaauuaaaauu.........................................   1      0    S03
...............................acgaaauaguuuuaucugguuacucaaaauuaaaauugu.......................................   4      0    S03
................................cgaaauaguuuuaucugguuacu......................................................   6      0    S03
................................cgaaauaguuuuaucugguuacuc.....................................................   1      0    S03
................................cgaaauaguuuuaucugguuacuca....................................................   1      0    S03
................................cgaaauaguuuuaucugguuacucaaaauuaaaau..........................................   1      0    S03
.................................gaaauaguuuuaucugguuacu......................................................   1      0    S03
...................................aauaguuuuaucugguuac.......................................................   1      0    S03
.............................................................uaaaauugucaccggaugaacuauuuugguu.................   1      0    S03
..................................................................uugucaccggaugaacuauuuugguu.................   1      0    S03
.....................................................................ucaccggaugaacuauuu......................   1      0    S03
.....................................................................ucaccggaugaacuauuuug....................   3      0    S03
.....................................................................ucaccggaugaacuauuuugg...................   1      0    S03
.....................................................................ucaccggaugaacuauuuuggu..................   52     0    S03
.....................................................................ucaccggaugaacuauuuugguu.................   136    0    S03
.....................................................................ucaccggaugaacuauuuugguuc................   3      0    S03
......................................................................caccggaugaacuauuuu.....................   3      0    S03
......................................................................caccggaugaacuauuuug....................   6      0    S03
......................................................................caccggaugaacuauuuugg...................   1      0    S03
......................................................................caccggaugaacuauuuuggu..................   55     0    S03
......................................................................caccggaugaacuauuuugguu.................   2499   0    S03
......................................................................caccggaugaacuauuuugguuc................   32     0    S03
.......................................................................accggaugaacuauuuug....................   1      0    S03
.......................................................................accggaugaacuauuuugg...................   1      0    S03
.......................................................................accggaugaacuauuuugguuc................   4      0    S03
.......................................................................accggaugaacuauuuugguucu...............   1      0    S03
........................................................................ccggaugaacuauuuugguu.................   5      0    S03
........................................................................ccggaugaacuauuuugguuc................   6      0    S03
........................................................................ccggaugaacuauuuugguucu...............   12     0    S03
..........................................................................ggaugaacuauuuugguu.................   2      0    S03

......caaagcaaugaauaucuaaaugga...............................................................................   1      0    S08
...........caaugaauaucuaaauggaa..............................................................................   2      0    S08
............aaugaauaucuaaauggaaa.............................................................................   1      0    S08
.............................aaacgaaauaguuuuaucuggu..........................................................   1      0    S08
.............................aaacgaaauaguuuuaucugguu.........................................................   60     0    S08
.............................aaacgaaauaguuuuaucugguuac.......................................................   1      0    S08
..............................aacgaaauaguuuuaucugguu.........................................................   49     0    S08
..............................aacgaaauaguuuuaucugguua........................................................   18     0    S08
..............................aacgaaauaguuuuaucugguuac.......................................................   42     0    S08
...............................acgaaauaguuuuaucug............................................................   1      0    S08
...............................acgaaauaguuuuaucugguu.........................................................   4      0    S08
...............................acgaaauaguuuuaucugguua........................................................   9      0    S08
...............................acgaaauaguuuuaucugguuac.......................................................   732    0    S08
...............................acgaaauaguuuuaucugguuacu......................................................   15     0    S08
...............................acgaaauaguuuuaucugguuacuc.....................................................   7      0    S08
...............................acgaaauaguuuuaucugguuacucaaaauu...............................................   3      0    S08
...............................acgaaauaguuuuaucugguuacucaaaauua..............................................   1      0    S08
...............................acgaaauaguuuuaucugguuacucaaaauuaaaau..........................................   1      0    S08
...............................acgaaauaguuuuaucugguuacucaaaauuaaaauu.........................................   2      0    S08
...............................acgaaauaguuuuaucugguuacucaaaauuaaaauug........................................   1      0    S08
```

Star Mature

```
aaauuacaaagcaaugaauaucuaaauggaaacgaaauaguuuuaucugguuacucaaaauuaaaauugucaccggaugaacuauuuugguucuaugggauauuuauca
...............................acgaaauaguuuuaucugguuacucaaaauuaaaauugu.......................................   19      0   S08
................................cgaaauaguuuuaucugguuac.......................................................   5       0   S08
................................cgaaauaguuuuaucugguuacu......................................................   49      0   S08
................................cgaaauaguuuuaucugguuacuc.....................................................   13      0   S08
................................cgaaauaguuuuaucugguuacuca....................................................   6       0   S08
................................cgaaauaguuuuaucugguuacucaaaauuaaaauu.........................................   4       0   S08
................................cgaaauaguuuuaucugguuacucaaaauuaaaauugu.......................................   1       0   S08
.................................gaaauaguuuuaucugguuac.......................................................   1       0   S08
..................................aaauaguuuuaucugguuac.......................................................   1       0   S08
...................................aauaguuuuaucugguuac.......................................................   2       0   S08
.........................................uuuaucugguuacucaaaauuaaaauugu.......................................   1       0   S08
...................................................uacucaaaauuaaaauugucaccggaugaacuauuuugguu.................   3       0   S08
.....................................................cucaaaauuaaaauugucaccgg.................................   1       0   S08
......................................................ucaaaauuaaaauugucaccggaugaacu..........................   1       0   S08
.........................................................aaauuaaaauugucaccggaugaacuauuuugguu.................   1       0   S08
................................................................aauugucaccggaugaacuauuuugguu.................   2       0   S08
...................................................................ugucaccggaugaacuauuuugguu.................   2       0   S08
.....................................................................ucaccggaugaacuauuu......................   1       0   S08
.....................................................................ucaccggaugaacuauuuug....................   3       0   S08
.....................................................................ucaccggaugaacuauuuugg...................   8       0   S08
.....................................................................ucaccggaugaacuauuuuggu..................   443     0   S08
.....................................................................ucaccggaugaacuauuuugguu.................   1206    0   S08
.....................................................................ucaccggaugaacuauuuugguuc................   19      0   S08
......................................................................caccggaugaacuauuuu.....................   9       0   S08
......................................................................caccggaugaacuauuuug....................   15      0   S08
......................................................................caccggaugaacuauuuugg...................   10      0   S08
......................................................................caccggaugaacuauuuuggu..................   453     0   S08
......................................................................caccggaugaacuauuuugguu.................   14088   0   S08
......................................................................caccggaugaacuauuuugguuc................   198     0   S08
......................................................................caccggaugaacuauuuugguucu...............   9       0   S08
.......................................................................accggaugaacuauuuugguu.................   19      0   S08
.......................................................................accggaugaacuauuuugguuc................   8       0   S08
.......................................................................accggaugaacuauuuugguucu...............   3       0   S08
........................................................................ccggaugaacuauuuuggu..................   11      0   S08
........................................................................ccggaugaacuauuuugguu.................   30      0   S08
........................................................................ccggaugaacuauuuugguuc................   23      0   S08
........................................................................ccggaugaacuauuuugguucu...............   23      0   S08
.........................................................................cggaugaacuauuuugguu.................   45      0   S08
.........................................................................cggaugaacuauuuugguuc................   1       0   S08
..........................................................................ggaugaacuauuuugguu.................   10      0   S08

........aagcaaugaauaucuaaaugga...............................................................................   1       0   S06
.............................aaacgaaauaguuuuaucuggu..........................................................   3       0   S06
.............................aaacgaaauaguuuuaucugguu.........................................................   54      0   S06
.............................aaacgaaauaguuuuaucugguuac.......................................................   5       0   S06
..............................aacgaaauaguuuuaucugguu.........................................................   17      0   S06
..............................aacgaaauaguuuuaucugguua........................................................   10      0   S06
..............................aacgaaauaguuuuaucugguuac.......................................................   29      0   S06
...............................acgaaauaguuuuaucugguu.........................................................   2       0   S06
...............................acgaaauaguuuuaucugguua........................................................   4       0   S06
...............................acgaaauaguuuuaucugguuac.......................................................   583     0   S06
...............................acgaaauaguuuuaucugguuacu......................................................   13      0   S06
...............................acgaaauaguuuuaucugguuacuc.....................................................   9       0   S06
...............................acgaaauaguuuuaucugguuacuca....................................................   1       0   S06
...............................acgaaauaguuuuaucugguuacucaaaauuaaaauugu.......................................   12      0   S06
................................cgaaauaguuuuaucugguu.........................................................   1       0   S06
................................cgaaauaguuuuaucugguuac.......................................................   4       0   S06
................................cgaaauaguuuuaucugguuacu......................................................   30      0   S06
................................cgaaauaguuuuaucugguuacuc.....................................................   6       0   S06
................................cgaaauaguuuuaucugguuacuca....................................................   5       0   S06
.................................gaaauaguuuuaucugguuacuc.....................................................   1       0   S06
...................................................................ugucaccggaugaacuauuuugguuc................   1       0   S06
....................................................................gucaccggaugaacuauuuugguu.................   1       0   S06
.....................................................................ucaccggaugaacuauuu......................   1       0   S06
.....................................................................ucaccggaugaacuauuuu.....................   2       0   S06
.....................................................................ucaccggaugaacuauuuug....................   2       0   S06
.....................................................................ucaccggaugaacuauuuugg...................   5       0   S06
.....................................................................ucaccggaugaacuauuuuggu..................   138     0   S06
.....................................................................ucaccggaugaacuauuuugguu.................   472     0   S06
.....................................................................ucaccggaugaacuauuuugguuc................   4       0   S06
```

Star **Mature**

```
aaauuacaaagcaaugaauaucuaaauggaaacgaaauaguuuuaucugguuacucaaaauuaaaauugucaccggaugaacuauuuugguucuaugggauauuuauca
.....................................................................ucaccggaugaacuauuuugguucu...............	1	0	S06
......................................................................caccggaugaacuauuuu.....................	5	0	S06
......................................................................caccggaugaacuauuuug....................	8	0	S06
......................................................................caccggaugaacuauuuugg...................	4	0	S06
......................................................................caccggaugaacuauuuuggu..................	188	0	S06
......................................................................caccggaugaacuauuuugguu.................	6221	0	S06
......................................................................caccggaugaacuauuuugguuc................	91	0	S06
......................................................................caccggaugaacuauuuugguucu...............	2	0	S06
.......................................................................accggaugaacuauuuug....................	1	0	S06
.......................................................................accggaugaacuauuuugguu.................	6	0	S06
.......................................................................accggaugaacuauuuugguuc................	7	0	S06
.......................................................................accggaugaacuauuuugguucu...............	3	0	S06
........................................................................ccggaugaacuauuuugguu.................	2	0	S06
........................................................................ccggaugaacuauuuugguuc................	3	0	S06
........................................................................ccggaugaacuauuuugguucu...............	3	0	S06
.........................................................................cggaugaacuauuuugguu.................	4	0	S06
.........................................................................cggaugaacuauuuugguuc................	1	0	S06
.........................................................................cggaugaacuauuuugguucu...............	1	0	S06
..........................................................................ggaugaacuauuuugguu.................	1	0	S06

...............................acgaaauaguuuuaucugguuac.......................................................	1	0	S01
.....................................................................ucaccggaugaacuauuuugguu.................	1	0	S01
......................................................................caccggaugaacuauuuugguu.................	8	0	S01

.............................aaacgaaauaguuuuaucuggu..........................................................	1	0	S07
.............................aaacgaaauaguuuuaucugguu.........................................................	49	0	S07
.............................aaacgaaauaguuuuaucugguuacu......................................................	1	0	S07
..............................aacgaaauaguuuuaucugguu.........................................................	10	0	S07
..............................aacgaaauaguuuuaucugguua........................................................	9	0	S07
..............................aacgaaauaguuuuaucugguuac.......................................................	33	0	S07
..............................aacgaaauaguuuuaucugguuacu......................................................	1	0	S07
..............................aacgaaauaguuuuaucugguuacuc.....................................................	1	0	S07
...............................acgaaauaguuuuaucugguu.........................................................	1	0	S07
...............................acgaaauaguuuuaucugguua........................................................	12	0	S07
...............................acgaaauaguuuuaucugguuac.......................................................	693	0	S07
...............................acgaaauaguuuuaucugguuacu......................................................	23	0	S07
...............................acgaaauaguuuuaucugguuacuc.....................................................	14	0	S07
...............................acgaaauaguuuuaucugguuacuca....................................................	1	0	S07
...............................acgaaauaguuuuaucugguuacucaaaauua..............................................	2	0	S07
...............................acgaaauaguuuuaucugguuacucaaaauuaaaau..........................................	2	0	S07
...............................acgaaauaguuuuaucugguuacucaaaauuaaaauugu.......................................	35	0	S07
................................cgaaauaguuuuaucugguuac.......................................................	2	0	S07
................................cgaaauaguuuuaucugguuacu......................................................	28	0	S07
................................cgaaauaguuuuaucugguuacuc.....................................................	23	0	S07
................................cgaaauaguuuuaucugguuacuca....................................................	11	0	S07
................................cgaaauaguuuuaucugguuacucaaaauuaaaau..........................................	1	0	S07
................................cgaaauaguuuuaucugguuacucaaaauuaaaauu.........................................	1	0	S07
................................cgaaauaguuuuaucugguuacucaaaauuaaaauug........................................	3	0	S07
................................cgaaauaguuuuaucugguuacucaaaauuaaaauugu.......................................	1	0	S07
.................................gaaauaguuuuaucugguuac.......................................................	1	0	S07
.................................gaaauaguuuuaucugguuacu......................................................	1	0	S07
.................................gaaauaguuuuaucugguuacuc.....................................................	3	0	S07
..................................aaauaguuuuaucugguuacuc.....................................................	1	0	S07
.....................................uaguuuuaucugguuacu......................................................	1	0	S07
.....................................................cucaaaauuaaaauugucaccg..................................	2	0	S07
.......................................................caaaauuaaaauugucaccggau...............................	2	0	S07
...........................................................auuaaaauugucaccggaugaacuauuuugguu.................	1	0	S07
............................................................uuaaaauugucaccggaugaacuauuuugguu.................	2	0	S07
.............................................................uaaaauugucaccggaugaacuauuuugguu.................	1	0	S07
....................................................................gucaccggaugaacuauuuugguu.................	1	0	S07
.....................................................................ucaccggaugaacuauuuu.....................	2	0	S07
.....................................................................ucaccggaugaacuauuuug....................	7	0	S07
.....................................................................ucaccggaugaacuauuuugg...................	29	0	S07
.....................................................................ucaccggaugaacuauuuuggu..................	850	0	S07
.....................................................................ucaccggaugaacuauuuugguu.................	2036	0	S07
.....................................................................ucaccggaugaacuauuuugguuc................	28	0	S07
.....................................................................ucaccggaugaacuauuuugguucu...............	1	0	S07
......................................................................caccggaugaacuauuuu.....................	6	0	S07
......................................................................caccggaugaacuauuuug....................	60	0	S07
......................................................................caccggaugaacuauuuugg...................	20	0	S07
```

Star Mature

aaauuacaaagcaaugaauaucuaaauggaaacgaaauaguuuuaucugguuacucaaaauuaaaaauugucaccggaugaacuauuuugguucuaugggauauuuauca			
....caccggaugaacuauuuuggu..................	890	0	S07
....caccggaugaacuauuuugguu.................	33079	0	S07
....caccggaugaacuauuuugguuc................	316	0	S07
....caccggaugaacuauuuugguucu...............	5	0	S07
....accggaugaacuauuuug....................	1	0	S07
....accggaugaacuauuuugg...................	1	0	S07
....accggaugaacuauuuuggu..................	2	0	S07
....accggaugaacuauuuugguu.................	24	0	S07
....accggaugaacuauuuugguuc................	9	0	S07
....accggaugaacuauuuugguucu...............	6	0	S07
....ccggaugaacuauuuuggu..................	1	0	S07
....ccggaugaacuauuuugguu.................	10	0	S07
....ccggaugaacuauuuugguuc................	13	0	S07
....ccggaugaacuauuuugguucu...............	15	0	S07
....cggaugaacuauuuuggu..................	3	0	S07
....cggaugaacuauuuugguu.................	6	0	S07
....cggaugaacuauuuugguuc................	1	0	S07
....ggaugaacuauuuugguu.................	5	0	S07

.............................aaacgaaauaguuuuaucugguu..	51	0	S05
..............................aacgaaauaguuuuaucugguu..	6	0	S05
..............................aacgaaauaguuuuaucugguua...	5	0	S05
..............................aacgaaauaguuuuaucugguuac..	20	0	S05
..............................aacgaaauaguuuuaucugguuacu...	2	0	S05
...............................acgaaauaguuuuaucugguu..	1	0	S05
...............................acgaaauaguuuuaucugguua...	2	0	S05
...............................acgaaauaguuuuaucugguuac..	238	0	S05
...............................acgaaauaguuuuaucugguuacu...	3	0	S05
...............................acgaaauaguuuuaucugguuacuc..	2	0	S05
...............................acgaaauaguuuuaucugguuacucaaaauuaaaaauugu.......................................	2	0	S05
................................cgaaauaguuuuaucugguuacu...	11	0	S05
................................cgaaauaguuuuaucugguuacuc..	6	0	S05
................................cgaaauaguuuuaucugguuacuca...	2	0	S05
..................................aaauaguuAuaucugguua...	1	1	S05
....ucaccggaugaacuauuu......................	1	0	S05
....ucaccggaugaacuauuuug....................	3	0	S05
....ucaccggaugaacuauuuugg...................	2	0	S05
....ucaccggaugaacuauuuuggu..................	97	0	S05
....ucaccggaugaacuauuuugguu.................	318	0	S05
....ucaccggaugaacuauuuugguuc................	10	0	S05
....caccggaugaacuauuu......................	1	0	S05
....caccggaugaacuauuuug....................	3	0	S05
....caccggaugaacuauuuugg...................	4	0	S05
....caccggaugaacuauuuuggu..................	88	0	S05
....caccggaugaacuauuuugguu.................	4470	0	S05
....caccggaugaacuauuuugguuc................	79	0	S05
....caccggaugaacuauuuugguucu...............	1	0	S05
....accggaugaacuauuuuggu..................	1	0	S05
....accggaugaacuauuuugguu.................	7	0	S05
....accggaugaacuauuuugguuc................	9	0	S05
....accggaugaacuauuuugguucu...............	2	0	S05
....ccggaugaacuauuuugguu.................	3	0	S05
....ccggaugaacuauuuugguuc................	6	0	S05
....ccggaugaacuauuuugguucu...............	6	0	S05
....cggaugaacuauuuugguu.................	4	0	S05
....ggaugaacuauuuugguu.................	2	0	S05

.............................aaacgaaauaguuuuaucuggu...	2	0	S10
.............................aaacgaaauaguuuuaucugguu..	124	0	S10
.............................aaacgaaauaguuuuaucugguua...	1	0	S10
.............................aaacgaaauaguuuuaucugguuac..	3	0	S10
..............................aacgaaauaguuuuaucuggu...	1	0	S10
..............................aacgaaauaguuuuaucugguu..	90	0	S10
..............................aacgaaauaguuuuaucugguua...	47	0	S10
..............................aacgaaauaguuuuaucugguuac..	100	0	S10
..............................aacgaaauaguuuuaucugguuacu...	3	0	S10
...............................acgaaauaguuuuaucug...	2	0	S10
...............................acgaaauaguuuuaucuggu...	2	0	S10
...............................acgaaauaguuuuaucugguu..	5	0	S10
...............................acgaaauaguuuuaucugguua...	34	0	S10

```
                                Star                                    Mature
aaauuacaaagcaaugaauaucuaaauggaaacgaaauaguuuuaucugguuacucaaaauuaaaauugucaccggaugaacuauuuugguucuaugggauauuuauca
...............................acgaaauaguuuuaucugguuac.......................................................	1758	0	S10
...............................acgaaauaguuuuaucugguuacu......................................................	61	0	S10
...............................acgaaauaguuuuaucugguuacuc.....................................................	14	0	S10
...............................acgaaauaguuuuaucugguuacuca....................................................	4	0	S10
...............................acgaaauaguuuuaucugguuacucaaaauuaaaau..........................................	1	0	S10
...............................acgaaauaguuuuaucugguuacucaaaauuaaaauu.........................................	4	0	S10
...............................acgaaauaguuuuaucugguuacucaaaauuaaaauugu.......................................	31	0	S10
...............................acgaaauaguuuuaucugguuacucaaaauuaaaauugucaccg..................................	1	0	S10
................................cgaaauaguuuuaucugg...........................................................	1	0	S10
................................cgaaauaguuuuaucugguua........................................................	1	0	S10
................................cgaaauaguuuuaucugguuac.......................................................	13	0	S10
................................cgaaauaguuuuaucugguuacu......................................................	98	0	S10
................................cgaaauaguuuuaucugguuacuc.....................................................	32	0	S10
................................cgaaauaguuuuaucugguuacuca....................................................	8	0	S10
................................cgaaauaguuuuaucugguuacucaaaauuaaaauu.........................................	6	0	S10
.................................gaaauaguuuuaucugguuacu......................................................	1	0	S10
..................................aaauaguuuuaucugguua........................................................	1	0	S10
....................................auaguuuuaucugguuac.......................................................	1	0	S10
.....................................................cucaaaauuaaaauugucaccg..................................	1	0	S10
.......................................................caaaauuaaaauugucaccggaug..............................	1	0	S10
.........................................................aaauuaaaauugucaccggaug..............................	1	0	S10
............................................................uuaaaauugucaccggaug..............................	1	0	S10
............................................................uuaaaauugucaccggaugaac...........................	3	0	S10
...............................................................aaauugucaccggaugaacuauu.......................	1	0	S10
................................................................aauugucaccggaugaacu..........................	1	0	S10
................................................................aauugucaccggaugaacuauuuugguu.................	2	0	S10
.................................................................auugucaccggaugaacu..........................	1	0	S10
...................................................................ugucaccggaugaacuauuuug....................	1	0	S10
...................................................................ugucaccggaugaacuauuuugguu.................	1	0	S10
.....................................................................ucaccggaugaacuauuu......................	2	0	S10
.....................................................................ucaccggaugaacuauuuug....................	10	0	S10
.....................................................................ucaccggaugaacuauuuugg...................	36	0	S10
.....................................................................ucaccggaugaacuauuuuggu..................	2167	0	S10
.....................................................................ucaccggaugaacuauuuugguu.................	3455	0	S10
.....................................................................ucaccggaugaacuauuuugguuc................	62	0	S10
.....................................................................ucaccggaugaacuauuuugguucu...............	2	0	S10
......................................................................caccggaugaacuauuu......................	11	0	S10
......................................................................caccggaugaacuauuuug....................	90	0	S10
......................................................................caccggaugaacuauuuugg...................	34	0	S10
......................................................................caccggaugaacuauuuuggu..................	1937	0	S10
......................................................................caccggaugaacuauuuugguu.................	53494	0	S10
......................................................................caccggaugaacuauuuugguuc................	472	0	S10
......................................................................caccggaugaacuauuuugguucu...............	10	0	S10
.......................................................................accggaugaacuauuuug....................	1	0	S10
.......................................................................accggaugaacuauuuugg...................	1	0	S10
.......................................................................accggaugaacuauuuuggu..................	4	0	S10
.......................................................................accggaugaacuauuuugguu.................	56	0	S10
.......................................................................accggaugaacuauuuugguuc................	18	0	S10
.......................................................................accggaugaacuauuuugguucu...............	4	0	S10
........................................................................ccggaugaacuauuuugguu.................	20	0	S10
........................................................................ccggaugaacuauuuugguuc................	5	0	S10
........................................................................ccggaugaacuauuuugguucu...............	17	0	S10
.........................................................................cggaugaacuauuuuggu..................	4	0	S10
.........................................................................cggaugaacuauuuugguu.................	18	0	S10
..........................................................................ggaugaacuauuuugguu.................	21	0	S10
```

Provisional ID	:	Scaffold2425_35146
Score total	:	270333.6
Score for star read(s)	:	3.9
Score for read counts	:	270326.2
Score for mfe	:	1.9
Score for randfold	:	1.6
Score for cons. seed	:	
Total read count	:	530245
Mature read count	:	505688
Loop read count	:	0
Star read count	:	24557

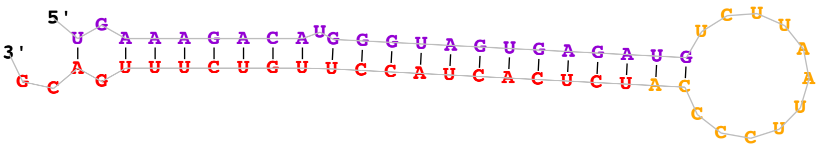

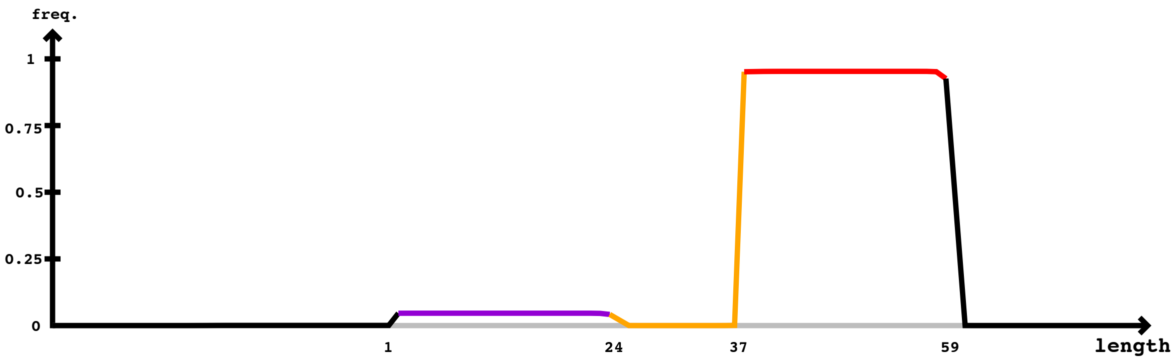


```
                                   Star                                 Mature
5'- aguaaauauuucuuuaaccuuagugauuuuacugugaaagacauggguagugagaugucuuaauuccccaucucacuaccuugucuuugacggaaagauuacuugaaucuuc   -3'   obs
    aguaaauauuucuuuaaccuuagugauuuuacugugaaagacauggguagugagaugucuuaauuccccaucucacuaccuugucuuugacggaaagauuacuugaaucuuc         exp
    .....................((((((((.((((.((((((.(((((((((((((((...........)))))))))))))))))))).)))).)))))))))......... reads  mm  sample
    ....aauauuucuuuaaccuuagugauuuuacug.............................................................................. 1      0   S06
    ...........cuuuaaccuuagugauuuuacug.............................................................................. 1      0   S06
    .............uuaaccuuagugauuuuacug.............................................................................. 1      0   S06
    ..............uaaccuuagugauuuuacug.............................................................................. 5      0   S06
    ...............aaccuuagugauuuuacug.............................................................................. 11     0   S06
    ................accuuagugauuuuacug.............................................................................. 1      0   S06
    ..................................ugaaagacauggguagugaga......................................................... 4      0   S06
    ..................................ugaaagacauggguagugagau........................................................ 60     0   S06
    ..................................ugaaagacauggguagugagaug....................................................... 344    0   S06
    ..................................ugaaagacauggguagugagaugu...................................................... 19     0   S06
    ...................................gaaagacauggguagugagaugu...................................................... 1      0   S06
    ................................................................uccccaucucacuaccuugucuuugacg.................... 1      0   S06
    ....................................................................caucucacuaccuugucuuuga...................... 1      0   S06
    ....................................................................caucucacuaccuugucuuugac..................... 3      0   S06
    .....................................................................aucucacuaccuugucuuugac..................... 25     0   S06
    .....................................................................aucucacuaccuugucuuugacg.................... 2      0   S06
    ......................................................................ucucacuaccuugucuuug....................... 4      0   S06
    ......................................................................ucucacuaccuugucuuuga...................... 19     0   S06
    ......................................................................ucucacuaccuugucuuugac..................... 1289   0   S06
    ......................................................................ucucacuaccuugucuuugacg.................... 61603  0   S06
    .......................................................................cucacuaccuugucuuugac..................... 3      0   S06
    .......................................................................cucacuaccuugucuuugacg.................... 40     0   S06
    ........................................................................ucacuaccuugucuuugacg.................... 36     0   S06
    ........................................................................ucacuaccuugucuuugacgg................... 2      0   S06
    .........................................................................cacuaccuugucuuugacg.................... 11     0   S06
    ..........................................................................acuaccuugucuuugacg.................... 4      0   S06

    ..................................ugaaagacauggguagugaga......................................................... 3      0   S01
    ..................................ugaaagacauggguagugagau........................................................ 8      0   S01
    ..................................ugaaagacauggguagugagaug....................................................... 27     0   S01
    ......................................................................ucucacuaccuugucuuuga...................... 3      0   S01
    ......................................................................ucucacuaccuugucuuugac..................... 56     0   S01
    ......................................................................ucucacuaccuugucuuugacg.................... 8319   0   S01
```

```
                                  Star                       Mature
aguaaauauuucuuuaaccuuagugauuuuacugugaaagacauggguagugagaugucuuaauuccccaucucacuaccuugucuuugacggaaagauuacuugaaucuuc
......................................................................ucucacuaccuugucuuugacgg...................    1      0    S01
.......................................................................cucacuaccuugucuuugacg....................    1      0    S01
........................................................................ucacuaccuugucuuugacg....................    9      0    S01
.........................................................................cacuaccuugucuuugacg....................    7      0    S01
..........................................................................acuaccuugucuuugacg....................    3      0    S01

.............uuaaccuuagugauuuuacug..............................................................................    1      0    S07
..............uaaccuuagugauuuuacug..............................................................................    1      0    S07
...............aaccuuagugauuuuacug..............................................................................    5      0    S07
................accuuagugauuuuacug..............................................................................    2      0    S07
................................ugugaaagacauggguagugaga.........................................................    2      0    S07
..................................ugaaagacauggguagugag..........................................................    3      0    S07
..................................ugaaagacauggguagugaga.........................................................    25     0    S07
..................................ugaaagacauggguagugagau........................................................    202    0    S07
..................................ugaaagacauggguagugagaug.......................................................    2347   0    S07
..................................ugaaagacauggguagugagaugu......................................................    94     0    S07
..................................ugaaagacauggguagugagauguc.....................................................    2      0    S07
...................................gaaagacauggguagugagaug.......................................................    2      0    S07
.........................................................ucuuaauuccccaucucacuaccuugucuuugacg....................    2      0    S07
................................................................uccccaucucacuaccuugucuuugacg....................    1      0    S07
....................................................................caucucacuaccuugucuuug.......................    1      0    S07
....................................................................caucucacuaccuugucuuuga......................    3      0    S07
....................................................................caucucacuaccuugucuuugac.....................    3      0    S07
.....................................................................aucucacuaccuugucuuuga......................    6      0    S07
.....................................................................aucucacuaccuugucuuugac.....................    35     0    S07
.....................................................................aucucacuaccuugucuuugacg....................    2      0    S07
......................................................................ucucacuaccuugucuuug.......................    1      0    S07
......................................................................ucucacuaccuugucuuuga......................    139    0    S07
......................................................................ucucacuaccuugucuuugac.....................    2135   0    S07
......................................................................ucucacuaccuugucuuugacg....................    87699  0    S07
......................................................................ucucacuaccuugucuuugacgg...................    8      0    S07
.......................................................................cucacuaccuugucuuugac.....................    2      0    S07
.......................................................................cucacuaccuugucuuugacg....................    38     0    S07
........................................................................ucacuaccuugucuuugac.....................    2      0    S07
........................................................................ucacuaccuugucuuugacg....................    50     0    S07
.........................................................................cacuaccuugucuuugacg....................    5      0    S07
..........................................................................acuaccuugucuuugacg....................    5      0    S07

.....auauuucuuuaaccuuagugauuuuacug..............................................................................    2      0    S05
...........cuuuaaccuuagugauuuuacug..............................................................................    1      0    S05
..............uaaccuuagugauuuuacug..............................................................................    1      0    S05
...............aaccuuagugauuuuacug..............................................................................    3      0    S05
..................................ugaaagacauggguagugaga.........................................................    5      0    S05
..................................ugaaagacauggguagugagau........................................................    32     0    S05
..................................ugaaagacauggguagugagaug.......................................................    221    0    S05
..................................ugaaagacauggguagugagaugu......................................................    10     0    S05
...................................gaaagacauggguagugagaug.......................................................    1      0    S05
.........................................................ucuuaauuccccaucucacuaccuugucuuugacg....................    1      0    S05
...................................................................ccaucucacuaccuugucuuugacg....................    1      0    S05
....................................................................caucucacuaccuugucuuugac.....................    1      0    S05
.....................................................................aucucacuaccuugucuuugac.....................    11     0    S05
.....................................................................aucucacuaccuugucuuugacg....................    3      0    S05
......................................................................ucucacuaccuugucuuug.......................    1      0    S05
......................................................................ucucacuaccuugucuuuga......................    9      0    S05
......................................................................ucucacuaccuugucuuugac.....................    707    0    S05
......................................................................ucucacuaccuugucuuugacg....................    37684  0    S05
......................................................................ucucacuaccuugucuuugacgg...................    1      0    S05
.......................................................................cucacuaccuugucuuugacg....................    29     0    S05
........................................................................ucacuaccuugucuuugacg....................    20     0    S05
........................................................................ucacuaccuugucuuugacgga..................    1      0    S05
.........................................................................cacuaccuugucuuugacg....................    8      0    S05
..........................................................................acuaccuugucuuugacg....................    2      0    S05

.........uucuuuaaccuuagugauuuuacug..............................................................................    1      0    S10
............uuuaaccuuagugauuuuacug..............................................................................    1      0    S10
.............uuaaccuuagugauuuuacug..............................................................................    5      0    S10
..............uaaccuuagugauuuuac................................................................................    1      0    S10
..............uaaccuuagugauuuuacu...............................................................................    1      0    S10
..............uaaccuuagugauuuuacug..............................................................................    17     0    S10
...............aaccuuagugauuuuacug..............................................................................    30     0    S10
```

Star Mature

aguaaauauuucuuuaaccuuagugauuuuacugugaaagacauggguagugagaugucuuaauuccccaucucacuaccuugucuuugacggaaagauuacuugaaucuuc			
................accuuagugauuuuacug..	11	0	S10
................................ugugaaagacauggguaguga...	1	0	S10
................................ugugaaagacauggguagugaga...	5	0	S10
..................................ugaaagacauggguagug..	1	0	S10
..................................ugaaagacauggguaguga...	1	0	S10
..................................ugaaagacauggguagugag..	8	0	S10
..................................ugaaagacauggguagugaga...	146	0	S10
..................................ugaaagacauggguagugagau..	899	0	S10
..................................ugaaagacauggguagugagaug...	9250	0	S10
..................................ugaaagacauggguagugagaugu..	301	0	S10
..................................ugaaagacauggguagugagauguc...	3	0	S10
...................................gaaagacauggguagugagau..	2	0	S10
...................................gaaagacauggguagugagaug...	6	0	S10
...................................gaaagacauggguagugagaugu..	1	0	S10
.....................................aagacauggguagugagaug...	1	0	S10
......................................agacauggguagugagaug...	1	0	S10
....ucuuaauuccccaucucacuaccuugucuuugacg....................	1	0	S10
....caucucacuaccuugucuuug.......................	1	0	S10
....caucucacuaccuugucuuuga......................	2	0	S10
....caucucacuaccuugucuuugac.....................	5	0	S10
....aucucacuaccuugucuuuga......................	1	0	S10
....aucucacuaccuugucuuugac.....................	42	0	S10
....aucucacuaccuugucuuugacg....................	3	0	S10
....ucucacuaccuugucuuu........................	2	0	S10
....ucucacuaccuugucuuug.......................	8	0	S10
....ucucacuaccuugucuuuga......................	105	0	S10
....ucucacuaccuugucuuugac.....................	2956	0	S10
....ucucacuaccuugucuuugacg....................	62865	0	S10
....ucucacuaccuugucuuugacgg...................	2	0	S10
....cucacuaccuugucuuugac.....................	5	0	S10
....cucacuaccuugucuuugacg....................	50	0	S10
....ucacuaccuugucuuugac.....................	1	0	S10
....ucacuaccuugucuuugacg....................	37	0	S10
....ucacuaccuugucuuugacgga..................	10	0	S10
....cacuaccuugucuuugac.....................	1	0	S10
....cacuaccuugucuuugacg....................	9	0	S10
....acuaccuugucuuugacg....................	11	0	S10
.............uuaaccuuagugauuuuacug..	1	0	S02
..............uaaccuuagugauuuuacug..	1	0	S02
...............aaccuuagugauuuuacug..	1	0	S02
..................................ugaaagacauggguagugaga...	2	0	S02
..................................ugaaagacauggguagugagau..	18	0	S02
..................................ugaaagacauggguagugagaug...	41	0	S02
..................................ugaaagacauggguagugagaugu..	2	0	S02
....aucucacuaccuugucuuugac.....................	1	0	S02
....ucucacuaccuugucuuuga......................	5	0	S02
....ucucacuaccuugucuuugac.....................	104	0	S02
....ucucacuaccuugucuuugacg....................	14489	0	S02
....ucucacuaccuugucuuugacgg...................	1	0	S02
....cucacuaccuugucuuugacg....................	7	0	S02
....ucacuaccuugucuuugacg....................	2	0	S02
....acuaccuugucuuugacg....................	1	0	S02
............uuuaaccuuagugauuuuacug..	2	0	S04
.............uuaaccuuagugauuuuacug..	1	0	S04
..............uaaccuuagugauuuuacug..	4	0	S04
...............aaccuuagugauuuuacug..	7	0	S04
................accuuagugauuuuacug..	2	0	S04
...................uuagugauuuuacugugaaagac..	1	0	S04
..................................ugaaagacauggguagugaga...	5	0	S04
..................................ugaaagacauggguagugagau..	28	0	S04
..................................ugaaagacauggguagugagaug...	284	0	S04
..................................ugaaagacauggguagugagaugu..	10	0	S04
..................................ugaaagacauggguagugagauguc...	1	0	S04
....aucucacuaccuugucuuugac.....................	12	0	S04
....aucucacuaccuugucuuugacg....................	1	0	S04
....ucucacuaccuugucuuug.......................	2	0	S04
....ucucacuaccuugucuuuga......................	15	0	S04
....ucucacuaccuugucuuugac.....................	509	0	S04

Star   Mature

```
aguaaauauuucuuuaaccuuagugauuuuacugugaaagacauggguagugagaugucuuaauuccccaucucacuaccuugucuuugacggaaagauuacuugaaucuuc
......................................................................ucucacuaccuugucuuugacg....................    36255    0    S04
......................................................................ucucacuaccuugucuuugacgg...................    1        0    S04
.......................................................................cucacuaccuugucuuugacg....................    26       0    S04
........................................................................ucacuaccuugucuuugac.....................    1        0    S04
........................................................................ucacuaccuugucuuugacg....................    28       0    S04
........................................................................ucacuaccuugucuuugacgga..................    1        0    S04
.........................................................................cacuaccuugucuuugacg....................    4        0    S04
..........................................................................acuaccuugucuuugacg....................    5        0    S04

.....auauuucuuuaaccuuagugauuuuacu...............................................................................    1        0    S09
.........uucuuuaaccuuagugauuuuacug..............................................................................    2        0    S09
............uuuaaccuuagugauuuuacug..............................................................................    4        0    S09
.............uuaaccuuagugauuuuacug..............................................................................    12       0    S09
..............uaaccuuagugauuuuacug..............................................................................    22       0    S09
...............aaccuuagugauuuuacug..............................................................................    54       0    S09
................accuuagugauuuuacug..............................................................................    18       0    S09
................................ugugaaagacauggguagugaga.........................................................    6        0    S09
..................................ugaaagacauggguagug............................................................    1        0    S09
..................................ugaaagacauggguaguga...........................................................    2        0    S09
..................................ugaaagacauggguagugag..........................................................    3        0    S09
..................................ugaaagacauggguagugaga.........................................................    82       0    S09
..................................ugaaagacauggguagugagau........................................................    647      0    S09
..................................ugaaagacauggguagugagaug.......................................................    7320     0    S09
..................................ugaaagacauggguagugagaugu......................................................    261      0    S09
..................................ugaaagacauggguagugagauguc.....................................................    5        0    S09
..................................ugaaagacauggguagugagaugucuuaauucccc...........................................    1        0    S09
..................................ugaaagacauggguagugagaugucuuaauuccccaucucacuaccuugucuuugacg....................    9        0    S09
...................................gaaagacauggguagugagaug.......................................................    6        0    S09
.....................................aagacauggguagugagaug.......................................................    1        0    S09
......................................agacauggguagugagaug.......................................................    2        0    S09
.......................................gacauggguagugagaug.......................................................    4        0    S09
.........................................................ucuuaauuccccaucucacuaccuugucuuuga......................    1        0    S09
.........................................................ucuuaauuccccaucucacuaccuugucuuugac.....................    1        0    S09
.........................................................ucuuaauuccccaucucacuaccuugucuuugacg....................    3        0    S09
....................................................................caucucacuaccuugucuuug.......................    2        0    S09
....................................................................caucucacuaccuugucuuuga......................    3        0    S09
....................................................................caucucacuaccuugucuuugac.....................    6        0    S09
.....................................................................aucucacuaccuugucuuuga......................    4        0    S09
.....................................................................aucucacuaccuugucuuugac.....................    91       0    S09
.....................................................................aucucacuaccuugucuuugacg....................    5        0    S09
......................................................................ucucacuaccuugucuuu........................    2        0    S09
......................................................................ucucacuaccuugucuuug.......................    1        0    S09
......................................................................ucucacuaccuugucuuuga......................    163      0    S09
......................................................................ucucacuaccuugucuuugac.....................    3420     0    S09
......................................................................ucucacuaccuugucuuugacg....................    81965    0    S09
......................................................................ucucacuaccuugucuuugacgg...................    6        0    S09
.......................................................................cucacuaccuugucuuugac.....................    5        0    S09
.......................................................................cucacuaccuugucuuugacg....................    42       0    S09
........................................................................ucacuaccuugucuuugac.....................    1        0    S09
........................................................................ucacuaccuugucuuugacg....................    39       0    S09
........................................................................ucacuaccuugucuuugacgg...................    2        0    S09
........................................................................ucacuaccuugucuuugacgga..................    14       0    S09
.........................................................................cacuaccuugucuuugacg....................    11       0    S09
..........................................................................acuaccuugucuuugacg....................    12       0    S09

.............uuaaccuuagugauuuuacug..............................................................................    1        0    S03
..............uaaccuuagugauuuuacug..............................................................................    1        0    S03
...............aaccuuagugauuuuacug..............................................................................    3        0    S03
..................................ugaaagacauggguagugag..........................................................    1        0    S03
..................................ugaaagacauggguagugaga.........................................................    7        0    S03
..................................ugaaagacauggguagugagau........................................................    19       0    S03
..................................ugaaagacauggguagugagaug.......................................................    177      0    S03
..................................ugaaagacauggguagugagaugu......................................................    8        0    S03
.........................................................ucuuaauuccccaucucacuaccuugucuuugacg....................    1        0    S03
.....................................................................aucucacuaccuugucuuugac.....................    3        0    S03
.....................................................................aucucacuaccuugucuuugacg....................    1        0    S03
......................................................................ucucacuaccuugucuuug.......................    1        0    S03
......................................................................ucucacuaccuugucuuuga......................    9        0    S03
......................................................................ucucacuaccuugucuuugac.....................    461      0    S03
......................................................................ucucacuaccuugucuuugacg....................    30830    0    S03
```

```
                                                   Star                                Mature
aguaaauauuucuuuaaccuuagugauuuuacugugaaagacauggguagugagaugucuuaauuccccaucucacuaccuugucuuugacggaaagauuacuugaaucuuc
......................................................................ucucacuaccuugucuuugacgg...................    1       0       S03
.......................................................................cucacuaccuugucuuugacg....................    15      0       S03
........................................................................ucacuaccuugucuuugacg....................    17      0       S03
........................................................................ucacuaccuugucuuugacgga..................    1       0       S03
.........................................................................cacuaccuugucuuugacg....................    3       0       S03
..........................................................................acuaccuugucuuugacg....................    1       0       S03

..........ucuuuaaccuuagugauuuuacug..............................................................................    2       0       S08
............uuuaaccuuagugauuuuacug..............................................................................    2       0       S08
.............uuaaccuuagugauuuuacug..............................................................................    2       0       S08
..............uaaccuuagugauuuuacug..............................................................................    6       0       S08
...............aaccuuagugauuuuacug..............................................................................    8       0       S08
................accuuagugauuuuacug..............................................................................    11      0       S08
................................ugugaaagacauggguagugaga.........................................................    1       0       S08
..................................ugaaagacauggguagugaga.........................................................    16      0       S08
..................................ugaaagacauggguagugagau........................................................    129     0       S08
..................................ugaaagacauggguagugagaug.......................................................    1380    0       S08
..................................ugaaagacauggguagugagaugu......................................................    46      0       S08
...................................gaaagacauggguagugagaug.......................................................    2       0       S08
...................................gaaagacauggguagugagaugu......................................................    2       0       S08
..........................................................cuuaauuccccaucucacuaccuugucuuugac.....................    1       0       S08
...............................................................uuccccaucucacuaccuugucuuugacg....................    1       0       S08
....................................................................caucucacuaccuugucuuuga......................    1       0       S08
....................................................................caucucacuaccuugucuuugac.....................    1       0       S08
.....................................................................aucucacuaccuugucuuuga......................    4       0       S08
.....................................................................aucucacuaccuugucuuugac.....................    19      0       S08
......................................................................ucucacuaccuugucuuug.......................    17      0       S08
......................................................................ucucacuaccuugucuuuga......................    162     0       S08
......................................................................ucucacuaccuugucuuugac.....................    1792    0       S08
......................................................................ucucacuaccuugucuuugacg....................    68756   0       S08
......................................................................ucucacuaccuugucuuugacgg...................    4       0       S08
.......................................................................cucacuaccuugucuuuga......................    1       0       S08
.......................................................................cucacuaccuugucuuugac.....................    2       0       S08
.......................................................................cucacuaccuugucuuugacg....................    57      0       S08
........................................................................ucacuaccuugucuuugac.....................    1       0       S08
........................................................................ucacuaccuugucuuugacg....................    51      0       S08
........................................................................ucacuaccuugucuuugacgga..................    1       0       S08
.........................................................................cacuaccuugucuuugacg....................    33      0       S08
..........................................................................acuaccuugucuuugacg....................    11      0       S08
```

Provisional ID	:	Scaffold300_7825
Score total	:	270330.2
Score for star read(s)	:	3.9
Score for read counts	:	270322.7
Score for mfe	:	2
Score for randfold	:	1.6
Score for cons. seed	:	
Total read count	:	530238
Mature read count	:	505688
Loop read count	:	1
Star read count	:	24549

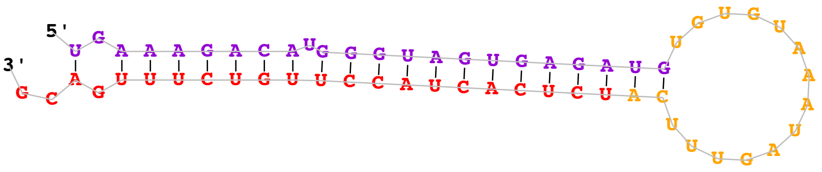

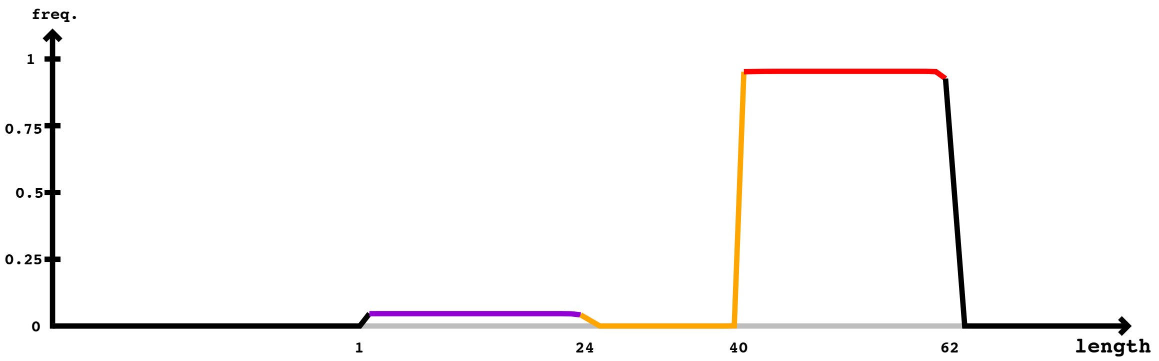

Star **Mature**

```
5'- ucuaacaggaggucagaaugcuuuuguucugugaaagacauggguagugagauguguguaaaauaguuucaucucacuaccuugucuuugacggaaaguggcacacaccca  -3'    obs
    ucuaacaggaggucagaaugcuuuuguucugugaaagacauggguagugagauguguguaaaauaguuucaucucacuaccuugucuuugacggaaaguggcacacaccca         exp
    .......((..((.....((((....((((((.(((((((.((((((((((((.................))))))))))))))))))).)))))))...)))).))..)).  reads   mm   sample
    .............................ugugaaagacauggguagugaga...........................................................  2       0    S07
    ...............................ugaaagacauggguagugag............................................................  3       0    S07
    ...............................ugaaagacauggguagugaga...........................................................  25      0    S07
    ...............................ugaaagacauggguagugagau..........................................................  202     0    S07
    ...............................ugaaagacauggguagugagaug.........................................................  2347    0    S07
    ...............................ugaaagacauggguagugagaugu........................................................  94      0    S07
    ...............................ugaaagacauggguagugagaugug.......................................................  1       0    S07
    ................................gaaagacauggguagugagaug.........................................................  2       0    S07
    ........................................................uguaaaauaguuucaucucacuaccuugucuuugacg..................  1       0    S07
    .....................................................................caucucacuaccuugucuuug.....................  1       0    S07
    .....................................................................caucucacuaccuugucuuuga....................  3       0    S07
    .....................................................................caucucacuaccuugucuuugac...................  3       0    S07
    ......................................................................aucucacuaccuugucuuuga....................  6       0    S07
    ......................................................................aucucacuaccuugucuuugac...................  35      0    S07
    ......................................................................aucucacuaccuugucuuugacg..................  2       0    S07
    .......................................................................ucucacuaccuugucuuug.....................  1       0    S07
    .......................................................................ucucacuaccuugucuuuga....................  139     0    S07
    .......................................................................ucucacuaccuugucuuugac...................  2135    0    S07
    .......................................................................ucucacuaccuugucuuugacg..................  87699   0    S07
    .......................................................................ucucacuaccuugucuuugacgg.................  8       0    S07
    ........................................................................cucacuaccuugucuuugac...................  2       0    S07
    ........................................................................cucacuaccuugucuuugacg..................  38      0    S07
    .........................................................................ucacuaccuugucuuugac...................  2       0    S07
    .........................................................................ucacuaccuugucuuugacg..................  50      0    S07
    ..........................................................................cacuaccuugucuuugacg..................  5       0    S07
    ...........................................................................acuaccuugucuuugacg..................  5       0    S07

    ...............................ugaaagacauggguagugaga...........................................................  3       0    S01
    ...............................ugaaagacauggguagugagau..........................................................  8       0    S01
    ...............................ugaaagacauggguagugagaug.........................................................  27      0    S01
    ..................................................................uuucaucucacuaccuugucuuugacg..................  1       0    S01
    .......................................................................ucucacuaccuugucuuuga....................  3       0    S01
    .......................................................................ucucacuaccuugucuuugac...................  56      0    S01
```

```
                                 Star                                   Mature
ucuaacaggaggucagaaugcuuuuguucugugaaagacauggguagugagauguguguaaauaguuucaucucacuaccuugucuuugacggaaaguggcacacaccca
......................................................................ucucacuaccuugucuuugacg..................  8319   0  S01
......................................................................ucucacuaccuugucuuugacgg.................  1      0  S01
.......................................................................cucacuaccuugucuuugacg..................  1      0  S01
........................................................................ucacuaccuugucuuugacg..................  9      0  S01
.........................................................................cacuaccuugucuuugacg..................  7      0  S01
..........................................................................acuaccuugucuuugacg..................  3      0  S01

.cuaacaggaggucagaaugcuuuuguucug...............................................................................  1      0  S06
...............................ugaaagacauggguagugaga..........................................................  4      0  S06
...............................ugaaagacauggguagugagau.........................................................  60     0  S06
...............................ugaaagacauggguagugagaug........................................................  344    0  S06
...............................ugaaagacauggguagugagaugu.......................................................  19     0  S06
................................gaaagacauggguagugagaugu.......................................................  1      0  S06
....................................................................caucucacuaccuugucuuuga....................  1      0  S06
....................................................................caucucacuaccuugucuuugac...................  3      0  S06
.....................................................................aucucacuaccuugucuuugac...................  25     0  S06
.....................................................................aucucacuaccuugucuuugacg..................  2      0  S06
......................................................................ucucacuaccuugucuuug.....................  4      0  S06
......................................................................ucucacuaccuugucuuuga....................  19     0  S06
......................................................................ucucacuaccuugucuuugac...................  1289   0  S06
......................................................................ucucacuaccuugucuuugacg..................  61603  0  S06
.......................................................................cucacuaccuugucuuugac...................  3      0  S06
.......................................................................cucacuaccuugucuuugacg..................  40     0  S06
........................................................................ucacuaccuugucuuugacg..................  36     0  S06
........................................................................ucacuaccuugucuuugacgg.................  2      0  S06
.........................................................................cacuaccuugucuuugacg..................  11     0  S06
..........................................................................acuaccuugucuuugacg..................  4      0  S06

.........aggucagaaugcuuuuguucug...............................................................................  1      0  S10
...........gucagaaugcuuuuguucug...............................................................................  1      0  S10
............ucagaaugcuuuuguucug...............................................................................  2      0  S10
.............................ugugaaagacauggguaguga............................................................  1      0  S10
.............................ugugaaagacauggguagugaga..........................................................  5      0  S10
...............................ugaaagacauggguagug.............................................................  1      0  S10
...............................ugaaagacauggguaguga............................................................  1      0  S10
...............................ugaaagacauggguagugag...........................................................  8      0  S10
...............................ugaaagacauggguagugaga..........................................................  146    0  S10
...............................ugaaagacauggguagugagau.........................................................  899    0  S10
...............................ugaaagacauggguagugagaug........................................................  9250   0  S10
...............................ugaaagacauggguagugagaugu.......................................................  301    0  S10
...............................ugaaagacauggguagugagaugugu.....................................................  1      0  S10
................................gaaagacauggguagugagau.........................................................  2      0  S10
................................gaaagacauggguagugagaug........................................................  6      0  S10
................................gaaagacauggguagugagaugu.......................................................  1      0  S10
..................................aagacauggguagugagaug........................................................  1      0  S10
...................................agacauggguagugagaug........................................................  1      0  S10
......................................................uguguaaauaguuucauc......................................  1      0  S10
........................................................uguaaauaguuucaucucacuaccuugucuuugacg..................  1      0  S10
...................................................................ucaucucacuaccuugucuuugacg..................  1      0  S10
....................................................................caucucacuaccuugucuuug.....................  1      0  S10
....................................................................caucucacuaccuugucuuuga....................  2      0  S10
....................................................................caucucacuaccuugucuuugac...................  5      0  S10
.....................................................................aucucacuaccuugucuuuga....................  1      0  S10
.....................................................................aucucacuaccuugucuuugac...................  42     0  S10
.....................................................................aucucacuaccuugucuuugacg..................  3      0  S10
......................................................................ucucacuaccuugucuuu......................  2      0  S10
......................................................................ucucacuaccuugucuuug.....................  8      0  S10
......................................................................ucucacuaccuugucuuuga....................  105    0  S10
......................................................................ucucacuaccuugucuuugac...................  2956   0  S10
......................................................................ucucacuaccuugucuuugacg..................  62865  0  S10
......................................................................ucucacuaccuugucuuugacgg.................  2      0  S10
.......................................................................cucacuaccuugucuuugac...................  5      0  S10
.......................................................................cucacuaccuugucuuugacg..................  50     0  S10
........................................................................ucacuaccuugucuuugac...................  1      0  S10
........................................................................ucacuaccuugucuuugacg..................  37     0  S10
........................................................................ucacuaccuugucuuugacgga................  10     0  S10
.........................................................................cacuaccuugucuuugac...................  1      0  S10
.........................................................................cacuaccuugucuuugacg..................  9      0  S10
..........................................................................acuaccuugucuuugacg..................  11     0  S10

...............................ugaaagacauggguagugaga..........................................................  5      0  S05
```

Star **Mature**

```
ucuaacaggaggucagaaugcuuuuguucugugaaagacauggguagugagauguguguaaauaguuucaucucacuaccuugucuuugacggaaaguggcacacaccca
...............................ugaaagacauggguagugagau.........................................................  32     0  S05
...............................ugaaagacauggguagugagaug........................................................  221    0  S05
...............................ugaaagacauggguagugagaugu.......................................................  10     0  S05
................................gaaagacauggguagugagaug........................................................  1      0  S05
....................................................................caucucacuaccuugucuuugac...................  1      0  S05
.....................................................................aucucacuaccuugucuuugac...................  11     0  S05
.....................................................................aucucacuaccuugucuuugacg..................  3      0  S05
......................................................................ucucacuaccuugucuuug.....................  1      0  S05
......................................................................ucucacuaccuugucuuuga....................  9      0  S05
......................................................................ucucacuaccuugucuuugac...................  707    0  S05
......................................................................ucucacuaccuugucuuugacg..................  37684  0  S05
......................................................................ucucacuaccuugucuuugacgg.................  1      0  S05
.......................................................................cucacuaccuugucuuugacg..................  29     0  S05
........................................................................ucacuaccuugucuuugacg..................  20     0  S05
........................................................................ucacuaccuugucuuugacgga................  1      0  S05
.........................................................................cacuaccuugucuuugacg..................  8      0  S05
..........................................................................acuaccuugucuuugacg..................  2      0  S05

...............................ugaaagacauggguagugaga..........................................................  2      0  S02
...............................ugaaagacauggguagugagau.........................................................  18     0  S02
...............................ugaaagacauggguagugagaug........................................................  41     0  S02
...............................ugaaagacauggguagugagaugu.......................................................  2      0  S02
.....................................................................aucucacuaccuugucuuugac...................  1      0  S02
......................................................................ucucacuaccuugucuuuga....................  5      0  S02
......................................................................ucucacuaccuugucuuugac...................  104    0  S02
......................................................................ucucacuaccuugucuuugacg..................  14489  0  S02
......................................................................ucucacuaccuugucuuugacgg.................  1      0  S02
.......................................................................cucacuaccuugucuuugacg..................  7      0  S02
........................................................................ucacuaccuugucuuugacg..................  2      0  S02
..........................................................................acuaccuugucuuugacg..................  1      0  S02

...............................ugaaagacauggguagugaga..........................................................  5      0  S04
...............................ugaaagacauggguagugagau.........................................................  28     0  S04
...............................ugaaagacauggguagugagaug........................................................  284    0  S04
...............................ugaaagacauggguagugagaugu.......................................................  10     0  S04
..........................................................uaaauaguuucaucucacuaccuugucuuugacg..................  1      0  S04
.....................................................................aucucacuaccuugucuuugac...................  12     0  S04
.....................................................................aucucacuaccuugucuuugacg..................  1      0  S04
......................................................................ucucacuaccuugucuuug.....................  2      0  S04
......................................................................ucucacuaccuugucuuuga....................  15     0  S04
......................................................................ucucacuaccuugucuuugac...................  509    0  S04
......................................................................ucucacuaccuugucuuugacg..................  36255  0  S04
......................................................................ucucacuaccuugucuuugacgg.................  1      0  S04
.......................................................................cucacuaccuugucuuugacg..................  26     0  S04
........................................................................ucacuaccuugucuuugac...................  1      0  S04
........................................................................ucacuaccuugucuuugacg..................  28     0  S04
........................................................................ucacuaccuugucuuugacgga................  1      0  S04
.........................................................................cacuaccuugucuuugacg..................  4      0  S04
..........................................................................acuaccuugucuuugacg..................  5      0  S04

......aggaggucagaaugcuuuuguucug...............................................................................  1      0  S08
.............................ugugaaagacauggguagugaga..........................................................  1      0  S08
...............................ugaaagacauggguagugaga..........................................................  16     0  S08
...............................ugaaagacauggguagugagau.........................................................  129    0  S08
...............................ugaaagacauggguagugagaug........................................................  1380   0  S08
...............................ugaaagacauggguagugagaugu.......................................................  46     0  S08
...............................ugaaagacauggguagugagaugugu.....................................................  1      0  S08
................................gaaagacauggguagugagaug........................................................  2      0  S08
................................gaaagacauggguagugagaugu.......................................................  2      0  S08
............................................................aauaguuucaucucacuaccuugucuuugacg..................  1      0  S08
..............................................................uaguuucaucucacuaccuugucuuugacg..................  1      0  S08
....................................................................caucucacuaccuugucuuuga....................  1      0  S08
....................................................................caucucacuaccuugucuuugac...................  1      0  S08
.....................................................................aucucacuaccuugucuuuga....................  4      0  S08
.....................................................................aucucacuaccuugucuuugac...................  19     0  S08
......................................................................ucucacuaccuugucuuug.....................  17     0  S08
......................................................................ucucacuaccuugucuuuga....................  162    0  S08
......................................................................ucucacuaccuugucuuugac...................  1792   0  S08
......................................................................ucucacuaccuugucuuugacg..................  68756  0  S08
......................................................................ucucacuaccuugucuuugacgg.................  4      0  S08
```

Star / Mature			
ucuaacaggaggucagaaugcuuuuguucugugaaagacauggguagugagauguguguaaauaguuucaucucacuaccuugucuuugacggaaaguggcacacaccca			
....cucacuaccuugucuuuga....................	1	0	S08
....cucacuaccuugucuuugac...................	2	0	S08
....cucacuaccuugucuuugacg..................	57	0	S08
....ucacuaccuugucuuugac...................	1	0	S08
....ucacuaccuugucuuugacg..................	51	0	S08
....ucacuaccuugucuuugacgga................	1	0	S08
....cacuaccuugucuuugacg..................	33	0	S08
....acuaccuugucuuugacg..................	11	0	S08
........gaggucagaaugcuuuuguucug...	1	0	S09
.........aggucagaaugcuuuuguucug...	1	0	S09
............ucagaaugcuuuuguucug...	1	0	S09
.............................ugugaaagacauggguagugaga..	6	0	S09
...............................ugaaagacauggguagug...	1	0	S09
...............................ugaaagacauggguaguga..	2	0	S09
...............................ugaaagacauggguagugag...	3	0	S09
...............................ugaaagacauggguagugaga..	82	0	S09
...............................ugaaagacauggguagugagau...	647	0	S09
...............................ugaaagacauggguagugagaug..	7320	0	S09
...............................ugaaagacauggguagugagaugu...	261	0	S09
................................gaaagacauggguagugagaug..	6	0	S09
..................................aagacauggguagugagaug..	1	0	S09
...................................agacauggguagugagaug..	2	0	S09
....................................gacauggguagugagaug..	4	0	S09
....uguaaauaguuucaucucacuaccuugucuuugacg..................	2	0	S09
....caucucacuaccuugucuuug.....................	2	0	S09
....caucucacuaccuugucuuuga....................	3	0	S09
....caucucacuaccuugucuuugac...................	6	0	S09
....aucucacuaccuugucuuuga....................	4	0	S09
....aucucacuaccuugucuuugac...................	91	0	S09
....aucucacuaccuugucuuugacg..................	5	0	S09
....ucucacuaccuugucuuu......................	2	0	S09
....ucucacuaccuugucuuug.....................	1	0	S09
....ucucacuaccuugucuuuga....................	163	0	S09
....ucucacuaccuugucuuugac...................	3420	0	S09
....ucucacuaccuugucuuugacg..................	81965	0	S09
....ucucacuaccuugucuuugacgg.................	6	0	S09
....cucacuaccuugucuuugac...................	5	0	S09
....cucacuaccuugucuuugacg..................	42	0	S09
....ucacuaccuugucuuugac...................	1	0	S09
....ucacuaccuugucuuugacg..................	39	0	S09
....ucacuaccuugucuuugacgg.................	2	0	S09
....ucacuaccuugucuuugacgga................	14	0	S09
....cacuaccuugucuuugacg..................	11	0	S09
....acuaccuugucuuugacg..................	12	0	S09
...............................ugaaagacauggguagugag...	1	0	S03
...............................ugaaagacauggguagugaga..	7	0	S03
...............................ugaaagacauggguagugagau...	19	0	S03
...............................ugaaagacauggguagugagaug..	177	0	S03
...............................ugaaagacauggguagugagaugu...	8	0	S03
....uguaaauaguuucaucucacuaccuugucuuugacg..................	1	0	S03
....aucucacuaccuugucuuugac...................	3	0	S03
....aucucacuaccuugucuuugacg..................	1	0	S03
....ucucacuaccuugucuuug.....................	1	0	S03
....ucucacuaccuugucuuuga....................	9	0	S03
....ucucacuaccuugucuuugac...................	461	0	S03
....ucucacuaccuugucuuugacg..................	30830	0	S03
....ucucacuaccuugucuuugacgg.................	1	0	S03
....cucacuaccuugucuuugacg..................	15	0	S03
....ucacuaccuugucuuugacg..................	17	0	S03
....ucacuaccuugucuuugacgga................	1	0	S03
....cacuaccuugucuuugacg..................	3	0	S03
....acuaccuugucuuugacg..................	1	0	S03

Provisional ID	:	Scaffold15_617
Score total	:	965.9
Score for star read(s)	:	3.9
Score for read counts	:	958.6
Score for mfe	:	1.8
Score for randfold	:	1.6
Score for cons. seed	:	
Total read count	:	1892
Mature read count	:	1706
Loop read count	:	0
Star read count	:	186

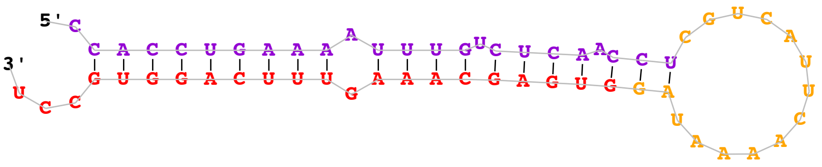

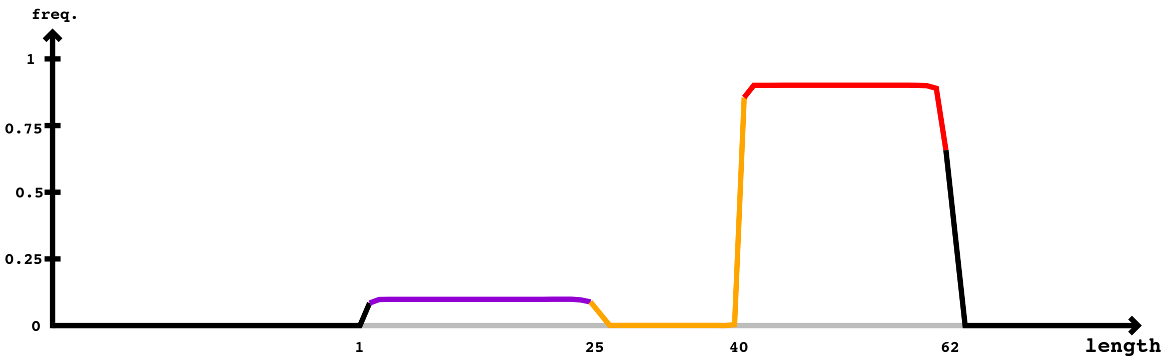


```
                               Star                                   Mature
5'- acuaguaucaacucuuaagggccuauucuaaccaccugaaaauuugucucaaccucgucauucaaaauaggugagcaaaguuucaggugccuagaguaggcuuacuccaaa  -3'  obs
    acuaguaucaacucuuaagggccuauucuaaccaccugaaaauuugucucaaccucgucauucaaaauaggugagcaaaguuucaggugccuagaguaggcuuacuccaaa       exp
    ..................((((((((((((..(((((((((.((((.((((.(((.............))))))))))).)))))))))..))))))))))))........      reads  mm  sample
    ...............................ccaccugaaaauuugucucaaccu........................................................      6      0   S02
    .....................................................................ggugagcaaaguuucaggugcc....................      1      0   S02
    ......................................................................gugagcaaaguuucaggugcc....................      2      0   S02
    ......................................................................gugagcaaaguuucaggugccu...................      7      0   S02
    .......................................................................ugagcaaaguuucaggugccu...................      2      0   S02

    ...............................ccaccugaaaauuugucucaaccu........................................................      4      0   S04
    ................................caccugaaaauuugucucaaccu........................................................      1      0   S04
    ..................................................aaccAcgucauucaaaau...........................................      1      1   S04
    ......................................................................gugagcaaaguuucaggugc.....................      2      0   S04
    ......................................................................gugagcaaaguuucaggugcc....................      4      0   S04
    ......................................................................gugagcaaaguuucaggugccu...................      18     0   S04
    .......................................................................ugagcaaaguuucaggugccu...................      4      0   S04

    ...............................ccaccugaaaauuugucucaacc.........................................................      1      0   S09
    ...............................ccaccugaaaauuugucucaaccu........................................................      19     0   S09
    ................................caccugaaaauuugucucaaccu........................................................      3      0   S09
    .....................................................................ggugagcaaaguuucaggugcc....................      2      0   S09
    .....................................................................ggugagcaaaguuucaggugccu...................      2      0   S09
    ......................................................................gugagcaaaguuucaggugc.....................      1      0   S09
    ......................................................................gugagcaaaguuucaggugcc....................      157    0   S09
    ......................................................................gugagcaaaguuucaggugccu...................      443    0   S09
    .......................................................................ugagcaaaguuucaggugc.....................      1      0   S09
    .......................................................................ugagcaaaguuucaggugccu...................      17     0   S09

    ...............................ccaccugaaaauuugucucaac..........................................................      1      0   S03
    ...............................ccaccugaaaauuugucucaaccu........................................................      4      0   S03
    ................................caccugaaaauuugucucaaccu........................................................      2      0   S03
    ......................................................................gugagcaaaguuucaggu.......................      1      0   S03
    ......................................................................gugagcaaaguuucaggugc.....................      3      0   S03
    ......................................................................gugagcaaaguuucaggugcc....................      10     0   S03
    ......................................................................gugagcaaaguuucaggugccu...................      16     0   S03
    .......................................................................ugagcaaaguuucaggugccu...................      3      0   S03
```

Star **Mature**

acuaguaucaacucuuaagggccuauucuaaccaccugaaaauuugucucaaccucgucauucaaaauaggugagcaaaguuucaggugccuagaguaggcuuacuccaaa

...............................ccaccugaaaauuugucucaac..	1	0	S08
...............................ccaccugaaaauuugucucaacc...	1	0	S08
...............................ccaccugaaaauuugucucaaccu..	42	0	S08
................................caccugaaaauuugucucaaccu..	3	0	S08
....gugagcaaaguuucaggugc.....................	2	0	S08
....gugagcaaaguuucaggugcc....................	37	0	S08
....gugagcaaaguuucaggugccu...................	83	0	S08
....ugagcaaaguuucaggugccu...................	5	0	S08
...............................ccaccugaaaauuugucucaac..	1	0	S01
...............................ccaccugaaaauuugucucaacc...	3	0	S01
...............................ccaccugaaaauuugucucaaccu..	4	0	S01
................................caccugaaaauuugucucaacc...	1	0	S01
....gugagcaaaguuucaggugc.....................	1	0	S01
....gugagcaaaguuucaggugcc....................	4	0	S01
....gugagcaaaguuucaggugccu...................	3	0	S01
....ugagcaaaguuucaggugccu...................	5	0	S01
...............................ccaccugaaaauuugucucaaccu..	15	0	S06
................................caccugaaaauuugucucaaccu..	2	0	S06
.................................accugaaaauuugucucaac..	1	0	S06
....gugagcaaaguuucaggugc.....................	3	0	S06
....gugagcaaaguuucaggugcc....................	12	0	S06
....gugagcaaaguuucaggugccu...................	26	0	S06
....ugagcaaaguuucaggugccu...................	5	0	S06
...............................ccaccugaaaauuugucucaac..	1	0	S07
...............................ccaccugaaaauuugucucaacc...	3	0	S07
...............................ccaccugaaaauuugucucaaccu..	16	0	S07
................................caccugaaaauuugucucaaccu..	6	0	S07
....gugagcaaaguuucaggugc.....................	2	0	S07
....gugagcaaaguuucaggugcc....................	89	0	S07
....gugagcaaaguuucaggugccu...................	214	0	S07
....ugagcaaaguuucaggugccu...................	16	0	S07
....gcaaaguuucaggugccu...................	1	0	S07
...............................ccaccugaaaauuugucucaacc...	2	0	S05
...............................ccaccugaaaauuugucucaaccu..	3	0	S05
................................caccugaaaauuugucucaaccu..	1	0	S05
....gugagcaaaguuucaggugc.....................	1	0	S05
....gugagcaaaguuucaggugcc....................	8	0	S05
....gugagcaaaguuucaggugccu...................	16	0	S05
....ugagcaaaguuucaggugccu...................	3	0	S05
...............................ccaccugaaaauuugucucaacc...	1	0	S10
...............................ccaccugaaaauuugucucaaccu..	32	0	S10
................................caccugaaaauuugucucaacc...	2	0	S10
................................caccugaaaauuugucucaaccu..	4	0	S10
....ggugagcaaaguuucaggugc.....................	2	0	S10
....gugagcaaaguuucaggug......................	2	0	S10
....gugagcaaaguuucaggugc.....................	1	0	S10
....gugagcaaaguuucaggugcc....................	112	0	S10
....gugagcaaaguuucaggugccu...................	332	0	S10
....ugagcaaaguuucaggugcc....................	1	0	S10
....ugagcaaaguuucaggugccu...................	24	0	S10

Provisional ID	:	Scaffold15_615
Score total	:	194.4
Score for star read(s)	:	3.9
Score for read counts	:	187.2
Score for mfe	:	1.7
Score for randfold	:	1.6
Score for cons. seed	:	
Total read count	:	379
Mature read count	:	363
Loop read count	:	0
Star read count	:	16

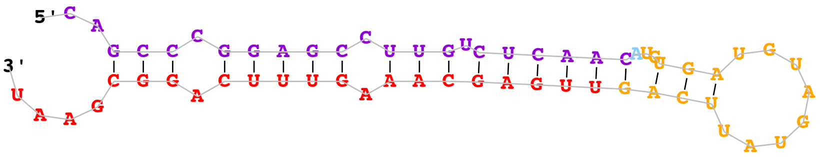

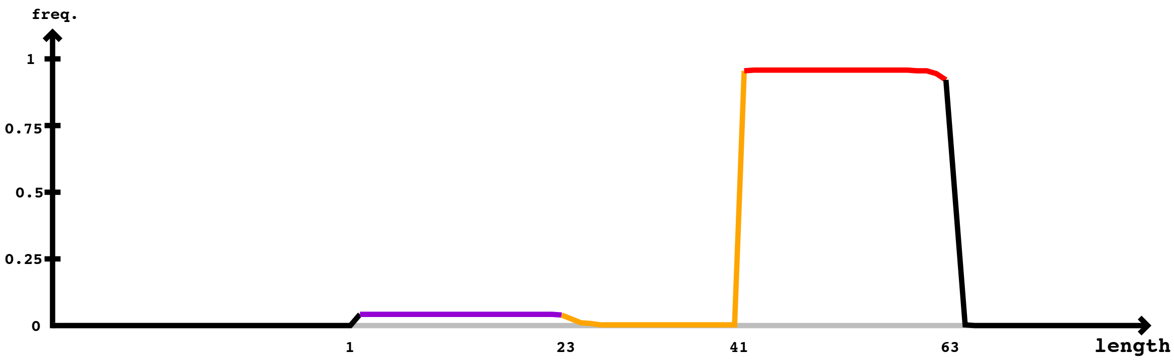

Star Mature

```
5'- cacuugguuuccugauaaugcgcaccgcaucagcccggagccuugucucaacaugugauguaguauucaguugagcaaaguuucaggcgaauucggugugugguuuuccuucu  -3'   obs
    cacuugguuuccugauaaugcgcaccgcaucagcccggagccuugucucaacaugugauguaguauucaguugagcaaaguuucaggcgaauucggugugugguuuuccuucu         exp
    ............((.((((((((((.((..(((.(((((.(((.((((((...(((........))))))))))).))))).))).)).))))))))))).)).....    reads   mm   sample
    ......................................................................uugagcaaaguuucaggcgaau.....................    25      0    S06

    ......................................................................uugagcaaaguuucaggcga.......................    1       0    S01
    ......................................................................uugagcaaaguuucaggcgaau.....................    9       0    S01

    ..............................cagcccggagccuugucucaac.............................................................    2       0    S07
    ..............................cagcccggagccuugucucaaca............................................................    1       0    S07
    ..............................cagcccggagccuugucucaacaug..........................................................    1       0    S07
    ......................................................................uugagcaaaguuucaggcgaa......................    1       0    S07
    ......................................................................uugagcaaaguuucaggcgaau.....................    43      0    S07
    ......................................................................uugagcaaaguuucaggcgaauu....................    1       0    S07

    ......................................................................uugagcaaaguuucaggcga.......................    1       0    S05
    ......................................................................uugagcaaaguuucaggcgaau.....................    16      0    S05

    ..............................cagcccggagccuugucucaa..............................................................    1       0    S10
    ..............................cagcccggagccuugucucaaca............................................................    1       0    S10
    ..............................cagcccggagccuugucucaacau...........................................................    1       0    S10
    .....................................................ugugauguaguauucaguugagcaaaguuucaggcgaau.....................    1       0    S10
    ......................................................................uugagcaaaguuucaggc.........................    1       0    S10
    ......................................................................uugagcaaaguuucaggcgaa......................    5       0    S10
    ......................................................................uugagcaaaguuucaggcgaau.....................    86      0    S10
    ......................................................................uugagcaaaguuucaggcgaauu....................    2       0    S10
    .......................................................................ugagcaaaguuucaggcgaau.....................    1       0    S10

    ......................................................................uugagcaaaguuucaggcgaa......................    1       0    S04
    ......................................................................uugagcaaaguuucaggcgaau.....................    10      0    S04
    ......................................................................uugagcaaaguuucaggcgaauuc...................    1       0    S04

    ......................................................................uugagcaaaguuucaggcgaau.....................    4       0    S02

    ......................................................................uugagcaaaguuucaggcga.......................    2       0    S03
    ......................................................................uugagcaaaguuucaggcgaau.....................    17      0    S03
```

```
                              Star                                        Mature
cacuugguuuccugauaaugcgcaccgcaucagcccggagccuugucucaacaugugauguaguauucaguugagcaaaguuucaggcgaauucggugugugguuuuccuucu
......................................................................uugagcaaaguuucaggcgaauu...................    1    0    S03

..............................cagcccggagccuugucucaac............................................................    4    0    S09
..............................cagcccggagccuugucucaaca...........................................................    1    0    S09
..............................cagcccggagccuugucucaacaug.........................................................    1    0    S09
......................................................................uugagcaaaguuucaggcgaa.....................    1    0    S09
......................................................................uugagcaaaguuucaggcgaau....................   93    0    S09
......................................................................uugagcaaaguuucaggcgaauu...................    2    0    S09

..............................cagcccggagccuugucucaac............................................................    2    0    S08
..............................cagcccggagccuugucucaaca...........................................................    1    0    S08
......................................................................uugagcaaaguuucaggcgaa.....................    1    0    S08
......................................................................uugagcaaaguuucaggcgaau....................   37    0    S08
......................................................................uugagcaaaguuucaggcgaauu...................    1    0    S08
```

Provisional ID	:	Scaffold31_1027
Score total	:	17.1
Score for star read(s)	:	3.9
Score for read counts	:	9.8
Score for mfe	:	1.8
Score for randfold	:	1.6
Score for cons. seed	:	
Total read count	:	31
Mature read count	:	27
Loop read count	:	0
Star read count	:	4

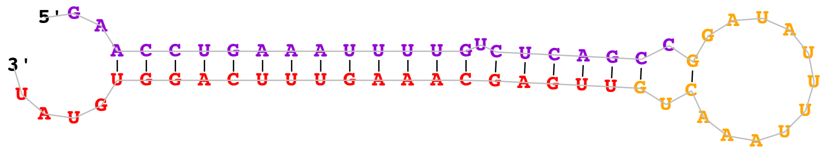

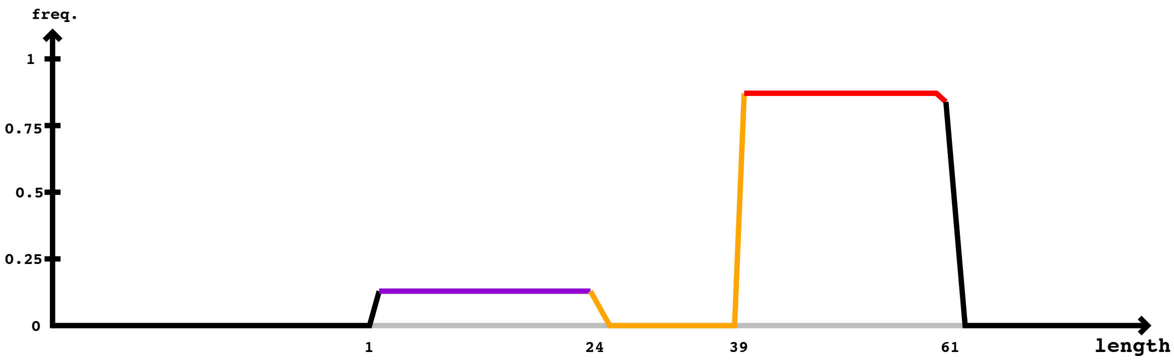


```
                                Star                                  Mature
5'- gagacgaggugucccguuuuguuugauccgaugaaccugaaauuuugucucagccggauauuuuaaacuguugagcaaaguuucagguguauuggcaucuaacgcaguucua   -3'   obs
    gagacgaggugucccguuuuguuugauccgaugaaccugaaauuuugucucagccggauauuuuaaacuguugagcaaaguuucagguguauuggcaucuaacgcaguucua         exp
    ((((((.((...))))))))(((.(((((((((.((((((((((((((.((((((.(...........).)))))))))))))))))))).)))))).))).)))........   reads   mm   sample
    ......................................................................uugagcaaaguuucagguguau....................   1       0    S08

    ......................................................................uugagcaaaguuucagguguau....................   2       0    S03

    ................................gaaccugaaauuuugucucagccg........................................................   1       0    S09
    ......................................................................uugagcaaaguuucagguguau....................   8       0    S09

    ......................................................................uugagcaaaguuucagguguau....................   2       0    S02

    ................................gaaccugaaauuuugucucagcc.........................................................   1       0    S10
    ......................................................................uugagcaaaguuucagguguau....................   6       0    S10
    ......................................................................uugagcaaaguuucagguguauu...................   3       0    S10

    ................................gaaccugaaauuuugucucagcc.........................................................   1       0    S05

    ......................................................................uugagcaaaguuucagguguau....................   3       0    S07

    ................................gaaccugaaauuuugucucagcc.........................................................   1       0    S01
    ......................................................................uugagcaaaguuucagguguau....................   1       0    S01

    ......................................................................uugagcaaaguuucaggugua.....................   1       0    S06
```

Provisional ID	: Scaffold1813_30090
Score total	: 976014.9
Score for star read(s)	: 3.9
Score for read counts	: 976007.3
Score for mfe	: 2.1
Score for randfold	: 1.6
Score for cons. seed	:
Total read count	: 1914408
Mature read count	: 1903595
Loop read count	: 0
Star read count	: 10813

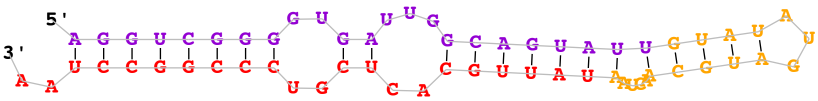

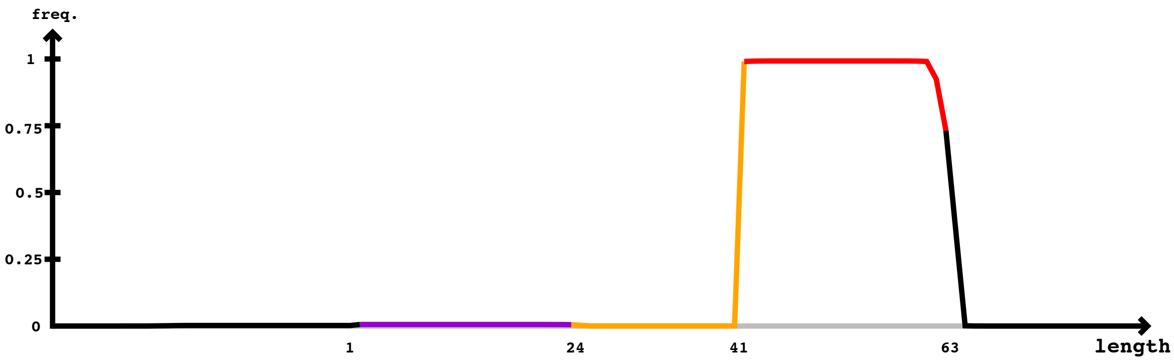

Star Mature

5'-	auacuuuggccugauaagccugcucuugccaggucggggugauuggcaguauuguauaugaugcaacuaauauugcacucgucccggccuaacaagggaggcgcaugcgccu	-3'	obs	
	auacuuuggccugauaagccugcucuugccaggucggggugauuggcaguauuguauaugaugcaacuaauauugcacucgucccggccuaacaagggaggcgcaugcgccu		exp	
	.......((((((....((((.((((((..((((((((..((...((((((((((((...)))))....)))))))..))..))))))))..))))))))))).)).).))).	reads	mm	sample
	auacuuuggccugauaagccugcucuugcc..	2	0	S08
	.uacuuuggccugauaagccugcucuugcc..	5	0	S08
	..acuuuggccugauaagccugcucuugcc..	1	0	S08
	...cuuuggccugauaagccugcucuugcc..	3	0	S08
	.....uuggccugauaagccugcucuugcc..	3	0	S08
	.....uuggccugauaagccugcucuugccaggucggggugauuggcaguauugu...	1	0	S08
	......uggccugauaagccugcucuugcc..	1	0	S08
	.......ggccugauaagccugcucuugcc..	1	0	S08
	........gccugauaagccugcucuugcc..	56	0	S08
	.........ccugauaagccugcucuugcc..	178	0	S08
	..........cugauaagccugcucuugcc..	60	0	S08
	...........ugauaagccugcucuugcc..	175	0	S08
	............gauaagccugcucuugcc..	21	0	S08
	.................gccugcucuugccaggucggggugauuggcaguauugua..	1	0	S08
	............................ccaggucggggugauuggcagu..	1	0	S08
	.............................caggucggggugauuggcagua...	3	0	S08
	.............................caggucggggugauuggcaguau..	1	0	S08
	.............................caggucggggugauuggcaguauu...	1	0	S08
	..............................aggucggggugauuggca..	13	0	S08
	..............................aggucggggugauuggcag...	11	0	S08
	..............................aggucggggugauuggcagu..	82	0	S08
	..............................aggucggggugauuggcagua...	30	0	S08
	..............................aggucggggugauuggcaguau..	303	0	S08
	..............................aggucggggugauuggcaguauu...	2228	0	S08
	..............................aggucggggugauuggcaguauugua..	1	0	S08
	..............................aggucggggugauuggcaguauuguaua..	1	0	S08
	..............................aggucggggugauuggcaguauuguauaugaugc..	3	0	S08
	..............................aggucggggugauuggcaguauuguauaugaugcaa..	1	0	S08
	..............................aggucggggugauuggcaguauuguauaugaugcaac...	5	0	S08
	..............................aggucggggugauuggcaguauuguauaugaugcaacu..	4	0	S08
	..............................aggucggggugauuggcaguauuguauaugaugcaacua...	2	0	S08
	..............................aggucggggugauuggcaguauuguauaugaugcaacuaa..	9	0	S08
	..............................aggucggggugauuggcaguauuguauaugaugcaacuaau...	5	0	S08

Star **Mature**

```
auacuuuggccugauaagccugcucuugccaggucggggugauuggcaguauuguauaugaugcaacuaauauugcacucgucccggccuaacaagggaggcgcaugcgccu
..............................aggucggggugauuggcaguauuguauaugaugcaacuaauauu......................................  1       0  S08
..............................aggucggggugauuggcaguauuguauaugaugcaacuaauauugcacucguc.............................  2       0  S08
.................................ucggggugauuggcaguauu...........................................................  1       0  S08
..................................cggggugauuggcaguauu...........................................................  1       0  S08
.......................................ugauuggcaguauuguauaugaugcaacuaauauugcacucgucccggcc.......................  1       0  S08
.......................................ugauuggcaguauuguauaugaugcaacuaauauugcacucgucccggccua.....................  1       0  S08
..........................................uuggcaguauuguauaugaugc................................................  1       0  S08
..........................................................ugaugcaacuaauauugcacucgucccggccuaa....................  1       0  S08
...............................................................caacuaauauugcacucguccc...........................  1       0  S08
...............................................................caacuaauauugcacucgucccggccuaa....................  1       0  S08
................................................................aacuaauauugcacucgucccggccua.....................  1       0  S08
................................................................aacuaauauugcacucgucccggccuaa....................  1       0  S08
................................................................aacuaauauugcacucgucccggccuaac...................  1       0  S08
.................................................................acuaauauugcacucgucccgg.........................  2       0  S08
.................................................................acuaauauugcacucgucccggccuaa....................  1       0  S08
..................................................................cuaauauugcacucgucccggccuaa....................  1       0  S08
...................................................................uaauauugcacucgucccggccu......................  1       0  S08
...................................................................uaauauugcacucgucccggccua.....................  1       0  S08
...................................................................uaauauugcacucgucccggccuaa....................  2       0  S08
...................................................................uaauauugcacucgucccggccuaac...................  1       0  S08
....................................................................aauauugcacucgucccggccua.....................  1       0  S08
....................................................................aauauugcacucgucccggccuaa....................  9       0  S08
....................................................................aauauugcacucgucccggccuaac...................  2       0  S08
.....................................................................auauugcacucgucccggc........................  2       0  S08
.....................................................................auauugcacucgucccggccua.....................  8       0  S08
.....................................................................auauugcacucgucccggccuaa....................  29      0  S08
......................................................................uauugcacucgucccggc........................  58      0  S08
......................................................................uauugcacucgucccggcc.......................  271     0  S08
......................................................................uauugcacucgucccggccu......................  14098   0  S08
......................................................................uauugcacucgucccggccua.....................  71548   0  S08
......................................................................uauugcacucgucccggccuaa....................  261601  0  S08
......................................................................uauugcacucgucccggccuaac...................  3123    0  S08
......................................................................uauugcacucgucccggccuaaca..................  202     0  S08
......................................................................uauugcacucgucccggccuaacaa.................  11      0  S08
.......................................................................auugcacucgucccggccu......................  12      0  S08
.......................................................................auugcacucgucccggccua.....................  72      0  S08
.......................................................................auugcacucgucccggccuaa....................  226     0  S08
.......................................................................auugcacucgucccggccuaac...................  48      0  S08
.......................................................................auugcacucgucccggccuaaca..................  1       0  S08
........................................................................uugcacucgucccggccu......................  18      0  S08
........................................................................uugcacucgucccggccua.....................  111     0  S08
........................................................................uugcacucgucccggccuaa....................  232     0  S08
........................................................................uugcacucgucccggccuaac...................  14      0  S08
........................................................................uugcacucgucccggccuaaca..................  15      0  S08
.........................................................................ugcacucgucccggccua.....................  44      0  S08
.........................................................................ugcacucgucccggccuaa....................  51      0  S08
.........................................................................ugcacucgucccggccuaac...................  2       0  S08
..........................................................................gcacucgucccggccuaa....................  16      0  S08
............................................................................................caagggaggcgcaugcgccu  1       0  S08

....uuuggccugauaagccugcucuugcc..................................................................................  1       0  S03
.....uuggccugauaagccugcucuugcc..................................................................................  5       0  S03
......uggccugauaagccugcucuugcc..................................................................................  3       0  S03
........gccugauaagccugcucuugcc..................................................................................  8       0  S03
.........ccugauaagccugcucuugcc..................................................................................  66      0  S03
..........cugauaagccugcucuugcc..................................................................................  43      0  S03
...........ugauaagccugcucuugcc..................................................................................  74      0  S03
............gauaagccugcucuugcc..................................................................................  16      0  S03
...........................gccaggucggggugauuggcaguauugu.........................................................  1       0  S03
.............................caggucggggugauuggcaguau............................................................  1       0  S03
..............................aggucggggugauuggca................................................................  1       0  S03
..............................aggucggggugauuggcagu..............................................................  1       0  S03
..............................aggucggggugauuggcagua.............................................................  2       0  S03
..............................aggucggggugauuggcaguau............................................................  25      0  S03
..............................aggucggggugauuggcaguauu...........................................................  480     0  S03
..............................aggucggggugauuggcaguauuguauaugaugcaacu............................................  2       0  S03
..............................aggucggggugauuggcaguauuguauaugaugcaacua...........................................  3       0  S03
.................................ucggggugauuggcaguauu...........................................................  1       0  S03
.................................ucggggugauuggcaguauugu.........................................................  1       0  S03
..................................cggggugauuggcaguauugua........................................................  1       0  S03
```

Star **Mature**

```
auacuuuggccugauaagccugcucuugccaggucggggugauuggcaguauuguauaugaugcaacuaauauugcacucgucccggccuaacaagggaggcgcaugcgccu
.....................................ggugauuggcaguauuguauau.....................................................	1	0	S03
.....................................................guauaugaugcaacuaauauugcacucgucccggccuaa....................	1	0	S03
......................................................uauaugaugcaacuaauauugcacucgucccggccuaa....................	2	0	S03
.........................................................augaugcaacuaauauugcacucgucccggccua.....................	1	0	S03
...............................................................caacuaauauugcacucgucccggccuaa....................	1	0	S03
................................................................aacuaauauugcacucgucccggccuaa....................	1	0	S03
...................................................................uaauauugcacucgucccggccua.....................	1	0	S03
...................................................................uaauauugcacucgucccggccuaa....................	2	0	S03
....................................................................aauauugcacucgucccggccuaa....................	6	0	S03
....................................................................aauauugcacucgucccggccuaac...................	1	0	S03
.....................................................................auauugcacucgucccggccu......................	3	0	S03
.....................................................................auauugcacucgucccggccua.....................	18	0	S03
.....................................................................auauugcacucgucccggccuaa....................	76	0	S03
......................................................................uauugcacucgucccggc........................	100	0	S03
......................................................................uauugcacucgucccggcc.......................	373	0	S03
......................................................................uauugcacucgucccggccu......................	16670	0	S03
......................................................................uauugcacucgucccggccua.....................	38652	0	S03
......................................................................uauugcacucgucccggccuaa....................	180180	0	S03
......................................................................uauugcacucgucccggccuaac...................	1025	0	S03
......................................................................uauugcacucgucccggccuaaca..................	272	0	S03
......................................................................uauugcacucgucccggccuaacaa.................	13	0	S03
.......................................................................auugcacucgucccggcc.......................	1	0	S03
.......................................................................auugcacucgucccggccu......................	7	0	S03
.......................................................................auugcacucgucccggccua.....................	59	0	S03
.......................................................................auugcacucgucccggccuaa....................	282	0	S03
.......................................................................auugcacucgucccggccuaac...................	48	0	S03
.......................................................................auugcacucgucccggccuaaca..................	3	0	S03
........................................................................uugcacucgucccggccu......................	2	0	S03
........................................................................uugcacucgucccggccua.....................	8	0	S03
........................................................................uugcacucgucccggccuaa....................	29	0	S03
........................................................................uugcacucgucccggccuaac...................	6	0	S03
........................................................................uugcacucgucccggccuaaca..................	10	0	S03
.........................................................................ugcacucgucccggccuaa....................	3	0	S03
..........................................................................gcacucgucccggccuaa....................	6	0	S03
...........................................................................cacucgucccggccuaac...................	1	0	S03

..acuuuggccugauaagccugcucuugcc..................................................................................	3	0	S09
...cuuuggccugauaagccugcucuugcc..................................................................................	1	0	S09
....uuuggccugauaagccugcucuugcc..................................................................................	3	0	S09
.....uuggccugauaagccugcucuugcc..................................................................................	9	0	S09
......uggccugauaagccugcucuugcc..................................................................................	7	0	S09
.......ggccugauaagccugcucuugcc..................................................................................	1	0	S09
........gccugauaagccugcucuugcc..................................................................................	27	0	S09
.........ccugauaagccugcucuugcc..................................................................................	135	0	S09
..........cugauaagccugcucuugcc..................................................................................	143	0	S09
...........ugauaagccugcucuugcc..................................................................................	273	0	S09
............gauaagccugcucuugcc..................................................................................	63	0	S09
...........................gccaggucggggugauuggcag...............................................................	1	0	S09
.............................caggucggggugauuggcaguau............................................................	4	0	S09
.............................caggucggggugauuggcaguauu...........................................................	1	0	S09
..............................aggucggggugauuggca................................................................	1	0	S09
..............................aggucggggugauuggcag...............................................................	2	0	S09
..............................aggucggggugauuggcagu..............................................................	9	0	S09
..............................aggucggggugauuggcagua.............................................................	9	0	S09
..............................aggucggggugauuggcaguau............................................................	84	0	S09
..............................aggucggggugauuggcaguauu...........................................................	1076	0	S09
..............................aggucggggugauuggcaguauuguauaugaugcaac.............................................	2	0	S09
..............................aggucggggugauuggcaguauuguauaugaugcaacu............................................	1	0	S09
..............................aggucggggugauuggcaguauuguauaugaugcaacua...........................................	1	0	S09
..............................aggucggggugauuggcaguauuguauaugaugcaacuaa..........................................	1	0	S09
..............................aggucggggugauuggcaguauuguauaugaugcaacuaauauugcacucguc.............................	1	0	S09
..................................cggggugauuggcaguauu...........................................................	1	0	S09
...................................ggggugauuggcaguauuguaua......................................................	1	0	S09
................................................................aacuaauauugcacucgucccggccuaa....................	1	0	S09
...................................................................uaauauugcacucgucccggccuaa....................	1	0	S09
....................................................................aauauugcacucgucccggccuaa....................	1	0	S09
.....................................................................auauugcacucgucccggccua.....................	7	0	S09
.....................................................................auauugcacucgucccggccuaa....................	10	0	S09
......................................................................uauugcacucgucccggc........................	83	0	S09
......................................................................uauugcacucgucccggcc.......................	154	0	S09
```

```
                              Star                          Mature
auacuuuggccugauaagccugcucuugccaggucggggugauuggcaguauuguauaugaugcaacuaauauugcacucgucccggccuaacaagggaggcgcaugcgccu
......................................................................uauugcacucgucccggccu......................	5353	0	S09
......................................................................uauugcacucgucccggccua.....................	21299	0	S09
......................................................................uauugcacucgucccggccuaa....................	53529	0	S09
......................................................................uauugcacucgucccggccuaac...................	774	0	S09
......................................................................uauugcacucgucccggccuaaca..................	38	0	S09
......................................................................uauugcacucgucccggccuaacaa.................	2	0	S09
.......................................................................auugcacucgucccggccu......................	3	0	S09
.......................................................................auugcacucgucccggccua.....................	31	0	S09
.......................................................................auugcacucgucccggccuaa....................	62	0	S09
.......................................................................auugcacucgucccggccuaac...................	17	0	S09
........................................................................uugcacucgucccggccu......................	2	0	S09
........................................................................uugcacucgucccggccua.....................	11	0	S09
........................................................................uugcacucgucccggccuaa....................	13	0	S09
........................................................................uugcacucgucccggccuaac...................	3	0	S09
........................................................................uugcacucgucccggccuaaca..................	5	0	S09
.........................................................................ugcacucgucccggccua.....................	2	0	S09
.........................................................................ugcacucgucccggccuaa....................	4	0	S09
..........................................................................gcacucgucccggccuaa....................	1	0	S09
............................................................................................caagggaggcgcaugcgccu	2	0	S09

.........ccugauaagccugcucuugcc..................................................................................	17	0	S02
..........cugauaagccugcucuugcc..................................................................................	18	0	S02
...........ugauaagccugcucuugcc..................................................................................	19	0	S02
............gauaagccugcucuugcc..................................................................................	4	0	S02
..............................aggucggggugauuggcag...............................................................	1	0	S02
..............................aggucggggugauuggcagu..............................................................	2	0	S02
..............................aggucggggugauuggcaguau............................................................	15	0	S02
..............................aggucggggugauuggcaguauu...........................................................	174	0	S02
..............................aggucggggugauuggcaguauuguauaugaugc................................................	1	0	S02
..............................aggucggggugauuggcaguauuguauaugaugcaac.............................................	1	0	S02
..............................aggucggggugauuggcaguauuguauaugaugcaacu............................................	2	0	S02
..............................aggucggggugauuggcaguauuguauaugaugcaacua...........................................	2	0	S02
..............................aggucggggugauuggcaguauuguauaugaugcaacuaa..........................................	2	0	S02
.....................................................................auauugcacucgucccggccu......................	1	0	S02
.....................................................................auauugcacucgucccggccua.....................	3	0	S02
.....................................................................auauugcacucgucccggccuaa....................	15	0	S02
......................................................................uauugcacucgucccggc........................	20	0	S02
......................................................................uauugcacucgucccggcc.......................	201	0	S02
......................................................................uauugcacucgucccggccu......................	10559	0	S02
......................................................................uauugcacucgucccggccua.....................	11352	0	S02
......................................................................uauugcacucgucccggccuaa....................	59569	0	S02
......................................................................uauugcacucgucccggccuaac...................	204	0	S02
......................................................................uauugcacucgucccggccuaaca..................	150	0	S02
......................................................................uauugcacucgucccggccuaacaa.................	50	0	S02
.......................................................................auugcacucgucccggcc.......................	1	0	S02
.......................................................................auugcacucgucccggccu......................	5	0	S02
.......................................................................auugcacucgucccggccua.....................	24	0	S02
.......................................................................auugcacucgucccggccuaa....................	104	0	S02
.......................................................................auugcacucgucccggccuaac...................	9	0	S02
.......................................................................auugcacucgucccggccuaaca..................	1	0	S02
.......................................................................auugcacucgucccggccuaacaa.................	1	0	S02
........................................................................uugcacucgucccggccu......................	2	0	S02
........................................................................uugcacucgucccggccua.....................	1	0	S02
........................................................................uugcacucgucccggccuaa....................	8	0	S02
........................................................................uugcacucgucccggccuaac...................	1	0	S02
........................................................................uugcacucgucccggccuaaca..................	4	0	S02
.........................................................................ugcacucgucccggccua.....................	1	0	S02
.........................................................................ugcacucgucccggccuaa....................	2	0	S02
..........................................................................gcacucgucccggccuaa....................	2	0	S02

...cuuuggccugauaagccugcucuugcc..................................................................................	1	0	S04
.....uuggccugauaagccugcucuugcc..................................................................................	2	0	S04
......uggccugauaagccugcucuugcc..................................................................................	2	0	S04
........gccugauaagccugcucuugcc..................................................................................	8	0	S04
.........ccugauaagccugcucuugcc..................................................................................	58	0	S04
..........cugauaagccugcucuugcc..................................................................................	61	0	S04
...........ugauaagccugcucuugcc..................................................................................	128	0	S04
............gauaagccugcucuugcc..................................................................................	28	0	S04
.............................caggucggggugauuggcaguau............................................................	3	0	S04
..............................aggucggggugauuggcagu..............................................................	7	0	S04
```

```
                             Star                     Mature
auacuuuggccugauaagccugcucuugccaggucggggugauuggcaguauuguauaugaugcaacuaauauugcacucgucccggccuaacaagggaggcgcaugcgccu
..............................aggucggggugauuggcagua.............................................................  3       0  S04
..............................aggucggggugauuggcaguau............................................................  23      0  S04
..............................aggucggggugauuggcaguauu...........................................................  411     0  S04
..............................aggucggggugauuggcaguauuguauaugaugc................................................  1       0  S04
..............................aggucggggugauuggcaguauuguauaugaugca...............................................  2       0  S04
..............................aggucggggugauuggcaguauuguauaugaugcaacu............................................  1       0  S04
..............................aggucggggugauuggcaguauuguauaugaugcaacuaa..........................................  5       0  S04
.................................................................acuaauauugcacucgucccggccuaa....................  1       0  S04
...................................................................uaauauugcacucgucccggccuaa....................  2       0  S04
....................................................................aauauugcacucgucccggccuaa....................  1       0  S04
.....................................................................auauugcacucgucccggccu......................  4       0  S04
.....................................................................auauugcacucgucccggccua.....................  10      0  S04
.....................................................................auauugcacucgucccggccuaa....................  44      0  S04
.....................................................................auauugcacucgucccggccuaac...................  1       0  S04
......................................................................uauugcacucgucccggc........................  150     0  S04
......................................................................uauugcacucgucccggcc.......................  421     0  S04
......................................................................uauugcacucgucccggccu......................  15760   0  S04
......................................................................uauugcacucgucccggccua.....................  37100   0  S04
......................................................................uauugcacucgucccggccuaa....................  140075  0  S04
......................................................................uauugcacucgucccggccuaac...................  848     0  S04
......................................................................uauugcacucgucccggccuaaca..................  188     0  S04
......................................................................uauugcacucgucccggccuaacaa.................  12      0  S04
.......................................................................auugcacucgucccggcc.......................  2       0  S04
.......................................................................auugcacucgucccggccu......................  2       0  S04
.......................................................................auugcacucgucccggccua.....................  49      0  S04
.......................................................................auugcacucgucccggccuaa....................  212     0  S04
.......................................................................auugcacucgucccggccuaac...................  22      0  S04
.......................................................................auugcacucgucccggccuaaca..................  4       0  S04
........................................................................uugcacucgucccggccu......................  1       0  S04
........................................................................uugcacucgucccggccua.....................  5       0  S04
........................................................................uugcacucgucccggccuaa....................  34      0  S04
........................................................................uugcacucgucccggccuaac...................  3       0  S04
........................................................................uugcacucgucccggccuaaca..................  8       0  S04
.........................................................................ugcacucgucccggccuaa....................  5       0  S04
..........................................................................gcacucgucccggccuaa....................  2       0  S04

.uacuuuggccugauaagccugcucuugcc..................................................................................  1       0  S10
..acuuuggccugauaagccugcucuugcc..................................................................................  4       0  S10
....uuuggccugauaagccugcucuug....................................................................................  1       0  S10
....uuuggccugauaagccugcucuugcc..................................................................................  2       0  S10
.....uuggccugauaagccugcucuugcc..................................................................................  4       0  S10
......uggccugauaagccugcucuugcc..................................................................................  12      0  S10
........gccugauaagccugcucuugcc..................................................................................  31      0  S10
.........ccugauaagccugcucuugcc..................................................................................  110     0  S10
..........cugauaagccugcucuugcc..................................................................................  137     0  S10
...........ugauaagccugcucuugcc..................................................................................  271     0  S10
............gauaagccugcucuugcc..................................................................................  51      0  S10
.........................uugccaggucggggugauugg..................................................................  1       0  S10
............................ccaggucggggugauuggcagu..............................................................  1       0  S10
.............................caggucggggugauuggcaguau............................................................  1       0  S10
..............................aggucggggugauuggca................................................................  3       0  S10
..............................aggucggggugauuggcag...............................................................  3       0  S10
..............................aggucggggugauuggcagu..............................................................  23      0  S10
..............................aggucggggugauuggcagua.............................................................  17      0  S10
..............................aggucggggugauuggcaguau............................................................  171     0  S10
..............................aggucggggugauuggcaguauu...........................................................  1596    0  S10
..............................aggucggggugauuggcaguauuguauaugaugcaacua.............................................  1       0  S10
..............................aggucggggugauuggcaguauuguauaugaugcaacuaa..........................................  1       0  S10
...............................ggucggggugauuggcaguauu...........................................................  1       0  S10
.................................ucggggugauuggcaguauu...........................................................  1       0  S10
..........................................uuggcaguauuguauaugaugc................................................  1       0  S10
................................................................aacuaauauugcacucgucccggccua.....................  1       0  S10
...................................................................uaauauugcacucgucccggccua.....................  2       0  S10
...................................................................uaauauugcacucgucccggccuaac...................  1       0  S10
....................................................................aauauugcacucgucccggccua.....................  1       0  S10
....................................................................aauauugcacucgucccggccuaa....................  2       0  S10
....................................................................aauauugcacucgucccggccuaac...................  1       0  S10
.....................................................................auauugcacucgucccggccu......................  1       0  S10
.....................................................................auauugcacucgucccggccua.....................  4       0  S10
.....................................................................auauugcacucgucccggccuaa....................  18      0  S10
```

```
                              Star                                    Mature
auacuuuggccugauaagccugcucuugccaggucggggugauuggcaguauuguauaugaugcaacuaauauugcacucgucccggccuaacaagggaggcgcaugcgccu
......................................................................uauugcacucgucccggc........................  107     0  S10
......................................................................uauugcacucgucccggcc.......................  202     0  S10
......................................................................uauugcacucgucccggccu......................  7235    0  S10
......................................................................uauugcacucgucccggccua.....................  31689   0  S10
......................................................................uauugcacucgucccggccuaa....................  71435   0  S10
......................................................................uauugcacucgucccggccuaac...................  932     0  S10
......................................................................uauugcacucgucccggccuaaca..................  40      0  S10
......................................................................uauugcacucgucccggccuaacaa.................  6       0  S10
.......................................................................auugcacucgucccggccu......................  8       0  S10
.......................................................................auugcacucgucccggccua.....................  45      0  S10
.......................................................................auugcacucgucccggccuaa....................  86      0  S10
.......................................................................auugcacucgucccggccuaac...................  26      0  S10
........................................................................uugcacucgucccggccu......................  1       0  S10
........................................................................uugcacucgucccggccua.....................  9       0  S10
........................................................................uugcacucgucccggccuaa....................  12      0  S10
........................................................................uugcacucgucccggccuaac...................  3       0  S10
........................................................................uugcacucgucccggccuaaca..................  7       0  S10
.........................................................................ugcacucgucccggccua.....................  1       0  S10
..........................................................................gcacucgucccggccuaa....................  3       0  S10
............................................................................................caagggaggcgcaugcgc..  1       0  S10
............................................................................................caagggaggcgcaugcgcc.  5       0  S10
............................................................................................caagggaggcgcaugcgccu  3       0  S10

..acuuuggccugauaagccugcucuugcc..................................................................................  2       0  S05
...cuuuggccugauaagccugcucuugcc..................................................................................  1       0  S05
....uuuggccugauaagccugcucuugcc..................................................................................  1       0  S05
.....uuggccugauaagccugcucuugcc..................................................................................  1       0  S05
......uggccugauaagccugcucuugcc..................................................................................  2       0  S05
........gccugauaagccugcucuugcc..................................................................................  5       0  S05
.........ccugauaagccugcucuugcc..................................................................................  58      0  S05
..........cugauaagccugcucuugcc..................................................................................  56      0  S05
...........ugauaagccugcucuugcc..................................................................................  95      0  S05
............gauaagccugcucuugcc..................................................................................  25      0  S05
............................ccaggucggggugauuggcagu..............................................................  1       0  S05
.............................caggucggggugauuggcaguau............................................................  1       0  S05
.............................caggucggggugauuggcaguauu...........................................................  1       0  S05
..............................aggucggggugauuggcag...............................................................  1       0  S05
..............................aggucggggugauuggcagu..............................................................  8       0  S05
..............................aggucggggugauuggcaguau............................................................  28      0  S05
..............................aggucggggugauuggcaguauu...........................................................  482     0  S05
..............................aggucggggugauuggcaguauug..........................................................  2       0  S05
..............................aggucggggugauuggcaguauugua........................................................  1       0  S05
..............................aggucggggugauuggcaguauuguauaugaugcaacu............................................  1       0  S05
................................gucggggugauuggcaguauu...........................................................  1       0  S05
.................................ucggggugauuggcaguauugu.........................................................  1       0  S05
..........................................................ugaugcaacuaauauugcacucgucccggcc.......................  1       0  S05
..........................................................ugaugcaacuaauauugcacucgucccggccuaa....................  1       0  S05
..............................................................gcaacuaauauugcacucgucccggccuaac...................  1       0  S05
...............................................................caacuaauauugcacucg...............................  1       0  S05
................................................................aacuaauauugcacucgucccggccu......................  1       0  S05
.................................................................acuaauauugcacucgucccggccuaa....................  1       0  S05
..................................................................cuaauauugcacucgucccggc........................  1       0  S05
...................................................................uaauauugcacucgucccggccu......................  1       0  S05
...................................................................uaauauugcacucgucccggccua.....................  3       0  S05
...................................................................uaauauugcacucgucccggccuaa....................  5       0  S05
....................................................................aauauugcacucgucccggcc.......................  1       0  S05
....................................................................aauauugcacucgucccggccu......................  1       0  S05
....................................................................aauauugcacucgucccggccua.....................  4       0  S05
....................................................................aauauugcacucgucccggccuaa....................  24      0  S05
....................................................................aauauugcacucgucccggccuaac...................  1       0  S05
.....................................................................auauugcacucgucccggcc.......................  1       0  S05
.....................................................................auauugcacucgucccggccu......................  2       0  S05
.....................................................................auauugcacucgucccggccua.....................  18      0  S05
.....................................................................auauugcacucgucccggccuaa....................  57      0  S05
.....................................................................auauugcacucgucccggccuaac...................  3       0  S05
......................................................................uauugcacucgucccggc........................  145     0  S05
......................................................................uauugcacucgucccggcc.......................  343     0  S05
......................................................................uauugcacucgucccggccu......................  14106   0  S05
......................................................................uauugcacucgucccggccua.....................  39247   0  S05
......................................................................uauugcacucgucccggccuaa....................  163497  0  S05
```

Star Mature

auacuuuggccugauaaagccugcucuugccaggucggggugauuggcaguauuguauaugaugcaacuaauauugcacucgucccggccuaacaagggaggcgcaugcgccu			
....uauugcacucgucccggccuaac...................	1197	0	S05
....uauugcacucgucccggccuaaca..................	198	0	S05
....uauugcacucgucccggccuaacaa................	10	0	S05
....auugcacucgucccggccu......................	9	0	S05
....auugcacucgucccggccua.....................	74	0	S05
....auugcacucgucccggccuaa....................	279	0	S05
....auugcacucgucccggccuaac...................	58	0	S05
....auugcacucgucccggccuaaca..................	4	0	S05
....uugcacucgucccggccu......................	5	0	S05
....uugcacucgucccggccua.....................	13	0	S05
....uugcacucgucccggccuaa....................	52	0	S05
....uugcacucgucccggccuaac...................	10	0	S05
....uugcacucgucccggccuaaca..................	9	0	S05
....uugcacucgucccggccuaacaa.................	1	0	S05
....ugcacucgucccggccua.....................	8	0	S05
....ugcacucgucccggccuaa....................	10	0	S05
....gcacucgucccggccuaa....................	5	0	S05
auacuuuggccugauaaagccugcucuugcc..	2	0	S07
..acuuuggccugauaaagccugcucuugcc..	3	0	S07
...cuuuggccugauaaagccugcucuugcc..	3	0	S07
....uuuggccugauaaagccugcucuugcc..	1	0	S07
.....uuggccugauaaagccugcucuugcc..	7	0	S07
........gccugauaaagccugcucuugcc..	32	0	S07
.........ccugauaaagccugcucuugcc..	176	0	S07
..........cugauaaagccugcucuugcc..	179	0	S07
...........ugauaaagccugcucuugc...	1	0	S07
...........ugauaaagccugcucuugcc..	278	0	S07
............gauaaagccugcucuugcc..	47	0	S07
..............................caggucggggugauuggcaguau..	2	0	S07
..............................caggucggggugauuggcaguauu...	1	0	S07
...............................aggucggggugauuggca..	3	0	S07
...............................aggucggggugauuggcag...	4	0	S07
...............................aggucggggugauuggcagu..	42	0	S07
...............................aggucggggugauuggcagua...	18	0	S07
...............................aggucggggugauuggcaguau..	262	0	S07
...............................aggucggggugauuggcaguauu...	2231	0	S07
...............................aggucggggugauuggcaguauuguauaugaugc..	3	0	S07
...............................aggucggggugauuggcaguauuguauaugaugca...	2	0	S07
...............................aggucggggugauuggcaguauuguauaugaugcaac...	2	0	S07
...............................aggucggggugauuggcaguauuguauaugaugcaacu..	1	0	S07
...............................aggucggggugauuggcaguauuguauaugaugcaacua...	1	0	S07
...............................aggucggggugauuggcaguauuguauaugaugcaacuaa..	8	0	S07
...............................aggucggggugauuggcaguauuguauaugaugcaacuaau...	1	0	S07
...............................aggucggggugauuggcaguauuguauaugaugcaacuaauauugcacucgu..............................	1	0	S07
...............................aggucggggugauuggcaguauuguauaugaugcaacuaauauugcacucguc.............................	1	0	S07
..................................ucggggugauuggcaguauu...	3	0	S07
...................................cggggugauuggcaguauu...	1	0	S07
...................................cggggugauuggcaguauuguauaugaugcaa..	1	0	S07
....ugauuggcaguauuguauaugaugcaacuaauauugcacucgucccggccua.....................	1	0	S07
....guauaugaugcaacuaauauugcacucgucccggccuaa....................	2	0	S07
....aacuaauauugcacucgucccggccua.....................	1	0	S07
....aacuaauauugcacucgucccggccuaa....................	1	0	S07
....uaauauugcacucgucccggcc.......................	1	0	S07
....aauauugcacucgucccggcc.......................	1	0	S07
....aauauugcacucgucccggccu......................	1	0	S07
....aauauugcacucgucccggccuaa....................	8	0	S07
....aauauugcacucgucccggccuaac...................	1	0	S07
....auauugcacucgucccggccu......................	8	0	S07
....auauugcacucgucccggccua.....................	15	0	S07
....auauugcacucgucccggccuaa....................	46	0	S07
....auauugcacucgucccggccuaac...................	2	0	S07
....uauugcacucgucccggc........................	109	0	S07
....uauugcacucgucccggcc.......................	256	0	S07
....uauugcacucgucccggccu......................	16391	0	S07
....uauugcacucgucccggccua.....................	54170	0	S07
....uauugcacucgucccggccuaa....................	181729	0	S07
....uauugcacucgucccggccuaac...................	3339	0	S07
....uauugcacucgucccggccuaaca..................	187	0	S07
....uauugcacucgucccggccuaacaa................	11	0	S07

Star / Mature			
auacuuuggccugauaagccugcucuugccaggucggggugauuggcaguauuguauaugaugcaacuaauauugcacucgucccggccuaacaagggaggcgcaugcgccu			
....auugcacucgucccggccu......................	11	0	S07
....auugcacucgucccggccua.....................	55	0	S07
....auugcacucgucccggccuaa....................	160	0	S07
....auugcacucgucccggccuaac...................	57	0	S07
....uugcacucgucccggccu......................	1	0	S07
....uugcacucgucccggccua.....................	13	0	S07
....uugcacucgucccggccuaa....................	38	0	S07
....uugcacucgucccggccuaac...................	28	0	S07
....uugcacucgucccggccuaaca..................	11	0	S07
....uugcacucgucccggccuaacaa.................	1	0	S07
....ugcacucgucccggccua.....................	4	0	S07
....ugcacucgucccggccuaa....................	3	0	S07
....gcacucgucccggccuaa....................	3	0	S07
....caagggaggcgcaugcgcc.	4	0	S07
....caagggaggcgcaugcgccu	3	0	S07
..........cugauaagccugcucuugcc..	1	0	S01
...........ugauaagccugcucuugcc..	2	0	S01
..............................aggucggggugauuggcaguauu...	1	0	S01
....auauugcacucgucccggccu......................	1	0	S01
....auauugcacucgucccggccua.....................	2	0	S01
....auauugcacucgucccggccuaa....................	10	0	S01
....uauugcacucgucccggc........................	19	0	S01
....uauugcacucgucccggcc.......................	202	0	S01
....uauugcacucgucccggccu......................	9744	0	S01
....uauugcacucgucccggccua.....................	6994	0	S01
....uauugcacucgucccggccuaa....................	30705	0	S01
....uauugcacucgucccggccuaac...................	80	0	S01
....uauugcacucgucccggccuaaca..................	39	0	S01
....uauugcacucgucccggccuaacaa.................	57	0	S01
....auugcacucgucccggccu......................	1	0	S01
....auugcacucgucccggccua.....................	11	0	S01
....auugcacucgucccggccuaa....................	52	0	S01
....auugcacucgucccggccuaac...................	3	0	S01
....auugcacucgucccggccuaacaa.................	1	0	S01
....uugcacucgucccggccu......................	1	0	S01
....uugcacucgucccggccuaa....................	9	0	S01
....ugcacucgucccggccua.....................	2	0	S01
....gcacucgucccggccuaa....................	3	0	S01
..acuuuggccugauaagccugcucuugcc..	1	0	S06
.....uuggccugauaagccugcucuugcc..	3	0	S06
........gccugauaagccugcucuugcc..	1	0	S06
.........ccugauaagccugcucuugcc..	58	0	S06
..........cugauaagccugcucuugcc..	70	0	S06
...........ugauaagccugcucuugcc..	108	0	S06
............gauaagccugcucuugcc..	21	0	S06
..............................aggucggggugauuggca..	6	0	S06
..............................aggucggggugauuggcag...	6	0	S06
..............................aggucggggugauuggcagu..	17	0	S06
..............................aggucggggugauuggcagua...	13	0	S06
..............................aggucggggugauuggcaguau..	58	0	S06
..............................aggucggggugauuggcaguauu...	777	0	S06
..............................aggucggggugauuggcaguauug..	1	0	S06
..............................aggucggggugauuggcaguauugua..	4	0	S06
..............................aggucggggugauuggcaguauuguaua..	1	0	S06
..............................aggucggggugauuggcaguauuguauau...	1	0	S06
..............................aggucggggugauuggcaguauuguauaugaugca...	1	0	S06
..............................aggucggggugauuggcaguauuguauaugaugcaacu..	3	0	S06
..............................aggucggggugauuggcaguauuguauaugaugcaacua...	1	0	S06
..............................aggucggggugauuggcaguauuguauaugaugcaacuaa..	1	0	S06
..............................aggucggggugauuggcaguauuguauaugaugcaacuaau...	1	0	S06
..............................aggucggggugauuggcaguauuguauaugaugcaacuaauauugc....................................	1	0	S06
.................................ucggggugauuggcaguauugu...	1	0	S06
..................................cggggugauuggcaguauuguauaugaugcaacuaaua..	1	0	S06
....uauaugaugcaacuaauauugcacucgucccggccuaa....................	1	0	S06
....gaugcaacuaauauugcacucgucccggccuaa....................	1	0	S06
....aacuaauauugcacucgucccggccuaa....................	2	0	S06
....acuaauauugcacucgucccggccuaa....................	2	0	S06
....cuaauauugcacucgucccggccua.....................	1	0	S06

**Star** / **Mature**			
auacuuuggccugauaagccugcucuugccaggucggggugauuggcaguauuguauaugaugcaacuaauauugcacucgucccggccuaacaagggaggcgcaugcgccu			
....uaauauugcacucgucccgg.........................	1	0	S06
....uaauauugcacucgucccggcc.......................	2	0	S06
....uaauauugcacucgucccggccua.....................	1	0	S06
....uaauauugcacucgucccggccuaa....................	4	0	S06
....aauauugcacucgucccggccu......................	6	0	S06
....aauauugcacucgucccggccua.....................	1	0	S06
....aauauugcacucgucccggccuaa....................	17	0	S06
....aauauugcacucgucccggccuaac...................	1	0	S06
....auauugcacucgucccggccu......................	7	0	S06
....auauugcacucgucccggccua.....................	20	0	S06
....auauugcacucgucccggccuaa....................	71	0	S06
....uauugcacucgucccggc........................	84	0	S06
....uauugcacucgucccggcc.......................	403	0	S06
....uauugcacucgucccggccu......................	17205	0	S06
....uauugcacucgucccggccua.....................	55660	0	S06
....uauugcacucgucccggccuaa....................	243088	0	S06
....uauugcacucgucccggccuaac...................	2093	0	S06
....uauugcacucgucccggccuaaca..................	158	0	S06
....uauugcacucgucccggccuaacaa................	10	0	S06
....auugcacucgucccggccu......................	6	0	S06
....auugcacucgucccggccua.....................	67	0	S06
....auugcacucgucccggccuaa....................	309	0	S06
....auugcacucgucccggccuaac...................	48	0	S06
....auugcacucgucccggccuaaca..................	5	0	S06
....auugcacucgucccggccuaacaa................	1	0	S06
....uugcacucgucccggccu......................	4	0	S06
....uugcacucgucccggccua.....................	14	0	S06
....uugcacucgucccggccuaa....................	73	0	S06
....uugcacucgucccggccuaac...................	8	0	S06
....uugcacucgucccggccuaaca..................	9	0	S06
....ugcacucgucccggccua.....................	7	0	S06
....ugcacucgucccggccuaa....................	17	0	S06
....gcacucgucccggccuaa....................	6	0	S06

Provisional ID : Scaffold76_2597
Score total : 374137.1
Score for star read(s) : 3.9
Score for read counts : 374129.7
Score for mfe : 2
Score for randfold : 1.6
Score for cons. seed :
Total read count : 733851
Mature read count : 731517
Loop read count : 0
Star read count : 2334

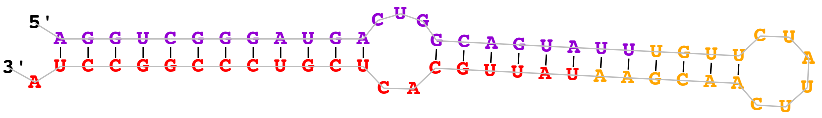

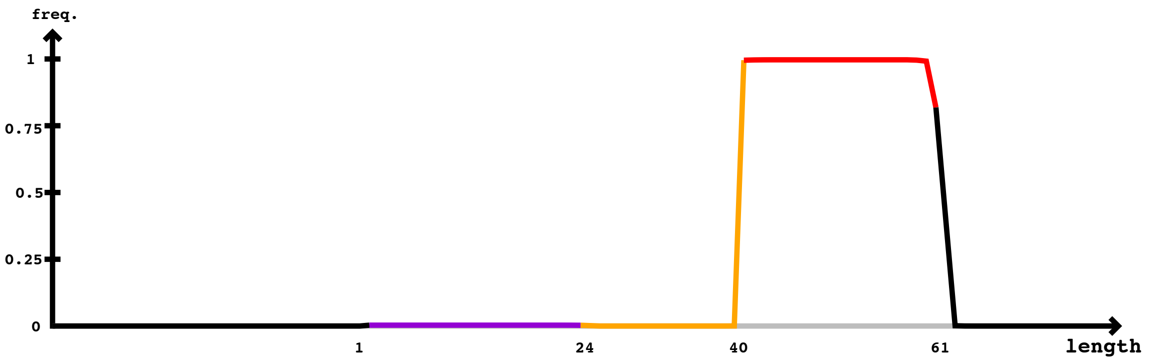

Star Mature

```
5'- uuuuuuuuuuuuuuucuauuuuacucuggccaggucgggaugacuggcaguauuuguucuauucaacgaauauugcacucgucccggccuaucaagagugacucuugca  -3'  obs
    uuuuuuuuuuuuuuucuauuuuacucuggccaggucgggaugacuggcaguauuuguucuauucaacgaauauugcacucgucccggccuaucaagagugacucuugca       exp
    ....................(((((((....((((((((((((...((((((((((((......))))))))))))..))))))))))))....))))))).........   reads  mm  sample
    ...............................aggucgggaugacuggcaguau........................................................   1      0   S05
    ...............................aggucgggaugacuggcaguauu.......................................................   9      0   S05
    ....................................................................aauauugcacucgucccggcc....................   1      0   S05
    ....................................................................aauauugcacucgucccggccu...................   1      0   S05
    ....................................................................aauauugcacucgucccggccua..................   4      0   S05
    ....................................................................aauauugcacucgucccggccuau.................   1      0   S05
    .....................................................................auauugcacucgucccggcc....................   1      0   S05
    .....................................................................auauugcacucgucccggccu...................   2      0   S05
    .....................................................................auauugcacucgucccggccua..................   18     0   S05
    .....................................................................auauugcacucgucccggccuau.................   1      0   S05
    ......................................................................uauugcacucgucccggc.....................   145    0   S05
    ......................................................................uauugcacucgucccggcc....................   343    0   S05
    ......................................................................uauugcacucgucccggccu...................   14106  0   S05
    ......................................................................uauugcacucgucccggccua..................   39247  0   S05
    ......................................................................uauugcacucgucccggccuau.................   7188   0   S05
    ......................................................................uauugcacucgucccggccuauc................   16     0   S05
    ......................................................................uauugcacucgucccggccuauca...............   8      0   S05
    .......................................................................augcacucgucccggccu...................   9      0   S05
    .......................................................................augcacucgucccggccua..................   74     0   S05
    .......................................................................augcacucgucccggccuau.................   10     0   S05
    .......................................................................augcacucgucccggccuauc................   1      0   S05
    ........................................................................uugcacucgucccggccu...................   5      0   S05
    ........................................................................uugcacucgucccggccua..................   13     0   S05
    ........................................................................uugcacucgucccggccuau.................   3      0   S05
    .........................................................................ugcacucgucccggccua..................   8      0   S05
    .........................................................................ugcacucgucccggccuau.................   1      0   S05
    ..........................................................................gcacucgucccggccuau.................   1      0   S05

    .uuuuuuuuuuuCuucuauuu........................................................................................   1      1   S10
    ......................acucuggccaggucgggaugac.................................................................   1      0   S10
    ...............................aggucgggaugacuggca............................................................   2      0   S10
    ...............................aggucgggaugacuggcagu..........................................................   7      0   S10
    ...............................aggucgggaugacuggcagua.........................................................   11     0   S10
```

Star Mature

uuuuuuuuuuuuuuucuauuuuacucuggccaggucgggaugacuggcaguauuuguucuauucaacgaauauugcacucgucccggccuaucaagagugacucuugca			
...............................aggucgggaugacuggcaguau..	62	0	S10
...............................aggucgggaugacuggcaguauu...	649	0	S10
...............................aggucgggaugacuggcaguauuu..	17	0	S10
....aauauugcacucgucccggccua..................	1	0	S10
....aauauugcacucgucccggccuau.................	2	0	S10
....auauugcacucgucccggccu...................	1	0	S10
....auauugcacucgucccggccua..................	4	0	S10
....uauugcacucgucccggc.....................	107	0	S10
....uauugcacucgucccggcc....................	202	0	S10
....uauugcacucgucccggccu...................	7235	0	S10
....uauugcacucgucccggccua..................	31689	0	S10
....uauugcacucgucccggccuau.................	43371	0	S10
....uauugcacucgucccggccuauc................	93	0	S10
....uauugcacucgucccggccuauca...............	9	0	S10
....auugcacucgucccggccu...................	8	0	S10
....auugcacucgucccggccua..................	45	0	S10
....auugcacucgucccggccuau.................	11	0	S10
....auugcacucgucccggccuauc................	1	0	S10
....uugcacucgucccggccu...................	1	0	S10
....uugcacucgucccggccua..................	9	0	S10
....uugcacucgucccggccuau.................	8	0	S10
....ugcacucgucccggccua..................	1	0	S10
....ugcacucgucccggccuau.................	1	0	S10
....gcacucgucccggccuau.................	2	0	S10
....auauugcacucgucccggccu...................	1	0	S01
....auauugcacucgucccggccua..................	2	0	S01
....auauugcacucgucccggccuau.................	1	0	S01
....uauugcacucgucccggc.....................	19	0	S01
....uauugcacucgucccggcc....................	202	0	S01
....uauugcacucgucccggccu...................	9744	0	S01
....uauugcacucgucccggccua..................	6994	0	S01
....uauugcacucgucccggccuau.................	689	0	S01
....uauugcacucgucccggccuauca...............	6	0	S01
....uauugcacucgucccggccuaucaa..............	3	0	S01
....auugcacucgucccggccu...................	1	0	S01
....auugcacucgucccggccua..................	11	0	S01
....uugcacucgucccggccu...................	1	0	S01
....ugcacucgucccggccua..................	2	0	S01
...............................aggucgggaugacuggca..	1	0	S06
...............................aggucgggaugacuggcagu..	1	0	S06
...............................aggucgggaugacuggcaguau..	10	0	S06
...............................aggucgggaugacuggcaguauu...	28	0	S06
....aauauugcacucgucccggccu...................	6	0	S06
....aauauugcacucgucccggccua..................	1	0	S06
....aauauugcacucgucccggccuau.................	3	0	S06
....auauugcacucgucccggccu...................	7	0	S06
....auauugcacucgucccggccua..................	20	0	S06
....auauugcacucgucccggccuau.................	3	0	S06
....uauugcacucgucccggc.....................	84	0	S06
....uauugcacucgucccggcc....................	403	0	S06
....uauugcacucgucccggccu...................	17205	0	S06
....uauugcacucgucccggccua..................	55660	0	S06
....uauugcacucgucccggccuau.................	24770	0	S06
....uauugcacucgucccggccuauc................	60	0	S06
....uauugcacucgucccggccuauca...............	11	0	S06
....uauugcacucgucccggccuaucaa..............	1	0	S06
....auugcacucgucccggccu...................	6	0	S06
....auugcacucgucccggccua..................	67	0	S06
....auugcacucgucccggccuau.................	29	0	S06
....uugcacucgucccggccu...................	4	0	S06
....uugcacucgucccggccua..................	14	0	S06
....uugcacucgucccggccuau.................	9	0	S06
....ugcacucgucccggccua..................	7	0	S06
....ugcacucgucccggccuau.................	2	0	S06
....gcacucgucccggccuau.................	1	0	S06
...............................aggucgggaugacuggca..	1	0	S07
...............................aggucgggaugacuggcagu..	3	0	S07

```
                              Star                                    Mature
uuuuuuuuuuuuuuucuauuuuacucuggccaggucgggaugacuggcaguauuuguucuauucaacgaauauugcacucgucccggccuaucaagagugacucuugca
...............................aggucgggaugacuggcagua.........................................................   6       0   S07
...............................aggucgggaugacuggcaguau........................................................   27      0   S07
...............................aggucgggaugacuggcaguauu.......................................................   342     0   S07
...............................aggucgggaugacuggcaguauuu......................................................   6       0   S07
....................................................................aauauugcacucgucccggcc....................   1       0   S07
....................................................................aauauugcacucgucccggccu...................   1       0   S07
.....................................................................auauugcacucgucccggccu...................   8       0   S07
.....................................................................auauugcacucgucccggccua..................   15      0   S07
.....................................................................auauugcacucgucccggccuau.................   4       0   S07
......................................................................uauugcacucgucccggc.....................   109     0   S07
......................................................................uauugcacucgucccggcc....................   256     0   S07
......................................................................uauugcacucgucccggccu...................   16391   0   S07
......................................................................uauugcacucgucccggccua..................   54170   0   S07
......................................................................uauugcacucgucccggccuau.................   47912   0   S07
......................................................................uauugcacucgucccggccuauc................   140     0   S07
......................................................................uauugcacucgucccggccuauca...............   14      0   S07
......................................................................uauugcacucgucccggccuaucaa..............   2       0   S07
.......................................................................auugcacucgucccggccu...................   11      0   S07
.......................................................................auugcacucgucccggccua..................   55      0   S07
.......................................................................auugcacucgucccggccuau.................   21      0   S07
........................................................................uugcacucgucccggccu...................   1       0   S07
........................................................................uugcacucgucccggccua..................   13      0   S07
........................................................................uugcacucgucccggccuau.................   7       0   S07
.........................................................................ugcacucgucccggccua..................   4       0   S07
.........................................................................ugcacucgucccggccuau.................   1       0   S07

...............................aggucgggaugacuggcaguauu.......................................................   3       0   S03
...............................aggucgggaugacuggcaguauuu......................................................   1       0   S03
.....................................................................auauugcacucgucccggccu...................   3       0   S03
.....................................................................auauugcacucgucccggccua..................   18      0   S03
.....................................................................auauugcacucgucccggccuau.................   2       0   S03
......................................................................uauugcacucgucccggc.....................   100     0   S03
......................................................................uauugcacucgucccggcc....................   373     0   S03
......................................................................uauugcacucgucccggccu...................   16670   0   S03
......................................................................uauugcacucgucccggccua..................   38652   0   S03
......................................................................uauugcacucgucccggccuau.................   5711    0   S03
......................................................................uauugcacucgucccggccuauc................   9       0   S03
......................................................................uauugcacucgucccggccuauca...............   5       0   S03
.......................................................................auugcacucgucccggcc....................   1       0   S03
.......................................................................auugcacucgucccggccu...................   7       0   S03
.......................................................................auugcacucgucccggccua..................   59      0   S03
.......................................................................auugcacucgucccggccuau.................   14      0   S03
........................................................................uugcacucgucccggccu...................   2       0   S03
........................................................................uugcacucgucccggccua..................   8       0   S03
........................................................................uugcacucgucccggccuau.................   1       0   S03

...............................aggucgggaugacuggcagua.........................................................   15      0   S09
...............................aggucgggaugacuggcaguau........................................................   59      0   S09
...............................aggucgggaugacuggcaguauu.......................................................   602     0   S09
...............................aggucgggaugacuggcaguauuu......................................................   16      0   S09
.....................................................................auauugcacucgucccggccua..................   7       0   S09
......................................................................uauugcacucgucccggc.....................   83      0   S09
......................................................................uauugcacucgucccggcc....................   154     0   S09
......................................................................uauugcacucgucccggccu...................   5353    0   S09
......................................................................uauugcacucgucccggccua..................   21299   0   S09
......................................................................uauugcacucgucccggccuau.................   28388   0   S09
......................................................................uauugcacucgucccggccuauc................   81      0   S09
......................................................................uauugcacucgucccggccuauca...............   3       0   S09
.......................................................................auugcacucgucccggccu...................   3       0   S09
.......................................................................auugcacucgucccggccua..................   31      0   S09
.......................................................................auugcacucgucccggccuau.................   9       0   S09
........................................................................uugcacucgucccggccu...................   2       0   S09
........................................................................uugcacucgucccggccua..................   11      0   S09
........................................................................uugcacucgucccggccuau.................   2       0   S09
.........................................................................ugcacucgucccggccua..................   2       0   S09
.........................................................................ugcacucgucccggccuau.................   1       0   S09
..........................................................................gcacucgucccggccuau.................   1       0   S09

...............................aggucgggaugacuggca............................................................   1       0   S08
...............................aggucgggaugacuggcagu..........................................................   6       0   S08
```

Star Mature

uuuuuuuuuuuuuuucuauuuuacucuggccaggucgggaugacuggcaguauuuguucuauucaacgaauauugcacucgucccggccuaucaagagugacucuugca			
...............................aggucgggaugacuggcagua...	5	0	S08
...............................aggucgggaugacuggcaguau..	48	0	S08
...............................aggucgggaugacuggcaguauu...	384	0	S08
...............................aggucgggaugacuggcaguauuu..	4	0	S08
....aauauugcacucgucccggccua..................	1	0	S08
....aauauugcacucgucccggccuau.................	2	0	S08
....auauugcacucgucccggc.....................	2	0	S08
....auauugcacucgucccggccua..................	8	0	S08
....uauugcacucgucccggc.....................	58	0	S08
....uauugcacucgucccggcc....................	271	0	S08
....uauugcacucgucccggccu...................	14098	0	S08
....uauugcacucgucccggccua..................	71548	0	S08
....uauugcacucgucccggccuau.................	66154	0	S08
....uauugcacucgucccggccuauc................	210	0	S08
....uauugcacucgucccggccuauca...............	22	0	S08
....uauugcacucgucccggccuaucaa..............	1	0	S08
....auugcacucgucccggccu...................	12	0	S08
....auugcacucgucccggccua..................	72	0	S08
....auugcacucgucccggccuau.................	39	0	S08
....auugcacucgucccggccuauc................	2	0	S08
....uugcacucgucccggccu...................	18	0	S08
....uugcacucgucccggccua..................	111	0	S08
....uugcacucgucccggccuau.................	50	0	S08
....uugcacucgucccggccuauc................	1	0	S08
....uugcacucgucccggccuauca...............	4	0	S08
....ugcacucgucccggccua..................	44	0	S08
....ugcacucgucccggccuau.................	6	0	S08
....gcacucgucccggccuau.................	6	0	S08
...............................aggucgggaugacuggcaguauu...	7	0	S04
....auauugcacucgucccggccu...................	4	0	S04
....auauugcacucgucccggccua..................	10	0	S04
....auauugcacucgucccggccuau.................	4	0	S04
....uauugcacucgucccggc.....................	150	0	S04
....uauugcacucgucccggcc....................	421	0	S04
....uauugcacucgucccggccu...................	15760	0	S04
....uauugcacucgucccggccua..................	37100	0	S04
....uauugcacucgucccggccuau.................	5539	0	S04
....uauugcacucgucccggccuauc................	17	0	S04
....uauugcacucgucccggccuauca...............	6	0	S04
....auugcacucgucccggcc....................	2	0	S04
....auugcacucgucccggccu...................	2	0	S04
....auugcacucgucccggccua..................	49	0	S04
....auugcacucgucccggccuau.................	11	0	S04
....uugcacucgucccggccu...................	1	0	S04
....uugcacucgucccggccua..................	5	0	S04
....auauugcacucgucccggccu...................	1	0	S02
....auauugcacucgucccggccua..................	3	0	S02
....uauugcacucgucccggc.....................	20	0	S02
....uauugcacucgucccggcc....................	201	0	S02
....uauugcacucgucccggccu...................	10559	0	S02
....uauugcacucgucccggccua..................	11352	0	S02
....uauugcacucgucccggccuau.................	1262	0	S02
....uauugcacucgucccggccuauc................	1	0	S02
....uauugcacucgucccggccuauca...............	4	0	S02
....auugcacucgucccggcc....................	1	0	S02
....auugcacucgucccggccu...................	5	0	S02
....auugcacucgucccggccua..................	24	0	S02
....auugcacucgucccggccuau.................	1	0	S02
....uugcacucgucccggccu...................	2	0	S02
....uugcacucgucccggccua..................	1	0	S02
....ugcacucgucccggccua..................	1	0	S02

Provisional ID	:	Scaffold260_6921
Score total	:	1066.4
Score for star read(s)	:	3.9
Score for read counts	:	1059
Score for mfe	:	1.9
Score for randfold	:	1.6
Score for cons. seed	:	
Total read count	:	2089
Mature read count	:	2088
Loop read count	:	0
Star read count	:	1

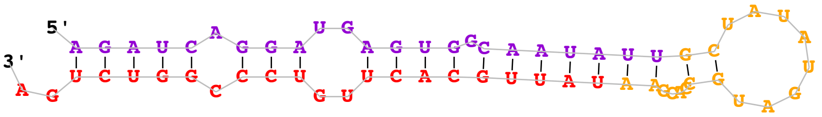

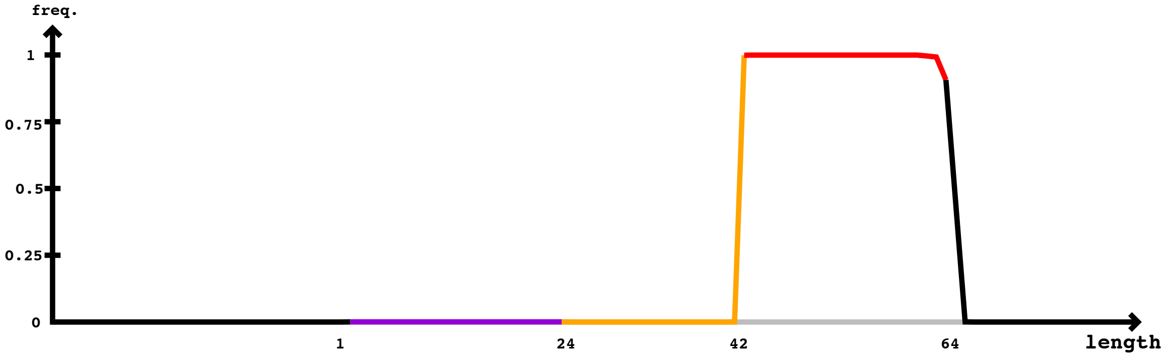

Star Mature

```
5'- uuaauuuggcagauaagucuguuguugucagaucaggaugaguggcaauauugcuauaugaugccacgaauauugcacuugucccggucugacaauagugacuccaacauc   -3'   obs
    uuaauuuggcagauaagucuguuguugucagaucaggaugaguggcaauauugcuauaugaugccacgaauauugcacuugucccggucugacaauagugacuccaacauc         exp
    .....(((......((((...(((((((((((.(((..((((.(((((((((........))....))))))))))..))).)))))))))))..))))))))....   reads   mm   sample
    ......................................................................uauugcacuugucccgguc......................   1   0   S05
    ......................................................................uauugcacuugucccggucu.....................   1   0   S05
    ......................................................................uauugcacuugucccggucug....................   14   0   S05
    ......................................................................uauugcacuugucccggucuga...................   192   0   S05
    ......................................................................uauugcacuugucccggucugac..................   1   0   S05

    .............................agaucaggaugaguggcaauauu...........................................................   1   0   S10
    ......................................................................uauugcacuugucccggucu.....................   2   0   S10
    ......................................................................uauugcacuugucccggucug....................   8   0   S10
    ......................................................................uauugcacuugucccggucuga...................   88   0   S10

    ......................................................................uauugcacuugucccgguc......................   1   0   S06
    ......................................................................uauugcacuugucccggucug....................   21   0   S06
    ......................................................................uauugcacuugucccggucuga...................   183   0   S06

    .....................................................................auauugcacuugucccggucuga...................   1   0   S01
    ......................................................................uauugcacuugucccgguc......................   1   0   S01
    ......................................................................uauugcacuugucccggucu.....................   2   0   S01
    ......................................................................uauugcacuugucccggucug....................   38   0   S01
    ......................................................................uauugcacuugucccggucuga...................   297   0   S01

    ......................................................................uauugcacuugucccggucu.....................   1   0   S07
    ......................................................................uauugcacuugucccggucug....................   8   0   S07
    ......................................................................uauugcacuugucccggucuga...................   92   0   S07
    ......................................................................uauugcacuugucccggucugac..................   1   0   S07

    ......................................................................uauugcacuugucccggucu.....................   1   0   S09
    ......................................................................uauugcacuugucccggucug....................   6   0   S09
    ......................................................................uauugcacuugucccggucuga...................   63   0   S09

    ......................................................................uauugcacuugucccgguc......................   3   0   S03
    ......................................................................uauugcacuugucccggucug....................   12   0   S03
    ......................................................................uauugcacuugucccggucuga...................   182   0   S03
```

Star Mature

```
uuaauuuggcagauaagucuguuguugucagaucaggaugaguggcaauauugcuauaugaugccacgaauauugcacuugucccggucugacaauagugacuccaacauc
......................................................................uauugcacuugucccggucugac..................	2	0	S03

......................................................................uauugcacuugucccggucug....................	10	0	S08
......................................................................uauugcacuugucccggucuga...................	94	0	S08

......................................................................uauugcacuugucccggucu.....................	1	0	S04
......................................................................uauugcacuugucccggucug....................	29	0	S04
......................................................................uauugcacuugucccggucuga...................	205	0	S04
......................................................................uauugcacuugucccggucugac..................	1	0	S04

......................................................................uauugcacuugucccgguc......................	1	0	S02
......................................................................uauugcacuugucccggucug....................	33	0	S02
......................................................................uauugcacuugucccggucuga...................	490	0	S02
......................................................................uauugcacuugucccggucugaca.................	1	0	S02
.......................................................................auugcacuugucccggucugac..................	1	0	S02
```

Provisional ID	:	Scaffold774_17511
Score total	:	374114.6
Score for star read(s)	:	3.9
Score for read counts	:	374107.3
Score for mfe	:	1.9
Score for randfold	:	1.6
Score for cons. seed	:	
Total read count	:	733807
Mature read count	:	731517
Loop read count	:	0
Star read count	:	2290

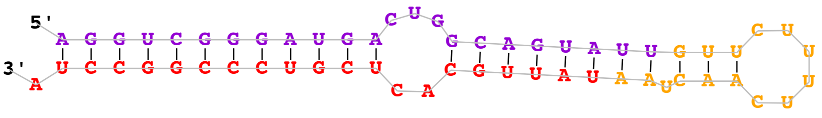

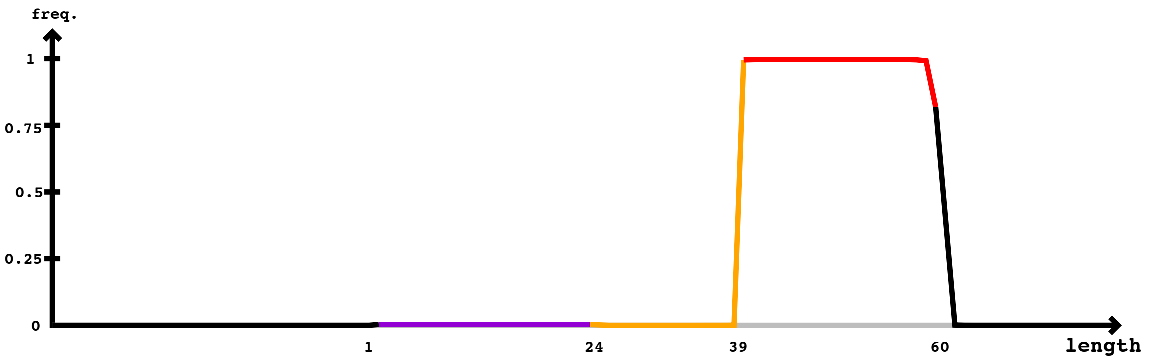


```
                                  Star                                 Mature
5'- ugguuagcagauaaguuuaguuuacucuggccaggucgggaugacuggcaguauuguucuuuucaacuaauauugcacucgucccggccuaucaagagugacucuugca   -3'   obs
    ugguuagcagauaaguuuaguuuacucuggccaggucgggaugacuggcaguauuguucuuuucaacuaauauugcacucgucccggccuaucaagagugacucuugca         exp
    .((((((.((.((((.....)))))))))))).(((((((((((...(((((((((((......))).))))))))..)))))))))))...((((((...))))))..   reads   mm   sample
    ............aaguuuaguuuacucuggcc.............................................................................   1       0    S08
    .............aguuuaguuuacucuggcc.............................................................................   5       0    S08
    ................................aggucgggaugacuggca...........................................................   1       0    S08
    ................................aggucgggaugacuggcagu.........................................................   6       0    S08
    ................................aggucgggaugacuggcagua........................................................   5       0    S08
    ................................aggucgggaugacuggcaguau.......................................................   48      0    S08
    ................................aggucgggaugacuggcaguauu......................................................   384     0    S08
    ................................aggucgggaugacuggcaguauuguucuuuucaacu.........................................   2       0    S08
    ................................aggucgggaugacuggcaguauuguucuuuucaacuaa.......................................   3       0    S08
    ..................................gucgggaugacuggcaguauuguucuuuucaacua........................................   1       0    S08
    ..............................................................ucaacuaauauugcacucgucccggccuau.................   1       0    S08
    ...............................................................caacuaauauugcacucguccc........................   1       0    S08
    ................................................................aacuaauauugcacucgucccggccua..................   1       0    S08
    .................................................................acuaauauugcacucgucccgg......................   2       0    S08
    .................................................................acuaauauugcacucgucccggccuau.................   1       0    S08
    ...................................................................uaauauugcacucgucccggccu...................   1       0    S08
    ...................................................................uaauauugcacucgucccggccua..................   1       0    S08
    ...................................................................uaauauugcacucgucccggccuau.................   2       0    S08
    ....................................................................aauauugcacucgucccggccua..................   1       0    S08
    ....................................................................aauauugcacucgucccggccuau.................   2       0    S08
    .....................................................................auauugcacucgucccggc.....................   2       0    S08
    .....................................................................auauugcacucgucccggccua..................   8       0    S08
    ......................................................................uauugcacucgucccggc.....................   58      0    S08
    ......................................................................uauugcacucgucccggcc....................   271     0    S08
    ......................................................................uauugcacucgucccggccu...................   14098   0    S08
    ......................................................................uauugcacucgucccggccua..................   71548   0    S08
    ......................................................................uauugcacucgucccggccuau.................   66154   0    S08
    ......................................................................uauugcacucgucccggccuauc................   210     0    S08
    ......................................................................uauugcacucgucccggccuauca...............   22      0    S08
    ......................................................................uauugcacucgucccggccuaucaa..............   1       0    S08
    .......................................................................auugcacucgucccggccu...................   12      0    S08
    .......................................................................auugcacucgucccggccua..................   72      0    S08
    .......................................................................auugcacucgucccggccuau.................   39      0    S08
```

Star **Mature**

```
ugguuagcagauaaguuuaguuuacucuggccaggucgggaugacuggcaguauuguucuuuucaacuaauauugcacucgucccggccuaucaagagugacucuugca
.......................................................................auugcacucgucccggccuauc................	2	0	S08
........................................................................uugcacucgucccggccu...................	18	0	S08
........................................................................uugcacucgucccggccua..................	111	0	S08
........................................................................uugcacucgucccggccuau.................	50	0	S08
........................................................................uugcacucgucccggccuauc................	1	0	S08
........................................................................uugcacucgucccggccuauca...............	4	0	S08
.........................................................................ugcacucgucccggccua..................	44	0	S08
.........................................................................ugcacucgucccggccuau.................	6	0	S08
..........................................................................gcacucgucccggccuau.................	6	0	S08

......gcagauaaguuuaguuuacucuggcc.............................................................................	1	0	S09
................................aggucgggaugacuggcagua........................................................	15	0	S09
................................aggucgggaugacuggcaguau.......................................................	59	0	S09
................................aggucgggaugacuggcaguauu......................................................	602	0	S09
................................aggucgggaugacuggcaguauuguucuuuucaac..........................................	1	0	S09
................................aggucgggaugacuggcaguauuguucuuuucaacu.........................................	1	0	S09
................................aggucgggaugacuggcaguauuguucuuuucaacuaa.......................................	1	0	S09
................................aggucgggaugacuggcaguauuguucuuuucaacuaau......................................	1	0	S09
................................aggucgggaugacuggcaguauuguucuuuucaacuaauauugca................................	1	0	S09
...............................................gcaguauuguucuuuucaacua........................................	1	0	S09
................................................caguauuguucuuuucaacua........................................	1	0	S09
.......................................................guucuuuucaacuaauauugcacucgucccggccuau.................	1	0	S09
...................................................................uaauauugcacucgucccggccuau.................	1	0	S09
.....................................................................auauugcacucgucccggccua..................	7	0	S09
......................................................................uauugcacucgucccggc.....................	83	0	S09
......................................................................uauugcacucgucccggcc....................	154	0	S09
......................................................................uauugcacucgucccggccu...................	5353	0	S09
......................................................................uauugcacucgucccggccua..................	21299	0	S09
......................................................................uauugcacucgucccggccuau.................	28388	0	S09
......................................................................uauugcacucgucccggccuauc................	81	0	S09
......................................................................uauugcacucgucccggccuauca...............	3	0	S09
.......................................................................auugcacucgucccggccu...................	3	0	S09
.......................................................................auugcacucgucccggccua..................	31	0	S09
.......................................................................auugcacucgucccggccuau.................	9	0	S09
........................................................................uugcacucgucccggccu...................	2	0	S09
........................................................................uugcacucgucccggccua..................	11	0	S09
........................................................................uugcacucgucccggccuau.................	2	0	S09
.........................................................................ugcacucgucccggccua..................	2	0	S09
.........................................................................ugcacucgucccggccuau.................	1	0	S09
..........................................................................gcacucgucccggccuau.................	1	0	S09

................................aggucgggaugacuggcaguauu......................................................	3	0	S03
...................................................................uaauauugcacucgucccggccua..................	1	0	S03
.....................................................................auauugcacucgucccggccu...................	3	0	S03
.....................................................................auauugcacucgucccggccua..................	18	0	S03
.....................................................................auauugcacucgucccggccuau.................	2	0	S03
......................................................................uauugcacucgucccggc.....................	100	0	S03
......................................................................uauugcacucgucccggcc....................	373	0	S03
......................................................................uauugcacucgucccggccu...................	16670	0	S03
......................................................................uauugcacucgucccggccua..................	38652	0	S03
......................................................................uauugcacucgucccggccuau.................	5711	0	S03
......................................................................uauugcacucgucccggccuauc................	9	0	S03
......................................................................uauugcacucgucccggccuauca...............	5	0	S03
.......................................................................auugcacucgucccggcc....................	1	0	S03
.......................................................................auugcacucgucccggccu...................	7	0	S03
.......................................................................auugcacucgucccggccua..................	59	0	S03
.......................................................................auugcacucgucccggccuau.................	14	0	S03
........................................................................uugcacucgucccggccu...................	2	0	S03
........................................................................uugcacucgucccggccua..................	8	0	S03
........................................................................uugcacucgucccggccuau.................	1	0	S03

.....................................................................auauugcacucgucccggccu...................	1	0	S02
.....................................................................auauugcacucgucccggccua..................	3	0	S02
......................................................................uauugcacucgucccggc.....................	20	0	S02
......................................................................uauugcacucgucccggcc....................	201	0	S02
......................................................................uauugcacucgucccggccu...................	10559	0	S02
......................................................................uauugcacucgucccggccua..................	11352	0	S02
......................................................................uauugcacucgucccggccuau.................	1262	0	S02
......................................................................uauugcacucgucccggccuauc................	1	0	S02
......................................................................uauugcacucgucccggccuauca...............	4	0	S02
```

Star **Mature**

```
ugguuagcagauaaguuuaguuuacucuggccaggucgggaugacuggcaguauuguucuuuucaacuaauauugcacucgucccggccuaucaagagugacucuugca
.......................................................................auugcacucgucccggcc....................    1       0   S02
.......................................................................auugcacucgucccggccu...................    5       0   S02
.......................................................................auugcacucgucccggccua..................    24      0   S02
.......................................................................auugcacucgucccggccuau.................    1       0   S02
........................................................................uugcacucgucccggccu...................    2       0   S02
........................................................................uugcacucgucccggccua..................    1       0   S02
.........................................................................ugcacucgucccggccua..................    1       0   S02

................................aggucgggaugacuggcaguauu......................................................    7       0   S04
.....................................................................auauugcacucgucccggccu...................    4       0   S04
.....................................................................auauugcacucgucccggccua..................    10      0   S04
.....................................................................auauugcacucgucccggccuau.................    4       0   S04
......................................................................uauugcacucgucccggc.....................    150     0   S04
......................................................................uauugcacucgucccggcc....................    421     0   S04
......................................................................uauugcacucgucccggccu...................    15760   0   S04
......................................................................uauugcacucgucccggccua..................    37100   0   S04
......................................................................uauugcacucgucccggccuau.................    5539    0   S04
......................................................................uauugcacucgucccggccuauc................    17      0   S04
......................................................................uauugcacucgucccggccuauca...............    6       0   S04
.......................................................................auugcacucgucccggcc....................    2       0   S04
.......................................................................auugcacucgucccggccu...................    2       0   S04
.......................................................................auugcacucgucccggccua..................    49      0   S04
.......................................................................auugcacucgucccggccuau.................    11      0   S04
........................................................................uugcacucgucccggccu...................    1       0   S04
........................................................................uugcacucgucccggccua..................    5       0   S04

..guuagcagauaaguuuaguuuacucuggcc.............................................................................    2       0   S10
........agauaaguuuaguuuacucuggcc.............................................................................    1       0   S10
............aaguuuaguuuacucuggcc.............................................................................    1       0   S10
.......................acucuggccaggucgggaugac................................................................    1       0   S10
................................aggucgggaugacuggca...........................................................    2       0   S10
................................aggucgggaugacuggcagu.........................................................    7       0   S10
................................aggucgggaugacuggcagua........................................................    11      0   S10
................................aggucgggaugacuggcaguau.......................................................    62      0   S10
................................aggucgggaugacuggcaguauu......................................................    649     0   S10
................................aggucgggaugacuggcaguauuguucuuuucaac..........................................    2       0   S10
................................aggucgggaugacuggcaguauuguucuuuucaacuaau......................................    2       0   S10
..............................................ggcaguauuguucuuuucaacua........................................    1       0   S10
................................................................aacuaauauugcacucgucccggccua..................    1       0   S10
...................................................................uaauauugcacucgucccggccua..................    2       0   S10
...................................................................uaauauugcacucgucccggccuau.................    1       0   S10
....................................................................aauauugcacucgucccggccua..................    1       0   S10
....................................................................aauauugcacucgucccggccuau.................    2       0   S10
.....................................................................auauugcacucgucccggccu...................    1       0   S10
.....................................................................auauugcacucgucccggccua..................    4       0   S10
......................................................................uauugcacucgucccggc.....................    107     0   S10
......................................................................uauugcacucgucccggcc....................    202     0   S10
......................................................................uauugcacucgucccggccu...................    7235    0   S10
......................................................................uauugcacucgucccggccua..................    31689   0   S10
......................................................................uauugcacucgucccggccuau.................    43371   0   S10
......................................................................uauugcacucgucccggccuauc................    93      0   S10
......................................................................uauugcacucgucccggccuauca...............    9       0   S10
.......................................................................auugcacucgucccggccu...................    8       0   S10
.......................................................................auugcacucgucccggccua..................    45      0   S10
.......................................................................auugcacucgucccggccuau.................    11      0   S10
.......................................................................auugcacucgucccggccuauc................    1       0   S10
........................................................................uugcacucgucccggccu...................    1       0   S10
........................................................................uugcacucgucccggccua..................    9       0   S10
........................................................................uugcacucgucccggccuau.................    8       0   S10
.........................................................................ugcacucgucccggccua..................    1       0   S10
.........................................................................ugcacucgucccggccuau.................    1       0   S10
..........................................................................gcacucgucccggccuau.................    2       0   S10

................................aggucgggaugacuggcaguau.......................................................    1       0   S05
................................aggucgggaugacuggcaguauu......................................................    9       0   S05
...............................................................caacuaauauugcacucg............................    1       0   S05
................................................................aacuaauauugcacucgucccggccu...................    1       0   S05
..................................................................cuaauauugcacucgucccggc.....................    1       0   S05
...................................................................uaauauugcacucgucccggccu...................    1       0   S05
...................................................................uaauauugcacucgucccggccua..................    3       0   S05
```

Star **Mature**

```
ugguuagcagauaaguuuaguuuacucuggccaggucgggaugacuggcaguauuguucuuuucaacuaauauugcacucgucccggccuaucaagagugacucuugca
....................................................................aauauugcacucgucccggcc....................    1       0   S05
....................................................................aauauugcacucgucccggccu...................    1       0   S05
....................................................................aauauugcacucgucccggccua..................    4       0   S05
....................................................................aauauugcacucgucccggccuau.................    1       0   S05
.....................................................................auauugcacucgucccggcc....................    1       0   S05
.....................................................................auauugcacucgucccggccu...................    2       0   S05
.....................................................................auauugcacucgucccggccua..................    18      0   S05
.....................................................................auauugcacucgucccggccuau.................    1       0   S05
......................................................................uauugcacucgucccggc.....................    145     0   S05
......................................................................uauugcacucgucccggcc....................    343     0   S05
......................................................................uauugcacucgucccggccu...................    14106   0   S05
......................................................................uauugcacucgucccggccua..................    39247   0   S05
......................................................................uauugcacucgucccggccuau.................    7188    0   S05
......................................................................uauugcacucgucccggccuauc................    16      0   S05
......................................................................uauugcacucgucccggccuauca...............    8       0   S05
.......................................................................auugcacucgucccggccu...................    9       0   S05
.......................................................................auugcacucgucccggccua..................    74      0   S05
.......................................................................auugcacucgucccggccuau.................    10      0   S05
.......................................................................auugcacucgucccggccuauc................    1       0   S05
........................................................................uugcacucgucccggccu...................    5       0   S05
........................................................................uugcacucgucccggccua..................    13      0   S05
........................................................................uugcacucgucccggccuau.................    3       0   S05
.........................................................................ugcacucgucccggccua..................    8       0   S05
.........................................................................ugcacucgucccggccuau.................    1       0   S05
..........................................................................gcacucgucccggccuau.................    1       0   S05

.........gauaaguuuaguuuacucuggcc.............................................................................    1       0   S07
.............aguuuaguuuacucuggcc.............................................................................    1       0   S07
................................aggucgggaugacuggca...........................................................    1       0   S07
................................aggucgggaugacuggcagu.........................................................    3       0   S07
................................aggucgggaugacuggcagua........................................................    6       0   S07
................................aggucgggaugacuggcaguau.......................................................    27      0   S07
................................aggucgggaugacuggcaguauu......................................................    342     0   S07
................................aggucgggaugacuggcaguauuguucuuuucaac..........................................    2       0   S07
................................aggucgggaugacuggcaguauuguucuuuucaacu.........................................    2       0   S07
................................aggucgggaugacuggcaguauuguucuuuucaacua........................................    2       0   S07
................................aggucgggaugacuggcaguauuguucuuuucaacuaa.......................................    1       0   S07
................................................................aacuaauauugcacucgucccggccua..................    1       0   S07
...................................................................uaauauugcacucgucccggcc....................    1       0   S07
...................................................................uaauauugcacucgucccggccuau.................    1       0   S07
....................................................................aauauugcacucgucccggcc....................    1       0   S07
....................................................................aauauugcacucgucccggccu...................    1       0   S07
.....................................................................auauugcacucgucccggccu...................    8       0   S07
.....................................................................auauugcacucgucccggccua..................    15      0   S07
.....................................................................auauugcacucgucccggccuau.................    4       0   S07
......................................................................uauugcacucgucccggc.....................    109     0   S07
......................................................................uauugcacucgucccggcc....................    256     0   S07
......................................................................uauugcacucgucccggccu...................    16391   0   S07
......................................................................uauugcacucgucccggccua..................    54170   0   S07
......................................................................uauugcacucgucccggccuau.................    47912   0   S07
......................................................................uauugcacucgucccggccuauc................    140     0   S07
......................................................................uauugcacucgucccggccuauca...............    14      0   S07
......................................................................uauugcacucgucccggccuaucaa..............    2       0   S07
.......................................................................auugcacucgucccggccu...................    11      0   S07
.......................................................................auugcacucgucccggccua..................    55      0   S07
.......................................................................auugcacucgucccggccuau.................    21      0   S07
........................................................................uugcacucgucccggccu...................    1       0   S07
........................................................................uugcacucgucccggccua..................    13      0   S07
........................................................................uugcacucgucccggccuau.................    7       0   S07
.........................................................................ugcacucgucccggccua..................    4       0   S07
.........................................................................ugcacucgucccggccuau.................    1       0   S07

.....................................................................auauugcacucgucccggccu...................    1       0   S01
.....................................................................auauugcacucgucccggccua..................    2       0   S01
.....................................................................auauugcacucgucccggccuau.................    1       0   S01
......................................................................uauugcacucgucccggc.....................    19      0   S01
......................................................................uauugcacucgucccggcc....................    202     0   S01
......................................................................uauugcacucgucccggccu...................    9744    0   S01
......................................................................uauugcacucgucccggccua..................    6994    0   S01
......................................................................uauugcacucgucccggccuau.................    689     0   S01
```

```
                                Star                                  Mature
ugguuagcagauaaguuuaguuuacucuggccaggucgggaugacuggcaguauuguucuuuucaacuaauauugcacucgucccggccuaucaagagugacucuugca
......................................................................uauugcacucgucccggccuauca...............    6      0    S01
......................................................................uauugcacucgucccggccuaucaa..............    3      0    S01
.......................................................................auugcacucgucccggccu...................    1      0    S01
.......................................................................auugcacucgucccggccua..................    11     0    S01
........................................................................uugcacucgucccggccu...................    1      0    S01
.........................................................................ugcacucgucccggccua..................    2      0    S01

................................aggucgggaugacuggca...........................................................    1      0    S06
................................aggucgggaugacuggcagu.........................................................    1      0    S06
................................aggucgggaugacuggcaguau.......................................................    10     0    S06
................................aggucgggaugacuggcaguauu......................................................    28     0    S06
..................................................................cuaauauugcacucgucccggccua..................    1      0    S06
..................................................................cuaauauugcacucgucccggccuau.................    1      0    S06
...................................................................uaauauugcacucgucccgg......................    1      0    S06
...................................................................uaauauugcacucgucccggcc....................    2      0    S06
...................................................................uaauauugcacucgucccggccua..................    1      0    S06
....................................................................aauauugcacucgucccggccu...................    6      0    S06
....................................................................aauauugcacucgucccggccua..................    1      0    S06
....................................................................aauauugcacucgucccggccuau.................    3      0    S06
.....................................................................auauugcacucgucccggccu...................    7      0    S06
.....................................................................auauugcacucgucccggccua..................    20     0    S06
.....................................................................auauugcacucgucccggccuau.................    3      0    S06
......................................................................uauugcacucgucccggc.....................    84     0    S06
......................................................................uauugcacucgucccggcc....................    403    0    S06
......................................................................uauugcacucgucccggccu...................    17205  0    S06
......................................................................uauugcacucgucccggccua..................    55660  0    S06
......................................................................uauugcacucgucccggccuau.................    24770  0    S06
......................................................................uauugcacucgucccggccuauc................    60     0    S06
......................................................................uauugcacucgucccggccuauca...............    11     0    S06
......................................................................uauugcacucgucccggccuaucaa..............    1      0    S06
.......................................................................auugcacucgucccggccu...................    6      0    S06
.......................................................................auugcacucgucccggccua..................    67     0    S06
.......................................................................auugcacucgucccggccuau.................    29     0    S06
........................................................................uugcacucgucccggccu...................    4      0    S06
........................................................................uugcacucgucccggccua..................    14     0    S06
........................................................................uugcacucgucccggccuau.................    9      0    S06
.........................................................................ugcacucgucccggccua..................    7      0    S06
.........................................................................ugcacucgucccggccuau.................    2      0    S06
..........................................................................gcacucgucccggccuau.................    1      0    S06
```

Provisional ID	:	Scaffold133_4002
Score total	:	4073.2
Score for star read(s)	:	3.9
Score for read counts	:	4066
Score for mfe	:	1.7
Score for randfold	:	1.6
Score for cons. seed	:	
Total read count	:	7987
Mature read count	:	6156
Loop read count	:	0
Star read count	:	1831

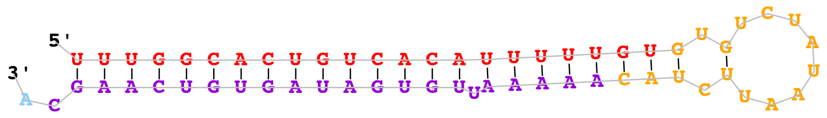

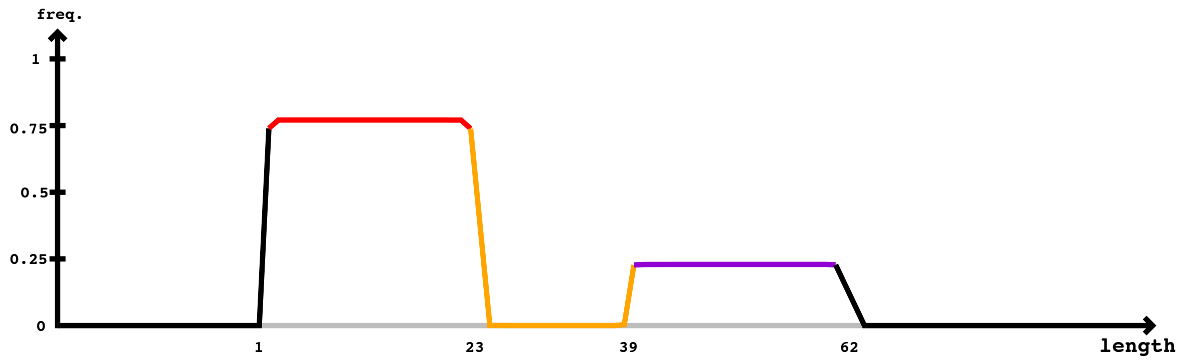

Mature **Star**

```
5'- aacgaacuccucuucuguuauuuggcacugucacauuuuuguguguсuauaaauucuacaaaaauugugauagugucaagcaacaugaagagcguugaaucaucgauuccaua   -3'    obs
    aacgaacuccucuucuguuauuuggcacugucacauuuuuguguguсuauaaauucuacaaaaauugugauagugucaagcaacaugaagagcguugaaucaucgauuccaua          exp
    ....(((..((((((((((.((((((((((((((((((((((((.(........).)))))))).))))))))))))))).)))).)))))).)))(((((...)))))....    reads     mm        sample
    ....................uuuggcacugucacauuuuug........................................................................    14        0         S04
    ....................uuuggcacugucacauuuuugu.......................................................................    298       0         S04
    ....................uuuggcacugucacauuuuugug......................................................................    1         0         S04
    .....................uuggcacugucacauuuuugu.......................................................................    14        0         S04
    ..........................................................caaaaauugugauagugucaagc................................    1         0         S04
    ...........................................................aaaaauugugauagugucaagc................................    89        0         S04
    ...........................................................aaaaauugugauagugucaagca...............................    11        0         S04
    ............................................................aaaauugugauagugucaagc................................    1         0         S04

    ....................uuuggcacugucacauuuuug........................................................................    5         0         S02
    ....................uuuggcacugucacauuuuugu.......................................................................    121       0         S02
    ....................uuuggcacugucacauuuuugug......................................................................    3         0         S02
    .....................uuggcacugucacauuuuug........................................................................    1         0         S02
    .....................uuggcacugucacauuuuugu.......................................................................    5         0         S02
    ...........................................................aaaaauugugauagugucaagc................................    33        0         S02
    ...........................................................aaaaauugugauagugucaagca...............................    2         0         S02
    ............................................................aaaauugugauagugucaagc................................    1         0         S02
    ............................................................aaaauugugauagugucaagca...............................    1         0         S02

    ....................uuuggcacugucacauuuuug........................................................................    15        0         S03
    ....................uuuggcacugucacauuuuugu.......................................................................    379       0         S03
    ....................uuuggcacugucacauuuuugug......................................................................    1         0         S03
    ....................uuuggcacugucacauuuuugugugu...................................................................    1         0         S03
    .....................uuggcacugucacauuuuugu.......................................................................    7         0         S03
    ..........................................................caaaaauugugauagugucaagc................................    4         0         S03
    ...........................................................aaaaauugugauagugucaagc................................    79        0         S03
    ...........................................................aaaaauugugauagugucaagca...............................    14        0         S03

    ....................uuuggcacugucacauuuuug........................................................................    69        0         S09
    ....................uuuggcacugucacauuuuugu.......................................................................    1661      0         S09
    ....................uuuggcacugucacauuuuugug......................................................................    11        0         S09
    ....................uuuggcacugucacauuuuugugugu...................................................................    1         0         S09
    .....................uuggcacugucacauuuuug........................................................................    1         0         S09
```

Mature **Star**

```
aacgaacuccucuucuguuauuuggcacugucacauuuuugugugucuauaauucuacaaaaauugugauagugucaagcaacaugaagagcguugaaucaucgauuccaua
.....................uuggcacugucacauuuuugu......................................................................    62     0    S09
.........................................................caaaaauugugauagugucaagc................................    4      0    S09
..........................................................aaaaauugugauagugucaag.................................    5      0    S09
..........................................................aaaaauugugauagugucaagc................................    503    0    S09
..........................................................aaaaauugugauagugucaagca...............................    37     0    S09
..........................................................aaaaauugugauagugucaagcaa..............................    1      0    S09
...........................................................aaaauugugauagugucaagc................................    7      0    S09

....................uuuggcacugucacauuuuug.......................................................................    10     0    S08
....................uuuggcacugucacauuuuugu......................................................................    126    0    S08
....................uuuggcacugucacauuuuugug.....................................................................    2      0    S08
.....................uuggcacugucacauuuuug.......................................................................    1      0    S08
.....................uuggcacugucacauuuuugu......................................................................    10     0    S08
.........................................................caaaaauugugauagugucaagc................................    5      0    S08
..........................................................aaaaauugugauagugucaagc................................    49     0    S08
..........................................................aaaaauugugauagugucaagca...............................    4      0    S08

....................uuuggcacugucacauuuuug.......................................................................    3      0    S01
....................uuuggcacugucacauuuuugu......................................................................    49     0    S01
.....................uuggcacugucacauuuuugu......................................................................    2      0    S01
..........................................................aaaaauugugauagugucaagc................................    5      0    S01

....................uuuggcacugucacauuuuug.......................................................................    17     0    S06
....................uuuggcacugucacauuuuugu......................................................................    277    0    S06
....................uuuggcacugucacauuuuugug.....................................................................    2      0    S06
.....................uuggcacugucacauuuuug.......................................................................    2      0    S06
.....................uuggcacugucacauuuuugu......................................................................    12     0    S06
.........................................................caaaaauugugauagugucaagc................................    5      0    S06
..........................................................aaaaauugugauagugucaagc................................    42     0    S06
..........................................................aaaaauugugauagugucaagca...............................    7      0    S06

....................uuuggcacugucacauuuuug.......................................................................    10     0    S07
....................uuuggcacugucacauuuuugu......................................................................    214    0    S07
.....................uuggcacugucacauuuuug.......................................................................    1      0    S07
.....................uuggcacugucacauuuuugu......................................................................    9      0    S07
.....................uuggcacugucacauuuuugug.....................................................................    1      0    S07
.........................................................caaaaauugugauagugucaagc................................    2      0    S07
..........................................................aaaaauugugauagugucaagc................................    48     0    S07
..........................................................aaaaauugugauagugucaagca...............................    7      0    S07

....................uuuggcacugucacauuuuug.......................................................................    14     0    S05
....................uuuggcacugucacauuuuugu......................................................................    345    0    S05
....................uuuggcacugucacauuuuugugu....................................................................    1      0    S05
.....................uuggcacugucacauuuuugu......................................................................    10     0    S05
.....................uuggcacugucacauuuuugug.....................................................................    1      0    S05
.........................................................caaaaauugugauagugucaagc................................    3      0    S05
..........................................................aaaaauugugauagugucaagc................................    118    0    S05
..........................................................aaaaauugugauagugucaagca...............................    3      0    S05
............................................................aaauugugauagugucaagc................................    1      0    S05
..............................................................auugugauagugucaagc................................    1      0    S05

....................uuuggcacugucacauuu..........................................................................    1      0    S10
....................uuuggcacugucacauuuuug.......................................................................    88     0    S10
....................uuuggcacugucacauuuuugu......................................................................    2164   0    S10
....................uuuggcacugucacauuuuugug.....................................................................    4      0    S10
.....................uuggcacugucacauuuuug.......................................................................    6      0    S10
.....................uuggcacugucacauuuuugu......................................................................    98     0    S10
.....................uuggcacugucacauuuuugug.....................................................................    6      0    S10
.........................................................caaaaauugugauagugucaagc................................    4      0    S10
..........................................................aaaaauugugauagugucaag.................................    6      0    S10
..........................................................aaaaauugugauagugucaagc................................    680    0    S10
..........................................................aaaaauugugauagugucaagca...............................    45     0    S10
...........................................................aaaauugugauagugucaagc................................    1      0    S10
...........................................................aaaauugugauagugucaagca...............................    1      0    S10
............................................................aaauugugauagugucaagc................................    1      0    S10
```

Provisional ID	: Scaffold1373_25321
Score total	: 1019.4
Score for star read(s)	: 3.9
Score for read counts	: 1012.1
Score for mfe	: 1.8
Score for randfold	: 1.6
Score for cons. seed	:
Total read count	: 1997
Mature read count	: 1771
Loop read count	: 0
Star read count	: 226

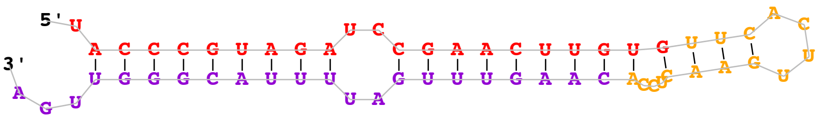

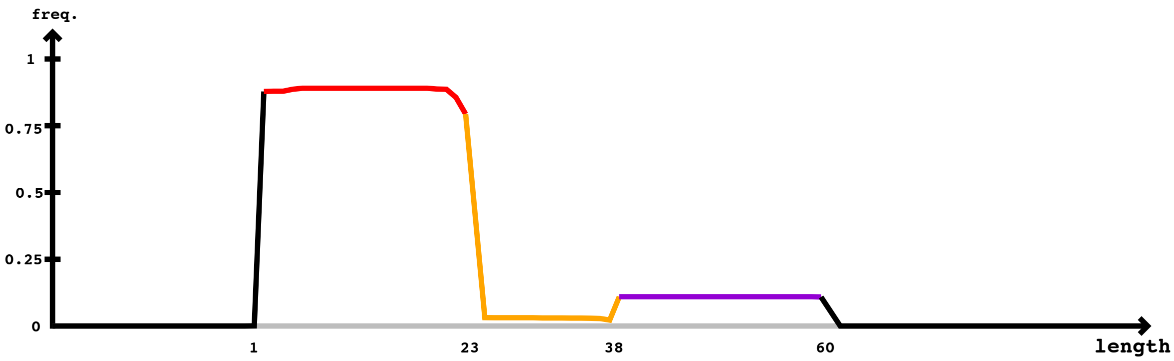

Mature **Star**

```
5'- cgucauagaccgcucacuucuacccguagauccgaacuuguguucacuugaacuccacaaguuugauuuuacggguugacguggguaggauuuuacacuuuuccuggaagaa   -3'   obs
    cgucauagaccgcucacuucuacccguagauccgaacuuguguucacuugaacuccacaaguuugauuuuacggguugacguggguaggauuuuacacuuuuccuggaagaa         exp
    .........((((((((.((.(((((((((..(((((((((((((....))))...)))))))))..))))))))).)).)))))).))........((((.....))))..   reads   mm   sample
    ....................uacccguagauccgaacuug........................................................................   6       0    S10
    ....................uacccguagauccgaacuugu.......................................................................   11      0    S10
    ....................uacccguagauccgaacuugug......................................................................   108     0    S10
    ....................uacccguagauccgaacuugugu.....................................................................   1       0    S10
    ....................uacccguagauccgaacuuguguucacuu...............................................................   1       0    S10
    ....................uacccguagauccgaacuuguguucacuugaacucca.......................................................   2       0    S10
    .....................acccguagauccgaacuugug......................................................................   1       0    S10
    .........................................................caaguuugauuuuacggguuga.................................   28      0    S10

    ....................uacccguagauccgaacu..........................................................................   1       0    S05
    ....................uacccguagauccgaacuug........................................................................   11      0    S05
    ....................uacccguagauccgaacuugu.......................................................................   10      0    S05
    ....................uacccguagauccgaacuugug......................................................................   194     0    S05
    ....................uacccguagauccgaacuugugu.....................................................................   1       0    S05
    ....................uacccguagauccgaacuuguguucacuugaacucc........................................................   1       0    S05
    ....................uacccguagauccgaacuuguguucacuugaacucca.......................................................   2       0    S05
    .......................ccguagauccgaacuugug......................................................................   3       0    S05
    ........................cguagauccgaacuuguguucacuugaacucca.......................................................   1       0    S05
    .........................................................caaguuugauuuuacggguuga.................................   15      0    S05

    ....................uacccguagauccgaacu..........................................................................   1       0    S07
    ....................uacccguagauccgaacuug........................................................................   6       0    S07
    ....................uacccguagauccgaacuugu.......................................................................   19      0    S07
    ....................uacccguagauccgaacuugug......................................................................   254     0    S07
    ....................uacccguagauccgaacuugugu.....................................................................   2       0    S07
    ....................uacccguagauccgaacuuguguucacuugaacuc.........................................................   1       0    S07
    ....................uacccguagauccgaacuuguguucacuugaacucc........................................................   3       0    S07
    ....................uacccguagauccgaacuuguguucacuugaacucca.......................................................   5       0    S07
    .......................ccguagauccgaacuugug......................................................................   1       0    S07
    ........................cguagauccgaacuugug......................................................................   2       0    S07
    .........................................................caaguuugauuuuacggguuga.................................   27      0    S07

    ....................uacccguagauccgaacuu.........................................................................   1       0    S06
```

```
                    Mature                              Star
cgucauagaccgcucacuucuacccguagauccgaacuuguguucacuugaacuccacaaguuugauuuuacggguugacguggguaggauuuuacacuuuuccuggaagaa
....................uacccguagauccgaacuug........................................................................   7     0   S06
....................uacccguagauccgaacuugu.......................................................................   11    0   S06
....................uacccguagauccgaacuugug......................................................................   148   0   S06
....................uacccguagauccgaacuugugu.....................................................................   3     0   S06
....................uacccguagauccgaacuuguguucacuugaacu..........................................................   1     0   S06
....................uacccguagauccgaacuuguguucacuugaacucca.......................................................   3     0   S06
.......................ccguagauccgaacuugug......................................................................   3     0   S06
........................cguagauccgaacuugug......................................................................   1     0   S06
........................cguagauccgaacuuguguucacuugaacucca.......................................................   2     0   S06
.........................................................caaguuugauuuuacggguuga.................................   17    0   S06
.........................................................caaguuugauuuuacggguugac................................   1     0   S06

....................uacccguagauccgaacu..........................................................................   1     0   S01
....................uacccguagauccgaacuu.........................................................................   1     0   S01
....................uacccguagauccgaacuug........................................................................   3     0   S01
....................uacccguagauccgaacuugu.......................................................................   17    0   S01
....................uacccguagauccgaacuugug......................................................................   103   0   S01
....................uacccguagauccgaacuuguguucacuugaacucc........................................................   3     0   S01
....................uacccguagauccgaacuuguguucacuugaacucca.......................................................   3     0   S01
.......................ccguagauccgaacuugug......................................................................   1     0   S01
.........................................................caaguuugauuuuacggguuga.................................   9     0   S01

....................uacccguagauccgaacuug........................................................................   4     0   S08
....................uacccguagauccgaacuugu.......................................................................   5     0   S08
....................uacccguagauccgaacuugug......................................................................   142   0   S08
....................uacccguagauccgaacuuguguucacuugaacucca.......................................................   5     0   S08
.......................ccguagauccgaacuugug......................................................................   5     0   S08
........................cguagauccgaacuugug......................................................................   1     0   S08
.........................................................caaguuugauuuuacggguug..................................   2     0   S08
.........................................................caaguuugauuuuacggguuga.................................   39    0   S08

....................uacccguagauccgaacuug........................................................................   2     0   S09
....................uacccguagauccgaacuugu.......................................................................   12    0   S09
....................uacccguagauccgaacuugug......................................................................   70    0   S09
....................uacccguagauccgaacuuguguucacuugaacucca.......................................................   3     0   S09
.....................acccguagauccgaacuugug......................................................................   1     0   S09
.......................ccguagauccgaacuugug......................................................................   1     0   S09
.........................................................caaguuugauuuuacggguuga.................................   39    0   S09

...................cuacccguagauccgaacuugug......................................................................   1     0   S03
....................uacccguagauccgaacu..........................................................................   2     0   S03
....................uacccguagauccgaacuug........................................................................   8     0   S03
....................uacccguagauccgaacuugu.......................................................................   14    0   S03
....................uacccguagauccgaacuugug......................................................................   201   0   S03
....................uacccguagauccgaacuuguguucacuu...............................................................   1     0   S03
....................uacccguagauccgaacuuguguucacuugaacucc........................................................   2     0   S03
....................uacccguagauccgaacuuguguucacuugaacucca.......................................................   6     0   S03
.........................................................caaguuugauuuuacggguuga.................................   11    0   S03

....................uacccguagauccgaacu..........................................................................   1     0   S02
....................uacccguagauccgaacuug........................................................................   10    0   S02
....................uacccguagauccgaacuugu.......................................................................   19    0   S02
....................uacccguagauccgaacuugug......................................................................   130   0   S02
....................uacccguagauccgaacuuguguu....................................................................   1     0   S02
....................uacccguagauccgaacuuguguucacuugaa............................................................   1     0   S02
....................uacccguagauccgaacuuguguucacuugaacucc........................................................   1     0   S02
....................uacccguagauccgaacuuguguucacuugaacucca.......................................................   3     0   S02
.......................ccguagauccgaacuugug......................................................................   1     0   S02
.........................................................caaguuugauuuuacggguuga.................................   17    0   S02

....................uacccguagauccgaacuug........................................................................   6     0   S04
....................uacccguagauccgaacuugu.......................................................................   10    0   S04
....................uacccguagauccgaacuugug......................................................................   191   0   S04
....................uacccguagauccgaacuuguguucacuugaacuc.........................................................   1     0   S04
....................uacccguagauccgaacuuguguucacuugaacucc........................................................   2     0   S04
....................uacccguagauccgaacuuguguucacuugaacucca.......................................................   11    0   S04
........................cguagauccgaacuugugu.....................................................................   1     0   S04
.........................................................caaguuugauuuuacggguuga.................................   21    0   S04
```

Provisional ID	: Scaffold590_13964
Score total	: 131.9
Score for star read(s)	: 3.9
Score for read counts	: 124.5
Score for mfe	: 1.9
Score for randfold	: 1.6
Score for cons. seed	:
Total read count	: 256
Mature read count	: 250
Loop read count	: 0
Star read count	: 6

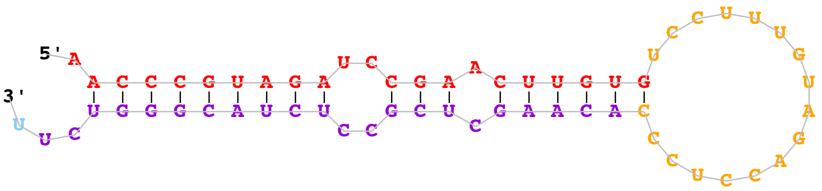

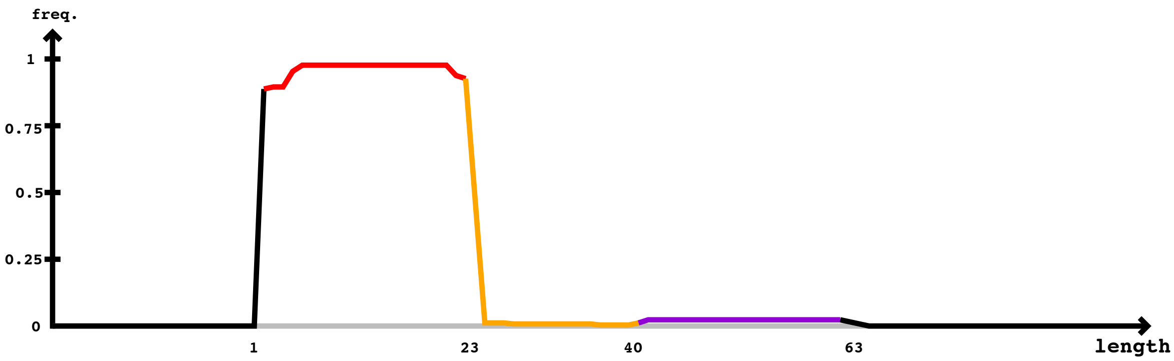

Mature Star

5'- gggguguagcugcuaagcgggaacccguagauccgaacuuguguccuuuguagaccucccacaagcucgccucuacgggucuuugcuuugcccugcgucaacucccccucau -3'	obs		
gggguguagcugcuaagcgggaacccguagauccgaacuuguguccuuuguagaccucccacaagcucgccucuacgggucuuugcuuugcccugcgucaacucccccucau	exp		
(((.((.((.((.((((((..(((((((((..(((.((((((...............)))))).)))..)))))))))..)))))).))...))....)).)))).......	reads	mm	sample
....................aacccguagauccgaacuug...	1	0	S04
....................aacccguagauccgaacuugu..	1	0	S04
....................aacccguagauccgaacuugug...	25	0	S04
........................cguagauccgaacuugugu..	1	0	S04
....................aacccguagauccgaacuugug...	6	0	S02
.......................ccguagauccgaacuugug...	1	0	S02
....................aacccguagauccgaacuugug...	10	0	S08
....................aacccguagauccgaacuuguguccuuuguagaccuccc..	1	0	S08
.......................ccguagauccgaacuugug...	5	0	S08
........................cguagauccgaacuugug...	1	0	S08
....................aacccguagauccgaacuugu..	1	0	S09
....................aacccguagauccgaacuugug...	8	0	S09
.....................acccguagauccgaacuugug...	1	0	S09
.......................ccguagauccgaacuugug...	1	0	S09
....caagcucgccucuacgggucu..............................	1	0	S09
....................aacccguagauccgaacuug...	1	0	S03
....................aacccguagauccgaacuugug...	4	0	S03
....................aacccguagauccgaacuuguguccuuuguagacc..	1	0	S03
....................aacccguagauccgaacuug...	1	0	S07
....................aacccguagauccgaacuugug...	15	0	S07
.......................ccguagauccgaacuugug...	1	0	S07
........................cguagauccgaacuugug...	2	0	S07
....................aacccguagauccgaacuugug...	7	0	S06
.......................ccguagauccgaacuugug...	3	0	S06
........................cguagauccgaacuugug...	1	0	S06
....................aacccguagauccgaacuugu..	1	0	S01

Mature Star			
ggguguagcugcuaagcgggaacccguagauccgaacuuguguccuuuguagaccucccacaagcucgccucuacgggucuuugcuuugcccugcgucaacuccccccucau			
....................aacccguagauccgaacuugug..	1	0	S01
.......................ccguagauccgaacuugug..	1	0	S01
....................aacccguagauccgaacuug..	7	0	S10
....................aacccguagauccgaacuugug..	128	0	S10
.....................acccguagauccgaacuugug..	1	0	S10
....acaagcucgccucuacgggucu...............................	3	0	S10
....caagcucgccucuacgggucu...............................	1	0	S10
....caagcucgccucuacgggucuu..............................	1	0	S10
....................aacccguagauccgaacuugug..	10	0	S05
.......................ccguagauccgaacuugug..	3	0	S05
........................cguagauccgaacuuguguccu..	1	0	S05

Provisional ID	:	Scaffold1373_25317
Score total	:	187
Score for star read(s)	:	3.9
Score for read counts	:	179.6
Score for mfe	:	1.9
Score for randfold	:	1.6
Score for cons. seed	:	
Total read count	:	364
Mature read count	:	357
Loop read count	:	0
Star read count	:	7

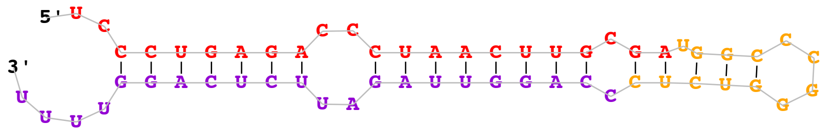

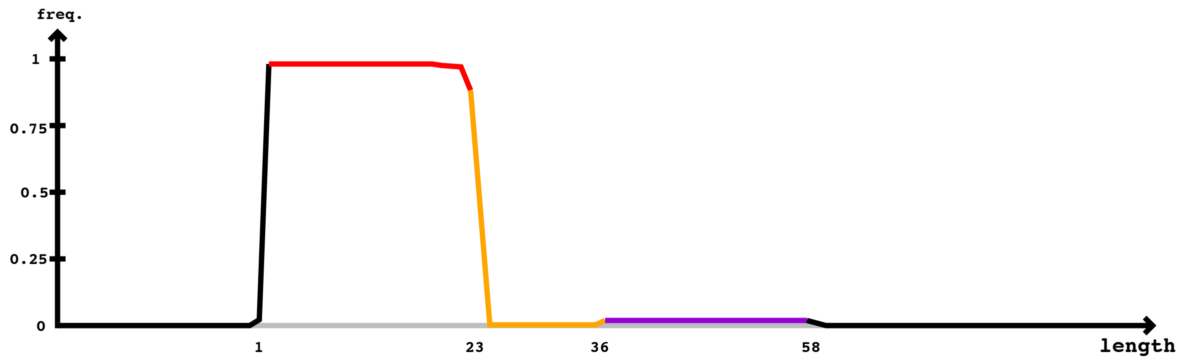

Mature **Star**

5'- uaauaaaccccuguaccccacucccugagacccuaacuugcgauggcccgggucucccagguuagauucucagguuuugggguugcaggucgaaaauucaucuuugcacagaaa -3'	obs		
uaauaaaccccuguaccccacucccugagacccuaacuugcgauggcccgggucucccagguuagauucucagguuuugggguugcaggucgaaaauucaucuuugcacagaaa	exp		
........((((((.((((...(((((((..((((((((.((.(((....))))).))))))))..)))))))...)))).))))))..........................	reads	mm	sample
....................cucccugagacccuaacuugcg...	1	0	S08
.....................ucccugagacccuaacuugcg...	8	0	S08
.....................ucccugagacccuaacuugcga..	83	0	S08
.....................ucccugagacccuaacuugc..	1	0	S03
.....................ucccugagacccuaacuugcg...	2	0	S03
.....................ucccugagacccuaacuugcga..	23	0	S03
.....................ucccugagacccuaacuugcg...	3	0	S09
.....................ucccugagacccuaacuugcga..	26	0	S09
....ccagguuagauucucagguuuu...................................	1	0	S09
....................cucccugagacccuaacuugcga..	1	0	S02
.....................ucccugagacccuaacuugcga..	8	0	S02
....................cucccugagacccuaacuugcg...	2	0	S04
.....................ucccugagacccuaacuugcga..	17	0	S04
.....................ucccugagacccuaacuugcgauggcccgggucuc...	1	0	S04
.....................ucccugagacccuaacuug...	1	0	S10
.....................ucccugagacccuaacuugcg...	2	0	S10
.....................ucccugagacccuaacuugcga..	13	0	S10
....ccagguuagauucucagguuuu...................................	1	0	S10
....................cucccugagacccuaacuugcg...	1	0	S05
.....................ucccugagacccuaacuu..	2	0	S05
.....................ucccugagacccuaacuugcga..	28	0	S05
....ccagguuagauucucagguuuu...................................	2	0	S05
....................cucccugagacccuaacuugcg...	1	0	S07
.....................ucccugagacccuaacuugcg...	7	0	S07
.....................ucccugagacccuaacuugcga..	87	0	S07
.....................ucccugagacccuaacuugcgau...	1	0	S07

```
                    Mature                                 Star
uaauaaccccuguaccccacucccugagacccuaacuugcgauggcccgggucucccagguuagauucucagguuuugggguugcaggucgaaaauucaucuuugcacagaaa
.......................................................ccagguuagauucucagguuuu...................................	2	0	S07

...................cucccugagacccuaacuugcg.......................................................................	2	0	S06
....................ucccugagacccuaacuugcg.......................................................................	2	0	S06
....................ucccugagacccuaacuugcga......................................................................	28	0	S06
.......................................................ccagguuagauucucagguuuu...................................	1	0	S06

....................ucccugagacccuaacuugcg.......................................................................	1	0	S01
....................ucccugagacccuaacuugcga......................................................................	6	0	S01
```

Provisional ID : Scaffold1466_26411
Score total : 51.3
Score for star read(s) : 3.9
Score for read counts : 43.5
Score for mfe : 2.4
Score for randfold : 1.6
Score for cons. seed :
Total read count : 97
Mature read count : 66
Loop read count : 0
Star read count : 31

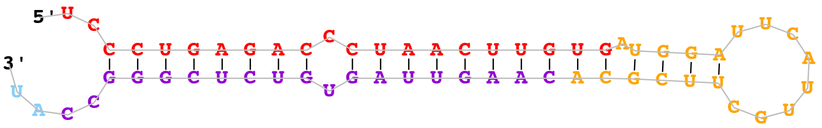

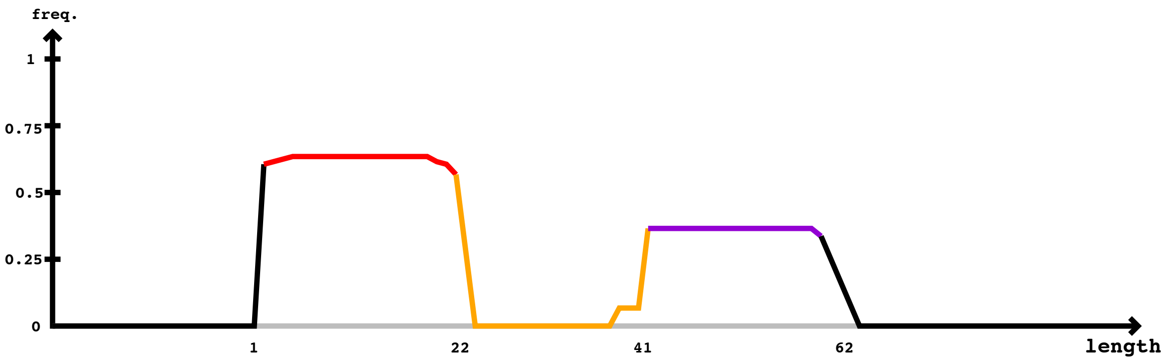

Mature Star

```
5'- gcccguguccugcuccccacucccugagacccuaacuugugauggauucauugcuucgcacaaguuagugucucgggccauggguggcaggugggcuccgucuccccaguc -3'   obs
    gcccguguccugcuccccacucccugagacccuaacuugugauggauucauugcuucgcacaaguuagugucucgggccauggguggcaggugggcuccgucuccccaguc        exp
    ((((...(((((..((((...((((((((.(((((((((.((((........))))))))))))).))))))))...))))..)))))))))...................   reads   mm   sample
    .........................................................gcacaaguuagugucucgggcc................................   1       0    S01
    ............................................................caaguuagugucucgggcc................................   1       0    S01

    ....................ucccugagacccuaacuugug......................................................................   6       0    S07
    ....................ucccugagacccuaacuuguga.....................................................................   1       0    S07
    .........................................................gcacaaguuagugucucgggc.................................   1       0    S07

    ....................ucccugagacccuaacuu.........................................................................   2       0    S05
    ....................ucccugagacccuaacuugu.......................................................................   1       0    S05
    ....................ucccugagacccuaacuugug......................................................................   4       0    S05
    .........................................................gcacaaguuagugucucgggc.................................   1       0    S05
    ............................................................caaguuagugucucgggcca...............................   1       0    S05

    ....................ucccugagacccuaacuug........................................................................   1       0    S10
    ....................ucccugagacccuaacuugu.......................................................................   1       0    S10
    ....................ucccugagacccuaacuugug......................................................................   33      0    S10
    .........................................................gcacaaguuagugucucgggcc................................   2       0    S10
    ............................................................caaguuagugucucgggcc................................   10      0    S10
    ............................................................caaguuagugucucgggcca...............................   9       0    S10
    ............................................................caaguuagugucucgggccau..............................   8       0    S10

    ....................ucccugagacccuaacuugu.......................................................................   2       0    S04
    ....................ucccugagacccuaacuugug......................................................................   1       0    S04
    .....................cccugagacccuaacuuguga.....................................................................   1       0    S04
    ............................................................caaguuagugucucgggcc................................   1       0    S04

    ....................ucccugagacccuaacuugug......................................................................   1       0    S02
    ......................ccugagacccuaacuuguga.....................................................................   1       0    S02
    .......................cugagacccuaacuugug......................................................................   1       0    S02
    .........................................................gcacaaguuagugucucgggcc................................   1       0    S02

    ....................ucccugagacccuaacuugug......................................................................   4       0    S09

    ....................ucccugagacccuaacuugug......................................................................   1       0    S03
```

```
                    Mature                                  Star
gcccguguccugcuccccacucccugagacccuaacuugugauggauucauugcuucgcacaaguuagugucucgggccaugggguggcaggugggcuccgucuccccaguc

....................ucccugagacccuaacuugug.......................................................................	5	0	S08
.........................................................gcacaaguuagugucucgggc..................................	1	0	S08
............................................................caaguuagugucucgggccau...............................	1	0	S08
```

Provisional ID	:	Scaffold313_8156
Score total	:	22578.9
Score for star read(s)	:	3.9
Score for read counts	:	22571.6
Score for mfe	:	1.8
Score for randfold	:	1.6
Score for cons. seed	:	
Total read count	:	44285
Mature read count	:	42538
Loop read count	:	0
Star read count	:	1747

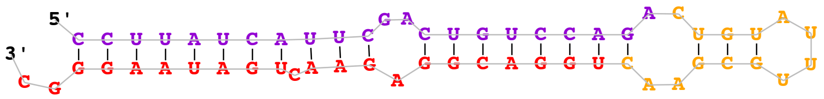

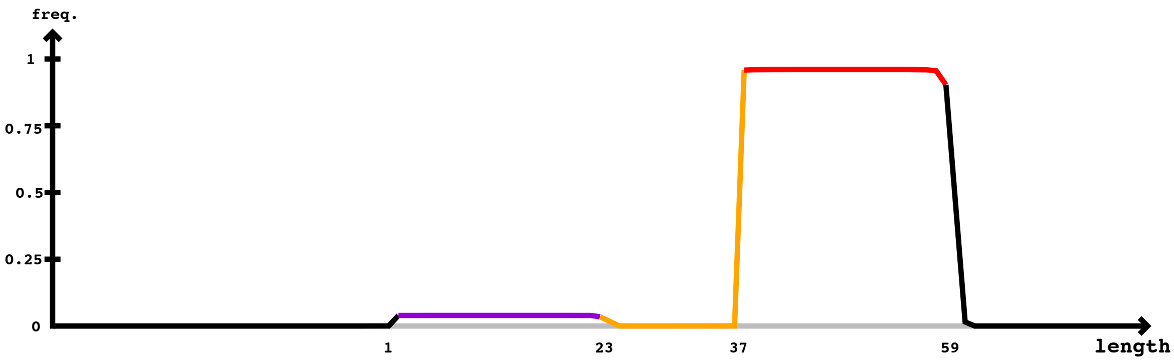

Star **Mature**

```
5'- gaagaacuacagaugaagaaaaguuuccacgucuccuuaucauucgacuguccagacuguauuugcgaacuggacggagaacugauaagggcuuguggcgacauaaaucauc  -3'   obs
    gaagaacuacagaugaagaaaaguuuccacgucuccuuaucauucgacuguccagacuguauuugcgaacuggacggagaacugauaagggcuuguggcgacauaaaucauc        exp
    ...........(((((......(((.(((((..((((((((((((..((((((((..(((....)))..)))))))).))).)))))))))..))))).))).....)))))   reads    mm    sample
    ..................................ccuuaucauucgacuguccag.........................................................   22       0     S04
    ..................................ccuuaucauucgacuguccaga........................................................   134      0     S04
    .......................................ucauucgacuguccagacuguauuugcgaa...........................................   1        0     S04
    ......................................................................uggacggagaacugauaag.......................   1        0     S04
    ......................................................................uggacggagaacugauaagg......................   4        0     S04
    ......................................................................uggacggagaacugauaaggg.....................   39       0     S04
    ......................................................................uggacggagaacugauaagggc....................   2119     0     S04
    ......................................................................uggacggagaacugauaagggcu...................   289      0     S04
    ......................................................................uggacggagaacugauaagggcuu..................   17       0     S04
    .......................................................................ggacggagaacugauaagggcu...................   1        0     S04
    .......................................................................ggacggagaacugauaagggcuu..................   2        0     S04

    ..................................ccuuaucauucgacuguccaga........................................................   6        0     S02
    ......................................................................uggacggagaacugauaa........................   5        0     S02
    ......................................................................uggacggagaacugauaag.......................   2        0     S02
    ......................................................................uggacggagaacugauaagg......................   4        0     S02
    ......................................................................uggacggagaacugauaaggg.....................   20       0     S02
    ......................................................................uggacggagaacugauaagggc....................   91       0     S02
    ......................................................................uggacggagaacugauaagggcu...................   8        0     S02
    .........................................................................acggagaacugauaagggcu...................   2        0     S02

    .................................uccuuaucauucgacuguccaga........................................................   1        0     S09
    ..................................ccuuaucauucgacuguccag.........................................................   15       0     S09
    ..................................ccuuaucauucgacuguccaga........................................................   228      0     S09
    ..................................ccuuaucauucgacuguccagac.......................................................   2        0     S09
    .....................................................................cuggacggagaacugauaagggc....................   1        0     S09
    ......................................................................uggacggagaacugauaa........................   2        0     S09
    ......................................................................uggacggagaacugauaag.......................   2        0     S09
    ......................................................................uggacggagaacugauaagg......................   47       0     S09
    ......................................................................uggacggagaacugauaaggg.....................   565      0     S09
    ......................................................................uggacggagaacugauaagggc....................   5627     0     S09
    ......................................................................uggacggagaacugauaagggcu...................   1076     0     S09
    ......................................................................uggacggagaacugauaagggcuu..................   100      0     S09
```

```
                                  Star                             Mature
gaagaacuacagaugaagaaaaguuuccacgucuccuuaucauucgacuguccagacuguauuugcgaacuggacggagaacugauaagggcuuguggcgacauaaaucauc
.......................................................................ggacggagaacugauaagggc....................    1     0     S09
.......................................................................ggacggagaacugauaagggcu...................    4     0     S09
.......................................................................ggacggagaacugauaagggcuu..................    2     0     S09
........................................................................gacggagaacugauaagggcuu..................    2     0     S09

..................................ccuuaucauucgacugucca..........................................................    1     0     S03
..................................ccuuaucauucgacuguccag.........................................................    10    0     S03
..................................ccuuaucauucgacuguccaga........................................................    102   0     S03
..................................ccuuaucauucgacuguccagacug.....................................................    1     0     S03
................................................uguccagacuguauuugcgaac..........................................    2     0     S03
......................................................................uggacggagaacugauaa........................    2     0     S03
......................................................................uggacggagaacugauaag.......................    1     0     S03
......................................................................uggacggagaacugauaagg......................    13    0     S03
......................................................................uggacggagaacugauaaggg.....................    30    0     S03
......................................................................uggacggagaacugauaagggc....................    1558  0     S03
......................................................................uggacggagaacugauaagggcu...................    249   0     S03
......................................................................uggacggagaacugauaagggcuu..................    30    0     S03
.......................................................................ggacggagaacugauaagggc....................    2     0     S03
.......................................................................ggacggagaacugauaagggcu...................    5     0     S03
.......................................................................ggacggagaacugauaagggcuu..................    1     0     S03
........................................................................gacggagaacugauaagggcuu..................    2     0     S03

...............................ucuccuuaucauucgacugucc...........................................................    2     0     S08
.................................uccuuaucauucgacuguccaga........................................................    1     0     S08
..................................ccuuaucauucgacuguccag.........................................................    65    0     S08
..................................ccuuaucauucgacuguccaga........................................................    241   0     S08
..................................ccuuaucauucgacuguccagac.......................................................    5     0     S08
..................................................................gaacuggacggagaacugauaagggc....................    3     0     S08
......................................................................uggacggagaacugauaa........................    1     0     S08
......................................................................uggacggagaacugauaag.......................    2     0     S08
......................................................................uggacggagaacugauaagg......................    21    0     S08
......................................................................uggacggagaacugauaaggg.....................    164   0     S08
......................................................................uggacggagaacugauaagggc....................    3915  0     S08
......................................................................uggacggagaacugauaagggcu...................    674   0     S08
......................................................................uggacggagaacugauaagggcuu..................    57    0     S08
......................................................................uggacggagaacugauaagggcuug.................    1     0     S08
.......................................................................ggacggagaacugauaagggc....................    1     0     S08
.......................................................................ggacggagaacugauaagggcu...................    5     0     S08
........................................................................gacggagaacugauaagggc....................    1     0     S08
........................................................................gacggagaacugauaagggcuu..................    2     0     S08
.........................................................................acggagaacugauaagggc....................    1     0     S08
..........................................................................cggagaacugauaagggc....................    2     0     S08
...........................................................................ggagaacugauaagggcu...................    1     0     S08

..................................ccuuaucauucgacuguccaga........................................................    6     0     S01
..................................ccuuaucauucgacuguccagac.......................................................    1     0     S01
......................................................................uggacggagaacugauaa........................    1     0     S01
......................................................................uggacggagaacugauaag.......................    1     0     S01
......................................................................uggacggagaacugauaagg......................    3     0     S01
......................................................................uggacggagaacugauaaggg.....................    21    0     S01
......................................................................uggacggagaacugauaagggc....................    106   0     S01
......................................................................uggacggagaacugauaagggcu...................    15    0     S01
......................................................................uggacggagaacugauaagggcuu..................    1     0     S01
........................................................................gacggagaacugauaagggcuu..................    1     0     S01

.................................uccuuaucauucgacuguccaga........................................................    1     0     S06
..................................ccuuaucauucgacuguccag.........................................................    34    0     S06
..................................ccuuaucauucgacuguccaga........................................................    180   0     S06
..................................ccuuaucauucgacuguccagacuguauuugcg.............................................    1     0     S06
..................................ccuuaucauucgacuguccagacuguauuugcgaac..........................................    1     0     S06
..................................................................gaacuggacggagaacugauaagggc....................    1     0     S06
......................................................................uggacggagaacugauaa........................    2     0     S06
......................................................................uggacggagaacugauaag.......................    2     0     S06
......................................................................uggacggagaacugauaagg......................    4     0     S06
......................................................................uggacggagaacugauaaggg.....................    75    0     S06
......................................................................uggacggagaacugauaagggc....................    2187  0     S06
......................................................................uggacggagaacugauaagggcu...................    496   0     S06
......................................................................uggacggagaacugauaagggcuu..................    91    0     S06
.......................................................................ggacggagaacugauaagggcuu..................    1     0     S06
........................................................................gacggagaacugauaagggcuu..................    2     0     S06
```

```
                                  Star                                Mature
gaagaacuacagaugaagaaaaguuuccacgucuccuuaucauucgacuguccagacuguauuugcgaacuggacggagaacugauaagggcuuguggcgacauaaaucauc
.........................................................................acggagaacugauaagggcuu..................    2       0    S06

..................................ccuuaucauucgacugucca..........................................................    1       0    S07
..................................ccuuaucauucgacuguccag.........................................................    31      0    S07
..................................ccuuaucauucgacuguccaga........................................................    295     0    S07
..................................ccuuaucauucgacuguccagac.......................................................    3       0    S07
...............................................................ugcgaacuggacggagaacugauaagggc....................    1       0    S07
..................................................................gaacuggacggagaacugauaagggc....................    1       0    S07
......................................................................uggacggagaacugauaa........................    2       0    S07
......................................................................uggacggagaacugauaag.......................    7       0    S07
......................................................................uggacggagaacugauaagg......................    27      0    S07
......................................................................uggacggagaacugauaaggg.....................    422     0    S07
......................................................................uggacggagaacugauaagggc....................    8494    0    S07
......................................................................uggacggagaacugauaagggcu...................    1667    0    S07
......................................................................uggacggagaacugauaagggcuu..................    196     0    S07
.......................................................................ggacggagaacugauaagggc....................    5       0    S07
.......................................................................ggacggagaacugauaagggcu...................    8       0    S07
.......................................................................ggacggagaacugauaagggcuu..................    7       0    S07
........................................................................gacggagaacugauaagggcuu..................    2       0    S07
...........................................................................ggagaacugauaagggcu...................    1       0    S07

...............................ucuccuuaucauucgacugucc...........................................................    1       0    S05
.................................uccuuaucauucgacuguccaga........................................................    2       0    S05
..................................ccuuaucauucgacuguccag.........................................................    20      0    S05
..................................ccuuaucauucgacuguccaga........................................................    138     0    S05
..................................ccuuaucauucgacuguccagacuguauuugcgaac..........................................    1       0    S05
.....................................uaucauucgacuguccaga........................................................    1       0    S05
......................................................................uggacggagaacugauaaggg.....................    17      0    S05
......................................................................uggacggagaacugauaagggc....................    1209    0    S05
......................................................................uggacggagaacugauaagggcu...................    154     0    S05
......................................................................uggacggagaacugauaagggcuu..................    11      0    S05
.......................................................................ggacggagaacugauaagggc....................    1       0    S05
.......................................................................ggacggagaacugauaagggcuu..................    1       0    S05
........................................................................gacggagaacugauaagggcuu..................    2       0    S05

.................................uccuuaucauucgacuguccaga........................................................    2       0    S10
..................................ccuuaucauucgacugucc...........................................................    1       0    S10
..................................ccuuaucauucgacuguccag.........................................................    14      0    S10
..................................ccuuaucauucgacuguccaga........................................................    181     0    S10
..................................ccuuaucauucgacuguccagac.......................................................    1       0    S10
.....................................uaucauucgacuguccaga........................................................    1       0    S10
.....................................................................cuggacggagaacugauaagggc....................    4       0    S10
......................................................................uggacggagaacugauaa........................    1       0    S10
......................................................................uggacggagaacugauaag.......................    2       0    S10
......................................................................uggacggagaacugauaagg......................    59      0    S10
......................................................................uggacggagaacugauaaggg.....................    953     0    S10
......................................................................uggacggagaacugauaagggc....................    8029    0    S10
......................................................................uggacggagaacugauaagggcu...................    1323    0    S10
......................................................................uggacggagaacugauaagggcuu..................    136     0    S10
.......................................................................ggacggagaacugauaagggc....................    1       0    S10
.......................................................................ggacggagaacugauaagggcu...................    7       0    S10
.......................................................................ggacggagaacugauaagggcuu..................    1       0    S10
........................................................................gacggagaacugauaagggcuu..................    2       0    S10
.........................................................................acggagaacugauaagggc....................    1       0    S10
.........................................................................acggagaacugauaagggcu...................    1       0    S10
```

Provisional ID : Scaffold390_9906
Score total : 18848
Score for star read(s) : 3.9
Score for read counts : 18840.7
Score for mfe : 1.8
Score for randfold : 1.6
Score for cons. seed :
Total read count : 36967
Mature read count : 36924
Loop read count : 14
Star read count : 29

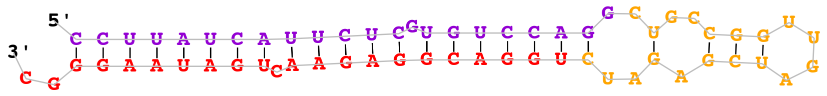

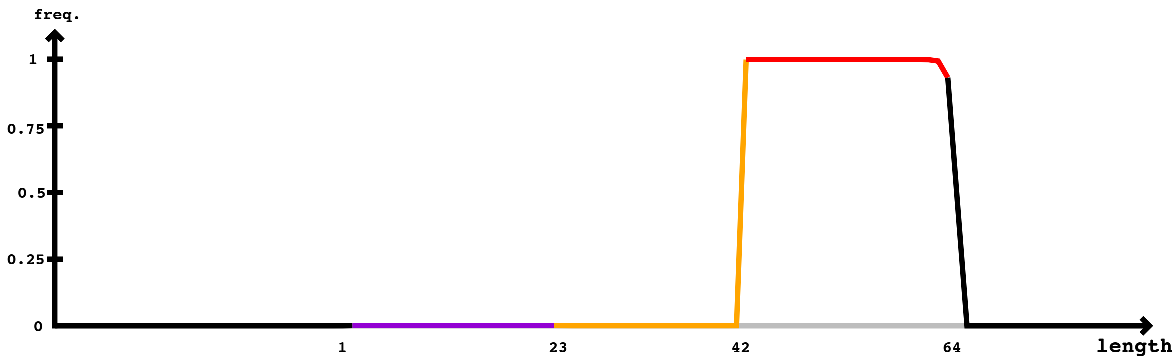

Star   Mature

```
5'- gugagauaagaagauaagucuccucgcauccuuaucauucucguguccaggcugccgguugaucgagaucuggacggagaacugauaagggcacguggaagacgauggcauc  -3'   obs
    gugagauaagaagauaagucuccucgcauccuuaucauucucguguccaggcugccgguugaucgagaucuggacggagaacugauaagggcacguggaagacgauggcauc        exp
    .................((((((.((..(((((((((((((.((((((((..(..(((....))).)..)))))))))))).)))))))))..)).)).))))........   reads   mm   sample
    ............................uccuuaucauucucguguccagg.............................................................   1       0    S10
    .............................ccuuaucauucucguguccagg.............................................................   2       0    S10
    ...................................................cugccgguugaucgagauc..........................................   5       0    S10
    .....................................................................cuggacggagaacugauaagggc....................   4       0    S10
    ......................................................................uggacggagaacugauaa........................   1       0    S10
    ......................................................................uggacggagaacugauaag.......................   2       0    S10
    ......................................................................uggacggagaacugauaagg......................   59      0    S10
    ......................................................................uggacggagaacugauaaggg.....................   953     0    S10
    ......................................................................uggacggagaacugauaagggc....................   8029    0    S10
    ......................................................................uggacggagaacugauaagggca...................   269     0    S10
    ........................................................................ggacggagaacugauaagggc....................   1       0    S10
    .........................................................................acggagaacugauaagggc....................   1       0    S10

    ...................................................cugccgguugaucgagauc..........................................   1       0    S05
    ......................................................................uggacggagaacugauaaggg.....................   17      0    S05
    ......................................................................uggacggagaacugauaagggc....................   1209    0    S05
    ......................................................................uggacggagaacugauaagggca...................   18      0    S05
    ........................................................................ggacggagaacugauaagggc....................   1       0    S05

    .............................ccuuaucauucucguguccagg.............................................................   3       0    S07
    .............................ccuuaucauucucguguccaggcu...........................................................   1       0    S07
    ......................................................................uggacggagaacugauaa........................   2       0    S07
    ......................................................................uggacggagaacugauaag.......................   7       0    S07
    ......................................................................uggacggagaacugauaagg......................   27      0    S07
    ......................................................................uggacggagaacugauaaggg.....................   422     0    S07
    ......................................................................uggacggagaacugauaagggc....................   8494    0    S07
    ......................................................................uggacggagaacugauaagggca...................   256     0    S07
    ......................................................................uggacggagaacugauaagggcac..................   1       0    S07
    ........................................................................ggacggagaacugauaagggc....................   5       0    S07

    ......................................................................uggacggagaacugauaa........................   1       0    S01
    ......................................................................uggacggagaacugauaag.......................   1       0    S01
    ......................................................................uggacggagaacugauaagg......................   3       0    S01
```

Star **Mature**

```
gugagauaagaagauaagucuccucgcauccuuaucauucucguguccaggcugccgguugaucgagaucuggacggagaacugauaagggcacguggaagacgauggcauc
......................................................................uggacggagaacugauaaggg.....................  21  0  S01
......................................................................uggacggagaacugauaagggc....................  106  0  S01
......................................................................uggacggagaacugauaagggca...................  6  0  S01

............................uccuuaucauucucguguccagg.............................................................  1  0  S06
......................................................................uggacggagaacugauaa........................  2  0  S06
......................................................................uggacggagaacugauaag.......................  2  0  S06
......................................................................uggacggagaacugauaagg......................  4  0  S06
......................................................................uggacggagaacugauaaggg.....................  75  0  S06
......................................................................uggacggagaacugauaagggc....................  2187  0  S06
......................................................................uggacggagaacugauaagggca...................  53  0  S06

............................uccuuaucauucucguguccagg.............................................................  2  0  S08
.............................ccuuaucauucucguguccagg.............................................................  14  0  S08
...................................................cugccgguugaucgagauc..........................................  7  0  S08
......................................................................uggacggagaacugauaa........................  1  0  S08
......................................................................uggacggagaacugauaag.......................  2  0  S08
......................................................................uggacggagaacugauaagg......................  21  0  S08
......................................................................uggacggagaacugauaaggg.....................  164  0  S08
......................................................................uggacggagaacugauaagggc....................  3915  0  S08
......................................................................uggacggagaacugauaagggca...................  163  0  S08
.......................................................................ggacggagaacugauaagggc....................  1  0  S08
.......................................................................ggacggagaacugauaagggca...................  1  0  S08
........................................................................gacggagaacugauaagggc....................  1  0  S08
.........................................................................acggagaacugauaagggc....................  1  0  S08
..........................................................................cggagaacugauaagggc....................  2  0  S08

...................................................cugccgguugaucgagauc..........................................  1  0  S03
......................................................................uggacggagaacugauaa........................  2  0  S03
......................................................................uggacggagaacugauaag.......................  1  0  S03
......................................................................uggacggagaacugauaagg......................  13  0  S03
......................................................................uggacggagaacugauaaggg.....................  30  0  S03
......................................................................uggacggagaacugauaagggc....................  1558  0  S03
......................................................................uggacggagaacugauaagggca...................  17  0  S03
.......................................................................ggacggagaacugauaagggc....................  2  0  S03

.............................ccuuaucauucucguguccagg.............................................................  4  0  S09
.....................................................................cuggacggagaacugauaagggc....................  1  0  S09
......................................................................uggacggagaacugauaa........................  2  0  S09
......................................................................uggacggagaacugauaag.......................  2  0  S09
......................................................................uggacggagaacugauaagg......................  47  0  S09
......................................................................uggacggagaacugauaaggg.....................  565  0  S09
......................................................................uggacggagaacugauaagggc....................  5627  0  S09
......................................................................uggacggagaacugauaagggca...................  222  0  S09
.......................................................................ggacggagaacugauaagggc....................  1  0  S09

......................................................................uggacggagaacugauaag.......................  1  0  S04
......................................................................uggacggagaacugauaagg......................  4  0  S04
......................................................................uggacggagaacugauaaggg.....................  39  0  S04
......................................................................uggacggagaacugauaagggc....................  2119  0  S04
......................................................................uggacggagaacugauaagggca...................  35  0  S04

.............................ccuuaucauucucgugucca...............................................................  1  0  S02
......................................................................uggacggagaacugauaa........................  5  0  S02
......................................................................uggacggagaacugauaag.......................  2  0  S02
......................................................................uggacggagaacugauaagg......................  4  0  S02
......................................................................uggacggagaacugauaaggg.....................  20  0  S02
......................................................................uggacggagaacugauaagggc....................  91  0  S02
......................................................................uggacggagaacugauaagggca...................  3  0  S02
```

Provisional ID : Scaffold2173_33652
Score total : 3481.6
Score for star read(s) : 3.9
Score for read counts : 3474.1
Score for mfe : 2
Score for randfold : 1.6
Score for cons. seed :
Total read count : 6826
Mature read count : 6577
Loop read count : 0
Star read count : 249

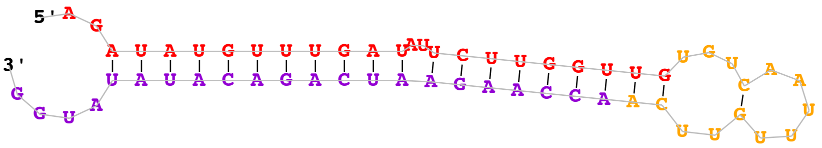

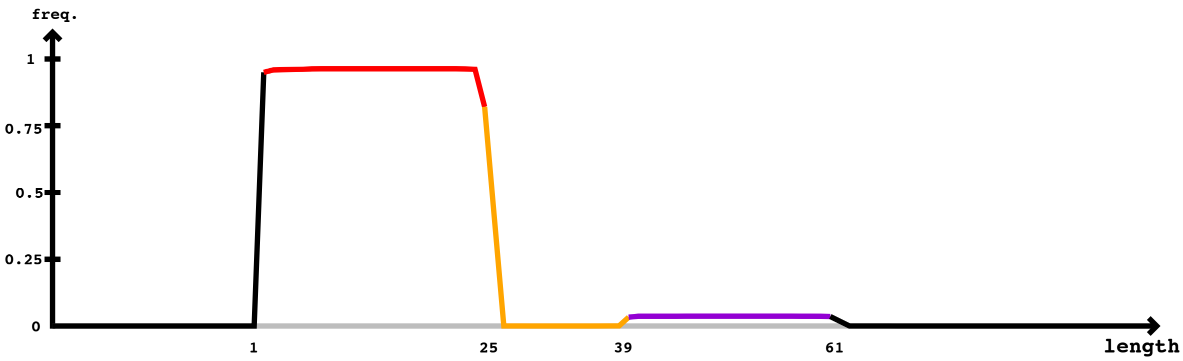

Mature **Star**

```
5'- aagacauagccauggcugucagauauguuugauauucuugguugugucaauuuguucaaccaagaaucagacauauauggcagacauggccuuuccucuggacaaacuuaugg   -3'   obs
    aagacauagccauggcugucagauauguuugauauucuugguugugucaauuuguucaaccaagaaucagacauauauggcagacauggccuuuccucuggacaaacuuaugg         exp
    ....((((((((((.((((((.(((((((((((..(((((((((...(.....)..)))))))))))))))))))).)))))).))))))...(((...)))......)))).   reads   mm   sample
    ....................agauauguuugauauucuugg........................................................................   1       0    S10
    ....................agauauguuugauauucuuggu.......................................................................   4       0    S10
    ....................agauauguuugauauucuugguu......................................................................   216     0    S10
    ....................agauauguuugauauucuugguug.....................................................................   1498    0    S10
    ....................agauauguuugauauucuugguugu....................................................................   12      0    S10
    .....................gauauguuugauauucuugguu......................................................................   3       0    S10
    .....................gauauguuugauauucuugguug.....................................................................   15      0    S10
    ......................auauguuugauauucuugguug.....................................................................   1       0    S10
    .......................uauguuugauauucuugguug.....................................................................   1       0    S10
    ........................auguuugauauucuugguug.....................................................................   2       0    S10
    .........................uguuugauauucuugguug.....................................................................   2       0    S10
    ........................................................caaccaagaaucagacauauaug..................................   1       0    S10
    ..........................................................accaagaaucagacauauaug..................................   1       0    S10
    ..........................................................accaagaaucagacauauaugg.................................   66      0    S10
    ...........................................................ccaagaaucagacauauaugg.................................   4       0    S10
    ................................................................aaucagacauauauggcagaca...........................   1       0    S10

    ....................agauauguuugauauucuugguu......................................................................   68      0    S05
    ....................agauauguuugauauucuugguug.....................................................................   223     0    S05
    ....................agauauguuugauauucuugguugu....................................................................   3       0    S05
    .....................gauauguuugauauucuugguu......................................................................   1       0    S05
    .....................gauauguuugauauucuugguug.....................................................................   3       0    S05
    ..........................................................accaagaaucagacauauaugg.................................   3       0    S05

    ....................agauauguuugauauucuuggu.......................................................................   2       0    S07
    ....................agauauguuugauauucuugguu......................................................................   84      0    S07
    ....................agauauguuugauauucuugguug.....................................................................   562     0    S07
    ....................agauauguuugauauucuugguugu....................................................................   4       0    S07
    .....................gauauguuugauauucuugguug.....................................................................   7       0    S07
    ........................auguuugauauucuugguu......................................................................   1       0    S07
    ..........................................................accaagaaucagacauauaugg.................................   17      0    S07
    ...........................................................ccaagaaucagacauauaugg.................................   5       0    S07

    ....................agauauguuugauauucuugguu......................................................................   68      0    S06
```

Mature **Star**

```
aagacauagccauggcugucagauauguuugauauucuugguugugucaauuuguucaaccaagaaucagacauauauggcagacauggccuuuccucuggacaaacuuaugg
....................agauauguuugauauucuugguug.....................................................................  216   0   S06
....................agauauguuugauauucuugguugu....................................................................  3     0   S06
.....................gauauguuugauauucuugguu......................................................................  1     0   S06
.....................gauauguuugauauucuugguug.....................................................................  1     0   S06
..........................................................accaagaaucagacauauaugg.................................  1     0   S06
...........................................................ccaagaaucagacauauaug..................................  2     0   S06
...........................................................ccaagaaucagacauauaugg.................................  1     0   S06

....................agauauguuugauauucuugguu......................................................................  58    0   S01
....................agauauguuugauauucuugguug.....................................................................  132   0   S01
....................agauauguuugauauucuugguugu....................................................................  1     0   S01
.....................gauauguuugauauucuugguu......................................................................  2     0   S01
.....................gauauguuugauauucuugguug.....................................................................  1     0   S01
..........................guuugauauucuugguug.....................................................................  2     0   S01
...........................................................ccaagaaucagacauauaugg.................................  1     0   S01

....................agauauguuugauauucuugguu......................................................................  14    0   S08
....................agauauguuugauauucuugguug.....................................................................  89    0   S08
.....................gauauguuugauauucuugguug.....................................................................  2     0   S08
......................auauguuugauauucuugguug.....................................................................  1     0   S08
.......................uauguuugauauucuugguug.....................................................................  1     0   S08
..........................................................accaagaaucagacauauaugg.................................  38    0   S08
...........................................................ccaagaaucagacauauaugg.................................  4     0   S08

....................agauauguuugauauucuugg........................................................................  2     0   S09
....................agauauguuugauauucuuggu.......................................................................  1     0   S09
....................agauauguuugauauucuugguu......................................................................  206   0   S09
....................agauauguuugauauucuugguug.....................................................................  2081  0   S09
....................agauauguuugauauucuugguugu....................................................................  1     0   S09
....................agauauguuugauauucuugguuguguc.................................................................  1     0   S09
.....................gauauguuugauauucuugguug.....................................................................  21    0   S09
......................auauguuugauauucuugguug.....................................................................  3     0   S09
.......................uauguuugauauucuugguug.....................................................................  1     0   S09
.........................uguuugauauucuugguug.....................................................................  9     0   S09
..........................................................accaagaaucagacauauaugg.................................  92    0   S09
...........................................................ccaagaaucagacauauaugg.................................  6     0   S09

....................agauauguuugauauucuuggu.......................................................................  1     0   S03
....................agauauguuugauauucuugguu......................................................................  62    0   S03
....................agauauguuugauauucuugguug.....................................................................  221   0   S03
....................agauauguuugauauucuugguugu....................................................................  2     0   S03
.....................gauauguuugauauucuugguug.....................................................................  1     0   S03
......................auauguuugauauucuugguug.....................................................................  1     0   S03
.......................uauguuugauauucuugguug.....................................................................  2     0   S03
........................auguuugauauucuugguu......................................................................  1     0   S03
.......................................................ucaaccaagaaucagacauau.....................................  1     0   S03
.........................................................aaccaagaaucagacauauau...................................  1     0   S03
...........................................................ccaagaaucagacauauaug..................................  1     0   S03

....................agauauguuugauauucuugg........................................................................  1     0   S04
....................agauauguuugauauucuuggu.......................................................................  1     0   S04
....................agauauguuugauauucuugguu......................................................................  69    0   S04
....................agauauguuugauauucuugguug.....................................................................  237   0   S04
....................agauauguuugauauucuugguugu....................................................................  2     0   S04
.....................gauauguuugauauucuugguug.....................................................................  2     0   S04
.........................uguuugauauucuugguug.....................................................................  1     0   S04
..........................................................accaagaaucagacauauaug..................................  1     0   S04
..........................................................accaagaaucagacauauaugg.................................  2     0   S04
...........................................................ccaagaaucagacauauaugg.................................  1     0   S04

....................agauauguuugauauucuuggu.......................................................................  1     0   S02
....................agauauguuugauauucuugguu......................................................................  104   0   S02
....................agauauguuugauauucuugguug.....................................................................  230   0   S02
....................agauauguuugauauucuugguugu....................................................................  5     0   S02
.....................gauauguuugauauucuugguug.....................................................................  2     0   S02
..........................................................accaagaaucagacauauaugg.................................  1     0   S02
```

Provisional ID	:	Scaffold708_16399
Score total	:	162.3
Score for star read(s)	:	3.9
Score for read counts	:	155.1
Score for mfe	:	1.6
Score for randfold	:	1.6
Score for cons. seed	:	
Total read count	:	316
Mature read count	:	314
Loop read count	:	0
Star read count	:	2

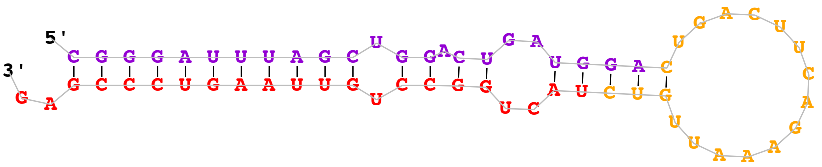

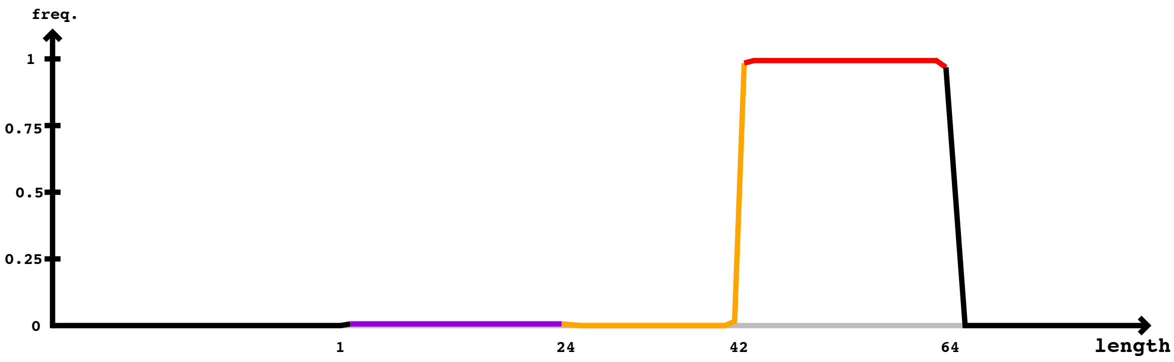


```
                                 Star                                        Mature
5'- uagguaucucugaaauggaagacugcacccgggauuuagcuggacugauggacugacuucagaaauugucuacuggccuguuaagucccgagugccgcucucacacuacccc  -3'  obs
    uagguaucucugaaauggaagacugcacccgggauuuagcuggacugauggacugacuucagaaauugucuacuggccuguuaagucccgagugccgcucucacacuacccc       exp
    ..((((....((...((..((.(.((((.((((((((((.((.((..(((((..............)))))..)))).)))))))))).)))).)))..)))).))))..   reads  mm  sample
    ......................................................................uacuggccuguuaagucccga.....................   1      0   S08
    ......................................................................uacuggccuguuaagucccgag....................   58     0   S08
    ......................................................................uacuggccuguuaagucccgagu...................   3      0   S08

    .....................................................................cuacuggccuguuaagucccga.....................   1      0   S09
    ......................................................................uacuggccuguuaagucccgag....................   19     0   S09
    ......................................................................uacuggccuguuaagucccgagu...................   1      0   S09

    ......................................................................uacuggccuguuaagucccga.....................   1      0   S03
    ......................................................................uacuggccuguuaagucccgag....................   29     0   S03
    .......................................................................acuggccuguuaagucccgagu...................   1      0   S03

    .............................cgggauuuagcuggacugaugga............................................................   1      0   S02
    ......................................................................uacuggccuguuaagucccgag....................   11     0   S02
    ......................................................................uacuggccuguuaagucccgagu...................   1      0   S02

    ......................................................................uacuggccuguuaagucccga.....................   1      0   S04
    ......................................................................uacuggccuguuaagucccgag....................   48     0   S04
    ......................................................................uacuggccuguuaagucccgagu...................   3      0   S04
    .......................................................................acuggccuguuaagucccgagu...................   1      0   S04

    .............................cgggauuuagcuggacugaugga............................................................   1      0   S10
    ......................................................................uacuggccuguuaagucccgag....................   17     0   S10
    ......................................................................uacuggccuguuaagucccgagu...................   2      0   S10

    .....................................................................cuacuggccuguuaagucccga.....................   3      0   S05
    ......................................................................uacuggccuguuaagucccgag....................   41     0   S05

    .....................................................................cuacuggccuguuaagucccga.....................   1      0   S07
    ......................................................................uacuggccuguuaagucccgag....................   42     0   S07
    ......................................................................uacuggccuguuaagucccgagu...................   2      0   S07

    ......................................................................uacuggccuguuaagucccgag....................   21     0   S06
```

```
                                Star                                        Mature
uagguaucucugaaauggaagacugcacccgggauuuagcuggacugauggacugacuucagaaauugucuacuggccuguuaagucccgagugccgcucucacacuacccc
.....................................................................uacuggccuguuaagucccgagu...................    3    0    S06
......................................................................acuggccuguuaagucccgagu...................    1    0    S06

.....................................................................uacuggccuguuaagucccgag....................    2    0    S01
```

Provisional ID	:	Scaffold171_5150
Score total	:	1.6
Score for star read(s)	:	-1.3
Score for read counts	:	0
Score for mfe	:	1.3
Score for randfold	:	1.6
Score for cons. seed	:	
Total read count	:	33
Mature read count	:	30
Loop read count	:	0
Star read count	:	3

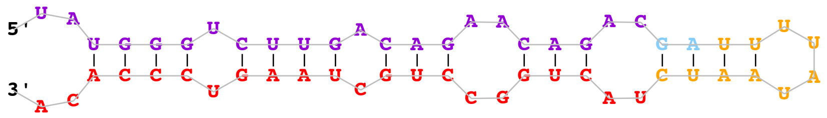

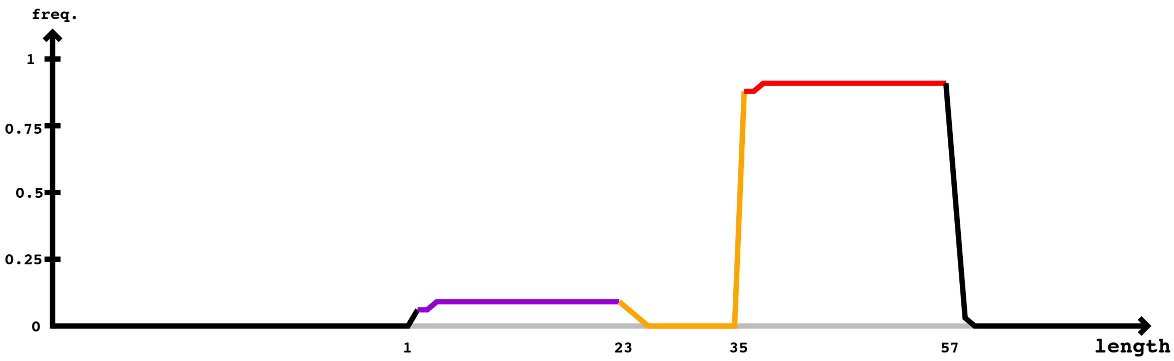


```
                                    Star                                 Mature
5'- gacacacagaagacuucaggaaugaaggugcuucguuaugggucuugacagaacagacgauuuuauaaaucuacuggccugcuaagucccacaacgcgguucuuucaauugca  -3'   obs
    gacacacagaagacuucaggaaugaaggugcuucguuaugggucuugacagaacagacgauuuuauaaaucuacuggccugcuaagucccacaacgcgguucuuucaauugca         exp
    ..................(.((((((((.(((.((((.((((.((((.(((..(((..((((....))))..)))..))).)))).)))).)))).))).)))))).))).).   reads   mm   sample
    ......................................................................uacuggccugcuaagucccaca.....................   3       0    S07
    ........................................................................cuggccugcuaagucccacaac...................   1       0    S07

    ......................................................................uacuggccugcuaagucccaca.....................   2       0    S08

    ....................................uaugggucuugacagaacagac.......................................................   2       0    S10
    ......................................ugggucuugacagaacagacga.....................................................   1       0    S10
    ......................................................................uacuggccugcuaagucccaca.....................   17      0    S10

    ......................................................................uacuggccugcuaagucccaca.....................   7       0    S09
```

Provisional ID	:	Scaffold22_817
Score total	:	855.2
Score for star read(s)	:	3.9
Score for read counts	:	848.5
Score for mfe	:	1.3
Score for randfold	:	1.6
Score for cons. seed	:	
Total read count	:	1676
Mature read count	:	1212
Loop read count	:	0
Star read count	:	464

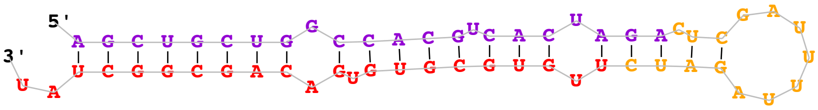

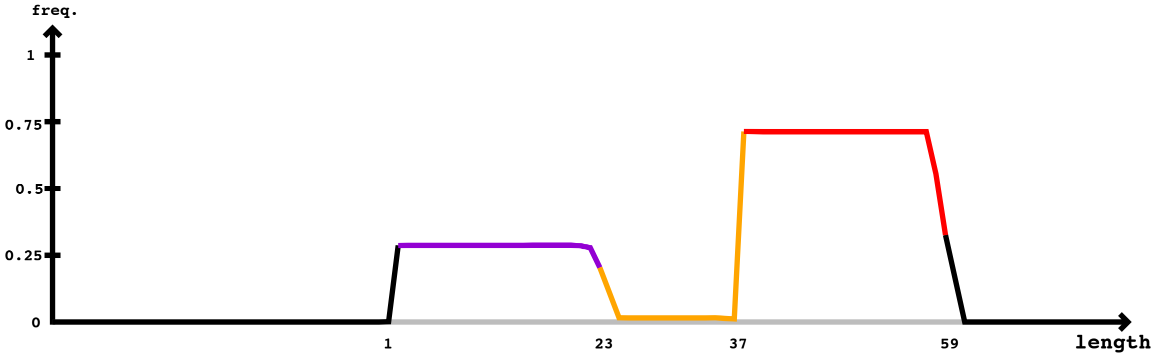

Star **Mature**

```
5'- cagaaaagaucgauggaugcuugcauuuaaaaguagcugcuggccacgucacuagacucgauuuuagaucuugugcgugugacagcggcuaucgucauaugcugaaugcu  -3'   obs
    cagaaaagaucgauggaugcuugcauuuaaaaguagcugcuggccacgucacuagacucgauuuuagaucuugugcgugugacagcggcuaucgucauaugcugaaugcu         exp
    (((....((.((.((((((....))))))...((((((((((.(((((.(((.(((.((.......))))).))))))).).)))))))))))))).....)))......   reads   mm   sample
    ..................................agcugcuggccacgucacu.........................................................   1       0    S10
    ..................................agcugcuggccacgucacua........................................................   5       0    S10
    ..................................agcugcuggccacgucacuag.......................................................   28      0    S10
    ..................................agcugcuggccacgucacuaga......................................................   99      0    S10
    ..................................agcugcuggccacgucacuagac.....................................................   2       0    S10
    ..................................agcugcuggccacgucacuagacucgauuuuagauc........................................   2       0    S10
    ..................................agcugcuggccacgucacuagacucgauuuuagaucu.......................................   1       0    S10
    ..................................agcugcuggccacgucacuagacucgauuuuagaucuu......................................   1       0    S10
    ...................................................................aucuugugcgugugacagcggcua...................   1       0    S10
    ......................................................................uugugcgugugacagcggcu....................   111     0    S10
    ......................................................................uugugcgugugacagcggcua...................   140     0    S10
    ......................................................................uugugcgugugacagcggcuau..................   235     0    S10

    .................................uagcugcuggccacgucacuaga......................................................   1       0    S05
    ..................................agcugcuggccacgucacuag.......................................................   2       0    S05
    ..................................agcugcuggccacgucacuaga......................................................   3       0    S05
    ...................................gcugcuggccacgucacuagac.....................................................   1       0    S05
    ......................................................................uugugcgugugacagcggcu....................   8       0    S05
    ......................................................................uugugcgugugacagcggcua...................   11      0    S05

    ..................................agcugcuggccacgucacua........................................................   1       0    S07
    ..................................agcugcuggccacgucacuag.......................................................   14      0    S07
    ..................................agcugcuggccacgucacuaga......................................................   32      0    S07
    ..................................agcugcuggccacgucacuagacucgauuuuaga..........................................   3       0    S07
    ......................................................................uugugcgugugacagcggcu....................   27      0    S07
    ......................................................................uugugcgugugacagcggcua...................   42      0    S07
    ......................................................................uugugcgugugacagcggcuau..................   47      0    S07

    ..................................agcugcuggccacgucacuaga......................................................   1       0    S01
    ................................................ucacuagacucgauuuuagauc........................................   1       0    S01
    ......................................................................uugugcgugugacagcggcu....................   5       0    S01
    ......................................................................uugugcgugugacagcggcuau..................   1       0    S01

    ..................................agcugcuggccacgucacua........................................................   3       0    S06
```

```
                                  Star                              Mature
cagaaaagaucgauggaugcuugcauuuaaaaguagcugcuggccacgucacuagacucgauuuuagaucuugugcgugugacagcggcuaucgucauaugcugaaugcu
..................................agcugcuggccacgucacuag.......................................................  23   0   S06
..................................agcugcuggccacgucacuaga......................................................  26   0   S06
..................................agcugcuggccacgucacuagac.....................................................  1    0   S06
..................................agcugcuggccacgucacuagacucgauuuuagauc........................................  4    0   S06
......................................................................uugugcgugugacagcggcu....................  11   0   S06
......................................................................uugugcgugugacagcggcua...................  17   0   S06
......................................................................uugugcgugugacagcggcuau..................  13   0   S06

..................................agcugcuggccacgucacu.........................................................  2    0   S08
..................................agcugcuggccacgucacua........................................................  3    0   S08
..................................agcugcuggccacgucacuag.......................................................  49   0   S08
..................................agcugcuggccacgucacuaga......................................................  91   0   S08
..................................agcugcuggccacgucacuagac.....................................................  3    0   S08
..................................agcugcuggccacgucacuagacu....................................................  1    0   S08
..................................agcugcuggccacgucacuagacucgauuuuaga...........................................  1    0   S08
..................................agcugcuggccacgucacuagacucgauuuuagau.........................................  3    0   S08
..................................agcugcuggccacgucacuagacucgauuuuagauc........................................  6    0   S08
......................................................................uugugcgugugacagcggcu....................  17   0   S08
......................................................................uugugcgugugacagcggcua...................  23   0   S08
......................................................................uugugcgugugacagcggcuau..................  21   0   S08
.......................................................................ugugcgugugacagcggcu....................  1    0   S08

..................................agcugcuggccacgucacuaga......................................................  1    0   S03
......................................................................uugugcgugugacagcggcu....................  15   0   S03
......................................................................uugugcgugugacagcggcua...................  7    0   S03
......................................................................uugugcgugugacagcggcuau..................  4    0   S03

..................................agcugcuggccacgucacu.........................................................  1    0   S09
..................................agcugcuggccacgucacuag.......................................................  10   0   S09
..................................agcugcuggccacgucacuaga......................................................  57   0   S09
..................................agcugcuggccacgucacuagacucgauuuuagauc........................................  2    0   S09
..................................agcugcuggccacgucacuagacucgauuuuagaucu.......................................  1    0   S09
......................................................................uugugcgugugacagcggcu....................  60   0   S09
......................................................................uugugcgugugacagcggcua...................  144  0   S09
......................................................................uugugcgugugacagcggcuau..................  220  0   S09
......................................................................uugugcgugugacagcggcuauc.................  3    0   S09

.................................uagcugcuggccacgucacuaga......................................................  1    0   S02
......................................................................uugugcgugugacagcggcu....................  8    0   S02
......................................................................uugugcgugugacagcggcua...................  3    0   S02
......................................................................uugugcgugugacagcggcuau..................  1    0   S02

..................................agcugcuggccacgucacuag.......................................................  1    0   S04
..................................agcugcuggccacgucacuaga......................................................  1    0   S04
..................................agcugcuggccacgucacuagacucgauuuuagauc........................................  1    0   S04
......................................................................uugugcgugugacagcggcu....................  5    0   S04
......................................................................uugugcgugugacagcggcua...................  3    0   S04
......................................................................uugugcgugugacagcggcuau..................  9    0   S04
```

Provisional ID	:	Scaffold133_4000
Score total	:	3547.8
Score for star read(s)	:	3.9
Score for read counts	:	3540.9
Score for mfe	:	1.5
Score for randfold	:	1.6
Score for cons. seed	:	
Total read count	:	6957
Mature read count	:	6882
Loop read count	:	0
Star read count	:	75

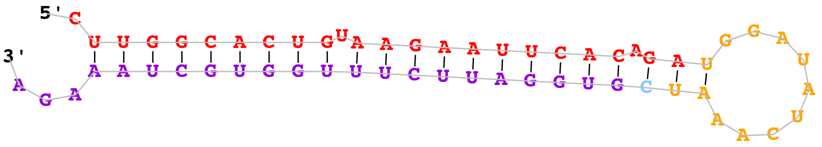

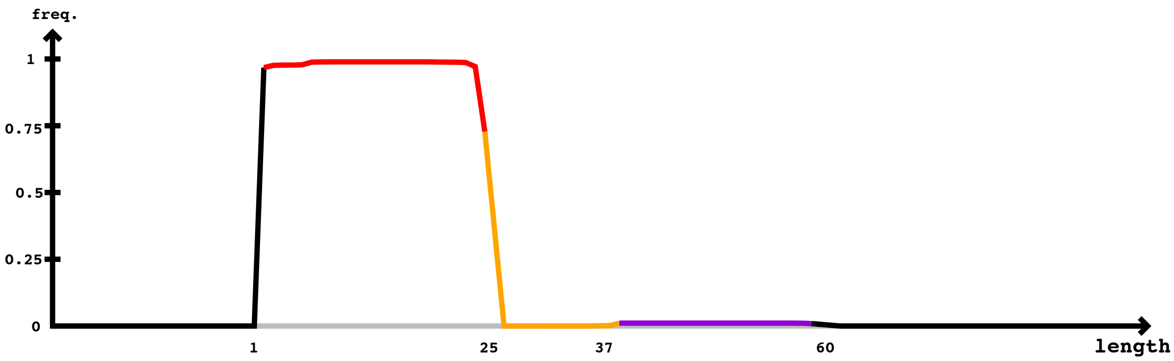

Mature Star

```
5'- caaauuguguuucuuuuaucuuggcacuguaagaauucacagauggauaucaaaucguggauucuuuggugcuaaagauaacaagaggcggcuucuucguggaaagauagu   -3'   obs
    caaauuguguuucuuuuaucuuggcacuguaagaauucacagauggauaucaaaucguggauucuuuggugcuaaagauaacaagaggcggcuucuucguggaaagauagu         exp
    ........((((((((((((.(((((((((.((((((((((.(((.........))))))))))))))))))))))).))))).))))))).(((((((......)))).)))   reads   mm   sample
    ....................cuuggcacuguaagaauuc.........................................................................   1       0    S02
    ....................cuuggcacuguaagaauuca........................................................................   1       0    S02
    ....................cuuggcacuguaagaauucacag.....................................................................   28      0    S02
    ....................cuuggcacuguaagaauucacaga....................................................................   39      0    S02
    ....................cuuggcacuguaagaauucacagau...................................................................   1       0    S02
    .........................cacuguaagaauucacag.....................................................................   1       0    S02
    .......................................................ucguggauucuuuggugcuaaa...................................   1       0    S02
    .........................................................guggauucuuuggugcuaaag..................................   2       0    S02
    .........................................................guggauucuuuggugcuaaaga.................................   1       0    S02

    ....................cuuggcacuguaagaauucaca......................................................................   3       0    S04
    ....................cuuggcacuguaagaauucacag.....................................................................   57      0    S04
    ....................cuuggcacuguaagaauucacaga....................................................................   157     0    S04
    ....................cuuggcacuguaagaauucacagau...................................................................   2       0    S04
    .....................uuggcacuguaagaauucacag.....................................................................   1       0    S04
    .....................uuggcacuguaagaauucacaga....................................................................   2       0    S04
    .........................cacuguaagaauucacag.....................................................................   2       0    S04
    .........................cacuguaagaauucacaga....................................................................   2       0    S04
    .......................................................ucguggauucuuuggugcuaaa...................................   1       0    S04
    .........................................................guggauucuuuggugcuaaa...................................   1       0    S04
    .........................................................guggauucuuuggugcuaaag..................................   2       0    S04
    .........................................................guggauucuuuggugcuaaaga.................................   6       0    S04

    ....................cuuggcacuguaagaauucac.......................................................................   1       0    S09
    ....................cuuggcacuguaagaauucaca......................................................................   36      0    S09
    ....................cuuggcacuguaagaauucacag.....................................................................   566     0    S09
    ....................cuuggcacuguaagaauucacaga....................................................................   2007    0    S09
    ....................cuuggcacuguaagaauucacagau...................................................................   24      0    S09
    .....................uuggcacuguaagaauucacag.....................................................................   5       0    S09
    .....................uuggcacuguaagaauucacaga....................................................................   15      0    S09
    ......................uggcacuguaagaauucac.......................................................................   1       0    S09
    .......................gcacuguaagaauucacag......................................................................   1       0    S09
    .......................gcacuguaagaauucacaga.....................................................................   1       0    S09
```

```
                    Mature                                 Star
caaauuguguuucuuuuauccuuggcacuguaagaauucacagauggauaucaaaucguggauucuuuggugcuaaagauaacaagaggcggcuucuucguggaaagauagu
.........................cacuguaagaauucacag.....................................................................   10    0   S09
.........................cacuguaagaauucacaga....................................................................   17    0   S09
.........................cacuguaagaauucacagau...................................................................   1     0   S09
..........................acuguaagaauucacaga....................................................................   3     0   S09
...........................cuguaagaauucacagau...................................................................   1     0   S09
........................................................cguggauucuuuggugcuaaaga.................................   1     0   S09
.........................................................guggauucuuuggugcuaa....................................   1     0   S09
.........................................................guggauucuuuggugcuaaa...................................   1     0   S09
.........................................................guggauucuuuggugcuaaag..................................   3     0   S09
.........................................................guggauucuuuggugcuaaaga.................................   17    0   S09

....................cuuggcacuguaagaauucac.......................................................................   2     0   S03
....................cuuggcacuguaagaauucaca......................................................................   3     0   S03
....................cuuggcacuguaagaauucacag.....................................................................   61    0   S03
....................cuuggcacuguaagaauucacaga....................................................................   159   0   S03
....................cuuggcacuguaagaauucacagau...................................................................   2     0   S03
....................cuuggcacuguaagaauucacagauggau...............................................................   1     0   S03
.....................uuggcacuguaagaauucacaga....................................................................   1     0   S03
......................uggcacuguaagaauucacag.....................................................................   1     0   S03
......................uggcacuguaagaauucacaga....................................................................   1     0   S03
.........................cacuguaagaauucacag.....................................................................   1     0   S03
.........................cacuguaagaauucacaga....................................................................   1     0   S03
.......................................................ucguggauucuuuggugcuaaa...................................   2     0   S03
.........................................................guggauucuuuggugcuaaa...................................   1     0   S03
.........................................................guggauucuuuggugcuaaaga.................................   1     0   S03

....................cuuggcacuguaagaauucaca......................................................................   7     0   S08
....................cuuggcacuguaagaauucacag.....................................................................   42    0   S08
....................cuuggcacuguaagaauucacaga....................................................................   127   0   S08
.....................uuggcacuguaagaauucacag.....................................................................   1     0   S08
.....................uuggcacuguaagaauucacaga....................................................................   2     0   S08
......................uggcacuguaagaauucaca......................................................................   1     0   S08
........................gcacuguaagaauucacaga....................................................................   2     0   S08
.........................cacuguaagaauucacag.....................................................................   1     0   S08
.........................cacuguaagaauucacaga....................................................................   8     0   S08
.........................cacuguaagaauucacagau...................................................................   1     0   S08
..........................acuguaagaauucacagau...................................................................   1     0   S08

....................cuuggcacuguaagaauu..........................................................................   2     0   S06
....................cuuggcacuguaagaauucac.......................................................................   1     0   S06
....................cuuggcacuguaagaauucaca......................................................................   5     0   S06
....................cuuggcacuguaagaauucacag.....................................................................   93    0   S06
....................cuuggcacuguaagaauucacaga....................................................................   112   0   S06
....................cuuggcacuguaagaauucacagau...................................................................   2     0   S06
.....................uuggcacuguaagaauucacag.....................................................................   1     0   S06
.....................uuggcacuguaagaauucacaga....................................................................   2     0   S06
......................uggcacuguaagaauucacag.....................................................................   1     0   S06
........................gcacuguaagaauucacag.....................................................................   1     0   S06
.........................cacuguaagaauucacag.....................................................................   1     0   S06
.........................cacuguaagaauucacaga....................................................................   3     0   S06
.........................................................guggauucuuuggugcuaaag..................................   1     0   S06
.........................................................guggauucuuuggugcuaaaga.................................   1     0   S06

....................cuuggcacuguaagaauuc.........................................................................   1     0   S01
....................cuuggcacuguaagaauucaca......................................................................   2     0   S01
....................cuuggcacuguaagaauucacag.....................................................................   15    0   S01
....................cuuggcacuguaagaauucacaga....................................................................   4     0   S01
.....................uuggcacuguaagaauucaca......................................................................   1     0   S01

....................cuuggcacuguaagaauucaca......................................................................   5     0   S07
....................cuuggcacuguaagaauucacag.....................................................................   86    0   S07
....................cuuggcacuguaagaauucacaga....................................................................   215   0   S07
....................cuuggcacuguaagaauucacagau...................................................................   5     0   S07
.....................uuggcacuguaagaauucacag.....................................................................   1     0   S07
.....................uuggcacuguaagaauucacaga....................................................................   4     0   S07
........................gcacuguaagaauucacaga....................................................................   1     0   S07
.........................cacuguaagaauucacag.....................................................................   1     0   S07
.........................cacuguaagaauucacaga....................................................................   3     0   S07
...........................cuguaagaauucacagau...................................................................   1     0   S07
.......................................................ucguggauucuuuggugcuaa....................................   1     0   S07
```

Mature Star

```
caaauuguguuucuuuuauccuuggcacuguaagaauucacagauggauaucaaaucguggauucuuuggugcuaaagauaacaagaggcggcuucuucguggaaagauagu
.........................................................guggauucuuuggugcuaaaga.................................	5	0	S07

....................cuuggcacuguaagaauu..........................................................................	1	0	S05
....................cuuggcacuguaagaauucac.......................................................................	1	0	S05
....................cuuggcacuguaagaauucaca......................................................................	6	0	S05
....................cuuggcacuguaagaauucacag.....................................................................	123	0	S05
....................cuuggcacuguaagaauucacaga....................................................................	190	0	S05
....................cuuggcacuguaagaauucacagau...................................................................	4	0	S05
.....................uuggcacuguaagaauucacag.....................................................................	1	0	S05
.....................uuggcacuguaagaauucacaga....................................................................	1	0	S05
......................uggcacuguaagaauucacaga....................................................................	1	0	S05
.........................cacuguaagaauucacag.....................................................................	1	0	S05
.......................................................ucguggauucuuuggugcuaaa...................................	1	0	S05

....................cuuggcacuguaagaauu..........................................................................	2	0	S10
....................cuuggcacuguaagaauuc.........................................................................	1	0	S10
....................cuuggcacuguaagaauuca........................................................................	1	0	S10
....................cuuggcacuguaagaauucac.......................................................................	1	0	S10
....................cuuggcacuguaagaauucaca......................................................................	37	0	S10
....................cuuggcacuguaagaauucacag.....................................................................	581	0	S10
....................cuuggcacuguaagaauucacaga....................................................................	1888	0	S10
....................cuuggcacuguaagaauucacagau...................................................................	22	0	S10
.....................uuggcacuguaagaauucacag.....................................................................	5	0	S10
.....................uuggcacuguaagaauucacaga....................................................................	15	0	S10
......................uggcacuguaagaauucacag.....................................................................	1	0	S10
........................gcacuguaagaauucacaga....................................................................	1	0	S10
........................gcacuguaagaauucacagau...................................................................	1	0	S10
.........................cacuguaagaauucacag.....................................................................	6	0	S10
.........................cacuguaagaauucacaga....................................................................	9	0	S10
...........................cuguaagaauucacagau...................................................................	1	0	S10
.......................................................ucguggauucuuuggugcuaaag..................................	1	0	S10
........................................................cguggauucuuuggugcuaaag..................................	1	0	S10
.........................................................guggauucuuuggugcuaaa...................................	2	0	S10
.........................................................guggauucuuuggugcuaaag..................................	5	0	S10
.........................................................guggauucuuuggugcuaaaga.................................	16	0	S10
```

Provisional ID	:	Scaffold133_3996
Score total	:	3216.5
Score for star read(s)	:	3.9
Score for read counts	:	3209
Score for mfe	:	2
Score for randfold	:	1.6
Score for cons. seed	:	
Total read count	:	6306
Mature read count	:	6299
Loop read count	:	0
Star read count	:	7

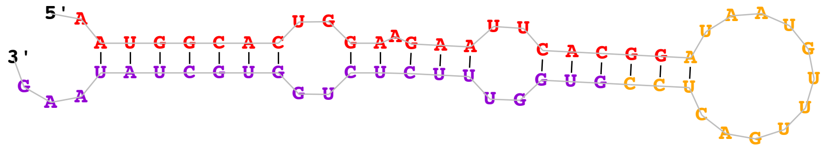

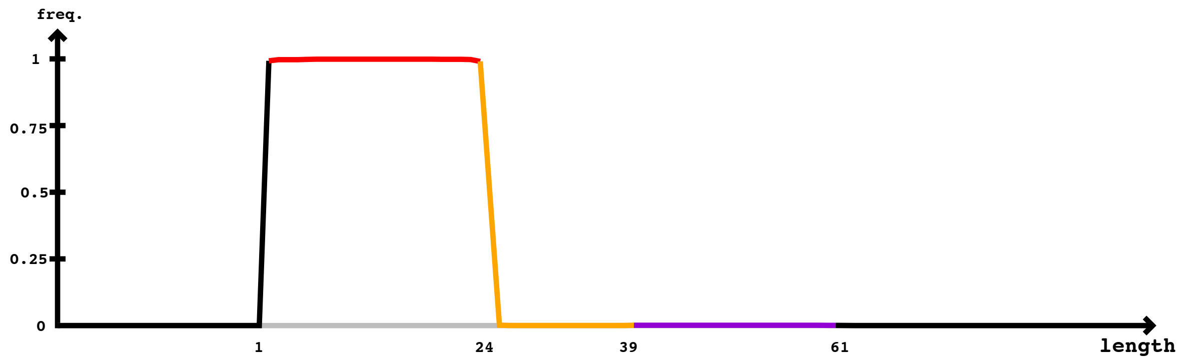


```
                         Mature                                     Star
5'- agaacuucugguuugcuucuaauggcacuggaagaauucacggauaaauguuugacuccgugguuucucuggugcuauaagaaguagaccaaaauuacaccuuuucaaaguuu  -3'   obs
    agaacuucugguuugcuucuaauggcacuggaagaauucacggauaaauguuugacuccgugguuucucuggugcuauaagaaguagaccaaaauuacaccuuuucaaaguuu        exp
    ........(((((((((((.(((((((..((.(((..((((((...........))))))..)))))..))))))).)))))))))))).........................  reads   mm   sample
    ....................aauggcacuggaagaauucacg.......................................................................  4       0    S04
    ....................aauggcacuggaagaauucacgg......................................................................  216     0    S04
    ....................aauggcacuggaagaauucacgga.....................................................................  22      0    S04
    ...........................................................gugguuucucuggugcuaua..................................  1       0    S04

    ....................aauggcacuggaagaauucac........................................................................  1       0    S02
    ....................aauggcacuggaagaauucacg.......................................................................  1       0    S02
    ....................aauggcacuggaagaauucacgg......................................................................  70      0    S02
    ....................aauggcacuggaagaauucacgga.....................................................................  4       0    S02
    ....................aauggcacuggaagaauucacggau....................................................................  1       0    S02

    ....................aauggcacuggaagaauucacg.......................................................................  3       0    S08
    ....................aauggcacuggaagaauucacgg......................................................................  197     0    S08
    ....................aauggcacuggaagaauucacgga.....................................................................  25      0    S08
    ....................aauggcacuggaagaauucacggau....................................................................  1       0    S08
    ..........................cacuggaagaauucacgg......................................................................  3       0    S08

    ....................aauggcacuggaagaauucac........................................................................  1       0    S09
    ....................aauggcacuggaagaauucacg.......................................................................  7       0    S09
    ....................aauggcacuggaagaauucacgg......................................................................  2031    0    S09
    ....................aauggcacuggaagaauucacgga.....................................................................  166     0    S09
    ....................aauggcacuggaagaauucacggau....................................................................  3       0    S09
    ....................aauggcacuggaagaauucacggaua...................................................................  1       0    S09
    .....................auggcacuggaagaauucacgg......................................................................  7       0    S09
    .....................auggcacuggaagaauucacgga.....................................................................  4       0    S09
    .........................gcacuggaagaauucacgg......................................................................  3       0    S09
    ..........................cacuggaagaauucacgg......................................................................  1       0    S09
    ...........................................................gugguuucucuggugcuauaag................................  2       0    S09

    ....................aauggcacuggaagaauucac........................................................................  1       0    S03
    ....................aauggcacuggaagaauucacg.......................................................................  3       0    S03
    ....................aauggcacuggaagaauucacgg......................................................................  159     0    S03
    ....................aauggcacuggaagaauucacgga.....................................................................  11      0    S03
```

Mature **Star**

agaacuucugguuugcuucuaauggcacuggaagaauucacggauaaauguuugacucc gugguuucucuggugcuauaagaaguagaccaaaauuacaccuuuucaaaguuu

....................aauggcacuggaagaauucacg...	8	0	S07
....................aauggcacuggaagaauucacgg..	413	0	S07
....................aauggcacuggaagaauucacgga...	52	0	S07
....................aauggcacuggaagaauucacggau..	1	0	S07
........................gcacuggaagaauucacg...	1	0	S07
....................aauggcacuggaagaauucacg...	1	0	S01
....................aauggcacuggaagaauucacgg..	30	0	S01
....................aauggcacuggaagaauucacg...	1	0	S06
....................aauggcacuggaagaauucacgg..	145	0	S06
....................aauggcacuggaagaauucacgga...	16	0	S06
.....................auggcacuggaagaauucacgg..	1	0	S06
....................aauggcacuggaagaauu...	3	0	S10
....................aauggcacuggaagaauucac..	2	0	S10
....................aauggcacuggaagaauucacg...	12	0	S10
....................aauggcacuggaagaauucacgg..	2271	0	S10
....................aauggcacuggaagaauucacgga...	166	0	S10
....................aauggcacuggaagaauucacggauaaauguuugacuc...	1	0	S10
.....................auggcacuggaagaauucacgg..	10	0	S10
.....................auggcacuggaagaauucacgga...	4	0	S10
........................gcacuggaagaauucacgg..	2	0	S10
.........................cacuggaagaauucacgg..	1	0	S10
....cgugguuucucuggugcuaua..................................	1	0	S10
....cgugguuucucuggugcuauaa.................................	1	0	S10
....gugguuucucuggugcuauaag................................	2	0	S10
....................aauggcacuggaagaauucac..	1	0	S05
....................aauggcacuggaagaauucacg...	3	0	S05
....................aauggcacuggaagaauucacgg..	190	0	S05
....................aauggcacuggaagaauucacgga...	16	0	S05
....................aauggcacuggaagaauucacggau..	1	0	S05
.....................auggcacuggaagaauucacgga...	1	0	S05
........................gcacuggaagaauucacgg..	1	0	S05

Provisional ID	:	Scaffold290_7704
Score total	:	3107.2
Score for star read(s)	:	3.9
Score for read counts	:	3099.4
Score for mfe	:	2.3
Score for randfold	:	1.6
Score for cons. seed	:	
Total read count	:	6091
Mature read count	:	3928
Loop read count	:	0
Star read count	:	2163

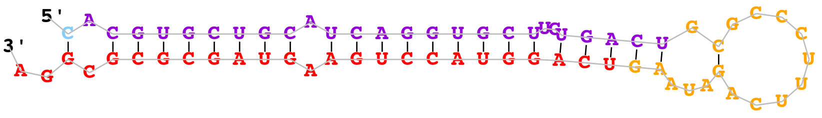

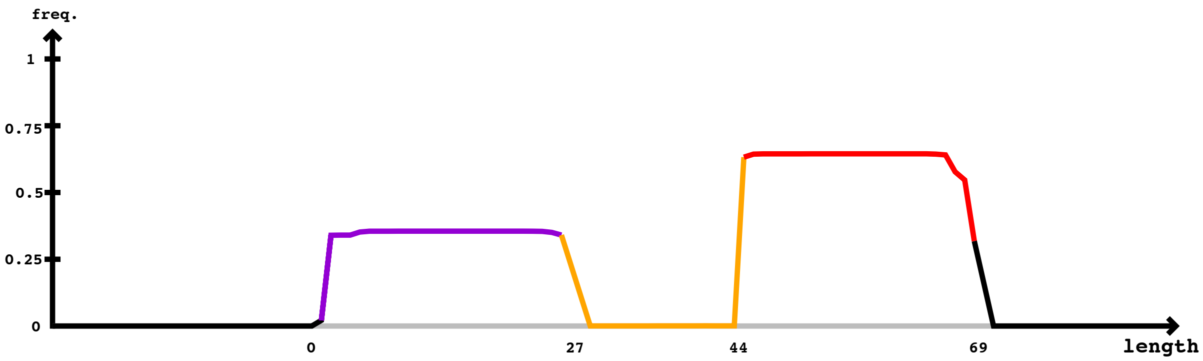

Star **Mature**

```
5'- aagaagaagagaggccgagggucucccacgugcugcaucaggugcuugugacugcgcccuuucagauaagucagguaccugaaguagcgcgcggagagccuugcggucauuacuu  -3'   obs
    aagaagaagagaggccgagggucucccacgugcugcaucaggugcuugugacugcgcccuuucagauaagucagguaccugaaguagcgcgcggagagccuugcggucauuacuu         exp
    ............(((((((((.(((((.((((((((.(((((((((..(((((.(.........)...)))))))))))))).)))))))).)).))))))).).......       reads   mm   sample
    ..........................cacgugcugcaucaggugcuuguga................................................................   1   0   S09
    ..........................cacgugcugcaucaggugcuugugac...............................................................   12   0   S09
    ..........................cacgugcugcaucaggugcuugugacu..............................................................   14   0   S09
    ...........................acgugcugcaucaggugcuugug.................................................................   5   0   S09
    ...........................acgugcugcaucaggugcuuguga................................................................   7   0   S09
    ...........................acgugcugcaucaggugcuugugac...............................................................   16   0   S09
    ...........................acgugcugcaucaggugcuugugacu..............................................................   161   0   S09
    ..............................ugcugcaucaggugcuuguga................................................................   2   0   S09
    ..............................ugcugcaucaggugcuugugacu..............................................................   5   0   S09
    ...............................gcugcaucaggugcuugugacu..............................................................   2   0   S09
    ......................................................................ucagguaccugaaguagcgcg........................   2   0   S09
    ......................................................................ucagguaccugaaguagcgcgc.......................   20   0   S09
    ......................................................................ucagguaccugaaguagcgcgcg......................   6   0   S09
    ......................................................................ucagguaccugaaguagcgcgcgg.....................   82   0   S09
    ......................................................................ucagguaccugaaguagcgcgcgga....................   64   0   S09
    ......................................................................ucagguaccugaaguagcgcgcggag...................   4   0   S09
    .......................................................................cagguaccugaaguagcgcgcg......................   1   0   S09
    .......................................................................cagguaccugaaguagcgcgcgg.....................   1   0   S09
    .......................................................................cagguaccugaaguagcgcgcgga....................   1   0   S09

    ..........................cacgugcugcaucaggugcuuguga................................................................   1   0   S03
    ..........................cacgugcugcaucaggugcuugugac...............................................................   7   0   S03
    ..........................cacgugcugcaucaggugcuugugacu..............................................................   3   0   S03
    ...........................acgugcugcaucaggugcuugug.................................................................   4   0   S03
    ...........................acgugcugcaucaggugcuuguga................................................................   9   0   S03
    ...........................acgugcugcaucaggugcuugugac...............................................................   50   0   S03
    ...........................acgugcugcaucaggugcuugugacu..............................................................   257   0   S03
    ............................cgugcugcaucaggugcuugugacu..............................................................   1   0   S03
    ..............................ugcugcaucaggugcuugugac...............................................................   2   0   S03
    ..............................ugcugcaucaggugcuugugacu..............................................................   4   0   S03
    ...............................gcugcaucaggugcuugugacu..............................................................   4   0   S03
    .............................................................ucagauaagucagguaccugaaguagcgcgcgga....................   1   0   S03
    ..................................................................uaagucagguaccugaaguagcgcgcgg.....................   1   0   S03
```

Star **Mature**

```
aagaagaagagaggccgagggucucccacgugcugcaucaggugcuugugacugcgcccuuucagauaagucagguaccugaaguagcgcgcggagagccuugcggucauuacuu
......................................................................ucagguaccugaaguagcgc.........................    1    0    S03
......................................................................ucagguaccugaaguagcgcg........................    1    0    S03
......................................................................ucagguaccugaaguagcgcgc.......................    46   0    S03
......................................................................ucagguaccugaaguagcgcgcg......................    16   0    S03
......................................................................ucagguaccugaaguagcgcgcgg.....................    154  0    S03
......................................................................ucagguaccugaaguagcgcgcgga....................    292  0    S03
......................................................................ucagguaccugaaguagcgcgcggag...................    6    0    S03
.......................................................................cagguaccugaaguagcgcgc.......................    1    0    S03
.......................................................................cagguaccugaaguagcgcgcgg.....................    11   0    S03
.......................................................................cagguaccugaaguagcgcgcgga....................    2    0    S03
........................................................................agguaccugaaguagcgcgcgg.....................    1    0    S03

..........................cacgugcugcaucaggugcuuguga................................................................    1    0    S08
..........................cacgugcugcaucaggugcuugugac...............................................................    11   0    S08
..........................cacgugcugcaucaggugcuugugacu..............................................................    1    0    S08
..........................cacgugcugcaucaggugcuugugacugcgcccuuucagau................................................    1    0    S08
...........................acgugcugcaucaggugcuugug.................................................................    3    0    S08
...........................acgugcugcaucaggugcuuguga................................................................    6    0    S08
...........................acgugcugcaucaggugcuugugac...............................................................    57   0    S08
...........................acgugcugcaucaggugcuugugacu..............................................................    97   0    S08
..............................ugcugcaucaggugcuugugacu..............................................................    3    0    S08
...............................gcugcaucaggugcuugugacu..............................................................    2    0    S08
..................................gcaucaggugcuugugacu..............................................................    1    0    S08
......................................................................ucagguaccugaaguagcgc.........................    1    0    S08
......................................................................ucagguaccugaaguagcgcg........................    3    0    S08
......................................................................ucagguaccugaaguagcgcgc.......................    54   0    S08
......................................................................ucagguaccugaaguagcgcgcg......................    37   0    S08
......................................................................ucagguaccugaaguagcgcgcgg.....................    365  0    S08
......................................................................ucagguaccugaaguagcgcgcgga....................    367  0    S08
......................................................................ucagguaccugaaguagcgcgcggag...................    3    0    S08
.......................................................................cagguaccugaaguagcgcg........................    1    0    S08
.......................................................................cagguaccugaaguagcgcgcgg.....................    2    0    S08
.......................................................................cagguaccugaaguagcgcgcgga....................    4    0    S08

..........................cacgugcugcaucaggugcuugugac...............................................................    1    0    S02
..........................cacgugcugcaucaggugcuugugacu..............................................................    2    0    S02
...........................acgugcugcaucaggugcuugug.................................................................    2    0    S02
...........................acgugcugcaucaggugcuugugac...............................................................    7    0    S02
...........................acgugcugcaucaggugcuugugacu..............................................................    48   0    S02
..............................ugcugcaucaggugcuugugac...............................................................    2    0    S02
..............................ugcugcaucaggugcuugugacu..............................................................    3    0    S02
...............................gcugcaucaggugcuugugacu..............................................................    2    0    S02
......................................................................ucagguaccugaaguagcgc.........................    2    0    S02
......................................................................ucagguaccugaaguagcgcg........................    4    0    S02
......................................................................ucagguaccugaaguagcgcgc.......................    54   0    S02
......................................................................ucagguaccugaaguagcgcgcg......................    9    0    S02
......................................................................ucagguaccugaaguagcgcgcgg.....................    34   0    S02
......................................................................ucagguaccugaaguagcgcgcgga....................    83   0    S02
......................................................................ucagguaccugaaguagcgcgcggag...................    2    0    S02
.......................................................................cagguaccugaaguagcgcgcgg.....................    1    0    S02
........................................................................agguaccugaaguagcgcgcgga....................    2    0    S02

..........................cacgugcugcaucaggugcuugugac...............................................................    13   0    S04
..........................cacgugcugcaucaggugcuugugacu..............................................................    7    0    S04
...........................acgugcugcaucaggugcuugu..................................................................    1    0    S04
...........................acgugcugcaucaggugcuugug.................................................................    3    0    S04
...........................acgugcugcaucaggugcuuguga................................................................    7    0    S04
...........................acgugcugcaucaggugcuugugac...............................................................    71   0    S04
...........................acgugcugcaucaggugcuugugacu..............................................................    321  0    S04
............................cgugcugcaucaggugcuugugacu..............................................................    1    0    S04
..............................ugcugcaucaggugcuugugac...............................................................    4    0    S04
..............................ugcugcaucaggugcuugugacu..............................................................    10   0    S04
...............................gcugcaucaggugcuugugacu..............................................................    3    0    S04
......................................................................ucagguaccugaaguagcgcg........................    1    0    S04
......................................................................ucagguaccugaaguagcgcgc.......................    39   0    S04
......................................................................ucagguaccugaaguagcgcgcg......................    27   0    S04
......................................................................ucagguaccugaaguagcgcgcgg.....................    176  0    S04
......................................................................ucagguaccugaaguagcgcgcgga....................    377  0    S04
......................................................................ucagguaccugaaguagcgcgcggag...................    5    0    S04
.......................................................................cagguaccugaaguagcgcgc.......................    1    0    S04
```

Star **Mature**

```
aagaagaagagaggccgagggucucccacgugcugcaucaggugcuugugacugcgcccuuucagauaagucagguaccugaaguagcgcgcggagagccuugcggucauuacuu
.......................................................................cagguaccugaaguagcgcgcgg.....................   7     0   S04
.......................................................................cagguaccugaaguagcgcgcgga....................   4     0   S04

..........................cacgugcugcaucaggugcuuguga................................................................   1     0   S05
..........................cacgugcugcaucaggugcuugugac...............................................................   3     0   S05
..........................cacgugcugcaucaggugcuugugacu..............................................................   4     0   S05
...........................acgugcugcaucaggugcuugug.................................................................   2     0   S05
...........................acgugcugcaucaggugcuuguga................................................................   1     0   S05
...........................acgugcugcaucaggugcuugugac...............................................................   35    0   S05
...........................acgugcugcaucaggugcuugugacu..............................................................   179   0   S05
...........................acgugcugcaucaggugcuugugacugcgcccuuucagauaaguc...........................................   1     0   S05
............................cgugcugcaucaggugcuugugacu..............................................................   1     0   S05
..............................ugcugcaucaggugcuugugacu..............................................................   5     0   S05
...............................gcugcaucaggugcuugugacu..............................................................   2     0   S05
......................................................................ucagguaccugaaguagcgc.........................   2     0   S05
......................................................................ucagguaccugaaguagcgcg........................   1     0   S05
......................................................................ucagguaccugaaguagcgcgc.......................   29    0   S05
......................................................................ucagguaccugaaguagcgcgcg......................   14    0   S05
......................................................................ucagguaccugaaguagcgcgcgg.....................   118   0   S05
......................................................................ucagguaccugaaguagcgcgcgga....................   177   0   S05
......................................................................ucagguaccugaaguagcgcgcggag...................   6     0   S05
.......................................................................cagguaccugaaguagcgcgcgg.....................   7     0   S05
........................................................................agguaccugaaguagcgcgcgga....................   2     0   S05

..........................cacgugcugcaucaggugcuugug.................................................................   1     0   S10
..........................cacgugcugcaucaggugcuuguga................................................................   1     0   S10
..........................cacgugcugcaucaggugcuugugac...............................................................   7     0   S10
..........................cacgugcugcaucaggugcuugugacu..............................................................   17    0   S10
...........................acgugcugcaucaggugcuugu..................................................................   2     0   S10
...........................acgugcugcaucaggugcuugug.................................................................   1     0   S10
...........................acgugcugcaucaggugcuuguga................................................................   11    0   S10
...........................acgugcugcaucaggugcuugugac...............................................................   22    0   S10
...........................acgugcugcaucaggugcuugugacu..............................................................   187   0   S10
..............................ugcugcaucaggugcuugugacu..............................................................   4     0   S10
...............................gcugcaucaggugcuugugacu..............................................................   2     0   S10
......................................................................ucagguaccugaaguagcgcg........................   1     0   S10
......................................................................ucagguaccugaaguagcgcgc.......................   15    0   S10
......................................................................ucagguaccugaaguagcgcgcg......................   22    0   S10
......................................................................ucagguaccugaaguagcgcgcgg.....................   127   0   S10
......................................................................ucagguaccugaaguagcgcgcgga....................   125   0   S10
......................................................................ucagguaccugaaguagcgcgcggag...................   4     0   S10
.......................................................................cagguaccugaaguagcgcg........................   1     0   S10
.......................................................................cagguaccugaaguagcgcgc.......................   1     0   S10
.......................................................................cagguaccugaaguagcgcgcgg.....................   3     0   S10
.......................................................................cagguaccugaaguagcgcgcgga....................   1     0   S10
........................................................................agguaccugaaguagcgcgcgga....................   1     0   S10

..........................cacgugcugcaucaggugcuugugac...............................................................   5     0   S06
..........................cacgugcugcaucaggugcuugugacu..............................................................   3     0   S06
...........................acgugcugcaucaggugcuuguga................................................................   3     0   S06
...........................acgugcugcaucaggugcuugugac...............................................................   32    0   S06
...........................acgugcugcaucaggugcuugugacu..............................................................   141   0   S06
..............................ugcugcaucaggugcuugugacu..............................................................   7     0   S06
...............................gcugcaucaggugcuugugacu..............................................................   1     0   S06
......................................................................ucagguaccugaaguagcgcg........................   1     0   S06
......................................................................ucagguaccugaaguagcgcgc.......................   39    0   S06
......................................................................ucagguaccugaaguagcgcgcg......................   14    0   S06
......................................................................ucagguaccugaaguagcgcgcgg.....................   116   0   S06
......................................................................ucagguaccugaaguagcgcgcgga....................   114   0   S06
.......................................................................cagguaccugaaguagcgcgcgg.....................   14    0   S06

...........................acgugcugcaucaggugcuugugacu..............................................................   4     0   S01
..............................ugcugcaucaggugcuugugac...............................................................   4     0   S01
..............................ugcugcaucaggugcuugugacu..............................................................   6     0   S01
......................................................................ucagguaccugaaguagcgc.........................   1     0   S01
......................................................................ucagguaccugaaguagcgcg........................   2     0   S01
......................................................................ucagguaccugaaguagcgcgc.......................   39    0   S01
......................................................................ucagguaccugaaguagcgcgcg......................   15    0   S01
......................................................................ucagguaccugaaguagcgcgcgg.....................   23    0   S01
......................................................................ucagguaccugaaguagcgcgcgga....................   34    0   S01
```

Star Mature

```
aagaagaagagaggccgagggucucccacgugcugcaucaggugcuugugacugcgcccuuucagauaagucagguaccugaaguagcgcgcggagagccuugcggucauuacuu
......................................................................ucagguaccugaaguagcgcgcggag...................	3	0	S01
.......................................................................cagguaccugaaguagcgcgcgg.....................	3	0	S01

..........................cacgugcugcaucaggugcuug...................................................................	1	0	S07
..........................cacgugcugcaucaggugcuugugac...............................................................	6	0	S07
..........................cacgugcugcaucaggugcuugugacu..............................................................	6	0	S07
...........................acgugcugcaucaggugcuugu..................................................................	1	0	S07
...........................acgugcugcaucaggugcuugug.................................................................	3	0	S07
...........................acgugcugcaucaggugcuuguga................................................................	9	0	S07
...........................acgugcugcaucaggugcuugugac...............................................................	30	0	S07
...........................acgugcugcaucaggugcuugugacu..............................................................	145	0	S07
............................cgugcugcaucaggugcuugugac...............................................................	1	0	S07
..............................ugcugcaucaggugcuugugac...............................................................	2	0	S07
..............................ugcugcaucaggugcuugugacu..............................................................	7	0	S07
...............................gcugcaucaggugcuugugacu..............................................................	1	0	S07
......................................................................ucagguaccugaaguagcgcg........................	1	0	S07
......................................................................ucagguaccugaaguagcgcgc.......................	48	0	S07
......................................................................ucagguaccugaaguagcgcgcg......................	24	0	S07
......................................................................ucagguaccugaaguagcgcgcgg.....................	149	0	S07
......................................................................ucagguaccugaaguagcgcgcgga....................	250	0	S07
.......................................................................cagguaccugaaguagcgcgcgg.....................	1	0	S07
.......................................................................cagguaccugaaguagcgcgcgga....................	1	0	S07
........................................................................agguaccugaaguagcgcgcgga....................	1	0	S07
.............................................................................ccugaaguagcgcgcgga....................	1	0	S07
```

Provisional ID	: Scaffold2464_35273
Score total	: 466.5
Score for star read(s)	: 3.9
Score for read counts	: 459
Score for mfe	: 2
Score for randfold	: 1.6
Score for cons. seed	:
Total read count	: 912
Mature read count	: 866
Loop read count	: 0
Star read count	: 46

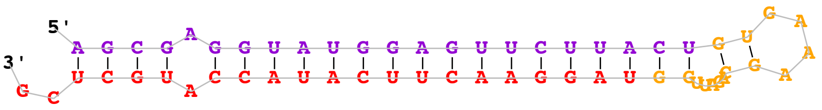

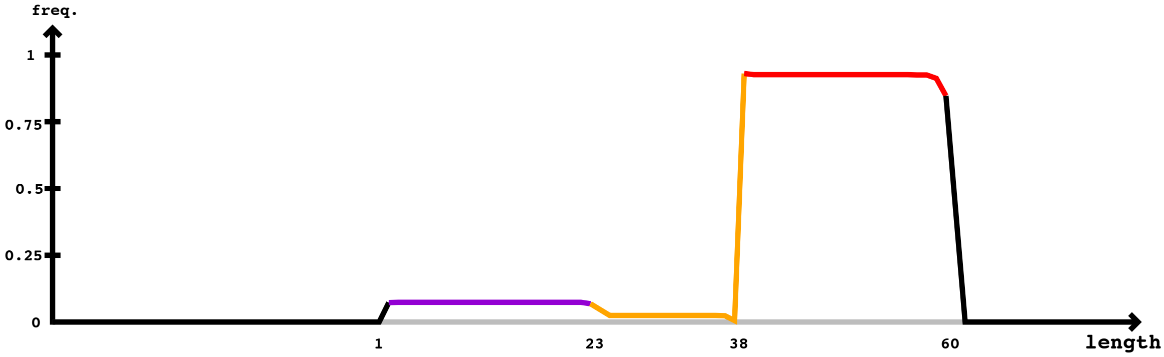


```
                                 Star                                Mature
5'- uaguuguaucguacugcauaguuguucaucgagagcgagguauggaguucuuacugugaaagcagauugguaggaacuucauaccaugcucgugaugaauuggcaaaucca  -3'  obs
    uaguuguaucguacugcauaguuguucaucgagagcgagguauggaguucuuacugugaaagcagauugguaggaacuucauaccaugcucgugaugaauuggcaaaucca       exp
    ..............(((......((((((((.(((((.(((((((((((((((((((....)).....)))))))))))))))))).))))).))))))))..)))......  reads  mm  sample
    .................................agcgagguauggaguucuuacu........................................................  12     0   S09
    .................................agcgagguauggaguucuuacugugaaagcagauuggu........................................  1      0   S09
    ......................................................................uaggaacuucauaccaugcu.....................  1      0   S09
    ......................................................................uaggaacuucauaccaugcuc....................  5      0   S09
    ......................................................................uaggaacuucauaccaugcucg...................  133    0   S09
    ......................................................................uaggaacuucauaccaugcucgu..................  5      0   S09

    .................................agcgagguauggaguucuuacug.......................................................  1      0   S03
    .................................agcgagguauggaguucuuacugugaaagcagauug..........................................  5      0   S03
    ......................................................................uaggaacuucauaccaugcuc....................  12     0   S03
    ......................................................................uaggaacuucauaccaugcucg...................  54     0   S03
    ......................................................................uaggaacuucauaccaugcucgu..................  1      0   S03

    .................................agcgagguauggaguucuuac.........................................................  1      0   S08
    .................................agcgagguauggaguucuuacugugaaagcagauuggu........................................  1      0   S08
    ......................................................................uaggaacuucauaccaug.......................  1      0   S08
    ......................................................................uaggaacuucauaccaugcu.....................  1      0   S08
    ......................................................................uaggaacuucauaccaugcuc....................  2      0   S08
    ......................................................................uaggaacuucauaccaugcucg...................  67     0   S08
    ......................................................................uaggaacuucauaccaugcucgu..................  8      0   S08

    .................................agcgagguauggaguucuuacugugaaagcagauug..........................................  1      0   S02
    ..................................gcgagguauggaguucuuacug.......................................................  1      0   S02
    ......................................................................uaggaacuucauaccaugcu.....................  2      0   S02
    ......................................................................uaggaacuucauaccaugcuc....................  7      0   S02
    ......................................................................uaggaacuucauaccaugcucg...................  26     0   S02

    .................................agcgagguauggaguucuuac.........................................................  2      0   S04
    .................................agcgagguauggaguucuuacu........................................................  7      0   S04
    .................................agcgagguauggaguucuuacug.......................................................  2      0   S04
    .................................agcgagguauggaguucuuacugugaaagcagauug..........................................  4      0   S04
    .................................agcgagguauggaguucuuacugugaaagcagauugg.........................................  1      0   S04
    .................................agcgagguauggaguucuuacugugaaagcagauuggu........................................  1      0   S04
```

```
                                 Star                                 Mature
uaguuguaucguacugcauaguuguucaucgagagcgagguauggaguucuuacugugaaagcagauugguaggaacuucauaccaugcucgugaugaauuggcaaaucca
......................................................................uaggaacuucauaccaugcuc....................    5     0    S04
......................................................................uaggaacuucauaccaugcucg...................    71    0    S04
......................................................................uaggaacuucauaccaugcucgu..................    2     0    S04

.................................agcgagguauggaguucuuacu........................................................    3     0    S05
.................................agcgagguauggaguucuuacug.......................................................    1     0    S05
.................................agcgagguauggaguucuuacugugaaagcagauug..........................................    2     0    S05
......................................................................uaggaacuucauaccaugcu.....................    1     0    S05
......................................................................uaggaacuucauaccaugcuc....................    8     0    S05
......................................................................uaggaacuucauaccaugcucg...................    51    0    S05
......................................................................uaggaacuucauaccaugcucgu..................    6     0    S05

.................................agcgagguauggaguucuuac.........................................................    1     0    S10
.................................agcgagguauggaguucuuacu........................................................    6     0    S10
.................................agcgagguauggaguucuuacug.......................................................    1     0    S10
.................................agcgagguauggaguucuuacugugaaagcagauu...........................................    1     0    S10
.................................agcgagguauggaguucuuacugugaaagcagauug..........................................    1     0    S10
......................................................................uaggaacuucauaccaugcu.....................    2     0    S10
......................................................................uaggaacuucauaccaugcuc....................    6     0    S10
......................................................................uaggaacuucauaccaugcucg...................    154   0    S10
......................................................................uaggaacuucauaccaugcucgu..................    7     0    S10

.................................agcgagguauggaguucuuacu........................................................    2     0    S06
.................................agcgagguauggaguucuuacugugaaagcagauug..........................................    2     0    S06
......................................................................uaggaacuucauaccaugcu.....................    1     0    S06
......................................................................uaggaacuucauaccaugcuc....................    2     0    S06
......................................................................uaggaacuucauaccaugcucg...................    44    0    S06
......................................................................uaggaacuucauaccaugcucgu..................    4     0    S06

.................................agcgagguauggaguucuuacug.......................................................    1     0    S01
......................................................................uaggaacuucauaccaugcu.....................    3     0    S01
......................................................................uaggaacuucauaccaugcuc....................    8     0    S01
......................................................................uaggaacuucauaccaugcucg...................    33    0    S01
......................................................................uaggaacuucauaccaugcucgu..................    1     0    S01

.................................agcgagguauggaguucuuac.........................................................    1     0    S07
.................................agcgagguauggaguucuuacu........................................................    4     0    S07
.................................agcgagguauggaguucuuacugugaaagcagauug..........................................    2     0    S07
.................................agcgagguauggaguucuuacugugaaagcagauuggu........................................    1     0    S07
......................................................................uaggaacuucauaccaugcu.....................    1     0    S07
......................................................................uaggaacuucauaccaugcuc....................    6     0    S07
......................................................................uaggaacuucauaccaugcucg...................    121   0    S07
......................................................................uaggaacuucauaccaugcucgu..................    4     0    S07
```

Provisional ID	:	Scaffold55_1663
Score total	:	449.8
Score for star read(s)	:	3.9
Score for read counts	:	442.1
Score for mfe	:	2.2
Score for randfold	:	1.6
Score for cons. seed	:	
Total read count	:	879
Mature read count	:	833
Loop read count	:	0
Star read count	:	46

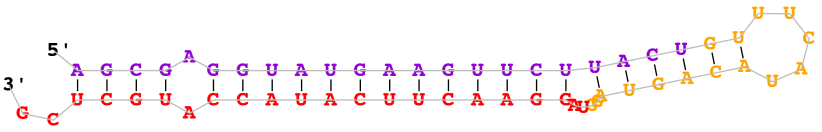

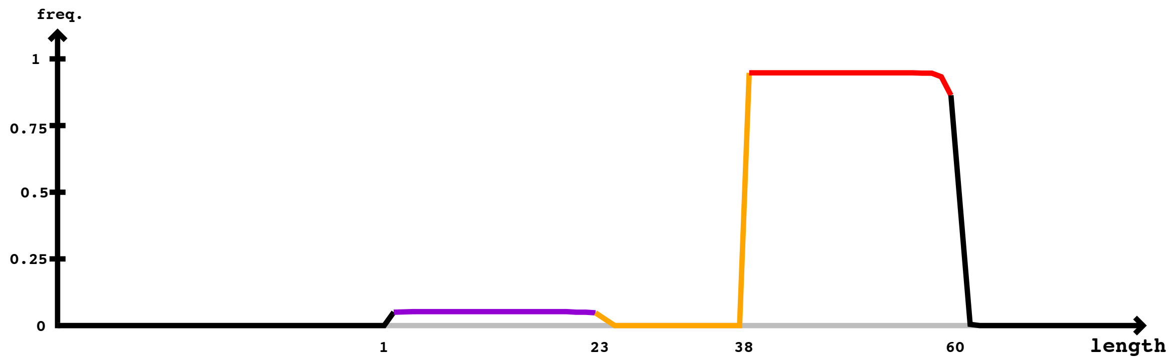


```
                                                                  Star                                  Mature
5'- guguggaguaaaggaaauguuuaaacuacgcagagcgagguaugaaguucuuacuguuucauacaguagguaggaacuucauaccaugcucggugugguuuaaaaaaugcc   -3'   obs
    guguggaguaaaggaaauguuuaaacuacgcagagcgagguaugaaguucuuacuguuucauacaguagguaggaacuucauaccaugcucggugugguuuaaaaaaugcc         exp
    ............((.....((((((((((((.(((((.(((((((((((((((((((.....))))))....))))))))))))).))))).)))))))))))).....))   reads   mm   sample
    .................................agcgagguaugaaguucuuac.........................................................   1       0    S07
    .................................agcgagguaugaaguucuuacu........................................................   3       0    S07
    ......................................................................uaggaacuucauaccaugcu.....................   1       0    S07
    ......................................................................uaggaacuucauaccaugcuc....................   6       0    S07
    ......................................................................uaggaacuucauaccaugcucg...................   121     0    S07
    ......................................................................uaggaacuucauaccaugcucggu.................   1       0    S07

    .................................agcgagguaugaaguucuuacu........................................................   2       0    S01
    ...................................cgagguaugaaguucuuacu........................................................   1       0    S01
    ......................................................................uaggaacuucauaccaugcu.....................   3       0    S01
    ......................................................................uaggaacuucauaccaugcuc....................   8       0    S01
    ......................................................................uaggaacuucauaccaugcucg...................   33      0    S01

    .................................agcgagguaugaaguucuuac.........................................................   1       0    S06
    .................................agcgagguaugaaguucuuacu........................................................   9       0    S06
    ......................................................................uaggaacuucauaccaugcu.....................   1       0    S06
    ......................................................................uaggaacuucauaccaugcuc....................   2       0    S06
    ......................................................................uaggaacuucauaccaugcucg...................   44      0    S06
    ......................................................................uaggaacuucauaccaugcucggu.................   2       0    S06

    .................................agcgagguaugaaguucuuacu........................................................   5       0    S10
    ......................................................................uaggaacuucauaccaugcu.....................   2       0    S10
    ......................................................................uaggaacuucauaccaugcuc....................   6       0    S10
    ......................................................................uaggaacuucauaccaugcucg...................   154     0    S10

    .................................agcgagguaugaaguucuuacu........................................................   7       0    S05
    ......................................................................uaggaacuucauaccaugcu.....................   1       0    S05
    ......................................................................uaggaacuucauaccaugcuc....................   8       0    S05
    ......................................................................uaggaacuucauaccaugcucg...................   51      0    S05

    .................................agcgagguaugaaguucuuacu........................................................   8       0    S04
    ......................................................................uaggaacuucauaccaugcuc....................   5       0    S04
    ......................................................................uaggaacuucauaccaugcucg...................   71      0    S04
```

```
                                 Star                                Mature
gugugagguaaaggaaauguuuaaacuacgcagagcgagguaugaaguucuuacuguuucauacaguagguaggaacuucauaccaugcucggugugguuuaaaaaaaugcc

.................................agcgagguaugaaguucuuacu.........................................................  3    0    S02
......................................................................uaggaacuucauaccaugcu......................  2    0    S02
......................................................................uaggaacuucauaccaugcuc.....................  7    0    S02
......................................................................uaggaacuucauaccaugcucg....................  26   0    S02

.................................agcgagguaugaaguucuuacu.........................................................  1    0    S08
......................................................................uaggaacuucauaccaug........................  1    0    S08
......................................................................uaggaacuucauaccaugcu......................  1    0    S08
......................................................................uaggaacuucauaccaugcuc.....................  2    0    S08
......................................................................uaggaacuucauaccaugcucg....................  67   0    S08

.................................agcgagguaugaaguucuu............................................................  2    0    S03
.................................agcgagguaugaaguucuuacu.........................................................  1    0    S03
..................................gcgagguaugaaguucuuacug........................................................  1    0    S03
......................................................................uaggaacuucauaccaugcuc.....................  12   0    S03
......................................................................uaggaacuucauaccaugcucg....................  54   0    S03

.................................agcgagguaugaaguucuuacu.........................................................  1    0    S09
......................................................................uaggaacuucauaccaugcu......................  1    0    S09
......................................................................uaggaacuucauaccaugcuc.....................  5    0    S09
......................................................................uaggaacuucauaccaugcucg....................  133  0    S09
......................................................................uaggaacuucauaccaugcucgg...................  1    0    S09
......................................................................uaggaacuucauaccaugcucggu..................  1    0    S09
```

Provisional ID : Scaffold33_1067
Score total : 38243.3
Score for star read(s) : 3.9
Score for read counts : 38236
Score for mfe : 1.8
Score for randfold : 1.6
Score for cons. seed :
Total read count : 75010
Mature read count : 74738
Loop read count : 8
Star read count : 264

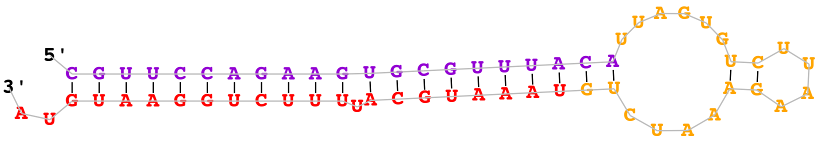

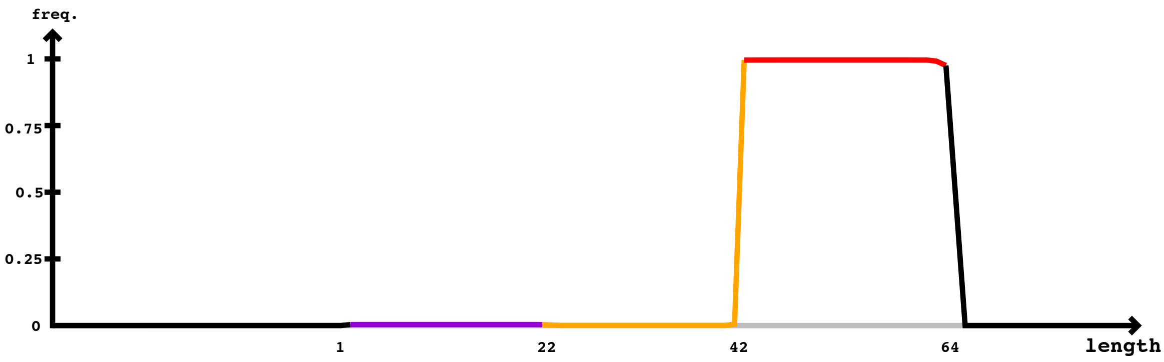

Star Mature

```
5'- cuugaagaugaagucagcugccguaccuccguuccagaagugcguuuacauuagugucuuaagaaaucuguaaaugcauuuucuggaauguaaggugcgauugccgcaucc   -3'   obs
    cuugaagaugaagucagcugccguaccuccguuccagaagugcguuuacauuagugucuuaagaaaucuguaaaugcauuuucuggaauguaaggugcgauugccgcaucc         exp
    ......((((......((...(((((((.(((((((((((((((((((((......((....))....)))))))))).)))))))))))..)))))))...))..)))).   reads   mm   sample
    ........ugaagucagcugccguaccuc..................................................................................   1       0    S04
    .............................cguuccagaagugcguuuaca.............................................................   6       0    S04
    .....................................................................guaaaugcauuuucuggaaug.....................   3       0    S04
    .....................................................................guaaaugcauuuucuggaaugua...................   27      0    S04
    ......................................................................uaaaugcauuuucuggaaug.....................   26      0    S04
    ......................................................................uaaaugcauuuucuggaaugu....................   79      0    S04
    ......................................................................uaaaugcauuuucuggaaugua...................   3986    0    S04
    ......................................................................uaaaugcauuuucuggaauguaa..................   1       0    S04

    .....................................................................guaaaugcauuuucuggaaugua...................   8       0    S02
    ......................................................................uaaaugcauuuucuggaau......................   1       0    S02
    ......................................................................uaaaugcauuuucuggaaug.....................   14      0    S02
    ......................................................................uaaaugcauuuucuggaaugu....................   9       0    S02
    ......................................................................uaaaugcauuuucuggaaugua...................   724     0    S02
    ......................................................................uaaaugcauuuucuggaauguaa..................   1       0    S02

    .............................cguuccagaagugcguuuac..............................................................   2       0    S08
    .............................cguuccagaagugcguuuaca.............................................................   7       0    S08
    .............................cguuccagaagugcguuuacau............................................................   1       0    S08
    .....................................................................guaaaugcauuuucuggaaugua...................   12      0    S08
    ......................................................................uaaaugcauuuucuggaa.......................   1       0    S08
    ......................................................................uaaaugcauuuucuggaaug.....................   9       0    S08
    ......................................................................uaaaugcauuuucuggaaugu....................   60      0    S08
    ......................................................................uaaaugcauuuucuggaaugua...................   2943    0    S08
    ......................................................................uaaaugcauuuucuggaauguaa..................   3       0    S08
    ........................................................................aaugcauuuucuggaaugua...................   1       0    S08
    ..........................................................................ugcauuuucuggaaugua...................   2       0    S08

    .......augaagucagcugccguaccuc..................................................................................   2       0    S03
    .............................cguuccagaagugcguuuac..............................................................   1       0    S03
    .............................cguuccagaagugcguuuaca.............................................................   5       0    S03
    .............................cguuccagaagugcguuuacau............................................................   1       0    S03
    ..................................................uuagugucuuaagaaaucug.........................................   1       0    S03
```

```
                                   Star                                         Mature
cuugaagaugaagucagcugccguaccuccguuccagaagugcguuuacauuagugucuuaagaaaucuguaaaugcauuuucuggaauguaaggugcgauugccgcaucc
.....................................................................guaaaugcauuuucuggaaug.....................   1       0   S03
.....................................................................guaaaugcauuuucuggaaugua...................   12      0   S03
......................................................................uaaaugcauuuucuggaaug.....................   9       0   S03
......................................................................uaaaugcauuuucuggaaugu....................   30      0   S03
......................................................................uaaaugcauuuucuggaaugua...................   1555    0   S03
......................................................................uaaaugcauuuucuggaauguaa..................   2       0   S03
.......................................................................aaaugcauuuucuggaaugua...................   2       0   S03

...........................uccguuccagaagugcguuuaca.............................................................   1       0   S09
.............................cguuccagaagugcguuuac..............................................................   7       0   S09
.............................cguuccagaagugcguuuaca.............................................................   96      0   S09
.............................cguuccagaagugcguuuacau............................................................   10      0   S09
.............................cguuccagaagugcguuuacauuagugucuuaagaaaucug.........................................   5       0   S09
.............................cguuccagaagugcguuuacauuagugucuuaagaaaucugu........................................   1       0   S09
..............................guuccagaagugcguuuaca.............................................................   1       0   S09
..................................................uuagugucuuaagaaaucug.........................................   2       0   S09
.....................................................................guaaaugcauuuucuggaaug.....................   2       0   S09
.....................................................................guaaaugcauuuucuggaaugu....................   2       0   S09
.....................................................................guaaaugcauuuucuggaaugua...................   134     0   S09
......................................................................uaaaugcauuuucuggaa.......................   3       0   S09
......................................................................uaaaugcauuuucuggaau......................   1       0   S09
......................................................................uaaaugcauuuucuggaaug.....................   71      0   S09
......................................................................uaaaugcauuuucuggaaugu....................   384     0   S09
......................................................................uaaaugcauuuucuggaaugua...................   27426   0   S09
......................................................................uaaaugcauuuucuggaauguaa..................   19      0   S09
.......................................................................aaaugcauuuucuggaaug.....................   1       0   S09
.......................................................................aaaugcauuuucuggaaugua...................   6       0   S09
........................................................................aaugcauuuucuggaaugua...................   4       0   S09
.........................................................................augcauuuucuggaaugua...................   1       0   S09
..........................................................................ugcauuuucuggaaugua...................   5       0   S09

.........gaagucagcugccguaccuc..................................................................................   1       0   S07
.............................cguuccagaagugcguuuac..............................................................   1       0   S07
.............................cguuccagaagugcguuuaca.............................................................   23      0   S07
.............................cguuccagaagugcguuuacau............................................................   7       0   S07
.............................cguuccagaagugcguuuacauuagugucuuaagaaaucug.........................................   8       0   S07
.............................cguuccagaagugcguuuacauuagugucuuaagaaaucugu........................................   1       0   S07
..............................guuccagaagugcguuuaca.............................................................   2       0   S07
..................................................uuagugucuuaagaaauc...........................................   2       0   S07
.....................................................................guaaaugcauuuucuggaaug.....................   1       0   S07
.....................................................................guaaaugcauuuucuggaaugua...................   10      0   S07
......................................................................uaaaugcauuuucuggaa.......................   1       0   S07
......................................................................uaaaugcauuuucuggaaug.....................   16      0   S07
......................................................................uaaaugcauuuucuggaaugu....................   53      0   S07
......................................................................uaaaugcauuuucuggaaugua...................   2229    0   S07

...............................................acauuagugucuuaagaaaucu..........................................   2       0   S01
.....................................................................guaaaugcauuuucuggaaugua...................   2       0   S01
......................................................................uaaaugcauuuucuggaau......................   2       0   S01
......................................................................uaaaugcauuuucuggaaug.....................   10      0   S01
......................................................................uaaaugcauuuucuggaaugu....................   31      0   S01
......................................................................uaaaugcauuuucuggaaugua...................   990     0   S01
......................................................................uaaaugcauuuucuggaauguaa..................   4       0   S01

.............................cguuccagaagugcguuuac..............................................................   1       0   S06
.............................cguuccagaagugcguuuaca.............................................................   4       0   S06
.............................cguuccagaagugcguuuacauuagugucuuaagaaaucug.........................................   1       0   S06
..............................guuccagaagugcguuuaca.............................................................   1       0   S06
..................................................uuagugucuuaagaaaucu..........................................   1       0   S06
.....................................................................guaaaugcauuuucuggaa.......................   1       0   S06
.....................................................................guaaaugcauuuucuggaaug.....................   2       0   S06
.....................................................................guaaaugcauuuucuggaaugua...................   17      0   S06
......................................................................uaaaugcauuuucuggaau......................   1       0   S06
......................................................................uaaaugcauuuucuggaaug.....................   39      0   S06
......................................................................uaaaugcauuuucuggaaugu....................   72      0   S06
......................................................................uaaaugcauuuucuggaaugua...................   3615    0   S06
......................................................................uaaaugcauuuucuggaauguaa..................   2       0   S06

.........gaagucagcugccguaccuc..................................................................................   2       0   S10
.............................cguuccagaagugcguuuac..............................................................   7       0   S10
```

Star **Mature**

cuugaagaugaagucagcugccguaccuccguuccagaagugcguuuacauuagugucuuaagaaaucuguaaaugcauuuucuggaauguaaggugcgauugccgcaucc			
.............................cguuccagaagugcguuuaca...	64	0	S10
.............................cguuccagaagugcguuuacau..	9	0	S10
.............................cguuccagaagugcguuuacauuagugucuuaagaaaucug...	2	0	S10
..............................guuccagaagugcguuuaca...	1	0	S10
....uuagugucuuaagaaaucu..	1	0	S10
....uuagugucuuaagaaaucugu..	1	0	S10
....cuuaagaaaucuguaaaugcau................................	1	0	S10
....guaaaugcauuuucuggaaug.....................	2	0	S10
....guaaaugcauuuucuggaaugu....................	1	0	S10
....guaaaugcauuuucuggaaugua...................	113	0	S10
....uaaaugcauuuucuggaa.......................	3	0	S10
....uaaaugcauuuucuggaaug.....................	75	0	S10
....uaaaugcauuuucuggaaugu....................	431	0	S10
....uaaaugcauuuucuggaaugua...................	24522	0	S10
....uaaaugcauuuucuggaauguaa..................	23	0	S10
....aaaugcauuuucuggaaugua...................	5	0	S10
....aaugcauuuucuggaaugua...................	1	0	S10
....aagaugaagucagcugccguacc..	1	0	S05
...........................uccguuccagaagugcguuuaca...	1	0	S05
.............................cguuccagaagugcguuuac..	2	0	S05
.............................cguuccagaagugcguuuaca...	3	0	S05
....guaaaugcauuuucuggaaugua...................	26	0	S05
....uaaaugcauuuucuggaa.......................	3	0	S05
....uaaaugcauuuucuggaaug.....................	28	0	S05
....uaaaugcauuuucuggaaugu....................	72	0	S05
....uaaaugcauuuucuggaaugua...................	4753	0	S05
....uaaaugcauuuucuggaauguaa..................	1	0	S05
....aaaugcauuuucuggaaugua...................	1	0	S05

Provisional ID	:	Scaffold633_15234
Score total	:	162.8
Score for star read(s)	:	3.9
Score for read counts	:	156.1
Score for mfe	:	1.2
Score for randfold	:	1.6
Score for cons. seed	:	
Total read count	:	318
Mature read count	:	317
Loop read count	:	0
Star read count	:	1

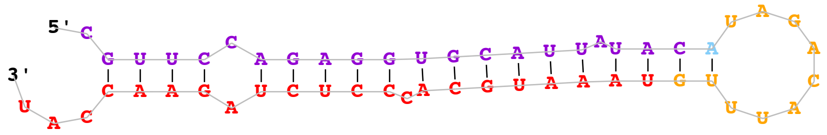

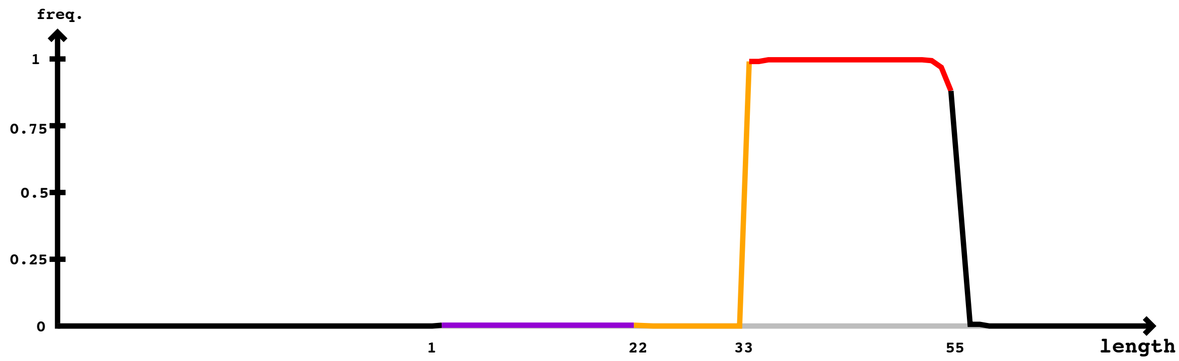


```
                                      Star                          Mature
5'- caugguuaaggauauauuuucuguuucgucuguaucuucguuccagaggugcauuauacauagacauuuguaaaugcacccucuagaaccaugauaugagcgacuaacuucu  -3'   obs
    caugguuaaggauauauuuucuguuucgucuguaucuucguuccagaggugcauuauacauagacauuuguaaaugcacccucuagaaccaugauaugagcgacuaacuucu         exp
    ...((((((((......))))...((((..(((((...((((.((((((((((.((((........)))))))))).))))).))))...)))))..)))).)))))...    reads   mm   sample
    ......................................................................uaaaugcacccucuagaacca.....................    2       0    S05
    ......................................................................uaaaugcacccucuagaaccau....................    8       0    S05

    ......................................cguuccagaggugcauuauac.....................................................    1       0    S10
    ......................................................................uaaaugcacccucuagaacc......................    3       0    S10
    ......................................................................uaaaugcacccucuagaacca.....................    3       0    S10
    ......................................................................uaaaugcacccucuagaaccau....................    30      0    S10

    ......................................................................uaaaugcacccucuagaacca.....................    4       0    S01
    ......................................................................uaaaugcacccucuagaaccau....................    30      0    S01

    ......................................................................uaaaugcacccucuagaacca.....................    3       0    S06
    ......................................................................uaaaugcacccucuagaaccau....................    15      0    S06

    ......................................................................uaaaugcacccucuagaacca.....................    2       0    S07
    ......................................................................uaaaugcacccucuagaaccau....................    41      0    S07

    ......................................................................uaaaugcacccucuagaacc......................    3       0    S03
    ......................................................................uaaaugcacccucuagaacca.....................    3       0    S03
    ......................................................................uaaaugcacccucuagaaccau....................    34      0    S03

    ......................................................................uaaaugcacccucuagaaccau....................    13      0    S09

    ......................................................................uaaaugcacccucuagaacc......................    1       0    S08
    ......................................................................uaaaugcacccucuagaaccau....................    26      0    S08

    ......................................................................uaaaugcacccucuagaac.......................    1       0    S02
    ......................................................................uaaaugcacccucuagaacc......................    1       0    S02
    ......................................................................uaaaugcacccucuagaacca.....................    8       0    S02
    ......................................................................uaaaugcacccucuagaaccau....................    75      0    S02
    .......................................................................aaaugcacccucuagaaccaugau.................    2       0    S02

    ......................................................................uaaaugcacccucuagaacca.....................    3       0    S04
```

```
                                       Star                          Mature
caugguuaaggauauauuuucuguuucgucuguaucuucguuccagaggugcauuauacauagacauuuguaaaugcacccucuagaaccaugauaugagcgacuaacuucu
.......................................................................uaaaugcacccucuagaaccau....................    6    0    S04
```

Provisional ID : Scaffold1260_24209
Score total : 3088.3
Score for star read(s) : 3.9
Score for read counts : 3081
Score for mfe : 1.8
Score for randfold : 1.6
Score for cons. seed :
Total read count : 6055
Mature read count : 6039
Loop read count : 0
Star read count : 16

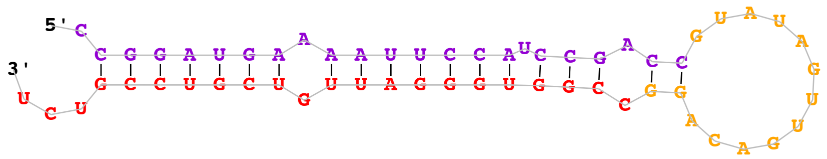

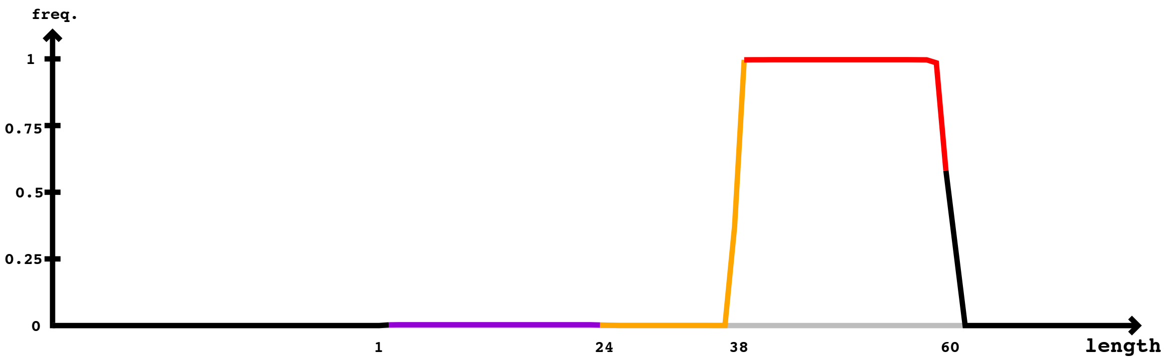


```
                                 Star                                 Mature
5'- uucccuuuuuggauuauaauuucugugaugcaaccggaugaaaauuccauccgaccguauaguugacaggccggugggauugucguccgucugugacacagauuuuauuca   -3'   obs
    uucccuuuuuggauuauaauuucugugaugcaaccggaugaaaauuccauccgaccguauaguugacaggccggugggauugucguccgucugugacacagauuuuauuca         exp
    .(((......)))..((((..((((((.((((..(((((((.(((((((.(((.((............)).)))))))))).)))))))..)))).))))))..))))...   reads   mm   sample
    .....................................................................gccggugggauugucguccguc....................   40      0    S04
    ......................................................................ccggugggauugucguccgu.....................   2       0    S04
    ......................................................................ccggugggauugucguccguc....................   6       0    S04
    ......................................................................ccggugggauugucguccgucu...................   107     0    S04

    .....................................................................gccggugggauugucguccgu.....................   6       0    S02
    .....................................................................gccggugggauugucguccguc....................   28      0    S02
    .....................................................................gccggugggauugucguccgucu...................   1       0    S02
    ......................................................................ccggugggauugucguccgu.....................   1       0    S02
    ......................................................................ccggugggauugucguccguc....................   3       0    S02
    ......................................................................ccggugggauugucguccgucu...................   46      0    S02

    .................................ccggaugaaaauuccauccgac........................................................   3       0    S08
    .................................ccggaugaaaauuccauccgacc.......................................................   6       0    S08
    .................................ccggaugaaaauuccauccgaccguauaguugacagg.........................................   1       0    S08
    .....................................................................gccggugggauugucguccgu.....................   9       0    S08
    .....................................................................gccggugggauugucguccguc....................   844     0    S08
    .....................................................................gccggugggauugucguccgucu...................   2       0    S08
    ......................................................................ccggugggauugucguccgu.....................   4       0    S08
    ......................................................................ccggugggauugucguccguc....................   85      0    S08
    ......................................................................ccggugggauugucguccgucu...................   1005    0    S08
    .........................................................................gugggauugucguccgucu...................   1       0    S08

    .................................ccggaugaaaauuccauccgac........................................................   1       0    S09
    .................................ccggaugaaaauuccauccgacc.......................................................   2       0    S09
    ..................................cggaugaaaauuccauccgac........................................................   1       0    S09
    ..................................cggaugaaaauuccauccgaccguauaguugac............................................   1       0    S09
    ..................................cggaugaaaauuccauccgaccguauaguugacag..........................................   1       0    S09
    .....................................................................gccggugggauugucguccgu.....................   9       0    S09
    .....................................................................gccggugggauugucguccguc....................   287     0    S09
    .....................................................................gccggugggauugucguccgucu...................   1       0    S09
    ......................................................................ccggugggauugucguccg......................   1       0    S09
    ......................................................................ccggugggauugucguccgu.....................   2       0    S09
```

```
                                 Star                             Mature
uucccuuuuuggauuauaauuucugugaugcaaccggaugaaaauuccauccgaccguauaguugacaggccggugggauugucguccgucugugacacagauuuuauuca
......................................................................ccggugggauugucguccguc....................   37   0   S09
......................................................................ccggugggauugucguccgucu...................   617  0   S09

.....................................................................gccggugggauugucguccgu.....................   4    0   S03
.....................................................................gccggugggauugucguccguc....................   39   0   S03
......................................................................ccggugggauugucguccgu.....................   1    0   S03
......................................................................ccggugggauugucguccguc....................   7    0   S03
......................................................................ccggugggauugucguccgucu...................   56   0   S03

.....................................................................gccggugggauugucguccgu.....................   6    0   S07
.....................................................................gccggugggauugucguccguc....................   454  0   S07
......................................................................ccggugggauugucgucc.......................   1    0   S07
......................................................................ccggugggauugucguccguc....................   56   0   S07
......................................................................ccggugggauugucguccgucu...................   544  0   S07

.....................................................................gccggugggauugucguccgu.....................   2    0   S06
.....................................................................gccggugggauugucguccguc....................   143  0   S06
.....................................................................gccggugggauugucguccgucu...................   3    0   S06
......................................................................ccggugggauugucguccguc....................   21   0   S06
......................................................................ccggugggauugucguccgucu...................   312  0   S06

.....................................................................gccggugggauugucguccg......................   1    0   S01
.....................................................................gccggugggauugucguccgu.....................   3    0   S01
.....................................................................gccggugggauugucguccguc....................   53   0   S01
.....................................................................gccggugggauugucguccgucu...................   1    0   S01
......................................................................ccggugggauugucguccgu.....................   2    0   S01
......................................................................ccggugggauugucguccguc....................   8    0   S01
......................................................................ccggugggauugucguccgucu...................   67   0   S01

.................................ccggaugaaaauuccauccgac........................................................   1    0   S10
.................................ccggaugaaaauuccauccgacc.......................................................   2    0   S10
.....................................................................gccggugggauugucguccgu.....................   12   0   S10
.....................................................................gccggugggauugucguccguc....................   262  0   S10
.....................................................................gccggugggauugucguccgucu...................   3    0   S10
......................................................................ccggugggauugucguccgu.....................   2    0   S10
......................................................................ccggugggauugucguccguc....................   39   0   S10
......................................................................ccggugggauugucguccgucu...................   698  0   S10
.......................................................................cggugggauugucguccgucu...................   2    0   S10

.....................................................................gccggugggauugucguccgu.....................   2    0   S05
.....................................................................gccggugggauugucguccguc....................   34   0   S05
......................................................................ccggugggauugucguccguc....................   4    0   S05
......................................................................ccggugggauugucguccgucu...................   53   0   S05
```

Provisional ID : Scaffold605_14471
Score total : 674.8
Score for star read(s) : 3.9
Score for read counts : 668
Score for mfe : 1.3
Score for randfold : 1.6
Score for cons. seed :
Total read count : 1322
Mature read count : 1113
Loop read count : 0
Star read count : 209

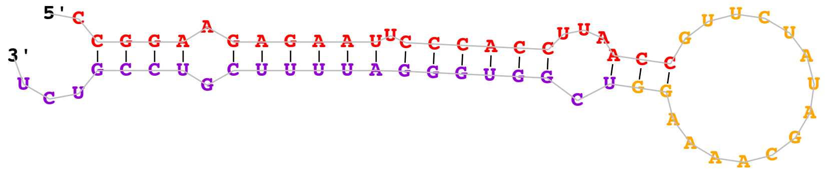

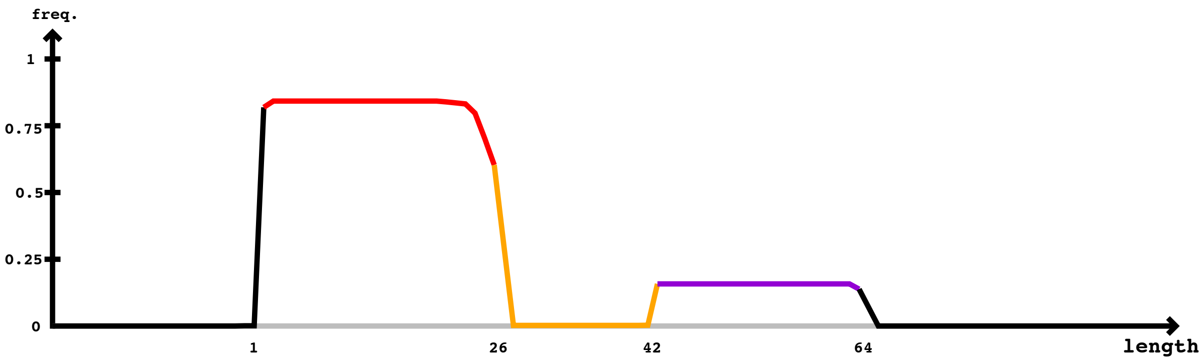

Mature **Star**

```
5'- caaggaaaguuguguugcacccggaagagaauucccaccuuaaccguucuauagcaaaaggucggugggauuuucguccgucuguuacacauuuguaaaucaucuuaagccauuu   -3'    obs
    caaggaaaguuguguugcacccggaagagaauucccaccuuaaccguucuauagcaaaaggucggugggauuuucguccgucuguuacacauuuguaaaucaucuuaagccauuu          exp
    ...((.(((.(((((.(((..((((.((((((.((((((...(((...............))).)))))))))))).))))..))).)))))...........)))...))....   reads   mm   sample
    ....................ccggaagagaauucccaccuu..........................................................................   1       0    S04
    ....................ccggaagagaauucccaccuuaa........................................................................   2       0    S04
    ....................ccggaagagaauucccaccuuaacc......................................................................   11      0    S04
    .............................................................ucggugggauuuucguccguc.................................   2       0    S04
    .............................................................ucggugggauuuucguccgucu................................   12      0    S04

    ....................ccggaagagaauucccacc............................................................................   1       0    S02
    ....................ccggaagagaauucccaccuuaa........................................................................   1       0    S02
    ....................ccggaagagaauucccaccuuaacc......................................................................   7       0    S02
    .....................cggaagagaauucccaccuu..........................................................................   1       0    S02
    .............................................................ucggugggauuuucguccguc.................................   2       0    S02
    .............................................................ucggugggauuuucguccgucu................................   6       0    S02

    ....................ccggaagagaauucccaccuu..........................................................................   1       0    S03
    ....................ccggaagagaauucccaccuua.........................................................................   1       0    S03
    ....................ccggaagagaauucccaccuuaac.......................................................................   1       0    S03
    ....................ccggaagagaauucccaccuuaacc......................................................................   3       0    S03
    .....................cggaagagaauucccaccuuaacc......................................................................   1       0    S03
    .............................................................ucggugggauuuucguccgucu................................   10      0    S03

    ....................ccggaagagaauucccaccu...........................................................................   1       0    S09
    ....................ccggaagagaauucccaccuua.........................................................................   13      0    S09
    ....................ccggaagagaauucccaccuuaa........................................................................   46      0    S09
    ....................ccggaagagaauucccaccuuaac.......................................................................   55      0    S09
    ....................ccggaagagaauucccaccuuaacc......................................................................   386     0    S09
    ....................ccggaagagaauucccaccuuaaccguucuauagcaaaagg......................................................   1       0    S09
    .....................cggaagagaauucccaccuuaa........................................................................   2       0    S09
    .....................cggaagagaauucccaccuuaacc......................................................................   12      0    S09
    .............................................................ucggugggauuuucguccguc.................................   3       0    S09
    .............................................................ucggugggauuuucguccgucu................................   42      0    S09

    ..................acccggaagagaauucccaccu...........................................................................   1       0    S08
    ....................ccggaagagaauucccaccuua.........................................................................   12      0    S08
```

Mature **Star**

```
caaggaaaguuguguugcacccggaagagaauucccaccuuaaccguucuauagcaaaaggucggugggauuuucguccgucuguuacacauuuguaaaucaucuuaagccauuu
....................ccggaagagaauucccaccuuaa........................................................................    20    0    S08
....................ccggaagagaauucccaccuuaac.......................................................................    11    0    S08
....................ccggaagagaauucccaccuuaacc......................................................................    47    0    S08
....................ccggaagagaauucccaccuuaaccguucuauagcaaaagg......................................................    1     0    S08
.....................cggaagagaauucccaccuuaac.......................................................................    1     0    S08
.....................cggaagagaauucccaccuuaacc......................................................................    1     0    S08
.............................................................ucggugggauuuucguccguc.................................    1     0    S08
.............................................................ucggugggauuuucguccgucu................................    16    0    S08

....................ccggaagagaauucccacc............................................................................    2     0    S06
....................ccggaagagaauucccaccuua.........................................................................    2     0    S06
....................ccggaagagaauucccaccuuaacc......................................................................    8     0    S06
............................................................gucggugggauuuucguccguc.................................    1     0    S06
.............................................................ucggugggauuuucguccguc.................................    4     0    S06
.............................................................ucggugggauuuucguccgucu................................    3     0    S06

....................ccggaagagaauucccaccuua.........................................................................    1     0    S01
....................ccggaagagaauucccaccuuaa........................................................................    1     0    S01
.............................................................ucggugggauuuucguccguc.................................    1     0    S01
.............................................................ucggugggauuuucguccgucu................................    13    0    S01

....................ccggaagagaauucccaccu...........................................................................    2     0    S07
....................ccggaagagaauucccaccuu..........................................................................    1     0    S07
....................ccggaagagaauucccaccuua.........................................................................    5     0    S07
....................ccggaagagaauucccaccuuaa........................................................................    3     0    S07
....................ccggaagagaauucccaccuuaac.......................................................................    5     0    S07
....................ccggaagagaauucccaccuuaacc......................................................................    30    0    S07
.............................................................ucggugggauuuucguccguc.................................    4     0    S07
.............................................................ucggugggauuuucguccgucu................................    16    0    S07

....................ccggaagagaauucccacc............................................................................    1     0    S05
....................ccggaagagaauucccaccuu..........................................................................    1     0    S05
....................ccggaagagaauucccaccuuaac.......................................................................    5     0    S05
....................ccggaagagaauucccaccuuaacc......................................................................    9     0    S05
.............................................................ucggugggauuuucguccguc.................................    3     0    S05
.............................................................ucggugggauuuucguccgucu................................    11    0    S05

....................ccggaagagaauucccaccuua.........................................................................    11    0    S10
....................ccggaagagaauucccaccuuaa........................................................................    48    0    S10
....................ccggaagagaauucccaccuuaac.......................................................................    54    0    S10
....................ccggaagagaauucccaccuuaacc......................................................................    273   0    S10
.....................cggaagagaauucccaccu...........................................................................    1     0    S10
.....................cggaagagaauucccaccuua.........................................................................    1     0    S10
.....................cggaagagaauucccaccuuaa........................................................................    1     0    S10
.....................cggaagagaauucccaccuuaac.......................................................................    1     0    S10
.....................cggaagagaauucccaccuuaacc......................................................................    8     0    S10
.....................cggaagagaauucccaccuuaaccguucuauagcaaaagg......................................................    1     0    S10
.............................................................ucggugggauuuucguccguc.................................    5     0    S10
.............................................................ucggugggauuuucguccgucu................................    54    0    S10
```

Provisional ID	:	Scaffold248_6742
Score total	:	819389.2
Score for star read(s)	:	3.9
Score for read counts	:	819382.4
Score for mfe	:	1.3
Score for randfold	:	1.6
Score for cons. seed	:	
Total read count	:	1607195
Mature read count	:	1606994
Loop read count	:	0
Star read count	:	201

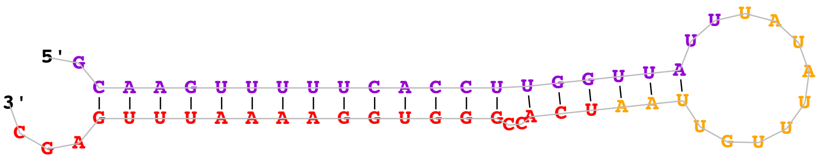

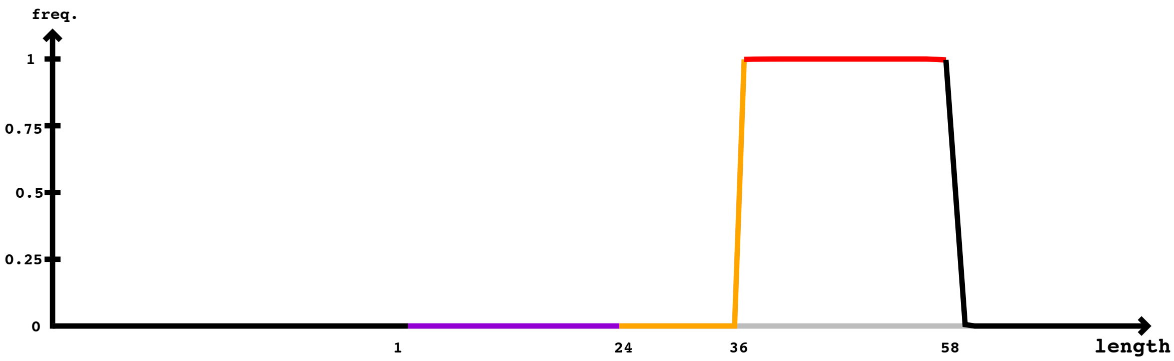


```
                                          Star                                  Mature
5'- aaauccaucaagucuggagaaaucuuucuucuagugcaaguuuuucaccuugguuauuuauauuuguuaaucaccggguggaaaauuugagcuugaagacggaucuaucauc   -3'   obs
    aaauccaucaagucuggagaaaucuuucuucuagugcaaguuuuucaccuugguuauuuauauuuguuaaucaccggguggaaaauuugagcuugaagacggaucuaucauc         exp
    ...((((.......))))...((((.(((((.(((.((((((((((((((((((((...........))))))..)))))))))))))).))).))))).))))........   reads   mm   sample
    .......ucaagucuggagaaaucuuucuucuagu.............................................................................   1       0    S05
    .............cuggagaaaucuuucuucuagu.............................................................................   1       0    S05
    ...............................uagugcaaguuuuucaccuug............................................................   1       0    S05
    ...............................uagugcaaguuuuucaccuugguu.........................................................   1       0    S05
    ...................................gcaaguuuuucaccuuggu..........................................................   2       0    S05
    ...................................gcaaguuuuucaccuugguu.........................................................   3       0    S05
    ...................................gcaaguuuuucaccuugguuauu......................................................   27      0    S05
    .....................................................uuauuuauauuuguuaaucaccgggu.................................   3       0    S05
    .........................................................uuauauuuguuaaucaccgggu.................................   1       0    S05
    ..........................................................uauauuuguuaaucaccggguggaaaauuugagc....................   10      0    S05
    ..........................................................uauauuuguuaaucaccggguggaaaauuugagcu...................   1       0    S05
    ...........................................................auauuuguuaaucaccggguggaaaauuugagc....................   2       0    S05
    ...................................................................uaaucaccggguggaaaauuugagc....................   1       0    S05
    ....................................................................aaucaccggguggaaaauuugagc....................   1       0    S05
    .....................................................................aucaccggguggaaaauuugagc....................   14      0    S05
    .....................................................................aucaccggguggaaaauuugagcu...................   1       0    S05
    ......................................................................ucaccggguggaaaauuug.......................   2       0    S05
    ......................................................................ucaccggguggaaaauuuga......................   108     0    S05
    ......................................................................ucaccggguggaaaauuugag.....................   199     0    S05
    ......................................................................ucaccggguggaaaauuugagc....................   179283  0    S05
    ......................................................................ucaccggguggaaaauuugagcu...................   14569   0    S05
    ......................................................................ucaccggguggaaaauuugagcuu..................   1174    0    S05
    ......................................................................ucaccggguggaaaauuugagcuug.................   1       0    S05
    .......................................................................caccggguggaaaauuugagc....................   169     0    S05
    .......................................................................caccggguggaaaauuugagcu...................   47      0    S05
    .......................................................................caccggguggaaaauuugagcuu..................   22      0    S05
    ........................................................................accggguggaaaauuugagc....................   45      0    S05
    ........................................................................accggguggaaaauuugagcuuga................   1       0    S05
    .........................................................................ccggguggaaaauuugagc....................   37      0    S05
    .........................................................................ccggguggaaaauuugagcuug.................   1       0    S05
    ..........................................................................cggguggaaaauuugagc....................   14      0    S05
    ...........................................................................ggguggaaaauuugagcu...................   2       0    S05

    ...................................gcaaguuuuucaccuugguu.........................................................   2       0    S10
```

Star **Mature**

```
aaauccaucaagucuggagaaaucuuucuucuagugcaaguuuuucaccuugguuauuuauauuuguuaaucaccggguggaaaauuugagcuugaagacggaucuaucauc
...................................gcaaguuuuucaccuugguuau.......................................................   3       0   S10
...................................gcaaguuuuucaccuugguuauu......................................................   19      0   S10
.....................................................................aucaccggguggaaaauuugagc....................   4       0   S10
.....................................................................aucaccggguggaaaauuugagcu...................   1       0   S10
......................................................................ucaccggguggaaaauuug.......................   3       0   S10
......................................................................ucaccggguggaaaauuuga......................   443     0   S10
......................................................................ucaccggguggaaaauuugag.....................   457     0   S10
......................................................................ucaccggguggaaaauuugagc....................   70893   0   S10
......................................................................ucaccggguggaaaauuugagcu...................   7795    0   S10
......................................................................ucaccggguggaaaauuugagcuu..................   302     0   S10
.......................................................................caccggguggaaaauuugagc....................   49      0   S10
.......................................................................caccggguggaaaauuugagcu...................   13      0   S10
.......................................................................caccggguggaaaauuugagcuu..................   5       0   S10
........................................................................accggguggaaaauuugagc....................   16      0   S10
........................................................................accggguggaaaauuugagcu...................   2       0   S10
.........................................................................ccggguggaaaauuugagc....................   6       0   S10
..........................................................................cggguggaaaauuugagc....................   2       0   S10
..........................................................................cggguggaaaauuugagcu...................   1       0   S10
..........................................................................cggguggaaaauuugagcuuga................   1       0   S10

......................................................................ucaccggguggaaaauuuga......................   8       0   S01
......................................................................ucaccggguggaaaauuugag.....................   15      0   S01
......................................................................ucaccggguggaaaauuugagc....................   6950    0   S01
......................................................................ucaccggguggaaaauuugagcu...................   152     0   S01
......................................................................ucaccggguggaaaauuugagcuu..................   6       0   S01
.......................................................................caccggguggaaaauuugagc....................   2       0   S01
.......................................................................caccggguggaaaauuugagcu...................   4       0   S01
........................................................................accggguggaaaauuugagc....................   1       0   S01
.........................................................................ccggguggaaaauuugag.....................   1       0   S01
.........................................................................ccggguggaaaauuugagc....................   3       0   S01
.........................................................................ccggguggaaaauuugagcu...................   1       0   S01
..........................................................................cggguggaaaauuugagc....................   1       0   S01

...............................uagugcaaguuuuucaccuuggu..........................................................   1       0   S06
...................................gcaaguuuuucaccuuggu..........................................................   1       0   S06
...................................gcaaguuuuucaccuugguuau.......................................................   2       0   S06
...................................gcaaguuuuucaccuugguuauu......................................................   15      0   S06
..........................................................uauauuuguuaaucaccggguggaaaauuugagc....................   9       0   S06
...........................................................auauuuguuaaucaccggguggaaaauuugagc....................   2       0   S06
....................................................................aaucaccggguggaaaauuugagc....................   3       0   S06
.....................................................................aucaccggguggaaaauuugagc....................   5       0   S06
......................................................................ucaccggguggaaaauuug.......................   6       0   S06
......................................................................ucaccggguggaaaauuuga......................   256     0   S06
......................................................................ucaccggguggaaaauuugag.....................   340     0   S06
......................................................................ucaccggguggaaaauuugagc....................   223149  0   S06
......................................................................ucaccggguggaaaauuugagcu...................   19204   0   S06
......................................................................ucaccggguggaaaauuugagcuu..................   1228    0   S06
.......................................................................caccggguggaaaauuugagc....................   152     0   S06
.......................................................................caccggguggaaaauuugagcu...................   36      0   S06
.......................................................................caccggguggaaaauuugagcuu..................   15      0   S06
........................................................................accggguggaaaauuugagc....................   53      0   S06
........................................................................accggguggaaaauuugagcu...................   7       0   S06
........................................................................accggguggaaaauuugagcuuga................   1       0   S06
.........................................................................ccggguggaaaauuugagc....................   62      0   S06
.........................................................................ccggguggaaaauuugagcu...................   5       0   S06
..........................................................................cggguggaaaauuugagc....................   29      0   S06
...........................................................................ggguggaaaauuugagcu...................   5       0   S06

...............................uagugcaaguuuuucaccuuggu..........................................................   1       0   S07
...................................gcaaguuuuucaccuugg...........................................................   2       0   S07
...................................gcaaguuuuucaccuuggu..........................................................   3       0   S07
...................................gcaaguuuuucaccuugguu.........................................................   1       0   S07
...................................gcaaguuuuucaccuugguuau.......................................................   2       0   S07
...................................gcaaguuuuucaccuugguuauu......................................................   22      0   S07
..........................................................uauauuuguuaaucaccggguggaaaauuugagc....................   5       0   S07
...........................................................auauuuguuaaucaccggguggaaaauuugagc....................   3       0   S07
.....................................................................aucaccggguggaaaauuugagc....................   3       0   S07
......................................................................ucaccggguggaaaauuu........................   2       0   S07
......................................................................ucaccggguggaaaauuug.......................   9       0   S07
......................................................................ucaccggguggaaaauuuga......................   505     0   S07
```

Star **Mature**

```
aaauccaucaagucuggagaaaucuuucuucuagugcaaguuuuucaccuugguuauuuauauuuguuaaucaccggguggaaaauuugagcuugaagacggaucuaucauc
......................................................................ucaccggguggaaaauuugag.....................  568     0  S07
......................................................................ucaccggguggaaaauuugagc....................  288367  0  S07
......................................................................ucaccggguggaaaauuugagcu...................  31839   0  S07
......................................................................ucaccggguggaaaauuugagcuu..................  1640    0  S07
.......................................................................caccggguggaaaauuugag.....................  1       0  S07
.......................................................................caccggguggaaaauuugagc....................  206     0  S07
.......................................................................caccggguggaaaauuugagcu...................  48      0  S07
.......................................................................caccggguggaaaauuugagcuu..................  12      0  S07
........................................................................accggguggaaaauuugagc....................  77      0  S07
........................................................................accggguggaaaauuugagcu...................  12      0  S07
.........................................................................ccggguggaaaauuugagc....................  46      0  S07
.........................................................................ccggguggaaaauuugagcu...................  4       0  S07
..........................................................................cggguggaaaauuugagc....................  22      0  S07
...........................................................................ggguggaaaauuugagcu...................  6       0  S07

...............................uagugcaaguuuuucaccuugguu.........................................................  1       0  S09
...................................gcaaguuuuucaccuugguu.........................................................  1       0  S09
...................................gcaaguuuuucaccuugguuau.......................................................  2       0  S09
...................................gcaaguuuuucaccuugguuauu......................................................  16      0  S09
..........................................................uauauuuguuaaucaccgggug................................  1       0  S09
..........................................................uauauuuguuaaucaccggguggaaaauuugagc....................  1       0  S09
...........................................................auauuuguuaaucaccggguggaaaauuugagc....................  1       0  S09
......................................................................ucaccggguggaaaauuu........................  2       0  S09
......................................................................ucaccggguggaaaauuug.......................  4       0  S09
......................................................................ucaccggguggaaaauuuga......................  318     0  S09
......................................................................ucaccggguggaaaauuugag.....................  293     0  S09
......................................................................ucaccggguggaaaauuugagc....................  65446   0  S09
......................................................................ucaccggguggaaaauuugagcu...................  7886    0  S09
......................................................................ucaccggguggaaaauuugagcuu..................  281     0  S09
.......................................................................caccggguggaaaauuugag.....................  1       0  S09
.......................................................................caccggguggaaaauuugagc....................  60      0  S09
.......................................................................caccggguggaaaauuugagcu...................  12      0  S09
.......................................................................caccggguggaaaauuugagcuu..................  2       0  S09
........................................................................accggguggaaaauuugagc....................  18      0  S09
........................................................................accggguggaaaauuugagcu...................  1       0  S09
.........................................................................ccggguggaaaauuugagc....................  3       0  S09
..........................................................................cggguggaaaauuugagc....................  2       0  S09
...........................................................................ggguggaaaauuugagcu...................  1       0  S09

................gagaaaucuuucuucuag..............................................................................  1       0  S03
...................................gcaaguuuuucaccuugguu.........................................................  1       0  S03
...................................gcaaguuuuucaccuugguuauu......................................................  20      0  S03
.....................................................uuauuuauauuuguuaaucaccgggug................................  1       0  S03
........................................................uuuauauuuguuaaucaccgggu..................................  1       0  S03
..........................................................uauauuuguuaaucaccggguggaaaauuugagc....................  22      0  S03
..................................................................uuaaucaccggguggaaaauuugagc....................  1       0  S03
.....................................................................aucaccggguggaaaauuugagc....................  3       0  S03
......................................................................ucaccggguggaaaauuug.......................  4       0  S03
......................................................................ucaccggguggaaaauuuga......................  94      0  S03
......................................................................ucaccggguggaaaauuugag.....................  197     0  S03
......................................................................ucaccggguggaaaauuugagc....................  172706  0  S03
......................................................................ucaccggguggaaaauuugagcu...................  12023   0  S03
......................................................................ucaccggguggaaaauuugagcuu..................  903     0  S03
......................................................................ucaccggguggaaaauuugagcuug.................  1       0  S03
.......................................................................caccggguggaaaauuuga......................  1       0  S03
.......................................................................caccggguggaaaauuugagc....................  131     0  S03
.......................................................................caccggguggaaaauuugagcu...................  26      0  S03
.......................................................................caccggguggaaaauuugagcuu..................  5       0  S03
........................................................................accggguggaaaauuugag.....................  1       0  S03
........................................................................accggguggaaaauuugagc....................  55      0  S03
........................................................................accggguggaaaauuugagcu...................  1       0  S03
........................................................................accggguggaaaauuugagcuu..................  1       0  S03
........................................................................accggguggaaaauuugagcuug.................  1       0  S03
........................................................................accggguggaaaauuugagcuuga................  1       0  S03
.........................................................................ccggguggaaaauuugagc....................  35      0  S03
.........................................................................ccggguggaaaauuugagcu...................  3       0  S03
..........................................................................cggguggaaaauuugagc....................  14      0  S03
..........................................................................cggguggaaaauuugagcuu..................  1       0  S03
..........................................................................cggguggaaaauuugagcuuga................  1       0  S03
...........................................................................ggguggaaaauuugagcu...................  1       0  S03
```

```
                                   Star                             Mature
aaauccaucaagucuggagaaaucuuucuucuagugcaaguuuuucaccuugguuauuuauauuuguuaaucaccggguggaaaauuugagcuugaagacggaucuaucauc
...........................................................................ggguggaaaauuugagcuu..................  1       0  S03

...................................gcaaguuuuucaccuugguua........................................................  3       0  S08
...................................gcaaguuuuucaccuugguuauu......................................................  18      0  S08
.................................................uugguuauuuauauuuguuaauc........................................  2       0  S08
.................................................................guuaaucaccggguggaaaauuugagc....................  1       0  S08
...................................................................uaaucaccggguggaaaauuugagc....................  1       0  S08
.....................................................................aucaccggguggaaaauuugagc....................  2       0  S08
......................................................................ucaccggguggaaaauuug.......................  5       0  S08
......................................................................ucaccggguggaaaauuuga......................  580     0  S08
......................................................................ucaccggguggaaaauuugag.....................  614     0  S08
......................................................................ucaccggguggaaaauuugagc....................  218427  0  S08
......................................................................ucaccggguggaaaauuugagcu...................  26187   0  S08
......................................................................ucaccggguggaaaauuugagcuu..................  1717    0  S08
.......................................................................caccggguggaaaauuuga......................  1       0  S08
.......................................................................caccggguggaaaauuugag.....................  1       0  S08
.......................................................................caccggguggaaaauuugagc....................  163     0  S08
.......................................................................caccggguggaaaauuugagcu...................  34      0  S08
.......................................................................caccggguggaaaauuugagcuu..................  13      0  S08
........................................................................accggguggaaaauuugagc....................  59      0  S08
........................................................................accggguggaaaauuugagcu...................  9       0  S08
........................................................................accggguggaaaauuugagcuu..................  1       0  S08
.........................................................................ccggguggaaaauuugagc....................  70      0  S08
.........................................................................ccggguggaaaauuugagcu...................  5       0  S08
.........................................................................ccggguggaaaauuugagcuu..................  2       0  S08
..........................................................................cggguggaaaauuugagc....................  31      0  S08
..........................................................................cggguggaaaauuugagcu...................  2       0  S08
...........................................................................ggguggaaaauuugagcu...................  5       0  S08

...................................gcaaguuuuucaccuugguu.........................................................  1       0  S02
...................................gcaaguuuuucaccuugguuauu......................................................  1       0  S02
......................................................................ucaccggguggaaaauuu........................  1       0  S02
......................................................................ucaccggguggaaaauuug.......................  2       0  S02
......................................................................ucaccggguggaaaauuuga......................  8       0  S02
......................................................................ucaccggguggaaaauuugag.....................  12      0  S02
......................................................................ucaccggguggaaaauuugagc....................  9300    0  S02
......................................................................ucaccggguggaaaauuugagcu...................  413     0  S02
......................................................................ucaccggguggaaaauuugagcuu..................  27      0  S02
.......................................................................caccggguggaaaauuugagc....................  7       0  S02
.......................................................................caccggguggaaaauuugagcu...................  4       0  S02
........................................................................accggguggaaaauuugagc....................  2       0  S02
........................................................................accggguggaaaauuugagcuu..................  1       0  S02
.........................................................................ccggguggaaaauuugagc....................  3       0  S02
..........................................................................cggguggaaaauuugagc....................  1       0  S02
..........................................................................cggguggaaaauuugagcuuga................  1       0  S02

...............................uagugcaaguuuuucaccuuggu..........................................................  1       0  S04
................................agugcaaguuuuucaccuugguu.........................................................  1       0  S04
...................................gcaaguuuuucaccuugguuau.......................................................  1       0  S04
...................................gcaaguuuuucaccuugguuauu......................................................  33      0  S04
.....................................................uuauuuauauuuguuaaucaccgggu.................................  1       0  S04
..........................................................uauauuuguuaaucaccggguggaaaauuugagc....................  17      0  S04
..........................................................uauauuuguuaaucaccggguggaaaauuugagcuu..................  1       0  S04
............................................................uauuuguuaaucaccgggug................................  1       0  S04
....................................................................aaucaccggguggaaaauuugagcu...................  2       0  S04
.....................................................................aucaccggguggaaaauuugagc....................  4       0  S04
......................................................................ucaccggguggaaaauuu........................  1       0  S04
......................................................................ucaccggguggaaaauuug.......................  2       0  S04
......................................................................ucaccggguggaaaauuuga......................  154     0  S04
......................................................................ucaccggguggaaaauuugag.....................  269     0  S04
......................................................................ucaccggguggaaaauuugagc....................  220819  0  S04
......................................................................ucaccggguggaaaauuugagcu...................  15149   0  S04
......................................................................ucaccggguggaaaauuugagcuu..................  1255    0  S04
.......................................................................caccggguggaaaauuugagc....................  154     0  S04
.......................................................................caccggguggaaaauuugagcu...................  35      0  S04
.......................................................................caccggguggaaaauuugagcuu..................  15      0  S04
........................................................................accggguggaaaauuugagc....................  56      0  S04
........................................................................accggguggaaaauuugagcu...................  1       0  S04
........................................................................accggguggaaaauuugagcuug.................  1       0  S04
........................................................................accggguggaaaauuugagcuuga................  2       0  S04
```

```
                                   Star                   Mature
aaauccaucaagucuggagaaaucuuucuucuagugcaaguuuuucaccuugguuauuuauauuuguuaaucaccggguggaaaauuugagcuugaagacggaucuaucauc
.........................................................................ccggguggaaaauuugagc....................   45   0   S04
.........................................................................ccggguggaaaauuugagcu...................   3    0   S04
..........................................................................cggguggaaaauuugagc....................   19   0   S04
..........................................................................cggguggaaaauuugagcu...................   1    0   S04
...........................................................................ggguggaaaauuugagcu...................   5    0   S04
...........................................................................ggguggaaaauuugagcuu..................   2    0   S04
```

Provisional ID	:	Scaffold237_6201
Score total	:	397538.7
Score for star read(s)	:	3.9
Score for read counts	:	397531.2
Score for mfe	:	2.1
Score for randfold	:	1.6
Score for cons. seed	:	
Total read count	:	779752
Mature read count	:	773491
Loop read count	:	2
Star read count	:	6259

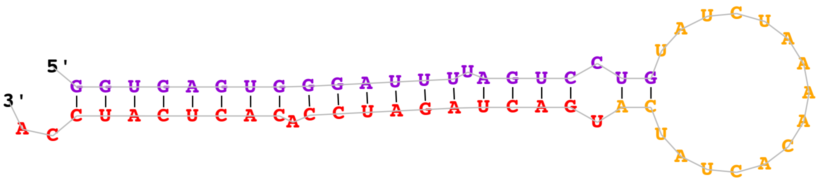

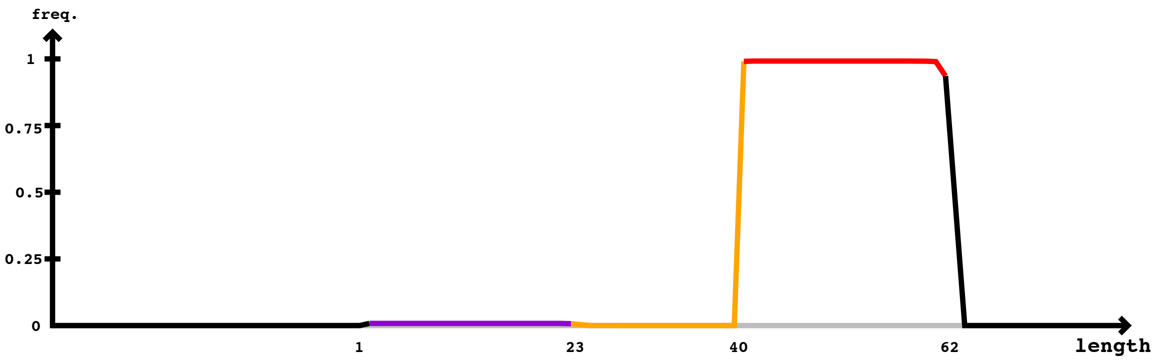


```
                                 Star                                  Mature
5'- guugcugugauuguuuucucuuccaugugugggugagugggauuuuaguccuguaucuaaaacacuaucaugacuagauccacacucauccacgcugggagugacuuuca  -3'   obs
    guugcugugauuguuuucucuuccaugugugggugagugggauuuuaguccuguaucuaaaacacuaucaugacuagauccacacucauccacgcugggagugacuuuca         exp
    .......(((..(((....((((((.(((((((((((((((((((.((((.((...............)).)))))))))).))))))))))))))))))).))).)))   reads   mm   sample
    ..ugcugugauuguuuucucuuccaugugug...............................................................................   1       0    S05
    ..............................gggugagugggauuuuaguccug.........................................................   2       0    S05
    ...............................ggugagugggauuuuaguc............................................................   1       0    S05
    ...............................ggugagugggauuuuagucc...........................................................   13      0    S05
    ...............................ggugagugggauuuuaguccu..........................................................   19      0    S05
    ...............................ggugagugggauuuuaguccug.........................................................   319     0    S05
    ...............................ggugagugggauuuuaguccuguauc.....................................................   3       0    S05
    ...............................ggugagugggauuuuaguccuguaucuaaaacacu............................................   1       0    S05
    ..................................gagugggauuuuaguccug.........................................................   1       0    S05
    .............................................uaguccuguaucuaaaacacua...........................................   1       0    S05
    ................................................uccuguaucuaaaacacuauca........................................   1       0    S05
    ......................................................................ugacuagauccacacuca......................   10      0    S05
    ......................................................................ugacuagauccacacucau.....................   14      0    S05
    ......................................................................ugacuagauccacacucauc....................   73      0    S05
    ......................................................................ugacuagauccacacucaucc...................   1143    0    S05
    ......................................................................ugacuagauccacacucaucca..................   17247   0    S05
    ......................................................................ugacuagauccacacucauccac.................   76      0    S05
    .......................................................................gacuagauccacacucaucc...................   1       0    S05
    .......................................................................gacuagauccacacucaucca..................   26      0    S05
    ..............................................................................uccacacucauccacgcug.............   1       0    S05

    ..............................gggugagugggauuuuaguccu..........................................................   2       0    S10
    ..............................gggugagugggauuuuaguccug.........................................................   3       0    S10
    ...............................ggugagugggauuuuaguc............................................................   1       0    S10
    ...............................ggugagugggauuuuagucc...........................................................   8       0    S10
    ...............................ggugagugggauuuuaguccu..........................................................   275     0    S10
    ...............................ggugagugggauuuuaguccug.........................................................   1678    0    S10
    ...............................ggugagugggauuuuaguccugu........................................................   4       0    S10
    ...............................ggugagugggauuuuaguccuguauc.....................................................   5       0    S10
    ...............................ggugagugggauuuuaguccuguaucu....................................................   2       0    S10
    ...............................ggugagugggauuuuaguccuguaucuaaaacacu............................................   5       0    S10
    ...............................ggugagugggauuuuaguccuguaucuaaaacacuauc.........................................   6       0    S10
    ...............................ggugagugggauuuuaguccuguaucuaaaacacuauca........................................   7       0    S10
```

```
                                 Star                                          Mature
guugcugugauuguuuucucuuccaugugugggugagugggauuuuaguccuguaucuaaaacacuaucaugacuagauccacacucauccacgcugggagugacuuuca
.................................ugagugggauuuuagucc...........................................................   1        0    S10
............................................uuaguccuguaucuaaaacacu............................................   2        0    S10
............................................uuaguccuguaucuaaaacacua...........................................   3        0    S10
.....................................................uaucuaaaacacuaucaugacu...................................   2        0    S10
.....................................................uaucuaaaacacuaucaugacuagauccacacucaucca..................   3        0    S10
.................................................................uaucaugacuagauccacacu........................   1        0    S10
...................................................................ucaugacuagauccacacucaucca..................   1        0    S10
....................................................................caugacuagauccacacucaucca..................   1        0    S10
.....................................................................augacuagauccacacucaucc...................   1        0    S10
.....................................................................augacuagauccacacucaucca..................   1        0    S10
......................................................................ugacuagauccacacuca......................   17       0    S10
......................................................................ugacuagauccacacucau.....................   42       0    S10
......................................................................ugacuagauccacacucauc....................   208      0    S10
......................................................................ugacuagauccacacucaucc...................   10910    0    S10
......................................................................ugacuagauccacacucaucca..................   172847   0    S10
......................................................................ugacuagauccacacucauccac.................   393      0    S10
......................................................................ugacuagauccacacucauccacg................   5        0    S10
.......................................................................gacuagauccacacucaucc...................   7        0    S10
.......................................................................gacuagauccacacucaucca..................   151      0    S10
........................................................................acuagauccacacucaucca..................   1        0    S10
.........................................................................cuagauccacacucaucca..................   3        0    S10
..........................................................................uagauccacacucaucca..................   2        0    S10
............................................................................................cgcugggagugacuuuca   1        0    S10

...............................ggugagugggauuuuaguccug.........................................................   5        0    S01
......................................................................ugacuagauccacacuca......................   2        0    S01
......................................................................ugacuagauccacacucau.....................   10       0    S01
......................................................................ugacuagauccacacucauc....................   36       0    S01
......................................................................ugacuagauccacacucaucc...................   890      0    S01
......................................................................ugacuagauccacacucaucca..................   2246     0    S01
......................................................................ugacuagauccacacucauccac.................   14       0    S01
......................................................................ugacuagauccacacucauccacg................   1        0    S01
.......................................................................gacuagauccacacucauc....................   1        0    S01
.......................................................................gacuagauccacacucaucca..................   1        0    S01

...gcugugauuguuuucucuuccaugugug...............................................................................   1        0    S06
...............................ggugagugggauuuuagucc...........................................................   35       0    S06
...............................ggugagugggauuuuaguccu..........................................................   33       0    S06
...............................ggugagugggauuuuaguccug.........................................................   619      0    S06
...............................ggugagugggauuuuaguccugu........................................................   2        0    S06
...............................ggugagugggauuuuaguccuguauc.....................................................   4        0    S06
...............................ggugagugggauuuuaguccuguaucu....................................................   1        0    S06
...............................ggugagugggauuuuaguccuguaucuaaaacacu............................................   2        0    S06
............................................uuaguccuguaucuaaaacacu............................................   1        0    S06
.............................................uaguccuguaucuaaaacacu............................................   1        0    S06
......................................................................ugacuagauccacacuca......................   6        0    S06
......................................................................ugacuagauccacacucau.....................   13       0    S06
......................................................................ugacuagauccacacucauc....................   111      0    S06
......................................................................ugacuagauccacacucaucc...................   1902     0    S06
......................................................................ugacuagauccacacucaucca..................   27212    0    S06
......................................................................ugacuagauccacacucauccac.................   204      0    S06
......................................................................ugacuagauccacacucauccacg................   1        0    S06
.......................................................................gacuagauccacacucaucca..................   27       0    S06

..ugcugugauuguuuucucuuccaugugug...............................................................................   2        0    S07
...gcugugauuguuuucucuuccaugugug...............................................................................   1        0    S07
......gugauuguuuucucuuccaugugug...............................................................................   1        0    S07
..............................gggugagugggauuuuaguccug.........................................................   5        0    S07
...............................ggugagugggauuuuaguc............................................................   2        0    S07
...............................ggugagugggauuuuagucc...........................................................   19       0    S07
...............................ggugagugggauuuuaguccu..........................................................   111      0    S07
...............................ggugagugggauuuuaguccug.........................................................   734      0    S07
...............................ggugagugggauuuuaguccuguauc.....................................................   8        0    S07
...............................ggugagugggauuuuaguccuguaucuaaaac...............................................   1        0    S07
...............................ggugagugggauuuuaguccuguaucuaaaacacu............................................   7        0    S07
...............................ggugagugggauuuuaguccuguaucuaaaacacuauc.........................................   6        0    S07
...............................ggugagugggauuuuaguccuguaucuaaaacacuauca........................................   1        0    S07
.................................ugagugggauuuuaguccuguauc.....................................................   1        0    S07
...................................agugggauuuuaguccug.........................................................   1        0    S07
............................................uuaguccuguaucuaaaacacu............................................   1        0    S07
```

Star **Mature**

```
guugcugugauuguuuucucuuccaugugugggugagugggauuuuaguccuguaucuaaaacacuaucaugacuagauccacacucauccacgcugggagugacuuuca
.....................................................uaucuaaaacacuaucaugacuagauccacacucaucca..................  3       0  S07
.....................................................................augacuagauccacacucaucca..................  1       0  S07
......................................................................ugacuagauccacacuca......................  5       0  S07
......................................................................ugacuagauccacacucau.....................  27      0  S07
......................................................................ugacuagauccacacucauc....................  112     0  S07
......................................................................ugacuagauccacacucaucc...................  5089    0  S07
......................................................................ugacuagauccacacucaucca..................  136048  0  S07
......................................................................ugacuagauccacacucauccac.................  439     0  S07
......................................................................ugacuagauccacacucauccacg................  2       0  S07
.......................................................................gacuagauccacacucaucc...................  2       0  S07
.......................................................................gacuagauccacacucaucca..................  118     0  S07
.......................................................................gacuagauccacacucauccac.................  1       0  S07
.........................................................................cuagauccacacucaucca..................  3       0  S07
..........................................................................uagauccacacucaucca..................  2       0  S07

....cugugauuguuuucucuuccaugugug...............................................................................  1       0  S09
.....ugugauuguuuucucuuccaugugug...............................................................................  1       0  S09
..............................gggugagugggauuuuaguccug.........................................................  6       0  S09
...............................ggugagugggauuuuaguc............................................................  1       0  S09
...............................ggugagugggauuuuagucc...........................................................  8       0  S09
...............................ggugagugggauuuuaguccu..........................................................  258     0  S09
...............................ggugagugggauuuuaguccug.........................................................  842     0  S09
...............................ggugagugggauuuuaguccugu........................................................  2       0  S09
...............................ggugagugggauuuuaguccugua.......................................................  1       0  S09
...............................ggugagugggauuuuaguccuguauc.....................................................  5       0  S09
...............................ggugagugggauuuuaguccuguaucu....................................................  2       0  S09
...............................ggugagugggauuuuaguccuguaucuaaaacacu............................................  1       0  S09
...............................ggugagugggauuuuaguccuguaucuaaaacacuau..........................................  1       0  S09
...............................ggugagugggauuuuaguccuguaucuaaaacacuauc.........................................  5       0  S09
...............................ggugagugggauuuuaguccuguaucuaaaacacuauca........................................  3       0  S09
................................gugagugggauuuuaguccu..........................................................  1       0  S09
................................gugagugggauuuuaguccug.........................................................  1       0  S09
.................................ugagugggauuuuaguccug.........................................................  1       0  S09
..............................................aguccuguaucuaaaacacua...........................................  1       0  S09
.....................................................uaucuaaaacacuaucaugacuagauccacacucaucca..................  1       0  S09
...............................................................acuaucaugacuagauccacacucaucca..................  1       0  S09
...................................................................ucaugacuagauccacacucaucca..................  2       0  S09
.....................................................................augacuagauccacacucaucca..................  1       0  S09
......................................................................ugacuagauccacacuca......................  47      0  S09
......................................................................ugacuagauccacacucau.....................  51      0  S09
......................................................................ugacuagauccacacucauc....................  177     0  S09
......................................................................ugacuagauccacacucaucc...................  9053    0  S09
......................................................................ugacuagauccacacucaucca..................  182168  0  S09
......................................................................ugacuagauccacacucauccac.................  328     0  S09
......................................................................ugacuagauccacacucauccacg................  4       0  S09
.......................................................................gacuagauccacacucaucc...................  13      0  S09
.......................................................................gacuagauccacacucaucca..................  148     0  S09
.......................................................................gacuagauccacacucauccac.................  1       0  S09
........................................................................acuagauccacacucaucca..................  2       0  S09
.........................................................................cuagauccacacucaucca..................  8       0  S09
..........................................................................uagauccacacucaucca..................  2       0  S09

...............................ggugagugggauuuuagucc...........................................................  10      0  S03
...............................ggugagugggauuuuaguccu..........................................................  12      0  S03
...............................ggugagugggauuuuaguccug.........................................................  218     0  S03
...............................ggugagugggauuuuaguccugu........................................................  1       0  S03
...............................ggugagugggauuuuaguccuguauc.....................................................  2       0  S03
...............................ggugagugggauuuuaguccuguaucu....................................................  1       0  S03
............................................uuaguccuguaucuaaaacacu............................................  1       0  S03
...................................................................ucaugacuagauccacacucaucca..................  1       0  S03
......................................................................ugacuagauccacacuca......................  1       0  S03
......................................................................ugacuagauccacacucau.....................  12      0  S03
......................................................................ugacuagauccacacucauc....................  77      0  S03
......................................................................ugacuagauccacacucaucc...................  1340    0  S03
......................................................................ugacuagauccacacucaucca..................  14678   0  S03
......................................................................ugacuagauccacacucauccac.................  76      0  S03
......................................................................ugacuagauccacacucauccacg................  1       0  S03
.......................................................................gacuagauccacacucauc....................  1       0  S03
.......................................................................gacuagauccacacucaucca..................  16      0  S03
.........................................................................cuagauccacacucaucca..................  1       0  S03
```

Star **Mature**

```
guugcugugauuguuuucucuuccaugugugggugagugggauuuuaguccuguaucuaaaacacuaucaugacuagauccacacucauccacgcugggagugacuuuca

guugcugugauuguuuucucuuccaugugug...............................................................................  2       0   S08
.uugcugugauuguuuucucuuccaugugug...............................................................................  1       0   S08
..ugcugugauuguuuucucuuccaugugug...............................................................................  1       0   S08
...gcugugauuguuuucucuuccaugugug...............................................................................  15      0   S08
....cugugauuguuuucucuuccaugugug...............................................................................  1       0   S08
..............................gggugagugggauuuuaguccug.........................................................  2       0   S08
...............................ggugagugggauuuuagucc...........................................................  16      0   S08
...............................ggugagugggauuuuaguccu..........................................................  43      0   S08
...............................ggugagugggauuuuaguccug.........................................................  303     0   S08
...............................ggugagugggauuuuaguccugua.......................................................  1       0   S08
...............................ggugagugggauuuuaguccuguauc.....................................................  1       0   S08
...............................ggugagugggauuuuaguccuguaucuaaaacacu............................................  6       0   S08
...............................ggugagugggauuuuaguccuguaucuaaaacacua...........................................  2       0   S08
...............................ggugagugggauuuuaguccuguaucuaaaacacuauc.........................................  3       0   S08
...............................ggugagugggauuuuaguccuguaucuaaaacacuauca........................................  8       0   S08
.................................ugagugggauuuuaguccu..........................................................  1       0   S08
.................................ugagugggauuuuaguccug.........................................................  1       0   S08
..................................gagugggauuuuaguccug.........................................................  1       0   S08
......................................gggauuuuaguccuguaucuaaaacacuauc.........................................  1       0   S08
......................................gggauuuuaguccuguaucuaaaacacuauca........................................  2       0   S08
............................................uuaguccuguaucuaaaacacu............................................  2       0   S08
.............................................uaguccuguaucuaaaacacu............................................  1       0   S08
.............................................uaguccuguaucuaaaacacua...........................................  2       0   S08
..............................................aguccuguaucuaaaacacua...........................................  1       0   S08
................................................uccuguaucuaaaacacuauca........................................  1       0   S08
.....................................................uaucuaaaacacuaucaugacuagauccacacucaucca..................  3       0   S08
........................................................cuaaaacacuaucaugacuagauccacacucaucca..................  1       0   S08
...........................................................aaacacuaucaugacuagauccacacucaucca..................  1       0   S08
..................................................................aucaugacuagauccacacucaucca..................  1       0   S08
...................................................................ucaugacuagauccacacucaucca..................  2       0   S08
....................................................................caugacuagauccacacucaucc...................  1       0   S08
......................................................................ugacuagauccacacuca......................  69      0   S08
......................................................................ugacuagauccacacucau.....................  54      0   S08
......................................................................ugacuagauccacacucauc....................  235     0   S08
......................................................................ugacuagauccacacucaucc...................  9466    0   S08
......................................................................ugacuagauccacacucaucca..................  153595  0   S08
......................................................................ugacuagauccacacucauccac.................  644     0   S08
......................................................................ugacuagauccacacucauccacg................  2       0   S08
.......................................................................gacuagauccacacucaucc...................  10      0   S08
.......................................................................gacuagauccacacucaucca..................  388     0   S08
.......................................................................gacuagauccacacucauccac.................  3       0   S08
.........................................................................cuagauccacacucaucca..................  6       0   S08
...........................................................................agauccacacucauccac.................  1       0   S08

.........auuguuuucucuuccaugugug...............................................................................  1       0   S02
..............................gggugagugggauuuuaguccug.........................................................  1       0   S02
...............................ggugagugggauuuuagucc...........................................................  4       0   S02
...............................ggugagugggauuuuaguccu..........................................................  3       0   S02
...............................ggugagugggauuuuaguccug.........................................................  80      0   S02
...............................ggugagugggauuuuaguccugu........................................................  1       0   S02
...............................ggugagugggauuuuaguccuguaucuaaaacacu............................................  1       0   S02
...............................ggugagugggauuuuaguccuguaucuaaaacacuauc.........................................  1       0   S02
............................................uuaguccuguaucuaaaacacu............................................  2       0   S02
......................................................................ugacuagauccacacuca......................  1       0   S02
......................................................................ugacuagauccacacucau.....................  7       0   S02
......................................................................ugacuagauccacacucauc....................  42      0   S02
......................................................................ugacuagauccacacucaucc...................  859     0   S02
......................................................................ugacuagauccacacucaucca..................  2697    0   S02
......................................................................ugacuagauccacacucauccac.................  8       0   S02
.......................................................................gacuagauccacacucaucca..................  7       0   S02

..............................gggugagugggauuuuagucc...........................................................  1       0   S04
..............................gggugagugggauuuuaguccug.........................................................  1       0   S04
...............................ggugagugggauuuuagucc...........................................................  17      0   S04
...............................ggugagugggauuuuaguccu..........................................................  30      0   S04
...............................ggugagugggauuuuaguccug.........................................................  461     0   S04
...............................ggugagugggauuuuaguccugu........................................................  1       0   S04
...............................ggugagugggauuuuaguccuguauc.....................................................  1       0   S04
...............................ggugagugggauuuuaguccuguaucuaaaacacu............................................  2       0   S04
```

```
                                Star                                    Mature
guugcugugauuguuuucucuuccaugugugggugagugggauuuuaguccuguaucuaaaacacuaucaugacuagauccacacucauccacgcugggagugacuuuca
.....................................................uaucuaaaacacuaucaugacuagauccacacucaucca..................    1       0    S04
......................................................aucuaaaacacuaucaugacuagauccacacucaucca..................    1       0    S04
.......................................................ucuaaaacacuaucaugacuag.................................    1       0    S04
.....................................................................augacuagauccacacucaucca..................    1       0    S04
......................................................................ugacuagauccacacuca......................    5       0    S04
......................................................................ugacuagauccacacucau.....................    16      0    S04
......................................................................ugacuagauccacacucauc....................    76      0    S04
......................................................................ugacuagauccacacucaucc...................    1471    0    S04
......................................................................ugacuagauccacacucaucca..................    17809   0    S04
......................................................................ugacuagauccacacucauccac.................    76      0    S04
.......................................................................gacuagauccacacucaucca..................    30      0    S04
```

Provisional ID	:	Scaffold248_6738
Score total	:	950805.9
Score for star read(s)	:	3.9
Score for read counts	:	950799.5
Score for mfe	:	0.9
Score for randfold	:	1.6
Score for cons. seed	:	
Total read count	:	1864964
Mature read count	:	1864676
Loop read count	:	0
Star read count	:	288

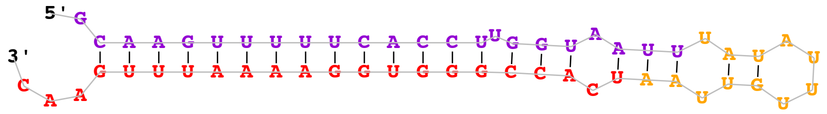

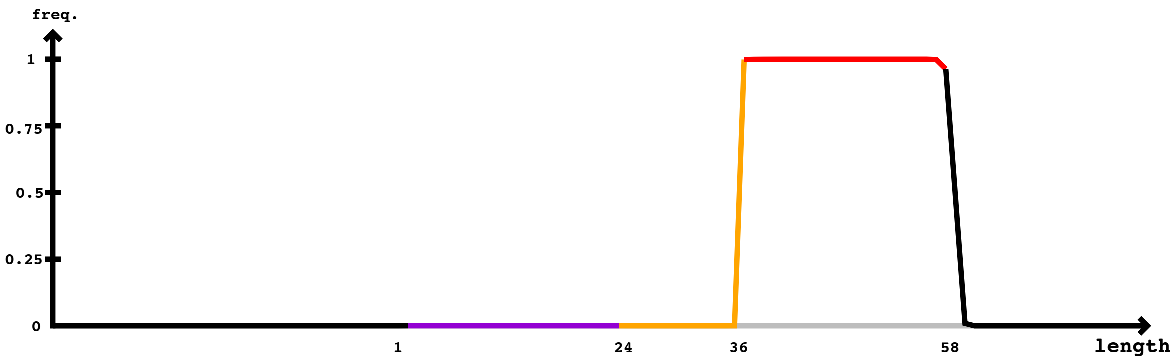

Star Mature

```
5'- aaauccaucaaguuugaagaaaucuuucuucuagugcaaguuuuucaccuugguaauuuauauuuguuaaucaccggguggaaaauuugaacuugaagacugaucuaucaua  -3'  obs
    aaauccaucaaguuugaagaaaucuuucuucuagugcaaguuuuucaccuugguaauuuauauuuguuaaucaccggguggaaaauuugaacuugaagacugaucuaucaua       exp
    ..............((((((..((..(((((.(((.((((((((((((((.(((.(((.((....)).))).))))))))))))))))).))).)))))..))))).)))..  reads  mm  sample
    ...................................gcaaguuuuucaccuuggua.........................................................  10  0  S10
    ...................................gcaaguuuuucaccuugguaa........................................................  1  0  S10
    ...................................gcaaguuuuucaccuugguaau.......................................................  3  0  S10
    ...................................gcaaguuuuucaccuugguaauu......................................................  23  0  S10
    ............................................................uauuuguuaaucaccggguggaaaauuugaac....................  1  0  S10
    .....................................................................aucaccggguggaaaauuugaac....................  1  0  S10
    ......................................................................ucaccggguggaaaauuug.......................  3  0  S10
    ......................................................................ucaccggguggaaaauuuga......................  443  0  S10
    ......................................................................ucaccggguggaaaauuugaa.....................  3729  0  S10
    ......................................................................ucaccggguggaaaauuugaac....................  56593  0  S10
    ......................................................................ucaccggguggaaaauuugaacu...................  11875  0  S10
    ......................................................................ucaccggguggaaaauuugaacuu..................  784  0  S10
    ......................................................................ucaccggguggaaaauuugaacuug.................  1  0  S10
    ........................................................................caccggguggaaaauuugaa.....................  3  0  S10
    ........................................................................caccggguggaaaauuugaac....................  31  0  S10
    ........................................................................caccggguggaaaauuugaacu...................  28  0  S10
    ........................................................................caccggguggaaaauuugaacuu..................  3  0  S10
    .........................................................................accggguggaaaauuugaa.....................  1  0  S10
    .........................................................................accggguggaaaauuugaac....................  17  0  S10
    .........................................................................accggguggaaaauuugaacu...................  5  0  S10
    ..........................................................................ccggguggaaaauuugaac....................  7  0  S10
    ..........................................................................ccggguggaaaauuugaacuug.................  2  0  S10
    ...........................................................................cggguggaaaauuugaac....................  1  0  S10
    ............................................................................ggguggaaaauuugaacu...................  2  0  S10
    ............................................................................ggguggaaaauuugaacuu..................  2  0  S10

    ...............................uagugcaaguuuuucaccuug............................................................  1  0  S05
    ...................................gcaaguuuuucaccuuggu..........................................................  2  0  S05
    ...................................gcaaguuuuucaccuuggua.........................................................  3  0  S05
    ...................................gcaaguuuuucaccuugguaau.......................................................  6  0  S05
    ...................................gcaaguuuuucaccuugguaauu......................................................  21  0  S05
    .........................................................uuauauuuguuaaucaccgggu.................................  1  0  S05
    ..........................................................uauauuuguuaaucaccggguggaaaauuugaac....................  12  0  S05
```

```
                                   Star                                   Mature
aaauccaucaaguuugaagaaaucuuucuucuagugcaaguuuuucaccuugguaauuuauauuuguuaaucaccggguggaaaauuugaacuugaagacugaucuaucaua
...........................................................auauuuguuaaucaccggguggaaaauuugaac....................   1       0   S05
....................................................................aaucaccggguggaaaauuugaac....................   4       0   S05
.....................................................................aucaccggguggaaaauuugaa.....................   1       0   S05
.....................................................................aucaccggguggaaaauuugaac....................   7       0   S05
.....................................................................aucaccggguggaaaauuugaacu...................   1       0   S05
......................................................................ucaccggguggaaaauuug.......................   2       0   S05
......................................................................ucaccggguggaaaauuuga......................   108     0   S05
......................................................................ucaccggguggaaaauuugaa.....................   6679    0   S05
......................................................................ucaccggguggaaaauuugaac....................   185975  0   S05
......................................................................ucaccggguggaaaauuugaacu...................   20212   0   S05
......................................................................ucaccggguggaaaauuugaacuu..................   2108    0   S05
......................................................................ucaccggguggaaaauuugaacuug.................   1       0   S05
.......................................................................caccggguggaaaauuugaa.....................   3       0   S05
.......................................................................caccggguggaaaauuugaac....................   98      0   S05
.......................................................................caccggguggaaaauuugaacu...................   54      0   S05
.......................................................................caccggguggaaaauuugaacuu..................   26      0   S05
........................................................................accggguggaaaauuugaa.....................   1       0   S05
........................................................................accggguggaaaauuugaac....................   43      0   S05
........................................................................accggguggaaaauuugaacu...................   5       0   S05
........................................................................accggguggaaaauuugaacuu..................   3       0   S05
........................................................................accggguggaaaauuugaacuug.................   1       0   S05
.........................................................................ccggguggaaaauuugaa.....................   2       0   S05
.........................................................................ccggguggaaaauuugaac....................   27      0   S05
.........................................................................ccggguggaaaauuugaacu...................   1       0   S05
.........................................................................ccggguggaaaauuugaacuu..................   1       0   S05
.........................................................................ccggguggaaaauuugaacuug.................   7       0   S05
..........................................................................cggguggaaaauuugaac....................   18      0   S05
..........................................................................cggguggaaaauuugaacu...................   1       0   S05
...........................................................................ggguggaaaauuugaacu...................   5       0   S05
............................................................................gguggaaaauuugaacuu..................   1       0   S05

...............................uagugcaaguuuuucaccuuggu..........................................................   1       0   S07
................................agugcaaguuuuucaccuuggua.........................................................   1       0   S07
...................................gcaaguuuuucaccuugg...........................................................   2       0   S07
...................................gcaaguuuuucaccuuggu..........................................................   3       0   S07
...................................gcaaguuuuucaccuuggua.........................................................   24      0   S07
...................................gcaaguuuuucaccuugguaa........................................................   4       0   S07
...................................gcaaguuuuucaccuugguaau.......................................................   8       0   S07
...................................gcaaguuuuucaccuugguaauu......................................................   27      0   S07
..........................................................uauauuuguuaaucaccggguggaaaauuugaac....................   4       0   S07
...........................................................auauuuguuaaucaccggguggaaaauuugaac....................   1       0   S07
....................................................................aaucaccggguggaaaauuugaac....................   1       0   S07
.....................................................................aucaccggguggaaaauuugaac....................   2       0   S07
.....................................................................aucaccggguggaaaauuugaacu...................   1       0   S07
......................................................................ucaccggguggaaaauuu........................   2       0   S07
......................................................................ucaccggguggaaaauuug.......................   9       0   S07
......................................................................ucaccggguggaaaauuuga......................   505     0   S07
......................................................................ucaccggguggaaaauuugaa.....................   13625   0   S07
......................................................................ucaccggguggaaaauuugaac....................   348954  0   S07
......................................................................ucaccggguggaaaauuugaacu...................   52145   0   S07
......................................................................ucaccggguggaaaauuugaacuu..................   4015    0   S07
.......................................................................caccggguggaaaauuugaa.....................   4       0   S07
.......................................................................caccggguggaaaauuugaac....................   190     0   S07
.......................................................................caccggguggaaaauuugaacu...................   131     0   S07
.......................................................................caccggguggaaaauuugaacuu..................   22      0   S07
........................................................................accggguggaaaauuugaa.....................   1       0   S07
........................................................................accggguggaaaauuugaac....................   83      0   S07
........................................................................accggguggaaaauuugaacu...................   14      0   S07
........................................................................accggguggaaaauuugaacuug.................   2       0   S07
.........................................................................ccggguggaaaauuugaa.....................   2       0   S07
.........................................................................ccggguggaaaauuugaac....................   43      0   S07
.........................................................................ccggguggaaaauuugaacu...................   9       0   S07
.........................................................................ccggguggaaaauuugaacuu..................   1       0   S07
.........................................................................ccggguggaaaauuugaacuug.................   9       0   S07
..........................................................................cggguggaaaauuugaac....................   33      0   S07
..........................................................................cggguggaaaauuugaacuug.................   1       0   S07
...........................................................................ggguggaaaauuugaacu...................   7       0   S07

...................................gcaaguuuuucaccuuggua.........................................................   1       0   S01
......................................................................ucaccggguggaaaauuuga......................   8       0   S01
```

	Star	Mature			

aaauccaucaaguuugaagaaaucuuucuucuagugcaaguuuuucaccuugguaauuuauauuuguuaaucaccggguggaaaauuugaacuugaagacugaucuaucaua			
....ucaccggguggaaaauuugaa.....................	619	0	S01
....ucaccggguggaaaauuugaac....................	15234	0	S01
....ucaccggguggaaaauuugaacu...................	158	0	S01
....ucaccggguggaaaauuugaacuu..................	2	0	S01
....caccggguggaaaauuugaa.....................	1	0	S01
....caccggguggaaaauuugaac....................	10	0	S01
....caccggguggaaaauuugaacu...................	1	0	S01
....accggguggaaaauuugaa.....................	1	0	S01
....accggguggaaaauuugaac....................	7	0	S01
....ccggguggaaaauuugaac....................	2	0	S01
....ccggguggaaaauuugaacuug.................	3	0	S01
....cggguggaaaauuugaac....................	2	0	S01
...............................uagugcaaguuuuucaccuuggu..	1	0	S06
................................agugcaaguuuuucaccuugguaa..	1	0	S06
...................................gcaaguuuuucaccuuggu..	1	0	S06
...................................gcaaguuuuucaccuuggua...	4	0	S06
...................................gcaaguuuuucaccuugguaau...	1	0	S06
...................................gcaaguuuuucaccuugguaauu..	7	0	S06
....uauauuuguuaaucaccggguggaaaauuugaac....................	9	0	S06
....auauuuguuaaucaccggguggaaaauuugaac....................	1	0	S06
....aaucaccggguggaaaauuugaac....................	6	0	S06
....aaucaccggguggaaaauuugaacu...................	1	0	S06
....aucaccggguggaaaauuugaac....................	13	0	S06
....ucaccggguggaaaauuug.......................	6	0	S06
....ucaccggguggaaaauuuga......................	256	0	S06
....ucaccggguggaaaauuugaa.....................	10589	0	S06
....ucaccggguggaaaauuugaac....................	239917	0	S06
....ucaccggguggaaaauuugaacu...................	25488	0	S06
....ucaccggguggaaaauuugaacuu..................	1786	0	S06
....ucaccggguggaaaauuugaacuug.................	1	0	S06
....caccggguggaaaauuugaa.....................	3	0	S06
....caccggguggaaaauuugaac....................	138	0	S06
....caccggguggaaaauuugaacu...................	81	0	S06
....caccggguggaaaauuugaacuu..................	20	0	S06
....caccggguggaaaauuugaacuug.................	1	0	S06
....accggguggaaaauuugaa.....................	1	0	S06
....accggguggaaaauuugaac....................	55	0	S06
....accggguggaaaauuugaacu...................	8	0	S06
....ccggguggaaaauuugaa.....................	4	0	S06
....ccggguggaaaauuugaac....................	54	0	S06
....ccggguggaaaauuugaacu...................	3	0	S06
....ccggguggaaaauuugaacuug.................	1	0	S06
....cggguggaaaauuugaac....................	17	0	S06
....ggguggaaaauuugaacu...................	4	0	S06
....gguggaaaauuugaacuu..................	1	0	S06
...................................gcaaguuuuucaccuuggua...	23	0	S08
...................................gcaaguuuuucaccuugguaa..	8	0	S08
...................................gcaaguuuuucaccuugguaau...	6	0	S08
...................................gcaaguuuuucaccuugguaauu..	22	0	S08
....uauauuuguuaaucaccggguggaaaauuugaacu...................	1	0	S08
....uaaucaccggguggaaaauuugaa.....................	1	0	S08
....aaucaccggguggaaaauuugaa.....................	1	0	S08
....aaucaccggguggaaaauuugaac....................	1	0	S08
....ucaccggguggaaaauuug.......................	5	0	S08
....ucaccggguggaaaauuuga......................	580	0	S08
....ucaccggguggaaaauuugaa.....................	12241	0	S08
....ucaccggguggaaaauuugaac....................	223868	0	S08
....ucaccggguggaaaauuugaacu...................	39219	0	S08
....ucaccggguggaaaauuugaacuu..................	2990	0	S08
....ucaccggguggaaaauuugaacuug.................	1	0	S08
....caccggguggaaaauuuga......................	1	0	S08
....caccggguggaaaauuugaa.....................	3	0	S08
....caccggguggaaaauuugaac....................	124	0	S08
....caccggguggaaaauuugaacu...................	74	0	S08
....caccggguggaaaauuugaacuu..................	26	0	S08
....accggguggaaaauuugaa.....................	3	0	S08
....accggguggaaaauuugaac....................	60	0	S08
....accggguggaaaauuugaacu...................	7	0	S08

Star Mature

```
aaauccaucaaguuugaagaaaucuuucuucuagugcaaguuuuucaccuugguaauuuauauuuguuaaucaccggguggaaaauuugaacuugaagacugaucuaucaua
........................................................................accggguggaaaauuugaacuu..................  4       0   S08
........................................................................accggguggaaaauuugaacuug.................  1       0   S08
.........................................................................ccggguggaaaauuugaa.....................  4       0   S08
.........................................................................ccggguggaaaauuugaac....................  39      0   S08
.........................................................................ccggguggaaaauuugaacu...................  8       0   S08
.........................................................................ccggguggaaaauuugaacuu..................  1       0   S08
.........................................................................ccggguggaaaauuugaacuug.................  9       0   S08
..........................................................................cggguggaaaauuugaac....................  38      0   S08
..........................................................................cggguggaaaauuugaacu...................  2       0   S08
...........................................................................ggguggaaaauuugaacu...................  6       0   S08

................aagaaaucuuucuucuagu.............................................................................  2       0   S03
...................................gcaaguuuuucaccuuggua.........................................................  3       0   S03
...................................gcaaguuuuucaccuugguaau.......................................................  1       0   S03
...................................gcaaguuuuucaccuugguaauu......................................................  16      0   S03
........................................................uuuauauuuguuaaucaccgggu.................................  1       0   S03
..........................................................uauauuuguuaaucaccggguggaaaauuugaac....................  19      0   S03
..........................................................uauauuuguuaaucaccggguggaaaauuugaacuu..................  1       0   S03
................................................................uguuaaucaccggguggaaaauuugaac....................  1       0   S03
.................................................................guuaaucaccggguggaaaauuugaac....................  2       0   S03
....................................................................aaucaccggguggaaaauuugaac....................  2       0   S03
....................................................................aaucaccggguggaaaauuugaacuu..................  1       0   S03
.....................................................................aucaccggguggaaaauuugaac....................  1       0   S03
......................................................................ucaccggguggaaaauuug.......................  4       0   S03
......................................................................ucaccggguggaaaauuuga......................  94      0   S03
......................................................................ucaccggguggaaaauuugaa.....................  6832    0   S03
......................................................................ucaccggguggaaaauuugaac....................  200477  0   S03
......................................................................ucaccggguggaaaauuugaacu...................  18224   0   S03
......................................................................ucaccggguggaaaauuugaacuu..................  1823    0   S03
......................................................................ucaccggguggaaaauuugaacuug.................  1       0   S03
.......................................................................caccggguggaaaauuuga......................  1       0   S03
.......................................................................caccggguggaaaauuugaa.....................  10      0   S03
.......................................................................caccggguggaaaauuugaac....................  116     0   S03
.......................................................................caccggguggaaaauuugaacu...................  41      0   S03
.......................................................................caccggguggaaaauuugaacuu..................  16      0   S03
........................................................................accggguggaaaauuugaa.....................  2       0   S03
........................................................................accggguggaaaauuugaac....................  45      0   S03
........................................................................accggguggaaaauuugaacu...................  5       0   S03
........................................................................accggguggaaaauuugaacuu..................  3       0   S03
........................................................................accggguggaaaauuugaacuug.................  4       0   S03
.........................................................................ccggguggaaaauuugaa.....................  3       0   S03
.........................................................................ccggguggaaaauuugaac....................  42      0   S03
.........................................................................ccggguggaaaauuugaacu...................  2       0   S03
.........................................................................ccggguggaaaauuugaacuu..................  1       0   S03
.........................................................................ccggguggaaaauuugaacuug.................  6       0   S03
..........................................................................cggguggaaaauuugaac....................  17      0   S03
..........................................................................cggguggaaaauuugaacu...................  1       0   S03
...........................................................................ggguggaaaauuugaacu...................  3       0   S03

...................................gcaaguuuuucaccuuggua.........................................................  6       0   S09
...................................gcaaguuuuucaccuugguaa........................................................  2       0   S09
...................................gcaaguuuuucaccuugguaau.......................................................  5       0   S09
...................................gcaaguuuuucaccuugguaauu......................................................  17      0   S09
..........................................................uauauuuguuaaucaccgggug................................  1       0   S09
......................................................................ucaccggguggaaaauuu........................  2       0   S09
......................................................................ucaccggguggaaaauuug.......................  4       0   S09
......................................................................ucaccggguggaaaauuuga......................  318     0   S09
......................................................................ucaccggguggaaaauuugaa.....................  2678    0   S09
......................................................................ucaccggguggaaaauuugaac....................  43043   0   S09
......................................................................ucaccggguggaaaauuugaacu...................  10383   0   S09
......................................................................ucaccggguggaaaauuugaacuu..................  679     0   S09
.......................................................................caccggguggaaaauuugaa.....................  2       0   S09
.......................................................................caccggguggaaaauuugaac....................  28      0   S09
.......................................................................caccggguggaaaauuugaacu...................  16      0   S09
.......................................................................caccggguggaaaauuugaacuu..................  4       0   S09
........................................................................accggguggaaaauuugaac....................  11      0   S09
........................................................................accggguggaaaauuugaacu...................  2       0   S09
.........................................................................ccggguggaaaauuugaac....................  2       0   S09
.........................................................................ccggguggaaaauuugaacuug.................  3       0   S09
..........................................................................cggguggaaaauuugaac....................  1       0   S09
```

Star Mature

```
aaauccaucaaguuugaagaaaucuuucuucuagugcaaguuuuucaccuugguaauuuauauuuguuaaucaccggguggaaaauuugaacuugaagacugaucuaucaua
...........................................................................ggguggaaaauuugaacu...................	1	0	S09
...........................................................................ggguggaaaauuuUaacu...................	1	1	S09

...............................uagugcaaguuuuucaccuuggu..........................................................	1	0	S04
...................................gcaaguuuuucaccuuggua.........................................................	4	0	S04
...................................gcaaguuuuucaccuugguaa........................................................	1	0	S04
...................................gcaaguuuuucaccuugguaau.......................................................	5	0	S04
...................................gcaaguuuuucaccuugguaauu......................................................	18	0	S04
..........................................................uauauuuguuaaucaccggguggaaaauuugaac....................	12	0	S04
..........................................................uauauuuguuaaucaccggguggaaaauuugaacu...................	1	0	S04
............................................................uauuuguuaaucaccgggug................................	1	0	S04
....................................................................aaucaccggguggaaaauuugaac....................	3	0	S04
......................................................................ucaccggguggaaaauuu........................	1	0	S04
......................................................................ucaccggguggaaaauuug.......................	2	0	S04
......................................................................ucaccggguggaaaauuuga......................	154	0	S04
......................................................................ucaccggguggaaaauuugaa.....................	8248	0	S04
......................................................................ucaccggguggaaaauuugaac....................	245259	0	S04
......................................................................ucaccggguggaaaauuugaacu...................	24831	0	S04
......................................................................ucaccggguggaaaauuugaacuu..................	2732	0	S04
.......................................................................caccggguggaaaauuugaa.....................	5	0	S04
.......................................................................caccggguggaaaauuugaac....................	132	0	S04
.......................................................................caccggguggaaaauuugaacu...................	61	0	S04
.......................................................................caccggguggaaaauuugaacuu..................	32	0	S04
........................................................................accggguggaaaauuugaa.....................	1	0	S04
........................................................................accggguggaaaauuugaac....................	72	0	S04
........................................................................accggguggaaaauuugaacu...................	6	0	S04
........................................................................accggguggaaaauuugaacuu..................	1	0	S04
........................................................................accggguggaaaauuugaacuug.................	5	0	S04
.........................................................................ccggguggaaaauuugaa.....................	1	0	S04
.........................................................................ccggguggaaaauuugaac....................	37	0	S04
.........................................................................ccggguggaaaauuugaacu...................	2	0	S04
.........................................................................ccggguggaaaauuugaacuu..................	1	0	S04
.........................................................................ccggguggaaaauuugaacuug.................	6	0	S04
..........................................................................cggguggaaaauuugaac....................	22	0	S04
..........................................................................cggguggaaaauuugaacu...................	2	0	S04
...........................................................................ggguggaaaauuugaacu...................	4	0	S04

......................................................................ucaccggguggaaaauuu........................	1	0	S02
......................................................................ucaccggguggaaaauuug.......................	2	0	S02
......................................................................ucaccggguggaaaauuuga......................	8	0	S02
......................................................................ucaccggguggaaaauuugaa.....................	572	0	S02
......................................................................ucaccggguggaaaauuugaac....................	14303	0	S02
......................................................................ucaccggguggaaaauuugaacu...................	571	0	S02
......................................................................ucaccggguggaaaauuugaacuu..................	71	0	S02
.......................................................................caccggguggaaaauuugaac....................	8	0	S02
.......................................................................caccggguggaaaauuugaacu...................	1	0	S02
........................................................................accggguggaaaauuugaac....................	7	0	S02
.........................................................................ccggguggaaaauuugaac....................	1	0	S02
..........................................................................cggguggaaaauuugaac....................	1	0	S02
```

Provisional ID	:	Scaffold1827_30268
Score total	:	86.8
Score for star read(s)	:	3.9
Score for read counts	:	79.7
Score for mfe	:	1.6
Score for randfold	:	1.6
Score for cons. seed	:	
Total read count	:	168
Mature read count	:	132
Loop read count	:	0
Star read count	:	36

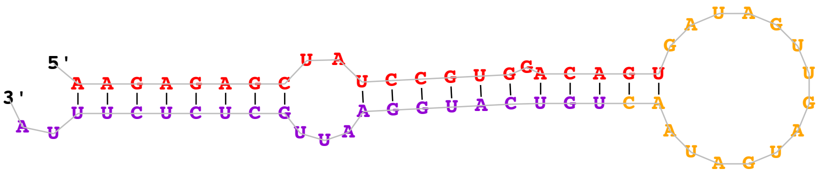

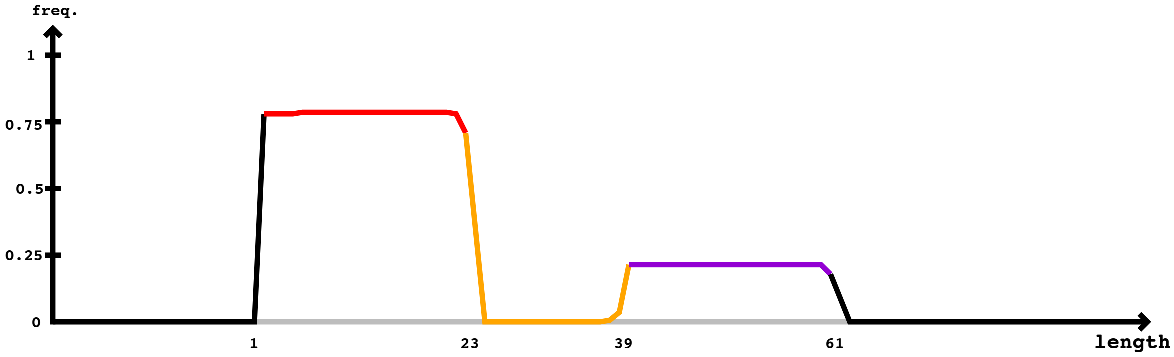

Mature **Star**

```
5'- aguuaagagaguuuugacaacgaagagagcuauccguggacagugauaguugaugauaacugucauggaauugcucucuuuauaguucugacucacaucuucagccacaugggg  -3'  obs
    aguuaagagaguuuugacaacgaagagagcuauccguggacagugauaguugaugauaacugucauggaauugcucucuuuauaguucugacucacaucuucagccacaugggg        exp
    ......(((((..(((.(..((((((((..((((((.(((((................)))))))))))...))))))))..).)))..)))))....(((((......)))))   reads   mm   sample
    ......................aagagagcuauccguggacag.......................................................................   1       0    S03
    ......................aagagagcuauccguggacagu......................................................................   6       0    S03
    ......................aagagagcuauccguggacagug.....................................................................   1       0    S03
    ...........................................................cugucauggaauugcucucuuua................................   1       0    S03

    ......................aagagagcuauccguggacagu......................................................................   4       0    S09
    ......................aagagagcuauccguggacagug.....................................................................   2       0    S09
    ..........................................................acugucauggaauugcucucuuua................................   1       0    S09
    ...........................................................cugucauggaauugcucucuuua................................   1       0    S09
    ............................................................ugucauggaauugcucucuuu.................................   1       0    S09
    ............................................................ugucauggaauugcucucuuua................................   7       0    S09

    ......................aagagagcuauccguggacag.......................................................................   2       0    S08
    ......................aagagagcuauccguggacagu......................................................................   5       0    S08
    ............................................................ugucauggaauugcucucuuu.................................   3       0    S08
    ............................................................ugucauggaauugcucucuuua................................   3       0    S08

    ......................aagagagcuauccguggacag.......................................................................   1       0    S02
    ......................aagagagcuauccguggacagu......................................................................   13      0    S02
    ............................................................ugucauggaauugcucucuuua................................   2       0    S02

    ......................aagagagcuauccguggacagu......................................................................   7       0    S04

    ......................aagagagcuauccguggacag.......................................................................   1       0    S05
    ......................aagagagcuauccguggacagu......................................................................   15      0    S05
    ............................................................ugucauggaauugcucucuuua................................   1       0    S05

    ......................aagagagcuauccguggacag.......................................................................   1       0    S10
    ......................aagagagcuauccguggacagu......................................................................   9       0    S10
    ...........................................................cugucauggaauugcucucuuua................................   1       0    S10
    ............................................................ugucauggaauugcucucuuu.................................   1       0    S10
    ............................................................ugucauggaauugcucucuuua................................   7       0    S10

    ......................aagagagcuauccguggaca........................................................................   1       0    S06
```

```
                    Mature                                  Star
aguuaagaguuuugacaacgaagagagcuauccguggacagugauaguugaugauaaacugucauggaauugcucucuuuauaguucugacucacaucuucagccacaugggg
....................aagagagcuauccguggacag........................................................................   5    0    S06
....................aagagagcuauccguggacagu.......................................................................   43   0    S06
....................aagagagcuauccguggacagug......................................................................   5    0    S06
........................gagcuauccguggacagu.......................................................................   1    0    S06
...........................................................ugucauggaauugcucucuuu.................................   1    0    S06
...........................................................ugucauggaauugcucucuuua................................   3    0    S06

....................aagagagcuauccguggacagu.......................................................................   4    0    S01

....................aagagagcuauccguggacag........................................................................   1    0    S07
....................aagagagcuauccguggacagu.......................................................................   4    0    S07
..........................................................cugucauggaauugcucucuuua................................   2    0    S07
...........................................................ugucauggaauugcucucuuua................................   1    0    S07
```

Provisional ID	:	Scaffold166_4787
Score total	:	56.2
Score for star read(s)	:	3.9
Score for read counts	:	50.1
Score for mfe	:	0.6
Score for randfold	:	1.6
Score for cons. seed	:	
Total read count	:	110
Mature read count	:	92
Loop read count	:	0
Star read count	:	18

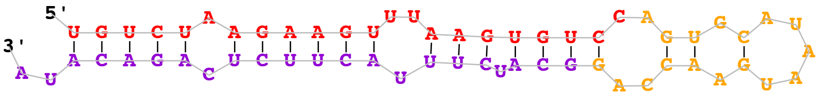

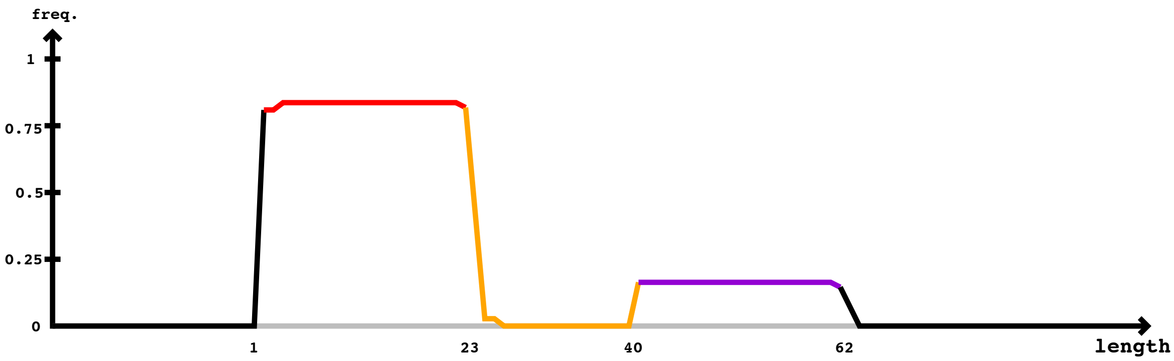

Mature **Star**

```
5'- ugaugaauugaaagacauuuugucuaagaaguuuaagugucc agugcauaaaugaaccaggcaucuuuacuucucagacauauuguucuuucaaucagacuuuucauauugau   -3'   obs
    ugaugaauugaaagacauuuugucuaagaaguuuaaguguccagugcauaaaugaaccaggcaucuuuacuucucagacauauuguucuuucaaucagacuuuucauauugau          exp
    ...(((.((((((((((...(((((.((((((..(((((((..((.(.....).))..)))).))).)))))).)))))...)).))))))))))).................  reads   mm   sample
    ....................ugucuaagaaguuuaagugucc.......................................................................  2       0    S08

    ....................ugucuaagaaguuuaagugucc.......................................................................  5       0    S03
    ............................................................Acaucuuuacuucucagacaua...............................  2       1    S03

    ....................ugucuaagaaguuuaagugucc.......................................................................  22      0    S09
    ......................ucuaagaaguuuaaguguccagu....................................................................  2       0    S09
    ............................................................Acaucuuuacuucucagacaua...............................  3       1    S09

    ....................ugucuaagaaguuuaagugucc.......................................................................  5       0    S02
    ....................ugucuaagaaguuuaaguguccca.....................................................................  1       0    S02
    ............................................................Acaucuuuacuucucagacaua...............................  1       1    S02

    ....................ugucuaagaaguuuaagugucc.......................................................................  4       0    S04

    ....................ugucuaagaaguuuaaguguc........................................................................  2       0    S10
    ....................ugucuaagaaguuuaagugucc.......................................................................  27      0    S10
    ............................................................Acaucuuuacuucucagacau................................  1       1    S10
    ............................................................Acaucuuuacuucucagacaua...............................  1       1    S10

    ....................ugucuaagaaguuuaagugucc.......................................................................  2       0    S05
    ............................................................Acaucuuuacuucucagacaua...............................  2       1    S05

    ....................ugucuaagaaguuuaagugucc.......................................................................  13      0    S07
    ......................ucuaagaaguuuaaguguccagu....................................................................  1       0    S07
    ............................................................Acaucuuuacuucucagacau................................  1       1    S07
    ............................................................Acaucuuuacuucucagacaua...............................  3       1    S07

    ....................ugucuaagaaguuuaagugucc.......................................................................  5       0    S06
    ............................................................Acaucuuuacuucucagacaua...............................  3       1    S06

    ....................ugucuaagaaguuuaagugucc.......................................................................  1       0    S01
    ............................................................Acaucuuuacuucucagacaua...............................  1       1    S01
```

Mature **Star**

ugaugaauugaaagacauuuugucuaagaaguuuaaguguccagugcauaaugaaccaggcaucuuuacuucucagacauauuguucuuucaaucagacuuuucauauugau

Provisional ID	:	Scaffold290_7701
Score total	:	2.2
Score for star read(s)	:	-1.3
Score for read counts	:	0
Score for mfe	:	1.9
Score for randfold	:	1.6
Score for cons. seed	:	
Total read count	:	1863
Mature read count	:	1838
Loop read count	:	0
Star read count	:	25

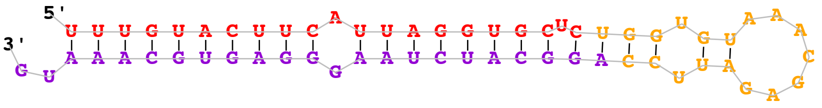

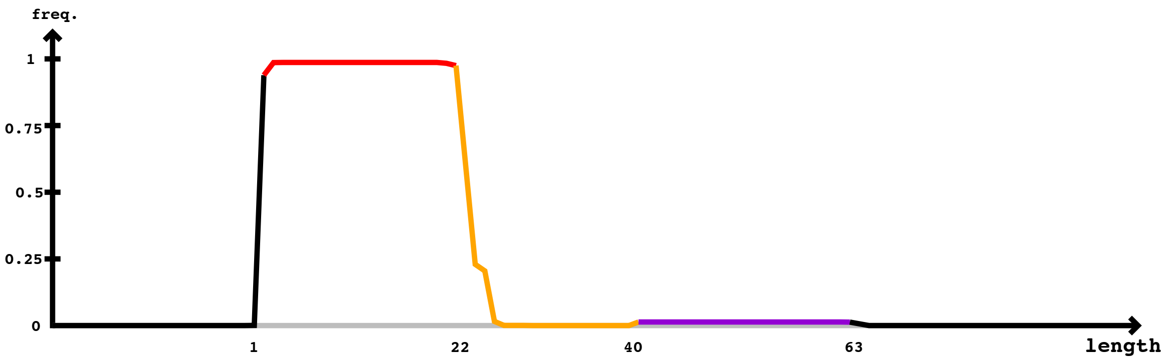


```
                         Mature                                    Star
5'- agagagaaugucaccaguuuuuuguacuucauuaggugcucugguguaaacgagauuccaggcaucuaagggagugcaaaugauagguggcugacauccaucaaagaagug   -3'   obs
    agagagaaugucaccaguuuuuuguacuucauuaggugcucugguguaaacgagauuccaggcaucuaagggagugcaaaugauagguggcugacauccaucaaagaagug         exp
    .((..((..((((((.(((.(((((((((.((((((((.((((.((.......)).)))))))))))).))))))))).))).))))))......))..)).........   reads   mm   sample
    ....................uuuguacuucauuaggugcuc......................................................................   37      0    S04
    ....................uuuguacuucauuaggugcucu.....................................................................   10      0    S04
    ....................uuuguacuucauuaggugcucugg...................................................................   64      0    S04
    ....................uuuguacuucauuaggugcucuggu..................................................................   2       0    S04
    .....................uuguacuucauuaggugcuc......................................................................   1       0    S04
    .....................uuguacuucauuaggugcucu.....................................................................   1       0    S04
    .....................uuguacuucauuaggugcucugg...................................................................   9       0    S04
    ...........................................................aggcaucuaagggagugcaaaug.............................   4       0    S04

    ....................uuuguacuucauuaggugc........................................................................   2       0    S02
    ....................uuuguacuucauuaggugcuc......................................................................   43      0    S02
    ....................uuuguacuucauuaggugcucu.....................................................................   10      0    S02
    ....................uuuguacuucauuaggugcucugg...................................................................   8       0    S02
    .....................uuguacuucauuaggugcuc......................................................................   1       0    S02

    ....................uuuguacuucauuaggugcuc......................................................................   81      0    S08
    ....................uuuguacuucauuaggugcucu.....................................................................   21      0    S08
    ....................uuuguacuucauuaggugcucug....................................................................   1       0    S08
    ....................uuuguacuucauuaggugcucugg...................................................................   7       0    S08
    .....................uuguacuucauuaggugcuc......................................................................   2       0    S08
    .....................uuguacuucauuaggugcucu.....................................................................   4       0    S08
    .....................uuguacuucauuaggugcucug....................................................................   2       0    S08

    ....................uuuguacuucauuaggugcu.......................................................................   6       0    S09
    ....................uuuguacuucauuaggugcuc......................................................................   321     0    S09
    ....................uuuguacuucauuaggugcucu.....................................................................   96      0    S09
    ....................uuuguacuucauuaggugcucug....................................................................   7       0    S09
    ....................uuuguacuucauuaggugcucugg...................................................................   61      0    S09
    ....................uuuguacuucauuaggugcucuggu..................................................................   3       0    S09
    .....................uuguacuucauuaggugcuc......................................................................   2       0    S09
    .....................uuguacuucauuaggugcucu.....................................................................   8       0    S09
    .....................uuguacuucauuaggugcucug....................................................................   2       0    S09
    .....................uuguacuucauuaggugcucugg...................................................................   6       0    S09
```

Mature **Star**

agagagaaugucaccaguuuuuuguacuucauuaggugcucugguguaaacgagauuccaggcaucuaagggagugcaaaugauagguggcugacauccaucaaagaagug			
......................uguacuucauuaggugcucuggu..	1	0	S09
....aggcaucuaagggagugcaaaug.............................	5	0	S09
....................uuuguacuucauuaggugcu...	2	0	S03
....................uuuguacuucauuaggugcuc..	33	0	S03
....................uuuguacuucauuaggugcucu...	6	0	S03
....................uuuguacuucauuaggugcucug..	2	0	S03
....................uuuguacuucauuaggugcucugg...	22	0	S03
....................uuuguacuucauuaggugcucuggu..	1	0	S03
....................uuuguacuucauuaggugcucuggugua...	1	0	S03
.....................uuguacuucauuaggugc..	1	0	S03
.....................uuguacuucauuaggugcuc..	2	0	S03
.....................uuguacuucauuaggugcucugg...	3	0	S03
....aggcaucuaagggagugcaaaug.............................	2	0	S03
...................uuuuguacuucauuaggugcucuggu..	1	0	S07
....................uuuguacuucauuaggugcu...	1	0	S07
....................uuuguacuucauuaggugcuc..	75	0	S07
....................uuuguacuucauuaggugcucu...	25	0	S07
....................uuuguacuucauuaggugcucug..	10	0	S07
....................uuuguacuucauuaggugcucugg...	35	0	S07
....................uuuguacuucauuaggugcucuggu..	8	0	S07
.....................uuguacuucauuaggugcucu...	5	0	S07
.....................uuguacuucauuaggugcucugg...	8	0	S07
.....................uuguacuucauuaggugcucuggu..	2	0	S07
....aggcaucuaagggagugcaaaug.............................	1	0	S07
....................uuuguacuucauuaggugcu...	1	0	S06
....................uuuguacuucauuaggugcuc..	83	0	S06
....................uuuguacuucauuaggugcucu...	24	0	S06
....................uuuguacuucauuaggugcucug..	3	0	S06
....................uuuguacuucauuaggugcucugg...	13	0	S06
....................uuuguacuucauuaggugcucuggu..	4	0	S06
.....................uuguacuucauuaggugcuc..	1	0	S06
.....................uuguacuucauuaggugcucu...	1	0	S06
.....................uuguacuucauuaggugcucugg...	1	0	S06
....aggcaucuaagggagugcaaau..............................	1	0	S06
....................uuuguacuucauuaggugcu...	1	0	S01
....................uuuguacuucauuaggugcuc..	54	0	S01
....................uuuguacuucauuaggugcucu...	8	0	S01
....................uuuguacuucauuaggugcucugg...	1	0	S01
.....................uuguacuucauuaggugc..	1	0	S01
.....................uuguacuucauuaggugcucu...	1	0	S01
....................uuuguacuucauuaggugcu...	4	0	S10
....................uuuguacuucauuaggugcuc..	234	0	S10
....................uuuguacuucauuaggugcucu...	116	0	S10
....................uuuguacuucauuaggugcucug..	13	0	S10
....................uuuguacuucauuaggugcucugg...	77	0	S10
....................uuuguacuucauuaggugcucuggu..	4	0	S10
.....................uuguacuucauuaggugcuc..	4	0	S10
.....................uuguacuucauuaggugcucu...	7	0	S10
.....................uuguacuucauuaggugcucug..	2	0	S10
.....................uuguacuucauuaggugcucugg...	9	0	S10
.....................uuguacuucauuaggugcucuggu..	1	0	S10
....aggcaucuaagggagugcaaaug.............................	10	0	S10
....................uuuguacuucauuaggugc..	2	0	S05
....................uuuguacuucauuaggugcu...	1	0	S05
....................uuuguacuucauuaggugcuc..	57	0	S05
....................uuuguacuucauuaggugcucu...	14	0	S05
....................uuuguacuucauuaggugcucug..	4	0	S05
....................uuuguacuucauuaggugcucugg...	29	0	S05
....................uuuguacuucauuaggugcucuggu..	1	0	S05
.....................uuguacuucauuaggugcucu...	1	0	S05
....aggcaucuaagggagugcaaaug.............................	2	0	S05

Provisional ID : Scaffold38_1202
Score total : 5076.9
Score for star read(s) : 3.9
Score for read counts : 5069.8
Score for mfe : 1.6
Score for randfold : 1.6
Score for cons. seed :
Total read count : 9956
Mature read count : 9335
Loop read count : 0
Star read count : 621

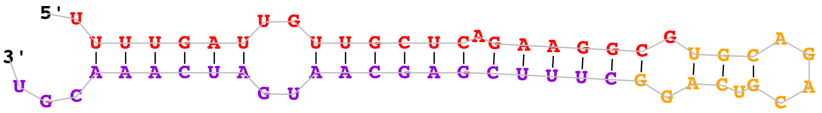

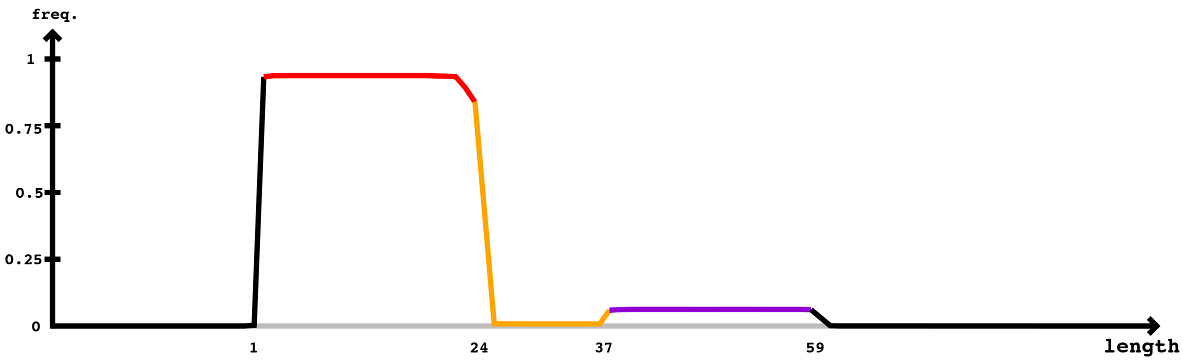

Mature Star

```
5'- guauuuaagucuucucgccuuuugauuguugcucagaaggcgugcagacgucaggcuuucgagcaaugaucaaacguugaggagggccaaauucaacacuuucguuacacac -3'   obs
    guauuuaagucuucucgccuuuugauuguugcucagaaggcgugcagacgucaggcuuucgagcaaugaucaaacguugaggagggccaaauucaacacuuucguuacacac        exp
    ..........((((((((.(.((((((..((((((.((((((.(((....).)).))))))))))))..)))))).).))))))))..........................   reads   mm   sample
    ..................cuuuugauuguugcucagaaggc.......................................................................   1       0    S07
    ...................uuuugauuguugcucagaag.........................................................................   2       0    S07
    ...................uuuugauuguugcucagaagg........................................................................   42      0    S07
    ...................uuuugauuguugcucagaaggc.......................................................................   47      0    S07
    ...................uuuugauuguugcucagaaggcg......................................................................   339     0    S07
    ...................uuuugauuguugcucagaaggcgu.....................................................................   9       0    S07
    ...................uuuugauuguugcucagaaggcgugcagacgucagg.........................................................   2       0    S07
    ....................uuugauuguugcucagaaggcg......................................................................   1       0    S07
    .......................................................cuuucgagcaaugaucaaacgu...................................   41      0    S07

    ...................uuuugauuguugcucaga...........................................................................   1       0    S01
    ...................uuuugauuguugcucagaagg........................................................................   1       0    S01
    ...................uuuugauuguugcucagaaggc.......................................................................   2       0    S01
    ...................uuuugauuguugcucagaaggcg......................................................................   17      0    S01
    .......................................................cuuucgagcaaugaucaaacgu...................................   2       0    S01

    ..................cuuuugauuguugcucagaaggc.......................................................................   1       0    S06
    ...................uuuugauuguugcucaga...........................................................................   1       0    S06
    ...................uuuugauuguugcucagaagg........................................................................   65      0    S06
    ...................uuuugauuguugcucagaaggc.......................................................................   39      0    S06
    ...................uuuugauuguugcucagaaggcg......................................................................   488     0    S06
    ...................uuuugauuguugcucagaaggcgu.....................................................................   4       0    S06
    ...................uuuugauuguugcucagaaggcgugcagacgucagg.........................................................   2       0    S06
    ....................uuugauuguugcucagaaggcg......................................................................   1       0    S06
    .......................................................cuuucgagcaaugaucaaacg....................................   2       0    S06
    .......................................................cuuucgagcaaugaucaaacgu...................................   22      0    S06
    ........................................................uuucgagcaaugaucaaacguu..................................   1       0    S06

    .................ccuuuugauuguugcucagaag.........................................................................   1       0    S10
    ..................cuuuugauuguugcucagaaggc.......................................................................   15      0    S10
    ...................uuuugauuguugcucaga...........................................................................   5       0    S10
    ...................uuuugauuguugcucagaa..........................................................................   4       0    S10
    ...................uuuugauuguugcucagaag.........................................................................   9       0    S10
```

```
                   Mature                             Star
guauuuaagucuucucgccuuuugauuguugcucagaaggcgugcagacgucaggcuuucgagcaaugaucaaacguugaggagggccaaauucaacacuuucguuacacac
...................uuuugauuguugcucagaagg........................................................................   131    0    S10
...................uuuugauuguugcucagaaggc.......................................................................   218    0    S10
...................uuuugauuguugcucagaaggcg......................................................................   5017   0    S10
...................uuuugauuguugcucagaaggcgu.....................................................................   21     0    S10
...................uuuugauuguugcucagaaggcgugcagacgucagg.........................................................   43     0    S10
...................uuuugauuguugcucagaaggcgugcagacgucaggc........................................................   3      0    S10
...................uuuugauuguugcucagaaggcgugcagacgucaggcu.......................................................   1      0    S10
....................uuugauuguugcucagaaggc.......................................................................   1      0    S10
....................uuugauuguugcucagaaggcg......................................................................   20     0    S10
....................uuugauuguugcucagaaggcgu.....................................................................   3      0    S10
....................uuugauuguugcucagaaggcgugcagacgucagg.........................................................   2      0    S10
......................ugauuguugcucagaaggcg......................................................................   1      0    S10
..........................................ugcagacgucaggcuuucgagcaaugaucaaacgu...................................   1      0    S10
.....................................................ggcuuucgagcaaugaucaaacg....................................   1      0    S10
......................................................gcuuucgagcaaugaucaaacg....................................   2      0    S10
.......................................................cuuucgagcaaugaucaaacg....................................   4      0    S10
.......................................................cuuucgagcaaugaucaaacgu...................................   367    0    S10
.......................................................cuuucgagcaaugaucaaacguu..................................   1      0    S10
........................................................uuucgagcaaugaucaaacg....................................   1      0    S10
........................................................uuucgagcaaugaucaaacgu...................................   16     0    S10
........................................................uuucgagcaaugaucaaacguu..................................   1      0    S10
.........................................................uucgagcaaugaucaaacguug.................................   6      0    S10
.............................................................................ugaggagggccaaauucaacacu............   1      0    S10

...................uuuugauuguugcucagaa..........................................................................   2      0    S05
...................uuuugauuguugcucagaagg........................................................................   20     0    S05
...................uuuugauuguugcucagaaggc.......................................................................   30     0    S05
...................uuuugauuguugcucagaaggcg......................................................................   475    0    S05
...................uuuugauuguugcucagaaggcgu.....................................................................   6      0    S05
...................uuuugauuguugcucagaaggcgugcagacgucagg.........................................................   5      0    S05
....................uuugauuguugcucagaaggc.......................................................................   1      0    S05
....................uuugauuguugcucagaaggcg......................................................................   3      0    S05
....................uuugauuguugcucagaaggcgu.....................................................................   1      0    S05
.......................................................cuuucgagcaaugaucaaacgu...................................   25     0    S05
........................................................uuucgagcaaugaucaaacgu...................................   1      0    S05

...................uuuugauuguugcucagaagg........................................................................   1      0    S02
...................uuuugauuguugcucagaaggcg......................................................................   19     0    S02
....................uuugauuguugcucagaaggcg......................................................................   1      0    S02
.......................................................cuuucgagcaaugaucaaacgu...................................   3      0    S02

..................cuuuugauuguugcucagaaggc.......................................................................   2      0    S04
...................uuuugauuguugcucagaagg........................................................................   19     0    S04
...................uuuugauuguugcucagaaggc.......................................................................   23     0    S04
...................uuuugauuguugcucagaaggcg......................................................................   510    0    S04
...................uuuugauuguugcucagaaggcgu.....................................................................   3      0    S04
...................uuuugauuguugcucagaaggcgugcagacgucagg.........................................................   6      0    S04
....................uuugauuguugcucagaaggcg......................................................................   1      0    S04
..........................................ugcagacgucaggcuuucgagcaaugauc.........................................   1      0    S04
.......................................................cuuucgagcaaugaucaaacgu...................................   24     0    S04

..................cuuuugauuguugcucagaagg........................................................................   1      0    S08
..................cuuuugauuguugcucagaaggc.......................................................................   5      0    S08
...................uuuugauuguugcucagaag.........................................................................   1      0    S08
...................uuuugauuguugcucagaagg........................................................................   24     0    S08
...................uuuugauuguugcucagaaggc.......................................................................   29     0    S08
...................uuuugauuguugcucagaaggcg......................................................................   261    0    S08
...................uuuugauuguugcucagaaggcgu.....................................................................   3      0    S08
...................uuuugauuguugcucagaaggcgugca..................................................................   2      0    S08
...................uuuugauuguugcucagaaggcgugcagacgucagg.........................................................   1      0    S08
...................uuuugauuguugcucagaaggcgugcagacgucaggcuuucgagcaaugaucaaacgu...................................   1      0    S08
.....................uugauuguugcucagaaggcg......................................................................   1      0    S08
.............................................agacgucaggcuuucgagc................................................   1      0    S08
.......................................................cuuucgagcaaugaucaaacg....................................   1      0    S08
.......................................................cuuucgagcaaugaucaaacgu...................................   26     0    S08
........................................................uuucgagcaaugaucaaacgu...................................   3      0    S08
.........................................................uucgagcaaugaucaaacguug.................................   1      0    S08
...................................................................gaucaaacguugaggagggcca.......................   1      0    S08

...................uuuugauuguugcucagaagg........................................................................   20     0    S03
```

Mature **Star**

```
guauuuaagucuucucgccuuuugauuguugcucagaaggcgugcagacgucaggcuuucgagcaaugaucaaacguugaggagggccaaauucaacacuuucguuacacac
...................uuuugauuguugcucagaaggc.......................................................................   16    0   S03
...................uuuugauuguugcucagaaggcg......................................................................   284   0   S03
...................uuuugauuguugcucagaaggcgu.....................................................................   4     0   S03
...................uuuugauuguugcucagaaggcgugcagacgucagg.........................................................   2     0   S03
....................uuugauuguugcucagaaggcg......................................................................   2     0   S03
.......................................................cuuucgagcaaugaucaaacgu...................................   11    0   S03
.........................................................uucgagcaaugaucaaacguug.................................   1     0   S03
..........................................................ucgagcaaugaucaaacgu...................................   2     0   S03

..................cuuuugauuguugcucagaaggc.......................................................................   2     0   S09
...................uuuugauuguugcucaga...........................................................................   5     0   S09
...................uuuugauuguugcucagaa..........................................................................   2     0   S09
...................uuuugauuguugcucagaag.........................................................................   8     0   S09
...................uuuugauuguugcucagaagg........................................................................   93    0   S09
...................uuuugauuguugcucagaaggc.......................................................................   106   0   S09
...................uuuugauuguugcucagaaggcg......................................................................   817   0   S09
...................uuuugauuguugcucagaaggcgu.....................................................................   19    0   S09
...................uuuugauuguugcucagaaggcgugca..................................................................   1     0   S09
...................uuuugauuguugcucagaaggcgugcagacgucagg.........................................................   7     0   S09
....................uuugauuguugcucagaaggcg......................................................................   3     0   S09
....................uuugauuguugcucagaaggcgu.....................................................................   1     0   S09
.....................uugauuguugcucagaaggcg......................................................................   1     0   S09
.......................................................cuuucgagcaaugaucaaacg....................................   1     0   S09
.......................................................cuuucgagcaaugaucaaacgu...................................   52    0   S09
........................................................uuucgagcaaugaucaaacgu...................................   2     0   S09
.........................................................uucgagcaaugaucaaacguug.................................   1     0   S09
```

Provisional ID	:	Scaffold633_15236
Score total	:	2384.1
Score for star read(s)	:	3.9
Score for read counts	:	2381
Score for mfe	:	1.4
Score for randfold	:	-2.2
Score for cons. seed	:	
Total read count	:	4682
Mature read count	:	4344
Loop read count	:	5
Star read count	:	333

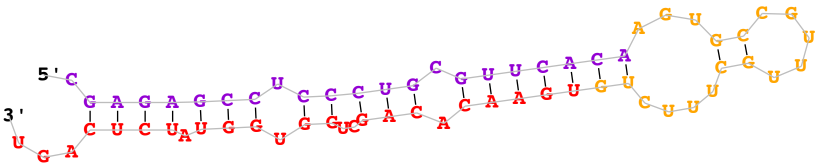

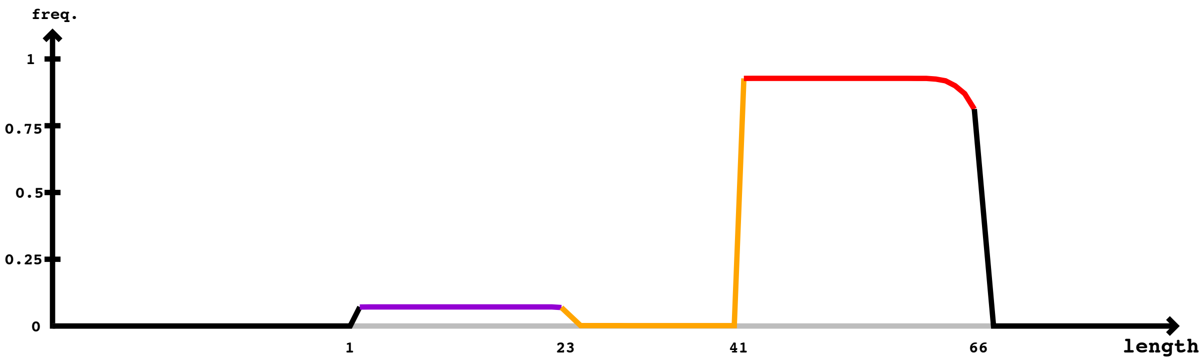


```
                                Star                                  Mature
5'- uauggagaaauuaaaaagcacuucuucugccgagagccucccugcguucacaagugccguuugcuuucugugaacacagcugguggguaucucaguggaacuugugugacgucauc  -3'   obs
    uauggagaaauuaaaaagcacuucuucugccgagagccucccugcguucacaagugccguuugcuuucugugaacacagcugguggguaucucaguggaacuugugugacgucauc        exp
    .................((((...(((..(.(((((((.(((((.(((((((...((.....))....))))))).)))..)).))).)))).)..)))...)))).........   reads   mm   sample
    ..............................cgagagccucccugcguucac................................................................   3       0    S08
    ..............................cgagagccucccugcguucaca...............................................................   119     0    S08
    ...............................gagagccucccugcguucaca...............................................................   2       0    S08
    ......................................................................ugaacacagcuggugguaucu........................   6       0    S08
    ......................................................................ugaacacagcuggugguaucuc.......................   8       0    S08
    ......................................................................ugaacacagcuggugguaucuca......................   10      0    S08
    ......................................................................ugaacacagcuggugguaucucag.....................   72      0    S08
    ......................................................................ugaacacagcuggugguaucucagu....................   771     0    S08

    ..............................cgagagccucccugcguucac................................................................   4       0    S09
    ..............................cgagagccucccugcguucaca...............................................................   41      0    S09
    ....................................................agugccguuugcuuucug.............................................   1       0    S09
    ......................................................................ugaacacagcuggugguauc.........................   1       0    S09
    ......................................................................ugaacacagcuggugguaucuc.......................   11      0    S09
    ......................................................................ugaacacagcuggugguaucuca......................   38      0    S09
    ......................................................................ugaacacagcuggugguaucucag.....................   38      0    S09
    ......................................................................ugaacacagcuggugguaucucagu....................   477     0    S09

    ..............................cgagagccucccugcguucaca...............................................................   2       0    S03
    ......................................................................ugaacacagcuggugguauc.........................   2       0    S03
    ......................................................................ugaacacagcuggugguaucu........................   9       0    S03
    ......................................................................ugaacacagcuggugguaucuc.......................   6       0    S03
    ......................................................................ugaacacagcuggugguaucuca......................   1       0    S03
    ......................................................................ugaacacagcuggugguaucucag.....................   2       0    S03
    ......................................................................ugaacacagcuggugguaucucagu....................   36      0    S03

    ..............................cgagagccucccugcguucaca...............................................................   3       0    S04
    ......................................................................ugaacacagcuggugguauc.........................   2       0    S04
    ......................................................................ugaacacagcuggugguaucu........................   1       0    S04
    ......................................................................ugaacacagcuggugguaucuc.......................   4       0    S04
    ......................................................................ugaacacagcuggugguaucucag.....................   4       0    S04
    ......................................................................ugaacacagcuggugguaucucagu....................   72      0    S04

    ......................................................................ugaacacagcuggugguauc.........................   3       0    S02
```

Star **Mature**

```
uauggagaaauuaaaaagcacuucuucugccgagagccucccugcguucacaagugccguuugcuuucugugaacacagcuggugguaucucaguggaacuugugugacgucauc
......................................................................ugaacacagcuggugguaucu........................      2      0      S02
......................................................................ugaacacagcuggugguaucuc.......................      6      0      S02
......................................................................ugaacacagcuggugguaucucag.....................      1      0      S02
......................................................................ugaacacagcuggugguaucucagu....................      73     0      S02

..............................cgagagccucccugcguucaca...............................................................      51     0      S10
..............................cgagagccucccugcguucacaagugcc.........................................................      1      0      S10
....................................................agugccguuugcuuucug.............................................      1      0      S10
......................................................................ugaacacagcuggugguaucu........................      5      0      S10
......................................................................ugaacacagcuggugguaucuc.......................      23     0      S10
......................................................................ugaacacagcuggugguaucuca......................      55     0      S10
......................................................................ugaacacagcuggugguaucucag.....................      86     0      S10
......................................................................ugaacacagcuggugguaucucagu....................      1182   0      S10
.......................................................................gaacacagcuggugguaucucagu....................      1      0      S10

..............................cgagagccucccugcguucaca...............................................................      2      0      S05
......................................................................ugaacacagcuggugguauc.........................      1      0      S05
......................................................................ugaacacagcuggugguaucu........................      2      0      S05
......................................................................ugaacacagcuggugguaucuc.......................      4      0      S05
......................................................................ugaacacagcuggugguaucuca......................      3      0      S05
......................................................................ugaacacagcuggugguaucucag.....................      1      0      S05
......................................................................ugaacacagcuggugguaucucagu....................      107    0      S05

..............................cgagagccucccugcguucac................................................................      3      0      S07
..............................cgagagccucccugcguucaca...............................................................      98     0      S07
..............................cgagagccucccugcguucacaagugccguuugcuuucug.............................................      1      0      S07
....................................................agugccguuugcuuucug.............................................      3      0      S07
......................................................................ugaacacagcuggugguaucu........................      1      0      S07
......................................................................ugaacacagcuggugguaucuc.......................      17     0      S07
......................................................................ugaacacagcuggugguaucuca......................      28     0      S07
......................................................................ugaacacagcuggugguaucucag.....................      62     0      S07
......................................................................ugaacacagcuggugguaucucagu....................      816    0      S07

......................................................................ugaacacagcuggugguа...........................      1      0      S01
......................................................................ugaacacagcuggugguauc.........................      2      0      S01
......................................................................ugaacacagcuggugguaucu........................      3      0      S01
......................................................................ugaacacagcuggugguaucuc.......................      7      0      S01
......................................................................ugaacacagcuggugguaucucag.....................      1      0      S01
......................................................................ugaacacagcuggugguaucucagu....................      50     0      S01

..............................cgagagccucccugcguucac................................................................      1      0      S06
..............................cgagagccucccugcguucaca...............................................................      4      0      S06
......................................................................ugaacacagcuggugguauc.........................      1      0      S06
......................................................................ugaacacagcuggugguaucu........................      1      0      S06
......................................................................ugaacacagcuggugguaucuc.......................      1      0      S06
......................................................................ugaacacagcuggugguaucuca......................      4      0      S06
......................................................................ugaacacagcuggugguaucucag.....................      3      0      S06
......................................................................ugaacacagcuggugguaucucagu....................      220    0      S06
```

Provisional ID : Scaffold15_622
Score total : 3394.1
Score for star read(s) : 3.9
Score for read counts : 3386.9
Score for mfe : 1.7
Score for randfold : 1.6
Score for cons. seed :
Total read count : 6655
Mature read count : 6645
Loop read count : 0
Star read count : 10

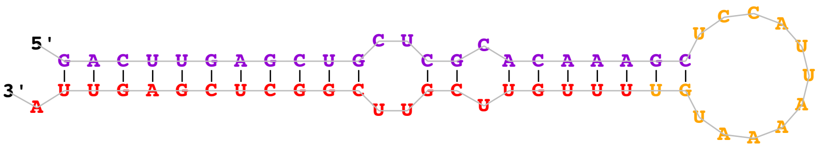

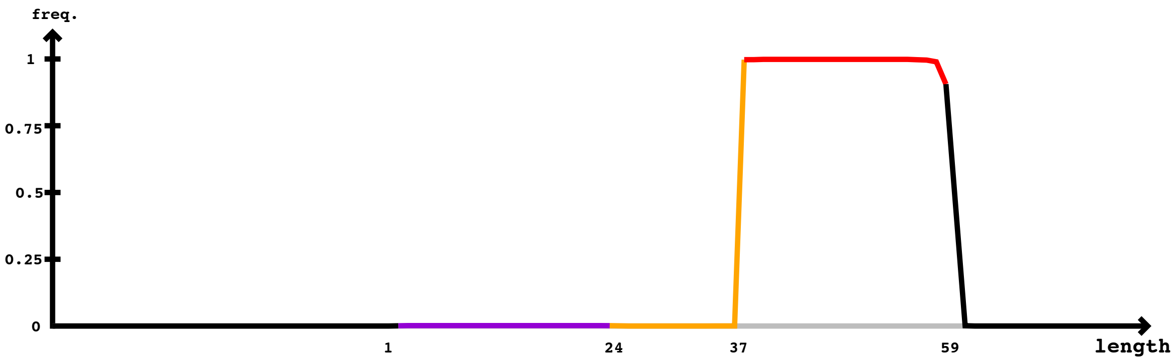

Star Mature

```
5'- aaaaaugaugugagaugacugaauuucgcuagcugacuugagcugcucgcacaaagcuccauuaaaaauguuuuguucguucggcucgaguuagggagcgauugaaagcugca  -3'   obs
    aaaaaugaugugagaugacugaauuucgcuagcugacuugagcugcucgcacaaagcuccauuaaaaauguuuuguucguucggcucgaguuagggagcgauugaaagcugca         exp
    .........(((((((......)))))))...((((((((((((..((.((((((((............))))))).))..)))))))))))))(.(((........))).).    reads   mm   sample
    .......................................................................uuuguucguucggcucgagu......................    5       0    S05
    .......................................................................uuuguucguucggcucgaguu.....................    34      0    S05
    .......................................................................uuuguucguucggcucgaguua....................    84      0    S05
    .......................................................................uuuguucguucggcucgaguuag...................    94      0    S05

    ..................................gacuugagcugcucgcacaaagc........................................................    1       0    S10
    ...................................acuugagcugcucgcacaaagc........................................................    2       0    S10
    .......................................................................uuuguucguucggcucga........................    1       0    S10
    .......................................................................uuuguucguucggcucgag.......................    2       0    S10
    .......................................................................uuuguucguucggcucgagu......................    5       0    S10
    .......................................................................uuuguucguucggcucgaguu.....................    170     0    S10
    .......................................................................uuuguucguucggcucgaguua....................    2276    0    S10
    .......................................................................uuuguucguucggcucgaguuag...................    319     0    S10
    .......................................................................uuuguucguucggcucgaguuagg..................    3       0    S10
    ........................................................................uuguucguucggcucgaguuag...................    1       0    S10
    .........................................................................uguucguucggcucgaguuag...................    2       0    S10
    .........................................................................uguucguucggcucgaguuagg..................    4       0    S10

    .......................................................................uuuguucguucggcucga........................    3       0    S01
    .......................................................................uuuguucguucggcucgagu......................    12      0    S01
    .......................................................................uuuguucguucggcucgaguu.....................    114     0    S01
    .......................................................................uuuguucguucggcucgaguua....................    252     0    S01
    .......................................................................uuuguucguucggcucgaguuag...................    6       0    S01

    .......................................................................uuuguucguucggcucgagu......................    2       0    S06
    .......................................................................uuuguucguucggcucgaguu.....................    36      0    S06
    .......................................................................uuuguucguucggcucgaguua....................    97      0    S06
    .......................................................................uuuguucguucggcucgaguuag...................    62      0    S06
    .........................................................................uguucguucggcucgaguuag...................    1       0    S06

    ...................................acuugagcugcucgcacaaag.........................................................    1       0    S07
    .......................................................................uuuguucguucggcucga........................    1       0    S07
    .......................................................................uuuguucguucggcucgag.......................    1       0    S07
```

```
                                  Star                               Mature
aaaaaugaugugagaugacugaauuucgcuagcugacuugagcugcucgcacaaagcuccauuaaaaauguuuuguucguucggcucgaguuagggagcgauugaaagcugca
.......................................................................uuuguucguucggcucgagu......................   4     0   S07
.......................................................................uuuguucguucggcucgaguu.....................   23    0   S07
.......................................................................uuuguucguucggcucgaguua....................   116   0   S07
.......................................................................uuuguucguucggcucgaguuag...................   227   0   S07

.......................................................................uuuguucguucggcucgag.......................   1     0   S03
.......................................................................uuuguucguucggcucgagu......................   2     0   S03
.......................................................................uuuguucguucggcucgaguu.....................   34    0   S03
.......................................................................uuuguucguucggcucgaguua....................   105   0   S03
.......................................................................uuuguucguucggcucgaguuag...................   60    0   S03

..................................gacuugagcugcucgcacaaagc........................................................   3     0   S09
...................................acuugagcugcucgcacaaagc........................................................   2     0   S09
....................................................................uguuuuguucguucggcucgag.......................   1     0   S09
.......................................................................uuuguucguucggcucga........................   2     0   S09
.......................................................................uuuguucguucggcucgagu......................   6     0   S09
.......................................................................uuuguucguucggcucgaguu.....................   87    0   S09
.......................................................................uuuguucguucggcucgaguua....................   1784  0   S09
.......................................................................uuuguucguucggcucgaguuag...................   145   0   S09

.......................................................................uuuguucguucggcucgag.......................   1     0   S08
.......................................................................uuuguucguucggcucgaguu.....................   7     0   S08
.......................................................................uuuguucguucggcucgaguua....................   54    0   S08
.......................................................................uuuguucguucggcucgaguuag...................   45    0   S08

.......................................................................uuuguucguucggcucga........................   2     0   S02
.......................................................................uuuguucguucggcucgag.......................   2     0   S02
.......................................................................uuuguucguucggcucgagu......................   3     0   S02
.......................................................................uuuguucguucggcucgaguu.....................   28    0   S02
.......................................................................uuuguucguucggcucgaguua....................   122   0   S02
.......................................................................uuuguucguucggcucgaguuag...................   16    0   S02

..................................gacuugagcugcucgcacaaag.........................................................   1     0   S04
.......................................................................uuuguucguucggcucgag.......................   1     0   S04
.......................................................................uuuguucguucggcucgagu......................   4     0   S04
.......................................................................uuuguucguucggcucgaguu.....................   23    0   S04
.......................................................................uuuguucguucggcucgaguua....................   83    0   S04
.......................................................................uuuguucguucggcucgaguuag...................   71    0   S04
```

Provisional ID	:	Scaffold1849_30604
Score total	:	3449
Score for star read(s)	:	3.9
Score for read counts	:	3442
Score for mfe	:	1.6
Score for randfold	:	1.6
Score for cons. seed	:	
Total read count	:	6763
Mature read count	:	6645
Loop read count	:	0
Star read count	:	118

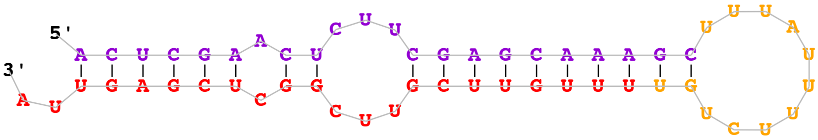

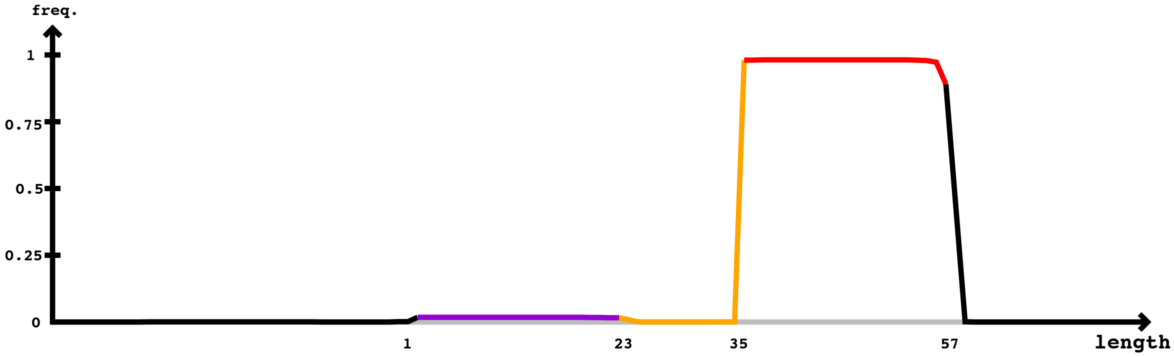

Star Mature

```
5'- aauauuuguuaaacucugaugaaaguuugcauacugacucgaacucuucgagcaaagcuuuauuuucuguuuuguucguucggcucgaguuaggcagcaaauugaaggcagc -3'  obs
    aauauuuguuaaacucugaugaaaguuugcauacugacucgaacucuucgagcaaagcuuuauuuucuguuuuguucguucggcucgaguuaggcagcaaauugaaggcagc      exp
    ...............(((.....(((((((...(((((((((.((...((((((((((..........))))))))))...)).)))))))))...))))))).....))).  reads  mm  sample
    ........uuaaacucugaugaaGgu......................................................................................  1  1  S09
    ..................................ugacucgaacucuucgagca..........................................................  1  0  S09
    ..................................ugacucgaacucuucgagcaaa........................................................  1  0  S09
    ....................................acucgaacucuucgagcaaagc......................................................  36  0  S09
    ....................................acucgaacucuucgagcaaagcu.....................................................  5  0  S09
    .......................................cgaacucuucgagcaaagc......................................................  1  0  S09
    ...................................................................uguuuuguucguucggcucgag.......................  1  0  S09
    ......................................................................uuuguucguucggcucga........................  2  0  S09
    ......................................................................uuuguucguucggcucgagu......................  6  0  S09
    ......................................................................uuuguucguucggcucgaguu.....................  87  0  S09
    ......................................................................uuuguucguucggcucgaguua....................  1784  0  S09
    ......................................................................uuuguucguucggcucgaguuag...................  145  0  S09

    ....................................acucgaacucuucgagcaaagc......................................................  1  0  S03
    ......................................................................uuuguucguucggcucgag.......................  1  0  S03
    ......................................................................uuuguucguucggcucgagu......................  2  0  S03
    ......................................................................uuuguucguucggcucgaguu.....................  34  0  S03
    ......................................................................uuuguucguucggcucgaguua....................  105  0  S03
    ......................................................................uuuguucguucggcucgaguuag...................  60  0  S03

    ....................................acucgaacucuucgagcaaagc......................................................  3  0  S08
    ....................................acucgaacucuucgagcaaagcu.....................................................  3  0  S08
    ....................................acucgaacucuucgagcaaagcuuuauuuucuguuuguucguucggc............................  1  0  S08
    ......................................................................uuuguucguucggcucgag.......................  1  0  S08
    ......................................................................uuuguucguucggcucgaguu.....................  7  0  S08
    ......................................................................uuuguucguucggcucgaguua....................  54  0  S08
    ......................................................................uuuguucguucggcucgaguuag...................  45  0  S08

    ....................................acucgaacucuucgagcaaagc......................................................  4  0  S04
    ......................................................................uuuguucguucggcucgag.......................  1  0  S04
    ......................................................................uuuguucguucggcucgagu......................  4  0  S04
    ......................................................................uuuguucguucggcucgaguu.....................  23  0  S04
    ......................................................................uuuguucguucggcucgaguua....................  83  0  S04
```

Star

Mature

```
aauauuuguuaaacucugaugaaaguuugcauacugacucgaacucuucgagcaaagcuuuauuuucuguuuuguucguucggcucgaguuaggcagcaaauugaaggcagc
......................................................................uuuguucguucggcucgaguuag...................   71     0    S04

........uuaaacucugaugaaGgu......................................................................................   2      1    S02
..................................ugacucgaacucuucgagcaaa........................................................   1      0    S02
....................................acucgaacucuucgagcaaagc......................................................   2      0    S02
......................................................................uuuguucguucggcucga........................   2      0    S02
......................................................................uuuguucguucggcucgag.......................   2      0    S02
......................................................................uuuguucguucggcucgagu......................   3      0    S02
......................................................................uuuguucguucggcucgaguu.....................   28     0    S02
......................................................................uuuguucguucggcucgaguua....................   122    0    S02
......................................................................uuuguucguucggcucgaguuag...................   16     0    S02

......................................................................uuuguucguucggcucgagu......................   5      0    S05
......................................................................uuuguucguucggcucgaguu.....................   34     0    S05
......................................................................uuuguucguucggcucgaguua....................   84     0    S05
......................................................................uuuguucguucggcucgaguuag...................   94     0    S05

........uuaaacucugaugaaGgu......................................................................................   1      1    S10
..................................ugacucgaacucuucgagca..........................................................   1      0    S10
....................................acucgaacucuucgagca..........................................................   1      0    S10
....................................acucgaacucuucgagcaaa........................................................   2      0    S10
....................................acucgaacucuucgagcaaagc......................................................   37     0    S10
....................................acucgaacucuucgagcaaagcu.....................................................   7      0    S10
....................................acucgaacucuucgagcaaagcuuuauuuucugu..........................................   1      0    S10
......................................................................uuuguucguucggcucga........................   1      0    S10
......................................................................uuuguucguucggcucgag.......................   2      0    S10
......................................................................uuuguucguucggcucgagu......................   5      0    S10
......................................................................uuuguucguucggcucgaguu.....................   170    0    S10
......................................................................uuuguucguucggcucgaguua....................   2276   0    S10
......................................................................uuuguucguucggcucgaguuag...................   319    0    S10
......................................................................uuuguucguucggcucgaguuagg..................   3      0    S10
.......................................................................uuguucguucggcucgaguuag...................   1      0    S10
........................................................................uguucguucggcucgaguuag...................   2      0    S10
........................................................................uguucguucggcucgaguuagg..................   4      0    S10

..................................ugacucgaacucuucgagcaaa........................................................   1      0    S06
....................................acucgaacucuucgagcaaagc......................................................   1      0    S06
....................................acucgaacucuucgagcaaagcu.....................................................   1      0    S06
......................................................................uuuguucguucggcucgagu......................   2      0    S06
......................................................................uuuguucguucggcucgaguu.....................   36     0    S06
......................................................................uuuguucguucggcucgaguua....................   97     0    S06
......................................................................uuuguucguucggcucgaguuag...................   62     0    S06
........................................................................uguucguucggcucgaguuag...................   1      0    S06

..................................ugacucgaacucuucgagca..........................................................   2      0    S01
..................................ugacucgaacucuucgagcaaa........................................................   2      0    S01
....................................acucgaacucuucgagcaaagc......................................................   4      0    S01
....................................acucgaacucuucgagcaaagcu.....................................................   1      0    S01
......................................................................uuuguucguucggcucga........................   3      0    S01
......................................................................uuuguucguucggcucgagu......................   12     0    S01
......................................................................uuuguucguucggcucgaguu.....................   114    0    S01
......................................................................uuuguucguucggcucgaguua....................   252    0    S01
......................................................................uuuguucguucggcucgaguuag...................   6      0    S01

......................................................................uuuguucguucggcucga........................   1      0    S07
......................................................................uuuguucguucggcucgag.......................   1      0    S07
......................................................................uuuguucguucggcucgagu......................   4      0    S07
......................................................................uuuguucguucggcucgaguu.....................   23     0    S07
......................................................................uuuguucguucggcucgaguua....................   116    0    S07
......................................................................uuuguucguucggcucgaguuag...................   227    0    S07
```

Provisional ID	:	Scaffold17_681
Score total	:	1857.2
Score for star read(s)	:	3.9
Score for read counts	:	1849.8
Score for mfe	:	2
Score for randfold	:	1.6
Score for cons. seed	:	
Total read count	:	3640
Mature read count	:	1798
Loop read count	:	0
Star read count	:	1842

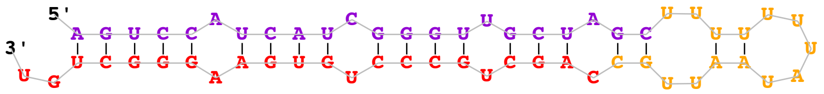

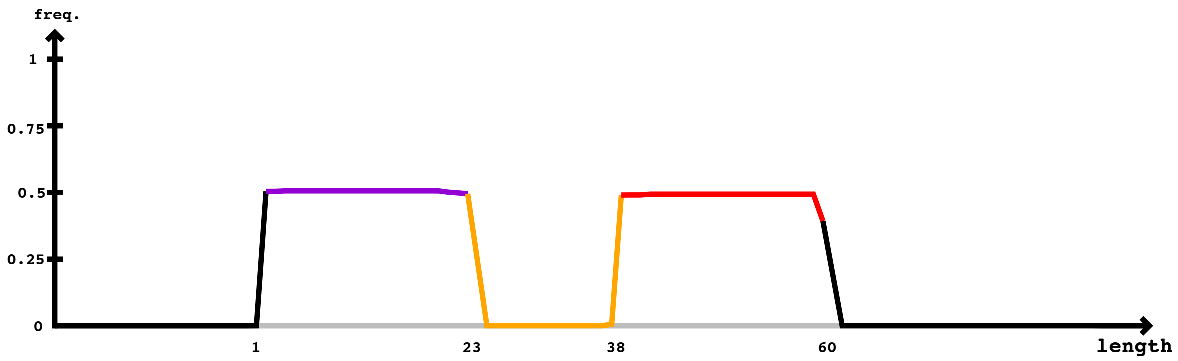

Star **Mature**

```
5'- ugauuuauuguaagaagagcaguccaucaucggguugcuagcuuuuuuuauaauugccagcugcccugugaagggcuguucuuuuuacuguuuauucaacacugccuggauc   -3'   obs
    ugauuuauuguaagaagagcaguccaucaucggguugcuagcuuuuuuuauaauugccagcugcccugugaagggcuguucuuuuuacuguuuauucaacacugccuggauc         exp
    (((...((.((((((((((((((((.((((.((((.(((.((..((.....))..)).))).)))).)))).)))))))))))))))).))....)))..............   reads   mm   sample
    ...................caguccaucaucggguugcu.........................................................................   1       0    S03
    ....................aguccaucaucggguugcuagc......................................................................   45      0    S03
    ....................aguccaucaucggguugcuagcu.....................................................................   2       0    S03
    ......................uccaucaucggguugcuagcu.....................................................................   1       0    S03
    .........................................................cagcugcccugugaagggcug..................................   7       0    S03
    .........................................................cagcugcccugugaagggcugu.................................   16      0    S03
    ............................................................cugcccugugaagggcug..................................   1       0    S03
    ...............................................................................ucuuuuuacuguuuauucaacacugccu.....   1       0    S03

    ....................aguccaucaucggguugcu.........................................................................   1       0    S09
    ....................aguccaucaucggguugcua........................................................................   1       0    S09
    ....................aguccaucaucggguugcuag.......................................................................   3       0    S09
    ....................aguccaucaucggguugcuagc......................................................................   331     0    S09
    ....................aguccaucaucggguugcuagcu.....................................................................   6       0    S09
    ....................aguccaucaucggguugcuagcuuuuuuuauaauugc.......................................................   1       0    S09
    ........................................................ccagcugcccugugaagggcug..................................   9       0    S09
    .........................................................cagcugcccugugaagggcug..................................   59      0    S09
    .........................................................cagcugcccugugaagggcugu.................................   294     0    S09
    .........................................................cagcugcccugugaagggcuguu................................   4       0    S09

    ....................aguccaucaucggguugcu.........................................................................   1       0    S08
    ....................aguccaucaucggguugcua........................................................................   1       0    S08
    ....................aguccaucaucggguugcuag.......................................................................   1       0    S08
    ....................aguccaucaucggguugcuagc......................................................................   393     0    S08
    ....................aguccaucaucggguugcuagcu.....................................................................   2       0    S08
    .....................guccaucaucggguugcuagc......................................................................   1       0    S08
    ........................................................ccagcugcccugugaagggcug..................................   2       0    S08
    .........................................................cagcugcccugugaagggcug..................................   68      0    S08
    .........................................................cagcugcccugugaagggcugu.................................   265     0    S08
    .........................................................cagcugcccugugaagggcuguu................................   6       0    S08

    ....................aguccaucaucggguugcu.........................................................................   1       0    S04
    ....................aguccaucaucggguugcuagc......................................................................   61      0    S04
```

Star **Mature**

```
ugauuuauuguaagaagagcaguccaucaucggguugcuagcuuuuuuuauaaauugccagcugcccugugaagggcuguucuuuuuacuguuuauucaacacugccuggauc
....................aguccaucaucggguugcuagcu......................................................................   2     0   S04
..........................................................cagcugcccugugaagggcug..................................   17    0   S04
..........................................................cagcugcccugugaagggcugu.................................   40    0   S04

....................aguccaucaucggguugcu..........................................................................   2     0   S02
....................aguccaucaucggguugcuag........................................................................   1     0   S02
....................aguccaucaucggguugcuagc.......................................................................   44    0   S02
......................uccaucaucggguugcuagcu......................................................................   2     0   S02
..........................................................cagcugcccugugaagggcug..................................   8     0   S02
..........................................................cagcugcccugugaagggcugu.................................   21    0   S02
..........................................................cagcugcccugugaagggcuguu................................   1     0   S02
.............................................................cugcccugugaagggcug..................................   1     0   S02

....................aguccaucaucggguugcu..........................................................................   3     0   S05
....................aguccaucaucggguugcua.........................................................................   2     0   S05
....................aguccaucaucggguugcuag........................................................................   2     0   S05
....................aguccaucaucggguugcuagc.......................................................................   69    0   S05
....................aguccaucaucggguugcuagcu......................................................................   3     0   S05
..........................................................cagcugcccugugaagggcug..................................   30    0   S05
..........................................................cagcugcccugugaagggcugu.................................   48    0   S05
..........................................................cagcugcccugugaagggcuguu................................   7     0   S05
.............................................................cugcccugugaagggcug..................................   1     0   S05

....................aguccaucaucggguugcu..........................................................................   5     0   S10
....................aguccaucaucggguugcua.........................................................................   3     0   S10
....................aguccaucaucggguugcuag........................................................................   2     0   S10
....................aguccaucaucggguugcuagc.......................................................................   452   0   S10
....................aguccaucaucggguugcuagcu......................................................................   6     0   S10
.........................................................ccagcugcccugugaagggcug..................................   8     0   S10
..........................................................cagcugcccugugaagggcug..................................   92    0   S10
..........................................................cagcugcccugugaagggcugu.................................   585   0   S10
..........................................................cagcugcccugugaagggcuguu................................   3     0   S10
...........................................................agcugcccugugaagggcugu.................................   1     0   S10

....................aguccaucaucggguugcu..........................................................................   1     0   S01
....................aguccaucaucggguugcuagc.......................................................................   6     0   S01
....................aguccaucaucggguugcuagcu......................................................................   1     0   S01
......................uccaucaucggguugcuagcu......................................................................   1     0   S01
..........................................................cagcugcccugugaagggcug..................................   17    0   S01
..........................................................cagcugcccugugaagggcugu.................................   41    0   S01
..........................................................cagcugcccugugaagggcuguu................................   2     0   S01
.............................................................cugcccugugaagggcug..................................   3     0   S01
.............................................................cugcccugugaagggcugu.................................   2     0   S01

....................aguccaucaucggguugc...........................................................................   1     0   S06
....................aguccaucaucggguugcu..........................................................................   2     0   S06
....................aguccaucaucggguugcua.........................................................................   2     0   S06
....................aguccaucaucggguugcuag........................................................................   2     0   S06
....................aguccaucaucggguugcuagc.......................................................................   142   0   S06
....................aguccaucaucggguugcuagcu......................................................................   2     0   S06
......................uccaucaucggguugcuagcu......................................................................   1     0   S06
..........................................................cagcugcccugugaagggcug..................................   32    0   S06
..........................................................cagcugcccugugaagggcugu.................................   67    0   S06
..........................................................cagcugcccugugaagggcuguu................................   2     0   S06
.............................................................cugcccugugaagggcug..................................   3     0   S06

....................aguccaucaucggguugcua.........................................................................   1     0   S07
....................aguccaucaucggguugcuagc.......................................................................   227   0   S07
....................aguccaucaucggguugcuagcu......................................................................   2     0   S07
......................uccaucaucggguugcuagcu......................................................................   1     0   S07
.........................................................ccagcugcccugugaagggcug..................................   1     0   S07
..........................................................cagcugcccugugaagggcug..................................   9     0   S07
..........................................................cagcugcccugugaagggcugu.................................   25    0   S07
```

Provisional ID	:	Scaffold3_108
Score total	:	124.1
Score for star read(s)	:	3.9
Score for read counts	:	116.9
Score for mfe	:	1.7
Score for randfold	:	1.6
Score for cons. seed	:	
Total read count	:	241
Mature read count	:	240
Loop read count	:	0
Star read count	:	1

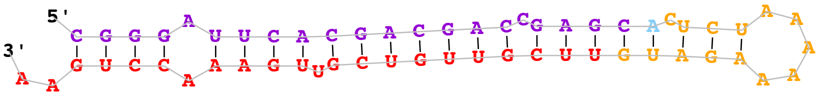

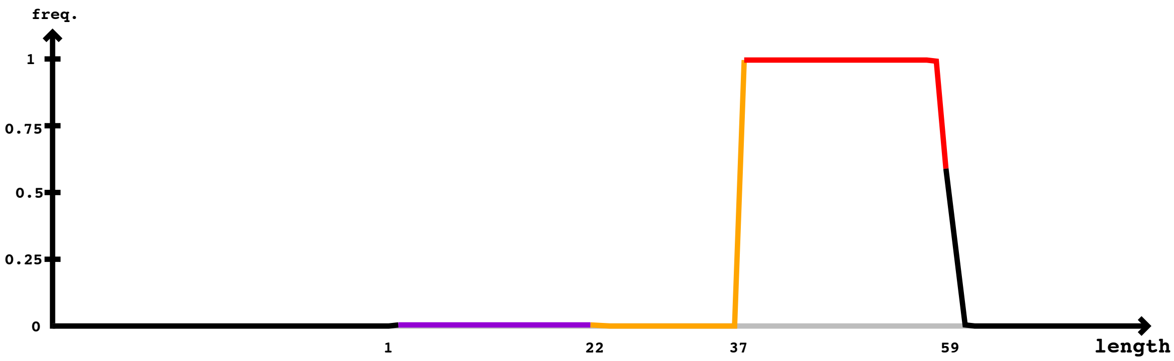

Star   Mature

```
5'- auaaauacggagcaaguagaucguuggcacugucgggauucacgacgaccgagcacucuaaaaaaagauguucguugucguugaaaccugaacgaggccuucugauagcugc  -3'   obs
    auaaauacggagcaaguagaucguuggcacugucgggauucacgacgaccgagcacucuaaaaaaagauguucguugucguugaaaccugaacgaggccuucugauagcugc         exp
    ........(.(((...((((.....(((..(((.((((.((((((((((.(((((.(((.....))))))))))))))))).)))).)))).)))..))).))))...))).)    reads    mm     sample
    ..................................cgggauucacgacgaccgagc..........................................................    1        0      S09
    ......................................................................uucguugucguugaaaccuga......................    10       0      S09
    ......................................................................uucguugucguugaaaccugaa.....................    21       0      S09
    ......................................................................uucguugucguugaaaccugaacg...................    1        0      S09

    ......................................................................uucguugucguugaaaccuga......................    4        0      S03
    ......................................................................uucguugucguugaaaccugaa.....................    6        0      S03

    ......................................................................uucguugucguugaaaccugaa.....................    5        0      S08

    ......................................................................uucguugucguugaaaccuga......................    7        0      S02
    ......................................................................uucguugucguugaaaccugaa.....................    12       0      S02

    ......................................................................uucguugucguugaaaccuga......................    27       0      S04
    ......................................................................uucguugucguugaaaccugaa.....................    20       0      S04

    ......................................................................uucguugucguugaaaccuga......................    12       0      S05
    ......................................................................uucguugucguugaaaccugaa.....................    13       0      S05

    ......................................................................uucguugucguugaaaccuga......................    15       0      S10
    ......................................................................uucguugucguugaaaccugaa.....................    34       0      S10

    ......................................................................uucguugucguugaaaccuga......................    7        0      S01
    ......................................................................uucguugucguugaaaccugaa.....................    14       0      S01

    ......................................................................uucguugucguugaaaccug.......................    1        0      S06
    ......................................................................uucguugucguugaaaccuga......................    5        0      S06
    ......................................................................uucguugucguugaaaccugaa.....................    4        0      S06

    ......................................................................uucguugucguugaaaccuga......................    10       0      S07
    ......................................................................uucguugucguugaaaccugaa.....................    12       0      S07
```

Provisional ID	:	Scaffold59_1802
Score total	:	45070
Score for star read(s)	:	3.9
Score for read counts	:	45062.5
Score for mfe	:	2
Score for randfold	:	1.6
Score for cons. seed	:	
Total read count	:	88400
Mature read count	:	64301
Loop read count	:	0
Star read count	:	24099

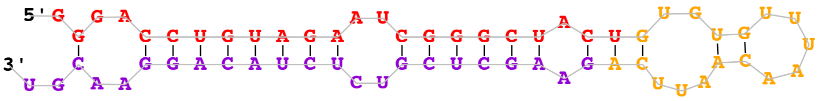

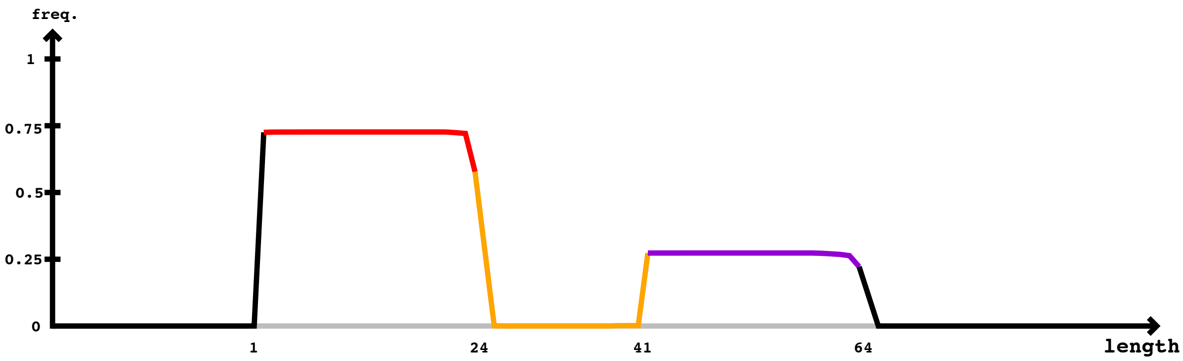

Mature　Star

5'- aauauuugguaauucgaaaugggaccuguagaaucgggcuacuguguguuuaacaauucagaagcucgucucuacaggaacguugcgaauuagcuagaucugcugaauucagu -3'	obs		
aauauuugguaauucgaaaugggaccuguagaaucgggcuacuguguguuuaacaauucagaagcucgucucuacaggaacguugcgaauuagcuagaucugcugaauucagu	exp		
...(((((((((((((.((((...((((((((..((((((.(((..((.....))...))).))))))..))))))))..)))).)))))).)))))))..((((....))))	reads	mm	sample
....................gggaccuguagaaucgggc..	1	0	S03
....................gggaccuguagaaucgggcu...	10	0	S03
....................gggaccuguagaaucgggcua..	5	0	S03
....................gggaccuguagaaucgggcuac...	430	0	S03
....................gggaccuguagaaucgggcuacu..	1489	0	S03
....................gggaccuguagaaucgggcuacug...	276	0	S03
....................gggaccuguagaaucgggcuacugu..	2	0	S03
....................gggaccuguagaaucgggcuacuguguguuuaacaau..	1	0	S03
.....................ggaccuguagaaucgggcuac...	2	0	S03
.....................ggaccuguagaaucgggcuacu..	2	0	S03
......................gaccuguagaaucgggcuacu..	1	0	S03
....ucagaagcucgucucuacagg...................................	1	0	S03
....ucagaagcucgucucuacagga..................................	3	0	S03
....ucagaagcucgucucuacaggaa.................................	8	0	S03
....ucagaagcucgucucuacaggaac................................	3	0	S03
....gaagcucgucucuacagg...................................	9	0	S03
....gaagcucgucucuacagga..................................	18	0	S03
....gaagcucgucucuacaggaa.................................	26	0	S03
....gaagcucgucucuacaggaac................................	32	0	S03
....gaagcucgucucuacaggaacg...............................	266	0	S03
....gaagcucgucucuacaggaacgu..............................	580	0	S03
....gaagcucgucucuacaggaacguu.............................	238	0	S03
....aagcucgucucuacaggaacgu..............................	1	0	S03
....aagcucgucucuacaggaacguu.............................	1	0	S03
...................ugggaccuguagaaucgggcuacu..	3	0	S09
....................gggaccuguagaaucgggc..	6	0	S09
....................gggaccuguagaaucgggcu...	57	0	S09
....................gggaccuguagaaucgggcua..	91	0	S09
....................gggaccuguagaaucgggcuac...	3906	0	S09
....................gggaccuguagaaucgggcuacu..	13397	0	S09
....................gggaccuguagaaucgggcuacug...	1854	0	S09
....................gggaccuguagaaucgggcuacugu..	11	0	S09

Mature Star

```
aauauuugguaauucgaaaugggaccuguagaaucgggcuacuguguguuuaacaauucagaagcucgucucuacaggaacguugcgaauuagcuagaucugcugaauucagu
....................gggaccuguagaaucgggcuacuguguguuuaacaauuca.....................................................  5     0  S09
.....................ggaccuguagaaucgggcuac.......................................................................  16    0  S09
.....................ggaccuguagaaucgggcuacu......................................................................  15    0  S09
......................gaccuguagaaucgggcuacu......................................................................  3     0  S09
......................gaccuguagaaucgggcuacug.....................................................................  3     0  S09
........................ccuguagaaucgggcuacug.....................................................................  1     0  S09
.........................cuguagaaucgggcuacu......................................................................  3     0  S09
.........................cuguagaaucgggcuacug.....................................................................  1     0  S09
..........................uguagaaucgggcuacug.....................................................................  1     0  S09
......................................cuacuguguguuuaacaauuca.....................................................  1     0  S09
.........................................................ucagaagcucgucucuacagga..................................  1     0  S09
.........................................................ucagaagcucgucucuacaggaa.................................  1     0  S09
.........................................................ucagaagcucgucucuacaggaac................................  1     0  S09
............................................................gaagcucgucucuacagg...................................  12    0  S09
............................................................gaagcucgucucuacagga..................................  24    0  S09
............................................................gaagcucgucucuacaggaa.................................  6     0  S09
............................................................gaagcucgucucuacaggaac................................  55    0  S09
............................................................gaagcucgucucuacaggaacg...............................  329   0  S09
............................................................gaagcucgucucuacaggaacgu..............................  3415  0  S09
............................................................gaagcucgucucuacaggaacguu.............................  363   0  S09
.............................................................aagcucgucucuacaggaacgu..............................  1     0  S09
...............................................................gcucgucucuacaggaacgu..............................  1     0  S09

....................gggaccuguagaaucgggc..........................................................................  2     0  S08
....................gggaccuguagaaucgggcu.........................................................................  9     0  S08
....................gggaccuguagaaucgggcua........................................................................  11    0  S08
....................gggaccuguagaaucgggcuac.......................................................................  544   0  S08
....................gggaccuguagaaucgggcuacu......................................................................  3356  0  S08
....................gggaccuguagaaucgggcuacug.....................................................................  776   0  S08
....................gggaccuguagaaucgggcuacugu....................................................................  5     0  S08
....................gggaccuguagaaucgggcuacuguguguuuaacaauuca.....................................................  3     0  S08
.....................ggaccuguagaaucgggcuac.......................................................................  2     0  S08
.....................ggaccuguagaaucgggcuacu......................................................................  2     0  S08
......................gaccuguagaaucgggcuacug.....................................................................  1     0  S08
........................ccuguagaaucgggcuacu......................................................................  1     0  S08
.........................cuguagaaucgggcuacu......................................................................  1     0  S08
..........................uguagaaucgggcuacug.....................................................................  1     0  S08
.........................................................ucagaagcucgucucuacag....................................  1     0  S08
.........................................................ucagaagcucgucucuacagga..................................  6     0  S08
.........................................................ucagaagcucgucucuacaggaa.................................  12    0  S08
.........................................................ucagaagcucgucucuacaggaac................................  1     0  S08
............................................................gaagcucgucucuacagg...................................  2     0  S08
............................................................gaagcucgucucuacagga..................................  23    0  S08
............................................................gaagcucgucucuacaggaa.................................  22    0  S08
............................................................gaagcucgucucuacaggaac................................  58    0  S08
............................................................gaagcucgucucuacaggaacg...............................  645   0  S08
............................................................gaagcucgucucuacaggaacgu..............................  2043  0  S08
............................................................gaagcucgucucuacaggaacguu.............................  454   0  S08
.............................................................aagcucgucucuacaggaacgu..............................  2     0  S08
.................................................................ucgucucuacaggaacgu..............................  1     0  S08

....................gggaccuguagaaucgggc..........................................................................  2     0  S04
....................gggaccuguagaaucgggcu.........................................................................  31    0  S04
....................gggaccuguagaaucgggcua........................................................................  18    0  S04
....................gggaccuguagaaucgggcuac.......................................................................  1063  0  S04
....................gggaccuguagaaucgggcuacu......................................................................  5271  0  S04
....................gggaccuguagaaucgggcuacug.....................................................................  919   0  S04
....................gggaccuguagaaucgggcuacugu....................................................................  9     0  S04
.....................ggaccuguagaaucgggcuac.......................................................................  3     0  S04
.....................ggaccuguagaaucgggcuacu......................................................................  1     0  S04
.....................ggaccuguagaaucgggcuacug.....................................................................  1     0  S04
........................ccuguagaaucgggcuacu......................................................................  1     0  S04
.........................cuguagaaucgggcuacu......................................................................  1     0  S04
.........................................................ucagaagcucgucucuacagg...................................  1     0  S04
.........................................................ucagaagcucgucucuacagga..................................  2     0  S04
.........................................................ucagaagcucgucucuacaggaa.................................  4     0  S04
.........................................................ucagaagcucgucucuacaggaac................................  4     0  S04
...........................................................agaagcucgucucuacaggaacg...............................  1     0  S04
............................................................gaagcucgucucuacagg...................................  6     0  S04
............................................................gaagcucgucucuacagga..................................  14    0  S04
```

```
                    Mature                                       Star
aauauuugguaauucgaaaugggaccuguagaaucgggcuacuguguguuuaacaauucagaagcucgucucuacaggaacguugcgaauuagcuagaucugcugaauucagu
............................................................gaagcucgucucuacaggaa.................................   20      0   S04
............................................................gaagcucgucucuacaggaac................................   27      0   S04
............................................................gaagcucgucucuacaggaacg...............................   343     0   S04
............................................................gaagcucgucucuacaggaacgu..............................   822     0   S04
............................................................gaagcucgucucuacaggaacguu.............................   258     0   S04

....................gggaccuguagaaucgggcu.........................................................................   2       0   S02
....................gggaccuguagaaucgggcua........................................................................   2       0   S02
....................gggaccuguagaaucgggcuac.......................................................................   56      0   S02
....................gggaccuguagaaucgggcuacu......................................................................   535     0   S02
....................gggaccuguagaaucgggcuacug.....................................................................   117     0   S02
....................gggaccuguagaaucgggcuacugu....................................................................   2       0   S02
........................ccuguagaaucgggcuacu......................................................................   1       0   S02
.........................................................ucagaagcucgucucuacaggaa.................................   1       0   S02
.........................................................ucagaagcucgucucuacaggaac................................   1       0   S02
............................................................gaagcucgucucuacagg...................................   9       0   S02
............................................................gaagcucgucucuacagga..................................   11      0   S02
............................................................gaagcucgucucuacaggaa.................................   12      0   S02
............................................................gaagcucgucucuacaggaac................................   27      0   S02
............................................................gaagcucgucucuacaggaacg...............................   140     0   S02
............................................................gaagcucgucucuacaggaacgu..............................   255     0   S02
............................................................gaagcucgucucuacaggaacguu.............................   68      0   S02

....................gggaccuguagaaucgggc..........................................................................   1       0   S05
....................gggaccuguagaaucgggcu.........................................................................   16      0   S05
....................gggaccuguagaaucgggcua........................................................................   13      0   S05
....................gggaccuguagaaucgggcuac.......................................................................   565     0   S05
....................gggaccuguagaaucgggcuacu......................................................................   2266    0   S05
....................gggaccuguagaaucgggcuacug.....................................................................   387     0   S05
....................gggaccuguagaaucgggcuacugu....................................................................   5       0   S05
.....................ggaccuguagaaucgggcuac.......................................................................   3       0   S05
.....................ggaccuguagaaucgggcuacu......................................................................   4       0   S05
......................gaccuguagaaucgggcuacu......................................................................   2       0   S05
.........................................................ucagaagcucgucucuacagga..................................   2       0   S05
.........................................................ucagaagcucgucucuacaggaa.................................   9       0   S05
.........................................................ucagaagcucgucucuacaggaac................................   4       0   S05
............................................................gaagcucgucucuacagg...................................   12      0   S05
............................................................gaagcucgucucuacagga..................................   15      0   S05
............................................................gaagcucgucucuacaggaa.................................   17      0   S05
............................................................gaagcucgucucuacaggaac................................   20      0   S05
............................................................gaagcucgucucuacaggaacg...............................   118     0   S05
............................................................gaagcucgucucuacaggaacgu..............................   304     0   S05
............................................................gaagcucgucucuacaggaacguu.............................   105     0   S05
.............................................................aagcucgucucuacaggaacguu.............................   1       0   S05
.................................................................ucgucucuacaggaacguu.............................   1       0   S05

...................ugggaccuguagaaucgggcuacu......................................................................   2       0   S10
....................gggaccuguagaaucggg...........................................................................   1       0   S10
....................gggaccuguagaaucgggc..........................................................................   1       0   S10
....................gggaccuguagaaucgggcu.........................................................................   57      0   S10
....................gggaccuguagaaucgggcua........................................................................   87      0   S10
....................gggaccuguagaaucgggcuac.......................................................................   4151    0   S10
....................gggaccuguagaaucgggcuacu......................................................................   11608   0   S10
....................gggaccuguagaaucgggcuacug.....................................................................   1790    0   S10
....................gggaccuguagaaucgggcuacugu....................................................................   3       0   S10
....................gggaccuguagaaucgggcuacuguguguuuaacaauuc......................................................   1       0   S10
....................gggaccuguagaaucgggcuacuguguguuuaacaauuca.....................................................   1       0   S10
.....................ggaccuguagaaucgggcua........................................................................   2       0   S10
.....................ggaccuguagaaucgggcuac.......................................................................   12      0   S10
.....................ggaccuguagaaucgggcuacu......................................................................   11      0   S10
.....................ggaccuguagaaucgggcuacug.....................................................................   1       0   S10
......................gaccuguagaaucgggcuac.......................................................................   1       0   S10
......................gaccuguagaaucgggcuacu......................................................................   2       0   S10
......................gaccuguagaaucgggcuacug.....................................................................   1       0   S10
.......................accuguagaaucgggcuacu......................................................................   1       0   S10
.......................accuguagaaucgggcuacug.....................................................................   2       0   S10
.........................cuguagaaucgggcuacu......................................................................   2       0   S10
.........................................................ucagaagcucgucucuacagg...................................   1       0   S10
.........................................................ucagaagcucgucucuacagga..................................   4       0   S10
.........................................................ucagaagcucgucucuacaggaa.................................   1       0   S10
```

Mature **Star**

```
aauauuugguaauucgaaaugggaccuguagaaucgggcuacugugugu uuaacaauucagaagcucgucucuacaggaacguugcgaauuagcuagaucugcugaauucagu
............................................................gaagcucgucucuacagg...................................  2     0  S10
............................................................gaagcucgucucuacagga..................................  13    0  S10
............................................................gaagcucgucucuacaggaa.................................  5     0  S10
............................................................gaagcucgucucuacaggaac................................  67    0  S10
............................................................gaagcucgucucuacaggaacg...............................  716   0  S10
............................................................gaagcucgucucuacaggaacgu..............................  5779  0  S10
............................................................gaagcucgucucuacaggaacguu.............................  523   0  S10
.............................................................aagcucgucucuacaggaacgu..............................  2     0  S10
................................................................cucgucucuacaggaacgu..............................  1     0  S10

....................gggaccuguagaaucgggc..........................................................................  1     0  S06
....................gggaccuguagaaucgggcu.........................................................................  12    0  S06
....................gggaccuguagaaucgggcua........................................................................  3     0  S06
....................gggaccuguagaaucgggcuac.......................................................................  504   0  S06
....................gggaccuguagaaucgggcuacu......................................................................  2130  0  S06
....................gggaccuguagaaucgggcuacug.....................................................................  394   0  S06
....................gggaccuguagaaucgggcuacugu....................................................................  8     0  S06
.....................ggaccuguagaaucgggcuac.......................................................................  1     0  S06
.....................ggaccuguagaaucgggcuacu......................................................................  1     0  S06
......................gaccuguagaaucgggcuacu......................................................................  2     0  S06
.....................................gcuacugugugu uuaacaauuca.....................................................  1     0  S06
.........................................................ucagaagcucgucucuacagga..................................  2     0  S06
.........................................................ucagaagcucgucucuacaggaa.................................  6     0  S06
............................................................gaagcucgucucuacagg...................................  6     0  S06
............................................................gaagcucgucucuacagga..................................  11    0  S06
............................................................gaagcucgucucuacaggaa.................................  23    0  S06
............................................................gaagcucgucucuacaggaac................................  30    0  S06
............................................................gaagcucgucucuacaggaacg...............................  370   0  S06
............................................................gaagcucgucucuacaggaacgu..............................  665   0  S06
............................................................gaagcucgucucuacaggaacguu.............................  277   0  S06
.............................................................aagcucgucucuacaggaacgu..............................  1     0  S06
..............................................................agcucgucucuacaggaacguu.............................  1     0  S06

....................gggaccuguagaaucgggcuac.......................................................................  4     0  S01
....................gggaccuguagaaucgggcuacu......................................................................  12    0  S01
....................gggaccuguagaaucgggcuacug.....................................................................  2     0  S01
.........................................................ucagaagcucgucucuacagg...................................  1     0  S01
.........................................................ucagaagcucgucucuacaggaa.................................  1     0  S01
............................................................gaagcucgucucuacagg...................................  7     0  S01
............................................................gaagcucgucucuacagga..................................  12    0  S01
............................................................gaagcucgucucuacaggaa.................................  6     0  S01
............................................................gaagcucgucucuacaggaac................................  26    0  S01
............................................................gaagcucgucucuacaggaacg...............................  86    0  S01
............................................................gaagcucgucucuacaggaacgu..............................  155   0  S01
............................................................gaagcucgucucuacaggaacguu.............................  22    0  S01

...................ugggaccuguagaaucgggcuacug.....................................................................  1     0  S07
...................ugggaccuguagaaucgggcuacugu....................................................................  1     0  S07
....................gggaccuguagaaucggg...........................................................................  1     0  S07
....................gggaccuguagaaucgggcu.........................................................................  18    0  S07
....................gggaccuguagaaucgggcua........................................................................  29    0  S07
....................gggaccuguagaaucgggcuac.......................................................................  1433  0  S07
....................gggaccuguagaaucgggcuacu......................................................................  3753  0  S07
....................gggaccuguagaaucgggcuacug.....................................................................  650   0  S07
....................gggaccuguagaaucgggcuacugu....................................................................  1     0  S07
....................gggaccuguagaaucgggcuacugugugu uuaacaauuca.....................................................  3     0  S07
.....................ggaccuguagaaucgggcuac.......................................................................  4     0  S07
.....................ggaccuguagaaucgggcuacu......................................................................  5     0  S07
.....................ggaccuguagaaucgggcuacug.....................................................................  1     0  S07
......................gaccuguagaaucgggcuacu......................................................................  1     0  S07
.........................................................ucagaagcucgucucuacagga..................................  6     0  S07
.........................................................ucagaagcucgucucuacaggaa.................................  8     0  S07
.........................................................ucagaagcucgucucuacaggaac................................  3     0  S07
............................................................gaagcucgucucuacagg...................................  8     0  S07
............................................................gaagcucgucucuacagga..................................  7     0  S07
............................................................gaagcucgucucuacaggaa.................................  18    0  S07
............................................................gaagcucgucucuacaggaac................................  69    0  S07
............................................................gaagcucgucucuacaggaacg...............................  631   0  S07
............................................................gaagcucgucucuacaggaacgu..............................  2667  0  S07
............................................................gaagcucgucucuacaggaacguu.............................  658   0  S07
```

```
                   Mature                                  Star
aauauuugguaauucgaaaugggaccuguagaaucgggcuacugugugu uuaacaauucagaagcucgucucuacaggaacguugcgaauuagcuagaucugcugaauucagu
.............................................................aagcucgucucuacaggaacguu.............................	1	0	S07
..............................................................agcucgucucuacaggaacguu.............................	1	0	S07
```

Provisional ID : Scaffold2243_34190
Score total : 10194.9
Score for star read(s) : 3.9
Score for read counts : 10187.5
Score for mfe : 2
Score for randfold : 1.6
Score for cons. seed :
Total read count : 19994
Mature read count : 18690
Loop read count : 432
Star read count : 872

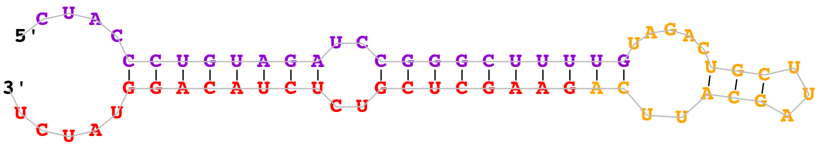

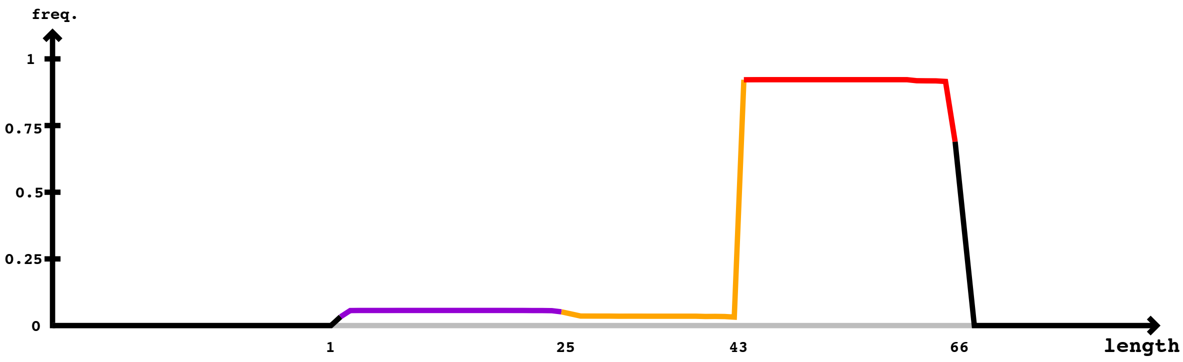

Star Mature

```
5'- ggauguuaucucucuagccguucguaaacuacccuguagauccgggcuuuuguagacugcuuagcauucagaagcucgucucuacagguaucuugcgaacgaugcugauugca   -3'   obs
    ggauguuaucucucuagccguucguaaacuacccuguagauccgggcuuuuguagacugcuuagcauucagaagcucgucucuacagguaucuugcgaacgaugcugauugca         exp
    ...(((.(((.....(((((((((((..((((.((((((..(((((((((((.....(((...)))..))))))))))..))))))))))..)))))))))..)))))).)))   reads   mm   sample
    ............................cuacccuguagauccgggcuuu...............................................................   1       0    S07
    ............................cuacccuguagauccgggcuuuu..............................................................   1       0    S07
    ............................cuacccuguagauccgggcuuuug.............................................................   4       0    S07
    ............................cuacccuguagauccgggcuuuugu............................................................   1       0    S07
    ............................cuacccuguagauccgggcuuuuguagac........................................................   1       0    S07
    ............................cuacccuguagauccgggcuuuuguagacugcuuagca...............................................   2       0    S07
    ............................cuacccuguagauccgggcuuuuguagacugcuuagcauuc............................................   4       0    S07
    ............................cuacccuguagauccgggcuuuuguagacugcuuagcauuca...........................................   15      0    S07
    .............................uacccuguagauccgggcuuuu..............................................................   1       0    S07
    .............................uacccuguagauccgggcuuuug.............................................................   3       0    S07
    .............................uacccuguagauccgggcuuuugu............................................................   2       0    S07
    .............................uacccuguagauccgggcuuuuguagac........................................................   1       0    S07
    .............................uacccuguagauccgggcuuuuguagacugcuua..................................................   1       0    S07
    .............................uacccuguagauccgggcuuuuguagacugcuuagca...............................................   4       0    S07
    .............................uacccuguagauccgggcuuuuguagacugcuuagcauu.............................................   2       0    S07
    .............................uacccuguagauccgggcuuuuguagacugcuuagcauuc............................................   2       0    S07
    .............................uacccuguagauccgggcuuuuguagacugcuuagcauuca...........................................   38      0    S07
    ..............................acccuguagauccgggcuuuuguagacugcuuagcauuca...........................................   1       0    S07
    ..................................uguagauccgggcuuuuguagacugcuuagca...............................................   1       0    S07
    ....................................................uagacugcuuagcauuca...........................................   48      0    S07
    ....................................................uagacugcuuagcauucagaagcucgucucuacagguaucu....................   1       0    S07
    ......................................................................gaagcucgucucuacagg.........................   8       0    S07
    ......................................................................gaagcucgucucuacaggu........................   1       0    S07
    ......................................................................gaagcucgucucuacagguau......................   6       0    S07
    ......................................................................gaagcucgucucuacagguauc.....................   515     0    S07
    ......................................................................gaagcucgucucuacagguaucu....................   1379    0    S07
    ......................................................................gaagcucgucucuacagguaucuu...................   15      0    S07

    ...................................................................ucagaagcucgucucuacagg.........................   1       0    S01
    ......................................................................gaagcucgucucuacagg.........................   7       0    S01
    ......................................................................gaagcucgucucuacagguau......................   1       0    S01
    ......................................................................gaagcucgucucuacagguauc.....................   25      0    S01
    ......................................................................gaagcucgucucuacagguaucu....................   63      0    S01
```

```
                            Star                                         Mature
ggauguuaucucucuagccguucguaaacuacccuguagauccgggcuuuuguagacugcuuagcauucagaagcucgucucuacagguaucuugcgaacgaugcugauugca

............................cuacccuguagauccgggcuuuu..............................................................    1      0      S06
............................cuacccuguagauccgggcuuuug.............................................................    3      0      S06
............................cuacccuguagauccgggcuuuugu............................................................    1      0      S06
............................cuacccuguagauccgggcuuuuguaga.........................................................    1      0      S06
............................cuacccuguagauccgggcuuuuguagacugcuuagcauuca...........................................    3      0      S06
.............................uacccuguagauccgggcuuu...............................................................    1      0      S06
.............................uacccuguagauccgggcuuuu..............................................................    1      0      S06
.............................uacccuguagauccgggcuuuug.............................................................    1      0      S06
.............................uacccuguagauccgggcuuuugu............................................................    1      0      S06
.............................uacccuguagauccgggcuuuugua...........................................................    1      0      S06
.............................uacccuguagauccgggcuuuuguagacugcuuagcauuca...........................................    3      0      S06
....................................................uagacugcuuagcauuca...........................................    10     0      S06
......................................................................gaagcucgucucuacagg.........................    6      0      S06
......................................................................gaagcucgucucuacaggu........................    1      0      S06
......................................................................gaagcucgucucuacagguauc.....................    231    0      S06
......................................................................gaagcucgucucuacagguaucu....................    198    0      S06
......................................................................gaagcucgucucuacagguaucuu...................    2      0      S06

............................cuacccuguagauccggg...................................................................    2      0      S10
............................cuacccuguagauccgggcu.................................................................    4      0      S10
............................cuacccuguagauccgggcuu................................................................    1      0      S10
............................cuacccuguagauccgggcuuu...............................................................    1      0      S10
............................cuacccuguagauccgggcuuuu..............................................................    23     0      S10
............................cuacccuguagauccgggcuuuug.............................................................    146    0      S10
............................cuacccuguagauccgggcuuuugu............................................................    23     0      S10
............................cuacccuguagauccgggcuuuuguagacugcuuagca...............................................    1      0      S10
............................cuacccuguagauccgggcuuuuguagacugcuuagcauuc............................................    5      0      S10
............................cuacccuguagauccgggcuuuuguagacugcuuagcauuca...........................................    12     0      S10
.............................uacccuguagauccgggcuuu...............................................................    1      0      S10
.............................uacccuguagauccgggcuuuu..............................................................    15     0      S10
.............................uacccuguagauccgggcuuuug.............................................................    82     0      S10
.............................uacccuguagauccgggcuuuugu............................................................    10     0      S10
.............................uacccuguagauccgggcuuuugua...........................................................    1      0      S10
.............................uacccuguagauccgggcuuuuguagac........................................................    1      0      S10
.............................uacccuguagauccgggcuuuuguagacu.......................................................    1      0      S10
.............................uacccuguagauccgggcuuuuguagacugc.....................................................    1      0      S10
.............................uacccuguagauccgggcuuuuguagacugcuuagca...............................................    2      0      S10
.............................uacccuguagauccgggcuuuuguagacugcuuagcauu.............................................    5      0      S10
.............................uacccuguagauccgggcuuuuguagacugcuuagcauuc............................................    3      0      S10
.............................uacccuguagauccgggcuuuuguagacugcuuagcauuca...........................................    25     0      S10
.................................................uuguagacugcuuagcauucagaa........................................    1      0      S10
....................................................uagacugcuuagcauuca...........................................    172    0      S10
..........................................................gcuuagcauucagaagcucgucucuacagguaucu....................    2      0      S10
...................................................................ucagaagcucgucucuacagg.........................    1      0      S10
......................................................................gaagcucgucucuacagg.........................    2      0      S10
......................................................................gaagcucgucucuacaggu........................    3      0      S10
......................................................................gaagcucgucucuacagguau......................    14     0      S10
......................................................................gaagcucgucucuacagguauc.....................    785    0      S10
......................................................................gaagcucgucucuacagguaucu....................    4394   0      S10
......................................................................gaagcucgucucuacagguaucuu...................    27     0      S10
.......................................................................aagcucgucucuacagguaucu....................    4      0      S10
........................................................................agcucgucucuacagguaucu....................    2      0      S10

............................cuacccuguagauccgggcuuuug.............................................................    1      0      S05
............................cuacccuguagauccgggcuuuugu............................................................    3      0      S05
.............................uacccuguagauccgggcuuuug.............................................................    1      0      S05
.............................uacccuguagauccgggcuuuugu............................................................    3      0      S05
....................................................uagacugcuuagcauuca...........................................    2      0      S05
......................................................................gaagcucgucucuacagg.........................    12     0      S05
......................................................................gaagcucgucucuacagguauc.....................    86     0      S05
......................................................................gaagcucgucucuacagguaucu....................    140    0      S05
......................................................................gaagcucgucucuacagguaucuu...................    5      0      S05

............................cuacccuguagauccgggcuuuug.............................................................    1      0      S04
............................cuacccuguagauccgggcuuuugu............................................................    1      0      S04
............................cuacccuguagauccgggcuuuuguagacugcuuagcauuc............................................    1      0      S04
.............................uacccuguagauccgggcuuuug.............................................................    2      0      S04
....................................................uagacugcuuagcauuca...........................................    10     0      S04
...................................................................ucagaagcucgucucuacagg.........................    1      0      S04
```

```
                            Star                                        Mature
ggauguuaucucucuagccguucguaaacuacccuguagauccgggcuuuuguagacugcuuagcauucagaagcucgucucuacagguaucuugcgaacgaugcugauugca
......................................................................gaagcucgucucuacagg.........................   6     0   S04
......................................................................gaagcucgucucuacaggu........................   1     0   S04
......................................................................gaagcucgucucuacagguau......................   2     0   S04
......................................................................gaagcucgucucuacagguauc.....................   82    0   S04
......................................................................gaagcucgucucuacagguaucu....................   165   0   S04
......................................................................gaagcucgucucuacagguaucuu...................   2     0   S04

............................cuacccuguagauccgggcuuuug.............................................................   1     0   S02
....................................................uagacugcuuagcauuca...........................................   2     0   S02
......................................................................gaagcucgucucuacagg.........................   9     0   S02
......................................................................gaagcucgucucuacaggu........................   1     0   S02
......................................................................gaagcucgucucuacagguauc.....................   24    0   S02
......................................................................gaagcucgucucuacagguaucu....................   66    0   S02

............................cuacccuguagauccgggcu.................................................................   1     0   S08
............................cuacccuguagauccgggcuuu...............................................................   1     0   S08
............................cuacccuguagauccgggcuuuu..............................................................   7     0   S08
............................cuacccuguagauccgggcuuuug.............................................................   59    0   S08
............................cuacccuguagauccgggcuuuugu............................................................   18    0   S08
............................cuacccuguagauccgggcuuuuguagacu.......................................................   1     0   S08
............................cuacccuguagauccgggcuuuuguagacugcuuagca...............................................   6     0   S08
............................cuacccuguagauccgggcuuuuguagacugcuuagcauuc............................................   24    0   S08
............................cuacccuguagauccgggcuuuuguagacugcuuagcauuca...........................................   23    0   S08
............................cuacccuguagauccgggcuuuuguagacugcuuagcauucag.........................................   2     0   S08
.............................uacccuguagauccgggcuuu...............................................................   2     0   S08
.............................uacccuguagauccgggcuuuu..............................................................   6     0   S08
.............................uacccuguagauccgggcuuuug.............................................................   16    0   S08
.............................uacccuguagauccgggcuuuugu............................................................   4     0   S08
.............................uacccuguagauccgggcuuuuguaga.........................................................   1     0   S08
.............................uacccuguagauccgggcuuuuguagac........................................................   1     0   S08
.............................uacccuguagauccgggcuuuuguagacugcuuagca...............................................   2     0   S08
.............................uacccuguagauccgggcuuuuguagacugcuuagcauuc............................................   3     0   S08
.............................uacccuguagauccgggcuuuuguagacugcuuagcauuca...........................................   25    0   S08
..............................acccuguagauccgggcuuuug.............................................................   2     0   S08
..............................acccuguagauccgggcuuuuguagacu.......................................................   1     0   S08
....................................................uagacugcuuagcauuca...........................................   30    0   S08
..........................................................gcuuagcauucagaagcucgucucuacagguaucuu...................   2     0   S08
...................................................................ucagaagcucgucucuacag..........................   1     0   S08
...................................................................ucagaagcucgucucuacaggua.......................   1     0   S08
.....................................................................agaagcucgucucuacagguauc.....................   1     0   S08
......................................................................gaagcucgucucuacagg.........................   2     0   S08
......................................................................gaagcucgucucuacagguau......................   6     0   S08
......................................................................gaagcucgucucuacagguauc.....................   2041  0   S08
......................................................................gaagcucgucucuacagguaucu....................   4191  0   S08
......................................................................gaagcucgucucuacagguaucuu...................   38    0   S08
.......................................................................aagcucgucucuacagguauc.....................   2     0   S08

............................cuacccuguagauccgggcuuu...............................................................   1     0   S03
....................................................uagacugcuuagcauuca...........................................   2     0   S03
...................................................................ucagaagcucgucucuacagg.........................   1     0   S03
......................................................................gaagcucgucucuacagg.........................   9     0   S03
......................................................................gaagcucgucucuacaggu........................   2     0   S03
......................................................................gaagcucgucucuacagguau......................   1     0   S03
......................................................................gaagcucgucucuacagguauc.....................   35    0   S03
......................................................................gaagcucgucucuacagguaucu....................   62    0   S03

............................cuacccuguagauccgggcuuu...............................................................   3     0   S09
............................cuacccuguagauccgggcuuuu..............................................................   16    0   S09
............................cuacccuguagauccgggcuuuug.............................................................   198   0   S09
............................cuacccuguagauccgggcuuuugu............................................................   22    0   S09
............................cuacccuguagauccgggcuuuuguagac........................................................   1     0   S09
............................cuacccuguagauccgggcuuuuguagacugcuuagca...............................................   1     0   S09
............................cuacccuguagauccgggcuuuuguagacugcuuagcauu.............................................   1     0   S09
............................cuacccuguagauccgggcuuuuguagacugcuuagcauuc............................................   3     0   S09
............................cuacccuguagauccgggcuuuuguagacugcuuagcauuca...........................................   17    0   S09
.............................uacccuguagauccgggcu.................................................................   1     0   S09
.............................uacccuguagauccgggcuuuu..............................................................   15    0   S09
.............................uacccuguagauccgggcuuuug.............................................................   111   0   S09
.............................uacccuguagauccgggcuuuugu............................................................   30    0   S09
.............................uacccuguagauccgggcuuuugua...........................................................   1     0   S09
```

Star Mature

```
ggauguuaucucucuagccguucguaaacuacccuguagauccgggcuuuuguagacugcuuagcauucagaagcucgucucuacagguaucuugcgaacgaugcugauugca
.............................uacccuguagauccgggcuuuuguag..........................................................	1	0	S09
.............................uacccuguagauccgggcuuuuguagac........................................................	2	0	S09
.............................uacccuguagauccgggcuuuuguagacugcuuagca...............................................	2	0	S09
.............................uacccuguagauccgggcuuuuguagacugcuuagcauu.............................................	1	0	S09
.............................uacccuguagauccgggcuuuuguagacugcuuagcauuc............................................	2	0	S09
.............................uacccuguagauccgggcuuuuguagacugcuuagcauuca...........................................	32	0	S09
..............................acccuguagauccgggcuuuug.............................................................	3	0	S09
....................................................uagacugcuuagcauuca...........................................	156	0	S09
.........................................................cugcuuagcauucagaagcucgucucuacagguaucu...................	1	0	S09
.....................................................................agaagcucgucucuacagguauc.....................	1	0	S09
......................................................................gaagcucgucucuacagg.........................	12	0	S09
......................................................................gaagcucgucucuacaggua.......................	2	0	S09
......................................................................gaagcucgucucuacagguau......................	13	0	S09
......................................................................gaagcucgucucuacagguauc.....................	758	0	S09
......................................................................gaagcucgucucuacagguaucu....................	3201	0	S09
......................................................................gaagcucgucucuacagguaucuu...................	22	0	S09
.......................................................................aagcucgucucuacagguaucu....................	1	0	S09
```

Provisional ID : Scaffold19_714
Score total : 586.7
Score for star read(s) : 3.9
Score for read counts : 579.3
Score for mfe : 2
Score for randfold : 1.6
Score for cons. seed :
Total read count : 1148
Mature read count : 863
Loop read count : 0
Star read count : 285

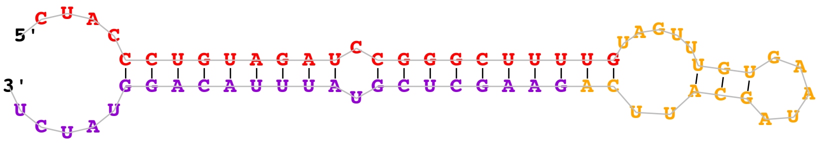

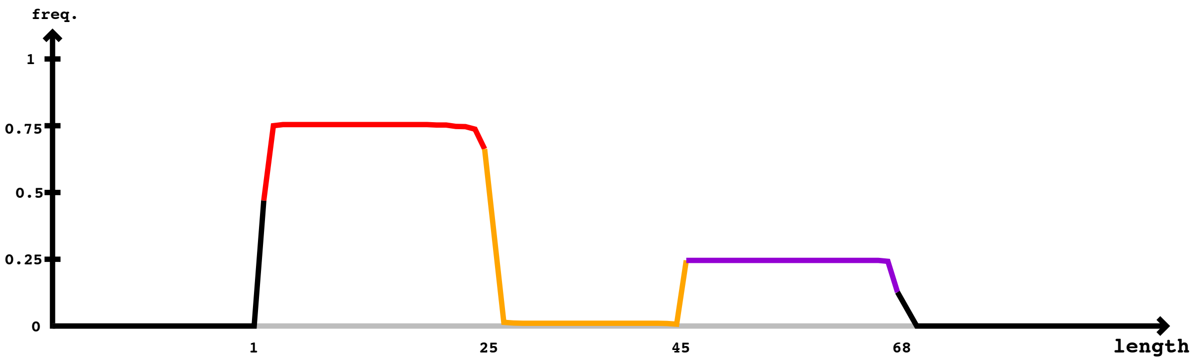

Mature **Star**

```
5'- ggcagguagccauucguaaccuacccuguagauccgggcuuuuguaguuugugaauagcauucagaagcucguauuuacagguaucuugcgaaucuggcugauugacaucauua  -3'   obs
    ggcagguagccauucguaaccuacccuguagauccgggcuuuuguaguuugugaauagcauucagaagcucguauuuacagguaucuugcgaaucuggcugauugacaucauua         exp
    ..(((.((((((((((((..((((.(((((((.((((((((((.....(((.....)))..))))))))))).)))))))))))..)))))))..)))))).)))..........   reads   mm   sample
    ....................cuacccuguagauccgggcuuuug......................................................................   1       0    S05
    ....................cuacccuguagauccgggcuuuugu.....................................................................   3       0    S05
    .....................uacccuguagauccgggcuuuug......................................................................   1       0    S05
    .....................uacccuguagauccgggcuuuugu.....................................................................   3       0    S05

    ....................cuacccuguagauccggg............................................................................   2       0    S10
    ....................cuacccuguagauccgggcu..........................................................................   4       0    S10
    ....................cuacccuguagauccgggcuu.........................................................................   1       0    S10
    ....................cuacccuguagauccgggcuuu........................................................................   1       0    S10
    ....................cuacccuguagauccgggcuuuu.......................................................................   23      0    S10
    ....................cuacccuguagauccgggcuuuug......................................................................   146     0    S10
    ....................cuacccuguagauccgggcuuuugu.....................................................................   23      0    S10
    .....................uacccuguagauccgggcuuu........................................................................   1       0    S10
    .....................uacccuguagauccgggcuuuu.......................................................................   15      0    S10
    .....................uacccuguagauccgggcuuuug......................................................................   82      0    S10
    .....................uacccuguagauccgggcuuuugu.....................................................................   10      0    S10
    .....................uacccuguagauccgggcuuuugua....................................................................   1       0    S10
    .....................uacccuguagauccgggcuuuuguaguuugugaauagcauuc...................................................   1       0    S10
    .....................uacccuguagauccgggcuuuuguaguuugugaauagcauuca..................................................   2       0    S10
    ................................................................gaagcucguauuuacagguauc............................   35      0    S10
    ................................................................gaagcucguauuuacagguaucu...........................   80      0    S10

    ....................cuacccuguagauccgggcuuuu.......................................................................   1       0    S06
    ....................cuacccuguagauccgggcuuuug......................................................................   3       0    S06
    ....................cuacccuguagauccgggcuuuugu.....................................................................   1       0    S06
    .....................uacccuguagauccgggcuuu........................................................................   1       0    S06
    .....................uacccuguagauccgggcuuuu.......................................................................   1       0    S06
    .....................uacccuguagauccgggcuuuug......................................................................   1       0    S06
    .....................uacccuguagauccgggcuuuugu.....................................................................   1       0    S06
    .....................uacccuguagauccgggcuuuugua....................................................................   1       0    S06

    ....................cuacccuguagauccgggcuuu........................................................................   1       0    S07
    ....................cuacccuguagauccgggcuuuu.......................................................................   1       0    S07
```

```
                    Mature                                      Star
ggcagguagccauucguaaccuacccuguagauccgggcuuuuguaguuugugaauagcauucagaagcucguauuuacagguaucuugcgaaucuggcugauugacaucauua
....................cuacccuguagauccgggcuuuug......................................................................    4    0    S07
....................cuacccuguagauccgggcuuuugu.....................................................................    1    0    S07
.....................uacccuguagauccgggcuuuu.......................................................................    1    0    S07
.....................uacccuguagauccgggcuuuug......................................................................    3    0    S07
.....................uacccuguagauccgggcuuuugu.....................................................................    2    0    S07
.....................uacccuguagauccgggcuuuuguaguuugugaauagcauuc...................................................    2    0    S07
.....................uacccuguagauccgggcuuuuguaguuugugaauagcauuca..................................................    1    0    S07
................................................................gaagcucguauuuacagguauc............................    2    0    S07
................................................................gaagcucguauuuacagguaucu...........................    6    0    S07
................................................................gaagcucguauuuacagguaucuu..........................    2    0    S07

....................cuacccuguagauccgggcuuu........................................................................    1    0    S03
................................................................gaagcucguauuuacagguauc............................    1    0    S03

....................cuacccuguagauccgggcuuu........................................................................    3    0    S09
....................cuacccuguagauccgggcuuuu.......................................................................    16   0    S09
....................cuacccuguagauccgggcuuuug......................................................................    198  0    S09
....................cuacccuguagauccgggcuuuugu.....................................................................    22   0    S09
.....................uacccuguagauccgggcu..........................................................................    1    0    S09
.....................uacccuguagauccgggcuuuu.......................................................................    15   0    S09
.....................uacccuguagauccgggcuuuug......................................................................    111  0    S09
.....................uacccuguagauccgggcuuuugu.....................................................................    30   0    S09
.....................uacccuguagauccgggcuuuugua....................................................................    1    0    S09
.....................uacccuguagauccgggcuuuuguag...................................................................    1    0    S09
.....................uacccuguagauccgggcuuuuguaguuugugaauagcauu....................................................    1    0    S09
.....................uacccuguagauccgggcuuuuguaguuugugaauagcauuca..................................................    5    0    S09
......................acccuguagauccgggcuuuug......................................................................    3    0    S09
................................................................gaagcucguauuuacagguau.............................    4    0    S09
................................................................gaagcucguauuuacagguauc............................    90   0    S09
................................................................gaagcucguauuuacagguaucu...........................    55   0    S09

....................cuacccuguagauccgggcu..........................................................................    1    0    S08
....................cuacccuguagauccgggcuuu........................................................................    1    0    S08
....................cuacccuguagauccgggcuuuu.......................................................................    7    0    S08
....................cuacccuguagauccgggcuuuug......................................................................    59   0    S08
....................cuacccuguagauccgggcuuuugu.....................................................................    18   0    S08
.....................uacccuguagauccgggcuuu........................................................................    2    0    S08
.....................uacccuguagauccgggcuuuu.......................................................................    6    0    S08
.....................uacccuguagauccgggcuuuug......................................................................    16   0    S08
.....................uacccuguagauccgggcuuuugu.....................................................................    4    0    S08
......................acccuguagauccgggcuuuug......................................................................    2    0    S08
................................................................gaagcucguauuuacagguauc............................    5    0    S08
................................................................gaagcucguauuuacagguaucu...........................    1    0    S08

....................cuacccuguagauccgggcuuuug......................................................................    1    0    S02
................................................................gaagcucguauuuacagguaucu...........................    2    0    S02

....................cuacccuguagauccgggcuuuug......................................................................    1    0    S04
....................cuacccuguagauccgggcuuuugu.....................................................................    1    0    S04
.....................uacccuguagauccgggcuuuug......................................................................    2    0    S04
................................................................gaagcucguauuuacagguauc............................    1    0    S04
................................................................gaagcucguauuuacagguaucu...........................    1    0    S04
```

Provisional ID	:	Scaffold13_568
Score total	:	1143.3
Score for star read(s)	:	3.9
Score for read counts	:	1136
Score for mfe	:	1.8
Score for randfold	:	1.6
Score for cons. seed	:	
Total read count	:	2240
Mature read count	:	2224
Loop read count	:	0
Star read count	:	16

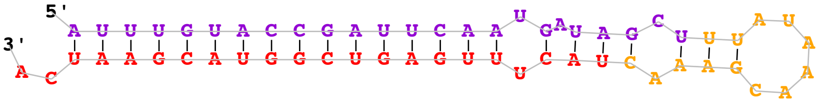

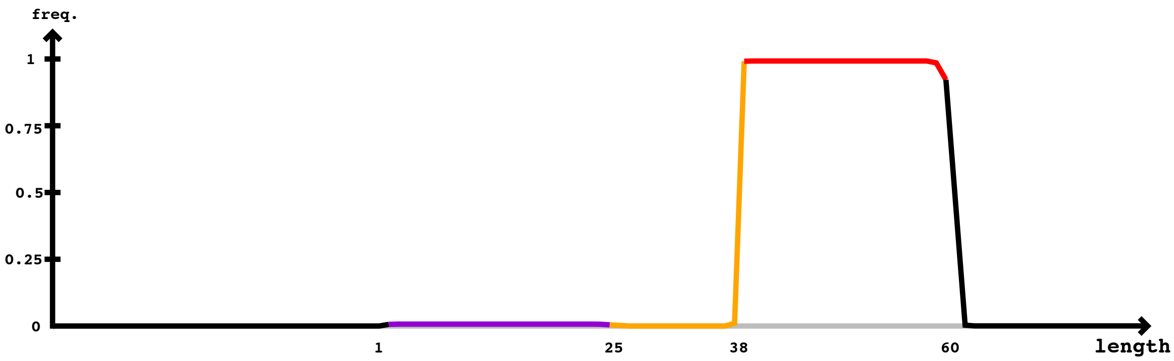


```
                                   Star                             Mature
5'- auaaccaagaaccagaauucuuggcuuaaagugauuuguaccgauucaaugauagcuuuauaaaacgaaacuacuuugagucgguacgaaucauuuuagucauggcugucacc  -3'   obs
    auaaccaagaaccagaauucuuggcuuaaagugauuuguaccgauucaaugauagcuuuauaaaacgaaacuacuuugagucgguacgaaucauuuuagucauggcugucacc        exp
    ....(((((((......))))))).((((((((((((((((((((((((.(.(((.(((......))).)))).)))))))))))))))))))))))))...............   reads   mm   sample
    .................................auuuguaccgauucaaugauag..........................................................   1       0    S10
    .................................auuuguaccgauucaaugauagcu........................................................   1       0    S10
    .......................................................................uacuuugagucgguacgaau......................   2       0    S10
    .......................................................................uacuuugagucgguacgaauc.....................   7       0    S10
    .......................................................................uacuuugagucgguacgaauca....................   135     0    S10
    .......................................................................uacuuugagucgguacgaaucau...................   1       0    S10
    .......................................................................uacuuugagucgguacgaaucauu..................   1       0    S10
    ........................................................................acuuugagucgguacgaauca....................   1       0    S10

    .................................auuuguaccgauucaaugauagcu........................................................   1       0    S05
    ......................................................................cuacuuugagucgguacgaauca....................   3       0    S05
    .......................................................................uacuuugagucgguacgaau......................   2       0    S05
    .......................................................................uacuuugagucgguacgaauc.....................   18      0    S05
    .......................................................................uacuuugagucgguacgaauca....................   201     0    S05
    .......................................................................uacuuugagucgguacgaaucau...................   5       0    S05
    .......................................................................uacuuugagucgguacgaaucauu..................   2       0    S05

    .................................auuuguaccgauucaaugauagc.........................................................   2       0    S07
    ......................................................................cuacuuugagucgguacgaauc.....................   1       0    S07
    ......................................................................cuacuuugagucgguacgaauca....................   4       0    S07
    .......................................................................uacuuugagucgguacgaauc.....................   15      0    S07
    .......................................................................uacuuugagucgguacgaauca....................   194     0    S07
    .......................................................................uacuuugagucgguacgaaucau...................   2       0    S07
    .............................................................................................uuuuagucauggcugucacc   1       0    S07

    ......................................................................cuacuuugagucgguacgaauca....................   3       0    S06
    .......................................................................uacuuugagucgguacgaauc.....................   17      0    S06
    .......................................................................uacuuugagucgguacgaauca....................   241     0    S06
    .......................................................................uacuuugagucgguacgaaucau...................   7       0    S06

    .................................auuuguaccgauucaaugauagc.........................................................   2       0    S01
    .................................auuuguaccgauucaaugauagcu........................................................   3       0    S01
    ......................................................................cuacuuugagucgguacgaauc.....................   1       0    S01
```

Star Mature

```
auaaccaagaaccagaauucuuggcuuaaagugauuuguaccgauucaaugauagcuuuauaaacgaaacuacuuugagucgguacgaaucauuuuagucauggcugucacc
......................................................................uacuuugagucgguacgaau......................	5	0	S01
......................................................................uacuuugagucgguacgaauc.....................	24	0	S01
......................................................................uacuuugagucgguacgaauca....................	294	0	S01
......................................................................uacuuugagucgguacgaaucau...................	1	0	S01

.................................auuuguaccgauucaaugauagcu.......................................................	1	0	S08
.....................................................................cuacuuugagucgguacgaauca....................	2	0	S08
......................................................................uacuuugagucgguacgaauc.....................	10	0	S08
......................................................................uacuuugagucgguacgaauca....................	74	0	S08
......................................................................uacuuugagucgguacgaaucau...................	2	0	S08
......................................................................uacuuugagucgguacgaaucauu..................	1	0	S08

.....................................................................cuacuuugagucgguacgaauca....................	2	0	S03
......................................................................uacuuugagucgguacgaau......................	3	0	S03
......................................................................uacuuugagucgguacgaauc.....................	6	0	S03
......................................................................uacuuugagucgguacgaauca....................	172	0	S03
......................................................................uacuuugagucgguacgaaucau...................	1	0	S03

.................................auuuguaccgauucaaugauagcu.......................................................	2	0	S09
......................................................................uacuuugagucgguacgaau......................	1	0	S09
......................................................................uacuuugagucgguacgaauc.....................	6	0	S09
......................................................................uacuuugagucgguacgaauca....................	91	0	S09
......................................................................uacuuugagucgguacgaaucau...................	1	0	S09
......................................................................uacuuugagucgguacgaaucauu..................	1	0	S09
.......................................................................acuuugagucgguacgaauca....................	1	0	S09

..................................uuuguaccgauucaaugauagcu.......................................................	1	0	S02
.....................................................................cuacuuugagucgguacgaauc.....................	1	0	S02
.....................................................................cuacuuugagucgguacgaauca....................	3	0	S02
......................................................................uacuuugagucgguacgaau......................	2	0	S02
......................................................................uacuuugagucgguacgaauc.....................	6	0	S02
......................................................................uacuuugagucgguacgaauca....................	242	0	S02
......................................................................uacuuugagucgguacgaaucau...................	2	0	S02

.................................auuuguaccgauucaaugauagc........................................................	1	0	S04
..................................uuuguaccgauucaaugauagcu.......................................................	1	0	S04
.....................................................................cuacuuugagucgguacgaauc.....................	2	0	S04
.....................................................................cuacuuugagucgguacgaauca....................	1	0	S04
......................................................................uacuuugagucgguacgaau......................	1	0	S04
......................................................................uacuuugagucgguacgaauc.....................	27	0	S04
......................................................................uacuuugagucgguacgaauca....................	368	0	S04
......................................................................uacuuugagucgguacgaaucau...................	7	0	S04
......................................................................uacuuugagucgguacgaaucauu..................	1	0	S04
```

Provisional ID	:	Scaffold2369_34880
Score total	:	2864.7
Score for star read(s)	:	3.9
Score for read counts	:	2857.2
Score for mfe	:	2
Score for randfold	:	1.6
Score for cons. seed	:	
Total read count	:	5616
Mature read count	:	5282
Loop read count	:	0
Star read count	:	334

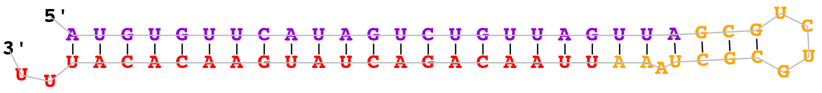

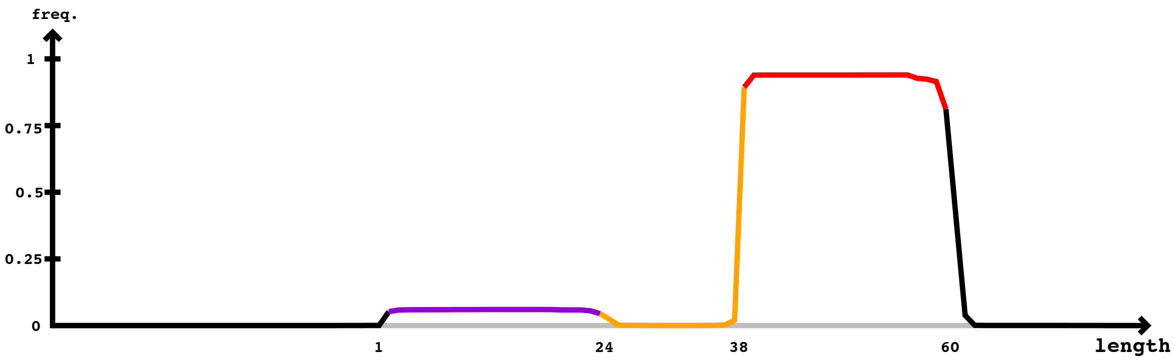

Star **Mature**

```
5'- uucaauaaaaaccuuaauuuucuuucaauccguauguguucauagucuguuaguuagcgucugcgcuaaauuaacagacuaugaacacauuuuauauugagacuaggagauc  -3'   obs
    uucaauaaaaaccuuaauuuucuuucaauccguauguguucauagucuguuaguuagcgucugcgcuaaauuaacagacuaugaacacauuuuauauugagacuaggagauc         exp
    ................(((((((((((((....((((((((((((((((((((((((((....)))).)))))))))))))))))))))).....))))))...))))))).    reads   mm    sample
    .................................auguguucauagucuguuaguu.........................................................    4       0     S03
    .................................auguguucauagucuguuaguua........................................................    13      0     S03
    ..................................uguguucauagucuguuaguua........................................................    2       0     S03
    ...........................................agucuguuaguuagcgucugcg...............................................    1       0     S03
    .....................................................................auuaacagacuaugaacacauu.....................    3       0     S03
    .....................................................................auuaacagacuaugaacacauuu....................    1       0     S03
    ......................................................................uuaacagacuaugaacaca.......................    1       0     S03
    ......................................................................uuaacagacuaugaacacauu.....................    12      0     S03
    ......................................................................uuaacagacuaugaacacauuu....................    62      0     S03
    ......................................................................uuaacagacuaugaacacauuuu...................    34      0     S03
    ......................................................................uuaacagacuaugaacacauuuua..................    7       0     S03
    .......................................................................uaacagacuaugaacacauuu....................    1       0     S03
    .......................................................................uaacagacuaugaacacauuuu...................    2       0     S03
    .......................................................................uaacagacuaugaacacauuuua..................    1       0     S03

    .................................auguguucauagucuguuagu..........................................................    2       0     S09
    .................................auguguucauagucuguuaguu.........................................................    12      0     S09
    .................................auguguucauagucuguuaguua........................................................    4       0     S09
    ..................................uguguucauagucuguuaguua........................................................    2       0     S09
    ....................................................................aauuaacagacuaugaacacau......................    1       0     S09
    .....................................................................auuaacagacuaugaacacau......................    2       0     S09
    .....................................................................auuaacagacuaugaacacauu.....................    5       0     S09
    ......................................................................uuaacagacuaugaacac........................    8       0     S09
    ......................................................................uuaacagacuaugaacaca.......................    3       0     S09
    ......................................................................uuaacagacuaugaacacau......................    8       0     S09
    ......................................................................uuaacagacuaugaacacauu.....................    98      0     S09
    ......................................................................uuaacagacuaugaacacauuu....................    524     0     S09
    ......................................................................uuaacagacuaugaacacauuuu...................    214     0     S09
    ......................................................................uuaacagacuaugaacacauuuua..................    27      0     S09
    .......................................................................uaacagacuaugaacacauuu....................    1       0     S09
    .......................................................................uaacagacuaugaacacauuuu...................    24      0     S09
    .......................................................................uaacagacuaugaacacauuuua..................    1       0     S09
    ..................................................................................gaacacauuuuauauugagacu........    1       0     S09
```

Star **Mature**

uucaauaaaaaccuuaauuuucuuucaauccguauguguucauagucuguuaguuagcgucugcgcuaaauuaacagacuaugaacacauuuuauauugagacuaggagauc

....uuaacagacuaugaacac........................	2	0	S08
....uuaacagacuaugaacacauu.....................	6	0	S08
....uuaacagacuaugaacacauuu....................	32	0	S08
....uuaacagacuaugaacacauuuu...................	5	0	S08
....uuaacagacuaugaacacauuuua..................	1	0	S08
....uaacagacuaugaacacauuuu...................	1	0	S08
............................uccguauguguucauagucugu..	1	0	S02
.................................auguguucauagucuguu...	1	0	S02
.................................auguguucauagucuguuagu..	15	0	S02
.................................auguguucauagucuguuaguu...	25	0	S02
.................................auguguucauagucuguuaguua..	119	0	S02
.................................auguguucauagucuguuaguuagc..	1	0	S02
..................................uguguucauagucuguuagu..	1	0	S02
..................................uguguucauagucuguuaguua..	22	0	S02
...................................guguucauagucuguuaguua..	3	0	S02
....cuaaauuaacagacuaugaacac........................	1	0	S02
....aaauuaacagacuaugaacacauu.....................	1	0	S02
....aauuaacagacuaugaacacauu.....................	7	0	S02
....aauuaacagacuaugaacacauuu....................	1	0	S02
....auuaacagacuaugaacaca.......................	1	0	S02
....auuaacagacuaugaacacau......................	4	0	S02
....auuaacagacuaugaacacauu.....................	48	0	S02
....auuaacagacuaugaacacauuu....................	2	0	S02
....uuaacagacuaugaacac........................	39	0	S02
....uuaacagacuaugaacaca.......................	5	0	S02
....uuaacagacuaugaacacau......................	15	0	S02
....uuaacagacuaugaacacauu.....................	266	0	S02
....uuaacagacuaugaacacauuu....................	1418	0	S02
....uuaacagacuaugaacacauuuu...................	820	0	S02
....uuaacagacuaugaacacauuuua..................	105	0	S02
....uuaacagacuaugaacacauuuuau.................	2	0	S02
....uuaacagacuaugaacacauuuuaua................	1	0	S02
....uaacagacuaugaacacauu.....................	1	0	S02
....uaacagacuaugaacacauuu....................	11	0	S02
....uaacagacuaugaacacauuuu...................	125	0	S02
....uaacagacuaugaacacauuuua..................	10	0	S02
....aacagacuaugaacacauuu....................	1	0	S02
....acagacuaugaacacauu.....................	1	0	S02
....acauuuuauauugagacuagga....	1	0	S02
.................................auguguucauagucuguu...	2	0	S04
.................................auguguucauagucuguuaguu...	4	0	S04
.................................auguguucauagucuguuaguua..	21	0	S04
...................................guguucauagucuguuaguua..	1	0	S04
....auuaacagacuaugaacacau......................	2	0	S04
....auuaacagacuaugaacacauu.....................	11	0	S04
....auuaacagacuaugaacacauuu....................	1	0	S04
....uuaacagacuaugaacac........................	1	0	S04
....uuaacagacuaugaacaca.......................	3	0	S04
....uuaacagacuaugaacacau......................	2	0	S04
....uuaacagacuaugaacacauu.....................	25	0	S04
....uuaacagacuaugaacacauuu....................	122	0	S04
....uuaacagacuaugaacacauuuu...................	48	0	S04
....uuaacagacuaugaacacauuuua..................	9	0	S04
....uaacagacuaugaacacauuu....................	1	0	S04
....uaacagacuaugaacacauuuu...................	7	0	S04
....uaacagacuaugaacacauuuua..................	2	0	S04
...............................guauguguucauagucuguua..	1	0	S05
...............................guauguguucauagucuguuaguua..	1	0	S05
.................................auguguucauagucuguu...	2	0	S05
.................................auguguucauagucuguuaguu...	2	0	S05
.................................auguguucauagucuguuaguua..	18	0	S05
..................................uguguucauagucuguuaguua..	3	0	S05
...................................guguucauagucuguuaguua..	1	0	S05
....................................uguucauagucuguuaguuagc..	1	0	S05
....cauagucuguuaguuagcgucu..	1	0	S05
....auuaacagacuaugaacacau......................	2	0	S05

Star **Mature**

```
uucaauaaaaaccuuaauuuucuuucaauccguauguguucauagucuguuaguuagcgucugcgcuaaauuaacagacuaugaacacauuuuauauugagacuaggagauc
.....................................................................auuaacagacuaugaacacauu.....................  5    0  S05
.....................................................................auuaacagacuaugaacacauuu....................  1    0  S05
......................................................................uuaacagacuaugaacac........................  8    0  S05
......................................................................uuaacagacuaugaacaca.......................  2    0  S05
......................................................................uuaacagacuaugaacacau......................  2    0  S05
......................................................................uuaacagacuaugaacacauu.....................  17   0  S05
......................................................................uuaacagacuaugaacacauuu....................  111  0  S05
......................................................................uuaacagacuaugaacacauuuu...................  55   0  S05
......................................................................uuaacagacuaugaacacauuuua..................  5    0  S05
.......................................................................uaacagacuaugaacacauuu....................  3    0  S05
.......................................................................uaacagacuaugaacacauuuu...................  12   0  S05
.......................................................................uaacagacuaugaacacauuuua..................  1    0  S05

.................................auguguucauagucuguu.............................................................  1    0  S10
.................................auguguucauagucuguuaguu.........................................................  7    0  S10
.................................auguguucauagucuguuaguua........................................................  2    0  S10
..................................uguguucauagucuguuaguua........................................................  1    0  S10
..................................................................uaaauuaacagacuaugaacac........................  1    0  S10
.....................................................................auuaacagacuaugaacacau......................  1    0  S10
.....................................................................auuaacagacuaugaacacauu.....................  3    0  S10
.....................................................................auuaacagacuaugaacacauuu....................  1    0  S10
......................................................................uuaacagacuaugaacac........................  3    0  S10
......................................................................uuaacagacuaugaacacauu.....................  16   0  S10
......................................................................uuaacagacuaugaacacauuu....................  122  0  S10
......................................................................uuaacagacuaugaacacauuuu...................  33   0  S10
.......................................................................uaacagacuaugaacacauuuu...................  4    0  S10

.................................auguguucauagucuguuagu..........................................................  1    0  S06
.................................auguguucauagucuguuaguu.........................................................  5    0  S06
.................................auguguucauagucuguuaguua........................................................  25   0  S06
.................................auguguucauagucuguuaguuagcgu....................................................  1    0  S06
..................................uguguucauagucuguuaguua........................................................  3    0  S06
...................................guguucauagucuguuaguua........................................................  1    0  S06
........................................cauagucuguuaguuagcgucu..................................................  1    0  S06
.....................................................................auuaacagacuaugaacacau......................  2    0  S06
.....................................................................auuaacagacuaugaacacauu.....................  3    0  S06
......................................................................uuaacagacuaugaacac........................  4    0  S06
......................................................................uuaacagacuaugaacaca.......................  3    0  S06
......................................................................uuaacagacuaugaacacau......................  10   0  S06
......................................................................uuaacagacuaugaacacauu.....................  43   0  S06
......................................................................uuaacagacuaugaacacauuu....................  247  0  S06
......................................................................uuaacagacuaugaacacauuuu...................  171  0  S06
......................................................................uuaacagacuaugaacacauuuua..................  30   0  S06
.......................................................................uaacagacuaugaacacauuuu...................  36   0  S06
.......................................................................uaacagacuaugaacacauuuua..................  4    0  S06

.................................auguguucauagucuguuaguua........................................................  1    0  S01
......................................uucauagucuguuaguuagcg.....................................................  1    0  S01
.................................................................cuaaauuaacagacuaugaacac........................  1    0  S01
...................................................................aaauuaacagacuaugaacac........................  1    0  S01
......................................................................uuaacagacuaugaacac........................  1    0  S01
......................................................................uuaacagacuaugaacacauu.....................  6    0  S01
......................................................................uuaacagacuaugaacacauuu....................  28   0  S01
......................................................................uuaacagacuaugaacacauuuu...................  28   0  S01
......................................................................uuaacagacuaugaacacauuuua..................  3    0  S01
.......................................................................uaacagacuaugaacacauuu....................  1    0  S01
.......................................................................uaacagacuaugaacacauuuu...................  3    0  S01
.......................................................................uaacagacuaugaacacauuuua..................  4    0  S01

.................................auguguucauagucuguuaguu.........................................................  1    0  S07
.................................auguguucauagucuguuaguua........................................................  1    0  S07
.....................................................................auuaacagacuaugaacacauu.....................  1    0  S07
......................................................................uuaacagacuaugaacac........................  1    0  S07
......................................................................uuaacagacuaugaacacauu.....................  5    0  S07
......................................................................uuaacagacuaugaacacauuu....................  22   0  S07
......................................................................uuaacagacuaugaacacauuuu...................  13   0  S07
......................................................................uuaacagacuaugaacacauuuua..................  1    0  S07
.......................................................................uaacagacuaugaacacauuuu...................  1    0  S07
```

Provisional ID	:	Scaffold1034_21346
Score total	:	2864.6
Score for star read(s)	:	3.9
Score for read counts	:	2857.2
Score for mfe	:	1.9
Score for randfold	:	1.6
Score for cons. seed	:	
Total read count	:	5616
Mature read count	:	5282
Loop read count	:	0
Star read count	:	334

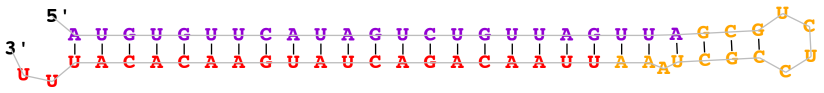

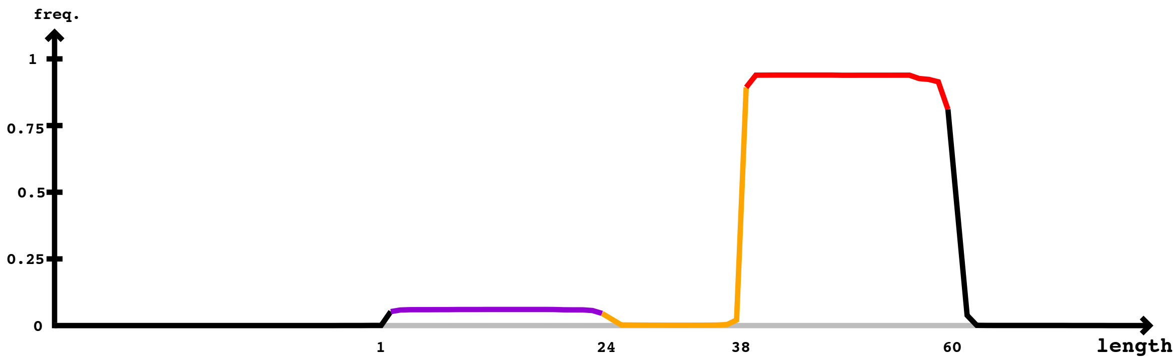

Star **Mature**

5'- uucaauaaaaaccuuuauuuucuuucaauccguauguguucauagucuguuaguuagcgucuccgcuaaauuaacagacuaugaacacauuuuauauugagacuaggagauc -3'	obs			
uucaauaaaaaccuuuauuuucuuucaauccguauguguucauagucuguuaguuagcgucuccgcuaaauuaacagacuaugaacacauuuuauauugagacuaggagauc	exp			
................((((((((((((....(((((((((((((((((((((((((((....)))).))))))))))))))))))))))).....))))))...))))))).		reads	mm	sample
.................................auguguucauagucuguu...		1	0	S10
.................................auguguucauagucuguuaguu...		7	0	S10
.................................auguguucauagucuguuaguua..		2	0	S10
..................................uguguucauagucuguuaguua..		1	0	S10
....uaaauuaacagacuaugaacac........................		1	0	S10
....auuaacagacuaugaacacau......................		1	0	S10
....auuaacagacuaugaacacauu.....................		3	0	S10
....auuaacagacuaugaacacauuu....................		1	0	S10
....uuaacagacuaugaacac........................		3	0	S10
....uuaacagacuaugaacacauu.....................		16	0	S10
....uuaacagacuaugaacacauuu....................		122	0	S10
....uuaacagacuaugaacacauuuu...................		33	0	S10
....uaacagacuaugaacacauuuu...................		4	0	S10
...............................guauguguucauagucuguua..		1	0	S05
...............................guauguguucauagucuguuaguua..		1	0	S05
.................................auguguucauagucuguu...		2	0	S05
.................................auguguucauagucuguuaguu...		2	0	S05
.................................auguguucauagucuguuaguua..		18	0	S05
..................................uguguucauagucuguuaguua..		3	0	S05
...................................guguucauagucuguuaguua..		1	0	S05
....................................uguucauagucuguuaguuagc..		1	0	S05
....cauagucuguuaguuagcgucu..		1	0	S05
....auuaacagacuaugaacacau......................		2	0	S05
....auuaacagacuaugaacacauu.....................		5	0	S05
....auuaacagacuaugaacacauuu....................		1	0	S05
....uuaacagacuaugaacac........................		8	0	S05
....uuaacagacuaugaacaca.......................		2	0	S05
....uuaacagacuaugaacacau......................		2	0	S05
....uuaacagacuaugaacacauu.....................		17	0	S05
....uuaacagacuaugaacacauuu....................		111	0	S05
....uuaacagacuaugaacacauuuu...................		55	0	S05
....uuaacagacuaugaacacauuuua..................		5	0	S05

Star Mature

uucaauaaaaaccuuuauuuucuuucaauccguauguguucauagucuguuaguuagcgucuccgcuaaauuaacagacuaugaacacauuuuauauugagacuaggagauc			
....uaacagacuaugaacacauuu....................	3	0	S05
....uaacagacuaugaacacauuuu...................	12	0	S05
....uaacagacuaugaacacauuuua..................	1	0	S05
.................................auguguucauagucuguuaguu...	1	0	S07
.................................auguguucauagucuguuaguua..	1	0	S07
.................................auguguucauagucuguuaguuagcgucuccgcuaaa..	1	0	S07
....auuaacagacuaugaacacauu.....................	1	0	S07
....uuaacagacuaugaacac........................	1	0	S07
....uuaacagacuaugaacacauu.....................	5	0	S07
....uuaacagacuaugaacacauuu....................	22	0	S07
....uuaacagacuaugaacacauuuu...................	13	0	S07
....uuaacagacuaugaacacauuuua..................	1	0	S07
....uaacagacuaugaacacauuuu...................	1	0	S07
.................................auguguucauagucuguuagu..	1	0	S06
.................................auguguucauagucuguuaguu...	5	0	S06
.................................auguguucauagucuguuaguua..	25	0	S06
.................................auguguucauagucuguuaguuagcgu..	1	0	S06
..................................uguguucauagucuguuaguua..	3	0	S06
...................................guguucauagucuguuaguua..	1	0	S06
....cauagucuguuaguuagcgucu..	1	0	S06
....agucuguuaguuagcgucuccg...	1	0	S06
....auuaacagacuaugaacacau......................	2	0	S06
....auuaacagacuaugaacacauu.....................	3	0	S06
....uuaacagacuaugaacac........................	4	0	S06
....uuaacagacuaugaacaca.......................	3	0	S06
....uuaacagacuaugaacacau......................	10	0	S06
....uuaacagacuaugaacacauu.....................	43	0	S06
....uuaacagacuaugaacacauuu....................	247	0	S06
....uuaacagacuaugaacacauuuu...................	171	0	S06
....uuaacagacuaugaacacauuuua..................	30	0	S06
....uaacagacuaugaacacauuuu...................	36	0	S06
....uaacagacuaugaacacauuuua..................	4	0	S06
.................................auguguucauagucuguuaguua..	1	0	S01
......................................uucauagucuguuaguuagcg...	1	0	S01
....cuaaauuaacagacuaugaacac........................	1	0	S01
....aaauuaacagacuaugaacac........................	1	0	S01
....uuaacagacuaugaacac........................	1	0	S01
....uuaacagacuaugaacacauu.....................	6	0	S01
....uuaacagacuaugaacacauuu....................	28	0	S01
....uuaacagacuaugaacacauuuu...................	28	0	S01
....uuaacagacuaugaacacauuuua..................	3	0	S01
....uaacagacuaugaacacauu.....................	1	0	S01
....uaacagacuaugaacacauuu....................	3	0	S01
....uaacagacuaugaacacauuuua..................	4	0	S01
....uuaacagacuaugaacac........................	2	0	S08
....uuaacagacuaugaacacauu.....................	6	0	S08
....uuaacagacuaugaacacauuu....................	32	0	S08
....uuaacagacuaugaacacauuuu...................	5	0	S08
....uuaacagacuaugaacacauuuua..................	1	0	S08
....uaacagacuaugaacacauuuu...................	1	0	S08
.................................auguguucauagucuguuagu..	2	0	S09
.................................auguguucauagucuguuaguu...	12	0	S09
.................................auguguucauagucuguuaguua..	4	0	S09
..................................uguguucauagucuguuaguua..	2	0	S09
....aauuaacagacuaugaacacau......................	1	0	S09
....auuaacagacuaugaacacau......................	2	0	S09
....auuaacagacuaugaacacauu.....................	5	0	S09
....uuaacagacuaugaacac........................	8	0	S09
....uuaacagacuaugaacaca.......................	3	0	S09
....uuaacagacuaugaacacau......................	8	0	S09
....uuaacagacuaugaacacauu.....................	98	0	S09
....uuaacagacuaugaacacauuu....................	524	0	S09
....uuaacagacuaugaacacauuuu...................	214	0	S09
....uuaacagacuaugaacacauuuua..................	27	0	S09
....uaacagacuaugaacacauuu....................	1	0	S09

```
                                  Star                                  Mature
uucaauaaaaaccuuuauuuucuuucaauccguauguguucauagucuguuaguuagcgucuccgcuaaauuaacagacuaugaacacauuuuauauugagacuaggagauc
.......................................................................uaacagacuaugaacacauuuu...................   24     0   S09
.......................................................................uaacagacuaugaacacauuuua..................   1      0   S09
..................................................................................gaacacauuuuauauugagacu........   1      0   S09

.................................auguguucauagucuguuaguu.........................................................   4      0   S03
.................................auguguucauagucuguuaguua........................................................   13     0   S03
..................................uguguucauagucuguuaguua........................................................   2      0   S03
.....................................................................auuaacagacuaugaacacauu.....................   3      0   S03
.....................................................................auuaacagacuaugaacacauuu....................   1      0   S03
......................................................................uuaacagacuaugaacaca.......................   1      0   S03
......................................................................uuaacagacuaugaacacauu.....................   12     0   S03
......................................................................uuaacagacuaugaacacauuu....................   62     0   S03
......................................................................uuaacagacuaugaacacauuuu...................   34     0   S03
......................................................................uuaacagacuaugaacacauuuua..................   7      0   S03
.......................................................................uaacagacuaugaacacauuu....................   1      0   S03
.......................................................................uaacagacuaugaacacauuuu...................   2      0   S03
.......................................................................uaacagacuaugaacacauuuua..................   1      0   S03

.................................auguguucauagucuguu.............................................................   2      0   S04
.................................auguguucauagucuguuaguu.........................................................   4      0   S04
.................................auguguucauagucuguuaguua........................................................   21     0   S04
...................................guguucauagucuguuaguua........................................................   1      0   S04
.....................................................................auuaacagacuaugaacacau......................   2      0   S04
.....................................................................auuaacagacuaugaacacauu.....................   11     0   S04
.....................................................................auuaacagacuaugaacacauuu....................   1      0   S04
......................................................................uuaacagacuaugaacac........................   1      0   S04
......................................................................uuaacagacuaugaacaca.......................   3      0   S04
......................................................................uuaacagacuaugaacacau......................   2      0   S04
......................................................................uuaacagacuaugaacacauu.....................   25     0   S04
......................................................................uuaacagacuaugaacacauuu....................   122    0   S04
......................................................................uuaacagacuaugaacacauuuu...................   48     0   S04
......................................................................uuaacagacuaugaacacauuuua..................   9      0   S04
.......................................................................uaacagacuaugaacacauuu....................   1      0   S04
.......................................................................uaacagacuaugaacacauuuu...................   7      0   S04
.......................................................................uaacagacuaugaacacauuuua..................   2      0   S04

............................uccguauguguucauagucugu..............................................................   1      0   S02
.................................auguguucauagucuguu.............................................................   1      0   S02
.................................auguguucauagucuguuagu..........................................................   15     0   S02
.................................auguguucauagucuguuaguu.........................................................   25     0   S02
.................................auguguucauagucuguuaguua........................................................   119    0   S02
.................................auguguucauagucuguuaguuagc......................................................   1      0   S02
.................................auguguucauagucuguuaguuagcgucuccgcuaa...........................................   1      0   S02
..................................uguguucauagucuguuagu..........................................................   1      0   S02
..................................uguguucauagucuguuaguua........................................................   22     0   S02
...................................guguucauagucuguuaguua........................................................   3      0   S02
......................................................uagcgucuccgcuaaauuaacagacu................................   4      0   S02
.................................................................cuaaauuaacagacuaugaacac........................   1      0   S02
...................................................................aaauuaacagacuaugaacacauu.....................   1      0   S02
....................................................................aauuaacagacuaugaacacauu.....................   7      0   S02
....................................................................aauuaacagacuaugaacacauuu....................   1      0   S02
.....................................................................auuaacagacuaugaacaca.......................   1      0   S02
.....................................................................auuaacagacuaugaacacau......................   4      0   S02
.....................................................................auuaacagacuaugaacacauu.....................   48     0   S02
.....................................................................auuaacagacuaugaacacauuu....................   2      0   S02
......................................................................uuaacagacuaugaacac........................   39     0   S02
......................................................................uuaacagacuaugaacaca.......................   5      0   S02
......................................................................uuaacagacuaugaacacau......................   15     0   S02
......................................................................uuaacagacuaugaacacauu.....................   266    0   S02
......................................................................uuaacagacuaugaacacauuu....................   1418   0   S02
......................................................................uuaacagacuaugaacacauuuu...................   820    0   S02
......................................................................uuaacagacuaugaacacauuuua..................   105    0   S02
......................................................................uuaacagacuaugaacacauuuuau.................   2      0   S02
......................................................................uuaacagacuaugaacacauuuuaua................   1      0   S02
.......................................................................uaacagacuaugaacacauu.....................   1      0   S02
.......................................................................uaacagacuaugaacacauuu....................   11     0   S02
.......................................................................uaacagacuaugaacacauuuu...................   125    0   S02
.......................................................................uaacagacuaugaacacauuuua..................   10     0   S02
........................................................................aacagacuaugaacacauuu....................   1      0   S02
.........................................................................acagacuaugaacacauu.....................   1      0   S02
```

Star Mature

```
uucaauaaaaaccuuuauuuucuuucaauccguauguguucauagucuguuaguuagcgucuccgcuaaauuaacagacuaugaacacauuuuauauugagacuaggagauc
......................................................................................acauuuuauauugagacuagga....    1    0    S02
```

Provisional ID	:	Scaffold705_16398
Score total	:	2.1
Score for star read(s)	:	-1.3
Score for read counts	:	0
Score for mfe	:	1.8
Score for randfold	:	1.6
Score for cons. seed	:	
Total read count	:	138
Mature read count	:	135
Loop read count	:	0
Star read count	:	3

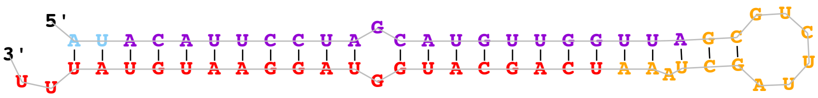

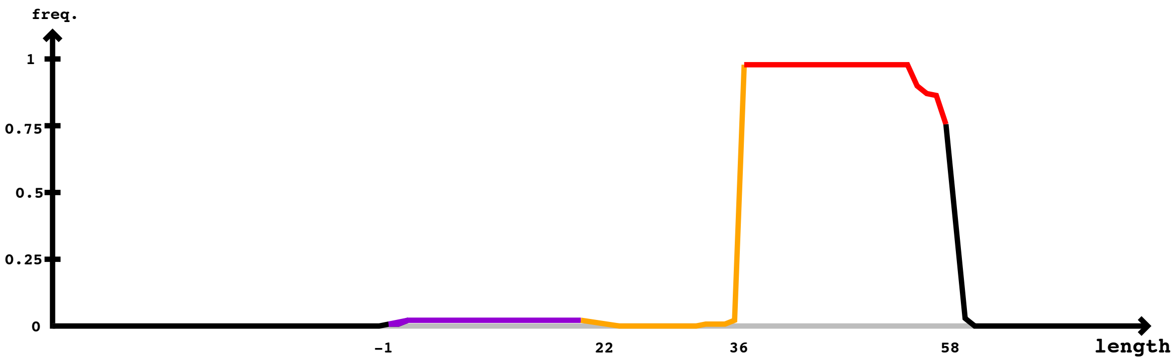

Star **Mature**

```
5'- aagaguaaaacuuuauauuuuccguugcuuuaaauacauuccuagcauguugguuagcgucuuagcuaaaucagcaugguaggaauguauuuuaaugucaagagcaugagau  -3'   obs
    aagaguaaaacuuuauauuuuccguugcuuuaaauacauuccuagcauguugguuagcgucuuagcuaaaucagcaugguaggaauguauuuuaaugucaagagcaugagau        exp
    .((((.....))))....((((...(((((((((((((((((((.(((((((((((((......))).)))))))))).)))))))))))))........)))))).)))).  reads   mm   sample
    .................................auacauuccuagcauguugguu.........................................................  1       0    S02
    ......................................................................ucagcaugguaggaaugu........................  1       0    S02
    ......................................................................ucagcaugguaggaaugua.......................  1       0    S02
    ......................................................................ucagcaugguaggaauguau......................  1       0    S02
    ......................................................................ucagcaugguaggaauguauu.....................  3       0    S02
    ......................................................................ucagcaugguaggaauguauuu....................  17      0    S02
    ......................................................................ucagcaugguaggaauguauuuu...................  5       0    S02
    ......................................................................ucagcaugguaggaauguauuuua..................  1       0    S02

    ......................................................................ucagcaugguaggaaugu........................  2       0    S04
    ......................................................................ucagcaugguaggaauguauu.....................  4       0    S04
    ......................................................................ucagcaugguaggaauguauuu....................  8       0    S04
    ......................................................................ucagcaugguaggaauguauuuu...................  7       0    S04
    ......................................................................ucagcaugguaggaauguauuuua..................  1       0    S04

    .....................................................................aucagcaugguaggaauguauu.....................  1       0    S03
    ......................................................................ucagcaugguaggaaugu........................  3       0    S03
    ......................................................................ucagcaugguaggaauguauuu....................  12      0    S03
    ......................................................................ucagcaugguaggaauguauuuu...................  4       0    S03
    ......................................................................ucagcaugguaggaauguauuuua..................  1       0    S03

    ......................................................................ucagcaugguaggaaugu........................  3       0    S09
    ......................................................................ucagcaugguaggaauguauu.....................  1       0    S09
    ......................................................................ucagcaugguaggaauguauuu....................  5       0    S09
    ......................................................................ucagcaugguaggaauguauuuu...................  4       0    S09
    ......................................................................ucagcaugguaggaauguauuuua..................  1       0    S09

    ......................................................................ucagcaugguaggaauguauu.....................  1       0    S08
    ......................................................................ucagcaugguaggaauguauuu....................  2       0    S08

    ......................................................................ucagcaugguaggaaugu........................  1       0    S06
```
[truncated: 573,198 more chars]
