# Supplementary figures and images for: Pervasive microRNA Duplication in Chelicerates: Insights from the Embryonic microRNA Repertoire of the Spider Parasteatoda tepidariorum
Source: Genome Biol Evol. 2016 Jun 19;8(7):2133–44. doi: 10.1093/gbe/evw143 (PMC4987109; doi:10.1093/gbe/evw143)

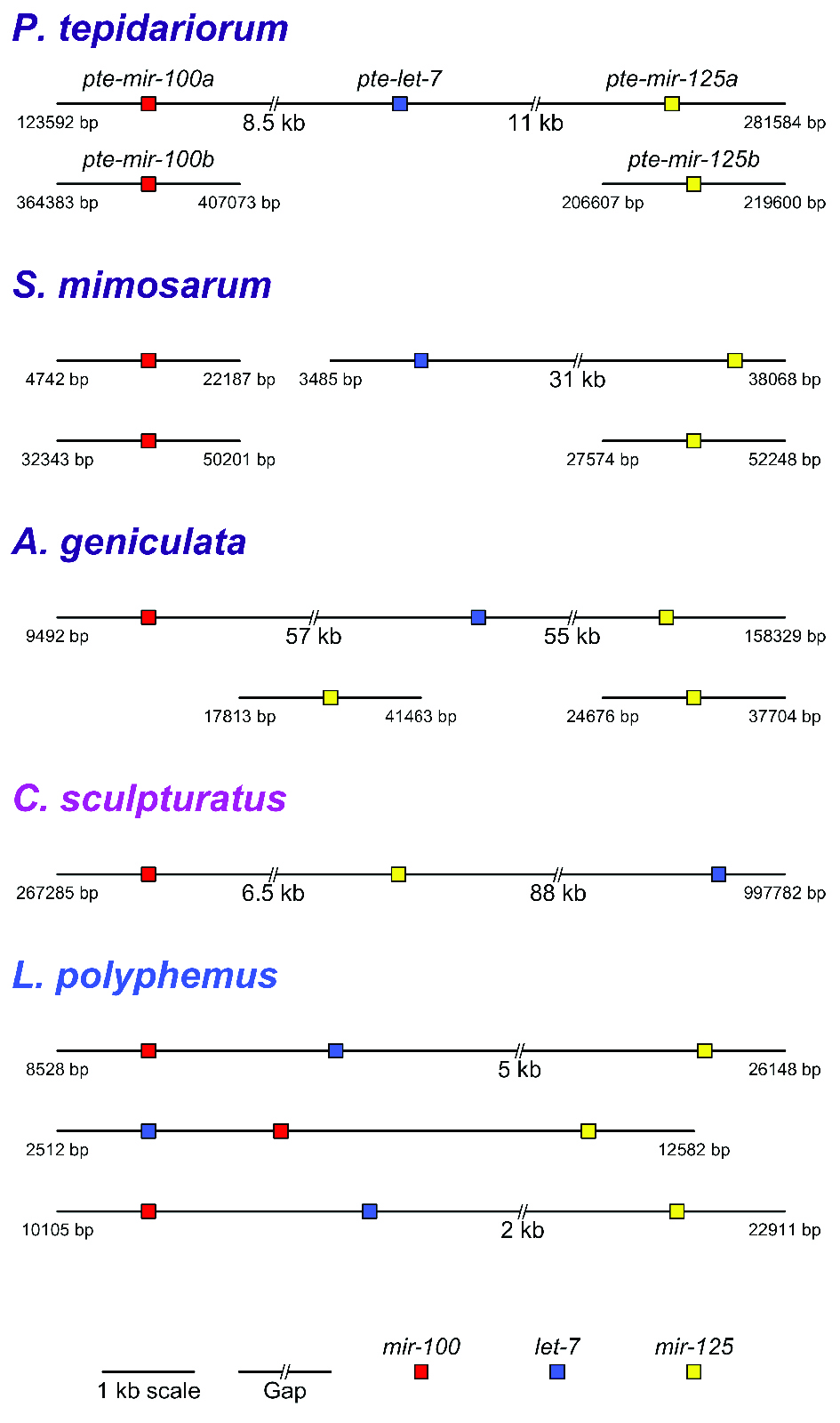

Supplement: Supplementary Data [file gbe_8_7_evw143_s1.zip › Sup Fig 4.jpg]

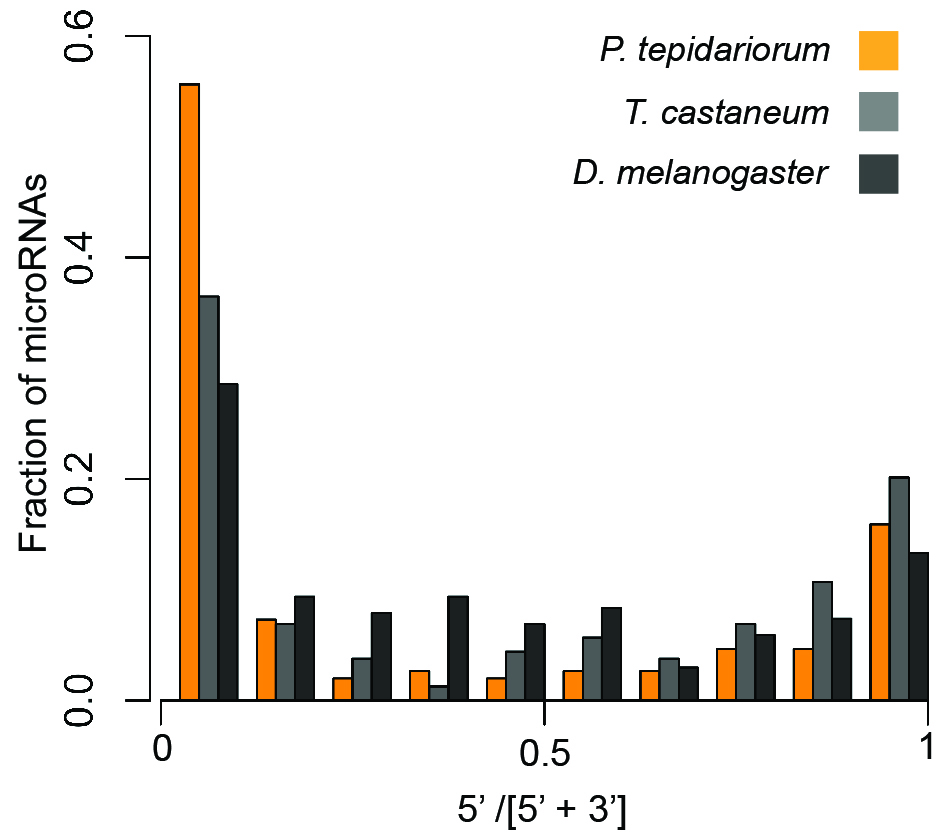

Supplement: Supplementary Data [file gbe_8_7_evw143_s1.zip › Sup Fig 5.jpg]

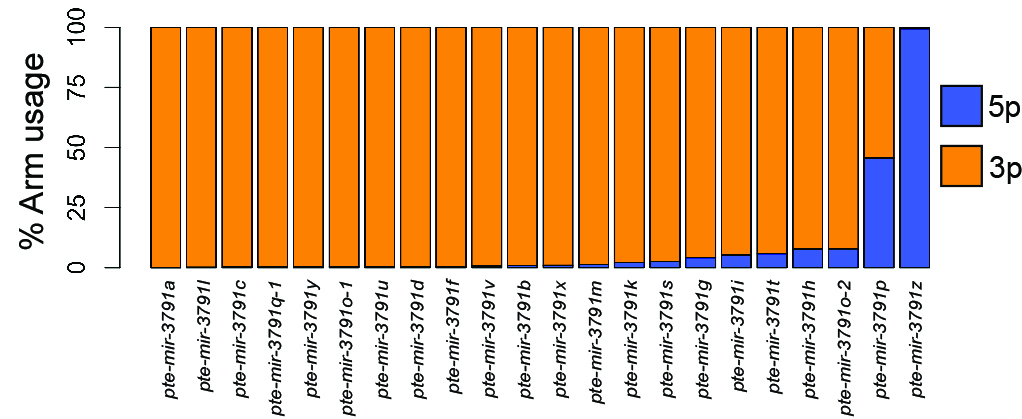

Supplement: Supplementary Data [file gbe_8_7_evw143_s1.zip › Sup Fig 6.jpg]

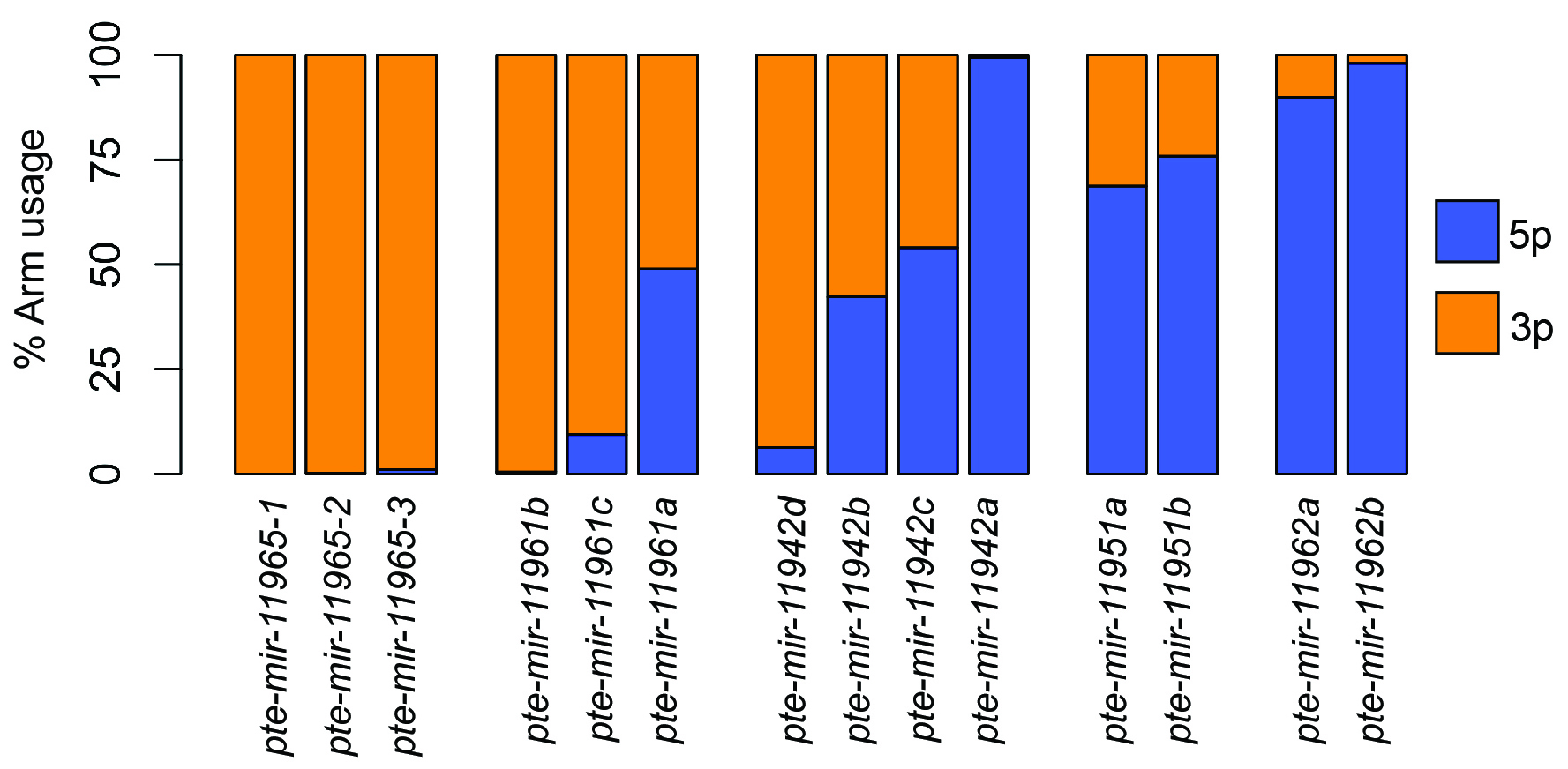

Supplement: Supplementary Data [file gbe_8_7_evw143_s1.zip › Sup Fig 7.jpg]

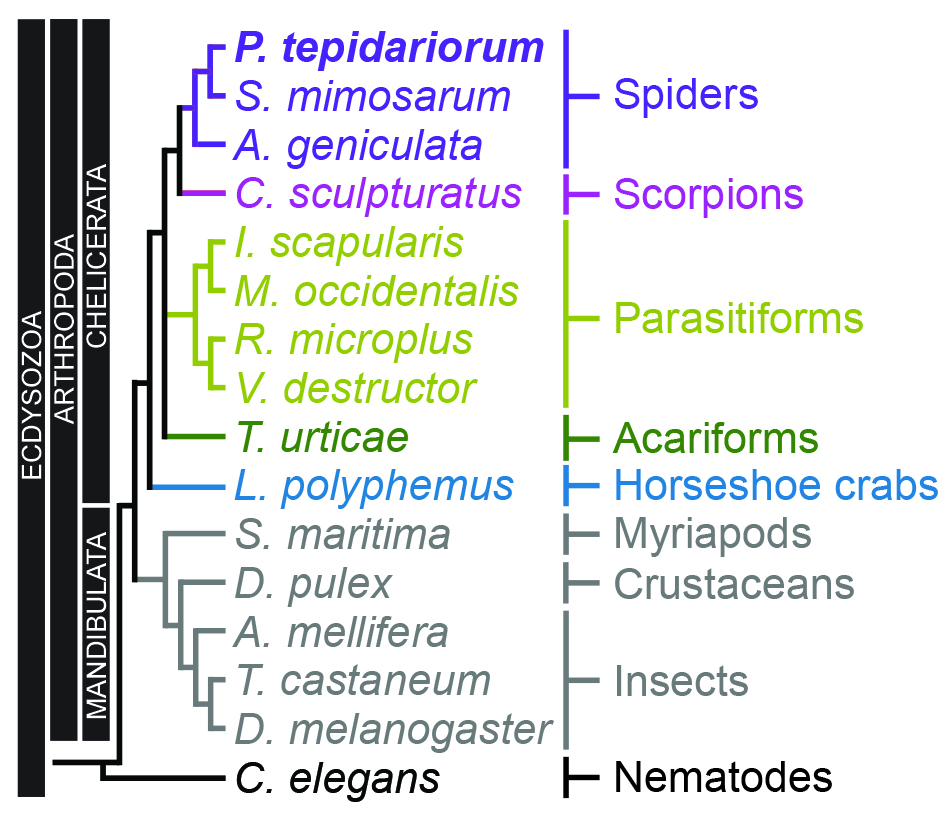

Supplement: Supplementary Data [file gbe_8_7_evw143_s1.zip › Sup Fig 1.jpg]

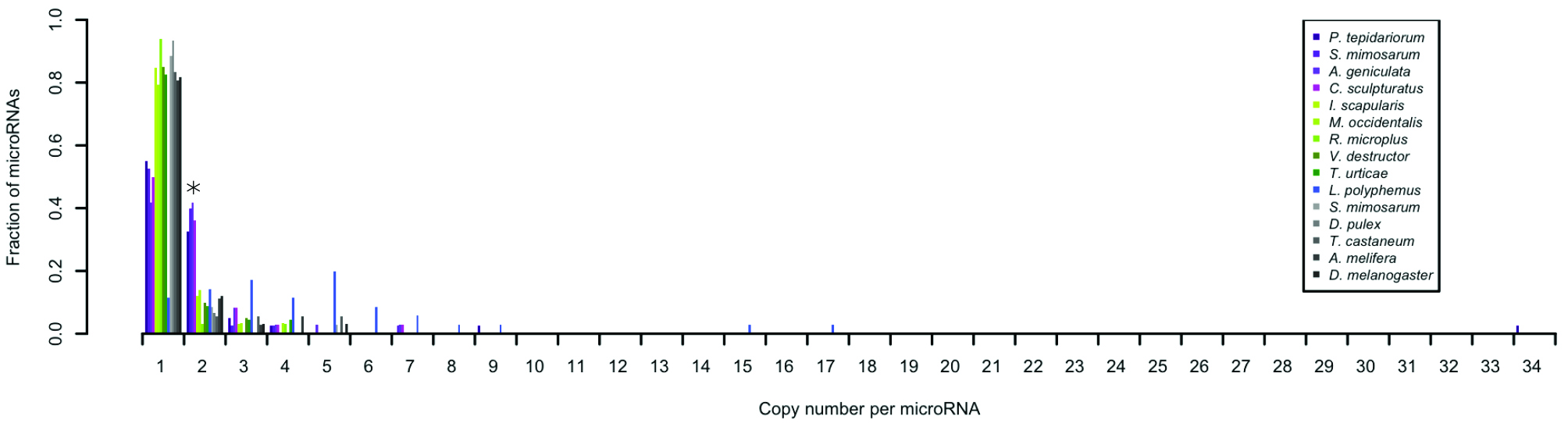

Supplement: Supplementary Data [file gbe_8_7_evw143_s1.zip › Sup Fig 2.jpg]

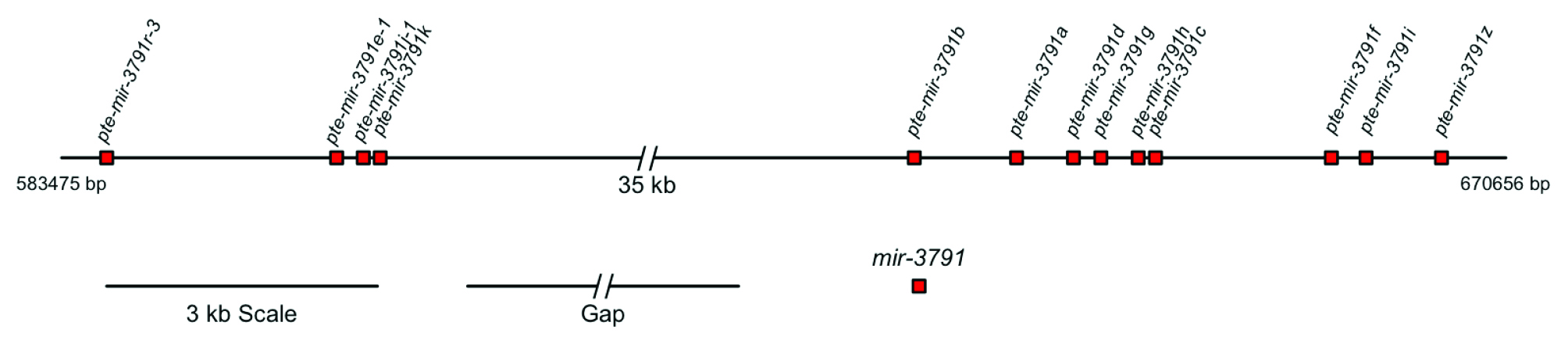

Supplement: Supplementary Data [file gbe_8_7_evw143_s1.zip › Sup Fig 3.jpg]
